# InDePTH: detection of hub genes for developing gene expression networks under anticancer drug treatment

**DOI:** 10.18632/oncotarget.25624

**Published:** 2018-06-26

**Authors:** Masaru Koido, Yuri Tani, Satomi Tsukahara, Yuka Okamoto, Akihiro Tomida

**Affiliations:** ^1^ Cancer Chemotherapy Center, Japanese Foundation for Cancer Research, 3-8-31 Ariake, Koto-ku, Tokyo 135-8550, Japan

**Keywords:** drug-induced gene expression change, transcriptome, network analysis, data mining, LINCS

## Abstract

It has been difficult to elucidate the structure of gene regulatory networks under anticancer drug treatment. Here, we developed an algorithm to highlight the hub genes that play a major role in creating the upstream and downstream relationships within a given set of differentially expressed genes. The directionality of the relationships between genes was defined using information from comprehensive collections of transcriptome profiles after gene knockdown and overexpression. As expected, among the drug-perturbed genes, our algorithm tended to derive plausible hub genes, such as transcription factors. Our validation experiments successfully showed the anticipated activity of certain hub gene in establishing the gene regulatory network that was associated with cell growth inhibition. Notably, giving such top priority to the hub gene was not achieved by ranking fold change in expression and by the conventional gene set enrichment analysis of drug-induced transcriptome data. Thus, our data-driven approach can facilitate to understand drug-induced gene regulatory networks for finding potential functional genes.

## INTRODUCTION

Comparative gene expression analysis defines differentially expressed genes (DEGs) under certain conditions of interest. To interpret DEGs from biological aspects, they have been compared with gene sets from curated databases of molecular functions [1–3]. In the field of biomedical research, the connectivity map (CMap) team developed a transcriptome database, composed of five human cell lines treated with 1309 small compounds [4, 5]. We have also constructed a transcriptome database focusing on anticancer compounds and related compounds, mainly using colon adenocarcinoma HT-29 cells [6, 7]. These drug-induced transcriptome databases are useful as reference databases of gene expression change. However, further prior knowledge and summarizing techniques are required to extract underlying biological information from these gene expression signatures [8].

Recently, the Library of Integrated Network-Based Cellular Signatures (LINCS) program (National Institutes of Health, USA) initiated an effort to generate a variety of biomedical big data [9]. In particular, the LINCS L1000 project has developed the high-throughput L1000 platform [9] and measured the expression of 978 landmark genes under 1.3 million cell conditions, consisting of compound treatments (multiple doses) and genetic perturbation treatments (knockdown by shRNA, overexpression, and ligand treatment) at multiple time points in several different cell lines [10]. 978 landmark genes were determined as informative genes from multivariate analysis using 12063 public transcriptome microarray data catalogued in the Gene Expression Omnibus [10]. Furthermore, based on measured expression levels of landmark genes, the expression levels of ∼21,000 unmeasured genes were inferred by a linear regression model, in which the weight coefficient was estimated from the substantial transcriptome data [10, 11].

In addition to the expansion of gene expression databases, bioinformatic methodologies are also required for linking different databases and extracting interpretable information from them. Subramanian et al. developed the Gene Set Enrichment Analysis (GSEA) methodology to evaluate the enrichment of gene sets in genes with increased or decreased expression ranked by user-prepared transcriptome data [12, 13]. Based on the concept of this enrichment analysis, the CMap team developed a pattern-matching algorithm (CMap algorithm) to search which conditions in the CMap database induce the pattern of gene expression change similar to the pattern in the user-prepared list of DEGs [4]. Currently, the CMap algorithm is widely accepted in the biomedical field [14, 15] and has contributed to biological interpretation of the activities of drugs [6, 16, 17].

Thus, the methodology of enrichment analysis has succeeded in interpreting the overall biological effects of a set of drug-induced DEGs, and thus the expansion of genetic perturbation data in LINCS is promising for providing further deep insights into DEGs. However, it remains a major challenge to interpret how a hierarchical network among DEGs was developed and which DEGs played a central role in this development. To address this, we defined an influential gene as one whose increased or decreased expression level centrally mediates the change of expression levels of many other genes. Herein, to find influential genes from among DEGs, we developed the influential gene detection in perturbed transcriptome hierarchical network (InDePTH) methodology. InDePTH is a novel algorithm to detect hubs of influential genes from reconstructed upstream and downstream relationships among DEGs (user-prepared, query DEGs), by referring to the rank matrix of Z-scores from a database of comprehensive genetic perturbations, such as the LINCS L1000 dataset (publicly available, reference data). The application of the InDePTH method could be effective in identifying influential genes from among DEGs under anticancer drug treatment.

## RESULTS

### Development of the InDePTH methodology

**Figure 1:**
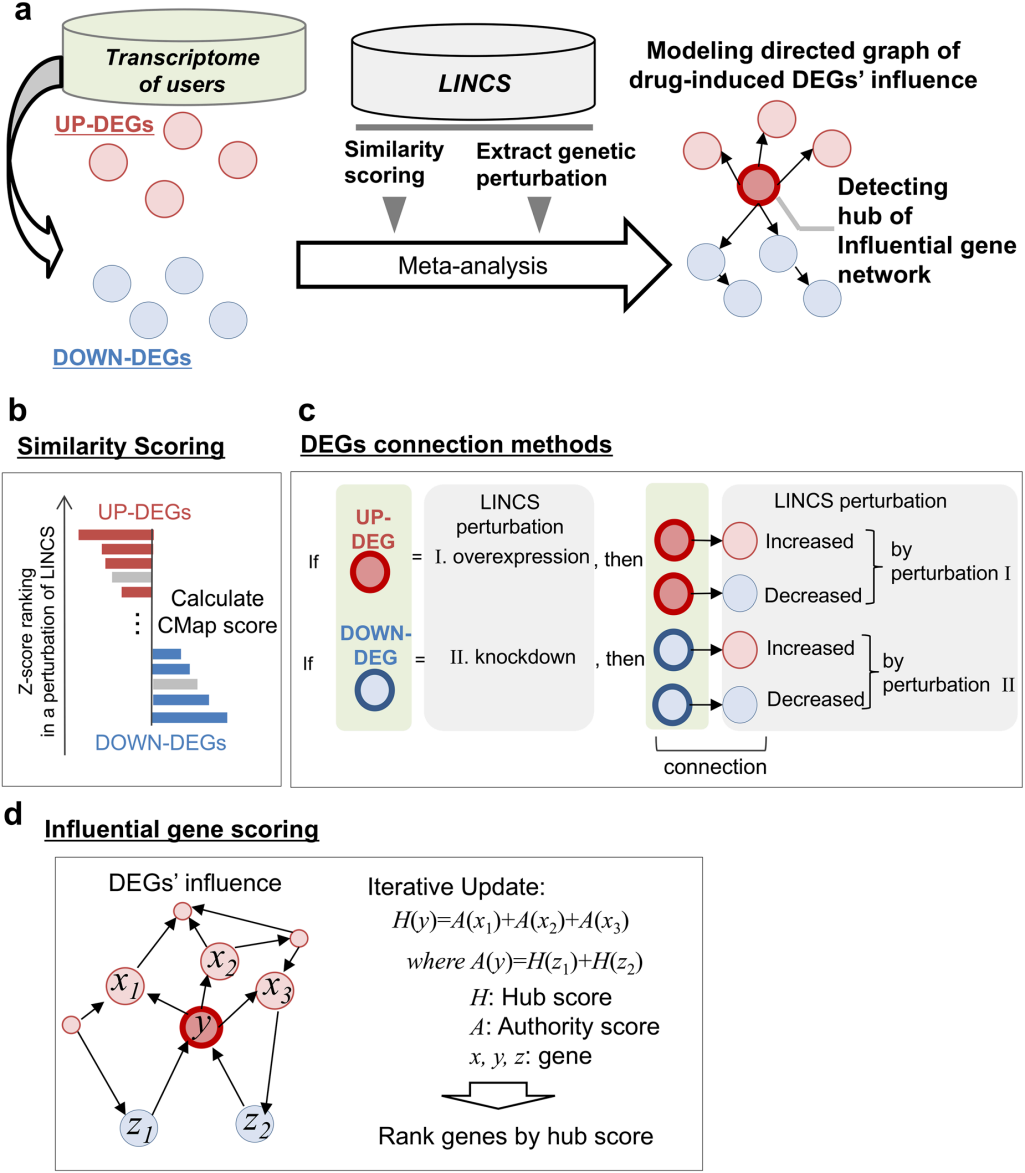
Overview of InDePTH algorithm **(a)** Overview of InDePTH methodology. Drug-induced DEGs (query DEGs) and LINCS gene expression perturbation database (reference data, high-throughput gene expression DB) were used for creating a directed graph of DEGs and subsequent detection of influential genes. **(b)** Similarity score calculation in InDePTH. **(c)** DEG connection method. Query DEG-related perturbations were selected from reference data using the following two criteria: 1) a record that showed a similarity score greater than the best cut-off point was selected, and 2) a record that showed a match in the direction of gene expression change between query DEGs and reference data was filtered. **(d)** Scoring method for influential genes [18].

InDePTH involves four steps for the identification of influential genes from among query DEGs (Figure 1a). First, it calculates similarity scores between patterns of query DEGs and those of perturbed genes from each of the genetic perturbations in LINCS, using the CMap algorithm [4] (Figure 1b). Second, if these similarity scores are above the predetermined cut-off point and if a gene subjected to the genetic perturbation satisfies the condition that the direction of change of its expression due to the perturbation is the same as that of the query DEGs, the gene is selected as an upstream gene. Third, InDePTH searches for downstream genes (genes whose expression change by an upstream gene perturbation is significant (z-score ≥ 2 or ≤ −2), as recorded in LINCS) whose direction of change in expression is the same as that of the query DEGs, and then upstream and downstream genes are connected by arrows (Figure 1c). Finally, from the hierarchical network of DEGs with connections by arrows (i.e. directed graph model), InDePTH mines the hub of upstream genes that play central roles in developing the gene network, using a data-mining algorithm for the complex world wide web to discover information sources and hubs that join the sources [18] (Figure 1d). For each of the query DEGs, a hub score is obtained within the range of 0 to 1, in which a DEG with a hub score = 1 is the most highly influential gene among the query DEGs, and the hub scores of other genes are values relative to the score of the highest one.

### Optimization of the InDePTH parameters

**Figure 2:**
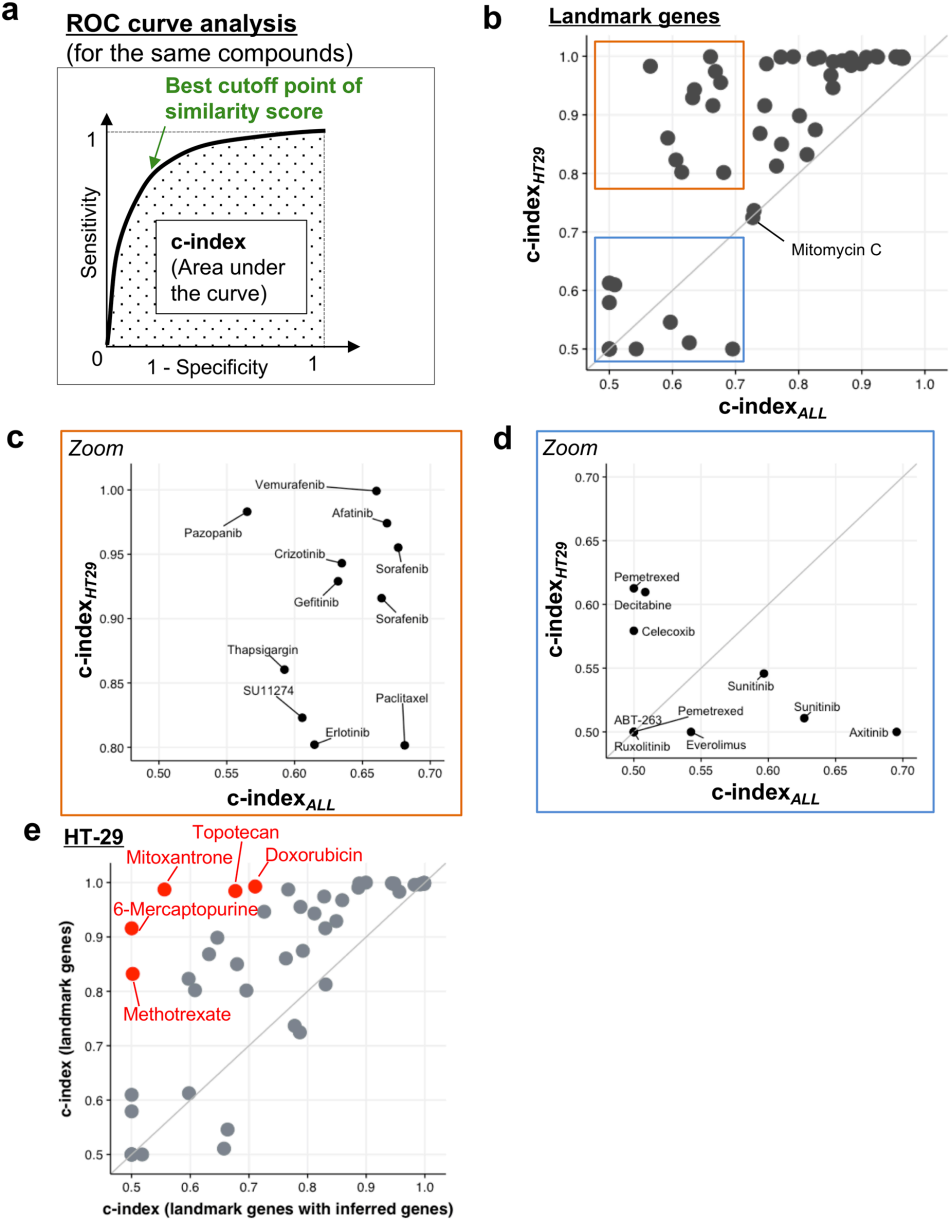
Identification of comparable conditions in InDePTH algorithm **(a)** Method for evaluating sensitivity and specificity, and the best cut-off point of similarity score. See also Materials and Methods. **(b–d)** A comparison of the c-index between one from the 1.3 million LINCS dataset [x-axis, c-index_*ALL*_] and one from the HT-29 LINCS dataset [y-axis, c-index_*HT29*_]. The area representing high c-index_*HT29*_ but low c-index_*ALL*_ is enlarged in (c). The area corresponding to a low c-index in both conditions is enlarged in (d). **(e)** A comparison of the c-index between only landmark genes (y-axis) and landmark genes with inferred genes (x-axis). Red plot indicates that the difference of c-index is statistically significant (*P*-value<9.6×10^−4^, Bonferroni-corrected, *n*=52). See also Supplementary Figures 1 and 2.

In the InDePTH algorithm, a critical tuning parameter for refining hierarchical network structure is the cut-off point of the CMap similarity score, but no method is available for determining the threshold of the score from a rank matrix obtained by ordering the Z-scores of the reference LINCS L1000 dataset. Thus, we measured the sensitivity and specificity of the similarity score calculated from the DEGs of HT-29 cells treated with anticancer compounds, obtained from a previously developed transcriptome database [6, 7] (Supplementary Table 1). Here, area under the receiver operating characteristic (ROC) curve (i.e. concordance index: c-index) was calculated by regarding the same drug treatment conditions as positive and the others as negative when assessing the similarity to experimental conditions that should substantially be the same between reference and query DEGs (Figure 2a). We first used 978 landmark genes for calculating the CMap similarity score and compared two types of calculation method for c-index: one that used all of the LINCS’ 1.3 million perturbations, including all of the cell lines contained in the LINCS database [i.e. c-index_*ALL*_], and the other that used perturbations for only HT-29 cells to consider the effect of cellular context on the origin of the query DEGs [i.e. c-index_*HT29*_]. Interestingly, both c-indexes for many compounds showed moderate accuracy (c-index>0.7) [19], despite only 978 genes having been used for the similarity scoring (Figure 2b). In the area corresponding to moderate accuracy for both c-indexes, each c-index_*HT29*_ of almost all compounds was higher than the corresponding c-index_*ALL*_, except for the case of mitomycin C (Figure 2b). In the area with poor accuracy for the c-index_*ALL*_ <0.7, each c-index_*HT29*_ of many tyrosine-kinase inhibitors was higher than the corresponding c-index_*ALL*_ (Figure 2c), but not for the cases of sunitinib and axitinib (Figure 2d). Thus, when selecting cut-off points, it is important to consider the cellular context.

Next, we investigated whether the expression data of ∼21,000 genes inferred from 978 landmark genes can improve the accuracy of the similarity score. Calculating the similarity score, however, revealed that including inferred expression data decreased c-index_*HT29*_ (Figure 2e), as well as c-index_*ALL*_ (Supplementary Figure 1), for many drugs. Notably, each c-index_*HT29*_ was also higher than the corresponding c-index_*ALL*_, even when inferred expression data were also used for calculating similarity scores (Supplementary Figure 2). Taking these findings together, in the InDePTH analysis, CMap similarity scores from only landmark gene sets were preferred. It is noted that, even in the area corresponding to high accuracy (c-index>0.9) [19] of the optimized conditions, the best cut-off point led to the CMap similarity score being distributed in the range of >0.2 (Supplementary Figure 3). Thus, an arbitrary threshold >0.2 can be acceptable when an appropriate cut-off threshold of the similarity score cannot be determined by ROC curve analysis.

### Validation of the InDePTH analysis

**Figure 3:**
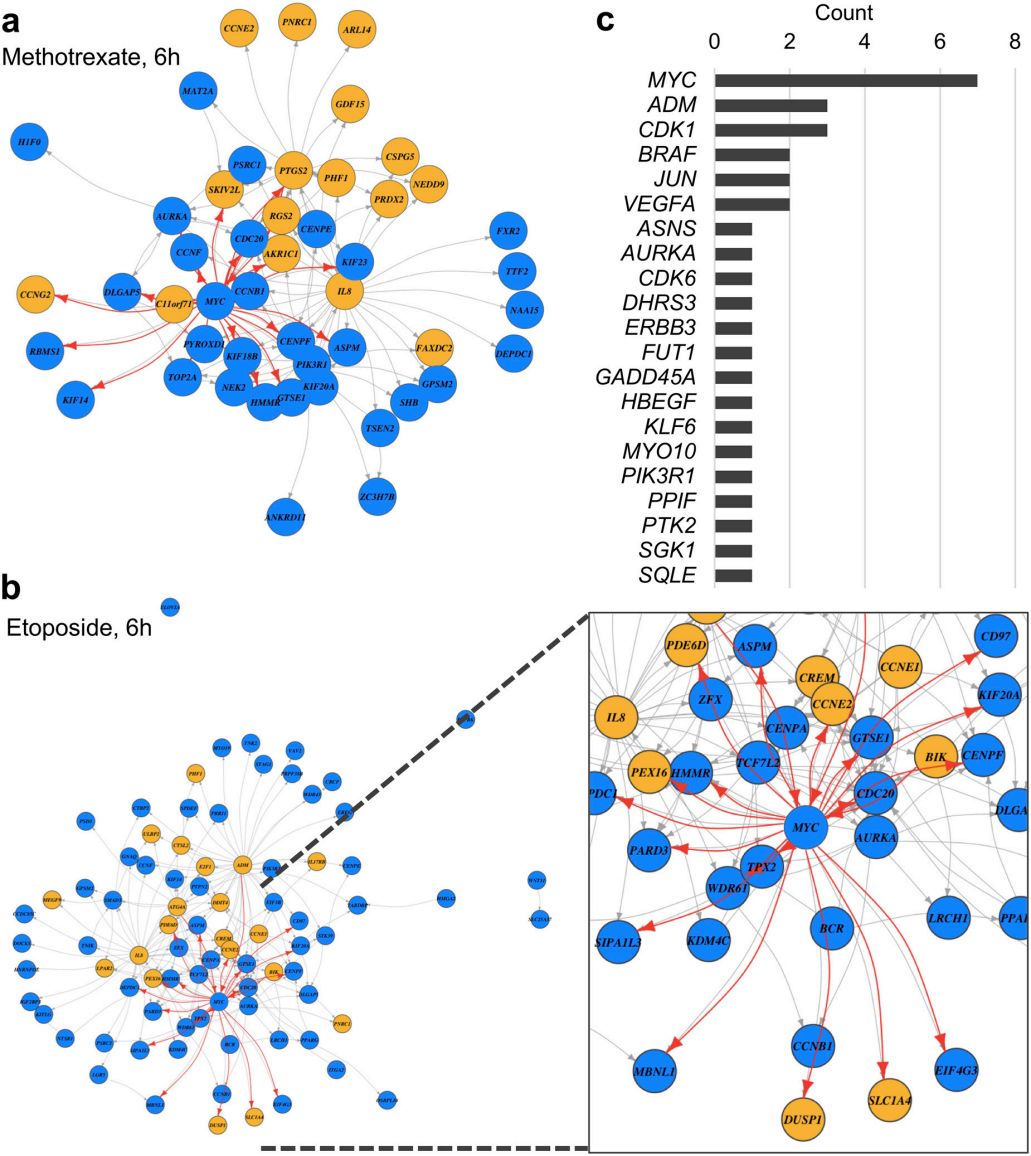
Prediction of drug-induced gene expression network **(a, b)** Constructed DEG directed graph. Yellow circles represent UP-DEGs and blue circles represent DOWN-DEGs from among the query DEGs. Each arrow indicates a direction of upstream and downstream relationships. Arrows connecting to MYC are highlighted by red. DEGs without an arrow mean that the upstream gene is over the cut-off value, but no downstream gene shows the same direction of gene expression change between query DEGs and reference data. **(c)** Counts of most highly influential genes (see Supplementary Table 2).

To evaluate whether InDePTH can reliably select hubs of influential genes, we closely examined its results using compounds showing moderate accuracy from c-index_*HT29*_. First, directed graphs modelling upstream and downstream relationships among DEGs were successfully created (Figure 3a and 3b), but in many cases, the graphs were too complex to interpret (Supplementary Figure 4), suggesting the importance of the scoring system for the hub in the data-mining algorithm. The most highly influential gene for each of the drug-induced DEGs, which was determined by the hub score, is shown in Supplementary Table 2, and relatively highly influential genes are shown in Supplementary Table 3. The most highly influential genes included transcription factors, such as v-myc myelocytomatosis viral oncogene homolog (*MYC*), in the conditions of 6-h treatments with methotrexate, mitomycin C, mitoxantrone, etoposide, and U-0126, and 16-h treatments with gemcitabine, methotrexate, and etoposide; jun proto-oncogene (*JUN*) in the conditions of pazopanib and SB218078 treatment; and Kruppel-like factor 6 (*KLF6*) in the condition of BEZ235 treatment (Figure 3c and Supplementary Table 2). Thus, it is likely that InDePTH could prioritize influential genes from potential upstream genes including those encoding transcription factors under drug treatment. It is notable that no genetic perturbation was selected in the analysis of some compounds, such as bortezomib and vemurafenib, due to the extremely high cut-off point of the similarity score (Supplementary Table 1).

**Figure 4:**
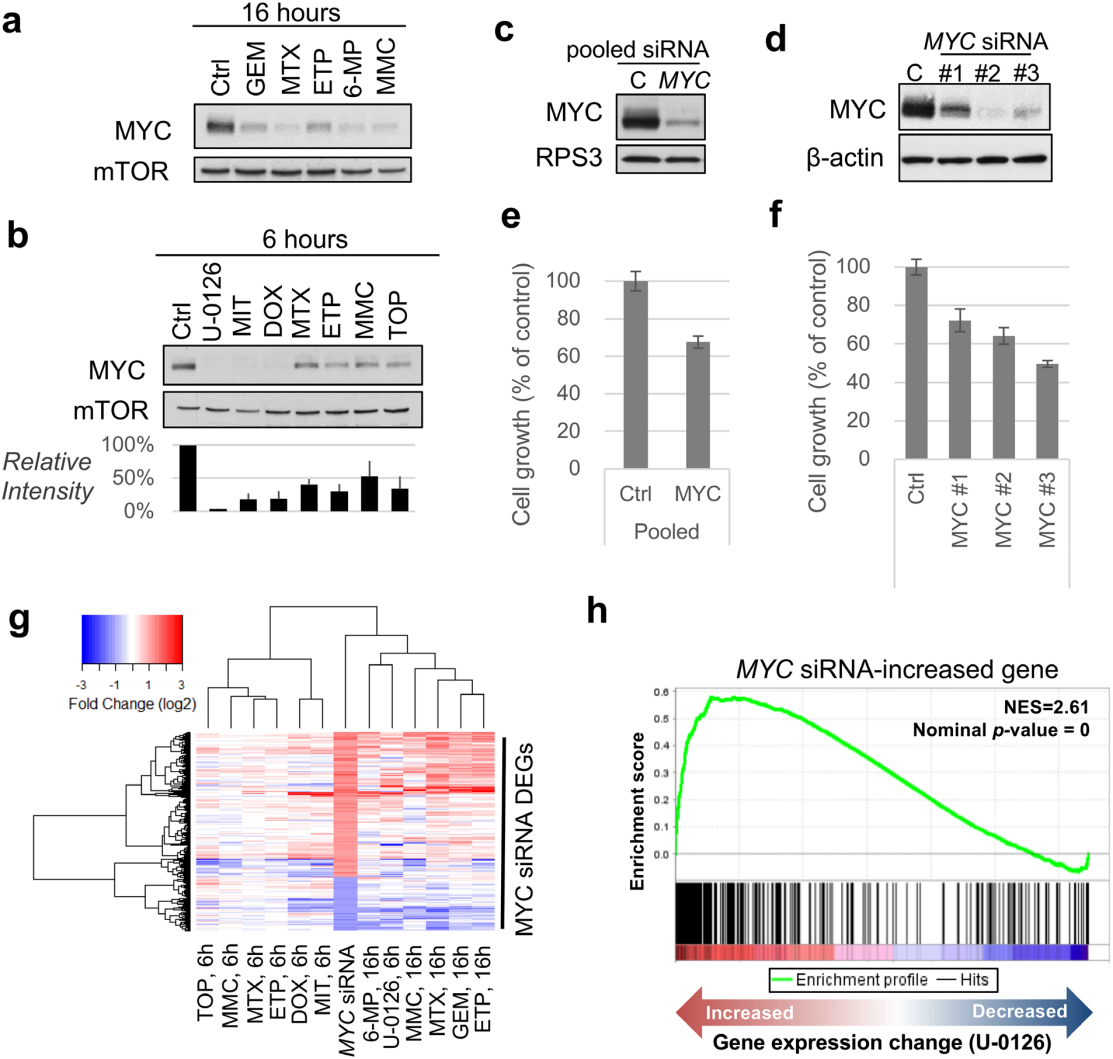
*MYC*, one of the most influential genes, accounts for the drug-induced change in gene expression **(a, b)** Immunoblot analysis of MYC under (a) 16-h treatment and (b) 6-h treatment of HT-29 cells with the indicated compounds. mTOR was used as a loading control. Blot intensities of MYC relative to those of mTOR (n=3 independent experiments, mean ± SD) are shown (b, below). The drug concentrations were the same with the description in Supplementary Table 1. **(c, d)** Immunoblot analysis of MYC upon treatment with *MYC* siRNAs. RPS3 and β-actin were used as a loading control. **(e, f)** Cell growth assay after treatment with MYC siRNAs. ON-TARGETplus SMART pool siRNA was used (in c, e) and Silencer Select Pre-designed siRNAs were used (in d, f). **(g)** Hierarchical clustering analysis of indicated conditions using DEGs of MYC siRNA. **(h)** Enrichment plot using *MYC* siRNA-increased gene sets. Running enrichment score (*top portion, green curve*) and the statistics were calculated from the order of gene sets based on the gene expression change (*bottom*) upon treatment with U-0126. GEM, gemcitabine; MTX, methotrexate; ETP, etoposide; 6-MP, 6-mercaptopurine; MMC, mitomycin C; TOP, topotecan; DOX, doxorubicin; MIT, mitoxantrone.

To conduct further validation of InDePTH by *in vitro* study, we focused on *MYC* because InDePTH analysis showed *MYC* to be the most highly influential gene for many conditions for the query DEGs (Figure 3c), and because the curated *MYC* target signatures [3] may be useful for unbiased comparison. We investigated the role of *MYC* in the transcriptome change associated with the compounds showing relatively high hub scores (>0.01), most of which were DNA damaging agents (Supplementary Table 4). Notably, in all conditions of compound treatment, the rank of *MYC* expression change was about 4000^th^–5000^th^ place (top 8%), in ascending order among the genes measured by the microarray (Supplementary Figure 5), suggesting that InDePTH could evaluate the transcriptome data from a perspective other than the degree of change in gene expression. Associated with these *MYC* expression changes, MYC protein levels were decreased by 16-h treatments with gemcitabine, methotrexate, etoposide, 6-mercaptopurine, and mitomycin C (DNA damaging agents) (Figure 4a), and by 6-h treatments with U-0126 (MEK inhibitor), mitoxantrone, doxorubicin, methotrexate, etoposide, mitomycin C, and topotecan (DNA damaging agents) (Figure 4b).

**Figure 5:**
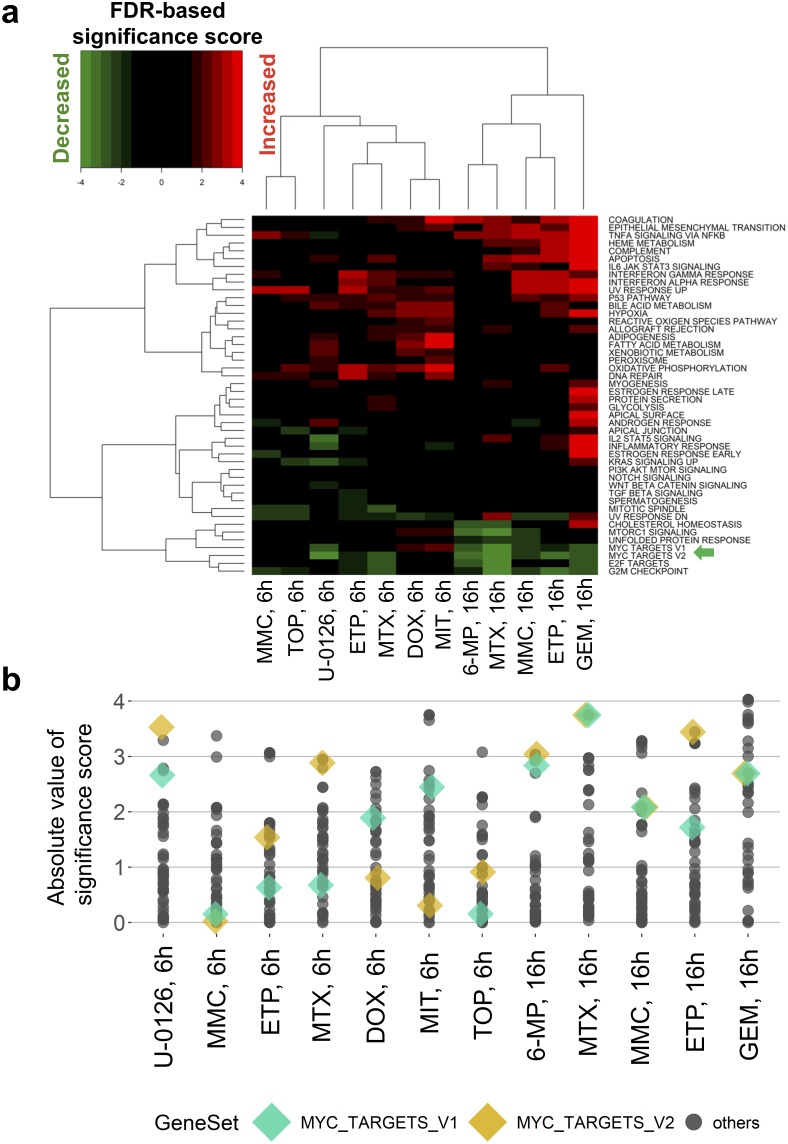
Conventional enrichment analysis entirely prioritized gene sets other than MYC target signatures **(a, b)** GSEA analysis of hallmark signatures of gene sets. (a) False discovery rate (FDR)-based significance scores (see Materials and Methods) of the indicated drug treatments (*column*) were shown for each hallmark signature (*row*) and (b) absolute values. MYC_TARGETS_V1 and MYC_TARGETS_V2, gene sets defined as subgroups of genes regulated by MYC in the hallmark signature of gene sets [3], were highlighted by the green arrow in (a) and colored diamond shapes in (b). GEM, gemcitabine; MTX, methotrexate; ETP, etoposide; 6-MP, 6-mercaptopurine; MMC, mitomycin C; TOP, topotecan; DOX, doxorubicin; MIT, mitoxantrone.

We also found that the knockdown of *MYC* in HT-29 cells by siRNA treatment (Figure 4c and 4d) decreased cell growth (Figure 4e and 4f) and the expression levels of genes from curated *MYC* target signatures as well (Supplementary Table 5). As expected, gene expression patterns under *MYC* siRNA treatments were similar to those under 16-h and 6-h treatments of DNA damaging agents (Figure 4g). In general, *MYC* knockdown-associated DEGs, which would include both primary and secondary transcription targets of MYC, were significantly enriched in the genes whose expression increased and decreased under treatment with the above compounds (Figure 4h, and Supplementary Figures 6 and 7). Notably, GSEA using hallmark signatures of gene sets [3] confirmed that the curated *MYC* target signatures were enriched in genes whose expression decreased under the drug treatments (Figure 5a), but many other signatures showed stronger significance than the *MYC* signatures (Figure 5b). This comparison indicated that InDePTH can detect the influence of *MYC* on other DEGs, in a different way from the conventional enrichment analysis.

**Figure 6:**
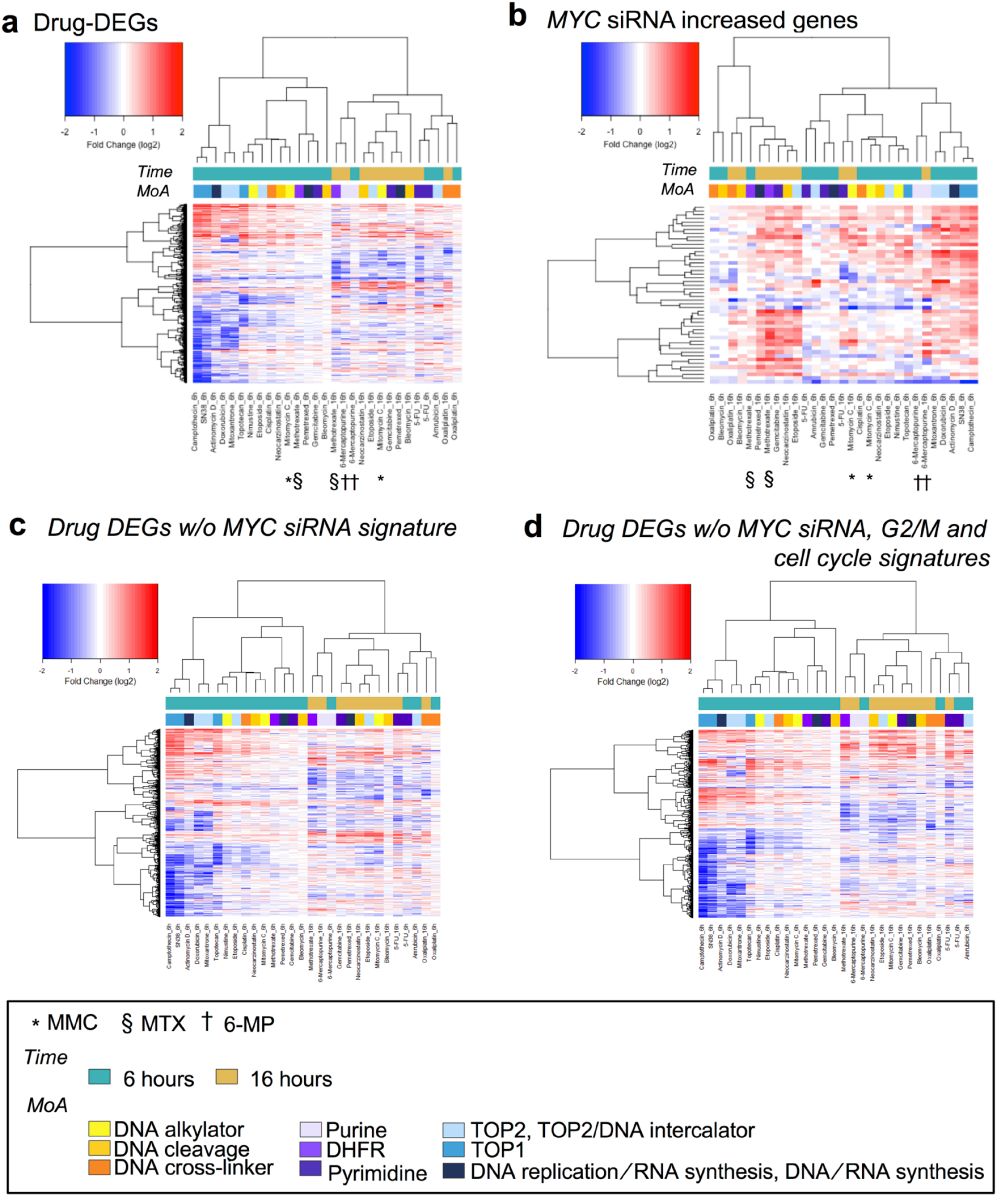
*MYC* downstream genes enabled DNA-damaging agents with the same MoA to cluster closer together Hierarchical clustering and heatmap. The used gene sets were as follows: **(a)** DEGs for each indicated drug, **(b)** *MYC* siRNA-increased DEGs, **(c)** DEGs for each indicated drug but without *MYC* siRNA-increased/decreased DEGs, and **(d)** DEGs in each indicated drug but without both *MYC* siRNA-increased/decreased DEGs and G_2_/M and cell cycle gene sets [3]. The legends for the coloured boxes are shown at the bottom. Drug MoA was obtained from reference #[7].

Our previous study indicated that data from 16-h treatment with DNA damaging agents tended to cluster together, despite these agents having different mechanisms of action (MoA) [6]. We found that the genes whose expression increased in association with *MYC* siRNA enabled DNA damaging agents with the same MoA to cluster closer together, especially for compounds targeting nucleic acid metabolism (Figure 6a and 6b). Interestingly, the pattern of hierarchical clustering retained the pattern of the original clustering, despite the *MYC* siRNA-induced DEGs (Figure 6c) and known cell cycle signature of gene sets (Figure 6d) having been removed from the original DNA damaging agent-induced DEGs. Consistent with these findings, a great number of genes were required for fully constructing the gene expression networks induced by the DNA damaging agents; however, a set of genes with high hub scores explained most of these complex structures (Supplementary Figures 8 and 9). Collectively, most of the drug-induced DEGs were derived from both primary and secondary effects of the drug treatments, and these effects can be distinguished by InDePTH.

We further analysed public transcriptome datasets including 14 compounds treatments on diffuse large B cell lymphoma cells (OCI-LY3) [20] by InDePTH and compared the result with an algorithm named detecting mechanism of action by network dysregulation (DeMAND), which prioritizes proteins whose interactions (such as protein-protein interactions) could be perturbed by drug treatments [21]. We successfully selected query DEGs from the datasets of 14 compounds (FDR <.10; no DEG in aclacinomycin A and geldanamycin; too small DEGs for InDePTH analysis in blebbistatin and vincristine). In general, the rank of genes from InDePTH and DeMAND analysis did not correlate (Supplementary Figure 10). Especially for DNA damaging agents including camptothecin, doxorubicin, etoposide and mitomycin C, the commonly prioritized genes in InDePTH (hub score >.01 and top 20) were *MYC* and polo-like kinase 1 (*PLK1*). Interestingly, PLK1 was also predicted by DeMAND as an effector protein for the drug perturbations [21]. The other effector proteins from DeMAND analysis were not the commonly prioritized in InDePTH analysis at mRNA levels, such as DNA damage-inducible gene 45A (*GADD45A*), cyclin-dependent kinase inhibitor 1A (*CDKN1A*), proliferating cell nuclear antigen (*PCNA*), cyclin B1 (*CCNB1*) and Aurora Kinase A (*AURKA*) [21] (Supplementary Figure 10). However, of interest was that InDePTH analysis could show potential hierarchical relationships of these genes for each agent (Supplementary Figure 11).

Finally, we performed InDePTH analysis of hypoxia-responsive genes whose expression levels were increased and decreased depending on mitochondrial functions [22]. InDePTH reconstructed the gene regulatory network and interestingly showed that the gene with the highest hub score was NADH:Ubiquinone Oxidoreductase Complex Assembly Factor 4 (*NDUFAF4*), an assembly factor for mitochondrial complex I [23] (Supplementary Figure 12).

## DISCUSSION

InDePTH is a novel semantic algorithm for linking DEGs to each other according to their influence on the expression levels of other genes. It then reconstructs hierarchical network models of upstream and downstream relationships among the DEGs. InDePTH is also equipped with a data-mining program for hub detection and can rank DEGs by their influence in a gene expression network. Indeed, InDePTH revealed that one of the genes whose expression had the greatest influence on the expression levels of many other genes upon exposure to many anticancer compounds was *MYC*, whose contribution was masked by other DEGs in conventional signature-based enrichment analysis. Taking these findings together, InDePTH is a powerful algorithm for creating networks of DEGs and focusing on the hubs of such networks. A package for implementation of the InDePTH algorithm in the software environment R is now available to the research community at the GitHub repository (https://github.com/koido/InDePTH).

InDePTH provides upstream and downstream relationships in network analysis. In general, upstream and downstream relationships in gene regulatory network have been provided from text mining-based approaches [24, 25], while the information from text mining is limited, partly because the names of genes are often not standardized and partly because it is also difficult to distinguish between genes and proteins in the literature [26]. In contrast to text mining-based approaches, InDePTH can utilize experimentally verified information about the upstream and downstream relationships of numerous genes, stored in the massive database LINCS. Similar to InDePTH, DeMAND also uses drug-induced transcriptome data and can shed light on the role of protein-coding genes in drug MoA, even when the extent of their change in expression is not significant [21]. Interestingly, a hub gene of DNA-damaging agents from InDePTH analysis overlapped with the DeMAND-identified effector protein for them, even though methods and overall results between the two methods were fundamentally different. In addition, InDePTH successfully showed the hierarchical relationships among mRNAs of DeMAND-identified effector proteins for the drug perturbations. Notably, for predicting the dysregulation of interacting proteins, DeMAND requires a minimum of six samples for both case and control samples [21], while InDePTH has no such limitation and requires only user-defined DEGs of any type. This advantage of InDePTH enables users to develop hypothesis even in the early stage of the research which in general collects minimum sample sets.

The direction of paths in the InDePTH-reconstructed network can be interpreted to represent the pseudo-time flow of gene expression change because these directions were determined based on the upstream and downstream relationships between perturbed genes after genetic perturbations. In fact, InDePTH detected *MYC* as the most highly influential gene upon treatment with methotrexate for 16 h, 10 h before which, the expression level of *MYC* was slightly decreased (Figure 4a, 4b). The same was true for mitomycin C treatments, whose hub score upon treatment for 6 h was the highest (Supplementary Table 2) and that upon treatment for 16 h was the second highest (Supplementary Table 3, Figure 4a, 4b). Therefore, InDePTH has one useful aspect of enabling the identification of genes whose expression can change at an early stage of drug treatment, without time-series data-based approach [27].

In many cases, the CMap similarity scores from 978 landmark genes were sufficient to analyse drug-induced DEGs by InDePTH. This indicates that the selection of landmark genes was preferable for expressing the features of drug-induced DEGs. However, a few compounds showed c-indexes of around 0.5, indicating that the true expression levels of genes other than landmark genes were required for InDePTH analysis in such cases. Unfortunately, we found that the current inferred expression levels could not address these limitations. Currently, LINCS makes inferences on genome-wide gene expression levels by a linear penalized regression model. For making inferences on gene expression, it may be necessary to include nonlinear effects (e.g. gene-to-gene interactions). One study already attempted to address this problem by applying a machine learning approach [11], and the LINCS team also has the aim of improving the inference accuracy by creating a cloud data analysis competition (http://crowdsourcing.topcoder.com/cmap, accessed on 16/4/2017). These approaches would lead to more accurate estimation of expression levels under drug and/or genetic perturbation in LINCS, which would also be promising for InDePTH in the future.

InDePTH analysis revealed *MYC* as a common influential hub gene, especially upon treatment with DNA damaging agents. Previous studies showed that the expression levels of *MYC* mRNA or MYC protein were reduced by methotrexate [28] and gemcitabine [29], while it depended on the cellular context whether the expression level of *MYC* increased or decreased upon exposure to etoposide [30–32]. In HT-29 cells, knockdown of *MYC* inhibited cell proliferation *in vitro* and *in vivo* [33], indicating that one of the basic characters of HT-29 cells depends on *MYC* expression. Similarly, suppression of *MYC* expression in OCI-LY3 cells is also considered to suppress the growth of the cells [34, 35]. Therefore, *MYC* can indeed be an influential gene, especially for HT-29 cells and OCI-LY3 cells treated with many anticancer compounds.

InDePTH was applicable to not only drug-induced DEGs but also other types of gene sets. Indeed, we successfully interpreted the mitochondria-dependent hypoxic responsive genes via the gene regulatory network and found that decreased expression of mitochondrial assembly factor *NDUFAF4* was the most influential in the network. Consistently, it has been reported that a missense mutation of *NDUFAF4* causes assembly defects of the mitochondrial complex I [36]. Thus, it is plausible to interpret *NDUFAF4* as an influential hub gene under the network.

Currently, InDePTH has two potential limitations. First, it strongly depends on the conditions catalogued in the LINCS database. For example, tissue-specific gene expression patterns [37] and genetic effects on gene expression [38] governs gene expression patterns as well as effects of oncogenes such as *MYC*. Therefore, when seeking the best cut-off point of CMap similarity score, the conditions of query DEGs would be preferable when the same reference data was obtained in terms of compounds and tissue origins of cell lines. To counter this limitation, we provided a reasonable cut-off range of a CMap similarity score >0.2 for hypothesis development by InDePTH (Supplementary Figure 3). In addition, as novel and low-cost methods for genome-wide transcriptome, such as pooled library amplification for transcriptome expression (PLATE-Seq) [39], have continuously developed, genome-wide transcriptome database of genetic perturbation in multiple cell types would expand more in future, leading to more reliable results from InDePTH. Second, as suggested by Figure 6c and 6d, it is the case that regulatory networks of gene expression under drug treatment might be due to conditions other than changes in expression levels, such as protein phosphorylation, degradation, and stabilization or non-coding RNAs [40]. Considering these potential limitations, it will be necessary in future to expand the reference database for dealing with more multiple cellular contexts and perturbations and incorporate other omics data. InDePTH source code is online available and therefore users can use reference database other than LINCS, and flexibly combine InDePTH algorithm with other omics tools, according to various purposes.

Taking the above findings together, InDePTH has been proven to be effective in identifying influential genes from among drug-induced DEGs, even when such influence was masked by many other signatures in conventional enrichment analysis. InDePTH should thus be useful to decipher the hierarchical networks of DEGs under anticancer drug treatment.

## MATERIALS AND METHODS

### LINCS L1000 dataset analysis

All data of LINCS L1000 were obtained from the Amazon S3 server, in which access keys were provided from lincscloud.org. L1000 gene expression data were obtained on 17/11/2014 (level 4 zspc data) and on 18/11/2014 (level 3 q2norm data), and the data description file (inst.info) was obtained on 13/11/2014. We defined the upregulated (downregulated) genes in LINCS using the threshold z-score ≥ 2 (≤ −2). If the item ‘pert_desc’ in inst.info was ‘−666’ and also the item ‘pert_type’ was ‘trt_cp’, we updated the inst.info file by merging with a chemical information file (downloaded on 24/2/2015). We manually confirmed that the names of compounds matched between LINCS and our database.

### In-house datasets and microarray analysis

The transcriptome dataset of anticancer compounds was obtained from our previous study [7]. In this study, we limited our analysis to only the dataset of HT-29 cells (see Supplementary Table 1 for detail conditions). Microarray analysis was conducted using GeneChip Human Genome U133 Plus 2.0 array (Affymetrix, Santa Clara, CA, USA), following standard protocols. Expression measurement was carried out using Affymetrix Microarray Suite version 5.0 from R package *affy* v1.40.0 [41]. Expression values were normalized to a mean target level of 100. Up- or downregulated genes (UP DEGs and DOWN DEGs, respectively) after exposure to the drug were determined as follows: For each treatment sample, we calculated treatment-to-control ratio statistics, where, if any intensity value was <50, the value was replaced by 50 [7], and we selected probe sets if the treatment-to-control ratio was ≥ 2 for UP DEGs or ≤ 0.5 for DOWN DEGs. Unsupervised hierarchical clustering was performed using the Pearson’s correlation distance and Ward’s linkage method. When performing network analysis, the average signal intensity ratio to the same gene was assigned. GSEA was performed with GSEA software (v2.0.14, Broad Institute) [12, 13] using the Molecular Signatures Database (MSigDB, v5.0) [13] or our defined signature gene sets. We set the false discovery rate (FDR) as 1 for gene sets, which means that the gene sets were not enriched at all. If the FDR of a gene set was 0, we set the FDR as the minimum FDR within each test. The FDR of gene sets was subjected to logarithmic transformation, and a positive or negative sign was used in front of this value if the gene set was enriched in genes with increased or decreased expression, respectively, under drug treatment. If there were both positive and negative scores due to marginal enrichment, we summed the two.

The transcriptome dataset for hypoxia-responsive genes were described in our previous paper [22]. From the hierarchical clustering of the hypoxia-responsive genes, mitochondria-dependent DEGs (Supplementary Figure 12a) were analyzed by InDePTH.

### Public transcriptome datasets

Normalized transcriptome data of 14 compounds on OCI-LY3 cells were obtained from NCBI Gene Expression Omnibus under the series accession no. GSE510681. Mapped genes were selected from R package hgu219.db v3.2.3 and the probe with the highest median of signal intensity for a gene was selected. T-statistics were calculated in log2 space by Welch’s two-sample t-test from all time points- and concentrations-aggregated datasets like DeMAND paper [21]. DEGs (FDR <.10) were assigned to probes of GeneChip Human Genome U133 Plus 2.0 array by hgu133plus2.db v3.2.3, in which using probes were limited to those with the median of signal intensity > 50 in the in-house datasets of anticancer compounds. Using these probes, InDePTH analysis was performed and the results were compared with the ranking of DeMAND analysis using U133p2 network (from a supplementary table in DeMAND paper [21]).

### Similarity scoring

CMap similarity scores were calculated from the CMap algorithm [4] using the R script described previously [6]. A reference rank matrix for the CMap algorithm was constructed from the LINCS gene expression database by ordering LINCS L1000 z-scores in descending order, in which, if z-scores had exactly the same values as the others, we set a higher rank for genes showing higher expression values. Using this LINCS rank matrix of 978 landmark genes or 22,268 inferred ones (including the 978 landmark genes), CMap similarity scores were calculated by using UP DEGs and DOWN DEGs obtained from the in-house dataset.

### ROC curve analysis

ROC curve analysis was conducted by regarding the drug treatment conditions of the same name as positive and the others as negative when assessing the similarity to experimental conditions that should substantially be the same between LINCS L1000 (reference data) and query DEGs. ROC curve analysis was performed using the R package *pROC* (version 1.7.3) [42]. When the c-index, area under the ROC curve, was <0.5, we set this value as 0.5 because c-index<0.5 means that there is no comparability between the two databases. Overall, 1,328,098 conditions were used for c-index_*ALL*_ and 113,867 HT-29 cell-specific conditions were used for c-index_*HT29*_. The Delong method [42] was used for calculating the *P*-value of the difference of c-index from the CMap similarity score from landmark genes and the CMap similarity score from landmark genes with inferred genes. Notably, we could not calculate the *P*-value between c-index_*HT29*_ and c-index_*ALL*_ because the number of genes used to create the ROC curve differed. The best cut-off of the CMap similarity score was determined at the point with the best sum of sensitivity and specificity, by using Youden’s J statistic [43].

### Network connection methods

To connect DEGs, perturbations of knockdown, overexpression, and ligand treatment (we refer to such perturbed genes as upstream genes) were selected if their CMap similarity scores were no less than the best cut-off point of the score. Upstream genes were further filtered using the following criteria: 1) if an upstream gene was knocked down in the reference data, the gene in the query DEGs must be DOWN DEG, and 2) if an upstream gene was overexpressed or treated with a ligand in the reference data, the gene must be UP DEG. If upstream genes with the same perturbation ID remained, the record with the highest CMap similarity score was used. Genes whose expression was increased or decreased by perturbation of upstream genes (referred to as downstream genes) were selected if the upstream genes significantly changed the expression of these downstream genes (LINCS Z-score>2 or Z-score<−2). When selecting knocked down conditions from the reference data, we discarded conditions for which only one independent perturbation ID remained, to avoid including off-target effects. In this process, we did not limit our analysis to HT-29 cells because the number of such genetic perturbations for HT-29 cells was small [0 records for overexpression, 865 records (296 genes) for ligand treatment, and 44,729 records (3666 genes) for knockdown].

By using these relationships between upstream and downstream genes, query DEGs were fully connected by arrows. If present, multiple edges and loops connecting a DEG to itself were removed. Kleinberg’s hub score [18] was used to identify the most influential gene in the network. This scoring method was originally developed for the complex world wide web to discover information sources and hubs that join the sources [18]. In short, the hub score was defined by the sum of authority scores, while the authority score was defined by the sum of hub scores. These recursive relationships were solved by finding the eigenvector of the autocorrelation matrix showing the link structure by using R package *igraph* (version 1.0.1) [44]. Components of the autocorrelation matrix were defined by the following formulation: α × δ. Here, α is a signal intensity ratio of query DEGs identified as an upstream gene in the network; δ is a penalty parameter that is the ratio of the number of upregulated or downregulated query DEGs to the number of upregulated or downregulated landmark genes in LINCS, respectively, to avoid off-target effects.

### Cell cultures and treatments

Human colorectal adenocarcinoma HT-29 cells [45] were cultured in RPMI-1640 (Wako, Osaka, Japan), supplemented with 10% heat-inactivated FBS and 100 μg/ml kanamycin. The chemical conditions for the in-house dataset were described previously [6, 7] and shown in Supplementary Table 1. 6-Mercaptopurine, doxorubicin, etoposide gemcitabine, methotrexate, mitomycin C, mitoxantrone, and topotecan have different MoA but ultimately induce DNA damage, so they were referred to here as DNA damaging agents [7].

### Immunoblot analysis

Immunoblot analysis was conducted as described previously [22]. Briefly, equal amounts of protein were resolved on an SDS-polyacrylamide gradient gel and transferred by electroblotting onto a nitrocellulose membrane. Membranes were probed with the indicated primary antibodies. The specific signals were visualized with a chemiluminescence detection system using appropriate secondary antibodies (Perkin-Elmer, Waltham, MA, USA). The following antibodies were used for immunoblotting: anti-β-actin (Sigma, St. Louis, MO, USA); anti-RPS3, anti-mTOR, and anti-MYC (Cell Signaling Technology, Danvers, MA, USA). β-actin, RPS3 and mTOR were used as controls.

### RNA preparation

Total RNA from cultured cells was extracted using an RNeasy RNA purification kit (Qiagen, Valencia, CA, USA). RNA quality was checked with a 2100 Bioanalyzer (Agilent Technologies, Santa Clara, CA, USA).

### siRNA treatment

ON-TARGETplus SMART pool siRNA (GE Healthcare, Little Chalfont, UK) and Silencer Select Pre-designed siRNA (Thermo Fisher Scientific, Waltham, MA, USA) were used for the knockdown of *MYC* expression. ON-TARGETplus Non-targeting Pool (GE Healthcare) or Silencer Select Pre-designed siRNA (Thermo Fisher Scientific) was used as a control. HT-29 cells were seeded at a density of 8×10^4^ cells/well on a six-well plate for immunoblot analysis and at a density of 3×10^3^ cells/well on a 96-well plate for cell viability assay, and were transfected for 24 h with 20 nM of each siRNA in Opti-MEM (Thermo Fisher Scientific) with lipofectamine RNAiMAX (Thermo Fisher Scientific), in accordance with the manufacturer’s reverse transfection protocol. After 48 h, the cells were used for further experiments.

### Cell growth assay

Cell growth was determined by a CellTiter-Glo luminescent cell viability assay (Promega), in accordance with the manufacturer’s protocol. Cell growth is shown as a percentage of the control level.

### Data availability

The microarray datasets of *MYC* siRNA experiments were deposited in the NCBI Gene Expression Omnibus under the series accession no. GSE104175.

### Computer code

The statistical computing language R (https://www.r-project.org/) was used for all InDePTH analyses, including estimating the best cut-off point of the similarity score.

## SUPPLEMENTARY MATERIALS FIGURES AND TABLES







## Abbreviations

6-MP: 6-mercaptopurine
AURKA: Aurora Kinase A
CCNB1: cyclin B1
CDKN1A: cyclin-dependent kinase inhibitor 1A
CMap: connectivity map
DEGs: differentially expressed genes
DeMAND: detecting mechanism of action by network dysregulation
DOX: doxorubicin
ETP: etoposide
GADD45A: DNA: damage-inducible gene 45A
GEM: gemcitabine
GSEA: Gene Set Enrichment Analysis
InDePTH: influential gene detection in perturbed transcriptome hierarchical network
JUN: jun proto-oncogene
KLF6: Kruppel-like factor 6
LINCS: The Library of Integrated Network-Based Cellular Signatures
MIT: mitoxantrone
MMC: mitomycin C
MoA: mechanisms of action
MTX: methotrexate
MYC: v-myc myelocytomatosis viral oncogene homolog
PCNA: proliferating cell nuclear antigen
PEM: pemetrexed
PLK1: polo-like kinase 1
ROC: receiver operating characteristic
RPS3: ribosomal protein S3
TOP: topotecan
