# Supplementary material for: InDePTH: detection of hub genes for developing gene expression networks under anticancer drug treatment
Source: Oncotarget. 2018 Jun 26;9(49):29097–111. doi: 10.18632/oncotarget.25624 (PMC6044382; doi:10.18632/oncotarget.25624)
Supplement: Supplementary file 1 [file oncotarget-09-29097-s001.pdf]

# InDePTH: detection of hub genes for developing gene expression networks under anticancer drug treatment

## SUPPLEMENTARY MATERIALS

## REFERENCES


1. Bansal M, Yang J, Karan C, Menden MP, Costello JC, Tang H, Xiao G, Li Y, Allen J, Zhong R, Chen B, Kim M, Wang T, et al. A community computational challenge to predict the activity of pairs of compounds. Nat Biotechnol. 2014; 32:1213–22. https://doi.org/10.1038/nbt.3052.
2. Woo JH, Shimoni Y, Yang WS, Subramaniam P, Iyer A, Nicoletti P, Rodríguez Martínez M, López G, Mattioli M, Realubit R, Karan C, Stockwell BR, Bansal M, et al. Elucidating Compound Mechanism of Action by Network Perturbation Analysis. Cell. 2015; 162:441–51. https://doi.org/10.1016/j.cell.2015.05.056.
3. Koido M, Haga N, Furuno A, Tsukahara S, Sakurai J, Tani Y, Sato S, Tomida A. Mitochondrial deficiency impairs hypoxic induction of HIF-1 transcriptional activity and retards tumor growth. Oncotarget. 2017; 8:11841–54. https://doi.org/10.18632/oncotarget.14415.

**1.3 M**

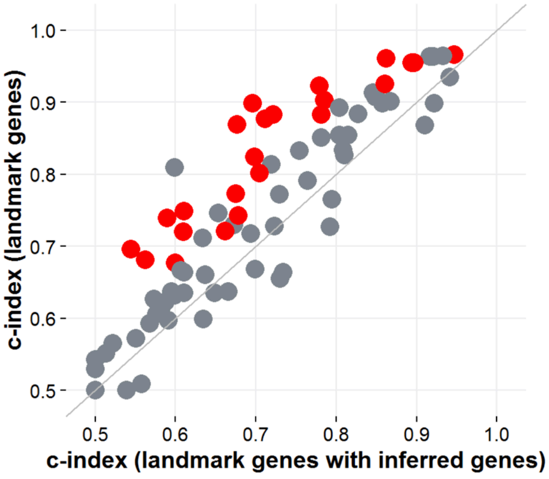


**Supplementary Figure 1: C-index comparison using LINCS 1.3 million perturbations.** C-index$_{ALL}$ comparison between from only landmark genes (y-axis) and from landmark genes with inferred genes (x-axis). Red plot indicates that the difference of c-index is statistically ($P$-value$<5.7\times10^{-4}$, Bonferroni corrected, $n$=88).

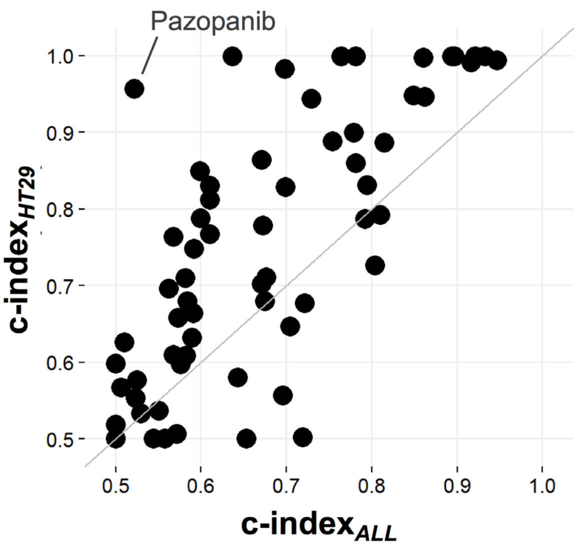


**Supplementary Figure 2: C-index comparison using landmark genes with inferred genes.** C-index from landmark genes with inferred genes comparison between one from 1.3 million LINCS dataset (x-axis, c-index$_{ALL}$) and the one from HT-29 LINCS dataset (y-axis, c-index$_{HT29}$).

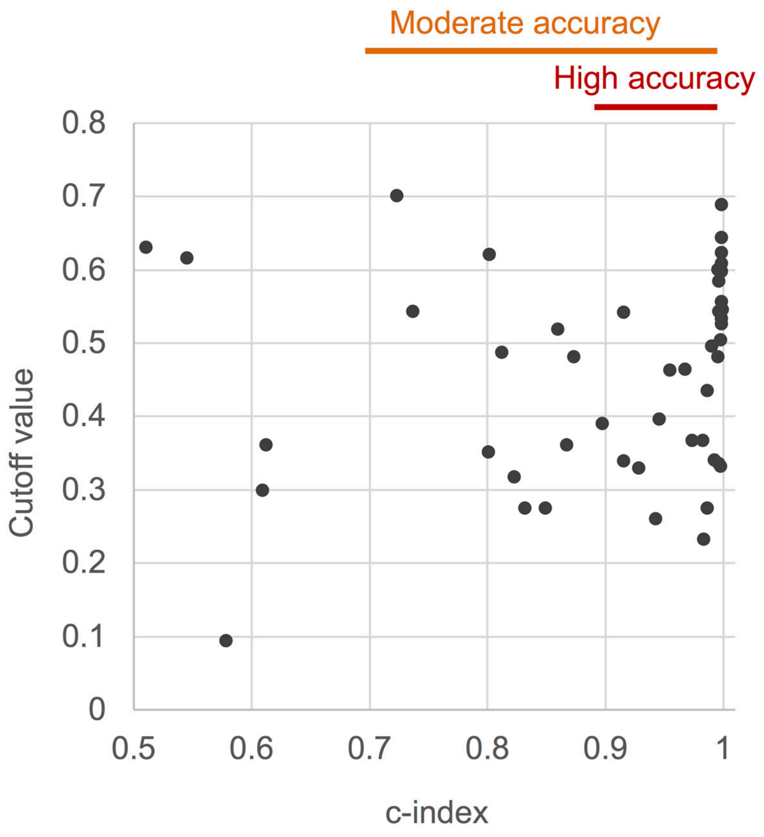


**Supplementary Figure 3: C-index and cutoff value of CMap similarity score.** Scatter plot of c-index$_{HT29}$ in x-axis and CMap similarity score cutoff value from InDePTH. See also Supplementary Table 1.

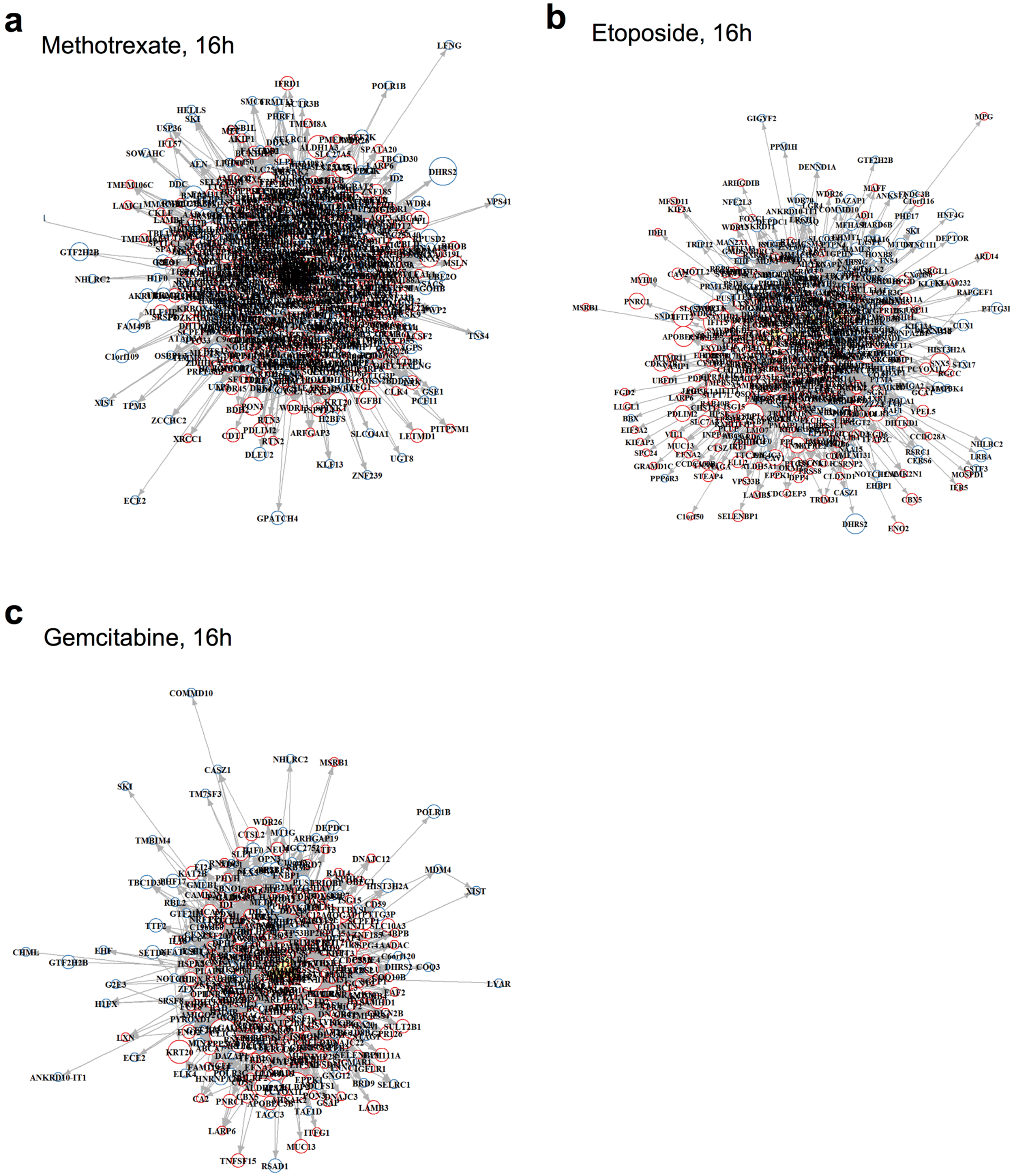


**Supplementary Figure 4: Complexity of drug-induced gene expression network.** See legend of Figure 3.

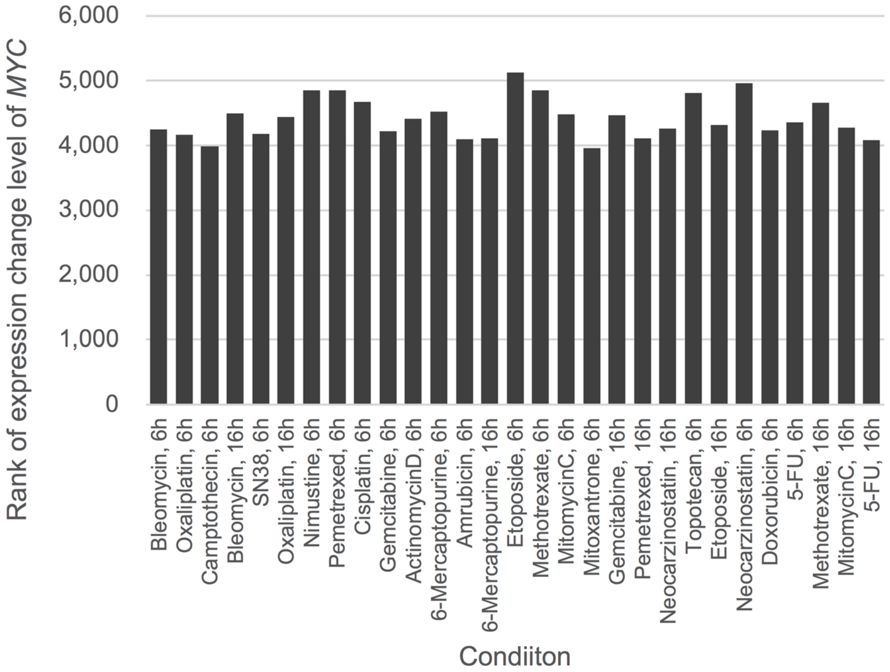


**Supplementary Figure 5: Moderate expression change of *MYC*.** Rank of *MYC* expression change levels by descendent order, under indicated DNA-damaging agents treatments.

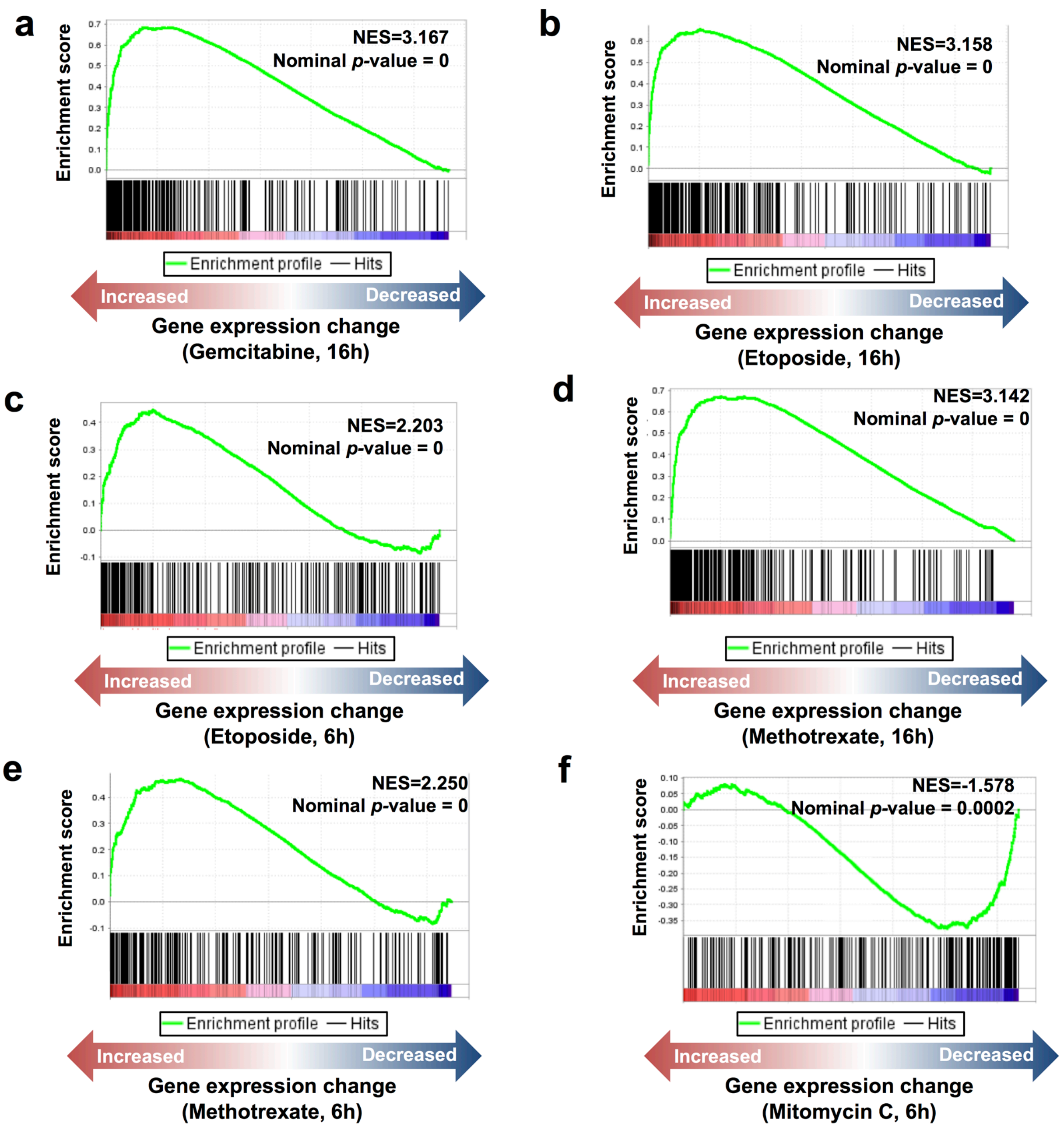


**Supplementary Figure 6: Enrichment plot of GSEA, *MYC* siRNA UP DEGs.** Enrichment plot using *MYC* siRNA-increased gene sets. See Figure 4.

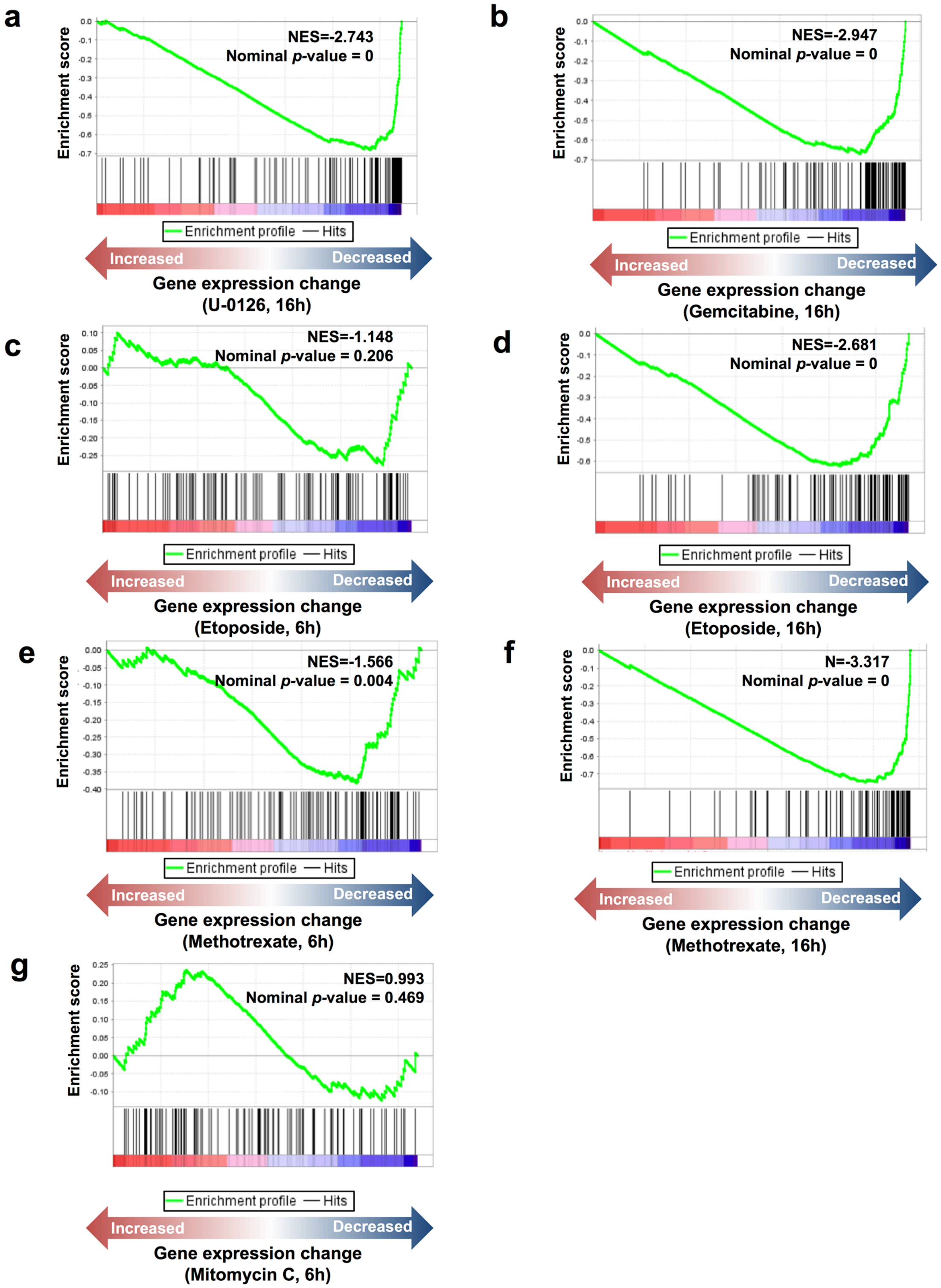


**Supplementary Figure 7: Enrichment plot of GSEA, MYC siRNA DOWN DEGs.** Enrichment plot using *MYC* siRNA-decreased gene sets. See Figure 4.

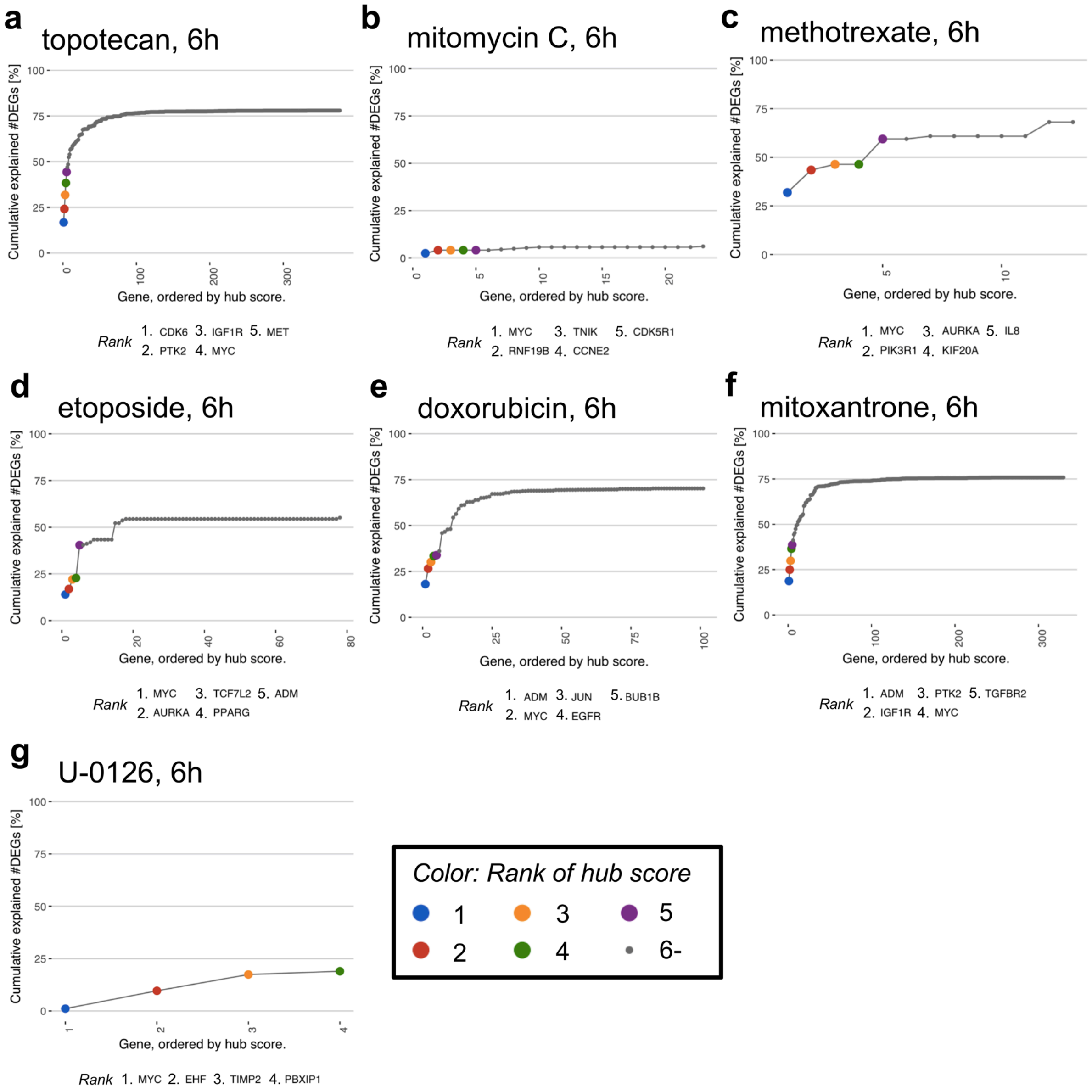


**Supplementary Figure 8: Collective effects of genes on drug-induced gene expression networks (6 hours drug treatments). (a-g)** Percentage of cumulative DEG number overlapped with downstream genes sequentially added from the top of the hub score by InDePTH analysis. Hub scores were determined from recursive relationships within query DEGs and signal intensity ratio of query DEGs (see Materials and Methods). In each figure, the top 5 hub genes from InDePTH analysis were specified by colors.

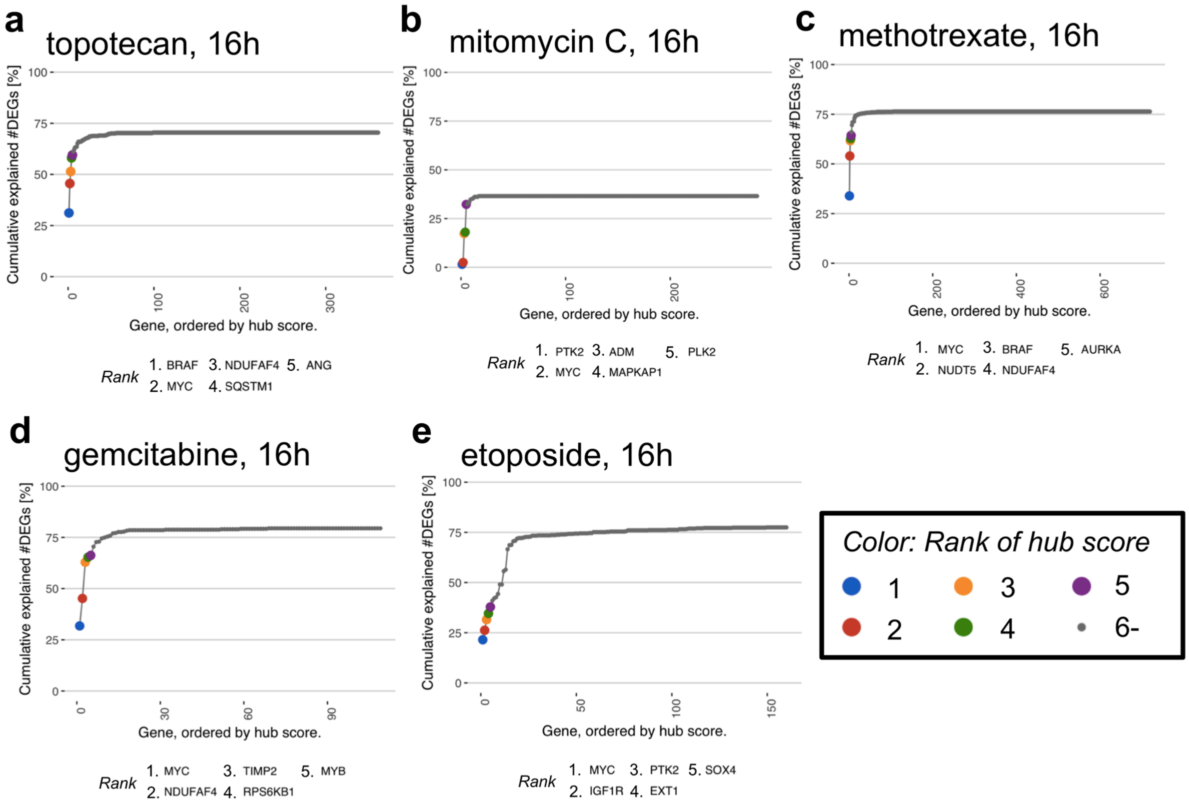


**Supplementary Figure 9: Collective effects of genes on drug-induced gene expression networks (16 hours drug treatments).** **(a-e)** Percentage of cumulative DEG number overlapped with downstream genes sequentially added from the top of the hub score by InDePTH analysis. Hub scores were determined from recursive relationships within query DEGs and signal intensity ratio of query DEGs (see Materials and Methods). In each figure, the top 5 hub genes from InDePTH analysis were specified by colors.

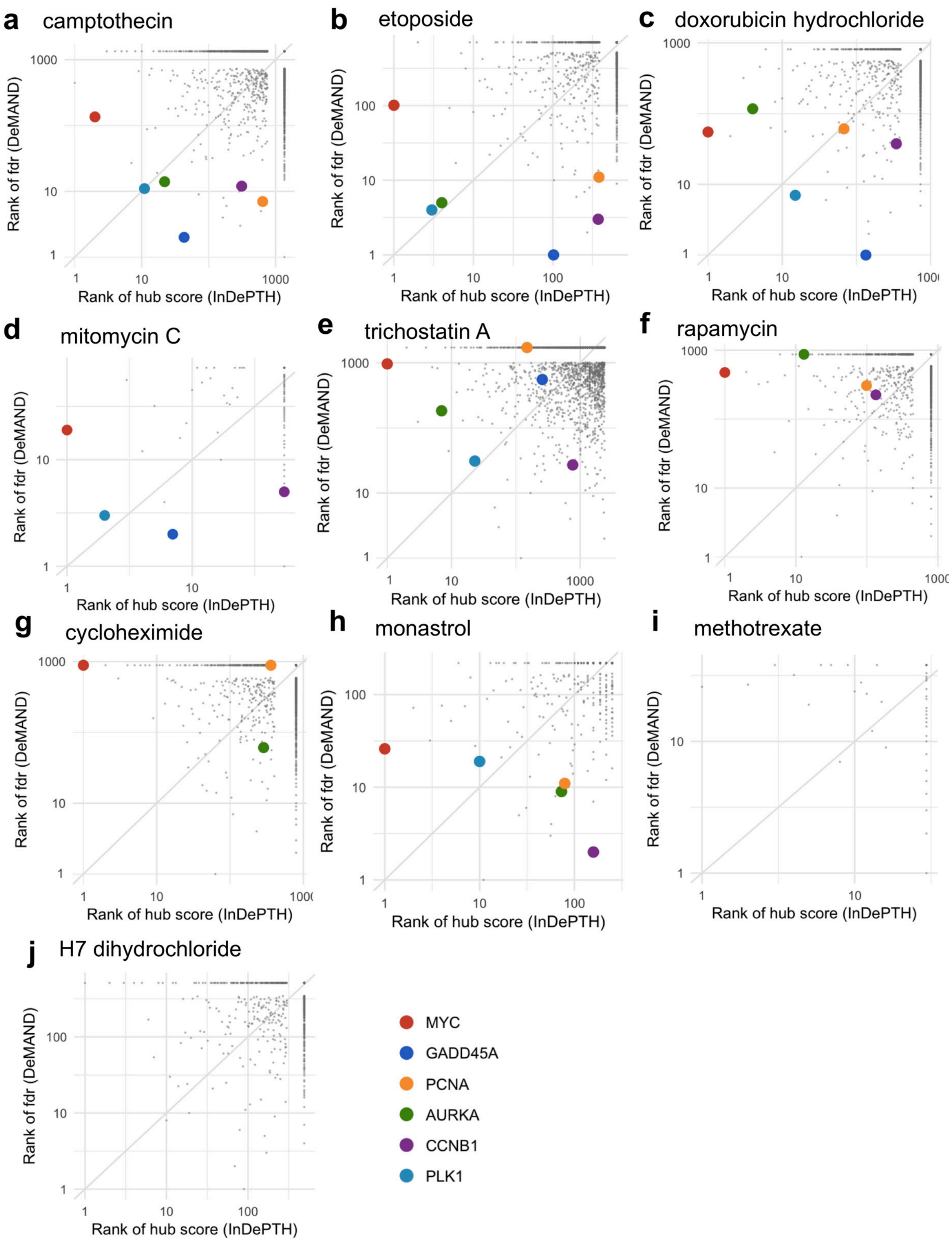


**Supplementary Figure 10: Comparison of gene ranking of InDePTH analysis with that of DeMAND analysis. (a-j)** Gene ranking from InDePTH analysis was compared with that from DeMAND analysis. The ranking was only in the indicated drug-induced DEGs (FDR <.10 in t-tests) in diffuse large B cell lymphoma cells (OCI-LY3) [1].

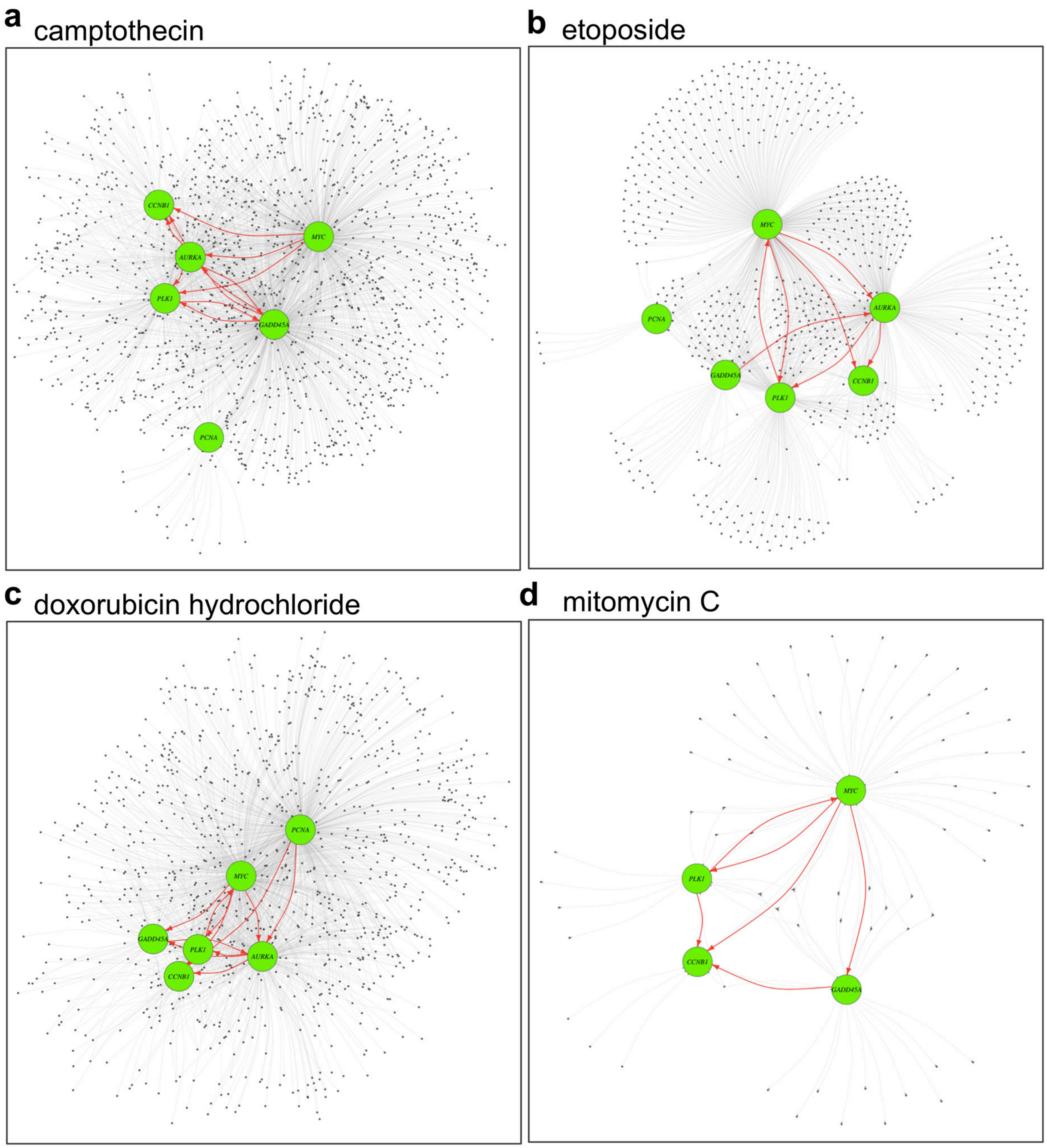


**Supplementary Figure 11: Hierarchical networks of DEGs by DNA damaging agents in OCI-LY3 cells. (a-d)** Gene regulatory networks reconstructed by InDePTH analysis. OCI-LY3 cells were treated with the indicated drugs. *MYC*, *PLK1*, and mRNAs of effector proteins for the drugs [2] were highlighted by green and arrows connecting to the genes were highlighted by red.

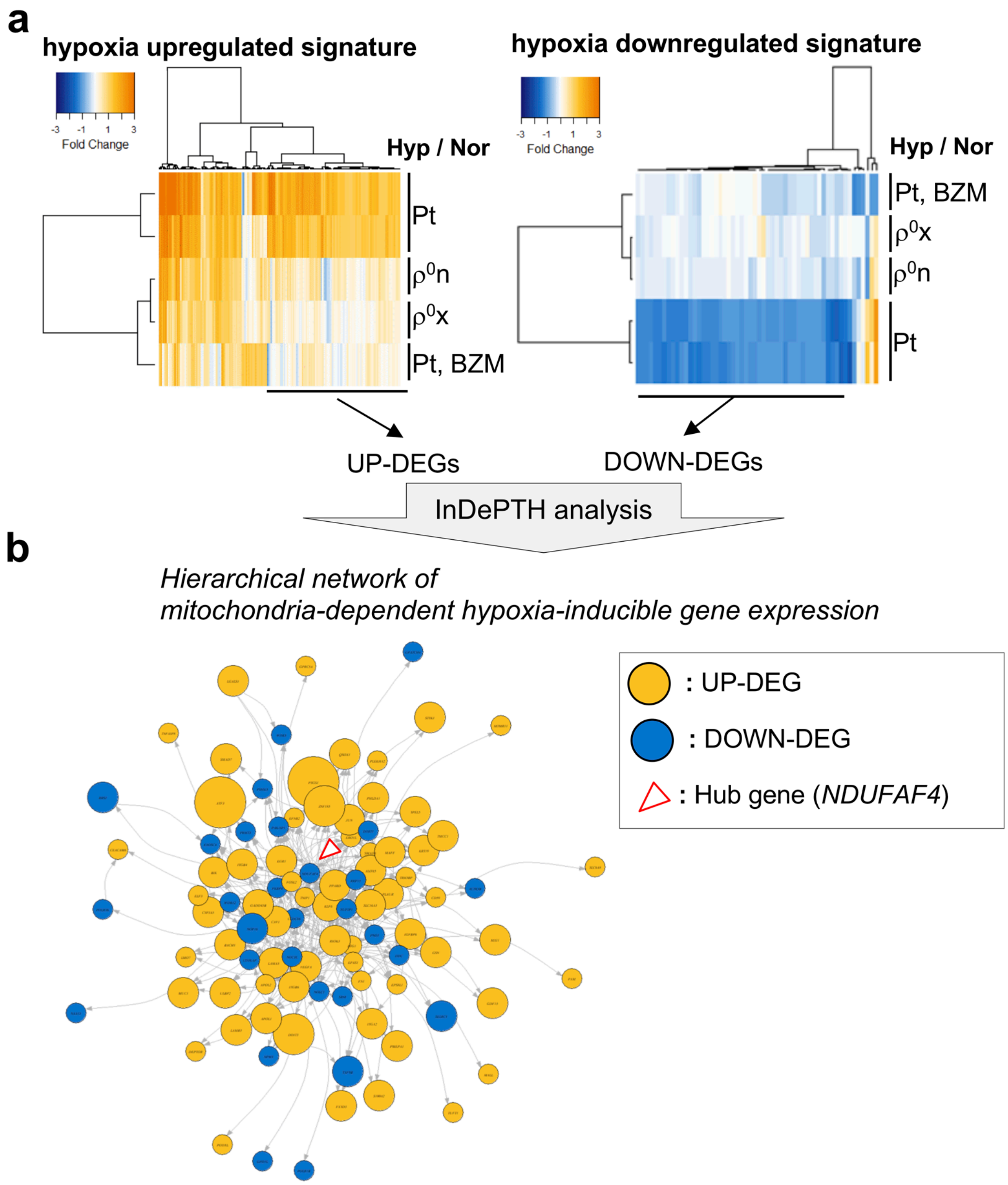


**Supplementary Figure 12: Mitochondria-dependent hypoxia-inducible gene expression network. (a)** Query DEGs. The heatmap was described from Figure 1b and 1c from [3] by different color keys and with query DEGs indication. **(b)** The hierarchical network of mitochondria-dependent hypoxia-inducible genes and the hub gene.

**Supplementary Table 1: Summary of conditions, c-index, and number of hit perturbations**

See Supplementary File 1

**Supplementary Table 2: Most influential genes in each compound-induced gene regulatory network**

| DB.INDEX | Drug | conc | time | c-index | Gene Symbol | Fold Change |
|---|---|---|---|---|---|---|
| GR_ChDB_0003 | Tunicamycin | 3ug/ml | 6h | 0.996 | GADD45A | 4.12 |
| GR_ChDB_0004 | Thapsigargin | 10nM | 6h | 0.86 | FUT1 | 2.88 |
| GR_ChDB_0012 | 17-AAG | 100nM | 6h | 0.996 | PIK3R1 | 0.36 |
| GR_ChDB_0018 | Gemcitabine | 1uM | 6h | 0.991 | AURKA | 0.36 |
| GR_ChDB_0021 | Mitomycin C | 10uM | 6h | 0.724 | MYC | 0.44 |
| GR_ChDB_0026 | Methotrexate | 1uM | 6h | 0.968 | MYC | 0.44 |
| GR_ChDB_0028 | Temsirolimus | 10uM | 6h | 0.946 | SQLE | 0.48 |
| GR_ChDB_0033 | SN38 | 3uM | 6h | 0.998 | ADM | 5.87 |
| GR_ChDB_0035 | Topotecan | 3uM | 6h | 0.984 | CDK6 | 0.25 |
| GR_ChDB_0036 | Doxorubicin | 3uM | 6h | 0.993 | ADM | 5.97 |
| GR_ChDB_0037 | Etoposide | 30uM | 6h | 0.874 | MYC | 0.46 |
| GR_ChDB_0038 | Mitoxantrone | 3uM | 6h | 0.987 | ADM | 8.11 |
| GR_ChDB_0047 | Sorafenib | 10uM | 6h | 0.955 | PPIF | 0.43 |
| GR_ChDB_0048 | Sorafenib | 10uM | 6h | 0.916 | VEGFA | 3.14 |
| GR_ChDB_0054 | Erlotinib | 30uM | 6h | 0.802 | ASNS | 4.05 |
| GR_ChDB_0056_2 | Gefitinib | 30uM | 6h | 0.929 | MYO10 | 0.35 |
| GR_ChDB_0057 | Pazopanib | 30uM | 6h | 0.983 | JUN | 0.32 |
| GR_ChDB_0063 | Vincristine | 30nM | 16h | 0.987 | CDK1 | 0.06 |
| GR_ChDB_0064 | Paclitaxel | 30nM | 16h | 0.801 | CDK1 | 0.10 |
| GR_ChDB_0065 | Docetaxel | 30nM | 16h | 0.868 | CDK1 | 0.13 |
| GR_ChDB_0067 | Mitomycin C | 10uM | 16h | 0.813 | PTK2 | 0.36 |
| GR_ChDB_0075 | Gemcitabine | 1uM | 16h | 0.85 | MYC | 0.36 |
| GR_ChDB_0079 | Methotrexate | 1uM | 16h | 0.832 | MYC | 0.23 |
| GR_ChDB_0080 | 6-Mercaptopurine | 100uM | 16h | 0.916 | BRAF | 2.27 |
| GR_ChDB_0081 | Temsirolimus | 10uM | 16h | 0.737 | DHRS3 | 2.07 |
| GR_ChDB_0084 | Etoposide | 30uM | 16h | 0.898 | MYC | 0.34 |
| GR_ChDB_0088 | MLN-4924 | 10uM | 6h | 0.997 | HBEGF | 2.15 |
| GR_ChDB_0092 | SB218078 | 3uM | 6h | 0.998 | JUN | 0.40 |
| GR_ChDB_0094 | GSK-3 inhibitor IX | 10uM | 6h | 0.997 | ERBB3 | 0.41 |
| GR_ChDB_0103 | U-0126 | 30uM | 6h | 0.999 | MYC | 0.11 |
| GR_ChDB_0104 | SU11274 | 30uM | 6h | 0.823 | SGK1 | 0.33 |
| GR_ChDB_0120 | Crizotinib | 10uM | 6h | 0.943 | BRAF | 2.70 |
| GR_ChDB_0154 | BEZ235 | 1uM | 6h | 0.999 | KLF6 | 5.34 |
| GR_ChDB_0157 | Afatinib | 10uM | 6h | 0.974 | VEGFA | 2.73 |

Most influential genes and their fold changes in query DEGs are shown. Conditions with c-index$_{HT29}$ >0.7 are shown. conc., concentration of drug.

**Supplementary Table 3: Upstream genes information with hub score of all tested compounds**

See Supplementary File 2

**Supplementary Table 4: Hub score of each compound focusing on *MYC***

| DB.INDEX | Drug | Conc. | Time | HubScore | Fold Change |
|---|---|---|---|---|---|
| HT29_10_GR_ChDB_0075 | Gemcitabine | 1uM | 16h | 1.00 | 0.36 |
| HT29_10_GR_ChDB_0079 | Methotrexate | 1uM | 16h | 1.00 | 0.23 |
| HT29_10_GR_ChDB_0084 | Etoposide | 30uM | 16h | 1.00 | 0.34 |
| HT29_12_GR_ChDB_0103 | U-0126 | 30uM | 6h | 1.00 | 0.11 |
| HT29_3_GR_ChDB_0021 | Mitomycin C | 10uM | 6h | 1.00 | 0.44 |
| HT29_3_GR_ChDB_0026 | Methotrexate | 1uM | 6h | 1.00 | 0.44 |
| HT29_4_GR_ChDB_0037 | Etoposide | 30uM | 6h | 1.00 | 0.46 |
| HT29_10_GR_ChDB_0080 | 6-Mercaptopurine | 100uM | 16h | 0.86 | 0.47 |
| HT29_4_GR_ChDB_0038 | Mitoxantrone | 3uM | 6h | 0.53 | 0.39 |
| HT29_4_GR_ChDB_0035 | Topotecan | 3uM | 6h | 0.46 | 0.34 |
| HT29_4_GR_ChDB_0036 | Doxorubicin | 3uM | 6h | 0.40 | 0.30 |
| HT29_7_GR_ChDB_0067 | Mitomycin C | 10uM | 16h | 0.09 | 0.21 |

Conc., concentration of the drug treatment; Fold Change, gene expression change level of *MYC* under the drug treatment.

**Supplementary Table 5: Result of GSEA analysis, enriched in *MYC* siRNA-decreased genes**

| Rank | Gene Set | NES | NOM p-val | FDR q-val |
|---|---|---|---|---|
| 1 | HALLMARK_MYC_TARGETS_V1 | 4.1 | 0 | 0 |
| 2 | HALLMARK_MYC_TARGETS_V2 | 3.9 | 0 | 0 |
| 3 | HALLMARK_MTORC1_SIGNALING | 2.6 | 0 | 0 |
| 4 | HALLMARK_E2F_TARGETS | 2.6 | 0 | 0 |
| 5 | HALLMARK_UNFOLDED_PROTEIN_RESPONSE | 2.4 | 0 | 0 |
| 6 | HALLMARK_G2M_CHECKPOINT | 2.3 | 0 | 0 |
| 7 | HALLMARK_DNA_REPAIR | 1.8 | 0.005 | 0.025 |
| 8 | HALLMARK_OXIDATIVE_PHOSPHORYLATION | 1.7 | 0.003 | 0.041 |

GSEA using hallmark signature of gene sets (FDR<.05).
