# Supplementary material for: InDePTH: detection of hub genes for developing gene expression networks under anticancer drug treatment
Source: Oncotarget. 2018 Jun 26;9(49):29097–111. doi: 10.18632/oncotarget.25624 (PMC6044382; doi:10.18632/oncotarget.25624)
Supplement: Supplementary file 2 [file oncotarget-09-29097-s002.docx]

## Supplementary Table S1. Summary of conditions, c-index, and number of hit perturbations.

Used compound names, calculated c-index, and hit perturbations from the InDePTH analysis are shown. conc., concentration of drug; smpl, number of samples in LINCS; sh, shRNA treatment; oe, overexpression; lig, ligand treatment.

|  |  |  |  | **ALL** | |  | **HT-29** | | |  | **Hit Perturbation** | | |
| --- | --- | --- | --- | --- | --- | --- | --- | --- | --- | --- | --- | --- | --- |
| **DB.INDEX** | **drug** | **Conc.** | **time** | **smpl** | **c-index** |  | **smpl** | **c-index** | **cutoff** |  | **sh** | **oe** | **lig** |
| GR_ChDB_0003 | Tunicamycin | 3ug/ml | 6h | 111 | 0.963 |  | 5 | 0.996 | 0.48 |  | 126 | 8 | 0 |
| GR_ChDB_0004 | Thapsigargin | 10nM | 6h | 254 | 0.593 |  | 16 | 0.86 | 0.518 |  | 738 | 122 | 25 |
| GR_ChDB_0005 | A23187 | 3uM | 6h | 49 | 0.913 |  | 0 | - | - |  | - | - | - |
| GR_ChDB_0006 | Cisplatin | 30uM | 6h | 108 | 0.500 |  | 0 | - | - |  | - | - | - |
| GR_ChDB_0007 | Trichostatin A | 300nM | 6h | 3697 | 0.966 |  | 237 | 0.997 | 0.335 |  | 14 | 0 | 8 |
| GR_ChDB_0009 | Bortezomib | 100nM | 6h | 292 | 0.964 |  | 10 | 0.999 | 0.525 |  | 44 | 0 | 0 |
| GR_ChDB_0010 | MG-132 | 1uM | 6h | 228 | 0.791 |  | 5 | 0.999 | 0.643 |  | 17 | 0 | 0 |
| GR_ChDB_0012 | 17-AAG | 100nM | 6h | 663 | 0.824 |  | 10 | 0.996 | 0.6 |  | 185 | 15 | 6 |
| GR_ChDB_0017 | 5-FU | 100uM | 6h | 142 | 0.551 |  | 0 | - | - |  | - | - | - |
| GR_ChDB_0018 | Gemcitabine | 1uM | 6h | 388 | 0.855 |  | 10 | 0.991 | 0.495 |  | 2721 | 361 | 44 |
| GR_ChDB_0020 | Melphala | 100uM | 6h | 27 | 0.809 |  | 0 | - | - |  | - | - | - |
| GR_ChDB_0021 | Mitomycin C | 10uM | 6h | 119 | 0.727 |  | 12 | 0.724 | 0.7 |  | 195 | 64 | 8 |
| GR_ChDB_0024 | Actinomycin D | 30nM | 6h | 18 | 0.500 |  | 0 | - | - |  | - | - | - |
| GR_ChDB_0026 | Methotrexate | 1uM | 6h | 119 | 0.851 |  | 4 | 0.968 | 0.464 |  | 2066 | 385 | 52 |
| GR_ChDB_0028 | Temsirolimus | 10uM | 6h | 173 | 0.854 |  | 10 | 0.946 | 0.396 |  | 9519 | 1974 | 244 |
| GR_ChDB_0030 | PP242 | 10uM | 6h | 15 | 0.868 |  | 0 | - | - |  | - | - | - |
| GR_ChDB_0033 | SN38 | 3uM | 6h | 278 | 0.907 |  | 10 | 0.998 | 0.331 |  | 332 | 43 | 11 |
| GR_ChDB_0034 | Camptothecin | 3uM | 6h | 199 | 0.922 |  | 5 | 1 | 0.545 |  | 0 | 0 | 0 |
| GR_ChDB_0035 | Topotecan | 3uM | 6h | 82 | 0.883 |  | 8 | 0.984 | 0.232 |  | 5467 | 1143 | 142 |
| GR_ChDB_0036 | Doxorubicin | 3uM | 6h | 444 | 0.869 |  | 38 | 0.993 | 0.34 |  | 894 | 114 | 17 |
| GR_ChDB_0037 | Etoposide | 30uM | 6h | 157 | 0.826 |  | 10 | 0.874 | 0.481 |  | 4148 | 769 | 67 |
| GR_ChDB_0038 | Mitoxantrone | 3uM | 6h | 343 | 0.899 |  | 9 | 0.987 | 0.274 |  | 4321 | 801 | 93 |
| GR_ChDB_0039 | Pemetrexed | 1uM | 6h | 165 | 0.500 |  | 10 | 0.613 | 0.36 |  | 10306 | 1888 | 282 |
| GR_ChDB_0047 | Sorafenib | 10uM | 6h | 472 | 0.676 |  | 12 | 0.955 | 0.462 |  | 401 | 16 | 0 |
| GR_ChDB_0048 | Sorafenib | 10uM | 6h | 472 | 0.664 |  | 12 | 0.916 | 0.541 |  | 255 | 17 | 5 |
| GR_ChDB_0049 | Sunitinib | 10uM | 6h | 264 | 0.597 |  | 10 | 0.546 | 0.615 |  | 86 | 8 | 4 |
| GR_ChDB_0050_3 | Sunitinib | 10uM | 6h | 264 | 0.627 |  | 10 | 0.511 | 0.63 |  | 144 | 12 | 4 |
| GR_ChDB_0054 | Erlotinib | 30uM | 6h | 214 | 0.615 |  | 5 | 0.802 | 0.62 |  | 962 | 147 | 18 |
| GR_ChDB_0056_2 | Gefitinib | 30uM | 6h | 387 | 0.632 |  | 10 | 0.929 | 0.329 |  | 7410 | 1493 | 229 |
| GR_ChDB_0057 | Pazopanib | 30uM | 6h | 201 | 0.565 |  | 5 | 0.983 | 0.366 |  | 1912 | 305 | 57 |
| GR_ChDB_0060 | Bortezomib | 100nM | 16h | 292 | 0.955 |  | 10 | 0.999 | 0.608 |  | 64 | 0 | 0 |
| GR_ChDB_0063 | Vincristine | 30nM | 16h | 207 | 0.749 |  | 12 | 0.987 | 0.434 |  | 3790 | 638 | 116 |
| GR_ChDB_0064 | Paclitaxel | 30nM | 16h | 322 | 0.681 |  | 13 | 0.801 | 0.351 |  | 7349 | 1461 | 221 |
| GR_ChDB_0065 | Docetaxel | 30nM | 16h | 112 | 0.739 |  | 10 | 0.868 | 0.36 |  | 5043 | 922 | 145 |
| GR_ChDB_0066 | 5-FU | 100uM | 16h | 142 | 0.53 |  | 0 | - | - |  | - | - | - |
| GR_ChDB_0067 | Mitomycin C | 10uM | 16h | 119 | 0.765 |  | 12 | 0.813 | 0.487 |  | 410 | 59 | 5 |
| GR_ChDB_0070 | Bortezomib | 100nM | 6h | 292 | 0.964 |  | 10 | 0.999 | 0.622 |  | 27 | 0 | 0 |
| GR_ChDB_0073 | Bortezomib | 100nM | 16h | 292 | 0.954 |  | 10 | 0.999 | 0.597 |  | 67 | 0 | 0 |
| GR_ChDB_0075 | Gemcitabine | 1uM | 16h | 388 | 0.773 |  | 10 | 0.85 | 0.274 |  | 12601 | 2269 | 261 |
| GR_ChDB_0079 | Methotrexate | 1uM | 16h | 119 | 0.813 |  | 4 | 0.832 | 0.274 |  | 11729 | 2045 | 194 |
| GR_ChDB_0080 | 6-Mercaptopurine | 100uM | 16h | 70 | 0.746 |  | 4 | 0.916 | 0.339 |  | 7002 | 1372 | 152 |
| GR_ChDB_0081 | Temsirolimus | 10uM | 16h | 173 | 0.729 |  | 10 | 0.737 | 0.542 |  | 4344 | 798 | 129 |
| GR_ChDB_0082 | Everolimus | 10uM | 16h | 58 | 0.543 |  | 4 | 0.500 | - |  | - | - | - |
| GR_ChDB_0084 | Etoposide | 30uM | 16h | 157 | 0.801 |  | 10 | 0.898 | 0.389 |  | 4182 | 824 | 74 |
| GR_ChDB_0085 | Pemetrexed | 1uM | 16h | 165 | 0.500 |  | 10 | 0.500 | - |  | - | - | - |
| GR_ChDB_0088 | MLN-4924 | 10uM | 6h | 209 | 0.961 |  | 10 | 0.997 | 0.542 |  | 241 | 12 | 0 |
| GR_ChDB_0092 | SB218078 | 3uM | 6h | 84 | 0.833 |  | 9 | 0.998 | 0.503 |  | 456 | 85 | 10 |
| GR_ChDB_0094 | GSK-3 inhibitor IX | 10uM | 6h | 270 | 0.883 |  | 5 | 0.997 | 0.584 |  | 251 | 30 | 7 |
| GR_ChDB_0096 | Decitabine | 100uM | 6h | 109 | 0.508 |  | 6 | 0.61 | 0.298 |  | 8846 | 2099 | 337 |
| GR_ChDB_0097 | FH535 | 30uM | 6h | 23 | 0.500 |  | 0 | - | - |  | - | - | - |
| GR_ChDB_0103 | U-0126 | 30uM | 6h | 167 | 0.772 |  | 9 | 0.999 | 0.533 |  | 98 | 1 | 1 |
| GR_ChDB_0104 | SU11274 | 30uM | 6h | 203 | 0.606 |  | 5 | 0.823 | 0.316 |  | 11379 | 2655 | 305 |
| GR_ChDB_0119 | Vemurafenib | 30uM | 6h | 557 | 0.66 |  | 15 | 0.999 | 0.688 |  | 1 | 0 | 0 |
| GR_ChDB_0120 | Crizotinib | 10uM | 6h | 367 | 0.635 |  | 22 | 0.943 | 0.26 |  | 5049 | 624 | 86 |
| GR_ChDB_0121 | Ruxolitinib | 100uM | 6h | 269 | 0.500 |  | 10 | 0.500 | - |  | - | - | - |
| GR_ChDB_0122 | Axitinib | 100uM | 6h | 62 | 0.695 |  | 6 | 0.500 | - |  | - | - | - |
| GR_ChDB_0151 | ABT-263 | 30uM | 6h | 169 | 0.500 |  | 10 | 0.500 | - |  | - | - | - |
| GR_ChDB_0152 | Celecoxib | 100uM | 6h | 156 | 0.500 |  | 11 | 0.579 | 0.093 |  | 14640 | 2350 | 346 |
| GR_ChDB_0154 | BEZ235 | 1uM | 6h | 302 | 0.925 |  | 10 | 0.999 | 0.556 |  | 15 | 5 | 0 |
| GR_ChDB_0155 | Dabrafenib | 10uM | 6h | 143 | 0.655 |  | 0 | - | - |  | - | - | - |
| GR_ChDB_0156 | Trametinib | 1nM | 6h | 133 | 0.664 |  | 0 | - | - |  | - | - | - |
| GR_ChDB_0157 | Afatinib | 10uM | 6h | 190 | 0.668 |  | 6 | 0.974 | 0.366 |  | 4703 | 694 | 94 |
