# Supplementary material for: InDePTH: detection of hub genes for developing gene expression networks under anticancer drug treatment
Source: Oncotarget. 2018 Jun 26;9(49):29097–111. doi: 10.18632/oncotarget.25624 (PMC6044382; doi:10.18632/oncotarget.25624)
Supplement: Supplementary file 3 [file oncotarget-09-29097-s003.docx]

## Supplementary Table S3. Upstream genes information with hub score of all tested compounds.

Upstream genes obtained from InDePTH (c-index*_HT29_* > 0.7) and their hub scores and drug-induced gene expression change levels.

| **DB.INDEX** | **Drug** | **Conc.** | **Time** | **Gene Symbol** | **Hub Score** | **Fold Change** |
| --- | --- | --- | --- | --- | --- | --- |
| HT29_1_GR_ChDB_0003 | Tunicamycin | 3ug/ml | 6h | GADD45A | 1.00 | 4.12 |
| HT29_1_GR_ChDB_0004 | Thapsigargin | 10nM | 6h | FUT1 | 1.00 | 2.88 |
| HT29_1_GR_ChDB_0004 | Thapsigargin | 10nM | 6h | PIK3R1 | 0.27 | 0.33 |
| HT29_1_GR_ChDB_0004 | Thapsigargin | 10nM | 6h | HERPUD1 | 0.20 | 7.23 |
| HT29_1_GR_ChDB_0004 | Thapsigargin | 10nM | 6h | STK25 | 0.09 | 0.47 |
| HT29_1_GR_ChDB_0004 | Thapsigargin | 10nM | 6h | SLC7A11 | 0.06 | 7.81 |
| HT29_1_GR_ChDB_0004 | Thapsigargin | 10nM | 6h | SLC3A2 | 0.05 | 2.01 |
| HT29_1_GR_ChDB_0004 | Thapsigargin | 10nM | 6h | CBLB | 0.04 | 2.79 |
| HT29_1_GR_ChDB_0004 | Thapsigargin | 10nM | 6h | E2F1 | 0.03 | 0.49 |
| HT29_1_GR_ChDB_0004 | Thapsigargin | 10nM | 6h | ETV5 | 0.03 | 2.87 |
| HT29_1_GR_ChDB_0004 | Thapsigargin | 10nM | 6h | MYB | 0.01 | 0.42 |
| HT29_1_GR_ChDB_0004 | Thapsigargin | 10nM | 6h | PSAT1 | 0.01 | 2.63 |
| HT29_1_GR_ChDB_0004 | Thapsigargin | 10nM | 6h | SDF2L1 | 0.01 | 3.15 |
| HT29_1_GR_ChDB_0004 | Thapsigargin | 10nM | 6h | BTG1 | 0.01 | 2.11 |
| HT29_1_GR_ChDB_0004 | Thapsigargin | 10nM | 6h | CLIC4 | 0.01 | 2.31 |
| HT29_1_GR_ChDB_0004 | Thapsigargin | 10nM | 6h | VEGFA | 0.01 | 4.07 |
| HT29_1_GR_ChDB_0004 | Thapsigargin | 10nM | 6h | FAM120A | 0.00 | 0.49 |
| HT29_1_GR_ChDB_0004 | Thapsigargin | 10nM | 6h | JUN | 0.00 | 0.39 |
| HT29_1_GR_ChDB_0004 | Thapsigargin | 10nM | 6h | HBEGF | 0.00 | 2.03 |
| HT29_1_GR_ChDB_0004 | Thapsigargin | 10nM | 6h | BCR | 0.00 | 0.48 |
| HT29_1_GR_ChDB_0004 | Thapsigargin | 10nM | 6h | SEL1L | 0.00 | 4.11 |
| HT29_1_GR_ChDB_0004 | Thapsigargin | 10nM | 6h | GRB10 | 0.00 | 2.08 |
| HT29_1_GR_ChDB_0004 | Thapsigargin | 10nM | 6h | RHOQ | 0.00 | 2.91 |
| HT29_1_GR_ChDB_0004 | Thapsigargin | 10nM | 6h | DNAJB9 | 0.00 | 5.27 |
| HT29_1_GR_ChDB_0004 | Thapsigargin | 10nM | 6h | HBP1 | 0.00 | 2.68 |
| HT29_1_GR_ChDB_0004 | Thapsigargin | 10nM | 6h | ICA1 | 0.00 | 2.27 |
| HT29_1_GR_ChDB_0004 | Thapsigargin | 10nM | 6h | RNF167 | 0.00 | 0.43 |
| HT29_1_GR_ChDB_0004 | Thapsigargin | 10nM | 6h | CSGALNACT2 | 0.00 | 2.34 |
| HT29_1_GR_ChDB_0004 | Thapsigargin | 10nM | 6h | ERP44 | 0.00 | 2.07 |
| HT29_1_GR_ChDB_0004 | Thapsigargin | 10nM | 6h | WARS | 0.00 | 3.14 |
| HT29_1_GR_ChDB_0004 | Thapsigargin | 10nM | 6h | YIPF4 | 0.00 | 2.20 |
| HT29_1_GR_ChDB_0004 | Thapsigargin | 10nM | 6h | HYOU1 | 0.00 | 4.03 |
| HT29_1_GR_ChDB_0004 | Thapsigargin | 10nM | 6h | SNX11 | 0.00 | 2.20 |
| HT29_1_GR_ChDB_0004 | Thapsigargin | 10nM | 6h | GCFC2 | 0.00 | 2.03 |
| HT29_1_GR_ChDB_0004 | Thapsigargin | 10nM | 6h | TIA1 | 0.00 | 0.48 |
| HT29_1_GR_ChDB_0004 | Thapsigargin | 10nM | 6h | ARHGEF2 | 0.00 | 3.02 |
| HT29_1_GR_ChDB_0004 | Thapsigargin | 10nM | 6h | TRIM2 | 0.00 | 2.13 |
| HT29_1_GR_ChDB_0004 | Thapsigargin | 10nM | 6h | KDELR3 | 0.00 | 2.06 |
| HT29_1_GR_ChDB_0004 | Thapsigargin | 10nM | 6h | UBE2J1 | 0.00 | 2.41 |
| HT29_1_GR_ChDB_0004 | Thapsigargin | 10nM | 6h | EDEM1 | 0.00 | 4.23 |
| HT29_1_GR_ChDB_0004 | Thapsigargin | 10nM | 6h | SLC1A4 | 0.00 | 3.91 |
| HT29_1_GR_ChDB_0004 | Thapsigargin | 10nM | 6h | MCFD2 | 0.00 | 2.10 |
| HT29_1_GR_ChDB_0004 | Thapsigargin | 10nM | 6h | SEC24D | 0.00 | 8.29 |
| HT29_1_GR_ChDB_0004 | Thapsigargin | 10nM | 6h | TMEM39A | 0.00 | 2.84 |
| HT29_1_GR_ChDB_0004 | Thapsigargin | 10nM | 6h | CTBS | 0.00 | 2.10 |
| HT29_1_GR_ChDB_0004 | Thapsigargin | 10nM | 6h | PCK2 | 0.00 | 4.51 |
| HT29_1_GR_ChDB_0004 | Thapsigargin | 10nM | 6h | IBTK | 0.00 | 3.15 |
| HT29_1_GR_ChDB_0004 | Thapsigargin | 10nM | 6h | OGT | 0.00 | 2.07 |
| HT29_1_GR_ChDB_0004 | Thapsigargin | 10nM | 6h | SKP2 | 0.00 | 0.48 |
| HT29_1_GR_ChDB_0004 | Thapsigargin | 10nM | 6h | CPNE3 | 0.00 | 0.43 |
| HT29_1_GR_ChDB_0004 | Thapsigargin | 10nM | 6h | NFE2L1 | 0.00 | 2.22 |
| HT29_1_GR_ChDB_0004 | Thapsigargin | 10nM | 6h | NUCB2 | 0.00 | 6.25 |
| HT29_1_GR_ChDB_0004 | Thapsigargin | 10nM | 6h | CLCN3 | 0.00 | 2.10 |
| HT29_1_GR_ChDB_0004 | Thapsigargin | 10nM | 6h | MPZL1 | 0.00 | 0.47 |
| HT29_1_GR_ChDB_0004 | Thapsigargin | 10nM | 6h | RIOK3 | 0.00 | 2.15 |
| HT29_1_GR_ChDB_0004 | Thapsigargin | 10nM | 6h | TRIB3 | 0.00 | 4.65 |
| HT29_1_GR_ChDB_0004 | Thapsigargin | 10nM | 6h | CRELD2 | 0.00 | 3.41 |
| HT29_1_GR_ChDB_0004 | Thapsigargin | 10nM | 6h | CCNG2 | 0.00 | 2.80 |
| HT29_1_GR_ChDB_0004 | Thapsigargin | 10nM | 6h | DDIT4 | 0.00 | 25.07 |
| HT29_1_GR_ChDB_0004 | Thapsigargin | 10nM | 6h | ATXN1 | 0.00 | 2.03 |
| HT29_1_GR_ChDB_0004 | Thapsigargin | 10nM | 6h | CBX4 | 0.00 | 2.23 |
| HT29_1_GR_ChDB_0004 | Thapsigargin | 10nM | 6h | FZD2 | 0.00 | 2.51 |
| HT29_1_GR_ChDB_0004 | Thapsigargin | 10nM | 6h | UFL1 | 0.00 | 2.36 |
| HT29_1_GR_ChDB_0004 | Thapsigargin | 10nM | 6h | TMF1 | 0.00 | 2.14 |
| HT29_1_GR_ChDB_0004 | Thapsigargin | 10nM | 6h | CDC73 | 0.00 | 2.54 |
| HT29_1_GR_ChDB_0004 | Thapsigargin | 10nM | 6h | IMPACT | 0.00 | 2.06 |
| HT29_1_GR_ChDB_0004 | Thapsigargin | 10nM | 6h | RNF170 | 0.00 | 2.04 |
| HT29_1_GR_ChDB_0004 | Thapsigargin | 10nM | 6h | ZNF165 | 0.00 | 2.99 |
| HT29_1_GR_ChDB_0004 | Thapsigargin | 10nM | 6h | RPS6KA5 | 0.00 | 2.07 |
| HT29_1_GR_ChDB_0004 | Thapsigargin | 10nM | 6h | PLAUR | 0.00 | 2.24 |
| HT29_1_GR_ChDB_0004 | Thapsigargin | 10nM | 6h | MED1 | 0.00 | 0.47 |
| HT29_1_GR_ChDB_0004 | Thapsigargin | 10nM | 6h | SON | 0.00 | 0.43 |
| HT29_1_GR_ChDB_0004 | Thapsigargin | 10nM | 6h | YLPM1 | 0.00 | 0.43 |
| HT29_1_GR_ChDB_0004 | Thapsigargin | 10nM | 6h | GTF2F1 | 0.00 | 0.42 |
| HT29_1_GR_ChDB_0004 | Thapsigargin | 10nM | 6h | TCEB3 | 0.00 | 0.36 |
| HT29_1_GR_ChDB_0004 | Thapsigargin | 10nM | 6h | P4HB | 0.00 | 2.27 |
| HT29_1_GR_ChDB_0004 | Thapsigargin | 10nM | 6h | OSBP | 0.00 | 2.09 |
| HT29_1_GR_ChDB_0004 | Thapsigargin | 10nM | 6h | IRS2 | 0.00 | 2.01 |
| HT29_1_GR_ChDB_0004 | Thapsigargin | 10nM | 6h | AARS | 0.00 | 2.68 |
| HT29_1_GR_ChDB_0004 | Thapsigargin | 10nM | 6h | FHL2 | 0.00 | 2.12 |
| HT29_1_GR_ChDB_0004 | Thapsigargin | 10nM | 6h | ASNS | 0.00 | 6.27 |
| HT29_1_GR_ChDB_0004 | Thapsigargin | 10nM | 6h | ARMCX3 | 0.00 | 3.85 |
| HT29_1_GR_ChDB_0004 | Thapsigargin | 10nM | 6h | CDC6 | 0.00 | 2.08 |
| HT29_1_GR_ChDB_0004 | Thapsigargin | 10nM | 6h | PRNP | 0.00 | 2.10 |
| HT29_1_GR_ChDB_0004 | Thapsigargin | 10nM | 6h | SLC38A2 | 0.00 | 2.26 |
| HT29_1_GR_ChDB_0004 | Thapsigargin | 10nM | 6h | CKAP4 | 0.00 | 3.87 |
| HT29_1_GR_ChDB_0004 | Thapsigargin | 10nM | 6h | CREB3L2 | 0.00 | 3.39 |
| HT29_1_GR_ChDB_0004 | Thapsigargin | 10nM | 6h | BBIP1 | 0.00 | 2.26 |
| HT29_1_GR_ChDB_0004 | Thapsigargin | 10nM | 6h | LARP6 | 0.00 | 2.24 |
| HT29_1_GR_ChDB_0004 | Thapsigargin | 10nM | 6h | SERTAD3 | 0.00 | 2.23 |
| HT29_1_GR_ChDB_0004 | Thapsigargin | 10nM | 6h | EDEM3 | 0.00 | 2.22 |
| HT29_1_GR_ChDB_0004 | Thapsigargin | 10nM | 6h | MANF | 0.00 | 2.76 |
| HT29_1_GR_ChDB_0004 | Thapsigargin | 10nM | 6h | AGR2 | 0.00 | 6.77 |
| HT29_1_GR_ChDB_0004 | Thapsigargin | 10nM | 6h | HSPA5 | 0.00 | 3.30 |
| HT29_1_GR_ChDB_0004 | Thapsigargin | 10nM | 6h | TMCO3 | 0.00 | 2.66 |
| HT29_1_GR_ChDB_0004 | Thapsigargin | 10nM | 6h | PDIA4 | 0.00 | 2.21 |
| HT29_1_GR_ChDB_0004 | Thapsigargin | 10nM | 6h | PTPN18 | 0.00 | 2.27 |
| HT29_1_GR_ChDB_0004 | Thapsigargin | 10nM | 6h | MBNL2 | 0.00 | 4.56 |
| HT29_1_GR_ChDB_0004 | Thapsigargin | 10nM | 6h | EIF2AK3 | 0.00 | 3.33 |
| HT29_1_GR_ChDB_0004 | Thapsigargin | 10nM | 6h | GOLGB1 | 0.00 | 2.90 |
| HT29_1_GR_ChDB_0004 | Thapsigargin | 10nM | 6h | WFS1 | 0.00 | 2.53 |
| HT29_1_GR_ChDB_0004 | Thapsigargin | 10nM | 6h | FYN | 0.00 | 2.00 |
| HT29_1_GR_ChDB_0004 | Thapsigargin | 10nM | 6h | ATP8B2 | 0.00 | 3.89 |
| HT29_1_GR_ChDB_0004 | Thapsigargin | 10nM | 6h | FICD | 0.00 | 3.19 |
| HT29_1_GR_ChDB_0004 | Thapsigargin | 10nM | 6h | IFRD1 | 0.00 | 3.16 |
| HT29_1_GR_ChDB_0004 | Thapsigargin | 10nM | 6h | CARS | 0.00 | 2.33 |
| HT29_1_GR_ChDB_0004 | Thapsigargin | 10nM | 6h | SUCO | 0.00 | 2.01 |
| HT29_1_GR_ChDB_0004 | Thapsigargin | 10nM | 6h | ADK | 0.00 | 2.33 |
| HT29_1_GR_ChDB_0004 | Thapsigargin | 10nM | 6h | PSPH | 0.00 | 2.79 |
| HT29_1_GR_ChDB_0004 | Thapsigargin | 10nM | 6h | PIGA | 0.00 | 2.09 |
| HT29_1_GR_ChDB_0004 | Thapsigargin | 10nM | 6h | DNAJC3 | 0.00 | 3.09 |
| HT29_1_GR_ChDB_0004 | Thapsigargin | 10nM | 6h | DDIT3 | 0.00 | 13.15 |
| HT29_1_GR_ChDB_0004 | Thapsigargin | 10nM | 6h | MAGT1 | 0.00 | 2.61 |
| HT29_1_GR_ChDB_0004 | Thapsigargin | 10nM | 6h | CTH | 0.00 | 10.35 |
| HT29_1_GR_ChDB_0004 | Thapsigargin | 10nM | 6h | EAF2 | 0.00 | 2.40 |
| HT29_1_GR_ChDB_0004 | Thapsigargin | 10nM | 6h | MCTP2 | 0.00 | 2.20 |
| HT29_1_GR_ChDB_0004 | Thapsigargin | 10nM | 6h | HSPA13 | 0.00 | 4.10 |
| HT29_1_GR_ChDB_0004 | Thapsigargin | 10nM | 6h | SLC33A1 | 0.00 | 3.21 |
| HT29_1_GR_ChDB_0004 | Thapsigargin | 10nM | 6h | GOLT1B | 0.00 | 2.18 |
| HT29_1_GR_ChDB_0004 | Thapsigargin | 10nM | 6h | MIS12 | 0.00 | 2.05 |
| HT29_1_GR_ChDB_0004 | Thapsigargin | 10nM | 6h | GDF15 | 0.00 | 3.32 |
| HT29_1_GR_ChDB_0004 | Thapsigargin | 10nM | 6h | SMIM14 | 0.00 | 2.13 |
| HT29_1_GR_ChDB_0004 | Thapsigargin | 10nM | 6h | NUS1P3 | 0.00 | 2.03 |
| HT29_1_GR_ChDB_0004 | Thapsigargin | 10nM | 6h | SARS | 0.00 | 3.47 |
| HT29_1_GR_ChDB_0004 | Thapsigargin | 10nM | 6h | TES | 0.00 | 2.17 |
| HT29_1_GR_ChDB_0004 | Thapsigargin | 10nM | 6h | GFPT1 | 0.00 | 3.61 |
| HT29_1_GR_ChDB_0004 | Thapsigargin | 10nM | 6h | CHAC1 | 0.00 | 3.49 |
| HT29_1_GR_ChDB_0004 | Thapsigargin | 10nM | 6h | ENTPD7 | 0.00 | 2.55 |
| HT29_1_GR_ChDB_0004 | Thapsigargin | 10nM | 6h | URB2 | 0.00 | 0.49 |
| HT29_1_GR_ChDB_0004 | Thapsigargin | 10nM | 6h | GAS2L1 | 0.00 | 0.40 |
| HT29_1_GR_ChDB_0004 | Thapsigargin | 10nM | 6h | DIXDC1 | 0.00 | 0.49 |
| HT29_1_GR_ChDB_0004 | Thapsigargin | 10nM | 6h | EIF4G1 | 0.00 | 0.46 |
| HT29_1_GR_ChDB_0004 | Thapsigargin | 10nM | 6h | DICER1 | 0.00 | 0.49 |
| HT29_1_GR_ChDB_0004 | Thapsigargin | 10nM | 6h | BICD2 | 0.00 | 0.41 |
| HT29_1_GR_ChDB_0004 | Thapsigargin | 10nM | 6h | CHAF1A | 0.00 | 0.45 |
| HT29_1_GR_ChDB_0004 | Thapsigargin | 10nM | 6h | TM9SF4 | 0.00 | 0.46 |
| HT29_1_GR_ChDB_0004 | Thapsigargin | 10nM | 6h | MAP4 | 0.00 | 0.44 |
| HT29_1_GR_ChDB_0004 | Thapsigargin | 10nM | 6h | ELAVL1 | 0.00 | 0.45 |
| HT29_1_GR_ChDB_0004 | Thapsigargin | 10nM | 6h | EP400 | 0.00 | 0.44 |
| HT29_1_GR_ChDB_0004 | Thapsigargin | 10nM | 6h | RRP1 | 0.00 | 0.44 |
| HT29_1_GR_ChDB_0004 | Thapsigargin | 10nM | 6h | AP3B1 | 0.00 | 0.46 |
| HT29_1_GR_ChDB_0004 | Thapsigargin | 10nM | 6h | HSPH1 | 0.00 | 0.32 |
| HT29_1_GR_ChDB_0004 | Thapsigargin | 10nM | 6h | USP10 | 0.00 | 0.42 |
| HT29_1_GR_ChDB_0004 | Thapsigargin | 10nM | 6h | TGOLN2 | 0.00 | 0.49 |
| HT29_1_GR_ChDB_0004 | Thapsigargin | 10nM | 6h | OSBPL11 | 0.00 | 0.47 |
| HT29_1_GR_ChDB_0004 | Thapsigargin | 10nM | 6h | ATAD2 | 0.00 | 0.42 |
| HT29_1_GR_ChDB_0004 | Thapsigargin | 10nM | 6h | ZMAT3 | 0.00 | 0.42 |
| HT29_1_GR_ChDB_0004 | Thapsigargin | 10nM | 6h | TFAM | 0.00 | 0.47 |
| HT29_1_GR_ChDB_0004 | Thapsigargin | 10nM | 6h | HNRNPR | 0.00 | 0.48 |
| HT29_1_GR_ChDB_0004 | Thapsigargin | 10nM | 6h | ADCY3 | 0.00 | 0.44 |
| HT29_1_GR_ChDB_0004 | Thapsigargin | 10nM | 6h | UTP20 | 0.00 | 0.46 |
| HT29_1_GR_ChDB_0004 | Thapsigargin | 10nM | 6h | KAZN | 0.00 | 0.46 |
| HT29_1_GR_ChDB_0004 | Thapsigargin | 10nM | 6h | LIMA1 | 0.00 | 0.44 |
| HT29_1_GR_ChDB_0012 | 17-AAG | 100nM | 6h | PIK3R1 | 1.00 | 0.36 |
| HT29_10_GR_ChDB_0075 | Gemcitabine | 1uM | 16h | MYC | 1.00 | 0.36 |
| HT29_10_GR_ChDB_0075 | Gemcitabine | 1uM | 16h | NDUFAF4 | 0.38 | 0.40 |
| HT29_10_GR_ChDB_0075 | Gemcitabine | 1uM | 16h | TIMP2 | 0.12 | 3.56 |
| HT29_10_GR_ChDB_0075 | Gemcitabine | 1uM | 16h | RPS6KB1 | 0.11 | 0.44 |
| HT29_10_GR_ChDB_0075 | Gemcitabine | 1uM | 16h | MYB | 0.11 | 0.44 |
| HT29_10_GR_ChDB_0075 | Gemcitabine | 1uM | 16h | MMP7 | 0.06 | 2.04 |
| HT29_10_GR_ChDB_0075 | Gemcitabine | 1uM | 16h | PLK2 | 0.05 | 2.06 |
| HT29_10_GR_ChDB_0075 | Gemcitabine | 1uM | 16h | HIST1H2BK | 0.03 | 0.47 |
| HT29_10_GR_ChDB_0075 | Gemcitabine | 1uM | 16h | ARNTL | 0.03 | 2.18 |
| HT29_10_GR_ChDB_0075 | Gemcitabine | 1uM | 16h | PRKACB | 0.03 | 2.00 |
| HT29_10_GR_ChDB_0075 | Gemcitabine | 1uM | 16h | CREB3 | 0.03 | 2.19 |
| HT29_10_GR_ChDB_0075 | Gemcitabine | 1uM | 16h | SQRDL | 0.02 | 2.05 |
| HT29_10_GR_ChDB_0075 | Gemcitabine | 1uM | 16h | ADM | 0.02 | 2.00 |
| HT29_10_GR_ChDB_0075 | Gemcitabine | 1uM | 16h | SFN | 0.02 | 2.10 |
| HT29_10_GR_ChDB_0075 | Gemcitabine | 1uM | 16h | CDKN1A | 0.02 | 2.14 |
| HT29_10_GR_ChDB_0075 | Gemcitabine | 1uM | 16h | PRKX | 0.02 | 0.48 |
| HT29_10_GR_ChDB_0075 | Gemcitabine | 1uM | 16h | PLK4 | 0.02 | 0.47 |
| HT29_10_GR_ChDB_0075 | Gemcitabine | 1uM | 16h | TM7SF2 | 0.02 | 3.01 |
| HT29_10_GR_ChDB_0075 | Gemcitabine | 1uM | 16h | TOX2 | 0.02 | 2.04 |
| HT29_10_GR_ChDB_0075 | Gemcitabine | 1uM | 16h | MBNL2 | 0.02 | 2.01 |
| HT29_10_GR_ChDB_0075 | Gemcitabine | 1uM | 16h | RXRA | 0.02 | 0.45 |
| HT29_10_GR_ChDB_0075 | Gemcitabine | 1uM | 16h | ARL4C | 0.01 | 2.58 |
| HT29_10_GR_ChDB_0075 | Gemcitabine | 1uM | 16h | SULT1A1 | 0.01 | 2.46 |
| HT29_10_GR_ChDB_0075 | Gemcitabine | 1uM | 16h | CREB1 | 0.01 | 0.42 |
| HT29_10_GR_ChDB_0075 | Gemcitabine | 1uM | 16h | BCCIP | 0.01 | 0.46 |
| HT29_10_GR_ChDB_0075 | Gemcitabine | 1uM | 16h | MED1 | 0.01 | 0.40 |
| HT29_10_GR_ChDB_0075 | Gemcitabine | 1uM | 16h | KLF4 | 0.01 | 2.61 |
| HT29_10_GR_ChDB_0075 | Gemcitabine | 1uM | 16h | GCAT | 0.01 | 2.28 |
| HT29_10_GR_ChDB_0075 | Gemcitabine | 1uM | 16h | PPIH | 0.01 | 0.35 |
| HT29_10_GR_ChDB_0075 | Gemcitabine | 1uM | 16h | STK11 | 0.01 | 0.46 |
| HT29_10_GR_ChDB_0075 | Gemcitabine | 1uM | 16h | SMAD3 | 0.01 | 2.93 |
| HT29_10_GR_ChDB_0075 | Gemcitabine | 1uM | 16h | DDIT3 | 0.01 | 2.51 |
| HT29_10_GR_ChDB_0075 | Gemcitabine | 1uM | 16h | FDX1 | 0.01 | 0.42 |
| HT29_10_GR_ChDB_0075 | Gemcitabine | 1uM | 16h | NOTCH1 | 0.01 | 0.46 |
| HT29_10_GR_ChDB_0075 | Gemcitabine | 1uM | 16h | BLVRA | 0.01 | 2.58 |
| HT29_10_GR_ChDB_0075 | Gemcitabine | 1uM | 16h | TUBB2A | 0.01 | 2.09 |
| HT29_10_GR_ChDB_0075 | Gemcitabine | 1uM | 16h | LGALS8 | 0.01 | 2.09 |
| HT29_10_GR_ChDB_0075 | Gemcitabine | 1uM | 16h | MXD1 | 0.01 | 2.54 |
| HT29_10_GR_ChDB_0075 | Gemcitabine | 1uM | 16h | MTAP | 0.01 | 0.42 |
| HT29_10_GR_ChDB_0075 | Gemcitabine | 1uM | 16h | SLC22A18 | 0.01 | 2.07 |
| HT29_10_GR_ChDB_0075 | Gemcitabine | 1uM | 16h | AKR1C1 | 0.01 | 3.30 |
| HT29_10_GR_ChDB_0075 | Gemcitabine | 1uM | 16h | RRP12 | 0.01 | 0.45 |
| HT29_10_GR_ChDB_0075 | Gemcitabine | 1uM | 16h | GALNS | 0.01 | 2.08 |
| HT29_10_GR_ChDB_0075 | Gemcitabine | 1uM | 16h | HPGD | 0.00 | 3.45 |
| HT29_10_GR_ChDB_0075 | Gemcitabine | 1uM | 16h | RRS1 | 0.00 | 0.32 |
| HT29_10_GR_ChDB_0075 | Gemcitabine | 1uM | 16h | OPN3 | 0.00 | 0.37 |
| HT29_10_GR_ChDB_0075 | Gemcitabine | 1uM | 16h | GMEB1 | 0.00 | 0.48 |
| HT29_10_GR_ChDB_0075 | Gemcitabine | 1uM | 16h | CAV1 | 0.00 | 2.03 |
| HT29_10_GR_ChDB_0075 | Gemcitabine | 1uM | 16h | G3BP1 | 0.00 | 0.49 |
| HT29_10_GR_ChDB_0075 | Gemcitabine | 1uM | 16h | FN1 | 0.00 | 5.09 |
| HT29_10_GR_ChDB_0075 | Gemcitabine | 1uM | 16h | IL1R2 | 0.00 | 2.11 |
| HT29_10_GR_ChDB_0075 | Gemcitabine | 1uM | 16h | PLAUR | 0.00 | 2.21 |
| HT29_10_GR_ChDB_0075 | Gemcitabine | 1uM | 16h | TPMT | 0.00 | 2.42 |
| HT29_10_GR_ChDB_0075 | Gemcitabine | 1uM | 16h | ASS1 | 0.00 | 2.01 |
| HT29_10_GR_ChDB_0075 | Gemcitabine | 1uM | 16h | STAT2 | 0.00 | 2.11 |
| HT29_10_GR_ChDB_0075 | Gemcitabine | 1uM | 16h | ARG2 | 0.00 | 2.46 |
| HT29_10_GR_ChDB_0075 | Gemcitabine | 1uM | 16h | HEATR1 | 0.00 | 0.46 |
| HT29_10_GR_ChDB_0075 | Gemcitabine | 1uM | 16h | ATP6V1D | 0.00 | 2.04 |
| HT29_10_GR_ChDB_0075 | Gemcitabine | 1uM | 16h | PRSS23 | 0.00 | 2.60 |
| HT29_10_GR_ChDB_0075 | Gemcitabine | 1uM | 16h | PWP1 | 0.00 | 0.50 |
| HT29_10_GR_ChDB_0075 | Gemcitabine | 1uM | 16h | IL8 | 0.00 | 3.50 |
| HT29_10_GR_ChDB_0075 | Gemcitabine | 1uM | 16h | SERPINA1 | 0.00 | 2.45 |
| HT29_10_GR_ChDB_0075 | Gemcitabine | 1uM | 16h | RHOD | 0.00 | 2.82 |
| HT29_10_GR_ChDB_0075 | Gemcitabine | 1uM | 16h | NFATC3 | 0.00 | 0.48 |
| HT29_10_GR_ChDB_0075 | Gemcitabine | 1uM | 16h | CRCP | 0.00 | 0.45 |
| HT29_10_GR_ChDB_0075 | Gemcitabine | 1uM | 16h | G2E3 | 0.00 | 0.45 |
| HT29_10_GR_ChDB_0075 | Gemcitabine | 1uM | 16h | F2R | 0.00 | 2.53 |
| HT29_10_GR_ChDB_0075 | Gemcitabine | 1uM | 16h | GTF2H2 | 0.00 | 0.46 |
| HT29_10_GR_ChDB_0075 | Gemcitabine | 1uM | 16h | OAS1 | 0.00 | 2.23 |
| HT29_10_GR_ChDB_0075 | Gemcitabine | 1uM | 16h | ID1 | 0.00 | 2.27 |
| HT29_10_GR_ChDB_0075 | Gemcitabine | 1uM | 16h | NDUFS1 | 0.00 | 0.49 |
| HT29_10_GR_ChDB_0075 | Gemcitabine | 1uM | 16h | YWHAQ | 0.00 | 0.46 |
| HT29_10_GR_ChDB_0075 | Gemcitabine | 1uM | 16h | CYP3A5 | 0.00 | 2.11 |
| HT29_10_GR_ChDB_0075 | Gemcitabine | 1uM | 16h | TESK1 | 0.00 | 2.19 |
| HT29_10_GR_ChDB_0075 | Gemcitabine | 1uM | 16h | SBNO1 | 0.00 | 0.45 |
| HT29_10_GR_ChDB_0075 | Gemcitabine | 1uM | 16h | DLGAP5 | 0.00 | 0.45 |
| HT29_10_GR_ChDB_0075 | Gemcitabine | 1uM | 16h | NGEF | 0.00 | 2.04 |
| HT29_10_GR_ChDB_0075 | Gemcitabine | 1uM | 16h | MEF2A | 0.00 | 2.13 |
| HT29_10_GR_ChDB_0075 | Gemcitabine | 1uM | 16h | PHF17 | 0.00 | 0.37 |
| HT29_10_GR_ChDB_0075 | Gemcitabine | 1uM | 16h | NAGK | 0.00 | 2.30 |
| HT29_10_GR_ChDB_0075 | Gemcitabine | 1uM | 16h | ZFX | 0.00 | 0.45 |
| HT29_10_GR_ChDB_0075 | Gemcitabine | 1uM | 16h | HADHA | 0.00 | 2.16 |
| HT29_10_GR_ChDB_0075 | Gemcitabine | 1uM | 16h | RBL2 | 0.00 | 0.49 |
| HT29_10_GR_ChDB_0075 | Gemcitabine | 1uM | 16h | KDELR3 | 0.00 | 2.00 |
| HT29_10_GR_ChDB_0075 | Gemcitabine | 1uM | 16h | GADD45A | 0.00 | 2.29 |
| HT29_10_GR_ChDB_0075 | Gemcitabine | 1uM | 16h | GLRX | 0.00 | 2.61 |
| HT29_10_GR_ChDB_0075 | Gemcitabine | 1uM | 16h | ZNF207 | 0.00 | 0.46 |
| HT29_10_GR_ChDB_0075 | Gemcitabine | 1uM | 16h | ZNF22 | 0.00 | 0.44 |
| HT29_10_GR_ChDB_0075 | Gemcitabine | 1uM | 16h | RNF19B | 0.00 | 2.05 |
| HT29_10_GR_ChDB_0075 | Gemcitabine | 1uM | 16h | HYAL1 | 0.00 | 2.01 |
| HT29_10_GR_ChDB_0075 | Gemcitabine | 1uM | 16h | CDK5 | 0.00 | 2.12 |
| HT29_10_GR_ChDB_0075 | Gemcitabine | 1uM | 16h | HSPA2 | 0.00 | 2.18 |
| HT29_10_GR_ChDB_0075 | Gemcitabine | 1uM | 16h | SWAP70 | 0.00 | 2.15 |
| HT29_10_GR_ChDB_0075 | Gemcitabine | 1uM | 16h | IL18 | 0.00 | 2.14 |
| HT29_10_GR_ChDB_0075 | Gemcitabine | 1uM | 16h | CTSL2 | 0.00 | 3.35 |
| HT29_10_GR_ChDB_0075 | Gemcitabine | 1uM | 16h | SMURF2 | 0.00 | 2.12 |
| HT29_10_GR_ChDB_0075 | Gemcitabine | 1uM | 16h | TACC3 | 0.00 | 0.49 |
| HT29_10_GR_ChDB_0075 | Gemcitabine | 1uM | 16h | SRSF8 | 0.00 | 0.49 |
| HT29_10_GR_ChDB_0075 | Gemcitabine | 1uM | 16h | TP53BP2 | 0.00 | 0.38 |
| HT29_10_GR_ChDB_0075 | Gemcitabine | 1uM | 16h | PHYH | 0.00 | 2.02 |
| HT29_10_GR_ChDB_0075 | Gemcitabine | 1uM | 16h | MDM4 | 0.00 | 0.41 |
| HT29_10_GR_ChDB_0075 | Gemcitabine | 1uM | 16h | PPP3CB | 0.00 | 0.40 |
| HT29_10_GR_ChDB_0075 | Gemcitabine | 1uM | 16h | BAD | 0.00 | 2.35 |
| HT29_10_GR_ChDB_0075 | Gemcitabine | 1uM | 16h | DHRS2 | 0.00 | 0.18 |
| HT29_10_GR_ChDB_0075 | Gemcitabine | 1uM | 16h | LYAR | 0.00 | 0.50 |
| HT29_10_GR_ChDB_0075 | Gemcitabine | 1uM | 16h | CD55 | 0.00 | 2.09 |
| HT29_10_GR_ChDB_0075 | Gemcitabine | 1uM | 16h | NEU1 | 0.00 | 3.45 |
| HT29_10_GR_ChDB_0075 | Gemcitabine | 1uM | 16h | CD59 | 0.00 | 2.30 |
| HT29_10_GR_ChDB_0075 | Gemcitabine | 1uM | 16h | GTF2H3 | 0.00 | 0.47 |
| HT29_10_GR_ChDB_0079 | Methotrexate | 1uM | 16h | MYC | 1.00 | 0.23 |
| HT29_10_GR_ChDB_0079 | Methotrexate | 1uM | 16h | NUDT5 | 0.25 | 0.44 |
| HT29_10_GR_ChDB_0079 | Methotrexate | 1uM | 16h | BRAF | 0.17 | 2.14 |
| HT29_10_GR_ChDB_0079 | Methotrexate | 1uM | 16h | NDUFAF4 | 0.14 | 0.28 |
| HT29_10_GR_ChDB_0079 | Methotrexate | 1uM | 16h | AURKA | 0.11 | 0.34 |
| HT29_10_GR_ChDB_0079 | Methotrexate | 1uM | 16h | MAPK8 | 0.11 | 0.47 |
| HT29_10_GR_ChDB_0079 | Methotrexate | 1uM | 16h | MMP7 | 0.05 | 2.81 |
| HT29_10_GR_ChDB_0079 | Methotrexate | 1uM | 16h | TIMP2 | 0.04 | 3.18 |
| HT29_10_GR_ChDB_0079 | Methotrexate | 1uM | 16h | PIK3R1 | 0.04 | 0.48 |
| HT29_10_GR_ChDB_0079 | Methotrexate | 1uM | 16h | GRK6 | 0.04 | 0.45 |
| HT29_10_GR_ChDB_0079 | Methotrexate | 1uM | 16h | BUB1 | 0.03 | 0.45 |
| HT29_10_GR_ChDB_0079 | Methotrexate | 1uM | 16h | BCR | 0.03 | 0.49 |
| HT29_10_GR_ChDB_0079 | Methotrexate | 1uM | 16h | KLF6 | 0.03 | 2.11 |
| HT29_10_GR_ChDB_0079 | Methotrexate | 1uM | 16h | DKK1 | 0.02 | 2.59 |
| HT29_10_GR_ChDB_0079 | Methotrexate | 1uM | 16h | RIPK2 | 0.02 | 2.39 |
| HT29_10_GR_ChDB_0079 | Methotrexate | 1uM | 16h | CXCR4 | 0.02 | 0.29 |
| HT29_10_GR_ChDB_0079 | Methotrexate | 1uM | 16h | CENPE | 0.02 | 0.47 |
| HT29_10_GR_ChDB_0079 | Methotrexate | 1uM | 16h | RUVBL1 | 0.01 | 0.31 |
| HT29_10_GR_ChDB_0079 | Methotrexate | 1uM | 16h | PLK2 | 0.01 | 2.12 |
| HT29_10_GR_ChDB_0079 | Methotrexate | 1uM | 16h | FOSL2 | 0.01 | 3.11 |
| HT29_10_GR_ChDB_0079 | Methotrexate | 1uM | 16h | AKR1C1 | 0.01 | 3.87 |
| HT29_10_GR_ChDB_0079 | Methotrexate | 1uM | 16h | IL15 | 0.01 | 2.52 |
| HT29_10_GR_ChDB_0079 | Methotrexate | 1uM | 16h | PSME1 | 0.01 | 2.11 |
| HT29_10_GR_ChDB_0079 | Methotrexate | 1uM | 16h | ABCD3 | 0.01 | 0.22 |
| HT29_10_GR_ChDB_0079 | Methotrexate | 1uM | 16h | TRIM29 | 0.01 | 2.76 |
| HT29_10_GR_ChDB_0079 | Methotrexate | 1uM | 16h | ETNK1 | 0.01 | 0.31 |
| HT29_10_GR_ChDB_0079 | Methotrexate | 1uM | 16h | LGALS8 | 0.01 | 2.32 |
| HT29_10_GR_ChDB_0079 | Methotrexate | 1uM | 16h | TUBB2A | 0.01 | 2.18 |
| HT29_10_GR_ChDB_0079 | Methotrexate | 1uM | 16h | SMAD3 | 0.01 | 4.25 |
| HT29_10_GR_ChDB_0079 | Methotrexate | 1uM | 16h | EBNA1BP2 | 0.01 | 0.36 |
| HT29_10_GR_ChDB_0079 | Methotrexate | 1uM | 16h | DDB2 | 0.01 | 2.16 |
| HT29_10_GR_ChDB_0079 | Methotrexate | 1uM | 16h | PDXK | 0.01 | 0.48 |
| HT29_10_GR_ChDB_0079 | Methotrexate | 1uM | 16h | HNMT | 0.01 | 2.35 |
| HT29_10_GR_ChDB_0079 | Methotrexate | 1uM | 16h | CTSL2 | 0.01 | 2.94 |
| HT29_10_GR_ChDB_0079 | Methotrexate | 1uM | 16h | P2RX4 | 0.01 | 2.37 |
| HT29_10_GR_ChDB_0079 | Methotrexate | 1uM | 16h | NSDHL | 0.01 | 0.47 |
| HT29_10_GR_ChDB_0079 | Methotrexate | 1uM | 16h | TFPI | 0.01 | 2.57 |
| HT29_10_GR_ChDB_0079 | Methotrexate | 1uM | 16h | IL18 | 0.01 | 3.65 |
| HT29_10_GR_ChDB_0079 | Methotrexate | 1uM | 16h | HSF2 | 0.01 | 2.13 |
| HT29_10_GR_ChDB_0079 | Methotrexate | 1uM | 16h | HSP90AA1 | 0.01 | 0.47 |
| HT29_10_GR_ChDB_0079 | Methotrexate | 1uM | 16h | ARL4C | 0.01 | 2.81 |
| HT29_10_GR_ChDB_0079 | Methotrexate | 1uM | 16h | SENP2 | 0.01 | 2.00 |
| HT29_10_GR_ChDB_0079 | Methotrexate | 1uM | 16h | RBBP4 | 0.01 | 0.48 |
| HT29_10_GR_ChDB_0079 | Methotrexate | 1uM | 16h | NUP88 | 0.01 | 0.43 |
| HT29_10_GR_ChDB_0079 | Methotrexate | 1uM | 16h | RAB27A | 0.01 | 2.08 |
| HT29_10_GR_ChDB_0079 | Methotrexate | 1uM | 16h | BAMBI | 0.01 | 0.44 |
| HT29_10_GR_ChDB_0079 | Methotrexate | 1uM | 16h | POLR3K | 0.01 | 0.47 |
| HT29_10_GR_ChDB_0079 | Methotrexate | 1uM | 16h | HPGD | 0.01 | 3.95 |
| HT29_10_GR_ChDB_0079 | Methotrexate | 1uM | 16h | RRP12 | 0.01 | 0.41 |
| HT29_10_GR_ChDB_0079 | Methotrexate | 1uM | 16h | SH3RF2 | 0.01 | 2.17 |
| HT29_10_GR_ChDB_0079 | Methotrexate | 1uM | 16h | FN1 | 0.01 | 3.91 |
| HT29_10_GR_ChDB_0079 | Methotrexate | 1uM | 16h | DDAH2 | 0.01 | 3.06 |
| HT29_10_GR_ChDB_0079 | Methotrexate | 1uM | 16h | LIPH | 0.01 | 2.35 |
| HT29_10_GR_ChDB_0079 | Methotrexate | 1uM | 16h | RRS1 | 0.01 | 0.27 |
| HT29_10_GR_ChDB_0079 | Methotrexate | 1uM | 16h | SLC25A12 | 0.01 | 0.44 |
| HT29_10_GR_ChDB_0079 | Methotrexate | 1uM | 16h | ABHD2 | 0.01 | 2.14 |
| HT29_10_GR_ChDB_0079 | Methotrexate | 1uM | 16h | SNAPC4 | 0.01 | 0.38 |
| HT29_10_GR_ChDB_0079 | Methotrexate | 1uM | 16h | MCM7 | 0.01 | 0.49 |
| HT29_10_GR_ChDB_0079 | Methotrexate | 1uM | 16h | PHB | 0.01 | 0.37 |
| HT29_10_GR_ChDB_0079 | Methotrexate | 1uM | 16h | SQLE | 0.01 | 0.45 |
| HT29_10_GR_ChDB_0079 | Methotrexate | 1uM | 16h | PRMT3 | 0.01 | 0.47 |
| HT29_10_GR_ChDB_0079 | Methotrexate | 1uM | 16h | HMGCS1 | 0.01 | 0.44 |
| HT29_10_GR_ChDB_0079 | Methotrexate | 1uM | 16h | GSTZ1 | 0.00 | 2.61 |
| HT29_10_GR_ChDB_0079 | Methotrexate | 1uM | 16h | NAGK | 0.00 | 3.01 |
| HT29_10_GR_ChDB_0079 | Methotrexate | 1uM | 16h | TUBB3 | 0.00 | 0.37 |
| HT29_10_GR_ChDB_0079 | Methotrexate | 1uM | 16h | BIK | 0.00 | 2.01 |
| HT29_10_GR_ChDB_0079 | Methotrexate | 1uM | 16h | BCCIP | 0.00 | 0.34 |
| HT29_10_GR_ChDB_0079 | Methotrexate | 1uM | 16h | PPP3CB | 0.00 | 0.49 |
| HT29_10_GR_ChDB_0079 | Methotrexate | 1uM | 16h | POLR1C | 0.00 | 0.50 |
| HT29_10_GR_ChDB_0079 | Methotrexate | 1uM | 16h | HSPA4 | 0.00 | 0.37 |
| HT29_10_GR_ChDB_0079 | Methotrexate | 1uM | 16h | MNT | 0.00 | 2.05 |
| HT29_10_GR_ChDB_0079 | Methotrexate | 1uM | 16h | MCCC1 | 0.00 | 2.25 |
| HT29_10_GR_ChDB_0079 | Methotrexate | 1uM | 16h | ELAVL1 | 0.00 | 0.47 |
| HT29_10_GR_ChDB_0079 | Methotrexate | 1uM | 16h | PPARGC1B | 0.00 | 0.37 |
| HT29_10_GR_ChDB_0079 | Methotrexate | 1uM | 16h | RNASE4 | 0.00 | 2.48 |
| HT29_10_GR_ChDB_0079 | Methotrexate | 1uM | 16h | JMJD6 | 0.00 | 0.40 |
| HT29_10_GR_ChDB_0079 | Methotrexate | 1uM | 16h | SMURF2 | 0.00 | 3.31 |
| HT29_10_GR_ChDB_0079 | Methotrexate | 1uM | 16h | FOSL1 | 0.00 | 0.41 |
| HT29_10_GR_ChDB_0079 | Methotrexate | 1uM | 16h | PRSS23 | 0.00 | 4.14 |
| HT29_10_GR_ChDB_0079 | Methotrexate | 1uM | 16h | TM7SF2 | 0.00 | 2.41 |
| HT29_10_GR_ChDB_0079 | Methotrexate | 1uM | 16h | PTGS2 | 0.00 | 4.54 |
| HT29_10_GR_ChDB_0079 | Methotrexate | 1uM | 16h | PCSK9 | 0.00 | 0.45 |
| HT29_10_GR_ChDB_0079 | Methotrexate | 1uM | 16h | HSPA14 | 0.00 | 0.34 |
| HT29_10_GR_ChDB_0079 | Methotrexate | 1uM | 16h | AKR1C3 | 0.00 | 2.06 |
| HT29_10_GR_ChDB_0079 | Methotrexate | 1uM | 16h | TARDBP | 0.00 | 0.40 |
| HT29_10_GR_ChDB_0079 | Methotrexate | 1uM | 16h | NEU1 | 0.00 | 4.70 |
| HT29_10_GR_ChDB_0079 | Methotrexate | 1uM | 16h | SRRT | 0.00 | 0.40 |
| HT29_10_GR_ChDB_0079 | Methotrexate | 1uM | 16h | EWSR1 | 0.00 | 0.44 |
| HT29_10_GR_ChDB_0079 | Methotrexate | 1uM | 16h | G2E3 | 0.00 | 0.35 |
| HT29_10_GR_ChDB_0079 | Methotrexate | 1uM | 16h | GCAT | 0.00 | 2.43 |
| HT29_10_GR_ChDB_0079 | Methotrexate | 1uM | 16h | EXOSC4 | 0.00 | 0.39 |
| HT29_10_GR_ChDB_0079 | Methotrexate | 1uM | 16h | HCFC1 | 0.00 | 0.49 |
| HT29_10_GR_ChDB_0079 | Methotrexate | 1uM | 16h | PMM2 | 0.00 | 0.46 |
| HT29_10_GR_ChDB_0079 | Methotrexate | 1uM | 16h | WDR67 | 0.00 | 0.48 |
| HT29_10_GR_ChDB_0079 | Methotrexate | 1uM | 16h | TOMM40 | 0.00 | 0.42 |
| HT29_10_GR_ChDB_0079 | Methotrexate | 1uM | 16h | SERINC3 | 0.00 | 2.43 |
| HT29_10_GR_ChDB_0079 | Methotrexate | 1uM | 16h | NAA50 | 0.00 | 0.42 |
| HT29_10_GR_ChDB_0079 | Methotrexate | 1uM | 16h | SOS2 | 0.00 | 2.95 |
| HT29_10_GR_ChDB_0079 | Methotrexate | 1uM | 16h | SAT1 | 0.00 | 2.93 |
| HT29_10_GR_ChDB_0079 | Methotrexate | 1uM | 16h | CCDC86 | 0.00 | 0.31 |
| HT29_10_GR_ChDB_0079 | Methotrexate | 1uM | 16h | IP6K2 | 0.00 | 3.19 |
| HT29_10_GR_ChDB_0079 | Methotrexate | 1uM | 16h | IL8 | 0.00 | 3.21 |
| HT29_10_GR_ChDB_0079 | Methotrexate | 1uM | 16h | PBXIP1 | 0.00 | 2.59 |
| HT29_10_GR_ChDB_0079 | Methotrexate | 1uM | 16h | SLC5A6 | 0.00 | 0.40 |
| HT29_10_GR_ChDB_0079 | Methotrexate | 1uM | 16h | STAT2 | 0.00 | 2.50 |
| HT29_10_GR_ChDB_0079 | Methotrexate | 1uM | 16h | GRWD1 | 0.00 | 0.37 |
| HT29_10_GR_ChDB_0079 | Methotrexate | 1uM | 16h | RARRES3 | 0.00 | 3.43 |
| HT29_10_GR_ChDB_0079 | Methotrexate | 1uM | 16h | HSP90B1 | 0.00 | 0.48 |
| HT29_10_GR_ChDB_0079 | Methotrexate | 1uM | 16h | MAT2A | 0.00 | 0.36 |
| HT29_10_GR_ChDB_0079 | Methotrexate | 1uM | 16h | SPAG7 | 0.00 | 2.11 |
| HT29_10_GR_ChDB_0079 | Methotrexate | 1uM | 16h | TCEB3 | 0.00 | 0.48 |
| HT29_10_GR_ChDB_0079 | Methotrexate | 1uM | 16h | ATP5S | 0.00 | 2.35 |
| HT29_10_GR_ChDB_0079 | Methotrexate | 1uM | 16h | BNIP2 | 0.00 | 0.25 |
| HT29_10_GR_ChDB_0079 | Methotrexate | 1uM | 16h | UBE2N | 0.00 | 0.48 |
| HT29_10_GR_ChDB_0079 | Methotrexate | 1uM | 16h | BNIP3L | 0.00 | 2.02 |
| HT29_10_GR_ChDB_0079 | Methotrexate | 1uM | 16h | TOP1 | 0.00 | 0.45 |
| HT29_10_GR_ChDB_0079 | Methotrexate | 1uM | 16h | GLS | 0.00 | 0.42 |
| HT29_10_GR_ChDB_0079 | Methotrexate | 1uM | 16h | JUN | 0.00 | 2.89 |
| HT29_10_GR_ChDB_0079 | Methotrexate | 1uM | 16h | RRAGC | 0.00 | 2.21 |
| HT29_10_GR_ChDB_0079 | Methotrexate | 1uM | 16h | DDX5 | 0.00 | 0.48 |
| HT29_10_GR_ChDB_0079 | Methotrexate | 1uM | 16h | ARG2 | 0.00 | 2.24 |
| HT29_10_GR_ChDB_0079 | Methotrexate | 1uM | 16h | CASP9 | 0.00 | 2.01 |
| HT29_10_GR_ChDB_0079 | Methotrexate | 1uM | 16h | PSRC1 | 0.00 | 0.47 |
| HT29_10_GR_ChDB_0079 | Methotrexate | 1uM | 16h | ALDH3B1 | 0.00 | 2.37 |
| HT29_10_GR_ChDB_0079 | Methotrexate | 1uM | 16h | DCUN1D4 | 0.00 | 0.32 |
| HT29_10_GR_ChDB_0079 | Methotrexate | 1uM | 16h | EIF3J | 0.00 | 0.47 |
| HT29_10_GR_ChDB_0079 | Methotrexate | 1uM | 16h | PAQR8 | 0.00 | 2.64 |
| HT29_10_GR_ChDB_0079 | Methotrexate | 1uM | 16h | CYP20A1 | 0.00 | 2.20 |
| HT29_10_GR_ChDB_0079 | Methotrexate | 1uM | 16h | STK19 | 0.00 | 2.37 |
| HT29_10_GR_ChDB_0079 | Methotrexate | 1uM | 16h | KIF20A | 0.00 | 0.25 |
| HT29_10_GR_ChDB_0079 | Methotrexate | 1uM | 16h | EEF2K | 0.00 | 0.39 |
| HT29_10_GR_ChDB_0079 | Methotrexate | 1uM | 16h | G3BP1 | 0.00 | 0.34 |
| HT29_10_GR_ChDB_0079 | Methotrexate | 1uM | 16h | NOLC1 | 0.00 | 0.37 |
| HT29_10_GR_ChDB_0079 | Methotrexate | 1uM | 16h | PUS7L | 0.00 | 0.48 |
| HT29_10_GR_ChDB_0079 | Methotrexate | 1uM | 16h | MPHOSPH9 | 0.00 | 0.47 |
| HT29_10_GR_ChDB_0079 | Methotrexate | 1uM | 16h | FDFT1 | 0.00 | 0.47 |
| HT29_10_GR_ChDB_0079 | Methotrexate | 1uM | 16h | KIF14 | 0.00 | 0.41 |
| HT29_10_GR_ChDB_0079 | Methotrexate | 1uM | 16h | PRKAR1A | 0.00 | 2.49 |
| HT29_10_GR_ChDB_0079 | Methotrexate | 1uM | 16h | HEATR1 | 0.00 | 0.45 |
| HT29_10_GR_ChDB_0079 | Methotrexate | 1uM | 16h | ADI1 | 0.00 | 2.18 |
| HT29_10_GR_ChDB_0079 | Methotrexate | 1uM | 16h | SGK2 | 0.00 | 0.43 |
| HT29_10_GR_ChDB_0079 | Methotrexate | 1uM | 16h | ALDH6A1 | 0.00 | 2.48 |
| HT29_10_GR_ChDB_0079 | Methotrexate | 1uM | 16h | SKP2 | 0.00 | 0.43 |
| HT29_10_GR_ChDB_0079 | Methotrexate | 1uM | 16h | CCNB1 | 0.00 | 0.30 |
| HT29_10_GR_ChDB_0079 | Methotrexate | 1uM | 16h | IFNGR1 | 0.00 | 2.70 |
| HT29_10_GR_ChDB_0079 | Methotrexate | 1uM | 16h | AIMP2 | 0.00 | 0.48 |
| HT29_10_GR_ChDB_0079 | Methotrexate | 1uM | 16h | SLC6A8 | 0.00 | 0.34 |
| HT29_10_GR_ChDB_0079 | Methotrexate | 1uM | 16h | ARNTL2 | 0.00 | 2.05 |
| HT29_10_GR_ChDB_0079 | Methotrexate | 1uM | 16h | ZBTB26 | 0.00 | 2.02 |
| HT29_10_GR_ChDB_0079 | Methotrexate | 1uM | 16h | FKBP4 | 0.00 | 0.44 |
| HT29_10_GR_ChDB_0079 | Methotrexate | 1uM | 16h | PTTG1 | 0.00 | 0.47 |
| HT29_10_GR_ChDB_0079 | Methotrexate | 1uM | 16h | STXBP1 | 0.00 | 2.39 |
| HT29_10_GR_ChDB_0079 | Methotrexate | 1uM | 16h | DUSP6 | 0.00 | 0.43 |
| HT29_10_GR_ChDB_0079 | Methotrexate | 1uM | 16h | BAD | 0.00 | 2.32 |
| HT29_10_GR_ChDB_0079 | Methotrexate | 1uM | 16h | CTNNBIP1 | 0.00 | 2.18 |
| HT29_10_GR_ChDB_0079 | Methotrexate | 1uM | 16h | SRM | 0.00 | 0.43 |
| HT29_10_GR_ChDB_0079 | Methotrexate | 1uM | 16h | STX4 | 0.00 | 2.14 |
| HT29_10_GR_ChDB_0079 | Methotrexate | 1uM | 16h | ID2 | 0.00 | 0.47 |
| HT29_10_GR_ChDB_0079 | Methotrexate | 1uM | 16h | GART | 0.00 | 0.41 |
| HT29_10_GR_ChDB_0079 | Methotrexate | 1uM | 16h | TACC3 | 0.00 | 0.33 |
| HT29_10_GR_ChDB_0079 | Methotrexate | 1uM | 16h | GOLGA2 | 0.00 | 2.10 |
| HT29_10_GR_ChDB_0079 | Methotrexate | 1uM | 16h | IFRD2 | 0.00 | 0.49 |
| HT29_10_GR_ChDB_0079 | Methotrexate | 1uM | 16h | GCDH | 0.00 | 2.09 |
| HT29_10_GR_ChDB_0079 | Methotrexate | 1uM | 16h | F2R | 0.00 | 3.68 |
| HT29_10_GR_ChDB_0079 | Methotrexate | 1uM | 16h | FKBP14 | 0.00 | 0.44 |
| HT29_10_GR_ChDB_0079 | Methotrexate | 1uM | 16h | ELOVL6 | 0.00 | 0.39 |
| HT29_10_GR_ChDB_0079 | Methotrexate | 1uM | 16h | ETV4 | 0.00 | 0.44 |
| HT29_10_GR_ChDB_0079 | Methotrexate | 1uM | 16h | LRRC59 | 0.00 | 0.49 |
| HT29_10_GR_ChDB_0079 | Methotrexate | 1uM | 16h | SLC25A14 | 0.00 | 2.23 |
| HT29_10_GR_ChDB_0079 | Methotrexate | 1uM | 16h | SLC25A15 | 0.00 | 0.49 |
| HT29_10_GR_ChDB_0079 | Methotrexate | 1uM | 16h | MRPL12 | 0.00 | 0.48 |
| HT29_10_GR_ChDB_0079 | Methotrexate | 1uM | 16h | MTERFD1 | 0.00 | 0.42 |
| HT29_10_GR_ChDB_0079 | Methotrexate | 1uM | 16h | EIF5A | 0.00 | 0.45 |
| HT29_10_GR_ChDB_0079 | Methotrexate | 1uM | 16h | CD59 | 0.00 | 3.08 |
| HT29_10_GR_ChDB_0079 | Methotrexate | 1uM | 16h | MGLL | 0.00 | 2.17 |
| HT29_10_GR_ChDB_0079 | Methotrexate | 1uM | 16h | ZNF581 | 0.00 | 2.10 |
| HT29_10_GR_ChDB_0079 | Methotrexate | 1uM | 16h | SULT1A1 | 0.00 | 2.02 |
| HT29_10_GR_ChDB_0079 | Methotrexate | 1uM | 16h | GUSB | 0.00 | 2.20 |
| HT29_10_GR_ChDB_0079 | Methotrexate | 1uM | 16h | MBNL2 | 0.00 | 2.66 |
| HT29_10_GR_ChDB_0079 | Methotrexate | 1uM | 16h | TSEN2 | 0.00 | 0.50 |
| HT29_10_GR_ChDB_0079 | Methotrexate | 1uM | 16h | INSIG1 | 0.00 | 0.40 |
| HT29_10_GR_ChDB_0079 | Methotrexate | 1uM | 16h | PRMT2 | 0.00 | 2.20 |
| HT29_10_GR_ChDB_0079 | Methotrexate | 1uM | 16h | DLGAP5 | 0.00 | 0.41 |
| HT29_10_GR_ChDB_0079 | Methotrexate | 1uM | 16h | GNB1L | 0.00 | 0.35 |
| HT29_10_GR_ChDB_0079 | Methotrexate | 1uM | 16h | DNAJC1 | 0.00 | 2.22 |
| HT29_10_GR_ChDB_0079 | Methotrexate | 1uM | 16h | SLC25A13 | 0.00 | 0.41 |
| HT29_10_GR_ChDB_0079 | Methotrexate | 1uM | 16h | PHYH | 0.00 | 2.17 |
| HT29_10_GR_ChDB_0079 | Methotrexate | 1uM | 16h | EIF4E | 0.00 | 0.43 |
| HT29_10_GR_ChDB_0079 | Methotrexate | 1uM | 16h | CTSL1 | 0.00 | 2.09 |
| HT29_10_GR_ChDB_0079 | Methotrexate | 1uM | 16h | FHL2 | 0.00 | 2.05 |
| HT29_10_GR_ChDB_0079 | Methotrexate | 1uM | 16h | DICER1 | 0.00 | 0.27 |
| HT29_10_GR_ChDB_0079 | Methotrexate | 1uM | 16h | PA2G4 | 0.00 | 0.36 |
| HT29_10_GR_ChDB_0079 | Methotrexate | 1uM | 16h | DHRS2 | 0.00 | 0.16 |
| HT29_10_GR_ChDB_0079 | Methotrexate | 1uM | 16h | CALCOCO2 | 0.00 | 2.04 |
| HT29_10_GR_ChDB_0079 | Methotrexate | 1uM | 16h | HMMR | 0.00 | 0.27 |
| HT29_10_GR_ChDB_0079 | Methotrexate | 1uM | 16h | PHRF1 | 0.00 | 0.47 |
| HT29_10_GR_ChDB_0079 | Methotrexate | 1uM | 16h | ASPM | 0.00 | 0.43 |
| HT29_10_GR_ChDB_0079 | Methotrexate | 1uM | 16h | TM9SF3 | 0.00 | 0.50 |
| HT29_10_GR_ChDB_0079 | Methotrexate | 1uM | 16h | EIF2B3 | 0.00 | 0.49 |
| HT29_10_GR_ChDB_0079 | Methotrexate | 1uM | 16h | EIF4G1 | 0.00 | 0.48 |
| HT29_10_GR_ChDB_0079 | Methotrexate | 1uM | 16h | TCF3 | 0.00 | 0.44 |
| HT29_10_GR_ChDB_0079 | Methotrexate | 1uM | 16h | DDC | 0.00 | 0.43 |
| HT29_10_GR_ChDB_0079 | Methotrexate | 1uM | 16h | CKS2 | 0.00 | 0.49 |
| HT29_10_GR_ChDB_0079 | Methotrexate | 1uM | 16h | CYP3A5 | 0.00 | 2.04 |
| HT29_10_GR_ChDB_0079 | Methotrexate | 1uM | 16h | CD55 | 0.00 | 2.27 |
| HT29_10_GR_ChDB_0079 | Methotrexate | 1uM | 16h | RFX5 | 0.00 | 2.93 |
| HT29_10_GR_ChDB_0079 | Methotrexate | 1uM | 16h | TSC22D3 | 0.00 | 2.21 |
| HT29_10_GR_ChDB_0079 | Methotrexate | 1uM | 16h | LPHN1 | 0.00 | 0.48 |
| HT29_10_GR_ChDB_0079 | Methotrexate | 1uM | 16h | DYRK2 | 0.00 | 0.48 |
| HT29_10_GR_ChDB_0079 | Methotrexate | 1uM | 16h | KCNK1 | 0.00 | 2.06 |
| HT29_10_GR_ChDB_0079 | Methotrexate | 1uM | 16h | LDLR | 0.00 | 0.42 |
| HT29_10_GR_ChDB_0079 | Methotrexate | 1uM | 16h | ALDH1B1 | 0.00 | 0.35 |
| HT29_10_GR_ChDB_0079 | Methotrexate | 1uM | 16h | IDS | 0.00 | 2.25 |
| HT29_10_GR_ChDB_0079 | Methotrexate | 1uM | 16h | CDC20 | 0.00 | 0.23 |
| HT29_10_GR_ChDB_0079 | Methotrexate | 1uM | 16h | DST | 0.00 | 2.61 |
| HT29_10_GR_ChDB_0079 | Methotrexate | 1uM | 16h | PSME3 | 0.00 | 0.41 |
| HT29_10_GR_ChDB_0079 | Methotrexate | 1uM | 16h | TPP1 | 0.00 | 2.39 |
| HT29_10_GR_ChDB_0079 | Methotrexate | 1uM | 16h | KPNB1 | 0.00 | 0.46 |
| HT29_10_GR_ChDB_0079 | Methotrexate | 1uM | 16h | SYNCRIP | 0.00 | 0.40 |
| HT29_10_GR_ChDB_0079 | Methotrexate | 1uM | 16h | BOP1 | 0.00 | 0.41 |
| HT29_10_GR_ChDB_0079 | Methotrexate | 1uM | 16h | SERBP1 | 0.00 | 0.39 |
| HT29_10_GR_ChDB_0079 | Methotrexate | 1uM | 16h | APPBP2 | 0.00 | 2.17 |
| HT29_10_GR_ChDB_0079 | Methotrexate | 1uM | 16h | GULP1 | 0.00 | 2.75 |
| HT29_10_GR_ChDB_0079 | Methotrexate | 1uM | 16h | KLHL22 | 0.00 | 2.08 |
| HT29_10_GR_ChDB_0079 | Methotrexate | 1uM | 16h | CDK19 | 0.00 | 2.74 |
| HT29_10_GR_ChDB_0079 | Methotrexate | 1uM | 16h | RAP1GAP | 0.00 | 2.09 |
| HT29_10_GR_ChDB_0079 | Methotrexate | 1uM | 16h | NF1 | 0.00 | 2.50 |
| HT29_10_GR_ChDB_0079 | Methotrexate | 1uM | 16h | KMT2A | 0.00 | 3.06 |
| HT29_10_GR_ChDB_0079 | Methotrexate | 1uM | 16h | EIF5B | 0.00 | 0.49 |
| HT29_10_GR_ChDB_0079 | Methotrexate | 1uM | 16h | ABCE1 | 0.00 | 0.47 |
| HT29_10_GR_ChDB_0079 | Methotrexate | 1uM | 16h | TSR1 | 0.00 | 0.38 |
| HT29_10_GR_ChDB_0079 | Methotrexate | 1uM | 16h | AK2 | 0.00 | 0.28 |
| HT29_10_GR_ChDB_0079 | Methotrexate | 1uM | 16h | CELF1 | 0.00 | 0.49 |
| HT29_10_GR_ChDB_0079 | Methotrexate | 1uM | 16h | EIF2S1 | 0.00 | 0.44 |
| HT29_10_GR_ChDB_0079 | Methotrexate | 1uM | 16h | EXOC7 | 0.00 | 2.29 |
| HT29_10_GR_ChDB_0079 | Methotrexate | 1uM | 16h | APLP2 | 0.00 | 2.35 |
| HT29_10_GR_ChDB_0079 | Methotrexate | 1uM | 16h | TACC1 | 0.00 | 2.01 |
| HT29_10_GR_ChDB_0079 | Methotrexate | 1uM | 16h | ALOX5 | 0.00 | 2.53 |
| HT29_10_GR_ChDB_0079 | Methotrexate | 1uM | 16h | MUC1 | 0.00 | 2.22 |
| HT29_10_GR_ChDB_0079 | Methotrexate | 1uM | 16h | TNS1 | 0.00 | 2.78 |
| HT29_10_GR_ChDB_0079 | Methotrexate | 1uM | 16h | TACSTD2 | 0.00 | 2.10 |
| HT29_10_GR_ChDB_0079 | Methotrexate | 1uM | 16h | KLHL24 | 0.00 | 2.28 |
| HT29_10_GR_ChDB_0079 | Methotrexate | 1uM | 16h | SGSM2 | 0.00 | 2.20 |
| HT29_10_GR_ChDB_0079 | Methotrexate | 1uM | 16h | ETF1 | 0.00 | 0.48 |
| HT29_10_GR_ChDB_0079 | Methotrexate | 1uM | 16h | EIF3B | 0.00 | 0.44 |
| HT29_10_GR_ChDB_0079 | Methotrexate | 1uM | 16h | SRSF1 | 0.00 | 0.36 |
| HT29_10_GR_ChDB_0079 | Methotrexate | 1uM | 16h | HNRNPC | 0.00 | 0.43 |
| HT29_10_GR_ChDB_0079 | Methotrexate | 1uM | 16h | GRSF1 | 0.00 | 0.41 |
| HT29_10_GR_ChDB_0079 | Methotrexate | 1uM | 16h | LRRFIP1 | 0.00 | 0.43 |
| HT29_10_GR_ChDB_0079 | Methotrexate | 1uM | 16h | MRPS12 | 0.00 | 0.43 |
| HT29_10_GR_ChDB_0079 | Methotrexate | 1uM | 16h | PARVA | 0.00 | 2.10 |
| HT29_10_GR_ChDB_0079 | Methotrexate | 1uM | 16h | NEBL | 0.00 | 3.55 |
| HT29_10_GR_ChDB_0079 | Methotrexate | 1uM | 16h | FAM134A | 0.00 | 2.10 |
| HT29_10_GR_ChDB_0079 | Methotrexate | 1uM | 16h | TTC39A | 0.00 | 2.57 |
| HT29_10_GR_ChDB_0079 | Methotrexate | 1uM | 16h | ATP2B4 | 0.00 | 2.71 |
| HT29_10_GR_ChDB_0079 | Methotrexate | 1uM | 16h | GSN | 0.00 | 3.32 |
| HT29_10_GR_ChDB_0079 | Methotrexate | 1uM | 16h | GOLGB1 | 0.00 | 2.01 |
| HT29_10_GR_ChDB_0079 | Methotrexate | 1uM | 16h | CEP57 | 0.00 | 2.15 |
| HT29_10_GR_ChDB_0079 | Methotrexate | 1uM | 16h | PATZ1 | 0.00 | 2.29 |
| HT29_10_GR_ChDB_0079 | Methotrexate | 1uM | 16h | AAK1 | 0.00 | 2.09 |
| HT29_10_GR_ChDB_0079 | Methotrexate | 1uM | 16h | IL1RN | 0.00 | 3.28 |
| HT29_10_GR_ChDB_0079 | Methotrexate | 1uM | 16h | LAMA3 | 0.00 | 4.19 |
| HT29_10_GR_ChDB_0079 | Methotrexate | 1uM | 16h | RAC2 | 0.00 | 3.04 |
| HT29_10_GR_ChDB_0079 | Methotrexate | 1uM | 16h | DNAJB12 | 0.00 | 2.29 |
| HT29_10_GR_ChDB_0079 | Methotrexate | 1uM | 16h | SRSF7 | 0.00 | 0.49 |
| HT29_10_GR_ChDB_0079 | Methotrexate | 1uM | 16h | SNRPA1 | 0.00 | 0.48 |
| HT29_10_GR_ChDB_0079 | Methotrexate | 1uM | 16h | WDR77 | 0.00 | 0.46 |
| HT29_10_GR_ChDB_0079 | Methotrexate | 1uM | 16h | NOP16 | 0.00 | 0.25 |
| HT29_10_GR_ChDB_0079 | Methotrexate | 1uM | 16h | DDX18 | 0.00 | 0.47 |
| HT29_10_GR_ChDB_0079 | Methotrexate | 1uM | 16h | GTPBP4 | 0.00 | 0.36 |
| HT29_10_GR_ChDB_0079 | Methotrexate | 1uM | 16h | U2AF2 | 0.00 | 0.44 |
| HT29_10_GR_ChDB_0079 | Methotrexate | 1uM | 16h | ANP32A | 0.00 | 0.45 |
| HT29_10_GR_ChDB_0079 | Methotrexate | 1uM | 16h | GEMIN4 | 0.00 | 0.45 |
| HT29_10_GR_ChDB_0079 | Methotrexate | 1uM | 16h | RBM25 | 0.00 | 0.44 |
| HT29_10_GR_ChDB_0079 | Methotrexate | 1uM | 16h | HNRNPDL | 0.00 | 0.34 |
| HT29_10_GR_ChDB_0079 | Methotrexate | 1uM | 16h | WDR12 | 0.00 | 0.41 |
| HT29_10_GR_ChDB_0079 | Methotrexate | 1uM | 16h | TFB2M | 0.00 | 0.44 |
| HT29_10_GR_ChDB_0079 | Methotrexate | 1uM | 16h | PSMD12 | 0.00 | 0.47 |
| HT29_10_GR_ChDB_0079 | Methotrexate | 1uM | 16h | H2AFX | 0.00 | 0.46 |
| HT29_10_GR_ChDB_0079 | Methotrexate | 1uM | 16h | DHX15 | 0.00 | 0.49 |
| HT29_10_GR_ChDB_0079 | Methotrexate | 1uM | 16h | MRTO4 | 0.00 | 0.47 |
| HT29_10_GR_ChDB_0079 | Methotrexate | 1uM | 16h | KPNA2 | 0.00 | 0.41 |
| HT29_10_GR_ChDB_0079 | Methotrexate | 1uM | 16h | GTF3A | 0.00 | 0.49 |
| HT29_10_GR_ChDB_0079 | Methotrexate | 1uM | 16h | PTGS1 | 0.00 | 2.04 |
| HT29_10_GR_ChDB_0079 | Methotrexate | 1uM | 16h | TRAK1 | 0.00 | 2.53 |
| HT29_10_GR_ChDB_0079 | Methotrexate | 1uM | 16h | IQGAP1 | 0.00 | 2.38 |
| HT29_10_GR_ChDB_0079 | Methotrexate | 1uM | 16h | MPPE1 | 0.00 | 2.31 |
| HT29_10_GR_ChDB_0079 | Methotrexate | 1uM | 16h | LAMC2 | 0.00 | 2.15 |
| HT29_10_GR_ChDB_0079 | Methotrexate | 1uM | 16h | SERINC2 | 0.00 | 2.13 |
| HT29_10_GR_ChDB_0079 | Methotrexate | 1uM | 16h | AMFR | 0.00 | 2.17 |
| HT29_10_GR_ChDB_0079 | Methotrexate | 1uM | 16h | ELL2 | 0.00 | 2.46 |
| HT29_10_GR_ChDB_0079 | Methotrexate | 1uM | 16h | ANK3 | 0.00 | 2.03 |
| HT29_10_GR_ChDB_0079 | Methotrexate | 1uM | 16h | AGFG1 | 0.00 | 0.45 |
| HT29_10_GR_ChDB_0079 | Methotrexate | 1uM | 16h | RNASEH1 | 0.00 | 0.45 |
| HT29_10_GR_ChDB_0079 | Methotrexate | 1uM | 16h | ARIH2 | 0.00 | 0.41 |
| HT29_10_GR_ChDB_0079 | Methotrexate | 1uM | 16h | HLA-G | 0.00 | 2.80 |
| HT29_10_GR_ChDB_0079 | Methotrexate | 1uM | 16h | ARSD | 0.00 | 2.80 |
| HT29_10_GR_ChDB_0079 | Methotrexate | 1uM | 16h | ITSN1 | 0.00 | 2.37 |
| HT29_10_GR_ChDB_0079 | Methotrexate | 1uM | 16h | DNAJC4 | 0.00 | 2.40 |
| HT29_10_GR_ChDB_0079 | Methotrexate | 1uM | 16h | SGCB | 0.00 | 2.93 |
| HT29_10_GR_ChDB_0079 | Methotrexate | 1uM | 16h | HDLBP | 0.00 | 3.80 |
| HT29_10_GR_ChDB_0079 | Methotrexate | 1uM | 16h | CAV2 | 0.00 | 2.81 |
| HT29_10_GR_ChDB_0079 | Methotrexate | 1uM | 16h | VAMP1 | 0.00 | 2.15 |
| HT29_10_GR_ChDB_0079 | Methotrexate | 1uM | 16h | MAPRE2 | 0.00 | 3.00 |
| HT29_10_GR_ChDB_0079 | Methotrexate | 1uM | 16h | SNX13 | 0.00 | 2.55 |
| HT29_10_GR_ChDB_0079 | Methotrexate | 1uM | 16h | FAM63A | 0.00 | 2.92 |
| HT29_10_GR_ChDB_0079 | Methotrexate | 1uM | 16h | HLA-DMA | 0.00 | 2.77 |
| HT29_10_GR_ChDB_0079 | Methotrexate | 1uM | 16h | KIAA0141 | 0.00 | 2.98 |
| HT29_10_GR_ChDB_0079 | Methotrexate | 1uM | 16h | CREBZF | 0.00 | 2.11 |
| HT29_10_GR_ChDB_0079 | Methotrexate | 1uM | 16h | ABCD4 | 0.00 | 2.08 |
| HT29_10_GR_ChDB_0079 | Methotrexate | 1uM | 16h | FXYD3 | 0.00 | 2.28 |
| HT29_10_GR_ChDB_0079 | Methotrexate | 1uM | 16h | ANKLE2 | 0.00 | 2.29 |
| HT29_10_GR_ChDB_0079 | Methotrexate | 1uM | 16h | SLC35E3 | 0.00 | 2.01 |
| HT29_10_GR_ChDB_0079 | Methotrexate | 1uM | 16h | MAN2A2 | 0.00 | 2.12 |
| HT29_10_GR_ChDB_0079 | Methotrexate | 1uM | 16h | NUFIP1 | 0.00 | 0.50 |
| HT29_10_GR_ChDB_0079 | Methotrexate | 1uM | 16h | DIMT1 | 0.00 | 0.46 |
| HT29_10_GR_ChDB_0079 | Methotrexate | 1uM | 16h | HAUS7 | 0.00 | 0.44 |
| HT29_10_GR_ChDB_0079 | Methotrexate | 1uM | 16h | HNRNPH3 | 0.00 | 0.39 |
| HT29_10_GR_ChDB_0079 | Methotrexate | 1uM | 16h | GAR1 | 0.00 | 0.44 |
| HT29_10_GR_ChDB_0079 | Methotrexate | 1uM | 16h | PSMD11 | 0.00 | 0.35 |
| HT29_10_GR_ChDB_0079 | Methotrexate | 1uM | 16h | FARSA | 0.00 | 0.43 |
| HT29_10_GR_ChDB_0079 | Methotrexate | 1uM | 16h | PNO1 | 0.00 | 0.39 |
| HT29_10_GR_ChDB_0079 | Methotrexate | 1uM | 16h | UBE2S | 0.00 | 0.50 |
| HT29_10_GR_ChDB_0079 | Methotrexate | 1uM | 16h | NUDC | 0.00 | 0.49 |
| HT29_10_GR_ChDB_0079 | Methotrexate | 1uM | 16h | DNPH1 | 0.00 | 2.08 |
| HT29_10_GR_ChDB_0079 | Methotrexate | 1uM | 16h | FNBP1 | 0.00 | 2.25 |
| HT29_10_GR_ChDB_0079 | Methotrexate | 1uM | 16h | VPS13C | 0.00 | 2.22 |
| HT29_10_GR_ChDB_0079 | Methotrexate | 1uM | 16h | RAB40B | 0.00 | 2.66 |
| HT29_10_GR_ChDB_0079 | Methotrexate | 1uM | 16h | AGO4 | 0.00 | 2.42 |
| HT29_10_GR_ChDB_0079 | Methotrexate | 1uM | 16h | MUT | 0.00 | 2.54 |
| HT29_10_GR_ChDB_0079 | Methotrexate | 1uM | 16h | HEBP1 | 0.00 | 2.06 |
| HT29_10_GR_ChDB_0079 | Methotrexate | 1uM | 16h | SLC19A1 | 0.00 | 0.45 |
| HT29_10_GR_ChDB_0079 | Methotrexate | 1uM | 16h | PPRC1 | 0.00 | 0.45 |
| HT29_10_GR_ChDB_0079 | Methotrexate | 1uM | 16h | HNRNPAB | 0.00 | 0.49 |
| HT29_10_GR_ChDB_0079 | Methotrexate | 1uM | 16h | GSPT1 | 0.00 | 0.33 |
| HT29_10_GR_ChDB_0079 | Methotrexate | 1uM | 16h | NOC2L | 0.00 | 0.42 |
| HT29_10_GR_ChDB_0079 | Methotrexate | 1uM | 16h | HSPH1 | 0.00 | 0.45 |
| HT29_10_GR_ChDB_0079 | Methotrexate | 1uM | 16h | RPS24 | 0.00 | 0.36 |
| HT29_10_GR_ChDB_0079 | Methotrexate | 1uM | 16h | TRMU | 0.00 | 0.41 |
| HT29_10_GR_ChDB_0079 | Methotrexate | 1uM | 16h | LSM14A | 0.00 | 0.45 |
| HT29_10_GR_ChDB_0079 | Methotrexate | 1uM | 16h | SCD | 0.00 | 0.41 |
| HT29_10_GR_ChDB_0079 | Methotrexate | 1uM | 16h | OASL | 0.00 | 2.03 |
| HT29_10_GR_ChDB_0079 | Methotrexate | 1uM | 16h | SAMD9 | 0.00 | 2.47 |
| HT29_10_GR_ChDB_0079 | Methotrexate | 1uM | 16h | CCNG2 | 0.00 | 2.98 |
| HT29_10_GR_ChDB_0079 | Methotrexate | 1uM | 16h | EPS8L1 | 0.00 | 2.26 |
| HT29_10_GR_ChDB_0079 | Methotrexate | 1uM | 16h | LAMP2 | 0.00 | 2.31 |
| HT29_10_GR_ChDB_0079 | Methotrexate | 1uM | 16h | CREBL2 | 0.00 | 2.39 |
| HT29_10_GR_ChDB_0079 | Methotrexate | 1uM | 16h | YPEL5 | 0.00 | 5.44 |
| HT29_10_GR_ChDB_0079 | Methotrexate | 1uM | 16h | OPTN | 0.00 | 4.38 |
| HT29_10_GR_ChDB_0079 | Methotrexate | 1uM | 16h | PINK1 | 0.00 | 2.41 |
| HT29_10_GR_ChDB_0079 | Methotrexate | 1uM | 16h | PLLP | 0.00 | 2.13 |
| HT29_10_GR_ChDB_0079 | Methotrexate | 1uM | 16h | CYP2C18 | 0.00 | 2.17 |
| HT29_10_GR_ChDB_0079 | Methotrexate | 1uM | 16h | TRIM31 | 0.00 | 2.06 |
| HT29_10_GR_ChDB_0079 | Methotrexate | 1uM | 16h | IL10RB | 0.00 | 2.33 |
| HT29_10_GR_ChDB_0079 | Methotrexate | 1uM | 16h | PAK6 | 0.00 | 2.17 |
| HT29_10_GR_ChDB_0079 | Methotrexate | 1uM | 16h | PDE6D | 0.00 | 2.12 |
| HT29_10_GR_ChDB_0079 | Methotrexate | 1uM | 16h | CCHCR1 | 0.00 | 2.01 |
| HT29_10_GR_ChDB_0079 | Methotrexate | 1uM | 16h | RGS2 | 0.00 | 2.08 |
| HT29_10_GR_ChDB_0079 | Methotrexate | 1uM | 16h | ABCG1 | 0.00 | 2.05 |
| HT29_10_GR_ChDB_0079 | Methotrexate | 1uM | 16h | ATP9A | 0.00 | 2.18 |
| HT29_10_GR_ChDB_0079 | Methotrexate | 1uM | 16h | BBIP1 | 0.00 | 2.34 |
| HT29_10_GR_ChDB_0079 | Methotrexate | 1uM | 16h | EPB41L5 | 0.00 | 2.10 |
| HT29_10_GR_ChDB_0079 | Methotrexate | 1uM | 16h | SLC29A3 | 0.00 | 2.00 |
| HT29_10_GR_ChDB_0079 | Methotrexate | 1uM | 16h | CYTH2 | 0.00 | 2.80 |
| HT29_10_GR_ChDB_0079 | Methotrexate | 1uM | 16h | ZNF277 | 0.00 | 2.42 |
| HT29_10_GR_ChDB_0079 | Methotrexate | 1uM | 16h | PRSS16 | 0.00 | 2.22 |
| HT29_10_GR_ChDB_0079 | Methotrexate | 1uM | 16h | CEACAM6 | 0.00 | 2.17 |
| HT29_10_GR_ChDB_0079 | Methotrexate | 1uM | 16h | CLTB | 0.00 | 2.12 |
| HT29_10_GR_ChDB_0079 | Methotrexate | 1uM | 16h | RAB27B | 0.00 | 2.81 |
| HT29_10_GR_ChDB_0079 | Methotrexate | 1uM | 16h | C1orf63 | 0.00 | 2.23 |
| HT29_10_GR_ChDB_0079 | Methotrexate | 1uM | 16h | DNASE2 | 0.00 | 2.08 |
| HT29_10_GR_ChDB_0079 | Methotrexate | 1uM | 16h | UBE2H | 0.00 | 2.11 |
| HT29_10_GR_ChDB_0079 | Methotrexate | 1uM | 16h | MMP28 | 0.00 | 2.50 |
| HT29_10_GR_ChDB_0079 | Methotrexate | 1uM | 16h | COL5A2 | 0.00 | 2.30 |
| HT29_10_GR_ChDB_0079 | Methotrexate | 1uM | 16h | HDAC5 | 0.00 | 2.19 |
| HT29_10_GR_ChDB_0079 | Methotrexate | 1uM | 16h | AGTPBP1 | 0.00 | 2.16 |
| HT29_10_GR_ChDB_0079 | Methotrexate | 1uM | 16h | B9D1 | 0.00 | 2.05 |
| HT29_10_GR_ChDB_0079 | Methotrexate | 1uM | 16h | SOS1 | 0.00 | 2.98 |
| HT29_10_GR_ChDB_0079 | Methotrexate | 1uM | 16h | DENND3 | 0.00 | 2.51 |
| HT29_10_GR_ChDB_0079 | Methotrexate | 1uM | 16h | TMLHE | 0.00 | 2.21 |
| HT29_10_GR_ChDB_0079 | Methotrexate | 1uM | 16h | SLC12A6 | 0.00 | 3.63 |
| HT29_10_GR_ChDB_0079 | Methotrexate | 1uM | 16h | CCDC28A | 0.00 | 3.61 |
| HT29_10_GR_ChDB_0079 | Methotrexate | 1uM | 16h | ATF5 | 0.00 | 2.07 |
| HT29_10_GR_ChDB_0079 | Methotrexate | 1uM | 16h | MYH10 | 0.00 | 2.58 |
| HT29_10_GR_ChDB_0079 | Methotrexate | 1uM | 16h | RHOD | 0.00 | 3.06 |
| HT29_10_GR_ChDB_0079 | Methotrexate | 1uM | 16h | LMO7 | 0.00 | 2.88 |
| HT29_10_GR_ChDB_0079 | Methotrexate | 1uM | 16h | THSD4 | 0.00 | 2.34 |
| HT29_10_GR_ChDB_0079 | Methotrexate | 1uM | 16h | SMIM14 | 0.00 | 2.27 |
| HT29_10_GR_ChDB_0079 | Methotrexate | 1uM | 16h | NEDD9 | 0.00 | 3.44 |
| HT29_10_GR_ChDB_0079 | Methotrexate | 1uM | 16h | SDPR | 0.00 | 3.36 |
| HT29_10_GR_ChDB_0079 | Methotrexate | 1uM | 16h | RRP15 | 0.00 | 0.36 |
| HT29_10_GR_ChDB_0079 | Methotrexate | 1uM | 16h | DDX52 | 0.00 | 0.40 |
| HT29_10_GR_ChDB_0079 | Methotrexate | 1uM | 16h | DIEXF | 0.00 | 0.49 |
| HT29_10_GR_ChDB_0079 | Methotrexate | 1uM | 16h | LSG1 | 0.00 | 0.41 |
| HT29_10_GR_ChDB_0079 | Methotrexate | 1uM | 16h | DHX9 | 0.00 | 0.39 |
| HT29_10_GR_ChDB_0079 | Methotrexate | 1uM | 16h | MAK16 | 0.00 | 0.40 |
| HT29_10_GR_ChDB_0079 | Methotrexate | 1uM | 16h | GCN1L1 | 0.00 | 0.44 |
| HT29_10_GR_ChDB_0079 | Methotrexate | 1uM | 16h | WDR43 | 0.00 | 0.35 |
| HT29_10_GR_ChDB_0079 | Methotrexate | 1uM | 16h | MRPS17 | 0.00 | 0.49 |
| HT29_10_GR_ChDB_0079 | Methotrexate | 1uM | 16h | LARP4 | 0.00 | 0.41 |
| HT29_10_GR_ChDB_0079 | Methotrexate | 1uM | 16h | SEC23IP | 0.00 | 0.49 |
| HT29_10_GR_ChDB_0079 | Methotrexate | 1uM | 16h | CD3EAP | 0.00 | 0.35 |
| HT29_10_GR_ChDB_0079 | Methotrexate | 1uM | 16h | BRIX1 | 0.00 | 0.43 |
| HT29_10_GR_ChDB_0079 | Methotrexate | 1uM | 16h | SRSF6 | 0.00 | 0.35 |
| HT29_10_GR_ChDB_0079 | Methotrexate | 1uM | 16h | PRMT5 | 0.00 | 0.46 |
| HT29_10_GR_ChDB_0079 | Methotrexate | 1uM | 16h | RBM3 | 0.00 | 0.39 |
| HT29_10_GR_ChDB_0079 | Methotrexate | 1uM | 16h | POLR3G | 0.00 | 0.29 |
| HT29_10_GR_ChDB_0079 | Methotrexate | 1uM | 16h | CDCA3 | 0.00 | 0.42 |
| HT29_10_GR_ChDB_0079 | Methotrexate | 1uM | 16h | TCEB2 | 0.00 | 0.41 |
| HT29_10_GR_ChDB_0079 | Methotrexate | 1uM | 16h | MRP63 | 0.00 | 0.33 |
| HT29_10_GR_ChDB_0079 | Methotrexate | 1uM | 16h | CENPF | 0.00 | 0.49 |
| HT29_10_GR_ChDB_0079 | Methotrexate | 1uM | 16h | MINA | 0.00 | 0.44 |
| HT29_10_GR_ChDB_0079 | Methotrexate | 1uM | 16h | NOL12 | 0.00 | 0.46 |
| HT29_10_GR_ChDB_0079 | Methotrexate | 1uM | 16h | HNRNPA2B1 | 0.00 | 0.41 |
| HT29_10_GR_ChDB_0079 | Methotrexate | 1uM | 16h | STIP1 | 0.00 | 0.40 |
| HT29_10_GR_ChDB_0079 | Methotrexate | 1uM | 16h | HLA-E | 0.00 | 3.02 |
| HT29_10_GR_ChDB_0079 | Methotrexate | 1uM | 16h | HLA-C | 0.00 | 2.00 |
| HT29_10_GR_ChDB_0079 | Methotrexate | 1uM | 16h | LAMP1 | 0.00 | 2.06 |
| HT29_10_GR_ChDB_0079 | Methotrexate | 1uM | 16h | DNAJC22 | 0.00 | 2.93 |
| HT29_10_GR_ChDB_0079 | Methotrexate | 1uM | 16h | KIAA0195 | 0.00 | 2.01 |
| HT29_10_GR_ChDB_0079 | Methotrexate | 1uM | 16h | TFAP2C | 0.00 | 2.53 |
| HT29_10_GR_ChDB_0079 | Methotrexate | 1uM | 16h | ERAP2 | 0.00 | 3.22 |
| HT29_10_GR_ChDB_0079 | Methotrexate | 1uM | 16h | SEPT8 | 0.00 | 2.32 |
| HT29_10_GR_ChDB_0079 | Methotrexate | 1uM | 16h | SYTL2 | 0.00 | 4.23 |
| HT29_10_GR_ChDB_0079 | Methotrexate | 1uM | 16h | LPP | 0.00 | 2.06 |
| HT29_10_GR_ChDB_0079 | Methotrexate | 1uM | 16h | TSPAN1 | 0.00 | 2.27 |
| HT29_10_GR_ChDB_0079 | Methotrexate | 1uM | 16h | HPSE | 0.00 | 3.03 |
| HT29_10_GR_ChDB_0079 | Methotrexate | 1uM | 16h | CUL4B | 0.00 | 2.16 |
| HT29_10_GR_ChDB_0079 | Methotrexate | 1uM | 16h | GNS | 0.00 | 2.24 |
| HT29_10_GR_ChDB_0079 | Methotrexate | 1uM | 16h | MMP24-AS1 | 0.00 | 2.25 |
| HT29_10_GR_ChDB_0079 | Methotrexate | 1uM | 16h | DGKQ | 0.00 | 2.14 |
| HT29_10_GR_ChDB_0079 | Methotrexate | 1uM | 16h | EFNA2 | 0.00 | 2.42 |
| HT29_10_GR_ChDB_0079 | Methotrexate | 1uM | 16h | VGLL1 | 0.00 | 4.21 |
| HT29_10_GR_ChDB_0079 | Methotrexate | 1uM | 16h | STAG3L4 | 0.00 | 2.20 |
| HT29_10_GR_ChDB_0079 | Methotrexate | 1uM | 16h | AIM1L | 0.00 | 2.22 |
| HT29_10_GR_ChDB_0079 | Methotrexate | 1uM | 16h | ARL14 | 0.00 | 4.25 |
| HT29_10_GR_ChDB_0079 | Methotrexate | 1uM | 16h | GDF15 | 0.00 | 3.46 |
| HT29_10_GR_ChDB_0079 | Methotrexate | 1uM | 16h | C9orf116 | 0.00 | 2.20 |
| HT29_10_GR_ChDB_0079 | Methotrexate | 1uM | 16h | CRAT | 0.00 | 3.27 |
| HT29_10_GR_ChDB_0079 | Methotrexate | 1uM | 16h | WDR5B | 0.00 | 2.01 |
| HT29_10_GR_ChDB_0079 | Methotrexate | 1uM | 16h | FOLR1 | 0.00 | 3.62 |
| HT29_10_GR_ChDB_0079 | Methotrexate | 1uM | 16h | ZNF451 | 0.00 | 2.09 |
| HT29_10_GR_ChDB_0079 | Methotrexate | 1uM | 16h | RAB33B | 0.00 | 2.66 |
| HT29_10_GR_ChDB_0079 | Methotrexate | 1uM | 16h | C4BPB | 0.00 | 2.48 |
| HT29_10_GR_ChDB_0079 | Methotrexate | 1uM | 16h | CHMP1B | 0.00 | 2.44 |
| HT29_10_GR_ChDB_0079 | Methotrexate | 1uM | 16h | INTS7 | 0.00 | 2.01 |
| HT29_10_GR_ChDB_0079 | Methotrexate | 1uM | 16h | CLU | 0.00 | 2.43 |
| HT29_10_GR_ChDB_0079 | Methotrexate | 1uM | 16h | STYK1 | 0.00 | 3.42 |
| HT29_10_GR_ChDB_0079 | Methotrexate | 1uM | 16h | ENPP4 | 0.00 | 2.59 |
| HT29_10_GR_ChDB_0079 | Methotrexate | 1uM | 16h | C1orf116 | 0.00 | 2.79 |
| HT29_10_GR_ChDB_0079 | Methotrexate | 1uM | 16h | METTL7A | 0.00 | 2.25 |
| HT29_10_GR_ChDB_0079 | Methotrexate | 1uM | 16h | CCNE2 | 0.00 | 2.85 |
| HT29_10_GR_ChDB_0079 | Methotrexate | 1uM | 16h | APOBEC3B | 0.00 | 5.34 |
| HT29_10_GR_ChDB_0079 | Methotrexate | 1uM | 16h | RBMS2 | 0.00 | 2.90 |
| HT29_10_GR_ChDB_0079 | Methotrexate | 1uM | 16h | CSGALNACT2 | 0.00 | 2.76 |
| HT29_10_GR_ChDB_0079 | Methotrexate | 1uM | 16h | DAAM1 | 0.00 | 2.15 |
| HT29_10_GR_ChDB_0079 | Methotrexate | 1uM | 16h | HNRNPU | 0.00 | 0.45 |
| HT29_10_GR_ChDB_0079 | Methotrexate | 1uM | 16h | TCOF1 | 0.00 | 0.43 |
| HT29_10_GR_ChDB_0079 | Methotrexate | 1uM | 16h | HNRNPA3 | 0.00 | 0.42 |
| HT29_10_GR_ChDB_0079 | Methotrexate | 1uM | 16h | WDR3 | 0.00 | 0.43 |
| HT29_10_GR_ChDB_0079 | Methotrexate | 1uM | 16h | RRP9 | 0.00 | 0.43 |
| HT29_10_GR_ChDB_0079 | Methotrexate | 1uM | 16h | XPOT | 0.00 | 0.29 |
| HT29_10_GR_ChDB_0079 | Methotrexate | 1uM | 16h | TRIM14 | 0.00 | 0.49 |
| HT29_10_GR_ChDB_0079 | Methotrexate | 1uM | 16h | EIF4E2 | 0.00 | 0.48 |
| HT29_10_GR_ChDB_0079 | Methotrexate | 1uM | 16h | CDKN3 | 0.00 | 0.47 |
| HT29_10_GR_ChDB_0079 | Methotrexate | 1uM | 16h | NAA15 | 0.00 | 0.44 |
| HT29_10_GR_ChDB_0079 | Methotrexate | 1uM | 16h | DNAJA1 | 0.00 | 0.42 |
| HT29_10_GR_ChDB_0079 | Methotrexate | 1uM | 16h | AP1S1 | 0.00 | 0.50 |
| HT29_10_GR_ChDB_0079 | Methotrexate | 1uM | 16h | CHORDC1 | 0.00 | 0.40 |
| HT29_10_GR_ChDB_0079 | Methotrexate | 1uM | 16h | BMS1 | 0.00 | 0.48 |
| HT29_10_GR_ChDB_0079 | Methotrexate | 1uM | 16h | FGD6 | 0.00 | 2.39 |
| HT29_10_GR_ChDB_0079 | Methotrexate | 1uM | 16h | POLD4 | 0.00 | 2.22 |
| HT29_10_GR_ChDB_0079 | Methotrexate | 1uM | 16h | RNF19B | 0.00 | 3.00 |
| HT29_10_GR_ChDB_0079 | Methotrexate | 1uM | 16h | AHNAK2 | 0.00 | 3.17 |
| HT29_10_GR_ChDB_0079 | Methotrexate | 1uM | 16h | CTBS | 0.00 | 2.27 |
| HT29_10_GR_ChDB_0079 | Methotrexate | 1uM | 16h | DPP4 | 0.00 | 2.55 |
| HT29_10_GR_ChDB_0079 | Methotrexate | 1uM | 16h | KRT7 | 0.00 | 3.52 |
| HT29_10_GR_ChDB_0079 | Methotrexate | 1uM | 16h | ZNF33B | 0.00 | 3.57 |
| HT29_10_GR_ChDB_0079 | Methotrexate | 1uM | 16h | PPP1R15A | 0.00 | 2.13 |
| HT29_10_GR_ChDB_0079 | Methotrexate | 1uM | 16h | TBC1D9 | 0.00 | 2.09 |
| HT29_10_GR_ChDB_0079 | Methotrexate | 1uM | 16h | C1RL | 0.00 | 2.35 |
| HT29_10_GR_ChDB_0079 | Methotrexate | 1uM | 16h | CARD8 | 0.00 | 2.34 |
| HT29_10_GR_ChDB_0079 | Methotrexate | 1uM | 16h | PNRC1 | 0.00 | 3.67 |
| HT29_10_GR_ChDB_0079 | Methotrexate | 1uM | 16h | OCLN | 0.00 | 2.56 |
| HT29_10_GR_ChDB_0079 | Methotrexate | 1uM | 16h | CNNM4 | 0.00 | 2.24 |
| HT29_10_GR_ChDB_0079 | Methotrexate | 1uM | 16h | GRAMD1C | 0.00 | 2.51 |
| HT29_10_GR_ChDB_0079 | Methotrexate | 1uM | 16h | ATP6V1A | 0.00 | 2.22 |
| HT29_10_GR_ChDB_0079 | Methotrexate | 1uM | 16h | INPP5F | 0.00 | 2.42 |
| HT29_10_GR_ChDB_0079 | Methotrexate | 1uM | 16h | GAD1 | 0.00 | 2.03 |
| HT29_10_GR_ChDB_0079 | Methotrexate | 1uM | 16h | CYTH3 | 0.00 | 2.41 |
| HT29_10_GR_ChDB_0079 | Methotrexate | 1uM | 16h | SCEL | 0.00 | 4.79 |
| HT29_10_GR_ChDB_0079 | Methotrexate | 1uM | 16h | NR1D2 | 0.00 | 2.36 |
| HT29_10_GR_ChDB_0079 | Methotrexate | 1uM | 16h | TNNC1 | 0.00 | 3.43 |
| HT29_10_GR_ChDB_0079 | Methotrexate | 1uM | 16h | NBPF1 | 0.00 | 4.30 |
| HT29_10_GR_ChDB_0079 | Methotrexate | 1uM | 16h | CLIC3 | 0.00 | 4.94 |
| HT29_10_GR_ChDB_0079 | Methotrexate | 1uM | 16h | SNX16 | 0.00 | 2.43 |
| HT29_10_GR_ChDB_0079 | Methotrexate | 1uM | 16h | FIG4 | 0.00 | 2.88 |
| HT29_10_GR_ChDB_0079 | Methotrexate | 1uM | 16h | FAM188A | 0.00 | 2.14 |
| HT29_10_GR_ChDB_0079 | Methotrexate | 1uM | 16h | ERMAP | 0.00 | 2.56 |
| HT29_10_GR_ChDB_0079 | Methotrexate | 1uM | 16h | PALLD | 0.00 | 2.79 |
| HT29_10_GR_ChDB_0079 | Methotrexate | 1uM | 16h | SERP1 | 0.00 | 2.44 |
| HT29_10_GR_ChDB_0079 | Methotrexate | 1uM | 16h | CNPY4 | 0.00 | 2.07 |
| HT29_10_GR_ChDB_0079 | Methotrexate | 1uM | 16h | TUFT1 | 0.00 | 2.49 |
| HT29_10_GR_ChDB_0079 | Methotrexate | 1uM | 16h | ITGB6 | 0.00 | 3.81 |
| HT29_10_GR_ChDB_0079 | Methotrexate | 1uM | 16h | LAMB3 | 0.00 | 4.32 |
| HT29_10_GR_ChDB_0079 | Methotrexate | 1uM | 16h | ULBP2 | 0.00 | 4.49 |
| HT29_10_GR_ChDB_0079 | Methotrexate | 1uM | 16h | PARD6A | 0.00 | 3.24 |
| HT29_10_GR_ChDB_0079 | Methotrexate | 1uM | 16h | PPP1R13L | 0.00 | 2.13 |
| HT29_10_GR_ChDB_0079 | Methotrexate | 1uM | 16h | CDADC1 | 0.00 | 2.58 |
| HT29_10_GR_ChDB_0079 | Methotrexate | 1uM | 16h | CHKB | 0.00 | 2.02 |
| HT29_10_GR_ChDB_0079 | Methotrexate | 1uM | 16h | NUPR1 | 0.00 | 3.88 |
| HT29_10_GR_ChDB_0079 | Methotrexate | 1uM | 16h | VSIG10 | 0.00 | 3.27 |
| HT29_10_GR_ChDB_0079 | Methotrexate | 1uM | 16h | CSRNP2 | 0.00 | 2.89 |
| HT29_10_GR_ChDB_0079 | Methotrexate | 1uM | 16h | BCKDHB | 0.00 | 2.47 |
| HT29_10_GR_ChDB_0079 | Methotrexate | 1uM | 16h | HYI | 0.00 | 2.27 |
| HT29_10_GR_ChDB_0079 | Methotrexate | 1uM | 16h | SEMA7A | 0.00 | 2.14 |
| HT29_10_GR_ChDB_0079 | Methotrexate | 1uM | 16h | SLC7A7 | 0.00 | 2.83 |
| HT29_10_GR_ChDB_0079 | Methotrexate | 1uM | 16h | AHSA1 | 0.00 | 0.45 |
| HT29_10_GR_ChDB_0079 | Methotrexate | 1uM | 16h | HEATR3 | 0.00 | 0.45 |
| HT29_10_GR_ChDB_0079 | Methotrexate | 1uM | 16h | C10orf2 | 0.00 | 0.26 |
| HT29_10_GR_ChDB_0079 | Methotrexate | 1uM | 16h | NIP7 | 0.00 | 0.40 |
| HT29_10_GR_ChDB_0079 | Methotrexate | 1uM | 16h | SLC7A5 | 0.00 | 0.49 |
| HT29_10_GR_ChDB_0079 | Methotrexate | 1uM | 16h | KNOP1 | 0.00 | 0.42 |
| HT29_10_GR_ChDB_0079 | Methotrexate | 1uM | 16h | FTSJ3 | 0.00 | 0.37 |
| HT29_10_GR_ChDB_0079 | Methotrexate | 1uM | 16h | EXOSC5 | 0.00 | 0.44 |
| HT29_10_GR_ChDB_0079 | Methotrexate | 1uM | 16h | DDX46 | 0.00 | 0.46 |
| HT29_10_GR_ChDB_0079 | Methotrexate | 1uM | 16h | SMCR7L | 0.00 | 0.49 |
| HT29_10_GR_ChDB_0079 | Methotrexate | 1uM | 16h | PPP2R1B | 0.00 | 0.48 |
| HT29_10_GR_ChDB_0079 | Methotrexate | 1uM | 16h | SFXN1 | 0.00 | 0.36 |
| HT29_10_GR_ChDB_0079 | Methotrexate | 1uM | 16h | FAM98A | 0.00 | 0.50 |
| HT29_10_GR_ChDB_0079 | Methotrexate | 1uM | 16h | TIA1 | 0.00 | 0.42 |
| HT29_10_GR_ChDB_0079 | Methotrexate | 1uM | 16h | HCCS | 0.00 | 0.47 |
| HT29_10_GR_ChDB_0079 | Methotrexate | 1uM | 16h | LAS1L | 0.00 | 0.44 |
| HT29_10_GR_ChDB_0079 | Methotrexate | 1uM | 16h | YARS2 | 0.00 | 0.42 |
| HT29_10_GR_ChDB_0079 | Methotrexate | 1uM | 16h | ESF1 | 0.00 | 0.49 |
| HT29_10_GR_ChDB_0079 | Methotrexate | 1uM | 16h | ARPC5L | 0.00 | 0.49 |
| HT29_10_GR_ChDB_0079 | Methotrexate | 1uM | 16h | TUSC2 | 0.00 | 0.44 |
| HT29_10_GR_ChDB_0079 | Methotrexate | 1uM | 16h | DFFA | 0.00 | 0.47 |
| HT29_10_GR_ChDB_0079 | Methotrexate | 1uM | 16h | SDCCAG3 | 0.00 | 0.42 |
| HT29_10_GR_ChDB_0079 | Methotrexate | 1uM | 16h | IDI1 | 0.00 | 0.46 |
| HT29_10_GR_ChDB_0079 | Methotrexate | 1uM | 16h | PAK1IP1 | 0.00 | 0.20 |
| HT29_10_GR_ChDB_0079 | Methotrexate | 1uM | 16h | DPEP1 | 0.00 | 2.34 |
| HT29_10_GR_ChDB_0079 | Methotrexate | 1uM | 16h | SYT17 | 0.00 | 2.26 |
| HT29_10_GR_ChDB_0079 | Methotrexate | 1uM | 16h | CES2 | 0.00 | 2.46 |
| HT29_10_GR_ChDB_0079 | Methotrexate | 1uM | 16h | ITFG1 | 0.00 | 3.15 |
| HT29_10_GR_ChDB_0079 | Methotrexate | 1uM | 16h | SNX24 | 0.00 | 2.04 |
| HT29_10_GR_ChDB_0079 | Methotrexate | 1uM | 16h | HECA | 0.00 | 2.11 |
| HT29_10_GR_ChDB_0079 | Methotrexate | 1uM | 16h | AQP3 | 0.00 | 3.32 |
| HT29_10_GR_ChDB_0079 | Methotrexate | 1uM | 16h | PBLD | 0.00 | 2.94 |
| HT29_10_GR_ChDB_0079 | Methotrexate | 1uM | 16h | ORAI3 | 0.00 | 2.86 |
| HT29_10_GR_ChDB_0079 | Methotrexate | 1uM | 16h | AADAC | 0.00 | 2.63 |
| HT29_10_GR_ChDB_0079 | Methotrexate | 1uM | 16h | PTPRH | 0.00 | 2.77 |
| HT29_10_GR_ChDB_0079 | Methotrexate | 1uM | 16h | ANXA11 | 0.00 | 2.25 |
| HT29_10_GR_ChDB_0079 | Methotrexate | 1uM | 16h | CAV1 | 0.00 | 5.11 |
| HT29_10_GR_ChDB_0079 | Methotrexate | 1uM | 16h | SPC24 | 0.00 | 3.22 |
| HT29_10_GR_ChDB_0079 | Methotrexate | 1uM | 16h | STEAP4 | 0.00 | 2.31 |
| HT29_10_GR_ChDB_0079 | Methotrexate | 1uM | 16h | ZNF185 | 0.00 | 2.96 |
| HT29_10_GR_ChDB_0079 | Methotrexate | 1uM | 16h | TPM4 | 0.00 | 2.01 |
| HT29_10_GR_ChDB_0079 | Methotrexate | 1uM | 16h | SELENBP1 | 0.00 | 2.00 |
| HT29_10_GR_ChDB_0079 | Methotrexate | 1uM | 16h | TTC33 | 0.00 | 2.12 |
| HT29_10_GR_ChDB_0079 | Methotrexate | 1uM | 16h | UCP2 | 0.00 | 3.05 |
| HT29_10_GR_ChDB_0079 | Methotrexate | 1uM | 16h | AMIGO2 | 0.00 | 2.22 |
| HT29_10_GR_ChDB_0079 | Methotrexate | 1uM | 16h | FGD2 | 0.00 | 2.37 |
| HT29_10_GR_ChDB_0079 | Methotrexate | 1uM | 16h | FAM111A | 0.00 | 2.35 |
| HT29_10_GR_ChDB_0079 | Methotrexate | 1uM | 16h | ZNF189 | 0.00 | 2.10 |
| HT29_10_GR_ChDB_0079 | Methotrexate | 1uM | 16h | SIDT2 | 0.00 | 2.13 |
| HT29_10_GR_ChDB_0079 | Methotrexate | 1uM | 16h | SCPEP1 | 0.00 | 3.88 |
| HT29_10_GR_ChDB_0079 | Methotrexate | 1uM | 16h | ZNF226 | 0.00 | 2.16 |
| HT29_10_GR_ChDB_0079 | Methotrexate | 1uM | 16h | ELF5 | 0.00 | 2.67 |
| HT29_10_GR_ChDB_0079 | Methotrexate | 1uM | 16h | KRBOX4 | 0.00 | 2.68 |
| HT29_10_GR_ChDB_0079 | Methotrexate | 1uM | 16h | PUS1 | 0.00 | 0.26 |
| HT29_10_GR_ChDB_0079 | Methotrexate | 1uM | 16h | AGPAT5 | 0.00 | 0.47 |
| HT29_10_GR_ChDB_0079 | Methotrexate | 1uM | 16h | ATAD3A | 0.00 | 0.40 |
| HT29_10_GR_ChDB_0079 | Methotrexate | 1uM | 16h | NRF1 | 0.00 | 0.50 |
| HT29_10_GR_ChDB_0079 | Methotrexate | 1uM | 16h | PEX5 | 0.00 | 0.49 |
| HT29_10_GR_ChDB_0079 | Methotrexate | 1uM | 16h | SLC27A5 | 0.00 | 0.49 |
| HT29_10_GR_ChDB_0079 | Methotrexate | 1uM | 16h | PTTG3P | 0.00 | 0.30 |
| HT29_10_GR_ChDB_0079 | Methotrexate | 1uM | 16h | DEPDC1 | 0.00 | 0.42 |
| HT29_10_GR_ChDB_0079 | Methotrexate | 1uM | 16h | SRSF11 | 0.00 | 0.38 |
| HT29_10_GR_ChDB_0079 | Methotrexate | 1uM | 16h | NUPL1 | 0.00 | 0.45 |
| HT29_10_GR_ChDB_0079 | Methotrexate | 1uM | 16h | H1FX | 0.00 | 0.42 |
| HT29_10_GR_ChDB_0079 | Methotrexate | 1uM | 16h | SLC25A40 | 0.00 | 2.10 |
| HT29_10_GR_ChDB_0079 | Methotrexate | 1uM | 16h | CYR61 | 0.00 | 3.72 |
| HT29_10_GR_ChDB_0079 | Methotrexate | 1uM | 16h | KAT2B | 0.00 | 2.48 |
| HT29_10_GR_ChDB_0079 | Methotrexate | 1uM | 16h | OAS3 | 0.00 | 2.33 |
| HT29_10_GR_ChDB_0079 | Methotrexate | 1uM | 16h | PDZK1IP1 | 0.00 | 2.62 |
| HT29_10_GR_ChDB_0079 | Methotrexate | 1uM | 16h | ALDH1A3 | 0.00 | 5.26 |
| HT29_10_GR_ChDB_0079 | Methotrexate | 1uM | 16h | CAMK2N1 | 0.00 | 2.59 |
| HT29_10_GR_ChDB_0079 | Methotrexate | 1uM | 16h | MTMR11 | 0.00 | 2.24 |
| HT29_10_GR_ChDB_0079 | Methotrexate | 1uM | 16h | KIAA0319L | 0.00 | 2.09 |
| HT29_10_GR_ChDB_0079 | Methotrexate | 1uM | 16h | MLF1IP | 0.00 | 2.82 |
| HT29_10_GR_ChDB_0079 | Methotrexate | 1uM | 16h | HCFC1R1 | 0.00 | 2.94 |
| HT29_10_GR_ChDB_0079 | Methotrexate | 1uM | 16h | OBFC1 | 0.00 | 2.18 |
| HT29_10_GR_ChDB_0079 | Methotrexate | 1uM | 16h | TTC17 | 0.00 | 2.23 |
| HT29_10_GR_ChDB_0079 | Methotrexate | 1uM | 16h | SPATS2 | 0.00 | 2.08 |
| HT29_10_GR_ChDB_0079 | Methotrexate | 1uM | 16h | BBS1 | 0.00 | 2.08 |
| HT29_10_GR_ChDB_0079 | Methotrexate | 1uM | 16h | ZDHHC4 | 0.00 | 3.40 |
| HT29_10_GR_ChDB_0079 | Methotrexate | 1uM | 16h | LMCD1 | 0.00 | 2.25 |
| HT29_10_GR_ChDB_0079 | Methotrexate | 1uM | 16h | C11orf68 | 0.00 | 2.38 |
| HT29_10_GR_ChDB_0079 | Methotrexate | 1uM | 16h | CD82 | 0.00 | 2.30 |
| HT29_10_GR_ChDB_0079 | Methotrexate | 1uM | 16h | DHTKD1 | 0.00 | 4.26 |
| HT29_10_GR_ChDB_0079 | Methotrexate | 1uM | 16h | IGFLR1 | 0.00 | 2.14 |
| HT29_10_GR_ChDB_0079 | Methotrexate | 1uM | 16h | SLPI | 0.00 | 3.09 |
| HT29_10_GR_ChDB_0079 | Methotrexate | 1uM | 16h | GLT8D1 | 0.00 | 2.44 |
| HT29_10_GR_ChDB_0079 | Methotrexate | 1uM | 16h | TCEAL1 | 0.00 | 2.20 |
| HT29_10_GR_ChDB_0079 | Methotrexate | 1uM | 16h | SULT2B1 | 0.00 | 2.38 |
| HT29_10_GR_ChDB_0079 | Methotrexate | 1uM | 16h | MED23 | 0.00 | 2.06 |
| HT29_10_GR_ChDB_0079 | Methotrexate | 1uM | 16h | PRSS8 | 0.00 | 2.03 |
| HT29_10_GR_ChDB_0079 | Methotrexate | 1uM | 16h | ACSF2 | 0.00 | 2.04 |
| HT29_10_GR_ChDB_0079 | Methotrexate | 1uM | 16h | CDKN2B | 0.00 | 3.27 |
| HT29_10_GR_ChDB_0079 | Methotrexate | 1uM | 16h | WDR45 | 0.00 | 3.24 |
| HT29_10_GR_ChDB_0079 | Methotrexate | 1uM | 16h | WDR13 | 0.00 | 2.79 |
| HT29_10_GR_ChDB_0079 | Methotrexate | 1uM | 16h | C1orf50 | 0.00 | 2.22 |
| HT29_10_GR_ChDB_0079 | Methotrexate | 1uM | 16h | PON3 | 0.00 | 3.67 |
| HT29_10_GR_ChDB_0079 | Methotrexate | 1uM | 16h | MKNK1 | 0.00 | 2.05 |
| HT29_10_GR_ChDB_0079 | Methotrexate | 1uM | 16h | ABCA7 | 0.00 | 2.32 |
| HT29_10_GR_ChDB_0079 | Methotrexate | 1uM | 16h | SPINK1 | 0.00 | 3.51 |
| HT29_10_GR_ChDB_0079 | Methotrexate | 1uM | 16h | SPTLC1 | 0.00 | 2.05 |
| HT29_10_GR_ChDB_0079 | Methotrexate | 1uM | 16h | REV3L | 0.00 | 2.06 |
| HT29_10_GR_ChDB_0079 | Methotrexate | 1uM | 16h | BCKDHA | 0.00 | 2.57 |
| HT29_10_GR_ChDB_0079 | Methotrexate | 1uM | 16h | PARP8 | 0.00 | 2.01 |
| HT29_10_GR_ChDB_0079 | Methotrexate | 1uM | 16h | GPR126 | 0.00 | 3.41 |
| HT29_10_GR_ChDB_0079 | Methotrexate | 1uM | 16h | TSPYL4 | 0.00 | 2.85 |
| HT29_10_GR_ChDB_0079 | Methotrexate | 1uM | 16h | SLC7A6 | 0.00 | 0.36 |
| HT29_10_GR_ChDB_0079 | Methotrexate | 1uM | 16h | URB2 | 0.00 | 0.45 |
| HT29_10_GR_ChDB_0079 | Methotrexate | 1uM | 16h | DBF4 | 0.00 | 0.47 |
| HT29_10_GR_ChDB_0079 | Methotrexate | 1uM | 16h | TIMM8A | 0.00 | 0.43 |
| HT29_10_GR_ChDB_0079 | Methotrexate | 1uM | 16h | PRPF38B | 0.00 | 0.44 |
| HT29_10_GR_ChDB_0079 | Methotrexate | 1uM | 16h | FASTKD1 | 0.00 | 0.46 |
| HT29_10_GR_ChDB_0079 | Methotrexate | 1uM | 16h | GNL2 | 0.00 | 0.47 |
| HT29_10_GR_ChDB_0079 | Methotrexate | 1uM | 16h | FUBP1 | 0.00 | 0.47 |
| HT29_10_GR_ChDB_0079 | Methotrexate | 1uM | 16h | MAGOHB | 0.00 | 0.35 |
| HT29_10_GR_ChDB_0079 | Methotrexate | 1uM | 16h | UTP3 | 0.00 | 0.45 |
| HT29_10_GR_ChDB_0079 | Methotrexate | 1uM | 16h | KRT20 | 0.00 | 3.83 |
| HT29_10_GR_ChDB_0079 | Methotrexate | 1uM | 16h | SFT2D2 | 0.00 | 2.15 |
| HT29_10_GR_ChDB_0079 | Methotrexate | 1uM | 16h | STAU2 | 0.00 | 2.13 |
| HT29_10_GR_ChDB_0079 | Methotrexate | 1uM | 16h | CBX5 | 0.00 | 2.66 |
| HT29_10_GR_ChDB_0079 | Methotrexate | 1uM | 16h | RHOF | 0.00 | 2.13 |
| HT29_10_GR_ChDB_0079 | Methotrexate | 1uM | 16h | ZNF362 | 0.00 | 2.27 |
| HT29_10_GR_ChDB_0079 | Methotrexate | 1uM | 16h | UBE2G2 | 0.00 | 0.39 |
| HT29_10_GR_ChDB_0079 | Methotrexate | 1uM | 16h | POGK | 0.00 | 0.47 |
| HT29_10_GR_ChDB_0079 | Methotrexate | 1uM | 16h | RWDD2B | 0.00 | 2.48 |
| HT29_10_GR_ChDB_0079 | Methotrexate | 1uM | 16h | BDH2 | 0.00 | 2.03 |
| HT29_10_GR_ChDB_0079 | Methotrexate | 1uM | 16h | TGFBI | 0.00 | 4.18 |
| HT29_10_GR_ChDB_0079 | Methotrexate | 1uM | 16h | AKIP1 | 0.00 | 2.02 |
| HT29_10_GR_ChDB_0079 | Methotrexate | 1uM | 16h | PPL | 0.00 | 4.46 |
| HT29_10_GR_ChDB_0079 | Methotrexate | 1uM | 16h | LARP6 | 0.00 | 2.25 |
| HT29_10_GR_ChDB_0079 | Methotrexate | 1uM | 16h | PWP2 | 0.00 | 0.48 |
| HT29_10_GR_ChDB_0079 | Methotrexate | 1uM | 16h | RPUSD2 | 0.00 | 0.28 |
| HT29_10_GR_ChDB_0079 | Methotrexate | 1uM | 16h | WDR4 | 0.00 | 0.36 |
| HT29_10_GR_ChDB_0079 | Methotrexate | 1uM | 16h | AGPS | 0.00 | 0.32 |
| HT29_10_GR_ChDB_0079 | Methotrexate | 1uM | 16h | UBE2O | 0.00 | 0.48 |
| HT29_10_GR_ChDB_0079 | Methotrexate | 1uM | 16h | SRSF8 | 0.00 | 0.43 |
| HT29_10_GR_ChDB_0079 | Methotrexate | 1uM | 16h | H1F0 | 0.00 | 0.43 |
| HT29_10_GR_ChDB_0079 | Methotrexate | 1uM | 16h | SACS | 0.00 | 0.48 |
| HT29_10_GR_ChDB_0079 | Methotrexate | 1uM | 16h | AEN | 0.00 | 0.46 |
| HT29_10_GR_ChDB_0079 | Methotrexate | 1uM | 16h | OSBPL3 | 0.00 | 0.50 |
| HT29_10_GR_ChDB_0079 | Methotrexate | 1uM | 16h | LAMB1 | 0.00 | 2.15 |
| HT29_10_GR_ChDB_0079 | Methotrexate | 1uM | 16h | SNX5 | 0.00 | 3.69 |
| HT29_10_GR_ChDB_0079 | Methotrexate | 1uM | 16h | CKLF | 0.00 | 2.23 |
| HT29_10_GR_ChDB_0079 | Methotrexate | 1uM | 16h | DNAAF2 | 0.00 | 0.50 |
| HT29_10_GR_ChDB_0079 | Methotrexate | 1uM | 16h | SELRC1 | 0.00 | 0.50 |
| HT29_10_GR_ChDB_0079 | Methotrexate | 1uM | 16h | H2BFS | 0.00 | 0.40 |
| HT29_10_GR_ChDB_0079 | Methotrexate | 1uM | 16h | TBL1XR1 | 0.00 | 0.49 |
| HT29_10_GR_ChDB_0079 | Methotrexate | 1uM | 16h | TMEM8A | 0.00 | 2.15 |
| HT29_10_GR_ChDB_0079 | Methotrexate | 1uM | 16h | ARFGAP3 | 0.00 | 2.09 |
| HT29_10_GR_ChDB_0079 | Methotrexate | 1uM | 16h | RTN2 | 0.00 | 2.14 |
| HT29_10_GR_ChDB_0079 | Methotrexate | 1uM | 16h | TMEM161A | 0.00 | 2.02 |
| HT29_10_GR_ChDB_0079 | Methotrexate | 1uM | 16h | LXN | 0.00 | 2.72 |
| HT29_10_GR_ChDB_0079 | Methotrexate | 1uM | 16h | PMEPA1 | 0.00 | 2.36 |
| HT29_10_GR_ChDB_0079 | Methotrexate | 1uM | 16h | MFF | 0.00 | 2.00 |
| HT29_10_GR_ChDB_0079 | Methotrexate | 1uM | 16h | MMACHC | 0.00 | 0.45 |
| HT29_10_GR_ChDB_0079 | Methotrexate | 1uM | 16h | TXLNG | 0.00 | 0.46 |
| HT29_10_GR_ChDB_0079 | Methotrexate | 1uM | 16h | TBC1D30 | 0.00 | 0.43 |
| HT29_10_GR_ChDB_0079 | Methotrexate | 1uM | 16h | SMC6 | 0.00 | 0.47 |
| HT29_10_GR_ChDB_0079 | Methotrexate | 1uM | 16h | RHOB | 0.00 | 2.08 |
| HT29_10_GR_ChDB_0079 | Methotrexate | 1uM | 16h | MSLN | 0.00 | 2.77 |
| HT29_10_GR_ChDB_0079 | Methotrexate | 1uM | 16h | SPATA20 | 0.00 | 2.37 |
| HT29_10_GR_ChDB_0079 | Methotrexate | 1uM | 16h | PDLIM2 | 0.00 | 2.76 |
| HT29_10_GR_ChDB_0079 | Methotrexate | 1uM | 16h | RTN3 | 0.00 | 2.17 |
| HT29_10_GR_ChDB_0079 | Methotrexate | 1uM | 16h | WDR26 | 0.00 | 2.25 |
| HT29_10_GR_ChDB_0079 | Methotrexate | 1uM | 16h | CLK4 | 0.00 | 2.02 |
| HT29_10_GR_ChDB_0079 | Methotrexate | 1uM | 16h | DDX31 | 0.00 | 0.42 |
| HT29_10_GR_ChDB_0079 | Methotrexate | 1uM | 16h | AKR1B10 | 0.00 | 0.39 |
| HT29_10_GR_ChDB_0079 | Methotrexate | 1uM | 16h | FAM49B | 0.00 | 0.31 |
| HT29_10_GR_ChDB_0079 | Methotrexate | 1uM | 16h | LAMC1 | 0.00 | 2.12 |
| HT29_10_GR_ChDB_0079 | Methotrexate | 1uM | 16h | SKI | 0.00 | 0.49 |
| HT29_10_GR_ChDB_0079 | Methotrexate | 1uM | 16h | ACTR3B | 0.00 | 0.43 |
| HT29_10_GR_ChDB_0079 | Methotrexate | 1uM | 16h | SLCO4A1 | 0.00 | 0.47 |
| HT29_10_GR_ChDB_0079 | Methotrexate | 1uM | 16h | TRMT13 | 0.00 | 0.45 |
| HT29_10_GR_ChDB_0079 | Methotrexate | 1uM | 16h | CDT1 | 0.00 | 2.72 |
| HT29_10_GR_ChDB_0079 | Methotrexate | 1uM | 16h | TMEM106C | 0.00 | 2.30 |
| HT29_10_GR_ChDB_0079 | Methotrexate | 1uM | 16h | KLF13 | 0.00 | 0.48 |
| HT29_10_GR_ChDB_0079 | Methotrexate | 1uM | 16h | GSE1 | 0.00 | 0.44 |
| HT29_10_GR_ChDB_0079 | Methotrexate | 1uM | 16h | DLEU2 | 0.00 | 0.31 |
| HT29_10_GR_ChDB_0079 | Methotrexate | 1uM | 16h | USP36 | 0.00 | 0.47 |
| HT29_10_GR_ChDB_0079 | Methotrexate | 1uM | 16h | PCF11 | 0.00 | 0.44 |
| HT29_10_GR_ChDB_0079 | Methotrexate | 1uM | 16h | IFT57 | 0.00 | 2.19 |
| HT29_10_GR_ChDB_0079 | Methotrexate | 1uM | 16h | XRCC1 | 0.00 | 2.32 |
| HT29_10_GR_ChDB_0079 | Methotrexate | 1uM | 16h | ZCCHC2 | 0.00 | 0.42 |
| HT29_10_GR_ChDB_0079 | Methotrexate | 1uM | 16h | NHLRC2 | 0.00 | 0.49 |
| HT29_10_GR_ChDB_0079 | Methotrexate | 1uM | 16h | HELLS | 0.00 | 0.42 |
| HT29_10_GR_ChDB_0079 | Methotrexate | 1uM | 16h | LETMD1 | 0.00 | 3.10 |
| HT29_10_GR_ChDB_0079 | Methotrexate | 1uM | 16h | IFRD1 | 0.00 | 3.21 |
| HT29_10_GR_ChDB_0079 | Methotrexate | 1uM | 16h | TPM3 | 0.00 | 0.48 |
| HT29_10_GR_ChDB_0079 | Methotrexate | 1uM | 16h | C1orf109 | 0.00 | 0.33 |
| HT29_10_GR_ChDB_0079 | Methotrexate | 1uM | 16h | ZNF239 | 0.00 | 0.47 |
| HT29_10_GR_ChDB_0079 | Methotrexate | 1uM | 16h | SOWAHC | 0.00 | 0.47 |
| HT29_10_GR_ChDB_0079 | Methotrexate | 1uM | 16h | TNS4 | 0.00 | 0.45 |
| HT29_10_GR_ChDB_0079 | Methotrexate | 1uM | 16h | XIST | 0.00 | 0.48 |
| HT29_10_GR_ChDB_0079 | Methotrexate | 1uM | 16h | VPS41 | 0.00 | 0.40 |
| HT29_10_GR_ChDB_0079 | Methotrexate | 1uM | 16h | PITPNM1 | 0.00 | 2.10 |
| HT29_10_GR_ChDB_0079 | Methotrexate | 1uM | 16h | UGT8 | 0.00 | 0.49 |
| HT29_10_GR_ChDB_0079 | Methotrexate | 1uM | 16h | POLR1B | 0.00 | 0.42 |
| HT29_10_GR_ChDB_0079 | Methotrexate | 1uM | 16h | GPATCH4 | 0.00 | 0.37 |
| HT29_10_GR_ChDB_0079 | Methotrexate | 1uM | 16h | GTF2H2B | 0.00 | 0.23 |
| HT29_10_GR_ChDB_0079 | Methotrexate | 1uM | 16h | ECE2 | 0.00 | 0.48 |
| HT29_10_GR_ChDB_0079 | Methotrexate | 1uM | 16h | BHLHE41 | 0.00 | 0.50 |
| HT29_10_GR_ChDB_0079 | Methotrexate | 1uM | 16h | LFNG | 0.00 | 0.49 |
| HT29_10_GR_ChDB_0080 | 6-Mercaptopurine | 100uM | 16h | BRAF | 1.00 | 2.27 |
| HT29_10_GR_ChDB_0080 | 6-Mercaptopurine | 100uM | 16h | MYC | 0.86 | 0.47 |
| HT29_10_GR_ChDB_0080 | 6-Mercaptopurine | 100uM | 16h | NDUFAF4 | 0.25 | 0.48 |
| HT29_10_GR_ChDB_0080 | 6-Mercaptopurine | 100uM | 16h | SQSTM1 | 0.10 | 2.41 |
| HT29_10_GR_ChDB_0080 | 6-Mercaptopurine | 100uM | 16h | ANG | 0.08 | 2.81 |
| HT29_10_GR_ChDB_0080 | 6-Mercaptopurine | 100uM | 16h | IL8 | 0.06 | 4.14 |
| HT29_10_GR_ChDB_0080 | 6-Mercaptopurine | 100uM | 16h | COL4A1 | 0.05 | 5.02 |
| HT29_10_GR_ChDB_0080 | 6-Mercaptopurine | 100uM | 16h | AKR1C3 | 0.05 | 7.53 |
| HT29_10_GR_ChDB_0080 | 6-Mercaptopurine | 100uM | 16h | PTK2 | 0.04 | 2.26 |
| HT29_10_GR_ChDB_0080 | 6-Mercaptopurine | 100uM | 16h | AKR1C1 | 0.03 | 7.46 |
| HT29_10_GR_ChDB_0080 | 6-Mercaptopurine | 100uM | 16h | CPD | 0.03 | 2.05 |
| HT29_10_GR_ChDB_0080 | 6-Mercaptopurine | 100uM | 16h | EGR1 | 0.03 | 2.89 |
| HT29_10_GR_ChDB_0080 | 6-Mercaptopurine | 100uM | 16h | INSIG1 | 0.02 | 0.41 |
| HT29_10_GR_ChDB_0080 | 6-Mercaptopurine | 100uM | 16h | HNMT | 0.02 | 2.84 |
| HT29_10_GR_ChDB_0080 | 6-Mercaptopurine | 100uM | 16h | DDIT4 | 0.02 | 0.41 |
| HT29_10_GR_ChDB_0080 | 6-Mercaptopurine | 100uM | 16h | BAMBI | 0.02 | 0.48 |
| HT29_10_GR_ChDB_0080 | 6-Mercaptopurine | 100uM | 16h | SMAD3 | 0.02 | 2.33 |
| HT29_10_GR_ChDB_0080 | 6-Mercaptopurine | 100uM | 16h | RBMS1 | 0.02 | 0.50 |
| HT29_10_GR_ChDB_0080 | 6-Mercaptopurine | 100uM | 16h | RNASE4 | 0.02 | 3.60 |
| HT29_10_GR_ChDB_0080 | 6-Mercaptopurine | 100uM | 16h | CA12 | 0.02 | 0.36 |
| HT29_10_GR_ChDB_0080 | 6-Mercaptopurine | 100uM | 16h | CYP3A5 | 0.02 | 2.16 |
| HT29_10_GR_ChDB_0080 | 6-Mercaptopurine | 100uM | 16h | BCL6 | 0.02 | 2.12 |
| HT29_10_GR_ChDB_0080 | 6-Mercaptopurine | 100uM | 16h | TSPAN4 | 0.02 | 0.49 |
| HT29_10_GR_ChDB_0080 | 6-Mercaptopurine | 100uM | 16h | YAP1 | 0.01 | 2.44 |
| HT29_10_GR_ChDB_0080 | 6-Mercaptopurine | 100uM | 16h | PTP4A3 | 0.01 | 2.10 |
| HT29_10_GR_ChDB_0080 | 6-Mercaptopurine | 100uM | 16h | CDC25A | 0.01 | 0.39 |
| HT29_10_GR_ChDB_0080 | 6-Mercaptopurine | 100uM | 16h | TUBB3 | 0.01 | 0.48 |
| HT29_10_GR_ChDB_0080 | 6-Mercaptopurine | 100uM | 16h | HSPA5 | 0.01 | 0.44 |
| HT29_10_GR_ChDB_0080 | 6-Mercaptopurine | 100uM | 16h | CREB3 | 0.01 | 2.04 |
| HT29_10_GR_ChDB_0080 | 6-Mercaptopurine | 100uM | 16h | HIST1H2BD | 0.01 | 3.45 |
| HT29_10_GR_ChDB_0080 | 6-Mercaptopurine | 100uM | 16h | PCSK9 | 0.01 | 0.26 |
| HT29_10_GR_ChDB_0080 | 6-Mercaptopurine | 100uM | 16h | JUN | 0.01 | 2.41 |
| HT29_10_GR_ChDB_0080 | 6-Mercaptopurine | 100uM | 16h | EXOSC4 | 0.01 | 0.42 |
| HT29_10_GR_ChDB_0080 | 6-Mercaptopurine | 100uM | 16h | EHF | 0.01 | 0.47 |
| HT29_10_GR_ChDB_0080 | 6-Mercaptopurine | 100uM | 16h | SSBP4 | 0.01 | 0.48 |
| HT29_10_GR_ChDB_0080 | 6-Mercaptopurine | 100uM | 16h | SMURF2 | 0.01 | 2.47 |
| HT29_10_GR_ChDB_0080 | 6-Mercaptopurine | 100uM | 16h | SAT1 | 0.01 | 2.48 |
| HT29_10_GR_ChDB_0080 | 6-Mercaptopurine | 100uM | 16h | RRAGC | 0.01 | 2.16 |
| HT29_10_GR_ChDB_0080 | 6-Mercaptopurine | 100uM | 16h | MBNL2 | 0.01 | 2.35 |
| HT29_10_GR_ChDB_0080 | 6-Mercaptopurine | 100uM | 16h | SNAPC4 | 0.01 | 0.45 |
| HT29_10_GR_ChDB_0080 | 6-Mercaptopurine | 100uM | 16h | HEATR1 | 0.01 | 0.46 |
| HT29_10_GR_ChDB_0080 | 6-Mercaptopurine | 100uM | 16h | DHRS2 | 0.01 | 5.41 |
| HT29_10_GR_ChDB_0080 | 6-Mercaptopurine | 100uM | 16h | DDAH2 | 0.01 | 2.25 |
| HT29_10_GR_ChDB_0080 | 6-Mercaptopurine | 100uM | 16h | PRKAR1A | 0.01 | 2.80 |
| HT29_10_GR_ChDB_0080 | 6-Mercaptopurine | 100uM | 16h | ABHD2 | 0.01 | 2.29 |
| HT29_10_GR_ChDB_0080 | 6-Mercaptopurine | 100uM | 16h | TRIM16 | 0.01 | 2.14 |
| HT29_10_GR_ChDB_0080 | 6-Mercaptopurine | 100uM | 16h | MXD1 | 0.01 | 2.06 |
| HT29_10_GR_ChDB_0080 | 6-Mercaptopurine | 100uM | 16h | AK2 | 0.01 | 0.30 |
| HT29_10_GR_ChDB_0080 | 6-Mercaptopurine | 100uM | 16h | PDXK | 0.01 | 0.43 |
| HT29_10_GR_ChDB_0080 | 6-Mercaptopurine | 100uM | 16h | GNAI1 | 0.01 | 2.13 |
| HT29_10_GR_ChDB_0080 | 6-Mercaptopurine | 100uM | 16h | RFX5 | 0.01 | 2.15 |
| HT29_10_GR_ChDB_0080 | 6-Mercaptopurine | 100uM | 16h | MLKL | 0.01 | 0.47 |
| HT29_10_GR_ChDB_0080 | 6-Mercaptopurine | 100uM | 16h | DLAT | 0.00 | 0.49 |
| HT29_10_GR_ChDB_0080 | 6-Mercaptopurine | 100uM | 16h | UGCG | 0.00 | 0.45 |
| HT29_10_GR_ChDB_0080 | 6-Mercaptopurine | 100uM | 16h | JMJD6 | 0.00 | 0.48 |
| HT29_10_GR_ChDB_0080 | 6-Mercaptopurine | 100uM | 16h | STX4 | 0.00 | 2.63 |
| HT29_10_GR_ChDB_0080 | 6-Mercaptopurine | 100uM | 16h | RHOBTB1 | 0.00 | 2.94 |
| HT29_10_GR_ChDB_0080 | 6-Mercaptopurine | 100uM | 16h | PBXIP1 | 0.00 | 2.01 |
| HT29_10_GR_ChDB_0080 | 6-Mercaptopurine | 100uM | 16h | TACC3 | 0.00 | 0.38 |
| HT29_10_GR_ChDB_0080 | 6-Mercaptopurine | 100uM | 16h | SQLE | 0.00 | 0.43 |
| HT29_10_GR_ChDB_0080 | 6-Mercaptopurine | 100uM | 16h | SLC16A3 | 0.00 | 0.44 |
| HT29_10_GR_ChDB_0080 | 6-Mercaptopurine | 100uM | 16h | TNFRSF10A | 0.00 | 0.50 |
| HT29_10_GR_ChDB_0080 | 6-Mercaptopurine | 100uM | 16h | MTAP | 0.00 | 0.42 |
| HT29_10_GR_ChDB_0080 | 6-Mercaptopurine | 100uM | 16h | RRS1 | 0.00 | 0.49 |
| HT29_10_GR_ChDB_0080 | 6-Mercaptopurine | 100uM | 16h | KLHDC9 | 0.00 | 2.19 |
| HT29_10_GR_ChDB_0080 | 6-Mercaptopurine | 100uM | 16h | DDIT3 | 0.00 | 3.78 |
| HT29_10_GR_ChDB_0080 | 6-Mercaptopurine | 100uM | 16h | ELAVL1 | 0.00 | 0.49 |
| HT29_10_GR_ChDB_0080 | 6-Mercaptopurine | 100uM | 16h | FASN | 0.00 | 0.43 |
| HT29_10_GR_ChDB_0080 | 6-Mercaptopurine | 100uM | 16h | CXCR4 | 0.00 | 0.44 |
| HT29_10_GR_ChDB_0080 | 6-Mercaptopurine | 100uM | 16h | HIST2H2BE | 0.00 | 2.01 |
| HT29_10_GR_ChDB_0080 | 6-Mercaptopurine | 100uM | 16h | WHSC1L1 | 0.00 | 2.72 |
| HT29_10_GR_ChDB_0080 | 6-Mercaptopurine | 100uM | 16h | PLK2 | 0.00 | 5.09 |
| HT29_10_GR_ChDB_0080 | 6-Mercaptopurine | 100uM | 16h | CXADR | 0.00 | 2.14 |
| HT29_10_GR_ChDB_0080 | 6-Mercaptopurine | 100uM | 16h | RXRA | 0.00 | 0.34 |
| HT29_10_GR_ChDB_0080 | 6-Mercaptopurine | 100uM | 16h | PPARGC1B | 0.00 | 0.37 |
| HT29_10_GR_ChDB_0080 | 6-Mercaptopurine | 100uM | 16h | DICER1 | 0.00 | 0.49 |
| HT29_10_GR_ChDB_0080 | 6-Mercaptopurine | 100uM | 16h | HMGCS1 | 0.00 | 0.48 |
| HT29_10_GR_ChDB_0080 | 6-Mercaptopurine | 100uM | 16h | MUC1 | 0.00 | 0.40 |
| HT29_10_GR_ChDB_0080 | 6-Mercaptopurine | 100uM | 16h | NEU1 | 0.00 | 4.85 |
| HT29_10_GR_ChDB_0080 | 6-Mercaptopurine | 100uM | 16h | ZNF451 | 0.00 | 0.50 |
| HT29_10_GR_ChDB_0080 | 6-Mercaptopurine | 100uM | 16h | PAFAH1B2 | 0.00 | 0.46 |
| HT29_10_GR_ChDB_0080 | 6-Mercaptopurine | 100uM | 16h | IRAK2 | 0.00 | 2.12 |
| HT29_10_GR_ChDB_0080 | 6-Mercaptopurine | 100uM | 16h | NAGK | 0.00 | 2.37 |
| HT29_10_GR_ChDB_0080 | 6-Mercaptopurine | 100uM | 16h | GCLC | 0.00 | 2.14 |
| HT29_10_GR_ChDB_0080 | 6-Mercaptopurine | 100uM | 16h | CEACAM1 | 0.00 | 0.33 |
| HT29_10_GR_ChDB_0080 | 6-Mercaptopurine | 100uM | 16h | EIF5 | 0.00 | 0.49 |
| HT29_10_GR_ChDB_0080 | 6-Mercaptopurine | 100uM | 16h | HBP1 | 0.00 | 2.54 |
| HT29_10_GR_ChDB_0080 | 6-Mercaptopurine | 100uM | 16h | LMO4 | 0.00 | 0.32 |
| HT29_10_GR_ChDB_0080 | 6-Mercaptopurine | 100uM | 16h | NAA25 | 0.00 | 0.50 |
| HT29_10_GR_ChDB_0080 | 6-Mercaptopurine | 100uM | 16h | E2F3 | 0.00 | 0.44 |
| HT29_10_GR_ChDB_0080 | 6-Mercaptopurine | 100uM | 16h | HIST1H2AC | 0.00 | 6.26 |
| HT29_10_GR_ChDB_0080 | 6-Mercaptopurine | 100uM | 16h | SLC25A14 | 0.00 | 2.07 |
| HT29_10_GR_ChDB_0080 | 6-Mercaptopurine | 100uM | 16h | FOSL1 | 0.00 | 0.41 |
| HT29_10_GR_ChDB_0080 | 6-Mercaptopurine | 100uM | 16h | PA2G4 | 0.00 | 0.44 |
| HT29_10_GR_ChDB_0080 | 6-Mercaptopurine | 100uM | 16h | GART | 0.00 | 0.47 |
| HT29_10_GR_ChDB_0080 | 6-Mercaptopurine | 100uM | 16h | HMOX1 | 0.00 | 0.41 |
| HT29_10_GR_ChDB_0080 | 6-Mercaptopurine | 100uM | 16h | IMPA1 | 0.00 | 2.01 |
| HT29_10_GR_ChDB_0080 | 6-Mercaptopurine | 100uM | 16h | PPP2R1A | 0.00 | 0.46 |
| HT29_10_GR_ChDB_0080 | 6-Mercaptopurine | 100uM | 16h | DCUN1D4 | 0.00 | 0.37 |
| HT29_10_GR_ChDB_0080 | 6-Mercaptopurine | 100uM | 16h | NOLC1 | 0.00 | 0.48 |
| HT29_10_GR_ChDB_0080 | 6-Mercaptopurine | 100uM | 16h | HSPA14 | 0.00 | 0.50 |
| HT29_10_GR_ChDB_0080 | 6-Mercaptopurine | 100uM | 16h | NR2C2 | 0.00 | 0.40 |
| HT29_10_GR_ChDB_0080 | 6-Mercaptopurine | 100uM | 16h | EEF2K | 0.00 | 0.44 |
| HT29_10_GR_ChDB_0080 | 6-Mercaptopurine | 100uM | 16h | IMPDH2 | 0.00 | 2.11 |
| HT29_10_GR_ChDB_0080 | 6-Mercaptopurine | 100uM | 16h | SLC25A15 | 0.00 | 0.50 |
| HT29_10_GR_ChDB_0080 | 6-Mercaptopurine | 100uM | 16h | G3BP1 | 0.00 | 0.47 |
| HT29_10_GR_ChDB_0080 | 6-Mercaptopurine | 100uM | 16h | POLR2D | 0.00 | 0.49 |
| HT29_10_GR_ChDB_0080 | 6-Mercaptopurine | 100uM | 16h | CEACAM6 | 0.00 | 0.35 |
| HT29_10_GR_ChDB_0080 | 6-Mercaptopurine | 100uM | 16h | RAB23 | 0.00 | 0.37 |
| HT29_10_GR_ChDB_0080 | 6-Mercaptopurine | 100uM | 16h | NF2 | 0.00 | 0.50 |
| HT29_10_GR_ChDB_0080 | 6-Mercaptopurine | 100uM | 16h | TNFRSF11A | 0.00 | 0.41 |
| HT29_10_GR_ChDB_0080 | 6-Mercaptopurine | 100uM | 16h | SKP2 | 0.00 | 0.41 |
| HT29_10_GR_ChDB_0080 | 6-Mercaptopurine | 100uM | 16h | ADI1 | 0.00 | 2.26 |
| HT29_10_GR_ChDB_0080 | 6-Mercaptopurine | 100uM | 16h | ATG4B | 0.00 | 0.43 |
| HT29_10_GR_ChDB_0080 | 6-Mercaptopurine | 100uM | 16h | ERO1L | 0.00 | 2.06 |
| HT29_10_GR_ChDB_0080 | 6-Mercaptopurine | 100uM | 16h | NAA50 | 0.00 | 0.44 |
| HT29_10_GR_ChDB_0080 | 6-Mercaptopurine | 100uM | 16h | IDS | 0.00 | 2.36 |
| HT29_10_GR_ChDB_0080 | 6-Mercaptopurine | 100uM | 16h | DST | 0.00 | 2.36 |
| HT29_10_GR_ChDB_0080 | 6-Mercaptopurine | 100uM | 16h | NF1 | 0.00 | 2.39 |
| HT29_10_GR_ChDB_0080 | 6-Mercaptopurine | 100uM | 16h | ANKRD10 | 0.00 | 2.38 |
| HT29_10_GR_ChDB_0080 | 6-Mercaptopurine | 100uM | 16h | PRMT2 | 0.00 | 2.31 |
| HT29_10_GR_ChDB_0080 | 6-Mercaptopurine | 100uM | 16h | UGT2B15 | 0.00 | 2.38 |
| HT29_10_GR_ChDB_0080 | 6-Mercaptopurine | 100uM | 16h | SYNCRIP | 0.00 | 0.45 |
| HT29_10_GR_ChDB_0080 | 6-Mercaptopurine | 100uM | 16h | VSNL1 | 0.00 | 2.04 |
| HT29_10_GR_ChDB_0080 | 6-Mercaptopurine | 100uM | 16h | MZF1 | 0.00 | 2.12 |
| HT29_10_GR_ChDB_0080 | 6-Mercaptopurine | 100uM | 16h | ATF7IP | 0.00 | 2.15 |
| HT29_10_GR_ChDB_0080 | 6-Mercaptopurine | 100uM | 16h | LAMA3 | 0.00 | 2.17 |
| HT29_10_GR_ChDB_0080 | 6-Mercaptopurine | 100uM | 16h | MBD1 | 0.00 | 2.18 |
| HT29_10_GR_ChDB_0080 | 6-Mercaptopurine | 100uM | 16h | MAPRE2 | 0.00 | 3.06 |
| HT29_10_GR_ChDB_0080 | 6-Mercaptopurine | 100uM | 16h | KIAA0141 | 0.00 | 2.14 |
| HT29_10_GR_ChDB_0080 | 6-Mercaptopurine | 100uM | 16h | AAMDC | 0.00 | 2.38 |
| HT29_10_GR_ChDB_0080 | 6-Mercaptopurine | 100uM | 16h | UBE2D3 | 0.00 | 0.47 |
| HT29_10_GR_ChDB_0080 | 6-Mercaptopurine | 100uM | 16h | EIF5B | 0.00 | 0.47 |
| HT29_10_GR_ChDB_0080 | 6-Mercaptopurine | 100uM | 16h | PSME3 | 0.00 | 0.43 |
| HT29_10_GR_ChDB_0080 | 6-Mercaptopurine | 100uM | 16h | ST3GAL5 | 0.00 | 2.58 |
| HT29_10_GR_ChDB_0080 | 6-Mercaptopurine | 100uM | 16h | DNAJB12 | 0.00 | 2.06 |
| HT29_10_GR_ChDB_0080 | 6-Mercaptopurine | 100uM | 16h | FAM63A | 0.00 | 2.14 |
| HT29_10_GR_ChDB_0080 | 6-Mercaptopurine | 100uM | 16h | CSRNP2 | 0.00 | 3.04 |
| HT29_10_GR_ChDB_0080 | 6-Mercaptopurine | 100uM | 16h | GULP1 | 0.00 | 4.01 |
| HT29_10_GR_ChDB_0080 | 6-Mercaptopurine | 100uM | 16h | EIF1 | 0.00 | 2.03 |
| HT29_10_GR_ChDB_0080 | 6-Mercaptopurine | 100uM | 16h | ABI2 | 0.00 | 2.16 |
| HT29_10_GR_ChDB_0080 | 6-Mercaptopurine | 100uM | 16h | APLP2 | 0.00 | 2.30 |
| HT29_10_GR_ChDB_0080 | 6-Mercaptopurine | 100uM | 16h | METTL13 | 0.00 | 0.49 |
| HT29_10_GR_ChDB_0080 | 6-Mercaptopurine | 100uM | 16h | GOLGA2 | 0.00 | 2.04 |
| HT29_10_GR_ChDB_0080 | 6-Mercaptopurine | 100uM | 16h | ATG12 | 0.00 | 3.96 |
| HT29_10_GR_ChDB_0080 | 6-Mercaptopurine | 100uM | 16h | TAF9B | 0.00 | 2.03 |
| HT29_10_GR_ChDB_0080 | 6-Mercaptopurine | 100uM | 16h | VRK3 | 0.00 | 2.30 |
| HT29_10_GR_ChDB_0080 | 6-Mercaptopurine | 100uM | 16h | NOP16 | 0.00 | 0.42 |
| HT29_10_GR_ChDB_0080 | 6-Mercaptopurine | 100uM | 16h | SCD | 0.00 | 0.45 |
| HT29_10_GR_ChDB_0080 | 6-Mercaptopurine | 100uM | 16h | UBE2G2 | 0.00 | 0.46 |
| HT29_10_GR_ChDB_0080 | 6-Mercaptopurine | 100uM | 16h | SLC5A6 | 0.00 | 0.45 |
| HT29_10_GR_ChDB_0080 | 6-Mercaptopurine | 100uM | 16h | HLA-E | 0.00 | 2.33 |
| HT29_10_GR_ChDB_0080 | 6-Mercaptopurine | 100uM | 16h | HDLBP | 0.00 | 2.75 |
| HT29_10_GR_ChDB_0080 | 6-Mercaptopurine | 100uM | 16h | RGS2 | 0.00 | 3.32 |
| HT29_10_GR_ChDB_0080 | 6-Mercaptopurine | 100uM | 16h | GTDC1 | 0.00 | 2.15 |
| HT29_10_GR_ChDB_0080 | 6-Mercaptopurine | 100uM | 16h | RANBP2 | 0.00 | 0.48 |
| HT29_10_GR_ChDB_0080 | 6-Mercaptopurine | 100uM | 16h | MRPS12 | 0.00 | 0.37 |
| HT29_10_GR_ChDB_0080 | 6-Mercaptopurine | 100uM | 16h | ATP13A3 | 0.00 | 0.43 |
| HT29_10_GR_ChDB_0080 | 6-Mercaptopurine | 100uM | 16h | GTPBP4 | 0.00 | 0.45 |
| HT29_10_GR_ChDB_0080 | 6-Mercaptopurine | 100uM | 16h | LAMP2 | 0.00 | 2.08 |
| HT29_10_GR_ChDB_0080 | 6-Mercaptopurine | 100uM | 16h | UBE2H | 0.00 | 2.23 |
| HT29_10_GR_ChDB_0080 | 6-Mercaptopurine | 100uM | 16h | CLN5 | 0.00 | 2.06 |
| HT29_10_GR_ChDB_0080 | 6-Mercaptopurine | 100uM | 16h | TRIM52 | 0.00 | 2.19 |
| HT29_10_GR_ChDB_0080 | 6-Mercaptopurine | 100uM | 16h | AGPAT4 | 0.00 | 2.20 |
| HT29_10_GR_ChDB_0080 | 6-Mercaptopurine | 100uM | 16h | ARSD | 0.00 | 2.55 |
| HT29_10_GR_ChDB_0080 | 6-Mercaptopurine | 100uM | 16h | C1orf63 | 0.00 | 2.34 |
| HT29_10_GR_ChDB_0080 | 6-Mercaptopurine | 100uM | 16h | CSGALNACT2 | 0.00 | 3.24 |
| HT29_10_GR_ChDB_0080 | 6-Mercaptopurine | 100uM | 16h | FUBP1 | 0.00 | 2.07 |
| HT29_10_GR_ChDB_0080 | 6-Mercaptopurine | 100uM | 16h | KLHL24 | 0.00 | 2.65 |
| HT29_10_GR_ChDB_0080 | 6-Mercaptopurine | 100uM | 16h | KRCC1 | 0.00 | 2.08 |
| HT29_10_GR_ChDB_0080 | 6-Mercaptopurine | 100uM | 16h | MUT | 0.00 | 2.00 |
| HT29_10_GR_ChDB_0080 | 6-Mercaptopurine | 100uM | 16h | RAD17 | 0.00 | 2.14 |
| HT29_10_GR_ChDB_0080 | 6-Mercaptopurine | 100uM | 16h | U2SURP | 0.00 | 0.48 |
| HT29_10_GR_ChDB_0080 | 6-Mercaptopurine | 100uM | 16h | TCOF1 | 0.00 | 0.46 |
| HT29_10_GR_ChDB_0080 | 6-Mercaptopurine | 100uM | 16h | MAGED2 | 0.00 | 2.08 |
| HT29_10_GR_ChDB_0080 | 6-Mercaptopurine | 100uM | 16h | HLA-G | 0.00 | 2.34 |
| HT29_10_GR_ChDB_0080 | 6-Mercaptopurine | 100uM | 16h | GSN | 0.00 | 2.24 |
| HT29_10_GR_ChDB_0080 | 6-Mercaptopurine | 100uM | 16h | PALLD | 0.00 | 2.42 |
| HT29_10_GR_ChDB_0080 | 6-Mercaptopurine | 100uM | 16h | CCNG2 | 0.00 | 3.56 |
| HT29_10_GR_ChDB_0080 | 6-Mercaptopurine | 100uM | 16h | SMIM7 | 0.00 | 2.24 |
| HT29_10_GR_ChDB_0080 | 6-Mercaptopurine | 100uM | 16h | SYTL2 | 0.00 | 3.07 |
| HT29_10_GR_ChDB_0080 | 6-Mercaptopurine | 100uM | 16h | BBIP1 | 0.00 | 2.31 |
| HT29_10_GR_ChDB_0080 | 6-Mercaptopurine | 100uM | 16h | MARCKS | 0.00 | 2.26 |
| HT29_10_GR_ChDB_0080 | 6-Mercaptopurine | 100uM | 16h | GDF15 | 0.00 | 4.30 |
| HT29_10_GR_ChDB_0080 | 6-Mercaptopurine | 100uM | 16h | SLC19A1 | 0.00 | 0.26 |
| HT29_10_GR_ChDB_0080 | 6-Mercaptopurine | 100uM | 16h | UMPS | 0.00 | 0.47 |
| HT29_10_GR_ChDB_0080 | 6-Mercaptopurine | 100uM | 16h | NUDC | 0.00 | 0.49 |
| HT29_10_GR_ChDB_0080 | 6-Mercaptopurine | 100uM | 16h | SLC35E1 | 0.00 | 0.49 |
| HT29_10_GR_ChDB_0080 | 6-Mercaptopurine | 100uM | 16h | MINA | 0.00 | 0.49 |
| HT29_10_GR_ChDB_0080 | 6-Mercaptopurine | 100uM | 16h | CHAF1A | 0.00 | 0.42 |
| HT29_10_GR_ChDB_0080 | 6-Mercaptopurine | 100uM | 16h | PHLDA1 | 0.00 | 0.47 |
| HT29_10_GR_ChDB_0080 | 6-Mercaptopurine | 100uM | 16h | DPP4 | 0.00 | 2.47 |
| HT29_10_GR_ChDB_0080 | 6-Mercaptopurine | 100uM | 16h | FIG4 | 0.00 | 2.19 |
| HT29_10_GR_ChDB_0080 | 6-Mercaptopurine | 100uM | 16h | VSIG10 | 0.00 | 2.07 |
| HT29_10_GR_ChDB_0080 | 6-Mercaptopurine | 100uM | 16h | ULBP2 | 0.00 | 2.74 |
| HT29_10_GR_ChDB_0080 | 6-Mercaptopurine | 100uM | 16h | BBS1 | 0.00 | 2.21 |
| HT29_10_GR_ChDB_0080 | 6-Mercaptopurine | 100uM | 16h | SMIM14 | 0.00 | 2.67 |
| HT29_10_GR_ChDB_0080 | 6-Mercaptopurine | 100uM | 16h | SPC24 | 0.00 | 2.65 |
| HT29_10_GR_ChDB_0080 | 6-Mercaptopurine | 100uM | 16h | KLF11 | 0.00 | 2.97 |
| HT29_10_GR_ChDB_0080 | 6-Mercaptopurine | 100uM | 16h | GLTSCR1 | 0.00 | 2.35 |
| HT29_10_GR_ChDB_0080 | 6-Mercaptopurine | 100uM | 16h | MARCH3 | 0.00 | 2.03 |
| HT29_10_GR_ChDB_0080 | 6-Mercaptopurine | 100uM | 16h | C10orf2 | 0.00 | 0.43 |
| HT29_10_GR_ChDB_0080 | 6-Mercaptopurine | 100uM | 16h | TIMM44 | 0.00 | 0.45 |
| HT29_10_GR_ChDB_0080 | 6-Mercaptopurine | 100uM | 16h | TSR1 | 0.00 | 0.44 |
| HT29_10_GR_ChDB_0080 | 6-Mercaptopurine | 100uM | 16h | AHCTF1 | 0.00 | 0.48 |
| HT29_10_GR_ChDB_0080 | 6-Mercaptopurine | 100uM | 16h | SFXN1 | 0.00 | 0.46 |
| HT29_10_GR_ChDB_0080 | 6-Mercaptopurine | 100uM | 16h | SPATS2 | 0.00 | 2.00 |
| HT29_10_GR_ChDB_0080 | 6-Mercaptopurine | 100uM | 16h | SORL1 | 0.00 | 2.23 |
| HT29_10_GR_ChDB_0080 | 6-Mercaptopurine | 100uM | 16h | POLR3G | 0.00 | 0.47 |
| HT29_10_GR_ChDB_0080 | 6-Mercaptopurine | 100uM | 16h | MOSPD1 | 0.00 | 2.45 |
| HT29_10_GR_ChDB_0080 | 6-Mercaptopurine | 100uM | 16h | BBS4 | 0.00 | 2.33 |
| HT29_10_GR_ChDB_0080 | 6-Mercaptopurine | 100uM | 16h | SCPEP1 | 0.00 | 2.69 |
| HT29_10_GR_ChDB_0080 | 6-Mercaptopurine | 100uM | 16h | SP4 | 0.00 | 2.24 |
| HT29_10_GR_ChDB_0080 | 6-Mercaptopurine | 100uM | 16h | TSPAN31 | 0.00 | 2.07 |
| HT29_10_GR_ChDB_0080 | 6-Mercaptopurine | 100uM | 16h | PPIP5K1 | 0.00 | 2.04 |
| HT29_10_GR_ChDB_0080 | 6-Mercaptopurine | 100uM | 16h | GJA3 | 0.00 | 2.01 |
| HT29_10_GR_ChDB_0080 | 6-Mercaptopurine | 100uM | 16h | NR1D2 | 0.00 | 4.19 |
| HT29_10_GR_ChDB_0080 | 6-Mercaptopurine | 100uM | 16h | WDR45 | 0.00 | 2.56 |
| HT29_10_GR_ChDB_0080 | 6-Mercaptopurine | 100uM | 16h | SPINK1 | 0.00 | 2.33 |
| HT29_10_GR_ChDB_0080 | 6-Mercaptopurine | 100uM | 16h | ANXA11 | 0.00 | 2.04 |
| HT29_10_GR_ChDB_0080 | 6-Mercaptopurine | 100uM | 16h | CELF1 | 0.00 | 2.35 |
| HT29_10_GR_ChDB_0080 | 6-Mercaptopurine | 100uM | 16h | MBIP | 0.00 | 2.21 |
| HT29_10_GR_ChDB_0080 | 6-Mercaptopurine | 100uM | 16h | RPN2 | 0.00 | 2.00 |
| HT29_10_GR_ChDB_0080 | 6-Mercaptopurine | 100uM | 16h | RNF19B | 0.00 | 2.00 |
| HT29_10_GR_ChDB_0080 | 6-Mercaptopurine | 100uM | 16h | EMP1 | 0.00 | 0.50 |
| HT29_10_GR_ChDB_0080 | 6-Mercaptopurine | 100uM | 16h | LRFN4 | 0.00 | 0.48 |
| HT29_10_GR_ChDB_0080 | 6-Mercaptopurine | 100uM | 16h | ATAD3A | 0.00 | 0.39 |
| HT29_10_GR_ChDB_0080 | 6-Mercaptopurine | 100uM | 16h | SRSF11 | 0.00 | 0.30 |
| HT29_10_GR_ChDB_0080 | 6-Mercaptopurine | 100uM | 16h | OTUD4 | 0.00 | 0.42 |
| HT29_10_GR_ChDB_0080 | 6-Mercaptopurine | 100uM | 16h | LGALSL | 0.00 | 0.34 |
| HT29_10_GR_ChDB_0080 | 6-Mercaptopurine | 100uM | 16h | TOPORS-AS1 | 0.00 | 2.25 |
| HT29_10_GR_ChDB_0080 | 6-Mercaptopurine | 100uM | 16h | BRE | 0.00 | 2.03 |
| HT29_10_GR_ChDB_0080 | 6-Mercaptopurine | 100uM | 16h | WDR43 | 0.00 | 0.43 |
| HT29_10_GR_ChDB_0080 | 6-Mercaptopurine | 100uM | 16h | ALDOC | 0.00 | 0.41 |
| HT29_10_GR_ChDB_0080 | 6-Mercaptopurine | 100uM | 16h | POLD4 | 0.00 | 2.52 |
| HT29_10_GR_ChDB_0080 | 6-Mercaptopurine | 100uM | 16h | YPEL5 | 0.00 | 3.98 |
| HT29_10_GR_ChDB_0080 | 6-Mercaptopurine | 100uM | 16h | TNFSF15 | 0.00 | 2.10 |
| HT29_10_GR_ChDB_0080 | 6-Mercaptopurine | 100uM | 16h | AQP3 | 0.00 | 3.92 |
| HT29_10_GR_ChDB_0080 | 6-Mercaptopurine | 100uM | 16h | PINK1 | 0.00 | 2.17 |
| HT29_10_GR_ChDB_0080 | 6-Mercaptopurine | 100uM | 16h | CYTH2 | 0.00 | 2.04 |
| HT29_10_GR_ChDB_0080 | 6-Mercaptopurine | 100uM | 16h | RAB40B | 0.00 | 2.15 |
| HT29_10_GR_ChDB_0080 | 6-Mercaptopurine | 100uM | 16h | NUPR1 | 0.00 | 6.43 |
| HT29_10_GR_ChDB_0080 | 6-Mercaptopurine | 100uM | 16h | CDADC1 | 0.00 | 2.13 |
| HT29_10_GR_ChDB_0080 | 6-Mercaptopurine | 100uM | 16h | DNAJC24 | 0.00 | 2.23 |
| HT29_10_GR_ChDB_0080 | 6-Mercaptopurine | 100uM | 16h | DHTKD1 | 0.00 | 3.09 |
| HT29_10_GR_ChDB_0080 | 6-Mercaptopurine | 100uM | 16h | PPL | 0.00 | 2.16 |
| HT29_10_GR_ChDB_0080 | 6-Mercaptopurine | 100uM | 16h | APOBEC3B | 0.00 | 2.51 |
| HT29_10_GR_ChDB_0080 | 6-Mercaptopurine | 100uM | 16h | SELENBP1 | 0.00 | 2.04 |
| HT29_10_GR_ChDB_0080 | 6-Mercaptopurine | 100uM | 16h | TTC17 | 0.00 | 2.44 |
| HT29_10_GR_ChDB_0080 | 6-Mercaptopurine | 100uM | 16h | WDR5B | 0.00 | 2.46 |
| HT29_10_GR_ChDB_0080 | 6-Mercaptopurine | 100uM | 16h | FAM198B | 0.00 | 2.71 |
| HT29_10_GR_ChDB_0080 | 6-Mercaptopurine | 100uM | 16h | LMBRD1 | 0.00 | 2.06 |
| HT29_10_GR_ChDB_0080 | 6-Mercaptopurine | 100uM | 16h | TTLL12 | 0.00 | 0.49 |
| HT29_10_GR_ChDB_0080 | 6-Mercaptopurine | 100uM | 16h | NUFIP1 | 0.00 | 0.50 |
| HT29_10_GR_ChDB_0080 | 6-Mercaptopurine | 100uM | 16h | GTF3A | 0.00 | 0.41 |
| HT29_10_GR_ChDB_0080 | 6-Mercaptopurine | 100uM | 16h | DFFA | 0.00 | 0.31 |
| HT29_10_GR_ChDB_0080 | 6-Mercaptopurine | 100uM | 16h | LYPLA2 | 0.00 | 0.49 |
| HT29_10_GR_ChDB_0080 | 6-Mercaptopurine | 100uM | 16h | CALML4 | 0.00 | 0.47 |
| HT29_10_GR_ChDB_0080 | 6-Mercaptopurine | 100uM | 16h | DDX52 | 0.00 | 0.40 |
| HT29_10_GR_ChDB_0080 | 6-Mercaptopurine | 100uM | 16h | RPP25 | 0.00 | 0.43 |
| HT29_10_GR_ChDB_0080 | 6-Mercaptopurine | 100uM | 16h | WDR82 | 0.00 | 0.47 |
| HT29_10_GR_ChDB_0080 | 6-Mercaptopurine | 100uM | 16h | LPCAT1 | 0.00 | 0.48 |
| HT29_10_GR_ChDB_0080 | 6-Mercaptopurine | 100uM | 16h | C8orf33 | 0.00 | 0.47 |
| HT29_10_GR_ChDB_0080 | 6-Mercaptopurine | 100uM | 16h | RPL31 | 0.00 | 2.92 |
| HT29_10_GR_ChDB_0080 | 6-Mercaptopurine | 100uM | 16h | CLU | 0.00 | 5.53 |
| HT29_10_GR_ChDB_0080 | 6-Mercaptopurine | 100uM | 16h | ADPRM | 0.00 | 2.28 |
| HT29_10_GR_ChDB_0080 | 6-Mercaptopurine | 100uM | 16h | FAM120C | 0.00 | 2.08 |
| HT29_10_GR_ChDB_0080 | 6-Mercaptopurine | 100uM | 16h | MRP63 | 0.00 | 0.40 |
| HT29_10_GR_ChDB_0080 | 6-Mercaptopurine | 100uM | 16h | TBL1XR1 | 0.00 | 0.49 |
| HT29_10_GR_ChDB_0080 | 6-Mercaptopurine | 100uM | 16h | AP3S1 | 0.00 | 2.84 |
| HT29_10_GR_ChDB_0080 | 6-Mercaptopurine | 100uM | 16h | TMEM66 | 0.00 | 2.02 |
| HT29_10_GR_ChDB_0080 | 6-Mercaptopurine | 100uM | 16h | ORAI3 | 0.00 | 2.26 |
| HT29_10_GR_ChDB_0080 | 6-Mercaptopurine | 100uM | 16h | GAREM | 0.00 | 2.14 |
| HT29_10_GR_ChDB_0080 | 6-Mercaptopurine | 100uM | 16h | ACOT8 | 0.00 | 2.31 |
| HT29_10_GR_ChDB_0080 | 6-Mercaptopurine | 100uM | 16h | CRBN | 0.00 | 2.12 |
| HT29_10_GR_ChDB_0080 | 6-Mercaptopurine | 100uM | 16h | TTC33 | 0.00 | 2.22 |
| HT29_10_GR_ChDB_0080 | 6-Mercaptopurine | 100uM | 16h | ZNF33B | 0.00 | 2.87 |
| HT29_10_GR_ChDB_0080 | 6-Mercaptopurine | 100uM | 16h | CDKN2B | 0.00 | 5.85 |
| HT29_10_GR_ChDB_0080 | 6-Mercaptopurine | 100uM | 16h | CBX5 | 0.00 | 2.41 |
| HT29_10_GR_ChDB_0080 | 6-Mercaptopurine | 100uM | 16h | SLC12A6 | 0.00 | 2.78 |
| HT29_10_GR_ChDB_0080 | 6-Mercaptopurine | 100uM | 16h | SLC25A17 | 0.00 | 0.49 |
| HT29_10_GR_ChDB_0080 | 6-Mercaptopurine | 100uM | 16h | ZFX | 0.00 | 0.48 |
| HT29_10_GR_ChDB_0080 | 6-Mercaptopurine | 100uM | 16h | TTF2 | 0.00 | 0.35 |
| HT29_10_GR_ChDB_0080 | 6-Mercaptopurine | 100uM | 16h | LRP8 | 0.00 | 0.50 |
| HT29_10_GR_ChDB_0080 | 6-Mercaptopurine | 100uM | 16h | ACTR3B | 0.00 | 0.41 |
| HT29_10_GR_ChDB_0080 | 6-Mercaptopurine | 100uM | 16h | SDC1 | 0.00 | 0.49 |
| HT29_10_GR_ChDB_0080 | 6-Mercaptopurine | 100uM | 16h | TAF1D | 0.00 | 0.48 |
| HT29_10_GR_ChDB_0080 | 6-Mercaptopurine | 100uM | 16h | BBS10 | 0.00 | 2.04 |
| HT29_10_GR_ChDB_0080 | 6-Mercaptopurine | 100uM | 16h | SLC38A4 | 0.00 | 3.77 |
| HT29_10_GR_ChDB_0080 | 6-Mercaptopurine | 100uM | 16h | PMM1 | 0.00 | 2.37 |
| HT29_10_GR_ChDB_0080 | 6-Mercaptopurine | 100uM | 16h | TTC31 | 0.00 | 2.01 |
| HT29_10_GR_ChDB_0080 | 6-Mercaptopurine | 100uM | 16h | CCDC28A | 0.00 | 4.22 |
| HT29_10_GR_ChDB_0080 | 6-Mercaptopurine | 100uM | 16h | ALDH1B1 | 0.00 | 0.43 |
| HT29_10_GR_ChDB_0080 | 6-Mercaptopurine | 100uM | 16h | AGPAT5 | 0.00 | 0.49 |
| HT29_10_GR_ChDB_0080 | 6-Mercaptopurine | 100uM | 16h | PUS1 | 0.00 | 0.44 |
| HT29_10_GR_ChDB_0080 | 6-Mercaptopurine | 100uM | 16h | AGPS | 0.00 | 0.33 |
| HT29_10_GR_ChDB_0080 | 6-Mercaptopurine | 100uM | 16h | CD82 | 0.00 | 0.49 |
| HT29_10_GR_ChDB_0080 | 6-Mercaptopurine | 100uM | 16h | GLT8D1 | 0.00 | 2.72 |
| HT29_10_GR_ChDB_0080 | 6-Mercaptopurine | 100uM | 16h | MT1H | 0.00 | 2.09 |
| HT29_10_GR_ChDB_0080 | 6-Mercaptopurine | 100uM | 16h | PARD6A | 0.00 | 2.30 |
| HT29_10_GR_ChDB_0080 | 6-Mercaptopurine | 100uM | 16h | WDR13 | 0.00 | 2.05 |
| HT29_10_GR_ChDB_0080 | 6-Mercaptopurine | 100uM | 16h | TXNRD1 | 0.00 | 2.51 |
| HT29_10_GR_ChDB_0080 | 6-Mercaptopurine | 100uM | 16h | HCFC1R1 | 0.00 | 2.19 |
| HT29_10_GR_ChDB_0080 | 6-Mercaptopurine | 100uM | 16h | LAMB3 | 0.00 | 2.52 |
| HT29_10_GR_ChDB_0080 | 6-Mercaptopurine | 100uM | 16h | TRMU | 0.00 | 0.45 |
| HT29_10_GR_ChDB_0080 | 6-Mercaptopurine | 100uM | 16h | ANKRD28 | 0.00 | 0.44 |
| HT29_10_GR_ChDB_0080 | 6-Mercaptopurine | 100uM | 16h | XPOT | 0.00 | 0.44 |
| HT29_10_GR_ChDB_0080 | 6-Mercaptopurine | 100uM | 16h | POP1 | 0.00 | 0.47 |
| HT29_10_GR_ChDB_0080 | 6-Mercaptopurine | 100uM | 16h | CAPRIN2 | 0.00 | 2.36 |
| HT29_10_GR_ChDB_0080 | 6-Mercaptopurine | 100uM | 16h | RPL28 | 0.00 | 2.19 |
| HT29_10_GR_ChDB_0080 | 6-Mercaptopurine | 100uM | 16h | MT1X | 0.00 | 2.20 |
| HT29_10_GR_ChDB_0080 | 6-Mercaptopurine | 100uM | 16h | AKR7A3 | 0.00 | 2.11 |
| HT29_10_GR_ChDB_0080 | 6-Mercaptopurine | 100uM | 16h | STEAP4 | 0.00 | 2.34 |
| HT29_10_GR_ChDB_0080 | 6-Mercaptopurine | 100uM | 16h | PSPC1 | 0.00 | 2.70 |
| HT29_10_GR_ChDB_0080 | 6-Mercaptopurine | 100uM | 16h | CYP20A1 | 0.00 | 2.53 |
| HT29_10_GR_ChDB_0080 | 6-Mercaptopurine | 100uM | 16h | AMD1 | 0.00 | 0.45 |
| HT29_10_GR_ChDB_0080 | 6-Mercaptopurine | 100uM | 16h | HS3ST1 | 0.00 | 0.41 |
| HT29_10_GR_ChDB_0080 | 6-Mercaptopurine | 100uM | 16h | LIPG | 0.00 | 0.42 |
| HT29_10_GR_ChDB_0080 | 6-Mercaptopurine | 100uM | 16h | URB2 | 0.00 | 0.45 |
| HT29_10_GR_ChDB_0080 | 6-Mercaptopurine | 100uM | 16h | OCEL1 | 0.00 | 2.38 |
| HT29_10_GR_ChDB_0080 | 6-Mercaptopurine | 100uM | 16h | TSPYL4 | 0.00 | 2.01 |
| HT29_10_GR_ChDB_0080 | 6-Mercaptopurine | 100uM | 16h | SRSF8 | 0.00 | 0.39 |
| HT29_10_GR_ChDB_0080 | 6-Mercaptopurine | 100uM | 16h | FAM49B | 0.00 | 0.39 |
| HT29_10_GR_ChDB_0080 | 6-Mercaptopurine | 100uM | 16h | ATP9B | 0.00 | 0.34 |
| HT29_10_GR_ChDB_0080 | 6-Mercaptopurine | 100uM | 16h | SMC6 | 0.00 | 0.44 |
| HT29_10_GR_ChDB_0080 | 6-Mercaptopurine | 100uM | 16h | HELLS | 0.00 | 0.46 |
| HT29_10_GR_ChDB_0080 | 6-Mercaptopurine | 100uM | 16h | ZDHHC4 | 0.00 | 2.01 |
| HT29_10_GR_ChDB_0080 | 6-Mercaptopurine | 100uM | 16h | IFRD1 | 0.00 | 2.72 |
| HT29_10_GR_ChDB_0080 | 6-Mercaptopurine | 100uM | 16h | HIST3H2A | 0.00 | 2.97 |
| HT29_10_GR_ChDB_0080 | 6-Mercaptopurine | 100uM | 16h | SPG11 | 0.00 | 2.07 |
| HT29_10_GR_ChDB_0080 | 6-Mercaptopurine | 100uM | 16h | PDSS2 | 0.00 | 2.07 |
| HT29_10_GR_ChDB_0080 | 6-Mercaptopurine | 100uM | 16h | PPP1R10 | 0.00 | 0.48 |
| HT29_10_GR_ChDB_0080 | 6-Mercaptopurine | 100uM | 16h | TRMT61A | 0.00 | 0.48 |
| HT29_10_GR_ChDB_0080 | 6-Mercaptopurine | 100uM | 16h | BHLHE41 | 0.00 | 0.35 |
| HT29_10_GR_ChDB_0080 | 6-Mercaptopurine | 100uM | 16h | ATP11A | 0.00 | 0.50 |
| HT29_10_GR_ChDB_0080 | 6-Mercaptopurine | 100uM | 16h | PAK1IP1 | 0.00 | 0.43 |
| HT29_10_GR_ChDB_0080 | 6-Mercaptopurine | 100uM | 16h | USP36 | 0.00 | 0.48 |
| HT29_10_GR_ChDB_0080 | 6-Mercaptopurine | 100uM | 16h | GPATCH4 | 0.00 | 0.45 |
| HT29_10_GR_ChDB_0080 | 6-Mercaptopurine | 100uM | 16h | DNAJA1 | 0.00 | 0.50 |
| HT29_10_GR_ChDB_0080 | 6-Mercaptopurine | 100uM | 16h | TMEM177 | 0.00 | 0.50 |
| HT29_10_GR_ChDB_0080 | 6-Mercaptopurine | 100uM | 16h | IQGAP2 | 0.00 | 0.36 |
| HT29_10_GR_ChDB_0080 | 6-Mercaptopurine | 100uM | 16h | C2CD2 | 0.00 | 0.45 |
| HT29_10_GR_ChDB_0080 | 6-Mercaptopurine | 100uM | 16h | TMED1 | 0.00 | 2.79 |
| HT29_10_GR_ChDB_0080 | 6-Mercaptopurine | 100uM | 16h | WBP2 | 0.00 | 3.21 |
| HT29_10_GR_ChDB_0080 | 6-Mercaptopurine | 100uM | 16h | RGCC | 0.00 | 2.19 |
| HT29_10_GR_ChDB_0080 | 6-Mercaptopurine | 100uM | 16h | LETMD1 | 0.00 | 2.09 |
| HT29_10_GR_ChDB_0080 | 6-Mercaptopurine | 100uM | 16h | C11orf68 | 0.00 | 2.66 |
| HT29_10_GR_ChDB_0080 | 6-Mercaptopurine | 100uM | 16h | RHOB | 0.00 | 2.68 |
| HT29_10_GR_ChDB_0080 | 6-Mercaptopurine | 100uM | 16h | SNX5 | 0.00 | 2.93 |
| HT29_10_GR_ChDB_0080 | 6-Mercaptopurine | 100uM | 16h | TBC1D30 | 0.00 | 0.41 |
| HT29_10_GR_ChDB_0080 | 6-Mercaptopurine | 100uM | 16h | C1orf109 | 0.00 | 0.40 |
| HT29_10_GR_ChDB_0080 | 6-Mercaptopurine | 100uM | 16h | LFNG | 0.00 | 0.47 |
| HT29_10_GR_ChDB_0080 | 6-Mercaptopurine | 100uM | 16h | TTLL4 | 0.00 | 0.47 |
| HT29_10_GR_ChDB_0080 | 6-Mercaptopurine | 100uM | 16h | HPS6 | 0.00 | 0.50 |
| HT29_10_GR_ChDB_0080 | 6-Mercaptopurine | 100uM | 16h | TPM3 | 0.00 | 0.49 |
| HT29_10_GR_ChDB_0080 | 6-Mercaptopurine | 100uM | 16h | ANO1 | 0.00 | 0.44 |
| HT29_10_GR_ChDB_0080 | 6-Mercaptopurine | 100uM | 16h | UBQLN4 | 0.00 | 0.47 |
| HT29_10_GR_ChDB_0080 | 6-Mercaptopurine | 100uM | 16h | WNT11 | 0.00 | 0.27 |
| HT29_10_GR_ChDB_0080 | 6-Mercaptopurine | 100uM | 16h | CORO2A | 0.00 | 0.38 |
| HT29_10_GR_ChDB_0080 | 6-Mercaptopurine | 100uM | 16h | PCF11 | 0.00 | 0.45 |
| HT29_10_GR_ChDB_0080 | 6-Mercaptopurine | 100uM | 16h | GALNT6 | 0.00 | 0.44 |
| HT29_10_GR_ChDB_0081 | Temsirolimus | 10uM | 16h | DHRS3 | 1.00 | 2.07 |
| HT29_10_GR_ChDB_0081 | Temsirolimus | 10uM | 16h | NEU1 | 0.55 | 2.35 |
| HT29_10_GR_ChDB_0081 | Temsirolimus | 10uM | 16h | DKK1 | 0.00 | 2.07 |
| HT29_10_GR_ChDB_0081 | Temsirolimus | 10uM | 16h | SMAD3 | 0.00 | 2.12 |
| HT29_10_GR_ChDB_0081 | Temsirolimus | 10uM | 16h | FKBP14 | 0.00 | 0.49 |
| HT29_10_GR_ChDB_0081 | Temsirolimus | 10uM | 16h | VEGFA | 0.00 | 0.49 |
| HT29_10_GR_ChDB_0081 | Temsirolimus | 10uM | 16h | FOSL2 | 0.00 | 2.15 |
| HT29_10_GR_ChDB_0081 | Temsirolimus | 10uM | 16h | TRAPPC6A | 0.00 | 2.19 |
| HT29_10_GR_ChDB_0081 | Temsirolimus | 10uM | 16h | ARIH2 | 0.00 | 0.50 |
| HT29_10_GR_ChDB_0081 | Temsirolimus | 10uM | 16h | SKP2 | 0.00 | 0.48 |
| HT29_10_GR_ChDB_0081 | Temsirolimus | 10uM | 16h | ITGB6 | 0.00 | 2.26 |
| HT29_10_GR_ChDB_0081 | Temsirolimus | 10uM | 16h | DDIT4 | 0.00 | 0.49 |
| HT29_10_GR_ChDB_0081 | Temsirolimus | 10uM | 16h | EIF1 | 0.00 | 0.49 |
| HT29_10_GR_ChDB_0081 | Temsirolimus | 10uM | 16h | LAMA3 | 0.00 | 4.33 |
| HT29_10_GR_ChDB_0081 | Temsirolimus | 10uM | 16h | SERINC3 | 0.00 | 2.00 |
| HT29_10_GR_ChDB_0081 | Temsirolimus | 10uM | 16h | TSPAN1 | 0.00 | 2.04 |
| HT29_10_GR_ChDB_0081 | Temsirolimus | 10uM | 16h | ZC3HAV1 | 0.00 | 2.06 |
| HT29_10_GR_ChDB_0084 | Etoposide | 30uM | 16h | MYC | 1.00 | 0.34 |
| HT29_10_GR_ChDB_0084 | Etoposide | 30uM | 16h | IGF1R | 0.22 | 0.33 |
| HT29_10_GR_ChDB_0084 | Etoposide | 30uM | 16h | PTK2 | 0.19 | 0.41 |
| HT29_10_GR_ChDB_0084 | Etoposide | 30uM | 16h | EXT1 | 0.09 | 0.42 |
| HT29_10_GR_ChDB_0084 | Etoposide | 30uM | 16h | SOX4 | 0.07 | 0.28 |
| HT29_10_GR_ChDB_0084 | Etoposide | 30uM | 16h | PIK3R1 | 0.07 | 0.42 |
| HT29_10_GR_ChDB_0084 | Etoposide | 30uM | 16h | PPARG | 0.06 | 0.25 |
| HT29_10_GR_ChDB_0084 | Etoposide | 30uM | 16h | IL8 | 0.04 | 7.27 |
| HT29_10_GR_ChDB_0084 | Etoposide | 30uM | 16h | BCR | 0.04 | 0.48 |
| HT29_10_GR_ChDB_0084 | Etoposide | 30uM | 16h | IGFBP6 | 0.03 | 2.18 |
| HT29_10_GR_ChDB_0084 | Etoposide | 30uM | 16h | NRIP1 | 0.03 | 0.42 |
| HT29_10_GR_ChDB_0084 | Etoposide | 30uM | 16h | ADM | 0.03 | 2.25 |
| HT29_10_GR_ChDB_0084 | Etoposide | 30uM | 16h | CREB3 | 0.03 | 2.70 |
| HT29_10_GR_ChDB_0084 | Etoposide | 30uM | 16h | NRBP2 | 0.02 | 2.00 |
| HT29_10_GR_ChDB_0084 | Etoposide | 30uM | 16h | PLK2 | 0.02 | 3.52 |
| HT29_10_GR_ChDB_0084 | Etoposide | 30uM | 16h | RNF19B | 0.02 | 2.37 |
| HT29_10_GR_ChDB_0084 | Etoposide | 30uM | 16h | CTSL2 | 0.02 | 3.60 |
| HT29_10_GR_ChDB_0084 | Etoposide | 30uM | 16h | TIMP2 | 0.02 | 3.87 |
| HT29_10_GR_ChDB_0084 | Etoposide | 30uM | 16h | MXD1 | 0.02 | 3.04 |
| HT29_10_GR_ChDB_0084 | Etoposide | 30uM | 16h | ABCC3 | 0.02 | 0.46 |
| HT29_10_GR_ChDB_0084 | Etoposide | 30uM | 16h | TCF7L2 | 0.02 | 0.31 |
| HT29_10_GR_ChDB_0084 | Etoposide | 30uM | 16h | PKDCC | 0.02 | 0.31 |
| HT29_10_GR_ChDB_0084 | Etoposide | 30uM | 16h | BACH1 | 0.02 | 2.15 |
| HT29_10_GR_ChDB_0084 | Etoposide | 30uM | 16h | FERMT1 | 0.02 | 0.48 |
| HT29_10_GR_ChDB_0084 | Etoposide | 30uM | 16h | ADK | 0.02 | 0.28 |
| HT29_10_GR_ChDB_0084 | Etoposide | 30uM | 16h | CXCL16 | 0.02 | 2.45 |
| HT29_10_GR_ChDB_0084 | Etoposide | 30uM | 16h | MMP7 | 0.02 | 2.12 |
| HT29_10_GR_ChDB_0084 | Etoposide | 30uM | 16h | LCK | 0.01 | 2.20 |
| HT29_10_GR_ChDB_0084 | Etoposide | 30uM | 16h | CXCR4 | 0.01 | 0.46 |
| HT29_10_GR_ChDB_0084 | Etoposide | 30uM | 16h | ISG20 | 0.01 | 2.24 |
| HT29_10_GR_ChDB_0084 | Etoposide | 30uM | 16h | IL18 | 0.01 | 3.17 |
| HT29_10_GR_ChDB_0084 | Etoposide | 30uM | 16h | DCUN1D4 | 0.01 | 0.46 |
| HT29_10_GR_ChDB_0084 | Etoposide | 30uM | 16h | LGR5 | 0.01 | 0.29 |
| HT29_10_GR_ChDB_0084 | Etoposide | 30uM | 16h | XRCC4 | 0.01 | 0.47 |
| HT29_10_GR_ChDB_0084 | Etoposide | 30uM | 16h | PPARGC1B | 0.01 | 0.39 |
| HT29_10_GR_ChDB_0084 | Etoposide | 30uM | 16h | HMGCS2 | 0.01 | 0.28 |
| HT29_10_GR_ChDB_0084 | Etoposide | 30uM | 16h | KDELR3 | 0.01 | 2.71 |
| HT29_10_GR_ChDB_0084 | Etoposide | 30uM | 16h | DUSP4 | 0.01 | 2.05 |
| HT29_10_GR_ChDB_0084 | Etoposide | 30uM | 16h | CTBP2 | 0.01 | 0.37 |
| HT29_10_GR_ChDB_0084 | Etoposide | 30uM | 16h | FN1 | 0.01 | 2.82 |
| HT29_10_GR_ChDB_0084 | Etoposide | 30uM | 16h | GTF2F2 | 0.01 | 0.49 |
| HT29_10_GR_ChDB_0084 | Etoposide | 30uM | 16h | RND3 | 0.01 | 2.33 |
| HT29_10_GR_ChDB_0084 | Etoposide | 30uM | 16h | FDXR | 0.01 | 2.09 |
| HT29_10_GR_ChDB_0084 | Etoposide | 30uM | 16h | IL1R2 | 0.01 | 2.19 |
| HT29_10_GR_ChDB_0084 | Etoposide | 30uM | 16h | GADD45B | 0.01 | 2.94 |
| HT29_10_GR_ChDB_0084 | Etoposide | 30uM | 16h | S100A13 | 0.01 | 2.06 |
| HT29_10_GR_ChDB_0084 | Etoposide | 30uM | 16h | TRIM2 | 0.01 | 0.41 |
| HT29_10_GR_ChDB_0084 | Etoposide | 30uM | 16h | ATG4A | 0.01 | 2.20 |
| HT29_10_GR_ChDB_0084 | Etoposide | 30uM | 16h | PRKX | 0.01 | 0.47 |
| HT29_10_GR_ChDB_0084 | Etoposide | 30uM | 16h | FAT1 | 0.01 | 0.45 |
| HT29_10_GR_ChDB_0084 | Etoposide | 30uM | 16h | AKR1C3 | 0.01 | 2.81 |
| HT29_10_GR_ChDB_0084 | Etoposide | 30uM | 16h | P4HA2 | 0.01 | 2.07 |
| HT29_10_GR_ChDB_0084 | Etoposide | 30uM | 16h | STK39 | 0.01 | 0.39 |
| HT29_10_GR_ChDB_0084 | Etoposide | 30uM | 16h | PRKAR1A | 0.01 | 3.00 |
| HT29_10_GR_ChDB_0084 | Etoposide | 30uM | 16h | PMM2 | 0.01 | 0.42 |
| HT29_10_GR_ChDB_0084 | Etoposide | 30uM | 16h | BNIP3L | 0.01 | 2.64 |
| HT29_10_GR_ChDB_0084 | Etoposide | 30uM | 16h | TM7SF2 | 0.01 | 2.60 |
| HT29_10_GR_ChDB_0084 | Etoposide | 30uM | 16h | GNB5 | 0.01 | 2.29 |
| HT29_10_GR_ChDB_0084 | Etoposide | 30uM | 16h | PRSS23 | 0.01 | 2.80 |
| HT29_10_GR_ChDB_0084 | Etoposide | 30uM | 16h | CRCP | 0.01 | 0.30 |
| HT29_10_GR_ChDB_0084 | Etoposide | 30uM | 16h | LIMK2 | 0.01 | 0.48 |
| HT29_10_GR_ChDB_0084 | Etoposide | 30uM | 16h | CFTR | 0.00 | 0.42 |
| HT29_10_GR_ChDB_0084 | Etoposide | 30uM | 16h | TFPI | 0.00 | 0.44 |
| HT29_10_GR_ChDB_0084 | Etoposide | 30uM | 16h | MAP4K4 | 0.00 | 0.48 |
| HT29_10_GR_ChDB_0084 | Etoposide | 30uM | 16h | HIST1H2BK | 0.00 | 0.47 |
| HT29_10_GR_ChDB_0084 | Etoposide | 30uM | 16h | FOSL2 | 0.00 | 2.10 |
| HT29_10_GR_ChDB_0084 | Etoposide | 30uM | 16h | E2F5 | 0.00 | 0.44 |
| HT29_10_GR_ChDB_0084 | Etoposide | 30uM | 16h | BLVRA | 0.00 | 2.56 |
| HT29_10_GR_ChDB_0084 | Etoposide | 30uM | 16h | ATF6 | 0.00 | 0.45 |
| HT29_10_GR_ChDB_0084 | Etoposide | 30uM | 16h | MBNL1 | 0.00 | 0.36 |
| HT29_10_GR_ChDB_0084 | Etoposide | 30uM | 16h | ARL4C | 0.00 | 2.96 |
| HT29_10_GR_ChDB_0084 | Etoposide | 30uM | 16h | SHB | 0.00 | 0.43 |
| HT29_10_GR_ChDB_0084 | Etoposide | 30uM | 16h | TUBB2A | 0.00 | 3.06 |
| HT29_10_GR_ChDB_0084 | Etoposide | 30uM | 16h | TNIK | 0.00 | 0.45 |
| HT29_10_GR_ChDB_0084 | Etoposide | 30uM | 16h | PTEN | 0.00 | 0.48 |
| HT29_10_GR_ChDB_0084 | Etoposide | 30uM | 16h | FGFR3 | 0.00 | 2.11 |
| HT29_10_GR_ChDB_0084 | Etoposide | 30uM | 16h | PJA1 | 0.00 | 2.17 |
| HT29_10_GR_ChDB_0084 | Etoposide | 30uM | 16h | SH3GL1 | 0.00 | 0.47 |
| HT29_10_GR_ChDB_0084 | Etoposide | 30uM | 16h | NEU1 | 0.00 | 4.88 |
| HT29_10_GR_ChDB_0084 | Etoposide | 30uM | 16h | G3BP1 | 0.00 | 0.36 |
| HT29_10_GR_ChDB_0084 | Etoposide | 30uM | 16h | RBBP4 | 0.00 | 0.48 |
| HT29_10_GR_ChDB_0084 | Etoposide | 30uM | 16h | ELOVL6 | 0.00 | 0.41 |
| HT29_10_GR_ChDB_0084 | Etoposide | 30uM | 16h | PTPRK | 0.00 | 0.30 |
| HT29_10_GR_ChDB_0084 | Etoposide | 30uM | 16h | CAMK2D | 0.00 | 0.44 |
| HT29_10_GR_ChDB_0084 | Etoposide | 30uM | 16h | MRPL12 | 0.00 | 0.43 |
| HT29_10_GR_ChDB_0084 | Etoposide | 30uM | 16h | MPHOSPH9 | 0.00 | 0.47 |
| HT29_10_GR_ChDB_0084 | Etoposide | 30uM | 16h | HSPA2 | 0.00 | 3.56 |
| HT29_10_GR_ChDB_0084 | Etoposide | 30uM | 16h | PHF17 | 0.00 | 0.46 |
| HT29_10_GR_ChDB_0084 | Etoposide | 30uM | 16h | BIK | 0.00 | 2.32 |
| HT29_10_GR_ChDB_0084 | Etoposide | 30uM | 16h | TRIO | 0.00 | 0.39 |
| HT29_10_GR_ChDB_0084 | Etoposide | 30uM | 16h | DKK1 | 0.00 | 2.15 |
| HT29_10_GR_ChDB_0084 | Etoposide | 30uM | 16h | MAP3K5 | 0.00 | 0.43 |
| HT29_10_GR_ChDB_0084 | Etoposide | 30uM | 16h | F2R | 0.00 | 2.95 |
| HT29_10_GR_ChDB_0084 | Etoposide | 30uM | 16h | GNAQ | 0.00 | 0.44 |
| HT29_10_GR_ChDB_0084 | Etoposide | 30uM | 16h | OAT | 0.00 | 2.04 |
| HT29_10_GR_ChDB_0084 | Etoposide | 30uM | 16h | SGK1 | 0.00 | 3.05 |
| HT29_10_GR_ChDB_0084 | Etoposide | 30uM | 16h | ATRX | 0.00 | 0.43 |
| HT29_10_GR_ChDB_0084 | Etoposide | 30uM | 16h | ARG2 | 0.00 | 2.51 |
| HT29_10_GR_ChDB_0084 | Etoposide | 30uM | 16h | PDXK | 0.00 | 0.42 |
| HT29_10_GR_ChDB_0084 | Etoposide | 30uM | 16h | LGALS8 | 0.00 | 2.67 |
| HT29_10_GR_ChDB_0084 | Etoposide | 30uM | 16h | BMP4 | 0.00 | 0.43 |
| HT29_10_GR_ChDB_0084 | Etoposide | 30uM | 16h | TCIRG1 | 0.00 | 2.12 |
| HT29_10_GR_ChDB_0084 | Etoposide | 30uM | 16h | HSD17B2 | 0.00 | 0.37 |
| HT29_10_GR_ChDB_0084 | Etoposide | 30uM | 16h | NAGK | 0.00 | 2.82 |
| HT29_10_GR_ChDB_0084 | Etoposide | 30uM | 16h | GTF2H2 | 0.00 | 0.44 |
| HT29_10_GR_ChDB_0084 | Etoposide | 30uM | 16h | MLLT10 | 0.00 | 0.50 |
| HT29_10_GR_ChDB_0084 | Etoposide | 30uM | 16h | AKR1C1 | 0.00 | 3.96 |
| HT29_10_GR_ChDB_0084 | Etoposide | 30uM | 16h | NAT1 | 0.00 | 2.96 |
| HT29_10_GR_ChDB_0084 | Etoposide | 30uM | 16h | PSRC1 | 0.00 | 0.43 |
| HT29_10_GR_ChDB_0084 | Etoposide | 30uM | 16h | GMDS | 0.00 | 0.26 |
| HT29_10_GR_ChDB_0084 | Etoposide | 30uM | 16h | SFN | 0.00 | 2.42 |
| HT29_10_GR_ChDB_0084 | Etoposide | 30uM | 16h | RARRES3 | 0.00 | 3.27 |
| HT29_10_GR_ChDB_0084 | Etoposide | 30uM | 16h | RFX5 | 0.00 | 2.22 |
| HT29_10_GR_ChDB_0084 | Etoposide | 30uM | 16h | TSEN2 | 0.00 | 0.47 |
| HT29_10_GR_ChDB_0084 | Etoposide | 30uM | 16h | ZFX | 0.00 | 0.37 |
| HT29_10_GR_ChDB_0084 | Etoposide | 30uM | 16h | KLF4 | 0.00 | 2.33 |
| HT29_10_GR_ChDB_0084 | Etoposide | 30uM | 16h | SRD5A1 | 0.00 | 2.56 |
| HT29_10_GR_ChDB_0084 | Etoposide | 30uM | 16h | ABCC4 | 0.00 | 0.45 |
| HT29_10_GR_ChDB_0084 | Etoposide | 30uM | 16h | STAT2 | 0.00 | 2.40 |
| HT29_10_GR_ChDB_0084 | Etoposide | 30uM | 16h | DDX10 | 0.00 | 0.39 |
| HT29_10_GR_ChDB_0084 | Etoposide | 30uM | 16h | SMURF1 | 0.00 | 0.50 |
| HT29_10_GR_ChDB_0084 | Etoposide | 30uM | 16h | SLC22A18 | 0.00 | 2.75 |
| HT29_10_GR_ChDB_0084 | Etoposide | 30uM | 16h | TCF12 | 0.00 | 0.36 |
| HT29_10_GR_ChDB_0084 | Etoposide | 30uM | 16h | PLAUR | 0.00 | 2.98 |
| HT29_10_GR_ChDB_0084 | Etoposide | 30uM | 16h | RAB27A | 0.00 | 2.01 |
| HT29_10_GR_ChDB_0084 | Etoposide | 30uM | 16h | ETV6 | 0.00 | 0.43 |
| HT29_10_GR_ChDB_0084 | Etoposide | 30uM | 16h | SULT1A2 | 0.00 | 2.19 |
| HT29_10_GR_ChDB_0084 | Etoposide | 30uM | 16h | NOTCH2NL | 0.00 | 0.44 |
| HT29_10_GR_ChDB_0084 | Etoposide | 30uM | 16h | CAV1 | 0.00 | 3.52 |
| HT29_10_GR_ChDB_0084 | Etoposide | 30uM | 16h | SRSF8 | 0.00 | 0.49 |
| HT29_10_GR_ChDB_0084 | Etoposide | 30uM | 16h | CDK5 | 0.00 | 2.29 |
| HT29_10_GR_ChDB_0084 | Etoposide | 30uM | 16h | BAD | 0.00 | 2.78 |
| HT29_10_GR_ChDB_0084 | Etoposide | 30uM | 16h | RRAGC | 0.00 | 2.39 |
| HT29_10_GR_ChDB_0084 | Etoposide | 30uM | 16h | POLA1 | 0.00 | 0.42 |
| HT29_10_GR_ChDB_0084 | Etoposide | 30uM | 16h | RHOD | 0.00 | 2.42 |
| HT29_10_GR_ChDB_0084 | Etoposide | 30uM | 16h | IFIH1 | 0.00 | 2.31 |
| HT29_10_GR_ChDB_0084 | Etoposide | 30uM | 16h | APP | 0.00 | 0.37 |
| HT29_10_GR_ChDB_0084 | Etoposide | 30uM | 16h | MLLT3 | 0.00 | 0.50 |
| HT29_10_GR_ChDB_0084 | Etoposide | 30uM | 16h | GNA12 | 0.00 | 0.50 |
| HT29_10_GR_ChDB_0084 | Etoposide | 30uM | 16h | THADA | 0.00 | 0.46 |
| HT29_10_GR_ChDB_0084 | Etoposide | 30uM | 16h | DFFA | 0.00 | 0.43 |
| HT29_10_GR_ChDB_0084 | Etoposide | 30uM | 16h | MFHAS1 | 0.00 | 0.35 |
| HT29_10_GR_ChDB_0084 | Etoposide | 30uM | 16h | PRMT3 | 0.00 | 0.48 |
| HT29_10_GR_ChDB_0084 | Etoposide | 30uM | 16h | ETS2 | 0.00 | 0.42 |
| HT29_10_GR_ChDB_0084 | Etoposide | 30uM | 16h | ITCH | 0.00 | 0.44 |
| HT29_10_GR_ChDB_0084 | Etoposide | 30uM | 16h | MTAP | 0.00 | 0.41 |
| HT29_10_GR_ChDB_0084 | Etoposide | 30uM | 16h | PHYH | 0.00 | 2.69 |
| HT29_10_GR_ChDB_0084 | Etoposide | 30uM | 16h | HPGD | 0.00 | 2.55 |
| HT29_10_GR_ChDB_0084 | Etoposide | 30uM | 16h | LGR4 | 0.00 | 0.50 |
| HT29_10_GR_ChDB_0084 | Etoposide | 30uM | 16h | SMYD3 | 0.00 | 0.49 |
| HT29_10_GR_ChDB_0084 | Etoposide | 30uM | 16h | TNFRSF11A | 0.00 | 0.48 |
| HT29_10_GR_ChDB_0084 | Etoposide | 30uM | 16h | PDS5B | 0.00 | 0.40 |
| HT29_10_GR_ChDB_0084 | Etoposide | 30uM | 16h | MAP7 | 0.00 | 0.50 |
| HT29_10_GR_ChDB_0084 | Etoposide | 30uM | 16h | RXRA | 0.00 | 0.38 |
| HT29_10_GR_ChDB_0084 | Etoposide | 30uM | 16h | FAF1 | 0.00 | 0.37 |
| HT29_10_GR_ChDB_0084 | Etoposide | 30uM | 16h | BCCIP | 0.00 | 0.42 |
| HT29_10_GR_ChDB_0084 | Etoposide | 30uM | 16h | SULT1A1 | 0.00 | 2.85 |
| HT29_10_GR_ChDB_0084 | Etoposide | 30uM | 16h | DICER1 | 0.00 | 0.50 |
| HT29_10_GR_ChDB_0084 | Etoposide | 30uM | 16h | DNAJA3 | 0.00 | 0.48 |
| HT29_10_GR_ChDB_0084 | Etoposide | 30uM | 16h | ATP6V1D | 0.00 | 2.13 |
| HT29_11_GR_ChDB_0088 | MLN-4924 | 10uM | 6h | HBEGF | 1.00 | 2.15 |
| HT29_11_GR_ChDB_0092 | SB218078 | 3uM | 6h | JUN | 1.00 | 0.40 |
| HT29_11_GR_ChDB_0092 | SB218078 | 3uM | 6h | PRKAA1 | 0.12 | 2.22 |
| HT29_11_GR_ChDB_0092 | SB218078 | 3uM | 6h | HMGCL | 0.05 | 2.52 |
| HT29_11_GR_ChDB_0092 | SB218078 | 3uM | 6h | IRS1 | 0.05 | 0.45 |
| HT29_11_GR_ChDB_0092 | SB218078 | 3uM | 6h | THBS1 | 0.04 | 0.39 |
| HT29_11_GR_ChDB_0092 | SB218078 | 3uM | 6h | CXADR | 0.04 | 2.15 |
| HT29_11_GR_ChDB_0092 | SB218078 | 3uM | 6h | TAF15 | 0.03 | 3.33 |
| HT29_11_GR_ChDB_0092 | SB218078 | 3uM | 6h | SLC1A1 | 0.02 | 2.18 |
| HT29_11_GR_ChDB_0092 | SB218078 | 3uM | 6h | CFLAR | 0.01 | 0.45 |
| HT29_11_GR_ChDB_0092 | SB218078 | 3uM | 6h | ASCL2 | 0.01 | 2.55 |
| HT29_11_GR_ChDB_0092 | SB218078 | 3uM | 6h | RARA | 0.01 | 2.51 |
| HT29_11_GR_ChDB_0092 | SB218078 | 3uM | 6h | PHF15 | 0.01 | 0.50 |
| HT29_11_GR_ChDB_0092 | SB218078 | 3uM | 6h | RNMT | 0.00 | 2.28 |
| HT29_11_GR_ChDB_0092 | SB218078 | 3uM | 6h | CLK3 | 0.00 | 2.93 |
| HT29_11_GR_ChDB_0092 | SB218078 | 3uM | 6h | DCLRE1B | 0.00 | 2.37 |
| HT29_11_GR_ChDB_0092 | SB218078 | 3uM | 6h | NCOA3 | 0.00 | 0.46 |
| HT29_11_GR_ChDB_0092 | SB218078 | 3uM | 6h | HNF1B | 0.00 | 0.48 |
| HT29_11_GR_ChDB_0092 | SB218078 | 3uM | 6h | RPL35A | 0.00 | 2.07 |
| HT29_11_GR_ChDB_0092 | SB218078 | 3uM | 6h | ZNF398 | 0.00 | 2.02 |
| HT29_11_GR_ChDB_0092 | SB218078 | 3uM | 6h | CLOCK | 0.00 | 0.41 |
| HT29_11_GR_ChDB_0092 | SB218078 | 3uM | 6h | FGFR2 | 0.00 | 0.45 |
| HT29_11_GR_ChDB_0092 | SB218078 | 3uM | 6h | SGK1 | 0.00 | 0.44 |
| HT29_11_GR_ChDB_0092 | SB218078 | 3uM | 6h | SLK | 0.00 | 0.41 |
| HT29_11_GR_ChDB_0092 | SB218078 | 3uM | 6h | HES1 | 0.00 | 0.48 |
| HT29_11_GR_ChDB_0092 | SB218078 | 3uM | 6h | AZI2 | 0.00 | 0.47 |
| HT29_11_GR_ChDB_0092 | SB218078 | 3uM | 6h | EGFR | 0.00 | 0.36 |
| HT29_11_GR_ChDB_0092 | SB218078 | 3uM | 6h | ZNF581 | 0.00 | 2.50 |
| HT29_11_GR_ChDB_0092 | SB218078 | 3uM | 6h | SOCS2 | 0.00 | 2.14 |
| HT29_11_GR_ChDB_0092 | SB218078 | 3uM | 6h | P4HA2 | 0.00 | 0.49 |
| HT29_11_GR_ChDB_0092 | SB218078 | 3uM | 6h | FOSL2 | 0.00 | 0.48 |
| HT29_11_GR_ChDB_0092 | SB218078 | 3uM | 6h | IGF1R | 0.00 | 0.47 |
| HT29_11_GR_ChDB_0092 | SB218078 | 3uM | 6h | PPAP2A | 0.00 | 0.42 |
| HT29_11_GR_ChDB_0092 | SB218078 | 3uM | 6h | EGR1 | 0.00 | 2.58 |
| HT29_11_GR_ChDB_0092 | SB218078 | 3uM | 6h | PNN | 0.00 | 0.32 |
| HT29_11_GR_ChDB_0092 | SB218078 | 3uM | 6h | PDS5B | 0.00 | 0.46 |
| HT29_11_GR_ChDB_0092 | SB218078 | 3uM | 6h | PARP2 | 0.00 | 0.43 |
| HT29_11_GR_ChDB_0092 | SB218078 | 3uM | 6h | PAPOLA | 0.00 | 0.34 |
| HT29_11_GR_ChDB_0092 | SB218078 | 3uM | 6h | TIA1 | 0.00 | 0.45 |
| HT29_11_GR_ChDB_0092 | SB218078 | 3uM | 6h | GEMIN2 | 0.00 | 0.47 |
| HT29_11_GR_ChDB_0092 | SB218078 | 3uM | 6h | EXT1 | 0.00 | 0.35 |
| HT29_11_GR_ChDB_0092 | SB218078 | 3uM | 6h | CCDC92 | 0.00 | 2.20 |
| HT29_11_GR_ChDB_0092 | SB218078 | 3uM | 6h | MAT2A | 0.00 | 0.42 |
| HT29_11_GR_ChDB_0092 | SB218078 | 3uM | 6h | RRM2 | 0.00 | 0.33 |
| HT29_11_GR_ChDB_0092 | SB218078 | 3uM | 6h | HNRNPDL | 0.00 | 0.46 |
| HT29_11_GR_ChDB_0092 | SB218078 | 3uM | 6h | ZFR | 0.00 | 2.23 |
| HT29_11_GR_ChDB_0092 | SB218078 | 3uM | 6h | PSIP1 | 0.00 | 0.46 |
| HT29_11_GR_ChDB_0092 | SB218078 | 3uM | 6h | MRPS12 | 0.00 | 0.49 |
| HT29_11_GR_ChDB_0092 | SB218078 | 3uM | 6h | HMGCS1 | 0.00 | 0.35 |
| HT29_11_GR_ChDB_0092 | SB218078 | 3uM | 6h | WTAP | 0.00 | 0.35 |
| HT29_11_GR_ChDB_0092 | SB218078 | 3uM | 6h | CSNK1A1 | 0.00 | 0.47 |
| HT29_11_GR_ChDB_0092 | SB218078 | 3uM | 6h | PDS5A | 0.00 | 0.46 |
| HT29_11_GR_ChDB_0092 | SB218078 | 3uM | 6h | UTP18 | 0.00 | 0.34 |
| HT29_11_GR_ChDB_0092 | SB218078 | 3uM | 6h | RAD51C | 0.00 | 0.48 |
| HT29_11_GR_ChDB_0092 | SB218078 | 3uM | 6h | ATP11B | 0.00 | 0.40 |
| HT29_11_GR_ChDB_0092 | SB218078 | 3uM | 6h | JMJD6 | 0.00 | 0.38 |
| HT29_11_GR_ChDB_0092 | SB218078 | 3uM | 6h | GIGYF2 | 0.00 | 2.84 |
| HT29_11_GR_ChDB_0092 | SB218078 | 3uM | 6h | SF3B3 | 0.00 | 2.34 |
| HT29_11_GR_ChDB_0092 | SB218078 | 3uM | 6h | EMC1 | 0.00 | 2.62 |
| HT29_11_GR_ChDB_0092 | SB218078 | 3uM | 6h | LGALS8 | 0.00 | 2.09 |
| HT29_11_GR_ChDB_0092 | SB218078 | 3uM | 6h | NSMCE4A | 0.00 | 0.42 |
| HT29_11_GR_ChDB_0092 | SB218078 | 3uM | 6h | KLHL9 | 0.00 | 0.46 |
| HT29_11_GR_ChDB_0092 | SB218078 | 3uM | 6h | TRMT11 | 0.00 | 0.39 |
| HT29_11_GR_ChDB_0092 | SB218078 | 3uM | 6h | ATP1B1 | 0.00 | 0.48 |
| HT29_11_GR_ChDB_0092 | SB218078 | 3uM | 6h | SKP2 | 0.00 | 0.35 |
| HT29_11_GR_ChDB_0092 | SB218078 | 3uM | 6h | GABPB1 | 0.00 | 0.37 |
| HT29_11_GR_ChDB_0092 | SB218078 | 3uM | 6h | ORC5 | 0.00 | 0.49 |
| HT29_11_GR_ChDB_0092 | SB218078 | 3uM | 6h | FASTKD2 | 0.00 | 0.48 |
| HT29_11_GR_ChDB_0092 | SB218078 | 3uM | 6h | PAFAH1B1 | 0.00 | 0.41 |
| HT29_11_GR_ChDB_0092 | SB218078 | 3uM | 6h | CREB1 | 0.00 | 0.35 |
| HT29_11_GR_ChDB_0092 | SB218078 | 3uM | 6h | MED13L | 0.00 | 0.44 |
| HT29_11_GR_ChDB_0092 | SB218078 | 3uM | 6h | SNAPC3 | 0.00 | 0.45 |
| HT29_11_GR_ChDB_0092 | SB218078 | 3uM | 6h | CUL3 | 0.00 | 0.47 |
| HT29_11_GR_ChDB_0092 | SB218078 | 3uM | 6h | NFIB | 0.00 | 0.49 |
| HT29_11_GR_ChDB_0092 | SB218078 | 3uM | 6h | KPNA1 | 0.00 | 0.44 |
| HT29_11_GR_ChDB_0092 | SB218078 | 3uM | 6h | YARS2 | 0.00 | 0.22 |
| HT29_11_GR_ChDB_0092 | SB218078 | 3uM | 6h | ITSN1 | 0.00 | 0.48 |
| HT29_11_GR_ChDB_0092 | SB218078 | 3uM | 6h | IL1RN | 0.00 | 0.31 |
| HT29_11_GR_ChDB_0092 | SB218078 | 3uM | 6h | RFC3 | 0.00 | 0.46 |
| HT29_11_GR_ChDB_0092 | SB218078 | 3uM | 6h | UBE3B | 0.00 | 2.09 |
| HT29_11_GR_ChDB_0092 | SB218078 | 3uM | 6h | FAM57A | 0.00 | 2.10 |
| HT29_11_GR_ChDB_0092 | SB218078 | 3uM | 6h | TMPO | 0.00 | 0.44 |
| HT29_11_GR_ChDB_0092 | SB218078 | 3uM | 6h | BLZF1 | 0.00 | 0.32 |
| HT29_11_GR_ChDB_0092 | SB218078 | 3uM | 6h | U2SURP | 0.00 | 0.38 |
| HT29_11_GR_ChDB_0092 | SB218078 | 3uM | 6h | RAI14 | 0.00 | 0.46 |
| HT29_11_GR_ChDB_0092 | SB218078 | 3uM | 6h | PAIP1 | 0.00 | 0.44 |
| HT29_11_GR_ChDB_0092 | SB218078 | 3uM | 6h | SYNCRIP | 0.00 | 0.28 |
| HT29_11_GR_ChDB_0092 | SB218078 | 3uM | 6h | FBXO3 | 0.00 | 0.49 |
| HT29_11_GR_ChDB_0092 | SB218078 | 3uM | 6h | INSIG1 | 0.00 | 0.43 |
| HT29_11_GR_ChDB_0092 | SB218078 | 3uM | 6h | METTL1 | 0.00 | 0.29 |
| HT29_11_GR_ChDB_0092 | SB218078 | 3uM | 6h | PRKACB | 0.00 | 0.43 |
| HT29_11_GR_ChDB_0092 | SB218078 | 3uM | 6h | MLLT3 | 0.00 | 0.42 |
| HT29_11_GR_ChDB_0092 | SB218078 | 3uM | 6h | TRIOBP | 0.00 | 2.20 |
| HT29_11_GR_ChDB_0092 | SB218078 | 3uM | 6h | SNAPC4 | 0.00 | 2.82 |
| HT29_11_GR_ChDB_0092 | SB218078 | 3uM | 6h | SMCR7L | 0.00 | 2.04 |
| HT29_11_GR_ChDB_0092 | SB218078 | 3uM | 6h | HBE1 | 0.00 | 2.50 |
| HT29_11_GR_ChDB_0092 | SB218078 | 3uM | 6h | GLS | 0.00 | 3.16 |
| HT29_11_GR_ChDB_0092 | SB218078 | 3uM | 6h | BBS1 | 0.00 | 2.36 |
| HT29_11_GR_ChDB_0092 | SB218078 | 3uM | 6h | LPGAT1 | 0.00 | 0.44 |
| HT29_11_GR_ChDB_0092 | SB218078 | 3uM | 6h | TNIK | 0.00 | 0.49 |
| HT29_11_GR_ChDB_0092 | SB218078 | 3uM | 6h | OIP5 | 0.00 | 0.45 |
| HT29_11_GR_ChDB_0092 | SB218078 | 3uM | 6h | FAM49B | 0.00 | 0.44 |
| HT29_11_GR_ChDB_0092 | SB218078 | 3uM | 6h | CD58 | 0.00 | 0.48 |
| HT29_11_GR_ChDB_0092 | SB218078 | 3uM | 6h | CHMP2B | 0.00 | 0.39 |
| HT29_11_GR_ChDB_0092 | SB218078 | 3uM | 6h | PGM3 | 0.00 | 0.42 |
| HT29_11_GR_ChDB_0092 | SB218078 | 3uM | 6h | KAT6B | 0.00 | 0.49 |
| HT29_11_GR_ChDB_0092 | SB218078 | 3uM | 6h | DICER1 | 0.00 | 0.48 |
| HT29_11_GR_ChDB_0092 | SB218078 | 3uM | 6h | CDC42EP4 | 0.00 | 0.48 |
| HT29_11_GR_ChDB_0092 | SB218078 | 3uM | 6h | TUG1 | 0.00 | 0.40 |
| HT29_11_GR_ChDB_0092 | SB218078 | 3uM | 6h | SUPT20H | 0.00 | 0.46 |
| HT29_11_GR_ChDB_0092 | SB218078 | 3uM | 6h | G3BP1 | 0.00 | 0.39 |
| HT29_11_GR_ChDB_0092 | SB218078 | 3uM | 6h | DDX3X | 0.00 | 0.35 |
| HT29_11_GR_ChDB_0092 | SB218078 | 3uM | 6h | INTS3 | 0.00 | 0.39 |
| HT29_11_GR_ChDB_0092 | SB218078 | 3uM | 6h | WBP4 | 0.00 | 0.46 |
| HT29_11_GR_ChDB_0092 | SB218078 | 3uM | 6h | CASP7 | 0.00 | 0.39 |
| HT29_11_GR_ChDB_0092 | SB218078 | 3uM | 6h | RBM39 | 0.00 | 0.45 |
| HT29_11_GR_ChDB_0092 | SB218078 | 3uM | 6h | SRSF3 | 0.00 | 0.44 |
| HT29_11_GR_ChDB_0092 | SB218078 | 3uM | 6h | OSER1 | 0.00 | 0.49 |
| HT29_11_GR_ChDB_0092 | SB218078 | 3uM | 6h | CHIC2 | 0.00 | 0.48 |
| HT29_11_GR_ChDB_0092 | SB218078 | 3uM | 6h | CYP20A1 | 0.00 | 0.47 |
| HT29_11_GR_ChDB_0092 | SB218078 | 3uM | 6h | NUSAP1 | 0.00 | 0.42 |
| HT29_11_GR_ChDB_0092 | SB218078 | 3uM | 6h | GAS2L1 | 0.00 | 0.39 |
| HT29_11_GR_ChDB_0092 | SB218078 | 3uM | 6h | SEC14L1 | 0.00 | 0.48 |
| HT29_11_GR_ChDB_0092 | SB218078 | 3uM | 6h | ATG12 | 0.00 | 2.15 |
| HT29_11_GR_ChDB_0092 | SB218078 | 3uM | 6h | PDXK | 0.00 | 2.91 |
| HT29_11_GR_ChDB_0092 | SB218078 | 3uM | 6h | SH3BP4 | 0.00 | 2.01 |
| HT29_11_GR_ChDB_0092 | SB218078 | 3uM | 6h | PAAF1 | 0.00 | 2.11 |
| HT29_11_GR_ChDB_0092 | SB218078 | 3uM | 6h | ZKSCAN1 | 0.00 | 2.75 |
| HT29_11_GR_ChDB_0092 | SB218078 | 3uM | 6h | TTC13 | 0.00 | 2.42 |
| HT29_11_GR_ChDB_0092 | SB218078 | 3uM | 6h | HNRNPD | 0.00 | 3.08 |
| HT29_11_GR_ChDB_0092 | SB218078 | 3uM | 6h | RB1CC1 | 0.00 | 0.18 |
| HT29_11_GR_ChDB_0092 | SB218078 | 3uM | 6h | SRSF6 | 0.00 | 0.50 |
| HT29_11_GR_ChDB_0092 | SB218078 | 3uM | 6h | TMEM41B | 0.00 | 0.47 |
| HT29_11_GR_ChDB_0092 | SB218078 | 3uM | 6h | FUBP1 | 0.00 | 0.38 |
| HT29_11_GR_ChDB_0092 | SB218078 | 3uM | 6h | RNFT1 | 0.00 | 0.42 |
| HT29_11_GR_ChDB_0092 | SB218078 | 3uM | 6h | PDE3B | 0.00 | 0.37 |
| HT29_11_GR_ChDB_0092 | SB218078 | 3uM | 6h | CTPS1 | 0.00 | 0.40 |
| HT29_11_GR_ChDB_0092 | SB218078 | 3uM | 6h | RGS2 | 0.00 | 0.20 |
| HT29_11_GR_ChDB_0092 | SB218078 | 3uM | 6h | SWAP70 | 0.00 | 0.41 |
| HT29_11_GR_ChDB_0092 | SB218078 | 3uM | 6h | NEDD4L | 0.00 | 0.50 |
| HT29_11_GR_ChDB_0092 | SB218078 | 3uM | 6h | PARD3 | 0.00 | 0.47 |
| HT29_11_GR_ChDB_0092 | SB218078 | 3uM | 6h | UBL3 | 0.00 | 0.47 |
| HT29_11_GR_ChDB_0092 | SB218078 | 3uM | 6h | RAB11FIP2 | 0.00 | 0.39 |
| HT29_11_GR_ChDB_0092 | SB218078 | 3uM | 6h | PEX3 | 0.00 | 0.49 |
| HT29_11_GR_ChDB_0092 | SB218078 | 3uM | 6h | USP48 | 0.00 | 0.38 |
| HT29_11_GR_ChDB_0092 | SB218078 | 3uM | 6h | PPP2R2A | 0.00 | 0.47 |
| HT29_11_GR_ChDB_0092 | SB218078 | 3uM | 6h | RBM12 | 0.00 | 0.47 |
| HT29_11_GR_ChDB_0092 | SB218078 | 3uM | 6h | NBPF1 | 0.00 | 0.42 |
| HT29_11_GR_ChDB_0092 | SB218078 | 3uM | 6h | NIPBL | 0.00 | 0.44 |
| HT29_11_GR_ChDB_0092 | SB218078 | 3uM | 6h | PRKCI | 0.00 | 0.48 |
| HT29_11_GR_ChDB_0092 | SB218078 | 3uM | 6h | NREP | 0.00 | 0.42 |
| HT29_11_GR_ChDB_0092 | SB218078 | 3uM | 6h | ACSL3 | 0.00 | 0.44 |
| HT29_11_GR_ChDB_0092 | SB218078 | 3uM | 6h | ALDH1A3 | 0.00 | 0.27 |
| HT29_11_GR_ChDB_0092 | SB218078 | 3uM | 6h | MTX2 | 0.00 | 0.47 |
| HT29_11_GR_ChDB_0092 | SB218078 | 3uM | 6h | DST | 0.00 | 0.47 |
| HT29_11_GR_ChDB_0092 | SB218078 | 3uM | 6h | CDR2L | 0.00 | 0.46 |
| HT29_11_GR_ChDB_0092 | SB218078 | 3uM | 6h | TTC27 | 0.00 | 0.47 |
| HT29_11_GR_ChDB_0092 | SB218078 | 3uM | 6h | KIF20A | 0.00 | 0.43 |
| HT29_11_GR_ChDB_0092 | SB218078 | 3uM | 6h | ZNF586 | 0.00 | 0.35 |
| HT29_11_GR_ChDB_0092 | SB218078 | 3uM | 6h | PLEKHA5 | 0.00 | 0.50 |
| HT29_11_GR_ChDB_0092 | SB218078 | 3uM | 6h | MOB4 | 0.00 | 0.47 |
| HT29_11_GR_ChDB_0092 | SB218078 | 3uM | 6h | ZNF518A | 0.00 | 0.33 |
| HT29_11_GR_ChDB_0092 | SB218078 | 3uM | 6h | TGFBR1 | 0.00 | 0.46 |
| HT29_11_GR_ChDB_0092 | SB218078 | 3uM | 6h | BRCA1 | 0.00 | 0.37 |
| HT29_11_GR_ChDB_0092 | SB218078 | 3uM | 6h | MT1G | 0.00 | 3.47 |
| HT29_11_GR_ChDB_0092 | SB218078 | 3uM | 6h | CAMLG | 0.00 | 2.14 |
| HT29_11_GR_ChDB_0092 | SB218078 | 3uM | 6h | ELAVL1 | 0.00 | 2.40 |
| HT29_11_GR_ChDB_0092 | SB218078 | 3uM | 6h | CRK | 0.00 | 2.16 |
| HT29_11_GR_ChDB_0092 | SB218078 | 3uM | 6h | MT1F | 0.00 | 5.08 |
| HT29_11_GR_ChDB_0092 | SB218078 | 3uM | 6h | CD47 | 0.00 | 2.25 |
| HT29_11_GR_ChDB_0092 | SB218078 | 3uM | 6h | LRPPRC | 0.00 | 2.04 |
| HT29_11_GR_ChDB_0092 | SB218078 | 3uM | 6h | ANKRD12 | 0.00 | 2.72 |
| HT29_11_GR_ChDB_0092 | SB218078 | 3uM | 6h | STAU2 | 0.00 | 0.49 |
| HT29_11_GR_ChDB_0092 | SB218078 | 3uM | 6h | MNAT1 | 0.00 | 0.33 |
| HT29_11_GR_ChDB_0092 | SB218078 | 3uM | 6h | SKIL | 0.00 | 0.49 |
| HT29_11_GR_ChDB_0092 | SB218078 | 3uM | 6h | NDUFAF1 | 0.00 | 0.50 |
| HT29_11_GR_ChDB_0092 | SB218078 | 3uM | 6h | TIPIN | 0.00 | 0.48 |
| HT29_11_GR_ChDB_0092 | SB218078 | 3uM | 6h | CENPI | 0.00 | 0.47 |
| HT29_11_GR_ChDB_0092 | SB218078 | 3uM | 6h | GTPBP8 | 0.00 | 0.44 |
| HT29_11_GR_ChDB_0092 | SB218078 | 3uM | 6h | RBL2 | 0.00 | 0.48 |
| HT29_11_GR_ChDB_0092 | SB218078 | 3uM | 6h | TSPAN14 | 0.00 | 0.48 |
| HT29_11_GR_ChDB_0092 | SB218078 | 3uM | 6h | NLK | 0.00 | 0.37 |
| HT29_11_GR_ChDB_0092 | SB218078 | 3uM | 6h | HERPUD1 | 0.00 | 0.43 |
| HT29_11_GR_ChDB_0092 | SB218078 | 3uM | 6h | ETV5 | 0.00 | 0.47 |
| HT29_11_GR_ChDB_0092 | SB218078 | 3uM | 6h | RYBP | 0.00 | 0.49 |
| HT29_11_GR_ChDB_0092 | SB218078 | 3uM | 6h | MED4 | 0.00 | 0.33 |
| HT29_11_GR_ChDB_0092 | SB218078 | 3uM | 6h | EXOC5 | 0.00 | 0.43 |
| HT29_11_GR_ChDB_0092 | SB218078 | 3uM | 6h | INTS8 | 0.00 | 0.42 |
| HT29_11_GR_ChDB_0092 | SB218078 | 3uM | 6h | NOL3 | 0.00 | 0.42 |
| HT29_11_GR_ChDB_0092 | SB218078 | 3uM | 6h | CDC14B | 0.00 | 0.44 |
| HT29_11_GR_ChDB_0092 | SB218078 | 3uM | 6h | CDC23 | 0.00 | 0.24 |
| HT29_11_GR_ChDB_0092 | SB218078 | 3uM | 6h | SLC35C2 | 0.00 | 0.35 |
| HT29_11_GR_ChDB_0092 | SB218078 | 3uM | 6h | IVNS1ABP | 0.00 | 0.37 |
| HT29_11_GR_ChDB_0092 | SB218078 | 3uM | 6h | ARIH2 | 0.00 | 0.49 |
| HT29_11_GR_ChDB_0092 | SB218078 | 3uM | 6h | FUT4 | 0.00 | 0.50 |
| HT29_11_GR_ChDB_0092 | SB218078 | 3uM | 6h | EIF1 | 0.00 | 2.60 |
| HT29_11_GR_ChDB_0092 | SB218078 | 3uM | 6h | PTK7 | 0.00 | 2.30 |
| HT29_11_GR_ChDB_0092 | SB218078 | 3uM | 6h | RNF114 | 0.00 | 2.32 |
| HT29_11_GR_ChDB_0092 | SB218078 | 3uM | 6h | PCBP2 | 0.00 | 2.27 |
| HT29_11_GR_ChDB_0092 | SB218078 | 3uM | 6h | KPNA4 | 0.00 | 2.52 |
| HT29_11_GR_ChDB_0092 | SB218078 | 3uM | 6h | LRCH3 | 0.00 | 2.12 |
| HT29_11_GR_ChDB_0092 | SB218078 | 3uM | 6h | SLC25A38 | 0.00 | 2.03 |
| HT29_11_GR_ChDB_0092 | SB218078 | 3uM | 6h | CCNB1IP1 | 0.00 | 2.04 |
| HT29_11_GR_ChDB_0092 | SB218078 | 3uM | 6h | PHLDA1 | 0.00 | 2.05 |
| HT29_11_GR_ChDB_0092 | SB218078 | 3uM | 6h | RWDD2B | 0.00 | 2.06 |
| HT29_11_GR_ChDB_0092 | SB218078 | 3uM | 6h | RPL31 | 0.00 | 3.35 |
| HT29_11_GR_ChDB_0092 | SB218078 | 3uM | 6h | SMIM7 | 0.00 | 3.00 |
| HT29_11_GR_ChDB_0092 | SB218078 | 3uM | 6h | C6orf48 | 0.00 | 2.04 |
| HT29_11_GR_ChDB_0092 | SB218078 | 3uM | 6h | ATXN2 | 0.00 | 2.05 |
| HT29_11_GR_ChDB_0092 | SB218078 | 3uM | 6h | CCNT1 | 0.00 | 2.50 |
| HT29_11_GR_ChDB_0092 | SB218078 | 3uM | 6h | RBM3 | 0.00 | 2.04 |
| HT29_11_GR_ChDB_0092 | SB218078 | 3uM | 6h | ZNF292 | 0.00 | 2.04 |
| HT29_11_GR_ChDB_0092 | SB218078 | 3uM | 6h | KBTBD2 | 0.00 | 2.41 |
| HT29_11_GR_ChDB_0092 | SB218078 | 3uM | 6h | SLC30A1 | 0.00 | 4.03 |
| HT29_11_GR_ChDB_0092 | SB218078 | 3uM | 6h | ZNF23 | 0.00 | 2.12 |
| HT29_11_GR_ChDB_0092 | SB218078 | 3uM | 6h | ZNF721 | 0.00 | 2.06 |
| HT29_11_GR_ChDB_0092 | SB218078 | 3uM | 6h | RRAGC | 0.00 | 2.24 |
| HT29_11_GR_ChDB_0092 | SB218078 | 3uM | 6h | RSF1 | 0.00 | 2.06 |
| HT29_11_GR_ChDB_0092 | SB218078 | 3uM | 6h | GTF3C4 | 0.00 | 2.27 |
| HT29_11_GR_ChDB_0092 | SB218078 | 3uM | 6h | DNAJB14 | 0.00 | 2.19 |
| HT29_11_GR_ChDB_0092 | SB218078 | 3uM | 6h | PALB2 | 0.00 | 2.17 |
| HT29_11_GR_ChDB_0092 | SB218078 | 3uM | 6h | ZFYVE21 | 0.00 | 2.36 |
| HT29_11_GR_ChDB_0092 | SB218078 | 3uM | 6h | CCNL2 | 0.00 | 2.39 |
| HT29_11_GR_ChDB_0092 | SB218078 | 3uM | 6h | ZNF207 | 0.00 | 2.66 |
| HT29_11_GR_ChDB_0092 | SB218078 | 3uM | 6h | CPSF6 | 0.00 | 2.35 |
| HT29_11_GR_ChDB_0092 | SB218078 | 3uM | 6h | DDIT4 | 0.00 | 2.31 |
| HT29_11_GR_ChDB_0092 | SB218078 | 3uM | 6h | CCNT2 | 0.00 | 2.16 |
| HT29_11_GR_ChDB_0092 | SB218078 | 3uM | 6h | DNMBP | 0.00 | 2.15 |
| HT29_11_GR_ChDB_0092 | SB218078 | 3uM | 6h | SLC41A3 | 0.00 | 2.02 |
| HT29_11_GR_ChDB_0092 | SB218078 | 3uM | 6h | PRPF39 | 0.00 | 2.41 |
| HT29_11_GR_ChDB_0092 | SB218078 | 3uM | 6h | HNRNPA1 | 0.00 | 2.76 |
| HT29_11_GR_ChDB_0092 | SB218078 | 3uM | 6h | KLHDC4 | 0.00 | 2.09 |
| HT29_11_GR_ChDB_0092 | SB218078 | 3uM | 6h | THAP10 | 0.00 | 2.77 |
| HT29_11_GR_ChDB_0092 | SB218078 | 3uM | 6h | ZFYVE16 | 0.00 | 0.34 |
| HT29_11_GR_ChDB_0092 | SB218078 | 3uM | 6h | RAD51AP1 | 0.00 | 0.39 |
| HT29_11_GR_ChDB_0092 | SB218078 | 3uM | 6h | RAD51 | 0.00 | 0.44 |
| HT29_11_GR_ChDB_0092 | SB218078 | 3uM | 6h | YTHDC2 | 0.00 | 0.38 |
| HT29_11_GR_ChDB_0092 | SB218078 | 3uM | 6h | CHML | 0.00 | 0.48 |
| HT29_11_GR_ChDB_0092 | SB218078 | 3uM | 6h | RAB28 | 0.00 | 0.37 |
| HT29_11_GR_ChDB_0092 | SB218078 | 3uM | 6h | CYP2R1 | 0.00 | 0.47 |
| HT29_11_GR_ChDB_0092 | SB218078 | 3uM | 6h | DDX52 | 0.00 | 0.35 |
| HT29_11_GR_ChDB_0092 | SB218078 | 3uM | 6h | KHNYN | 0.00 | 0.45 |
| HT29_11_GR_ChDB_0092 | SB218078 | 3uM | 6h | SMCHD1 | 0.00 | 0.44 |
| HT29_11_GR_ChDB_0092 | SB218078 | 3uM | 6h | SMC5 | 0.00 | 0.48 |
| HT29_11_GR_ChDB_0092 | SB218078 | 3uM | 6h | SKA1 | 0.00 | 0.45 |
| HT29_11_GR_ChDB_0092 | SB218078 | 3uM | 6h | PRKD3 | 0.00 | 0.49 |
| HT29_11_GR_ChDB_0092 | SB218078 | 3uM | 6h | SMUG1 | 0.00 | 0.48 |
| HT29_11_GR_ChDB_0092 | SB218078 | 3uM | 6h | MLF1IP | 0.00 | 0.50 |
| HT29_11_GR_ChDB_0092 | SB218078 | 3uM | 6h | NEIL3 | 0.00 | 0.48 |
| HT29_11_GR_ChDB_0092 | SB218078 | 3uM | 6h | SLC38A2 | 0.00 | 0.44 |
| HT29_11_GR_ChDB_0092 | SB218078 | 3uM | 6h | NFYB | 0.00 | 0.45 |
| HT29_11_GR_ChDB_0092 | SB218078 | 3uM | 6h | AMIGO2 | 0.00 | 0.18 |
| HT29_11_GR_ChDB_0092 | SB218078 | 3uM | 6h | MYO10 | 0.00 | 0.47 |
| HT29_11_GR_ChDB_0092 | SB218078 | 3uM | 6h | GPRC5A | 0.00 | 0.33 |
| HT29_11_GR_ChDB_0092 | SB218078 | 3uM | 6h | LGR4 | 0.00 | 0.44 |
| HT29_11_GR_ChDB_0092 | SB218078 | 3uM | 6h | PIGA | 0.00 | 0.47 |
| HT29_11_GR_ChDB_0092 | SB218078 | 3uM | 6h | KIF1B | 0.00 | 0.41 |
| HT29_11_GR_ChDB_0092 | SB218078 | 3uM | 6h | NUP98 | 0.00 | 0.22 |
| HT29_11_GR_ChDB_0092 | SB218078 | 3uM | 6h | ZC3H14 | 0.00 | 0.44 |
| HT29_11_GR_ChDB_0092 | SB218078 | 3uM | 6h | TASP1 | 0.00 | 0.38 |
| HT29_11_GR_ChDB_0092 | SB218078 | 3uM | 6h | UBA2 | 0.00 | 0.37 |
| HT29_11_GR_ChDB_0092 | SB218078 | 3uM | 6h | WSB1 | 0.00 | 0.41 |
| HT29_11_GR_ChDB_0092 | SB218078 | 3uM | 6h | PPP2R1B | 0.00 | 0.49 |
| HT29_11_GR_ChDB_0092 | SB218078 | 3uM | 6h | GCH1 | 0.00 | 0.39 |
| HT29_11_GR_ChDB_0092 | SB218078 | 3uM | 6h | CLPX | 0.00 | 0.48 |
| HT29_11_GR_ChDB_0092 | SB218078 | 3uM | 6h | ATXN7L3B | 0.00 | 0.48 |
| HT29_11_GR_ChDB_0092 | SB218078 | 3uM | 6h | NFATC2IP | 0.00 | 0.44 |
| HT29_11_GR_ChDB_0092 | SB218078 | 3uM | 6h | PAWR | 0.00 | 0.32 |
| HT29_11_GR_ChDB_0092 | SB218078 | 3uM | 6h | TDP1 | 0.00 | 0.43 |
| HT29_11_GR_ChDB_0092 | SB218078 | 3uM | 6h | TCF7L2 | 0.00 | 0.44 |
| HT29_11_GR_ChDB_0092 | SB218078 | 3uM | 6h | SRSF7 | 0.00 | 0.25 |
| HT29_11_GR_ChDB_0092 | SB218078 | 3uM | 6h | CD55 | 0.00 | 0.40 |
| HT29_11_GR_ChDB_0092 | SB218078 | 3uM | 6h | TRAM2 | 0.00 | 0.41 |
| HT29_11_GR_ChDB_0092 | SB218078 | 3uM | 6h | ITGB1BP1 | 0.00 | 0.43 |
| HT29_11_GR_ChDB_0092 | SB218078 | 3uM | 6h | CD82 | 0.00 | 0.47 |
| HT29_11_GR_ChDB_0092 | SB218078 | 3uM | 6h | DKK1 | 0.00 | 0.33 |
| HT29_11_GR_ChDB_0092 | SB218078 | 3uM | 6h | POLE2 | 0.00 | 0.37 |
| HT29_11_GR_ChDB_0092 | SB218078 | 3uM | 6h | SLC25A11 | 0.00 | 0.50 |
| HT29_11_GR_ChDB_0092 | SB218078 | 3uM | 6h | LMO4 | 0.00 | 0.35 |
| HT29_11_GR_ChDB_0092 | SB218078 | 3uM | 6h | IL8 | 0.00 | 0.40 |
| HT29_11_GR_ChDB_0092 | SB218078 | 3uM | 6h | NRP1 | 0.00 | 0.42 |
| HT29_11_GR_ChDB_0092 | SB218078 | 3uM | 6h | PVR | 0.00 | 0.49 |
| HT29_11_GR_ChDB_0092 | SB218078 | 3uM | 6h | TROVE2 | 0.00 | 0.49 |
| HT29_11_GR_ChDB_0092 | SB218078 | 3uM | 6h | ETS1 | 0.00 | 0.48 |
| HT29_11_GR_ChDB_0092 | SB218078 | 3uM | 6h | AHCTF1 | 0.00 | 0.37 |
| HT29_11_GR_ChDB_0092 | SB218078 | 3uM | 6h | MLPH | 0.00 | 0.41 |
| HT29_11_GR_ChDB_0092 | SB218078 | 3uM | 6h | SAV1 | 0.00 | 0.46 |
| HT29_11_GR_ChDB_0092 | SB218078 | 3uM | 6h | PAGR1 | 0.00 | 0.36 |
| HT29_11_GR_ChDB_0092 | SB218078 | 3uM | 6h | KANK2 | 0.00 | 0.33 |
| HT29_11_GR_ChDB_0092 | SB218078 | 3uM | 6h | TSEN2 | 0.00 | 0.50 |
| HT29_11_GR_ChDB_0092 | SB218078 | 3uM | 6h | PANK3 | 0.00 | 0.46 |
| HT29_11_GR_ChDB_0092 | SB218078 | 3uM | 6h | NDC80 | 0.00 | 0.50 |
| HT29_11_GR_ChDB_0092 | SB218078 | 3uM | 6h | PKNOX1 | 0.00 | 0.47 |
| HT29_11_GR_ChDB_0092 | SB218078 | 3uM | 6h | GRK5 | 0.00 | 0.41 |
| HT29_11_GR_ChDB_0092 | SB218078 | 3uM | 6h | KIF11 | 0.00 | 0.43 |
| HT29_11_GR_ChDB_0092 | SB218078 | 3uM | 6h | YIPF6 | 0.00 | 0.44 |
| HT29_11_GR_ChDB_0092 | SB218078 | 3uM | 6h | C4orf19 | 0.00 | 0.44 |
| HT29_11_GR_ChDB_0092 | SB218078 | 3uM | 6h | POLA2 | 0.00 | 0.40 |
| HT29_11_GR_ChDB_0092 | SB218078 | 3uM | 6h | ARMCX5 | 0.00 | 0.29 |
| HT29_11_GR_ChDB_0092 | SB218078 | 3uM | 6h | DEPDC1 | 0.00 | 0.45 |
| HT29_11_GR_ChDB_0092 | SB218078 | 3uM | 6h | RAPGEF2 | 0.00 | 0.38 |
| HT29_11_GR_ChDB_0092 | SB218078 | 3uM | 6h | CDS1 | 0.00 | 0.38 |
| HT29_11_GR_ChDB_0092 | SB218078 | 3uM | 6h | ITGB6 | 0.00 | 0.36 |
| HT29_11_GR_ChDB_0092 | SB218078 | 3uM | 6h | DAAM1 | 0.00 | 0.41 |
| HT29_11_GR_ChDB_0092 | SB218078 | 3uM | 6h | GRHL2 | 0.00 | 0.46 |
| HT29_11_GR_ChDB_0092 | SB218078 | 3uM | 6h | C1orf116 | 0.00 | 0.37 |
| HT29_11_GR_ChDB_0092 | SB218078 | 3uM | 6h | TRMT61B | 0.00 | 0.26 |
| HT29_11_GR_ChDB_0092 | SB218078 | 3uM | 6h | ENSA | 0.00 | 0.46 |
| HT29_11_GR_ChDB_0092 | SB218078 | 3uM | 6h | TLE1 | 0.00 | 0.46 |
| HT29_11_GR_ChDB_0092 | SB218078 | 3uM | 6h | JUND | 0.00 | 0.25 |
| HT29_11_GR_ChDB_0092 | SB218078 | 3uM | 6h | SARS | 0.00 | 0.46 |
| HT29_11_GR_ChDB_0092 | SB218078 | 3uM | 6h | SRSF1 | 0.00 | 0.20 |
| HT29_11_GR_ChDB_0092 | SB218078 | 3uM | 6h | RBMS1 | 0.00 | 0.29 |
| HT29_11_GR_ChDB_0092 | SB218078 | 3uM | 6h | MBNL2 | 0.00 | 0.25 |
| HT29_11_GR_ChDB_0092 | SB218078 | 3uM | 6h | B4GALT6 | 0.00 | 0.43 |
| HT29_11_GR_ChDB_0092 | SB218078 | 3uM | 6h | ITSN2 | 0.00 | 0.43 |
| HT29_11_GR_ChDB_0092 | SB218078 | 3uM | 6h | TRMT13 | 0.00 | 0.47 |
| HT29_11_GR_ChDB_0092 | SB218078 | 3uM | 6h | CAV2 | 0.00 | 0.46 |
| HT29_11_GR_ChDB_0092 | SB218078 | 3uM | 6h | PTS | 0.00 | 0.49 |
| HT29_11_GR_ChDB_0092 | SB218078 | 3uM | 6h | CASP6 | 0.00 | 0.46 |
| HT29_11_GR_ChDB_0092 | SB218078 | 3uM | 6h | TSPYL4 | 0.00 | 0.49 |
| HT29_11_GR_ChDB_0092 | SB218078 | 3uM | 6h | NAGK | 0.00 | 0.41 |
| HT29_11_GR_ChDB_0092 | SB218078 | 3uM | 6h | TMEM168 | 0.00 | 0.47 |
| HT29_11_GR_ChDB_0092 | SB218078 | 3uM | 6h | KRIT1 | 0.00 | 0.35 |
| HT29_11_GR_ChDB_0092 | SB218078 | 3uM | 6h | ZDHHC18 | 0.00 | 0.42 |
| HT29_11_GR_ChDB_0092 | SB218078 | 3uM | 6h | INTS6 | 0.00 | 0.46 |
| HT29_11_GR_ChDB_0094 | GSK-3 inhibitor IX | 10uM | 6h | ERBB3 | 1.00 | 0.41 |
| HT29_11_GR_ChDB_0094 | GSK-3 inhibitor IX | 10uM | 6h | HMOX1 | 0.00 | 2.40 |
| HT29_11_GR_ChDB_0094 | GSK-3 inhibitor IX | 10uM | 6h | PMAIP1 | 0.00 | 2.48 |
| HT29_11_GR_ChDB_0094 | GSK-3 inhibitor IX | 10uM | 6h | OSBP | 0.00 | 2.02 |
| HT29_11_GR_ChDB_0094 | GSK-3 inhibitor IX | 10uM | 6h | SLC1A4 | 0.00 | 2.73 |
| HT29_11_GR_ChDB_0094 | GSK-3 inhibitor IX | 10uM | 6h | TNS3 | 0.00 | 0.46 |
| HT29_11_GR_ChDB_0094 | GSK-3 inhibitor IX | 10uM | 6h | BNIP3L | 0.00 | 0.38 |
| HT29_11_GR_ChDB_0094 | GSK-3 inhibitor IX | 10uM | 6h | EMP1 | 0.00 | 0.25 |
| HT29_11_GR_ChDB_0094 | GSK-3 inhibitor IX | 10uM | 6h | GPRC5A | 0.00 | 0.32 |
| HT29_11_GR_ChDB_0094 | GSK-3 inhibitor IX | 10uM | 6h | MAGI1 | 0.00 | 0.46 |
| HT29_11_GR_ChDB_0094 | GSK-3 inhibitor IX | 10uM | 6h | CASP6 | 0.00 | 0.49 |
| HT29_11_GR_ChDB_0094 | GSK-3 inhibitor IX | 10uM | 6h | NET1 | 0.00 | 0.41 |
| HT29_12_GR_ChDB_0103 | U-0126 | 30uM | 6h | MYC | 1.00 | 0.11 |
| HT29_12_GR_ChDB_0103 | U-0126 | 30uM | 6h | EHF | 0.01 | 2.21 |
| HT29_12_GR_ChDB_0103 | U-0126 | 30uM | 6h | TIMP2 | 0.01 | 2.07 |
| HT29_12_GR_ChDB_0103 | U-0126 | 30uM | 6h | PBXIP1 | 0.00 | 2.10 |
| HT29_12_GR_ChDB_0104 | SU11274 | 30uM | 6h | SGK1 | 1.00 | 0.33 |
| HT29_12_GR_ChDB_0104 | SU11274 | 30uM | 6h | PLK2 | 0.37 | 0.42 |
| HT29_12_GR_ChDB_0104 | SU11274 | 30uM | 6h | PKDCC | 0.36 | 0.39 |
| HT29_12_GR_ChDB_0104 | SU11274 | 30uM | 6h | HMGCR | 0.33 | 2.46 |
| HT29_12_GR_ChDB_0104 | SU11274 | 30uM | 6h | OPN3 | 0.17 | 2.72 |
| HT29_12_GR_ChDB_0104 | SU11274 | 30uM | 6h | NAB1 | 0.12 | 0.47 |
| HT29_12_GR_ChDB_0104 | SU11274 | 30uM | 6h | HIST1H2AC | 0.08 | 4.00 |
| HT29_12_GR_ChDB_0104 | SU11274 | 30uM | 6h | KLF6 | 0.07 | 2.01 |
| HT29_12_GR_ChDB_0104 | SU11274 | 30uM | 6h | THRAP3 | 0.06 | 0.49 |
| HT29_12_GR_ChDB_0104 | SU11274 | 30uM | 6h | CCDC92 | 0.05 | 2.06 |
| HT29_12_GR_ChDB_0104 | SU11274 | 30uM | 6h | BNIP3L | 0.04 | 2.04 |
| HT29_12_GR_ChDB_0104 | SU11274 | 30uM | 6h | PIK3R3 | 0.02 | 2.18 |
| HT29_12_GR_ChDB_0104 | SU11274 | 30uM | 6h | PLAUR | 0.02 | 0.47 |
| HT29_12_GR_ChDB_0104 | SU11274 | 30uM | 6h | ATF5 | 0.01 | 0.50 |
| HT29_12_GR_ChDB_0104 | SU11274 | 30uM | 6h | P2RY2 | 0.01 | 0.46 |
| HT29_12_GR_ChDB_0104 | SU11274 | 30uM | 6h | EHF | 0.01 | 0.41 |
| HT29_12_GR_ChDB_0104 | SU11274 | 30uM | 6h | SUPT16H | 0.00 | 0.37 |
| HT29_12_GR_ChDB_0104 | SU11274 | 30uM | 6h | INSIG1 | 0.00 | 3.54 |
| HT29_12_GR_ChDB_0104 | SU11274 | 30uM | 6h | SMURF2 | 0.00 | 2.20 |
| HT29_12_GR_ChDB_0104 | SU11274 | 30uM | 6h | CDC25C | 0.00 | 2.20 |
| HT29_12_GR_ChDB_0104 | SU11274 | 30uM | 6h | HIST1H2BK | 0.00 | 2.18 |
| HT29_12_GR_ChDB_0104 | SU11274 | 30uM | 6h | ZNF92 | 0.00 | 2.37 |
| HT29_12_GR_ChDB_0104 | SU11274 | 30uM | 6h | KAT5 | 0.00 | 0.48 |
| HT29_12_GR_ChDB_0104 | SU11274 | 30uM | 6h | SUCNR1 | 0.00 | 0.40 |
| HT29_12_GR_ChDB_0104 | SU11274 | 30uM | 6h | PDS5A | 0.00 | 0.48 |
| HT29_13_GR_ChDB_0120 | Crizotinib | 10uM | 6h | BRAF | 1.00 | 2.70 |
| HT29_13_GR_ChDB_0120 | Crizotinib | 10uM | 6h | KLF6 | 0.22 | 12.46 |
| HT29_13_GR_ChDB_0120 | Crizotinib | 10uM | 6h | CDK6 | 0.14 | 0.36 |
| HT29_13_GR_ChDB_0120 | Crizotinib | 10uM | 6h | CCND1 | 0.14 | 0.23 |
| HT29_13_GR_ChDB_0120 | Crizotinib | 10uM | 6h | IL8 | 0.12 | 22.41 |
| HT29_13_GR_ChDB_0120 | Crizotinib | 10uM | 6h | ACVR1 | 0.11 | 2.12 |
| HT29_13_GR_ChDB_0120 | Crizotinib | 10uM | 6h | NFE2L2 | 0.11 | 2.50 |
| HT29_13_GR_ChDB_0120 | Crizotinib | 10uM | 6h | NDUFAF4 | 0.10 | 0.49 |
| HT29_13_GR_ChDB_0120 | Crizotinib | 10uM | 6h | BRCA1 | 0.09 | 0.17 |
| HT29_13_GR_ChDB_0120 | Crizotinib | 10uM | 6h | PIK3C2A | 0.09 | 0.35 |
| HT29_13_GR_ChDB_0120 | Crizotinib | 10uM | 6h | HBEGF | 0.09 | 2.75 |
| HT29_13_GR_ChDB_0120 | Crizotinib | 10uM | 6h | CSNK1E | 0.08 | 0.48 |
| HT29_13_GR_ChDB_0120 | Crizotinib | 10uM | 6h | IRAK2 | 0.06 | 19.01 |
| HT29_13_GR_ChDB_0120 | Crizotinib | 10uM | 6h | SYK | 0.06 | 0.47 |
| HT29_13_GR_ChDB_0120 | Crizotinib | 10uM | 6h | MAPK9 | 0.05 | 0.36 |
| HT29_13_GR_ChDB_0120 | Crizotinib | 10uM | 6h | UGCG | 0.05 | 8.96 |
| HT29_13_GR_ChDB_0120 | Crizotinib | 10uM | 6h | HNMT | 0.05 | 2.76 |
| HT29_13_GR_ChDB_0120 | Crizotinib | 10uM | 6h | HDHD1 | 0.05 | 0.39 |
| HT29_13_GR_ChDB_0120 | Crizotinib | 10uM | 6h | RPS6KA1 | 0.05 | 0.48 |
| HT29_13_GR_ChDB_0120 | Crizotinib | 10uM | 6h | POLE2 | 0.05 | 0.21 |
| HT29_13_GR_ChDB_0120 | Crizotinib | 10uM | 6h | MTF2 | 0.05 | 2.56 |
| HT29_13_GR_ChDB_0120 | Crizotinib | 10uM | 6h | SMAD3 | 0.05 | 5.15 |
| HT29_13_GR_ChDB_0120 | Crizotinib | 10uM | 6h | ATR | 0.04 | 0.32 |
| HT29_13_GR_ChDB_0120 | Crizotinib | 10uM | 6h | SQSTM1 | 0.04 | 4.82 |
| HT29_13_GR_ChDB_0120 | Crizotinib | 10uM | 6h | RIT1 | 0.04 | 3.69 |
| HT29_13_GR_ChDB_0120 | Crizotinib | 10uM | 6h | CD58 | 0.03 | 0.39 |
| HT29_13_GR_ChDB_0120 | Crizotinib | 10uM | 6h | BMP4 | 0.03 | 2.23 |
| HT29_13_GR_ChDB_0120 | Crizotinib | 10uM | 6h | DDIT4 | 0.03 | 4.14 |
| HT29_13_GR_ChDB_0120 | Crizotinib | 10uM | 6h | PARP2 | 0.02 | 0.34 |
| HT29_13_GR_ChDB_0120 | Crizotinib | 10uM | 6h | CCL20 | 0.02 | 13.80 |
| HT29_13_GR_ChDB_0120 | Crizotinib | 10uM | 6h | SLK | 0.02 | 0.34 |
| HT29_13_GR_ChDB_0120 | Crizotinib | 10uM | 6h | PLXNA1 | 0.02 | 0.24 |
| HT29_13_GR_ChDB_0120 | Crizotinib | 10uM | 6h | VRK3 | 0.02 | 0.37 |
| HT29_13_GR_ChDB_0120 | Crizotinib | 10uM | 6h | CNOT8 | 0.02 | 3.06 |
| HT29_13_GR_ChDB_0120 | Crizotinib | 10uM | 6h | ROCK2 | 0.02 | 0.29 |
| HT29_13_GR_ChDB_0120 | Crizotinib | 10uM | 6h | BCL6 | 0.02 | 6.62 |
| HT29_13_GR_ChDB_0120 | Crizotinib | 10uM | 6h | ZNF503 | 0.02 | 2.09 |
| HT29_13_GR_ChDB_0120 | Crizotinib | 10uM | 6h | ZNF217 | 0.02 | 2.71 |
| HT29_13_GR_ChDB_0120 | Crizotinib | 10uM | 6h | UBE3C | 0.02 | 4.86 |
| HT29_13_GR_ChDB_0120 | Crizotinib | 10uM | 6h | PIK3R1 | 0.02 | 0.28 |
| HT29_13_GR_ChDB_0120 | Crizotinib | 10uM | 6h | MCL1 | 0.02 | 2.86 |
| HT29_13_GR_ChDB_0120 | Crizotinib | 10uM | 6h | GCLC | 0.02 | 2.75 |
| HT29_13_GR_ChDB_0120 | Crizotinib | 10uM | 6h | NFKBIA | 0.02 | 8.53 |
| HT29_13_GR_ChDB_0120 | Crizotinib | 10uM | 6h | FANCL | 0.02 | 0.14 |
| HT29_13_GR_ChDB_0120 | Crizotinib | 10uM | 6h | TLK2 | 0.02 | 2.61 |
| HT29_13_GR_ChDB_0120 | Crizotinib | 10uM | 6h | BIK | 0.02 | 3.26 |
| HT29_13_GR_ChDB_0120 | Crizotinib | 10uM | 6h | CDKN1B | 0.02 | 3.44 |
| HT29_13_GR_ChDB_0120 | Crizotinib | 10uM | 6h | OVOL2 | 0.02 | 2.54 |
| HT29_13_GR_ChDB_0120 | Crizotinib | 10uM | 6h | EIF2AK2 | 0.02 | 0.46 |
| HT29_13_GR_ChDB_0120 | Crizotinib | 10uM | 6h | MACF1 | 0.02 | 0.41 |
| HT29_13_GR_ChDB_0120 | Crizotinib | 10uM | 6h | KLF3 | 0.01 | 2.68 |
| HT29_13_GR_ChDB_0120 | Crizotinib | 10uM | 6h | GNA13 | 0.01 | 2.08 |
| HT29_13_GR_ChDB_0120 | Crizotinib | 10uM | 6h | ZNF212 | 0.01 | 2.22 |
| HT29_13_GR_ChDB_0120 | Crizotinib | 10uM | 6h | BCL10 | 0.01 | 3.18 |
| HT29_13_GR_ChDB_0120 | Crizotinib | 10uM | 6h | SRC | 0.01 | 2.05 |
| HT29_13_GR_ChDB_0120 | Crizotinib | 10uM | 6h | MXD1 | 0.01 | 5.22 |
| HT29_13_GR_ChDB_0120 | Crizotinib | 10uM | 6h | RND3 | 0.01 | 2.29 |
| HT29_13_GR_ChDB_0120 | Crizotinib | 10uM | 6h | MEN1 | 0.01 | 0.48 |
| HT29_13_GR_ChDB_0120 | Crizotinib | 10uM | 6h | CLIC4 | 0.01 | 2.88 |
| HT29_13_GR_ChDB_0120 | Crizotinib | 10uM | 6h | PLK2 | 0.01 | 9.70 |
| HT29_13_GR_ChDB_0120 | Crizotinib | 10uM | 6h | IRF2 | 0.01 | 2.09 |
| HT29_13_GR_ChDB_0120 | Crizotinib | 10uM | 6h | MDH2 | 0.01 | 0.35 |
| HT29_13_GR_ChDB_0120 | Crizotinib | 10uM | 6h | TNFRSF10B | 0.01 | 5.02 |
| HT29_13_GR_ChDB_0120 | Crizotinib | 10uM | 6h | CRK | 0.01 | 2.05 |
| HT29_13_GR_ChDB_0120 | Crizotinib | 10uM | 6h | DDIT3 | 0.01 | 3.33 |
| HT29_13_GR_ChDB_0120 | Crizotinib | 10uM | 6h | EWSR1 | 0.01 | 3.70 |
| HT29_13_GR_ChDB_0120 | Crizotinib | 10uM | 6h | CREB3 | 0.01 | 2.18 |
| HT29_13_GR_ChDB_0120 | Crizotinib | 10uM | 6h | MARK4 | 0.01 | 0.48 |
| HT29_13_GR_ChDB_0120 | Crizotinib | 10uM | 6h | TNFAIP3 | 0.01 | 4.49 |
| HT29_13_GR_ChDB_0120 | Crizotinib | 10uM | 6h | EXO1 | 0.01 | 0.32 |
| HT29_13_GR_ChDB_0120 | Crizotinib | 10uM | 6h | TRIM27 | 0.01 | 0.42 |
| HT29_13_GR_ChDB_0120 | Crizotinib | 10uM | 6h | CREB1 | 0.01 | 2.31 |
| HT29_13_GR_ChDB_0120 | Crizotinib | 10uM | 6h | VEGFA | 0.01 | 2.86 |
| HT29_13_GR_ChDB_0120 | Crizotinib | 10uM | 6h | BLZF1 | 0.01 | 2.48 |
| HT29_13_GR_ChDB_0120 | Crizotinib | 10uM | 6h | SMARCE1 | 0.01 | 2.08 |
| HT29_13_GR_ChDB_0120 | Crizotinib | 10uM | 6h | POLR3B | 0.01 | 0.30 |
| HT29_13_GR_ChDB_0120 | Crizotinib | 10uM | 6h | ARID5B | 0.01 | 0.37 |
| HT29_13_GR_ChDB_0120 | Crizotinib | 10uM | 6h | PKN2 | 0.01 | 0.47 |
| HT29_13_GR_ChDB_0120 | Crizotinib | 10uM | 6h | NFIL3 | 0.01 | 2.02 |
| HT29_13_GR_ChDB_0120 | Crizotinib | 10uM | 6h | MPZL1 | 0.01 | 0.44 |
| HT29_13_GR_ChDB_0120 | Crizotinib | 10uM | 6h | MBTPS1 | 0.01 | 0.32 |
| HT29_13_GR_ChDB_0120 | Crizotinib | 10uM | 6h | ZNF317 | 0.01 | 2.15 |
| HT29_13_GR_ChDB_0120 | Crizotinib | 10uM | 6h | GCA | 0.01 | 0.44 |
| HT29_13_GR_ChDB_0120 | Crizotinib | 10uM | 6h | GRB7 | 0.01 | 2.54 |
| HT29_13_GR_ChDB_0120 | Crizotinib | 10uM | 6h | CTTN | 0.01 | 4.50 |
| HT29_13_GR_ChDB_0120 | Crizotinib | 10uM | 6h | PCGF3 | 0.01 | 4.73 |
| HT29_13_GR_ChDB_0120 | Crizotinib | 10uM | 6h | KIAA0907 | 0.01 | 0.41 |
| HT29_13_GR_ChDB_0120 | Crizotinib | 10uM | 6h | HSD17B2 | 0.01 | 3.71 |
| HT29_13_GR_ChDB_0120 | Crizotinib | 10uM | 6h | FBXL20 | 0.01 | 2.07 |
| HT29_13_GR_ChDB_0120 | Crizotinib | 10uM | 6h | ATF2 | 0.01 | 3.62 |
| HT29_13_GR_ChDB_0120 | Crizotinib | 10uM | 6h | PFKFB3 | 0.01 | 0.37 |
| HT29_13_GR_ChDB_0120 | Crizotinib | 10uM | 6h | PLAUR | 0.01 | 3.90 |
| HT29_13_GR_ChDB_0120 | Crizotinib | 10uM | 6h | NR2F6 | 0.01 | 0.20 |
| HT29_13_GR_ChDB_0120 | Crizotinib | 10uM | 6h | CXCR4 | 0.01 | 5.50 |
| HT29_13_GR_ChDB_0120 | Crizotinib | 10uM | 6h | ITFG1 | 0.01 | 0.44 |
| HT29_13_GR_ChDB_0120 | Crizotinib | 10uM | 6h | ITSN1 | 0.01 | 0.49 |
| HT29_13_GR_ChDB_0120 | Crizotinib | 10uM | 6h | TGIF1 | 0.01 | 2.27 |
| HT29_13_GR_ChDB_0120 | Crizotinib | 10uM | 6h | PTEN | 0.01 | 0.43 |
| HT29_13_GR_ChDB_0120 | Crizotinib | 10uM | 6h | NONO | 0.01 | 2.18 |
| HT29_13_GR_ChDB_0120 | Crizotinib | 10uM | 6h | SMNDC1 | 0.01 | 2.56 |
| HT29_13_GR_ChDB_0120 | Crizotinib | 10uM | 6h | PIK3C2B | 0.01 | 0.40 |
| HT29_13_GR_ChDB_0120 | Crizotinib | 10uM | 6h | DNA2 | 0.01 | 0.24 |
| HT29_13_GR_ChDB_0120 | Crizotinib | 10uM | 6h | PDS5A | 0.01 | 2.09 |
| HT29_13_GR_ChDB_0120 | Crizotinib | 10uM | 6h | ABHD2 | 0.01 | 4.78 |
| HT29_13_GR_ChDB_0120 | Crizotinib | 10uM | 6h | IGHMBP2 | 0.01 | 2.40 |
| HT29_13_GR_ChDB_0120 | Crizotinib | 10uM | 6h | BTG3 | 0.01 | 2.35 |
| HT29_13_GR_ChDB_0120 | Crizotinib | 10uM | 6h | AGL | 0.01 | 0.49 |
| HT29_13_GR_ChDB_0120 | Crizotinib | 10uM | 6h | ICAM1 | 0.01 | 4.98 |
| HT29_13_GR_ChDB_0120 | Crizotinib | 10uM | 6h | ADCY3 | 0.00 | 0.36 |
| HT29_13_GR_ChDB_0120 | Crizotinib | 10uM | 6h | ZNF3 | 0.00 | 3.79 |
| HT29_13_GR_ChDB_0120 | Crizotinib | 10uM | 6h | STAM2 | 0.00 | 0.42 |
| HT29_13_GR_ChDB_0120 | Crizotinib | 10uM | 6h | ZNF8 | 0.00 | 2.09 |
| HT29_13_GR_ChDB_0120 | Crizotinib | 10uM | 6h | CCNE1 | 0.00 | 0.38 |
| HT29_13_GR_ChDB_0120 | Crizotinib | 10uM | 6h | ELF4 | 0.00 | 2.47 |
| HT29_13_GR_ChDB_0120 | Crizotinib | 10uM | 6h | INSIG1 | 0.00 | 2.89 |
| HT29_13_GR_ChDB_0120 | Crizotinib | 10uM | 6h | PRKDC | 0.00 | 0.49 |
| HT29_13_GR_ChDB_0120 | Crizotinib | 10uM | 6h | TMEM109 | 0.00 | 0.49 |
| HT29_13_GR_ChDB_0120 | Crizotinib | 10uM | 6h | USP6NL | 0.00 | 2.15 |
| HT29_13_GR_ChDB_0120 | Crizotinib | 10uM | 6h | ADORA2B | 0.00 | 0.35 |
| HT29_13_GR_ChDB_0120 | Crizotinib | 10uM | 6h | BCL2L2 | 0.00 | 0.22 |
| HT29_13_GR_ChDB_0120 | Crizotinib | 10uM | 6h | IKBKAP | 0.00 | 0.33 |
| HT29_13_GR_ChDB_0120 | Crizotinib | 10uM | 6h | UBAP1 | 0.00 | 2.40 |
| HT29_13_GR_ChDB_0120 | Crizotinib | 10uM | 6h | NRP1 | 0.00 | 0.48 |
| HT29_13_GR_ChDB_0120 | Crizotinib | 10uM | 6h | MAPKAP1 | 0.00 | 0.42 |
| HT29_13_GR_ChDB_0120 | Crizotinib | 10uM | 6h | RSPRY1 | 0.00 | 2.28 |
| HT29_13_GR_ChDB_0120 | Crizotinib | 10uM | 6h | SLC35A1 | 0.00 | 0.42 |
| HT29_13_GR_ChDB_0120 | Crizotinib | 10uM | 6h | TNFRSF11A | 0.00 | 0.38 |
| HT29_13_GR_ChDB_0120 | Crizotinib | 10uM | 6h | FOSL1 | 0.00 | 2.01 |
| HT29_13_GR_ChDB_0120 | Crizotinib | 10uM | 6h | NF1 | 0.00 | 0.45 |
| HT29_13_GR_ChDB_0120 | Crizotinib | 10uM | 6h | SAT1 | 0.00 | 4.61 |
| HT29_13_GR_ChDB_0120 | Crizotinib | 10uM | 6h | ZAK | 0.00 | 0.43 |
| HT29_13_GR_ChDB_0120 | Crizotinib | 10uM | 6h | ANKRD10 | 0.00 | 0.45 |
| HT29_13_GR_ChDB_0120 | Crizotinib | 10uM | 6h | ACAD8 | 0.00 | 0.36 |
| HT29_13_GR_ChDB_0120 | Crizotinib | 10uM | 6h | DHRS3 | 0.00 | 2.52 |
| HT29_13_GR_ChDB_0120 | Crizotinib | 10uM | 6h | E2F5 | 0.00 | 0.44 |
| HT29_13_GR_ChDB_0120 | Crizotinib | 10uM | 6h | FGFR1OP | 0.00 | 0.29 |
| HT29_13_GR_ChDB_0120 | Crizotinib | 10uM | 6h | ZMYM2 | 0.00 | 0.38 |
| HT29_13_GR_ChDB_0120 | Crizotinib | 10uM | 6h | ESRRA | 0.00 | 0.47 |
| HT29_13_GR_ChDB_0120 | Crizotinib | 10uM | 6h | DICER1 | 0.00 | 0.24 |
| HT29_13_GR_ChDB_0120 | Crizotinib | 10uM | 6h | CCDC85B | 0.00 | 0.35 |
| HT29_13_GR_ChDB_0120 | Crizotinib | 10uM | 6h | CSNK1D | 0.00 | 3.09 |
| HT29_13_GR_ChDB_0120 | Crizotinib | 10uM | 6h | TUBB3 | 0.00 | 0.37 |
| HT29_13_GR_ChDB_0120 | Crizotinib | 10uM | 6h | RC3H2 | 0.00 | 2.41 |
| HT29_13_GR_ChDB_0120 | Crizotinib | 10uM | 6h | SNX13 | 0.00 | 0.37 |
| HT29_13_GR_ChDB_0120 | Crizotinib | 10uM | 6h | ETV6 | 0.00 | 2.63 |
| HT29_13_GR_ChDB_0120 | Crizotinib | 10uM | 6h | CXCL1 | 0.00 | 6.38 |
| HT29_13_GR_ChDB_0120 | Crizotinib | 10uM | 6h | DDAH2 | 0.00 | 2.09 |
| HT29_13_GR_ChDB_0120 | Crizotinib | 10uM | 6h | KLHL9 | 0.00 | 2.32 |
| HT29_13_GR_ChDB_0120 | Crizotinib | 10uM | 6h | AKR1C1 | 0.00 | 3.54 |
| HT29_13_GR_ChDB_0120 | Crizotinib | 10uM | 6h | CDC7 | 0.00 | 0.19 |
| HT29_13_GR_ChDB_0120 | Crizotinib | 10uM | 6h | NCOA3 | 0.00 | 2.15 |
| HT29_13_GR_ChDB_0120 | Crizotinib | 10uM | 6h | ETS2 | 0.00 | 2.47 |
| HT29_13_GR_ChDB_0120 | Crizotinib | 10uM | 6h | IRF6 | 0.00 | 2.75 |
| HT29_13_GR_ChDB_0120 | Crizotinib | 10uM | 6h | NR2F2 | 0.00 | 0.44 |
| HT29_13_GR_ChDB_0120 | Crizotinib | 10uM | 6h | PIK3R4 | 0.00 | 0.45 |
| HT29_13_GR_ChDB_0120 | Crizotinib | 10uM | 6h | PDXK | 0.00 | 0.45 |
| HT29_13_GR_ChDB_0120 | Crizotinib | 10uM | 6h | CCDC86 | 0.00 | 0.34 |
| HT29_13_GR_ChDB_0120 | Crizotinib | 10uM | 6h | GADD45A | 0.00 | 0.27 |
| HT29_13_GR_ChDB_0120 | Crizotinib | 10uM | 6h | MCM8 | 0.00 | 0.43 |
| HT29_13_GR_ChDB_0120 | Crizotinib | 10uM | 6h | MAP4K4 | 0.00 | 0.47 |
| HT29_13_GR_ChDB_0120 | Crizotinib | 10uM | 6h | HSPA5 | 0.00 | 0.44 |
| HT29_13_GR_ChDB_0120 | Crizotinib | 10uM | 6h | TXLNA | 0.00 | 0.44 |
| HT29_13_GR_ChDB_0120 | Crizotinib | 10uM | 6h | CRY1 | 0.00 | 0.29 |
| HT29_13_GR_ChDB_0120 | Crizotinib | 10uM | 6h | PPAP2B | 0.00 | 2.49 |
| HT29_13_GR_ChDB_0120 | Crizotinib | 10uM | 6h | THRAP3 | 0.00 | 3.23 |
| HT29_13_GR_ChDB_0120 | Crizotinib | 10uM | 6h | PHTF2 | 0.00 | 0.19 |
| HT29_13_GR_ChDB_0120 | Crizotinib | 10uM | 6h | CA12 | 0.00 | 0.33 |
| HT29_13_GR_ChDB_0120 | Crizotinib | 10uM | 6h | NEDD4 | 0.00 | 0.43 |
| HT29_13_GR_ChDB_0120 | Crizotinib | 10uM | 6h | SCAF8 | 0.00 | 2.70 |
| HT29_13_GR_ChDB_0120 | Crizotinib | 10uM | 6h | CDK7 | 0.00 | 2.24 |
| HT29_13_GR_ChDB_0120 | Crizotinib | 10uM | 6h | ARAF | 0.00 | 2.73 |
| HT29_13_GR_ChDB_0120 | Crizotinib | 10uM | 6h | TRIB1 | 0.00 | 2.39 |
| HT29_13_GR_ChDB_0120 | Crizotinib | 10uM | 6h | PPP2R5E | 0.00 | 0.48 |
| HT29_13_GR_ChDB_0120 | Crizotinib | 10uM | 6h | ETNK1 | 0.00 | 0.34 |
| HT29_13_GR_ChDB_0120 | Crizotinib | 10uM | 6h | BMP2 | 0.00 | 2.26 |
| HT29_13_GR_ChDB_0120 | Crizotinib | 10uM | 6h | SLC5A6 | 0.00 | 0.28 |
| HT29_13_GR_ChDB_0120 | Crizotinib | 10uM | 6h | ARIH1 | 0.00 | 3.12 |
| HT29_13_GR_ChDB_0120 | Crizotinib | 10uM | 6h | GLS | 0.00 | 0.21 |
| HT29_13_GR_ChDB_0120 | Crizotinib | 10uM | 6h | ATF7 | 0.00 | 2.66 |
| HT29_13_GR_ChDB_0120 | Crizotinib | 10uM | 6h | SERINC3 | 0.00 | 2.51 |
| HT29_13_GR_ChDB_0120 | Crizotinib | 10uM | 6h | BRCA2 | 0.00 | 0.33 |
| HT29_13_GR_ChDB_0120 | Crizotinib | 10uM | 6h | GLUL | 0.00 | 2.05 |
| HT29_13_GR_ChDB_0120 | Crizotinib | 10uM | 6h | TFPI | 0.00 | 2.78 |
| HT29_13_GR_ChDB_0120 | Crizotinib | 10uM | 6h | G3BP1 | 0.00 | 0.19 |
| HT29_13_GR_ChDB_0120 | Crizotinib | 10uM | 6h | CASP2 | 0.00 | 2.22 |
| HT29_13_GR_ChDB_0120 | Crizotinib | 10uM | 6h | GPR125 | 0.00 | 0.29 |
| HT29_13_GR_ChDB_0120 | Crizotinib | 10uM | 6h | PHF10 | 0.00 | 0.39 |
| HT29_13_GR_ChDB_0120 | Crizotinib | 10uM | 6h | PPIG | 0.00 | 0.36 |
| HT29_13_GR_ChDB_0120 | Crizotinib | 10uM | 6h | METTL1 | 0.00 | 0.49 |
| HT29_13_GR_ChDB_0120 | Crizotinib | 10uM | 6h | LAMTOR3 | 0.00 | 2.51 |
| HT29_13_GR_ChDB_0120 | Crizotinib | 10uM | 6h | TNIK | 0.00 | 0.43 |
| HT29_13_GR_ChDB_0120 | Crizotinib | 10uM | 6h | KITLG | 0.00 | 0.26 |
| HT29_13_GR_ChDB_0120 | Crizotinib | 10uM | 6h | HEATR1 | 0.00 | 0.41 |
| HT29_13_GR_ChDB_0120 | Crizotinib | 10uM | 6h | CYP3A5 | 0.00 | 3.47 |
| HT29_13_GR_ChDB_0120 | Crizotinib | 10uM | 6h | SMAD1 | 0.00 | 2.58 |
| HT29_13_GR_ChDB_0120 | Crizotinib | 10uM | 6h | ATP2A2 | 0.00 | 0.19 |
| HT29_13_GR_ChDB_0120 | Crizotinib | 10uM | 6h | SLC35A3 | 0.00 | 0.43 |
| HT29_13_GR_ChDB_0120 | Crizotinib | 10uM | 6h | KLF4 | 0.00 | 2.75 |
| HT29_13_GR_ChDB_0120 | Crizotinib | 10uM | 6h | DDX5 | 0.00 | 0.43 |
| HT29_13_GR_ChDB_0120 | Crizotinib | 10uM | 6h | EXOSC4 | 0.00 | 0.45 |
| HT29_13_GR_ChDB_0120 | Crizotinib | 10uM | 6h | CNOT7 | 0.00 | 2.86 |
| HT29_13_GR_ChDB_0120 | Crizotinib | 10uM | 6h | LGALS8 | 0.00 | 2.41 |
| HT29_13_GR_ChDB_0120 | Crizotinib | 10uM | 6h | CAMK2G | 0.00 | 0.46 |
| HT29_13_GR_ChDB_0120 | Crizotinib | 10uM | 6h | HEXIM1 | 0.00 | 0.42 |
| HT29_13_GR_ChDB_0120 | Crizotinib | 10uM | 6h | LRRC16A | 0.00 | 0.49 |
| HT29_13_GR_ChDB_0120 | Crizotinib | 10uM | 6h | CHUK | 0.00 | 0.43 |
| HT29_13_GR_ChDB_0120 | Crizotinib | 10uM | 6h | BCL2L11 | 0.00 | 2.20 |
| HT29_13_GR_ChDB_0120 | Crizotinib | 10uM | 6h | EGR1 | 0.00 | 2.48 |
| HT29_13_GR_ChDB_0120 | Crizotinib | 10uM | 6h | KLHDC2 | 0.00 | 0.39 |
| HT29_13_GR_ChDB_0120 | Crizotinib | 10uM | 6h | TNFRSF21 | 0.00 | 2.35 |
| HT29_13_GR_ChDB_0120 | Crizotinib | 10uM | 6h | HIF1AN | 0.00 | 0.33 |
| HT29_13_GR_ChDB_0120 | Crizotinib | 10uM | 6h | RAI14 | 0.00 | 0.22 |
| HT29_13_GR_ChDB_0120 | Crizotinib | 10uM | 6h | ADAT1 | 0.00 | 0.28 |
| HT29_13_GR_ChDB_0120 | Crizotinib | 10uM | 6h | CXCL3 | 0.00 | 2.78 |
| HT29_13_GR_ChDB_0120 | Crizotinib | 10uM | 6h | TMEM5 | 0.00 | 0.36 |
| HT29_13_GR_ChDB_0120 | Crizotinib | 10uM | 6h | MSH6 | 0.00 | 0.39 |
| HT29_13_GR_ChDB_0120 | Crizotinib | 10uM | 6h | SPTLC2 | 0.00 | 2.32 |
| HT29_13_GR_ChDB_0120 | Crizotinib | 10uM | 6h | CD59 | 0.00 | 2.26 |
| HT29_13_GR_ChDB_0120 | Crizotinib | 10uM | 6h | PURA | 0.00 | 0.32 |
| HT29_13_GR_ChDB_0120 | Crizotinib | 10uM | 6h | SIK3 | 0.00 | 0.39 |
| HT29_13_GR_ChDB_0120 | Crizotinib | 10uM | 6h | PRKCA | 0.00 | 0.43 |
| HT29_13_GR_ChDB_0120 | Crizotinib | 10uM | 6h | NR1D2 | 0.00 | 2.07 |
| HT29_13_GR_ChDB_0120 | Crizotinib | 10uM | 6h | PLA2G4A | 0.00 | 0.42 |
| HT29_13_GR_ChDB_0120 | Crizotinib | 10uM | 6h | BMPR2 | 0.00 | 0.46 |
| HT29_13_GR_ChDB_0120 | Crizotinib | 10uM | 6h | FAM114A2 | 0.00 | 0.48 |
| HT29_13_GR_ChDB_0120 | Crizotinib | 10uM | 6h | ATP7A | 0.00 | 0.49 |
| HT29_13_GR_ChDB_0120 | Crizotinib | 10uM | 6h | KIAA1033 | 0.00 | 0.49 |
| HT29_13_GR_ChDB_0120 | Crizotinib | 10uM | 6h | LARS2 | 0.00 | 0.47 |
| HT29_13_GR_ChDB_0120 | Crizotinib | 10uM | 6h | NEK6 | 0.00 | 0.42 |
| HT29_13_GR_ChDB_0120 | Crizotinib | 10uM | 6h | TAF1B | 0.00 | 0.35 |
| HT29_13_GR_ChDB_0120 | Crizotinib | 10uM | 6h | RBBP6 | 0.00 | 0.25 |
| HT29_13_GR_ChDB_0120 | Crizotinib | 10uM | 6h | NFIB | 0.00 | 0.26 |
| HT29_13_GR_ChDB_0120 | Crizotinib | 10uM | 6h | PIP4K2A | 0.00 | 0.33 |
| HT29_13_GR_ChDB_0120 | Crizotinib | 10uM | 6h | SBNO1 | 0.00 | 0.34 |
| HT29_13_GR_ChDB_0120 | Crizotinib | 10uM | 6h | EED | 0.00 | 0.50 |
| HT29_13_GR_ChDB_0120 | Crizotinib | 10uM | 6h | CLK1 | 0.00 | 3.45 |
| HT29_13_GR_ChDB_0120 | Crizotinib | 10uM | 6h | PLEKHG3 | 0.00 | 2.41 |
| HT29_13_GR_ChDB_0120 | Crizotinib | 10uM | 6h | HOXA10 | 0.00 | 0.42 |
| HT29_13_GR_ChDB_0120 | Crizotinib | 10uM | 6h | HMGCS2 | 0.00 | 0.40 |
| HT29_13_GR_ChDB_0120 | Crizotinib | 10uM | 6h | TANK | 0.00 | 2.22 |
| HT29_13_GR_ChDB_0120 | Crizotinib | 10uM | 6h | PRPF4B | 0.00 | 3.08 |
| HT29_13_GR_ChDB_0120 | Crizotinib | 10uM | 6h | F2RL1 | 0.00 | 2.03 |
| HT29_13_GR_ChDB_0120 | Crizotinib | 10uM | 6h | PIGB | 0.00 | 0.46 |
| HT29_13_GR_ChDB_0120 | Crizotinib | 10uM | 6h | PER2 | 0.00 | 0.47 |
| HT29_13_GR_ChDB_0120 | Crizotinib | 10uM | 6h | RFK | 0.00 | 0.49 |
| HT29_13_GR_ChDB_0120 | Crizotinib | 10uM | 6h | MYCBP2 | 0.00 | 0.32 |
| HT29_13_GR_ChDB_0120 | Crizotinib | 10uM | 6h | THADA | 0.00 | 0.44 |
| HT29_13_GR_ChDB_0120 | Crizotinib | 10uM | 6h | PEAK1 | 0.00 | 0.40 |
| HT29_13_GR_ChDB_0120 | Crizotinib | 10uM | 6h | ADO | 0.00 | 0.47 |
| HT29_13_GR_ChDB_0120 | Crizotinib | 10uM | 6h | CXADR | 0.00 | 3.44 |
| HT29_13_GR_ChDB_0120 | Crizotinib | 10uM | 6h | CHCHD7 | 0.00 | 0.21 |
| HT29_13_GR_ChDB_0120 | Crizotinib | 10uM | 6h | LIAS | 0.00 | 0.45 |
| HT29_13_GR_ChDB_0120 | Crizotinib | 10uM | 6h | PLXNA2 | 0.00 | 0.37 |
| HT29_13_GR_ChDB_0120 | Crizotinib | 10uM | 6h | SQLE | 0.00 | 3.03 |
| HT29_13_GR_ChDB_0120 | Crizotinib | 10uM | 6h | CD44 | 0.00 | 2.19 |
| HT29_13_GR_ChDB_0120 | Crizotinib | 10uM | 6h | TRIP13 | 0.00 | 0.47 |
| HT29_13_GR_ChDB_0120 | Crizotinib | 10uM | 6h | MLLT6 | 0.00 | 0.38 |
| HT29_13_GR_ChDB_0120 | Crizotinib | 10uM | 6h | PRKACB | 0.00 | 2.81 |
| HT29_13_GR_ChDB_0120 | Crizotinib | 10uM | 6h | NET1 | 0.00 | 2.78 |
| HT29_13_GR_ChDB_0120 | Crizotinib | 10uM | 6h | RRS1 | 0.00 | 0.33 |
| HT29_13_GR_ChDB_0120 | Crizotinib | 10uM | 6h | GPR126 | 0.00 | 0.41 |
| HT29_13_GR_ChDB_0120 | Crizotinib | 10uM | 6h | SYF2 | 0.00 | 4.64 |
| HT29_13_GR_ChDB_0120 | Crizotinib | 10uM | 6h | MAP3K2 | 0.00 | 0.46 |
| HT29_13_GR_ChDB_0120 | Crizotinib | 10uM | 6h | KLF12 | 0.00 | 2.38 |
| HT29_13_GR_ChDB_0120 | Crizotinib | 10uM | 6h | ERLIN1 | 0.00 | 0.47 |
| HT29_13_GR_ChDB_0120 | Crizotinib | 10uM | 6h | PHF17 | 0.00 | 0.39 |
| HT29_13_GR_ChDB_0120 | Crizotinib | 10uM | 6h | ARGLU1 | 0.00 | 0.44 |
| HT29_13_GR_ChDB_0120 | Crizotinib | 10uM | 6h | SLC25A46 | 0.00 | 0.35 |
| HT29_13_GR_ChDB_0120 | Crizotinib | 10uM | 6h | GFOD1 | 0.00 | 0.47 |
| HT29_13_GR_ChDB_0120 | Crizotinib | 10uM | 6h | ZNF24 | 0.00 | 2.43 |
| HT29_13_GR_ChDB_0120 | Crizotinib | 10uM | 6h | WHSC1L1 | 0.00 | 3.56 |
| HT29_13_GR_ChDB_0120 | Crizotinib | 10uM | 6h | SLC16A1 | 0.00 | 0.44 |
| HT29_13_GR_ChDB_0120 | Crizotinib | 10uM | 6h | PPIF | 0.00 | 0.36 |
| HT29_13_GR_ChDB_0120 | Crizotinib | 10uM | 6h | LMO4 | 0.00 | 0.35 |
| HT29_13_GR_ChDB_0120 | Crizotinib | 10uM | 6h | RRP8 | 0.00 | 0.41 |
| HT29_13_GR_ChDB_0120 | Crizotinib | 10uM | 6h | MEIS2 | 0.00 | 0.33 |
| HT29_13_GR_ChDB_0120 | Crizotinib | 10uM | 6h | SMARCA2 | 0.00 | 0.49 |
| HT29_13_GR_ChDB_0120 | Crizotinib | 10uM | 6h | GGPS1 | 0.00 | 0.44 |
| HT29_13_GR_ChDB_0120 | Crizotinib | 10uM | 6h | RAD51C | 0.00 | 0.36 |
| HT29_13_GR_ChDB_0120 | Crizotinib | 10uM | 6h | AKTIP | 0.00 | 0.39 |
| HT29_13_GR_ChDB_0120 | Crizotinib | 10uM | 6h | POLA2 | 0.00 | 0.42 |
| HT29_13_GR_ChDB_0120 | Crizotinib | 10uM | 6h | FTSJ1 | 0.00 | 0.46 |
| HT29_13_GR_ChDB_0120 | Crizotinib | 10uM | 6h | TCF7L2 | 0.00 | 2.25 |
| HT29_13_GR_ChDB_0120 | Crizotinib | 10uM | 6h | LAMB1 | 0.00 | 0.44 |
| HT29_13_GR_ChDB_0120 | Crizotinib | 10uM | 6h | RRP12 | 0.00 | 0.46 |
| HT29_13_GR_ChDB_0120 | Crizotinib | 10uM | 6h | PIM1 | 0.00 | 3.20 |
| HT29_13_GR_ChDB_0120 | Crizotinib | 10uM | 6h | YTHDF2 | 0.00 | 2.14 |
| HT29_13_GR_ChDB_0120 | Crizotinib | 10uM | 6h | SMAD6 | 0.00 | 0.38 |
| HT29_13_GR_ChDB_0120 | Crizotinib | 10uM | 6h | B3GNT1 | 0.00 | 0.48 |
| HT29_13_GR_ChDB_0120 | Crizotinib | 10uM | 6h | VDR | 0.00 | 0.30 |
| HT29_13_GR_ChDB_0120 | Crizotinib | 10uM | 6h | GPR110 | 0.00 | 0.48 |
| HT29_13_GR_ChDB_0120 | Crizotinib | 10uM | 6h | HERPUD1 | 0.00 | 2.09 |
| HT29_13_GR_ChDB_0120 | Crizotinib | 10uM | 6h | CAMSAP2 | 0.00 | 0.49 |
| HT29_13_GR_ChDB_0120 | Crizotinib | 10uM | 6h | KDM4C | 0.00 | 0.48 |
| HT29_13_GR_ChDB_0120 | Crizotinib | 10uM | 6h | OPA1 | 0.00 | 0.43 |
| HT29_13_GR_ChDB_0120 | Crizotinib | 10uM | 6h | MED28 | 0.00 | 2.61 |
| HT29_13_GR_ChDB_0120 | Crizotinib | 10uM | 6h | PTK2 | 0.00 | 2.10 |
| HT29_13_GR_ChDB_0120 | Crizotinib | 10uM | 6h | SRSF4 | 0.00 | 2.11 |
| HT29_13_GR_ChDB_0120 | Crizotinib | 10uM | 6h | PMS1 | 0.00 | 0.16 |
| HT29_13_GR_ChDB_0120 | Crizotinib | 10uM | 6h | CDC45 | 0.00 | 0.37 |
| HT29_13_GR_ChDB_0120 | Crizotinib | 10uM | 6h | FAM20B | 0.00 | 0.47 |
| HT29_13_GR_ChDB_0120 | Crizotinib | 10uM | 6h | ITGB1BP1 | 0.00 | 0.40 |
| HT29_13_GR_ChDB_0120 | Crizotinib | 10uM | 6h | DPH2 | 0.00 | 0.30 |
| HT29_13_GR_ChDB_0120 | Crizotinib | 10uM | 6h | FDX1 | 0.00 | 0.39 |
| HT29_13_GR_ChDB_0120 | Crizotinib | 10uM | 6h | HMOX2 | 0.00 | 0.37 |
| HT29_13_GR_ChDB_0120 | Crizotinib | 10uM | 6h | SLC16A5 | 0.00 | 0.18 |
| HT29_13_GR_ChDB_0120 | Crizotinib | 10uM | 6h | E2F8 | 0.00 | 0.45 |
| HT29_13_GR_ChDB_0120 | Crizotinib | 10uM | 6h | PDS5B | 0.00 | 0.40 |
| HT29_13_GR_ChDB_0120 | Crizotinib | 10uM | 6h | MTAP | 0.00 | 0.23 |
| HT29_13_GR_ChDB_0120 | Crizotinib | 10uM | 6h | COG2 | 0.00 | 0.34 |
| HT29_13_GR_ChDB_0120 | Crizotinib | 10uM | 6h | FBXW7 | 0.00 | 2.05 |
| HT29_13_GR_ChDB_0120 | Crizotinib | 10uM | 6h | CCNB1 | 0.00 | 0.35 |
| HT29_13_GR_ChDB_0120 | Crizotinib | 10uM | 6h | GOLIM4 | 0.00 | 0.47 |
| HT29_13_GR_ChDB_0120 | Crizotinib | 10uM | 6h | CEP55 | 0.00 | 0.48 |
| HT29_13_GR_ChDB_0120 | Crizotinib | 10uM | 6h | POLG2 | 0.00 | 0.49 |
| HT29_13_GR_ChDB_0120 | Crizotinib | 10uM | 6h | CYP1B1 | 0.00 | 0.33 |
| HT29_13_GR_ChDB_0120 | Crizotinib | 10uM | 6h | UBAP2L | 0.00 | 0.44 |
| HT29_13_GR_ChDB_0120 | Crizotinib | 10uM | 6h | PREB | 0.00 | 0.44 |
| HT29_13_GR_ChDB_0120 | Crizotinib | 10uM | 6h | AFF1 | 0.00 | 0.20 |
| HT29_13_GR_ChDB_0120 | Crizotinib | 10uM | 6h | HNF1B | 0.00 | 0.35 |
| HT29_13_GR_ChDB_0120 | Crizotinib | 10uM | 6h | TBC1D2B | 0.00 | 0.39 |
| HT29_13_GR_ChDB_0120 | Crizotinib | 10uM | 6h | ARNTL2 | 0.00 | 0.47 |
| HT29_13_GR_ChDB_0120 | Crizotinib | 10uM | 6h | AAK1 | 0.00 | 0.37 |
| HT29_13_GR_ChDB_0120 | Crizotinib | 10uM | 6h | LIPH | 0.00 | 6.18 |
| HT29_13_GR_ChDB_0120 | Crizotinib | 10uM | 6h | PRDX2 | 0.00 | 2.17 |
| HT29_13_GR_ChDB_0120 | Crizotinib | 10uM | 6h | RNMT | 0.00 | 2.85 |
| HT29_13_GR_ChDB_0120 | Crizotinib | 10uM | 6h | PPM1B | 0.00 | 3.20 |
| HT29_13_GR_ChDB_0120 | Crizotinib | 10uM | 6h | PRKAG2 | 0.00 | 0.44 |
| HT29_13_GR_ChDB_0120 | Crizotinib | 10uM | 6h | THAP11 | 0.00 | 0.45 |
| HT29_13_GR_ChDB_0120 | Crizotinib | 10uM | 6h | RRP1B | 0.00 | 0.21 |
| HT29_13_GR_ChDB_0120 | Crizotinib | 10uM | 6h | TRERF1 | 0.00 | 0.43 |
| HT29_13_GR_ChDB_0120 | Crizotinib | 10uM | 6h | SLC25A32 | 0.00 | 0.15 |
| HT29_13_GR_ChDB_0120 | Crizotinib | 10uM | 6h | SLC6A8 | 0.00 | 0.43 |
| HT29_13_GR_ChDB_0120 | Crizotinib | 10uM | 6h | STK38L | 0.00 | 0.34 |
| HT29_13_GR_ChDB_0120 | Crizotinib | 10uM | 6h | PPARGC1B | 0.00 | 0.44 |
| HT29_13_GR_ChDB_0120 | Crizotinib | 10uM | 6h | ID1 | 0.00 | 0.43 |
| HT29_13_GR_ChDB_0120 | Crizotinib | 10uM | 6h | DNAJC6 | 0.00 | 0.28 |
| HT29_13_GR_ChDB_0120 | Crizotinib | 10uM | 6h | RRAGC | 0.00 | 4.83 |
| HT29_13_GR_ChDB_0120 | Crizotinib | 10uM | 6h | MAT2A | 0.00 | 0.22 |
| HT29_13_GR_ChDB_0120 | Crizotinib | 10uM | 6h | ERCC3 | 0.00 | 0.45 |
| HT29_13_GR_ChDB_0120 | Crizotinib | 10uM | 6h | XK | 0.00 | 0.28 |
| HT29_13_GR_ChDB_0120 | Crizotinib | 10uM | 6h | WDR67 | 0.00 | 0.26 |
| HT29_13_GR_ChDB_0120 | Crizotinib | 10uM | 6h | PIAS1 | 0.00 | 2.06 |
| HT29_13_GR_ChDB_0120 | Crizotinib | 10uM | 6h | ABCC10 | 0.00 | 0.46 |
| HT29_13_GR_ChDB_0120 | Crizotinib | 10uM | 6h | PPP3CB | 0.00 | 0.43 |
| HT29_13_GR_ChDB_0120 | Crizotinib | 10uM | 6h | NAT10 | 0.00 | 0.33 |
| HT29_13_GR_ChDB_0120 | Crizotinib | 10uM | 6h | PRSS2 | 0.00 | 2.27 |
| HT29_13_GR_ChDB_0120 | Crizotinib | 10uM | 6h | ABCC3 | 0.00 | 0.47 |
| HT29_13_GR_ChDB_0120 | Crizotinib | 10uM | 6h | TRAM2 | 0.00 | 0.41 |
| HT29_13_GR_ChDB_0120 | Crizotinib | 10uM | 6h | LNPEP | 0.00 | 0.37 |
| HT29_13_GR_ChDB_0120 | Crizotinib | 10uM | 6h | ATP6V1D | 0.00 | 2.01 |
| HT29_13_GR_ChDB_0120 | Crizotinib | 10uM | 6h | NF2 | 0.00 | 0.34 |
| HT29_13_GR_ChDB_0120 | Crizotinib | 10uM | 6h | RAB11FIP2 | 0.00 | 0.35 |
| HT29_13_GR_ChDB_0120 | Crizotinib | 10uM | 6h | TCEB3 | 0.00 | 0.40 |
| HT29_13_GR_ChDB_0120 | Crizotinib | 10uM | 6h | CXCL2 | 0.00 | 2.45 |
| HT29_13_GR_ChDB_0120 | Crizotinib | 10uM | 6h | RAD1 | 0.00 | 0.46 |
| HT29_13_GR_ChDB_0120 | Crizotinib | 10uM | 6h | MED21 | 0.00 | 2.26 |
| HT29_13_GR_ChDB_0120 | Crizotinib | 10uM | 6h | MDM4 | 0.00 | 0.37 |
| HT29_13_GR_ChDB_0120 | Crizotinib | 10uM | 6h | EPB41L4B | 0.00 | 0.36 |
| HT29_13_GR_ChDB_0120 | Crizotinib | 10uM | 6h | KIAA1804 | 0.00 | 0.20 |
| HT29_13_GR_ChDB_0120 | Crizotinib | 10uM | 6h | ATG12 | 0.00 | 0.49 |
| HT29_13_GR_ChDB_0120 | Crizotinib | 10uM | 6h | IFIT5 | 0.00 | 0.39 |
| HT29_13_GR_ChDB_0120 | Crizotinib | 10uM | 6h | SOS1 | 0.00 | 0.36 |
| HT29_13_GR_ChDB_0120 | Crizotinib | 10uM | 6h | NMI | 0.00 | 0.50 |
| HT29_13_GR_ChDB_0120 | Crizotinib | 10uM | 6h | GCH1 | 0.00 | 0.50 |
| HT29_13_GR_ChDB_0120 | Crizotinib | 10uM | 6h | ACTR3 | 0.00 | 0.43 |
| HT29_13_GR_ChDB_0120 | Crizotinib | 10uM | 6h | PHF13 | 0.00 | 2.01 |
| HT29_13_GR_ChDB_0120 | Crizotinib | 10uM | 6h | USP15 | 0.00 | 0.47 |
| HT29_13_GR_ChDB_0120 | Crizotinib | 10uM | 6h | GTF3C2 | 0.00 | 0.41 |
| HT29_13_GR_ChDB_0120 | Crizotinib | 10uM | 6h | AKR1C3 | 0.00 | 2.39 |
| HT29_13_GR_ChDB_0120 | Crizotinib | 10uM | 6h | BAMBI | 0.00 | 0.43 |
| HT29_13_GR_ChDB_0120 | Crizotinib | 10uM | 6h | AKAP11 | 0.00 | 0.18 |
| HT29_13_GR_ChDB_0120 | Crizotinib | 10uM | 6h | MAP3K7 | 0.00 | 0.47 |
| HT29_13_GR_ChDB_0120 | Crizotinib | 10uM | 6h | SOCS4 | 0.00 | 0.37 |
| HT29_13_GR_ChDB_0120 | Crizotinib | 10uM | 6h | PCGF5 | 0.00 | 0.34 |
| HT29_13_GR_ChDB_0120 | Crizotinib | 10uM | 6h | HLA-A | 0.00 | 2.11 |
| HT29_13_GR_ChDB_0120 | Crizotinib | 10uM | 6h | TFCP2 | 0.00 | 0.41 |
| HT29_13_GR_ChDB_0120 | Crizotinib | 10uM | 6h | SUPV3L1 | 0.00 | 0.48 |
| HT29_13_GR_ChDB_0120 | Crizotinib | 10uM | 6h | ZDHHC6 | 0.00 | 0.44 |
| HT29_13_GR_ChDB_0120 | Crizotinib | 10uM | 6h | ABHD6 | 0.00 | 0.50 |
| HT29_13_GR_ChDB_0120 | Crizotinib | 10uM | 6h | FKBP14 | 0.00 | 0.43 |
| HT29_13_GR_ChDB_0120 | Crizotinib | 10uM | 6h | JAG1 | 0.00 | 0.42 |
| HT29_13_GR_ChDB_0120 | Crizotinib | 10uM | 6h | SACM1L | 0.00 | 0.48 |
| HT29_13_GR_ChDB_0120 | Crizotinib | 10uM | 6h | PPFIBP2 | 0.00 | 0.37 |
| HT29_13_GR_ChDB_0120 | Crizotinib | 10uM | 6h | NR2C1 | 0.00 | 0.43 |
| HT29_13_GR_ChDB_0120 | Crizotinib | 10uM | 6h | SOX4 | 0.00 | 3.52 |
| HT29_13_GR_ChDB_0120 | Crizotinib | 10uM | 6h | PDLIM5 | 0.00 | 2.17 |
| HT29_13_GR_ChDB_0120 | Crizotinib | 10uM | 6h | YME1L1 | 0.00 | 2.11 |
| HT29_13_GR_ChDB_0120 | Crizotinib | 10uM | 6h | KDM5B | 0.00 | 3.10 |
| HT29_13_GR_ChDB_0120 | Crizotinib | 10uM | 6h | RIOK3 | 0.00 | 3.55 |
| HT29_13_GR_ChDB_0120 | Crizotinib | 10uM | 6h | PSIP1 | 0.00 | 0.50 |
| HT29_13_GR_ChDB_0120 | Crizotinib | 10uM | 6h | CCDC93 | 0.00 | 2.24 |
| HT29_13_GR_ChDB_0120 | Crizotinib | 10uM | 6h | KDM5A | 0.00 | 2.31 |
| HT29_13_GR_ChDB_0120 | Crizotinib | 10uM | 6h | SPTBN1 | 0.00 | 4.12 |
| HT29_13_GR_ChDB_0120 | Crizotinib | 10uM | 6h | SRSF7 | 0.00 | 0.50 |
| HT29_13_GR_ChDB_0120 | Crizotinib | 10uM | 6h | WSB1 | 0.00 | 2.08 |
| HT29_13_GR_ChDB_0120 | Crizotinib | 10uM | 6h | APPBP2 | 0.00 | 2.48 |
| HT29_13_GR_ChDB_0120 | Crizotinib | 10uM | 6h | ANKRD12 | 0.00 | 3.14 |
| HT29_13_GR_ChDB_0120 | Crizotinib | 10uM | 6h | DNAJB12 | 0.00 | 2.01 |
| HT29_13_GR_ChDB_0120 | Crizotinib | 10uM | 6h | ITCH | 0.00 | 2.08 |
| HT29_13_GR_ChDB_0120 | Crizotinib | 10uM | 6h | WDR48 | 0.00 | 2.83 |
| HT29_13_GR_ChDB_0120 | Crizotinib | 10uM | 6h | ZMYM4 | 0.00 | 0.42 |
| HT29_13_GR_ChDB_0120 | Crizotinib | 10uM | 6h | DYRK1A | 0.00 | 2.27 |
| HT29_13_GR_ChDB_0120 | Crizotinib | 10uM | 6h | MED13L | 0.00 | 2.38 |
| HT29_13_GR_ChDB_0120 | Crizotinib | 10uM | 6h | CHMP1B | 0.00 | 3.46 |
| HT29_13_GR_ChDB_0120 | Crizotinib | 10uM | 6h | CYTH1 | 0.00 | 2.98 |
| HT29_13_GR_ChDB_0120 | Crizotinib | 10uM | 6h | UBE2B | 0.00 | 2.21 |
| HT29_13_GR_ChDB_0120 | Crizotinib | 10uM | 6h | GFPT1 | 0.00 | 2.17 |
| HT29_13_GR_ChDB_0120 | Crizotinib | 10uM | 6h | CELF1 | 0.00 | 2.43 |
| HT29_13_GR_ChDB_0120 | Crizotinib | 10uM | 6h | EIF2S1 | 0.00 | 0.47 |
| HT29_13_GR_ChDB_0120 | Crizotinib | 10uM | 6h | ANAPC5 | 0.00 | 0.44 |
| HT29_13_GR_ChDB_0120 | Crizotinib | 10uM | 6h | METTL13 | 0.00 | 0.42 |
| HT29_13_GR_ChDB_0120 | Crizotinib | 10uM | 6h | C12orf52 | 0.00 | 0.36 |
| HT29_13_GR_ChDB_0120 | Crizotinib | 10uM | 6h | EXOSC2 | 0.00 | 0.38 |
| HT29_13_GR_ChDB_0120 | Crizotinib | 10uM | 6h | BRAP | 0.00 | 2.02 |
| HT29_13_GR_ChDB_0120 | Crizotinib | 10uM | 6h | SCAMP1 | 0.00 | 2.86 |
| HT29_13_GR_ChDB_0120 | Crizotinib | 10uM | 6h | PAWR | 0.00 | 3.08 |
| HT29_13_GR_ChDB_0120 | Crizotinib | 10uM | 6h | NIPBL | 0.00 | 2.27 |
| HT29_13_GR_ChDB_0120 | Crizotinib | 10uM | 6h | LDLR | 0.00 | 3.71 |
| HT29_13_GR_ChDB_0120 | Crizotinib | 10uM | 6h | MARCKS | 0.00 | 5.46 |
| HT29_13_GR_ChDB_0120 | Crizotinib | 10uM | 6h | DDX3X | 0.00 | 2.50 |
| HT29_13_GR_ChDB_0120 | Crizotinib | 10uM | 6h | ANGEL2 | 0.00 | 0.20 |
| HT29_13_GR_ChDB_0120 | Crizotinib | 10uM | 6h | RFC3 | 0.00 | 0.41 |
| HT29_13_GR_ChDB_0120 | Crizotinib | 10uM | 6h | SKP2 | 0.00 | 0.22 |
| HT29_13_GR_ChDB_0120 | Crizotinib | 10uM | 6h | MCAM | 0.00 | 0.26 |
| HT29_13_GR_ChDB_0120 | Crizotinib | 10uM | 6h | ZFX | 0.00 | 2.64 |
| HT29_13_GR_ChDB_0120 | Crizotinib | 10uM | 6h | NUP50 | 0.00 | 2.53 |
| HT29_13_GR_ChDB_0120 | Crizotinib | 10uM | 6h | KCNK1 | 0.00 | 7.48 |
| HT29_13_GR_ChDB_0120 | Crizotinib | 10uM | 6h | DR1 | 0.00 | 2.03 |
| HT29_13_GR_ChDB_0120 | Crizotinib | 10uM | 6h | CREBZF | 0.00 | 3.52 |
| HT29_13_GR_ChDB_0120 | Crizotinib | 10uM | 6h | AVL9 | 0.00 | 3.97 |
| HT29_13_GR_ChDB_0120 | Crizotinib | 10uM | 6h | TRIM14 | 0.00 | 0.36 |
| HT29_13_GR_ChDB_0120 | Crizotinib | 10uM | 6h | TM4SF1 | 0.00 | 3.89 |
| HT29_13_GR_ChDB_0120 | Crizotinib | 10uM | 6h | BAZ2A | 0.00 | 2.08 |
| HT29_13_GR_ChDB_0120 | Crizotinib | 10uM | 6h | SS18 | 0.00 | 2.62 |
| HT29_13_GR_ChDB_0120 | Crizotinib | 10uM | 6h | PPFIA1 | 0.00 | 3.42 |
| HT29_13_GR_ChDB_0120 | Crizotinib | 10uM | 6h | ZFAND5 | 0.00 | 2.76 |
| HT29_13_GR_ChDB_0120 | Crizotinib | 10uM | 6h | PTGS2 | 0.00 | 4.27 |
| HT29_13_GR_ChDB_0120 | Crizotinib | 10uM | 6h | IL6ST | 0.00 | 2.58 |
| HT29_13_GR_ChDB_0120 | Crizotinib | 10uM | 6h | KLHL24 | 0.00 | 8.48 |
| HT29_13_GR_ChDB_0120 | Crizotinib | 10uM | 6h | TFE3 | 0.00 | 2.02 |
| HT29_13_GR_ChDB_0120 | Crizotinib | 10uM | 6h | SMURF1 | 0.00 | 2.99 |
| HT29_13_GR_ChDB_0120 | Crizotinib | 10uM | 6h | FXR1 | 0.00 | 2.87 |
| HT29_13_GR_ChDB_0120 | Crizotinib | 10uM | 6h | HBP1 | 0.00 | 14.33 |
| HT29_13_GR_ChDB_0120 | Crizotinib | 10uM | 6h | WDFY3 | 0.00 | 3.30 |
| HT29_13_GR_ChDB_0120 | Crizotinib | 10uM | 6h | RMND5A | 0.00 | 4.99 |
| HT29_13_GR_ChDB_0120 | Crizotinib | 10uM | 6h | DUSP1 | 0.00 | 8.76 |
| HT29_13_GR_ChDB_0120 | Crizotinib | 10uM | 6h | LPIN1 | 0.00 | 2.52 |
| HT29_13_GR_ChDB_0120 | Crizotinib | 10uM | 6h | GULP1 | 0.00 | 4.70 |
| HT29_13_GR_ChDB_0120 | Crizotinib | 10uM | 6h | TRAK1 | 0.00 | 2.93 |
| HT29_13_GR_ChDB_0120 | Crizotinib | 10uM | 6h | NSMCE4A | 0.00 | 0.31 |
| HT29_13_GR_ChDB_0120 | Crizotinib | 10uM | 6h | ADAM17 | 0.00 | 2.31 |
| HT29_13_GR_ChDB_0120 | Crizotinib | 10uM | 6h | AAMDC | 0.00 | 2.34 |
| HT29_13_GR_ChDB_0120 | Crizotinib | 10uM | 6h | ENSA | 0.00 | 4.24 |
| HT29_13_GR_ChDB_0120 | Crizotinib | 10uM | 6h | MAP1LC3B | 0.00 | 3.79 |
| HT29_13_GR_ChDB_0120 | Crizotinib | 10uM | 6h | PTP4A1 | 0.00 | 2.15 |
| HT29_13_GR_ChDB_0120 | Crizotinib | 10uM | 6h | HNRNPDL | 0.00 | 2.18 |
| HT29_13_GR_ChDB_0120 | Crizotinib | 10uM | 6h | ORC5 | 0.00 | 0.34 |
| HT29_13_GR_ChDB_0120 | Crizotinib | 10uM | 6h | CDC27 | 0.00 | 0.42 |
| HT29_13_GR_ChDB_0120 | Crizotinib | 10uM | 6h | GART | 0.00 | 0.43 |
| HT29_13_GR_ChDB_0120 | Crizotinib | 10uM | 6h | PTBP3 | 0.00 | 2.60 |
| HT29_13_GR_ChDB_0120 | Crizotinib | 10uM | 6h | TNIP1 | 0.00 | 2.78 |
| HT29_13_GR_ChDB_0120 | Crizotinib | 10uM | 6h | FOXJ3 | 0.00 | 15.32 |
| HT29_13_GR_ChDB_0120 | Crizotinib | 10uM | 6h | BNIP3L | 0.00 | 2.36 |
| HT29_13_GR_ChDB_0120 | Crizotinib | 10uM | 6h | GJB3 | 0.00 | 2.12 |
| HT29_13_GR_ChDB_0120 | Crizotinib | 10uM | 6h | EIF1 | 0.00 | 2.03 |
| HT29_13_GR_ChDB_0120 | Crizotinib | 10uM | 6h | CEBPD | 0.00 | 2.38 |
| HT29_13_GR_ChDB_0120 | Crizotinib | 10uM | 6h | STK17B | 0.00 | 5.82 |
| HT29_13_GR_ChDB_0120 | Crizotinib | 10uM | 6h | EPS15L1 | 0.00 | 2.15 |
| HT29_13_GR_ChDB_0120 | Crizotinib | 10uM | 6h | BCL3 | 0.00 | 6.44 |
| HT29_13_GR_ChDB_0120 | Crizotinib | 10uM | 6h | ELF1 | 0.00 | 2.33 |
| HT29_13_GR_ChDB_0120 | Crizotinib | 10uM | 6h | WIPI2 | 0.00 | 2.24 |
| HT29_13_GR_ChDB_0120 | Crizotinib | 10uM | 6h | FANCI | 0.00 | 0.42 |
| HT29_13_GR_ChDB_0120 | Crizotinib | 10uM | 6h | CAND1 | 0.00 | 0.44 |
| HT29_13_GR_ChDB_0120 | Crizotinib | 10uM | 6h | METTL3 | 0.00 | 0.14 |
| HT29_13_GR_ChDB_0120 | Crizotinib | 10uM | 6h | DNAJA3 | 0.00 | 0.44 |
| HT29_13_GR_ChDB_0120 | Crizotinib | 10uM | 6h | TROVE2 | 0.00 | 0.20 |
| HT29_13_GR_ChDB_0120 | Crizotinib | 10uM | 6h | PEX3 | 0.00 | 0.38 |
| HT29_13_GR_ChDB_0120 | Crizotinib | 10uM | 6h | CCNE2 | 0.00 | 0.34 |
| HT29_13_GR_ChDB_0120 | Crizotinib | 10uM | 6h | OPTN | 0.00 | 3.71 |
| HT29_13_GR_ChDB_0120 | Crizotinib | 10uM | 6h | DNAJB9 | 0.00 | 2.66 |
| HT29_13_GR_ChDB_0120 | Crizotinib | 10uM | 6h | ELF3 | 0.00 | 7.78 |
| HT29_13_GR_ChDB_0120 | Crizotinib | 10uM | 6h | UBXN7 | 0.00 | 4.19 |
| HT29_13_GR_ChDB_0120 | Crizotinib | 10uM | 6h | GLG1 | 0.00 | 2.21 |
| HT29_13_GR_ChDB_0120 | Crizotinib | 10uM | 6h | ARPP19 | 0.00 | 2.83 |
| HT29_13_GR_ChDB_0120 | Crizotinib | 10uM | 6h | C10orf118 | 0.00 | 3.10 |
| HT29_13_GR_ChDB_0120 | Crizotinib | 10uM | 6h | REL | 0.00 | 2.19 |
| HT29_13_GR_ChDB_0120 | Crizotinib | 10uM | 6h | CTBP2 | 0.00 | 2.06 |
| HT29_13_GR_ChDB_0120 | Crizotinib | 10uM | 6h | OSBP | 0.00 | 3.04 |
| HT29_13_GR_ChDB_0120 | Crizotinib | 10uM | 6h | SLC11A2 | 0.00 | 0.38 |
| HT29_13_GR_ChDB_0120 | Crizotinib | 10uM | 6h | ZC3H14 | 0.00 | 0.46 |
| HT29_13_GR_ChDB_0120 | Crizotinib | 10uM | 6h | TSR1 | 0.00 | 0.41 |
| HT29_13_GR_ChDB_0120 | Crizotinib | 10uM | 6h | LBR | 0.00 | 0.38 |
| HT29_13_GR_ChDB_0120 | Crizotinib | 10uM | 6h | CCNT2 | 0.00 | 2.76 |
| HT29_13_GR_ChDB_0120 | Crizotinib | 10uM | 6h | RBM48 | 0.00 | 2.45 |
| HT29_13_GR_ChDB_0120 | Crizotinib | 10uM | 6h | CDC42EP2 | 0.00 | 2.50 |
| HT29_13_GR_ChDB_0120 | Crizotinib | 10uM | 6h | SLC25A37 | 0.00 | 2.04 |
| HT29_13_GR_ChDB_0120 | Crizotinib | 10uM | 6h | ZNF609 | 0.00 | 2.00 |
| HT29_13_GR_ChDB_0120 | Crizotinib | 10uM | 6h | RQCD1 | 0.00 | 2.69 |
| HT29_13_GR_ChDB_0120 | Crizotinib | 10uM | 6h | CCNG2 | 0.00 | 20.82 |
| HT29_13_GR_ChDB_0120 | Crizotinib | 10uM | 6h | PMAIP1 | 0.00 | 10.16 |
| HT29_13_GR_ChDB_0120 | Crizotinib | 10uM | 6h | OSBPL2 | 0.00 | 2.70 |
| HT29_13_GR_ChDB_0120 | Crizotinib | 10uM | 6h | ARID4B | 0.00 | 3.97 |
| HT29_13_GR_ChDB_0120 | Crizotinib | 10uM | 6h | WTAP | 0.00 | 2.47 |
| HT29_13_GR_ChDB_0120 | Crizotinib | 10uM | 6h | WAPAL | 0.00 | 2.72 |
| HT29_13_GR_ChDB_0120 | Crizotinib | 10uM | 6h | FAM134A | 0.00 | 2.04 |
| HT29_13_GR_ChDB_0120 | Crizotinib | 10uM | 6h | SLC19A1 | 0.00 | 0.26 |
| HT29_13_GR_ChDB_0120 | Crizotinib | 10uM | 6h | NUFIP1 | 0.00 | 0.39 |
| HT29_13_GR_ChDB_0120 | Crizotinib | 10uM | 6h | SLC25A12 | 0.00 | 0.35 |
| HT29_13_GR_ChDB_0120 | Crizotinib | 10uM | 6h | C2CD5 | 0.00 | 0.31 |
| HT29_13_GR_ChDB_0120 | Crizotinib | 10uM | 6h | DHX30 | 0.00 | 0.44 |
| HT29_13_GR_ChDB_0120 | Crizotinib | 10uM | 6h | SDC1 | 0.00 | 0.46 |
| HT29_13_GR_ChDB_0120 | Crizotinib | 10uM | 6h | DDX6 | 0.00 | 2.43 |
| HT29_13_GR_ChDB_0120 | Crizotinib | 10uM | 6h | ITGB8 | 0.00 | 4.12 |
| HT29_13_GR_ChDB_0120 | Crizotinib | 10uM | 6h | CTDSP2 | 0.00 | 5.20 |
| HT29_13_GR_ChDB_0120 | Crizotinib | 10uM | 6h | WASL | 0.00 | 3.57 |
| HT29_13_GR_ChDB_0120 | Crizotinib | 10uM | 6h | NFAT5 | 0.00 | 2.38 |
| HT29_13_GR_ChDB_0120 | Crizotinib | 10uM | 6h | NBR1 | 0.00 | 2.24 |
| HT29_13_GR_ChDB_0120 | Crizotinib | 10uM | 6h | SERINC1 | 0.00 | 3.88 |
| HT29_13_GR_ChDB_0120 | Crizotinib | 10uM | 6h | CTDSPL | 0.00 | 0.46 |
| HT29_13_GR_ChDB_0120 | Crizotinib | 10uM | 6h | TNPO2 | 0.00 | 0.49 |
| HT29_13_GR_ChDB_0120 | Crizotinib | 10uM | 6h | HSPA9 | 0.00 | 0.48 |
| HT29_13_GR_ChDB_0120 | Crizotinib | 10uM | 6h | NOP2 | 0.00 | 0.22 |
| HT29_13_GR_ChDB_0120 | Crizotinib | 10uM | 6h | MCM3AP | 0.00 | 0.45 |
| HT29_13_GR_ChDB_0120 | Crizotinib | 10uM | 6h | SEC23B | 0.00 | 0.48 |
| HT29_13_GR_ChDB_0120 | Crizotinib | 10uM | 6h | TIA1 | 0.00 | 0.36 |
| HT29_13_GR_ChDB_0120 | Crizotinib | 10uM | 6h | IPO7 | 0.00 | 0.32 |
| HT29_13_GR_ChDB_0120 | Crizotinib | 10uM | 6h | AGRN | 0.00 | 0.41 |
| HT29_13_GR_ChDB_0120 | Crizotinib | 10uM | 6h | GRSF1 | 0.00 | 0.43 |
| HT29_13_GR_ChDB_0120 | Crizotinib | 10uM | 6h | PNO1 | 0.00 | 0.40 |
| HT29_13_GR_ChDB_0120 | Crizotinib | 10uM | 6h | SSX2IP | 0.00 | 0.34 |
| HT29_13_GR_ChDB_0120 | Crizotinib | 10uM | 6h | GEMIN4 | 0.00 | 0.43 |
| HT29_13_GR_ChDB_0120 | Crizotinib | 10uM | 6h | RPL31 | 0.00 | 4.03 |
| HT29_13_GR_ChDB_0120 | Crizotinib | 10uM | 6h | LAMC2 | 0.00 | 2.34 |
| HT29_13_GR_ChDB_0120 | Crizotinib | 10uM | 6h | PHLDA1 | 0.00 | 2.11 |
| HT29_13_GR_ChDB_0120 | Crizotinib | 10uM | 6h | UBE2H | 0.00 | 2.96 |
| HT29_13_GR_ChDB_0120 | Crizotinib | 10uM | 6h | EEA1 | 0.00 | 2.68 |
| HT29_13_GR_ChDB_0120 | Crizotinib | 10uM | 6h | STX3 | 0.00 | 2.64 |
| HT29_13_GR_ChDB_0120 | Crizotinib | 10uM | 6h | RNASEH1 | 0.00 | 0.47 |
| HT29_13_GR_ChDB_0120 | Crizotinib | 10uM | 6h | FAM208A | 0.00 | 0.35 |
| HT29_13_GR_ChDB_0120 | Crizotinib | 10uM | 6h | MLEC | 0.00 | 0.49 |
| HT29_13_GR_ChDB_0120 | Crizotinib | 10uM | 6h | DST | 0.00 | 0.43 |
| HT29_13_GR_ChDB_0120 | Crizotinib | 10uM | 6h | COQ9 | 0.00 | 0.44 |
| HT29_13_GR_ChDB_0120 | Crizotinib | 10uM | 6h | FBXO9 | 0.00 | 0.34 |
| HT29_13_GR_ChDB_0120 | Crizotinib | 10uM | 6h | UBE2N | 0.00 | 0.43 |
| HT29_13_GR_ChDB_0120 | Crizotinib | 10uM | 6h | MBD4 | 0.00 | 0.40 |
| HT29_13_GR_ChDB_0120 | Crizotinib | 10uM | 6h | SHQ1 | 0.00 | 0.40 |
| HT29_13_GR_ChDB_0120 | Crizotinib | 10uM | 6h | KIF20B | 0.00 | 0.46 |
| HT29_13_GR_ChDB_0120 | Crizotinib | 10uM | 6h | MRPS11 | 0.00 | 0.49 |
| HT29_13_GR_ChDB_0120 | Crizotinib | 10uM | 6h | YARS2 | 0.00 | 0.42 |
| HT29_13_GR_ChDB_0120 | Crizotinib | 10uM | 6h | GOLGB1 | 0.00 | 2.43 |
| HT29_13_GR_ChDB_0120 | Crizotinib | 10uM | 6h | BTG1 | 0.00 | 6.32 |
| HT29_13_GR_ChDB_0120 | Crizotinib | 10uM | 6h | TRIM23 | 0.00 | 3.42 |
| HT29_13_GR_ChDB_0120 | Crizotinib | 10uM | 6h | PLCL2 | 0.00 | 2.16 |
| HT29_13_GR_ChDB_0120 | Crizotinib | 10uM | 6h | PAPOLG | 0.00 | 3.47 |
| HT29_13_GR_ChDB_0120 | Crizotinib | 10uM | 6h | MKRN1 | 0.00 | 9.19 |
| HT29_13_GR_ChDB_0120 | Crizotinib | 10uM | 6h | BRWD1 | 0.00 | 2.65 |
| HT29_13_GR_ChDB_0120 | Crizotinib | 10uM | 6h | CCT5 | 0.00 | 0.50 |
| HT29_13_GR_ChDB_0120 | Crizotinib | 10uM | 6h | ZNF451 | 0.00 | 0.32 |
| HT29_13_GR_ChDB_0120 | Crizotinib | 10uM | 6h | ARFGEF2 | 0.00 | 0.43 |
| HT29_13_GR_ChDB_0120 | Crizotinib | 10uM | 6h | IVD | 0.00 | 0.37 |
| HT29_13_GR_ChDB_0120 | Crizotinib | 10uM | 6h | EIF3B | 0.00 | 0.49 |
| HT29_13_GR_ChDB_0120 | Crizotinib | 10uM | 6h | NFYB | 0.00 | 0.40 |
| HT29_13_GR_ChDB_0120 | Crizotinib | 10uM | 6h | LUC7L3 | 0.00 | 0.36 |
| HT29_13_GR_ChDB_0120 | Crizotinib | 10uM | 6h | DLG1 | 0.00 | 0.47 |
| HT29_13_GR_ChDB_0120 | Crizotinib | 10uM | 6h | DEGS1 | 0.00 | 0.38 |
| HT29_13_GR_ChDB_0120 | Crizotinib | 10uM | 6h | PDCL | 0.00 | 2.24 |
| HT29_13_GR_ChDB_0120 | Crizotinib | 10uM | 6h | MYB | 0.00 | 2.29 |
| HT29_13_GR_ChDB_0120 | Crizotinib | 10uM | 6h | MKL1 | 0.00 | 2.41 |
| HT29_13_GR_ChDB_0120 | Crizotinib | 10uM | 6h | AFF4 | 0.00 | 3.90 |
| HT29_13_GR_ChDB_0120 | Crizotinib | 10uM | 6h | OTUD4 | 0.00 | 3.06 |
| HT29_13_GR_ChDB_0120 | Crizotinib | 10uM | 6h | PCIF1 | 0.00 | 2.14 |
| HT29_13_GR_ChDB_0120 | Crizotinib | 10uM | 6h | VEZF1 | 0.00 | 3.02 |
| HT29_13_GR_ChDB_0120 | Crizotinib | 10uM | 6h | RSRC2 | 0.00 | 5.25 |
| HT29_13_GR_ChDB_0120 | Crizotinib | 10uM | 6h | F11R | 0.00 | 3.05 |
| HT29_13_GR_ChDB_0120 | Crizotinib | 10uM | 6h | BACH1 | 0.00 | 5.25 |
| HT29_13_GR_ChDB_0120 | Crizotinib | 10uM | 6h | NFKBIE | 0.00 | 3.03 |
| HT29_13_GR_ChDB_0120 | Crizotinib | 10uM | 6h | KDM6A | 0.00 | 2.53 |
| HT29_13_GR_ChDB_0120 | Crizotinib | 10uM | 6h | SCD | 0.00 | 2.67 |
| HT29_13_GR_ChDB_0120 | Crizotinib | 10uM | 6h | SIK2 | 0.00 | 2.01 |
| HT29_13_GR_ChDB_0120 | Crizotinib | 10uM | 6h | PRPF18 | 0.00 | 2.44 |
| HT29_13_GR_ChDB_0120 | Crizotinib | 10uM | 6h | ATG13 | 0.00 | 2.23 |
| HT29_13_GR_ChDB_0120 | Crizotinib | 10uM | 6h | WAC | 0.00 | 2.59 |
| HT29_13_GR_ChDB_0120 | Crizotinib | 10uM | 6h | GNAI1 | 0.00 | 4.98 |
| HT29_13_GR_ChDB_0120 | Crizotinib | 10uM | 6h | SKIL | 0.00 | 4.19 |
| HT29_13_GR_ChDB_0120 | Crizotinib | 10uM | 6h | E2F3 | 0.00 | 2.43 |
| HT29_13_GR_ChDB_0120 | Crizotinib | 10uM | 6h | POFUT2 | 0.00 | 2.10 |
| HT29_13_GR_ChDB_0120 | Crizotinib | 10uM | 6h | ZFP36 | 0.00 | 3.20 |
| HT29_13_GR_ChDB_0120 | Crizotinib | 10uM | 6h | CWC25 | 0.00 | 3.45 |
| HT29_13_GR_ChDB_0120 | Crizotinib | 10uM | 6h | GON4L | 0.00 | 3.49 |
| HT29_13_GR_ChDB_0120 | Crizotinib | 10uM | 6h | ZFP36L2 | 0.00 | 2.41 |
| HT29_13_GR_ChDB_0120 | Crizotinib | 10uM | 6h | AKAP10 | 0.00 | 2.68 |
| HT29_13_GR_ChDB_0120 | Crizotinib | 10uM | 6h | FARSA | 0.00 | 0.42 |
| HT29_13_GR_ChDB_0120 | Crizotinib | 10uM | 6h | METTL17 | 0.00 | 0.37 |
| HT29_13_GR_ChDB_0120 | Crizotinib | 10uM | 6h | YLPM1 | 0.00 | 0.40 |
| HT29_13_GR_ChDB_0120 | Crizotinib | 10uM | 6h | MAPKAPK5-AS1 | 0.00 | 0.43 |
| HT29_13_GR_ChDB_0120 | Crizotinib | 10uM | 6h | INTS7 | 0.00 | 0.37 |
| HT29_13_GR_ChDB_0120 | Crizotinib | 10uM | 6h | UBE2I | 0.00 | 0.43 |
| HT29_13_GR_ChDB_0120 | Crizotinib | 10uM | 6h | PSMD12 | 0.00 | 0.32 |
| HT29_13_GR_ChDB_0120 | Crizotinib | 10uM | 6h | CUL5 | 0.00 | 0.48 |
| HT29_13_GR_ChDB_0120 | Crizotinib | 10uM | 6h | NFATC2IP | 0.00 | 0.25 |
| HT29_13_GR_ChDB_0120 | Crizotinib | 10uM | 6h | NOC3L | 0.00 | 0.28 |
| HT29_13_GR_ChDB_0120 | Crizotinib | 10uM | 6h | CLCC1 | 0.00 | 0.32 |
| HT29_13_GR_ChDB_0120 | Crizotinib | 10uM | 6h | U2SURP | 0.00 | 0.34 |
| HT29_13_GR_ChDB_0120 | Crizotinib | 10uM | 6h | DCUN1D4 | 0.00 | 0.29 |
| HT29_13_GR_ChDB_0120 | Crizotinib | 10uM | 6h | MRPL34 | 0.00 | 0.49 |
| HT29_13_GR_ChDB_0120 | Crizotinib | 10uM | 6h | LARP4 | 0.00 | 0.31 |
| HT29_13_GR_ChDB_0120 | Crizotinib | 10uM | 6h | MTRR | 0.00 | 0.32 |
| HT29_13_GR_ChDB_0120 | Crizotinib | 10uM | 6h | TSEN2 | 0.00 | 0.29 |
| HT29_13_GR_ChDB_0120 | Crizotinib | 10uM | 6h | BAG4 | 0.00 | 2.12 |
| HT29_13_GR_ChDB_0120 | Crizotinib | 10uM | 6h | FKBP1A | 0.00 | 2.35 |
| HT29_13_GR_ChDB_0120 | Crizotinib | 10uM | 6h | MAFF | 0.00 | 7.74 |
| HT29_13_GR_ChDB_0120 | Crizotinib | 10uM | 6h | ZNF292 | 0.00 | 3.87 |
| HT29_13_GR_ChDB_0120 | Crizotinib | 10uM | 6h | GPATCH2L | 0.00 | 4.50 |
| HT29_13_GR_ChDB_0120 | Crizotinib | 10uM | 6h | IER3 | 0.00 | 2.62 |
| HT29_13_GR_ChDB_0120 | Crizotinib | 10uM | 6h | SPSB3 | 0.00 | 3.87 |
| HT29_13_GR_ChDB_0120 | Crizotinib | 10uM | 6h | PNRC1 | 0.00 | 19.78 |
| HT29_13_GR_ChDB_0120 | Crizotinib | 10uM | 6h | NR4A2 | 0.00 | 8.48 |
| HT29_13_GR_ChDB_0120 | Crizotinib | 10uM | 6h | KIAA0040 | 0.00 | 2.81 |
| HT29_13_GR_ChDB_0120 | Crizotinib | 10uM | 6h | SERTAD3 | 0.00 | 3.89 |
| HT29_13_GR_ChDB_0120 | Crizotinib | 10uM | 6h | HNRNPD | 0.00 | 4.30 |
| HT29_13_GR_ChDB_0120 | Crizotinib | 10uM | 6h | SMEK1 | 0.00 | 2.04 |
| HT29_13_GR_ChDB_0120 | Crizotinib | 10uM | 6h | PYROXD1 | 0.00 | 0.34 |
| HT29_13_GR_ChDB_0120 | Crizotinib | 10uM | 6h | CBX5 | 0.00 | 0.40 |
| HT29_13_GR_ChDB_0120 | Crizotinib | 10uM | 6h | MPHOSPH9 | 0.00 | 0.49 |
| HT29_13_GR_ChDB_0120 | Crizotinib | 10uM | 6h | MCM10 | 0.00 | 0.41 |
| HT29_13_GR_ChDB_0120 | Crizotinib | 10uM | 6h | SAMD4A | 0.00 | 0.33 |
| HT29_13_GR_ChDB_0120 | Crizotinib | 10uM | 6h | AGK | 0.00 | 0.25 |
| HT29_13_GR_ChDB_0120 | Crizotinib | 10uM | 6h | SLC30A5 | 0.00 | 0.41 |
| HT29_13_GR_ChDB_0120 | Crizotinib | 10uM | 6h | LARP1 | 0.00 | 0.50 |
| HT29_13_GR_ChDB_0120 | Crizotinib | 10uM | 6h | ASPM | 0.00 | 0.44 |
| HT29_13_GR_ChDB_0120 | Crizotinib | 10uM | 6h | OGT | 0.00 | 0.38 |
| HT29_13_GR_ChDB_0120 | Crizotinib | 10uM | 6h | WNK1 | 0.00 | 0.28 |
| HT29_13_GR_ChDB_0120 | Crizotinib | 10uM | 6h | CDC16 | 0.00 | 0.45 |
| HT29_13_GR_ChDB_0120 | Crizotinib | 10uM | 6h | CASP8 | 0.00 | 0.32 |
| HT29_13_GR_ChDB_0120 | Crizotinib | 10uM | 6h | MAN1A2 | 0.00 | 0.31 |
| HT29_13_GR_ChDB_0120 | Crizotinib | 10uM | 6h | NAPG | 0.00 | 2.03 |
| HT29_13_GR_ChDB_0120 | Crizotinib | 10uM | 6h | GFER | 0.00 | 2.19 |
| HT29_13_GR_ChDB_0120 | Crizotinib | 10uM | 6h | ZBTB1 | 0.00 | 4.62 |
| HT29_13_GR_ChDB_0120 | Crizotinib | 10uM | 6h | PUM1 | 0.00 | 2.34 |
| HT29_13_GR_ChDB_0120 | Crizotinib | 10uM | 6h | SERBP1 | 0.00 | 2.13 |
| HT29_13_GR_ChDB_0120 | Crizotinib | 10uM | 6h | JUND | 0.00 | 3.08 |
| HT29_13_GR_ChDB_0120 | Crizotinib | 10uM | 6h | DDX58 | 0.00 | 2.23 |
| HT29_13_GR_ChDB_0120 | Crizotinib | 10uM | 6h | GNAI3 | 0.00 | 2.10 |
| HT29_13_GR_ChDB_0120 | Crizotinib | 10uM | 6h | CHIC2 | 0.00 | 4.97 |
| HT29_13_GR_ChDB_0120 | Crizotinib | 10uM | 6h | KDM4A | 0.00 | 2.19 |
| HT29_13_GR_ChDB_0120 | Crizotinib | 10uM | 6h | ENOX2 | 0.00 | 0.46 |
| HT29_13_GR_ChDB_0120 | Crizotinib | 10uM | 6h | PUS1 | 0.00 | 0.31 |
| HT29_13_GR_ChDB_0120 | Crizotinib | 10uM | 6h | CLN5 | 0.00 | 0.36 |
| HT29_13_GR_ChDB_0120 | Crizotinib | 10uM | 6h | PSMD11 | 0.00 | 0.22 |
| HT29_13_GR_ChDB_0120 | Crizotinib | 10uM | 6h | SIGMAR1 | 0.00 | 0.46 |
| HT29_13_GR_ChDB_0120 | Crizotinib | 10uM | 6h | TMEM194A | 0.00 | 0.34 |
| HT29_13_GR_ChDB_0120 | Crizotinib | 10uM | 6h | ALG13 | 0.00 | 0.45 |
| HT29_13_GR_ChDB_0120 | Crizotinib | 10uM | 6h | TIMM44 | 0.00 | 0.46 |
| HT29_13_GR_ChDB_0120 | Crizotinib | 10uM | 6h | DPH5 | 0.00 | 0.48 |
| HT29_13_GR_ChDB_0120 | Crizotinib | 10uM | 6h | NAA40 | 0.00 | 0.35 |
| HT29_13_GR_ChDB_0120 | Crizotinib | 10uM | 6h | NARG2 | 0.00 | 0.43 |
| HT29_13_GR_ChDB_0120 | Crizotinib | 10uM | 6h | DIMT1 | 0.00 | 0.46 |
| HT29_13_GR_ChDB_0120 | Crizotinib | 10uM | 6h | HSPA13 | 0.00 | 2.19 |
| HT29_13_GR_ChDB_0120 | Crizotinib | 10uM | 6h | GAN | 0.00 | 3.59 |
| HT29_13_GR_ChDB_0120 | Crizotinib | 10uM | 6h | AMBRA1 | 0.00 | 2.14 |
| HT29_13_GR_ChDB_0120 | Crizotinib | 10uM | 6h | DCLRE1C | 0.00 | 2.30 |
| HT29_13_GR_ChDB_0120 | Crizotinib | 10uM | 6h | KLHDC10 | 0.00 | 4.08 |
| HT29_13_GR_ChDB_0120 | Crizotinib | 10uM | 6h | TBC1D8 | 0.00 | 2.08 |
| HT29_13_GR_ChDB_0120 | Crizotinib | 10uM | 6h | AKAP17A | 0.00 | 2.36 |
| HT29_13_GR_ChDB_0120 | Crizotinib | 10uM | 6h | TJP1 | 0.00 | 2.54 |
| HT29_13_GR_ChDB_0120 | Crizotinib | 10uM | 6h | IRX5 | 0.00 | 3.27 |
| HT29_13_GR_ChDB_0120 | Crizotinib | 10uM | 6h | RAB5A | 0.00 | 2.34 |
| HT29_13_GR_ChDB_0120 | Crizotinib | 10uM | 6h | DNMBP | 0.00 | 5.48 |
| HT29_13_GR_ChDB_0120 | Crizotinib | 10uM | 6h | UCHL5 | 0.00 | 0.50 |
| HT29_13_GR_ChDB_0120 | Crizotinib | 10uM | 6h | CIAO1 | 0.00 | 0.31 |
| HT29_13_GR_ChDB_0120 | Crizotinib | 10uM | 6h | PREPL | 0.00 | 0.36 |
| HT29_13_GR_ChDB_0120 | Crizotinib | 10uM | 6h | TBC1D4 | 0.00 | 0.34 |
| HT29_13_GR_ChDB_0120 | Crizotinib | 10uM | 6h | MRPL46 | 0.00 | 0.46 |
| HT29_13_GR_ChDB_0120 | Crizotinib | 10uM | 6h | GTF2H1 | 0.00 | 0.43 |
| HT29_13_GR_ChDB_0120 | Crizotinib | 10uM | 6h | FUBP1 | 0.00 | 0.38 |
| HT29_13_GR_ChDB_0120 | Crizotinib | 10uM | 6h | ENOSF1 | 0.00 | 0.47 |
| HT29_13_GR_ChDB_0120 | Crizotinib | 10uM | 6h | MAP4 | 0.00 | 0.44 |
| HT29_13_GR_ChDB_0120 | Crizotinib | 10uM | 6h | TRIM33 | 0.00 | 0.32 |
| HT29_13_GR_ChDB_0120 | Crizotinib | 10uM | 6h | TMF1 | 0.00 | 0.37 |
| HT29_13_GR_ChDB_0120 | Crizotinib | 10uM | 6h | RFX5 | 0.00 | 0.30 |
| HT29_13_GR_ChDB_0120 | Crizotinib | 10uM | 6h | LIMCH1 | 0.00 | 0.41 |
| HT29_13_GR_ChDB_0120 | Crizotinib | 10uM | 6h | TOR3A | 0.00 | 0.47 |
| HT29_13_GR_ChDB_0120 | Crizotinib | 10uM | 6h | LPCAT4 | 0.00 | 0.37 |
| HT29_13_GR_ChDB_0120 | Crizotinib | 10uM | 6h | RAP2B | 0.00 | 2.15 |
| HT29_13_GR_ChDB_0120 | Crizotinib | 10uM | 6h | LAMB3 | 0.00 | 4.38 |
| HT29_13_GR_ChDB_0120 | Crizotinib | 10uM | 6h | ABTB2 | 0.00 | 2.78 |
| HT29_13_GR_ChDB_0120 | Crizotinib | 10uM | 6h | CHMP2B | 0.00 | 2.16 |
| HT29_13_GR_ChDB_0120 | Crizotinib | 10uM | 6h | TRIM31 | 0.00 | 3.04 |
| HT29_13_GR_ChDB_0120 | Crizotinib | 10uM | 6h | CNOT1 | 0.00 | 5.50 |
| HT29_13_GR_ChDB_0120 | Crizotinib | 10uM | 6h | MARCH7 | 0.00 | 2.21 |
| HT29_13_GR_ChDB_0120 | Crizotinib | 10uM | 6h | CDYL | 0.00 | 2.06 |
| HT29_13_GR_ChDB_0120 | Crizotinib | 10uM | 6h | TRAF6 | 0.00 | 3.48 |
| HT29_13_GR_ChDB_0120 | Crizotinib | 10uM | 6h | TMBIM6 | 0.00 | 2.02 |
| HT29_13_GR_ChDB_0120 | Crizotinib | 10uM | 6h | RSF1 | 0.00 | 2.53 |
| HT29_13_GR_ChDB_0120 | Crizotinib | 10uM | 6h | FAF2 | 0.00 | 2.05 |
| HT29_13_GR_ChDB_0120 | Crizotinib | 10uM | 6h | NRAS | 0.00 | 3.22 |
| HT29_13_GR_ChDB_0120 | Crizotinib | 10uM | 6h | NCAPD3 | 0.00 | 0.48 |
| HT29_13_GR_ChDB_0120 | Crizotinib | 10uM | 6h | SLC5A3 | 0.00 | 0.41 |
| HT29_13_GR_ChDB_0120 | Crizotinib | 10uM | 6h | AP5M1 | 0.00 | 0.40 |
| HT29_13_GR_ChDB_0120 | Crizotinib | 10uM | 6h | ABHD10 | 0.00 | 0.32 |
| HT29_13_GR_ChDB_0120 | Crizotinib | 10uM | 6h | SRSF5 | 0.00 | 0.38 |
| HT29_13_GR_ChDB_0120 | Crizotinib | 10uM | 6h | POLRMT | 0.00 | 0.47 |
| HT29_13_GR_ChDB_0120 | Crizotinib | 10uM | 6h | CD3EAP | 0.00 | 0.40 |
| HT29_13_GR_ChDB_0120 | Crizotinib | 10uM | 6h | KNOP1 | 0.00 | 0.44 |
| HT29_13_GR_ChDB_0120 | Crizotinib | 10uM | 6h | APTX | 0.00 | 0.46 |
| HT29_13_GR_ChDB_0120 | Crizotinib | 10uM | 6h | FAM111A | 0.00 | 0.31 |
| HT29_13_GR_ChDB_0120 | Crizotinib | 10uM | 6h | CDK13 | 0.00 | 0.41 |
| HT29_13_GR_ChDB_0120 | Crizotinib | 10uM | 6h | PDSS1 | 0.00 | 0.47 |
| HT29_13_GR_ChDB_0120 | Crizotinib | 10uM | 6h | KAT8 | 0.00 | 0.28 |
| HT29_13_GR_ChDB_0120 | Crizotinib | 10uM | 6h | YIPF6 | 0.00 | 0.48 |
| HT29_13_GR_ChDB_0120 | Crizotinib | 10uM | 6h | DSCC1 | 0.00 | 0.48 |
| HT29_13_GR_ChDB_0120 | Crizotinib | 10uM | 6h | C1orf109 | 0.00 | 0.49 |
| HT29_13_GR_ChDB_0120 | Crizotinib | 10uM | 6h | AGPAT5 | 0.00 | 0.46 |
| HT29_13_GR_ChDB_0120 | Crizotinib | 10uM | 6h | MFN1 | 0.00 | 0.49 |
| HT29_13_GR_ChDB_0120 | Crizotinib | 10uM | 6h | SRSF6 | 0.00 | 0.42 |
| HT29_13_GR_ChDB_0120 | Crizotinib | 10uM | 6h | SFSWAP | 0.00 | 0.39 |
| HT29_13_GR_ChDB_0120 | Crizotinib | 10uM | 6h | PAK1IP1 | 0.00 | 0.49 |
| HT29_13_GR_ChDB_0120 | Crizotinib | 10uM | 6h | TGFBRAP1 | 0.00 | 0.48 |
| HT29_13_GR_ChDB_0120 | Crizotinib | 10uM | 6h | LARP4B | 0.00 | 0.45 |
| HT29_13_GR_ChDB_0120 | Crizotinib | 10uM | 6h | ACSL3 | 0.00 | 0.36 |
| HT29_13_GR_ChDB_0120 | Crizotinib | 10uM | 6h | PUS7 | 0.00 | 0.28 |
| HT29_13_GR_ChDB_0120 | Crizotinib | 10uM | 6h | RHOT1 | 0.00 | 0.50 |
| HT29_13_GR_ChDB_0120 | Crizotinib | 10uM | 6h | ACYP1 | 0.00 | 0.22 |
| HT29_13_GR_ChDB_0120 | Crizotinib | 10uM | 6h | MRS2 | 0.00 | 0.35 |
| HT29_13_GR_ChDB_0120 | Crizotinib | 10uM | 6h | CDC40 | 0.00 | 2.46 |
| HT29_13_GR_ChDB_0120 | Crizotinib | 10uM | 6h | NR2C2 | 0.00 | 2.19 |
| HT29_13_GR_ChDB_0120 | Crizotinib | 10uM | 6h | DUSP5 | 0.00 | 6.92 |
| HT29_13_GR_ChDB_0120 | Crizotinib | 10uM | 6h | TFIP11 | 0.00 | 2.30 |
| HT29_13_GR_ChDB_0120 | Crizotinib | 10uM | 6h | CDC5L | 0.00 | 2.02 |
| HT29_13_GR_ChDB_0120 | Crizotinib | 10uM | 6h | RAB9A | 0.00 | 2.30 |
| HT29_13_GR_ChDB_0120 | Crizotinib | 10uM | 6h | KIAA0232 | 0.00 | 2.62 |
| HT29_13_GR_ChDB_0120 | Crizotinib | 10uM | 6h | HMGCR | 0.00 | 2.31 |
| HT29_13_GR_ChDB_0120 | Crizotinib | 10uM | 6h | CDK17 | 0.00 | 3.58 |
| HT29_13_GR_ChDB_0120 | Crizotinib | 10uM | 6h | BTN2A1 | 0.00 | 2.73 |
| HT29_13_GR_ChDB_0120 | Crizotinib | 10uM | 6h | CD164 | 0.00 | 2.93 |
| HT29_13_GR_ChDB_0120 | Crizotinib | 10uM | 6h | RNF114 | 0.00 | 2.02 |
| HT29_13_GR_ChDB_0120 | Crizotinib | 10uM | 6h | BTBD7 | 0.00 | 2.09 |
| HT29_13_GR_ChDB_0120 | Crizotinib | 10uM | 6h | SEC14L1 | 0.00 | 2.53 |
| HT29_13_GR_ChDB_0120 | Crizotinib | 10uM | 6h | CLDN1 | 0.00 | 2.66 |
| HT29_13_GR_ChDB_0120 | Crizotinib | 10uM | 6h | LMO7 | 0.00 | 3.20 |
| HT29_13_GR_ChDB_0120 | Crizotinib | 10uM | 6h | TMEM57 | 0.00 | 2.42 |
| HT29_13_GR_ChDB_0120 | Crizotinib | 10uM | 6h | HMGXB4 | 0.00 | 2.29 |
| HT29_13_GR_ChDB_0120 | Crizotinib | 10uM | 6h | FAM120A | 0.00 | 3.31 |
| HT29_13_GR_ChDB_0120 | Crizotinib | 10uM | 6h | RNF167 | 0.00 | 2.18 |
| HT29_13_GR_ChDB_0120 | Crizotinib | 10uM | 6h | WEE1 | 0.00 | 3.07 |
| HT29_13_GR_ChDB_0120 | Crizotinib | 10uM | 6h | FBXW2 | 0.00 | 0.17 |
| HT29_13_GR_ChDB_0120 | Crizotinib | 10uM | 6h | TDP1 | 0.00 | 0.30 |
| HT29_13_GR_ChDB_0120 | Crizotinib | 10uM | 6h | OSBPL10 | 0.00 | 0.48 |
| HT29_13_GR_ChDB_0120 | Crizotinib | 10uM | 6h | NOL6 | 0.00 | 0.45 |
| HT29_13_GR_ChDB_0120 | Crizotinib | 10uM | 6h | MDM1 | 0.00 | 0.49 |
| HT29_13_GR_ChDB_0120 | Crizotinib | 10uM | 6h | TRAPPC12 | 0.00 | 0.36 |
| HT29_13_GR_ChDB_0120 | Crizotinib | 10uM | 6h | TIMM8A | 0.00 | 0.33 |
| HT29_13_GR_ChDB_0120 | Crizotinib | 10uM | 6h | WDR3 | 0.00 | 0.25 |
| HT29_13_GR_ChDB_0120 | Crizotinib | 10uM | 6h | CAAP1 | 0.00 | 0.33 |
| HT29_13_GR_ChDB_0120 | Crizotinib | 10uM | 6h | HEATR3 | 0.00 | 0.27 |
| HT29_13_GR_ChDB_0120 | Crizotinib | 10uM | 6h | TCTN3 | 0.00 | 0.41 |
| HT29_13_GR_ChDB_0120 | Crizotinib | 10uM | 6h | GEMIN6 | 0.00 | 0.42 |
| HT29_13_GR_ChDB_0120 | Crizotinib | 10uM | 6h | CKAP4 | 0.00 | 0.46 |
| HT29_13_GR_ChDB_0120 | Crizotinib | 10uM | 6h | ATP5G3 | 0.00 | 0.37 |
| HT29_13_GR_ChDB_0120 | Crizotinib | 10uM | 6h | SLC35A5 | 0.00 | 0.37 |
| HT29_13_GR_ChDB_0120 | Crizotinib | 10uM | 6h | PEX5 | 0.00 | 0.42 |
| HT29_13_GR_ChDB_0120 | Crizotinib | 10uM | 6h | TNPO1 | 0.00 | 0.39 |
| HT29_13_GR_ChDB_0120 | Crizotinib | 10uM | 6h | HNRNPU | 0.00 | 0.47 |
| HT29_13_GR_ChDB_0120 | Crizotinib | 10uM | 6h | RALGAPA1 | 0.00 | 0.43 |
| HT29_13_GR_ChDB_0120 | Crizotinib | 10uM | 6h | FAM64A | 0.00 | 0.45 |
| HT29_13_GR_ChDB_0120 | Crizotinib | 10uM | 6h | CPSF6 | 0.00 | 0.35 |
| HT29_13_GR_ChDB_0120 | Crizotinib | 10uM | 6h | KLC1 | 0.00 | 0.42 |
| HT29_13_GR_ChDB_0120 | Crizotinib | 10uM | 6h | MINA | 0.00 | 0.46 |
| HT29_13_GR_ChDB_0120 | Crizotinib | 10uM | 6h | UBR5 | 0.00 | 0.36 |
| HT29_13_GR_ChDB_0120 | Crizotinib | 10uM | 6h | GAS2L1 | 0.00 | 0.38 |
| HT29_13_GR_ChDB_0120 | Crizotinib | 10uM | 6h | NEIL3 | 0.00 | 0.31 |
| HT29_13_GR_ChDB_0120 | Crizotinib | 10uM | 6h | NCOR1 | 0.00 | 0.43 |
| HT29_13_GR_ChDB_0120 | Crizotinib | 10uM | 6h | MARCH6 | 0.00 | 0.20 |
| HT29_13_GR_ChDB_0120 | Crizotinib | 10uM | 6h | RANBP2 | 0.00 | 0.36 |
| HT29_13_GR_ChDB_0120 | Crizotinib | 10uM | 6h | REXO4 | 0.00 | 0.45 |
| HT29_13_GR_ChDB_0120 | Crizotinib | 10uM | 6h | YTHDC2 | 0.00 | 0.37 |
| HT29_13_GR_ChDB_0120 | Crizotinib | 10uM | 6h | C12orf4 | 0.00 | 0.26 |
| HT29_13_GR_ChDB_0120 | Crizotinib | 10uM | 6h | TIPIN | 0.00 | 0.44 |
| HT29_13_GR_ChDB_0120 | Crizotinib | 10uM | 6h | TRMT61B | 0.00 | 0.19 |
| HT29_13_GR_ChDB_0120 | Crizotinib | 10uM | 6h | RFC4 | 0.00 | 0.24 |
| HT29_13_GR_ChDB_0120 | Crizotinib | 10uM | 6h | EVI5 | 0.00 | 0.28 |
| HT29_13_GR_ChDB_0120 | Crizotinib | 10uM | 6h | SMCHD1 | 0.00 | 0.30 |
| HT29_13_GR_ChDB_0120 | Crizotinib | 10uM | 6h | MRP63 | 0.00 | 0.40 |
| HT29_13_GR_ChDB_0120 | Crizotinib | 10uM | 6h | LDLRAP1 | 0.00 | 0.34 |
| HT29_13_GR_ChDB_0120 | Crizotinib | 10uM | 6h | TAF11 | 0.00 | 0.26 |
| HT29_13_GR_ChDB_0120 | Crizotinib | 10uM | 6h | KAZN | 0.00 | 0.43 |
| HT29_13_GR_ChDB_0120 | Crizotinib | 10uM | 6h | FASTKD1 | 0.00 | 0.13 |
| HT29_13_GR_ChDB_0120 | Crizotinib | 10uM | 6h | XPOT | 0.00 | 0.48 |
| HT29_13_GR_ChDB_0120 | Crizotinib | 10uM | 6h | COX11 | 0.00 | 0.49 |
| HT29_13_GR_ChDB_0120 | Crizotinib | 10uM | 6h | MTMR4 | 0.00 | 0.43 |
| HT29_13_GR_ChDB_0120 | Crizotinib | 10uM | 6h | MIOS | 0.00 | 0.47 |
| HT29_13_GR_ChDB_0120 | Crizotinib | 10uM | 6h | MEX3C | 0.00 | 2.30 |
| HT29_13_GR_ChDB_0120 | Crizotinib | 10uM | 6h | TUFT1 | 0.00 | 5.32 |
| HT29_13_GR_ChDB_0120 | Crizotinib | 10uM | 6h | RPS21 | 0.00 | 2.07 |
| HT29_13_GR_ChDB_0120 | Crizotinib | 10uM | 6h | GSE1 | 0.00 | 2.67 |
| HT29_13_GR_ChDB_0120 | Crizotinib | 10uM | 6h | ZNF507 | 0.00 | 2.17 |
| HT29_13_GR_ChDB_0120 | Crizotinib | 10uM | 6h | APOBEC3B | 0.00 | 3.29 |
| HT29_13_GR_ChDB_0120 | Crizotinib | 10uM | 6h | PRKAB1 | 0.00 | 2.86 |
| HT29_13_GR_ChDB_0120 | Crizotinib | 10uM | 6h | HMGCS1 | 0.00 | 4.68 |
| HT29_13_GR_ChDB_0120 | Crizotinib | 10uM | 6h | NEDD4L | 0.00 | 3.39 |
| HT29_13_GR_ChDB_0120 | Crizotinib | 10uM | 6h | SETD4 | 0.00 | 2.39 |
| HT29_13_GR_ChDB_0120 | Crizotinib | 10uM | 6h | GDF15 | 0.00 | 19.87 |
| HT29_13_GR_ChDB_0120 | Crizotinib | 10uM | 6h | SERTAD2 | 0.00 | 2.17 |
| HT29_13_GR_ChDB_0120 | Crizotinib | 10uM | 6h | CAD | 0.00 | 0.31 |
| HT29_13_GR_ChDB_0120 | Crizotinib | 10uM | 6h | CD47 | 0.00 | 0.38 |
| HT29_13_GR_ChDB_0120 | Crizotinib | 10uM | 6h | USP33 | 0.00 | 0.46 |
| HT29_13_GR_ChDB_0120 | Crizotinib | 10uM | 6h | MLF1IP | 0.00 | 0.49 |
| HT29_13_GR_ChDB_0120 | Crizotinib | 10uM | 6h | RWDD3 | 0.00 | 0.37 |
| HT29_13_GR_ChDB_0120 | Crizotinib | 10uM | 6h | ABHD3 | 0.00 | 0.36 |
| HT29_13_GR_ChDB_0120 | Crizotinib | 10uM | 6h | INPP4A | 0.00 | 0.40 |
| HT29_13_GR_ChDB_0120 | Crizotinib | 10uM | 6h | COX15 | 0.00 | 0.45 |
| HT29_13_GR_ChDB_0120 | Crizotinib | 10uM | 6h | SLC33A1 | 0.00 | 0.40 |
| HT29_13_GR_ChDB_0120 | Crizotinib | 10uM | 6h | INTS8 | 0.00 | 0.39 |
| HT29_13_GR_ChDB_0120 | Crizotinib | 10uM | 6h | ABI2 | 0.00 | 0.38 |
| HT29_13_GR_ChDB_0120 | Crizotinib | 10uM | 6h | KRR1 | 0.00 | 0.42 |
| HT29_13_GR_ChDB_0120 | Crizotinib | 10uM | 6h | FLAD1 | 0.00 | 0.44 |
| HT29_13_GR_ChDB_0120 | Crizotinib | 10uM | 6h | DUSP4 | 0.00 | 0.43 |
| HT29_13_GR_ChDB_0120 | Crizotinib | 10uM | 6h | PDCD11 | 0.00 | 0.39 |
| HT29_13_GR_ChDB_0120 | Crizotinib | 10uM | 6h | NETO2 | 0.00 | 0.42 |
| HT29_13_GR_ChDB_0120 | Crizotinib | 10uM | 6h | HK2 | 0.00 | 0.36 |
| HT29_13_GR_ChDB_0120 | Crizotinib | 10uM | 6h | EXPH5 | 0.00 | 0.37 |
| HT29_13_GR_ChDB_0120 | Crizotinib | 10uM | 6h | USP3 | 0.00 | 0.35 |
| HT29_13_GR_ChDB_0120 | Crizotinib | 10uM | 6h | C5orf28 | 0.00 | 0.36 |
| HT29_13_GR_ChDB_0120 | Crizotinib | 10uM | 6h | ASB7 | 0.00 | 3.79 |
| HT29_13_GR_ChDB_0120 | Crizotinib | 10uM | 6h | ODF2 | 0.00 | 2.05 |
| HT29_13_GR_ChDB_0120 | Crizotinib | 10uM | 6h | RND1 | 0.00 | 2.32 |
| HT29_13_GR_ChDB_0120 | Crizotinib | 10uM | 6h | UBE2W | 0.00 | 2.81 |
| HT29_13_GR_ChDB_0120 | Crizotinib | 10uM | 6h | AMOTL2 | 0.00 | 3.61 |
| HT29_13_GR_ChDB_0120 | Crizotinib | 10uM | 6h | RBM39 | 0.00 | 2.60 |
| HT29_13_GR_ChDB_0120 | Crizotinib | 10uM | 6h | BIRC3 | 0.00 | 4.30 |
| HT29_13_GR_ChDB_0120 | Crizotinib | 10uM | 6h | OCLN | 0.00 | 3.24 |
| HT29_13_GR_ChDB_0120 | Crizotinib | 10uM | 6h | C2orf49 | 0.00 | 2.05 |
| HT29_13_GR_ChDB_0120 | Crizotinib | 10uM | 6h | KLHL28 | 0.00 | 3.62 |
| HT29_13_GR_ChDB_0120 | Crizotinib | 10uM | 6h | RTF1 | 0.00 | 2.78 |
| HT29_13_GR_ChDB_0120 | Crizotinib | 10uM | 6h | UBE2K | 0.00 | 0.45 |
| HT29_13_GR_ChDB_0120 | Crizotinib | 10uM | 6h | VPS13A | 0.00 | 0.47 |
| HT29_13_GR_ChDB_0120 | Crizotinib | 10uM | 6h | PRKD3 | 0.00 | 0.46 |
| HT29_13_GR_ChDB_0120 | Crizotinib | 10uM | 6h | PSME4 | 0.00 | 0.26 |
| HT29_13_GR_ChDB_0120 | Crizotinib | 10uM | 6h | C12orf29 | 0.00 | 0.35 |
| HT29_13_GR_ChDB_0120 | Crizotinib | 10uM | 6h | OSBPL3 | 0.00 | 0.46 |
| HT29_13_GR_ChDB_0120 | Crizotinib | 10uM | 6h | POLR3G | 0.00 | 0.20 |
| HT29_13_GR_ChDB_0120 | Crizotinib | 10uM | 6h | TTI1 | 0.00 | 0.29 |
| HT29_13_GR_ChDB_0120 | Crizotinib | 10uM | 6h | SIKE1 | 0.00 | 0.35 |
| HT29_13_GR_ChDB_0120 | Crizotinib | 10uM | 6h | GLMN | 0.00 | 0.16 |
| HT29_13_GR_ChDB_0120 | Crizotinib | 10uM | 6h | GATC | 0.00 | 0.45 |
| HT29_13_GR_ChDB_0120 | Crizotinib | 10uM | 6h | MTMR1 | 0.00 | 0.48 |
| HT29_13_GR_ChDB_0120 | Crizotinib | 10uM | 6h | SLC25A11 | 0.00 | 0.47 |
| HT29_13_GR_ChDB_0120 | Crizotinib | 10uM | 6h | MKNK1 | 0.00 | 0.46 |
| HT29_13_GR_ChDB_0120 | Crizotinib | 10uM | 6h | KRIT1 | 0.00 | 0.29 |
| HT29_13_GR_ChDB_0120 | Crizotinib | 10uM | 6h | DPY19L1 | 0.00 | 0.36 |
| HT29_13_GR_ChDB_0120 | Crizotinib | 10uM | 6h | EIF2AK3 | 0.00 | 2.43 |
| HT29_13_GR_ChDB_0120 | Crizotinib | 10uM | 6h | WASF2 | 0.00 | 2.61 |
| HT29_13_GR_ChDB_0120 | Crizotinib | 10uM | 6h | GCNT3 | 0.00 | 3.06 |
| HT29_13_GR_ChDB_0120 | Crizotinib | 10uM | 6h | GMFB | 0.00 | 2.47 |
| HT29_13_GR_ChDB_0120 | Crizotinib | 10uM | 6h | MAP3K3 | 0.00 | 2.01 |
| HT29_13_GR_ChDB_0120 | Crizotinib | 10uM | 6h | FEM1C | 0.00 | 3.17 |
| HT29_13_GR_ChDB_0120 | Crizotinib | 10uM | 6h | PI3 | 0.00 | 2.37 |
| HT29_13_GR_ChDB_0120 | Crizotinib | 10uM | 6h | HIVEP1 | 0.00 | 2.43 |
| HT29_13_GR_ChDB_0120 | Crizotinib | 10uM | 6h | ZNF574 | 0.00 | 2.07 |
| HT29_13_GR_ChDB_0120 | Crizotinib | 10uM | 6h | PPP4R2 | 0.00 | 2.13 |
| HT29_13_GR_ChDB_0120 | Crizotinib | 10uM | 6h | TMEM39A | 0.00 | 2.07 |
| HT29_13_GR_ChDB_0120 | Crizotinib | 10uM | 6h | KDM3A | 0.00 | 4.31 |
| HT29_13_GR_ChDB_0120 | Crizotinib | 10uM | 6h | TSPAN5 | 0.00 | 2.36 |
| HT29_13_GR_ChDB_0120 | Crizotinib | 10uM | 6h | PRRC1 | 0.00 | 2.63 |
| HT29_13_GR_ChDB_0120 | Crizotinib | 10uM | 6h | COQ10B | 0.00 | 3.08 |
| HT29_13_GR_ChDB_0120 | Crizotinib | 10uM | 6h | GAREM | 0.00 | 4.79 |
| HT29_13_GR_ChDB_0120 | Crizotinib | 10uM | 6h | CLK4 | 0.00 | 2.43 |
| HT29_13_GR_ChDB_0120 | Crizotinib | 10uM | 6h | STYK1 | 0.00 | 2.74 |
| HT29_13_GR_ChDB_0120 | Crizotinib | 10uM | 6h | ATF3 | 0.00 | 4.22 |
| HT29_13_GR_ChDB_0120 | Crizotinib | 10uM | 6h | RLF | 0.00 | 2.31 |
| HT29_13_GR_ChDB_0120 | Crizotinib | 10uM | 6h | LIN7C | 0.00 | 2.59 |
| HT29_13_GR_ChDB_0120 | Crizotinib | 10uM | 6h | RBM7 | 0.00 | 2.96 |
| HT29_13_GR_ChDB_0120 | Crizotinib | 10uM | 6h | ZNF408 | 0.00 | 2.86 |
| HT29_13_GR_ChDB_0120 | Crizotinib | 10uM | 6h | POP1 | 0.00 | 0.22 |
| HT29_13_GR_ChDB_0120 | Crizotinib | 10uM | 6h | ELK4 | 0.00 | 0.37 |
| HT29_13_GR_ChDB_0120 | Crizotinib | 10uM | 6h | COL4A2 | 0.00 | 0.44 |
| HT29_13_GR_ChDB_0120 | Crizotinib | 10uM | 6h | PPWD1 | 0.00 | 0.30 |
| HT29_13_GR_ChDB_0120 | Crizotinib | 10uM | 6h | POGLUT1 | 0.00 | 0.33 |
| HT29_13_GR_ChDB_0120 | Crizotinib | 10uM | 6h | MED27 | 0.00 | 0.45 |
| HT29_13_GR_ChDB_0120 | Crizotinib | 10uM | 6h | AIM1 | 0.00 | 0.24 |
| HT29_13_GR_ChDB_0120 | Crizotinib | 10uM | 6h | SNAPC3 | 0.00 | 0.40 |
| HT29_13_GR_ChDB_0120 | Crizotinib | 10uM | 6h | TEX2 | 0.00 | 0.38 |
| HT29_13_GR_ChDB_0120 | Crizotinib | 10uM | 6h | SRPK1 | 0.00 | 0.44 |
| HT29_13_GR_ChDB_0120 | Crizotinib | 10uM | 6h | SEC23IP | 0.00 | 0.38 |
| HT29_13_GR_ChDB_0120 | Crizotinib | 10uM | 6h | C21orf33 | 0.00 | 0.36 |
| HT29_13_GR_ChDB_0120 | Crizotinib | 10uM | 6h | DYNC2LI1 | 0.00 | 0.46 |
| HT29_13_GR_ChDB_0120 | Crizotinib | 10uM | 6h | LRP8 | 0.00 | 0.37 |
| HT29_13_GR_ChDB_0120 | Crizotinib | 10uM | 6h | ERI2 | 0.00 | 0.40 |
| HT29_13_GR_ChDB_0120 | Crizotinib | 10uM | 6h | ATP6V1C1 | 0.00 | 0.44 |
| HT29_13_GR_ChDB_0120 | Crizotinib | 10uM | 6h | STAG1 | 0.00 | 0.36 |
| HT29_13_GR_ChDB_0120 | Crizotinib | 10uM | 6h | SCAF11 | 0.00 | 0.24 |
| HT29_13_GR_ChDB_0120 | Crizotinib | 10uM | 6h | BPTF | 0.00 | 0.37 |
| HT29_13_GR_ChDB_0120 | Crizotinib | 10uM | 6h | TMEM177 | 0.00 | 0.26 |
| HT29_13_GR_ChDB_0120 | Crizotinib | 10uM | 6h | ZDHHC3 | 0.00 | 2.22 |
| HT29_13_GR_ChDB_0120 | Crizotinib | 10uM | 6h | SH3GLB1 | 0.00 | 0.41 |
| HT29_13_GR_ChDB_0120 | Crizotinib | 10uM | 6h | IPO5 | 0.00 | 3.00 |
| HT29_13_GR_ChDB_0120 | Crizotinib | 10uM | 6h | OSER1 | 0.00 | 2.99 |
| HT29_13_GR_ChDB_0120 | Crizotinib | 10uM | 6h | GAB2 | 0.00 | 2.35 |
| HT29_13_GR_ChDB_0120 | Crizotinib | 10uM | 6h | C11orf30 | 0.00 | 6.25 |
| HT29_13_GR_ChDB_0120 | Crizotinib | 10uM | 6h | CDS2 | 0.00 | 2.10 |
| HT29_13_GR_ChDB_0120 | Crizotinib | 10uM | 6h | RBMS1 | 0.00 | 2.12 |
| HT29_13_GR_ChDB_0120 | Crizotinib | 10uM | 6h | GTF2IRD1 | 0.00 | 2.10 |
| HT29_13_GR_ChDB_0120 | Crizotinib | 10uM | 6h | SLC12A7 | 0.00 | 2.89 |
| HT29_13_GR_ChDB_0120 | Crizotinib | 10uM | 6h | ARL14 | 0.00 | 2.87 |
| HT29_13_GR_ChDB_0120 | Crizotinib | 10uM | 6h | ZNF430 | 0.00 | 2.64 |
| HT29_13_GR_ChDB_0120 | Crizotinib | 10uM | 6h | B3GNT2 | 0.00 | 2.25 |
| HT29_13_GR_ChDB_0120 | Crizotinib | 10uM | 6h | PRPF40A | 0.00 | 2.17 |
| HT29_13_GR_ChDB_0120 | Crizotinib | 10uM | 6h | ATP6V0A2 | 0.00 | 0.39 |
| HT29_13_GR_ChDB_0120 | Crizotinib | 10uM | 6h | STIP1 | 0.00 | 0.46 |
| HT29_13_GR_ChDB_0120 | Crizotinib | 10uM | 6h | PDE3B | 0.00 | 0.32 |
| HT29_13_GR_ChDB_0120 | Crizotinib | 10uM | 6h | PAXBP1 | 0.00 | 0.39 |
| HT29_13_GR_ChDB_0120 | Crizotinib | 10uM | 6h | SNTB2 | 0.00 | 0.41 |
| HT29_13_GR_ChDB_0120 | Crizotinib | 10uM | 6h | DDX17 | 0.00 | 0.43 |
| HT29_13_GR_ChDB_0120 | Crizotinib | 10uM | 6h | AMD1 | 0.00 | 0.49 |
| HT29_13_GR_ChDB_0120 | Crizotinib | 10uM | 6h | NSL1 | 0.00 | 0.34 |
| HT29_13_GR_ChDB_0120 | Crizotinib | 10uM | 6h | HIRA | 0.00 | 0.32 |
| HT29_13_GR_ChDB_0120 | Crizotinib | 10uM | 6h | CYR61 | 0.00 | 0.44 |
| HT29_13_GR_ChDB_0120 | Crizotinib | 10uM | 6h | ACAP2 | 0.00 | 0.35 |
| HT29_13_GR_ChDB_0120 | Crizotinib | 10uM | 6h | LOC100506469 | 0.00 | 0.42 |
| HT29_13_GR_ChDB_0120 | Crizotinib | 10uM | 6h | GOLGA8A | 0.00 | 0.49 |
| HT29_13_GR_ChDB_0120 | Crizotinib | 10uM | 6h | ALDH1B1 | 0.00 | 0.31 |
| HT29_13_GR_ChDB_0120 | Crizotinib | 10uM | 6h | INTS5 | 0.00 | 0.43 |
| HT29_13_GR_ChDB_0120 | Crizotinib | 10uM | 6h | WDR59 | 0.00 | 0.37 |
| HT29_13_GR_ChDB_0120 | Crizotinib | 10uM | 6h | TRAF4 | 0.00 | 0.48 |
| HT29_13_GR_ChDB_0120 | Crizotinib | 10uM | 6h | GPHN | 0.00 | 0.43 |
| HT29_13_GR_ChDB_0120 | Crizotinib | 10uM | 6h | GLT8D1 | 0.00 | 0.36 |
| HT29_13_GR_ChDB_0120 | Crizotinib | 10uM | 6h | FUT4 | 0.00 | 0.31 |
| HT29_13_GR_ChDB_0120 | Crizotinib | 10uM | 6h | ATP13A3 | 0.00 | 0.33 |
| HT29_13_GR_ChDB_0120 | Crizotinib | 10uM | 6h | CDC14B | 0.00 | 0.44 |
| HT29_13_GR_ChDB_0120 | Crizotinib | 10uM | 6h | DUSP12 | 0.00 | 0.40 |
| HT29_13_GR_ChDB_0120 | Crizotinib | 10uM | 6h | CUL2 | 0.00 | 0.46 |
| HT29_13_GR_ChDB_0120 | Crizotinib | 10uM | 6h | KNTC1 | 0.00 | 0.35 |
| HT29_13_GR_ChDB_0120 | Crizotinib | 10uM | 6h | ARIH2 | 0.00 | 0.40 |
| HT29_13_GR_ChDB_0120 | Crizotinib | 10uM | 6h | S100A10 | 0.00 | 0.41 |
| HT29_13_GR_ChDB_0120 | Crizotinib | 10uM | 6h | NGLY1 | 0.00 | 0.50 |
| HT29_13_GR_ChDB_0120 | Crizotinib | 10uM | 6h | ZBTB10 | 0.00 | 5.64 |
| HT29_13_GR_ChDB_0120 | Crizotinib | 10uM | 6h | USP48 | 0.00 | 2.40 |
| HT29_13_GR_ChDB_0120 | Crizotinib | 10uM | 6h | RIPK4 | 0.00 | 2.43 |
| HT29_13_GR_ChDB_0120 | Crizotinib | 10uM | 6h | TOPORS | 0.00 | 2.37 |
| HT29_13_GR_ChDB_0120 | Crizotinib | 10uM | 6h | IFNGR1 | 0.00 | 3.04 |
| HT29_13_GR_ChDB_0120 | Crizotinib | 10uM | 6h | ARL4A | 0.00 | 5.18 |
| HT29_13_GR_ChDB_0120 | Crizotinib | 10uM | 6h | CCNL1 | 0.00 | 5.56 |
| HT29_13_GR_ChDB_0120 | Crizotinib | 10uM | 6h | TMEM159 | 0.00 | 2.04 |
| HT29_13_GR_ChDB_0120 | Crizotinib | 10uM | 6h | SEMA4C | 0.00 | 4.83 |
| HT29_13_GR_ChDB_0120 | Crizotinib | 10uM | 6h | TFF1 | 0.00 | 2.04 |
| HT29_13_GR_ChDB_0120 | Crizotinib | 10uM | 6h | MIIP | 0.00 | 0.50 |
| HT29_13_GR_ChDB_0120 | Crizotinib | 10uM | 6h | NHLRC2 | 0.00 | 0.43 |
| HT29_13_GR_ChDB_0120 | Crizotinib | 10uM | 6h | ARHGAP19 | 0.00 | 0.45 |
| HT29_13_GR_ChDB_0120 | Crizotinib | 10uM | 6h | PAQR4 | 0.00 | 0.33 |
| HT29_13_GR_ChDB_0120 | Crizotinib | 10uM | 6h | NFX1 | 0.00 | 0.47 |
| HT29_13_GR_ChDB_0120 | Crizotinib | 10uM | 6h | MED24 | 0.00 | 0.47 |
| HT29_13_GR_ChDB_0120 | Crizotinib | 10uM | 6h | ENDOD1 | 0.00 | 0.35 |
| HT29_13_GR_ChDB_0120 | Crizotinib | 10uM | 6h | OCRL | 0.00 | 0.46 |
| HT29_13_GR_ChDB_0120 | Crizotinib | 10uM | 6h | HAUS6 | 0.00 | 0.27 |
| HT29_13_GR_ChDB_0120 | Crizotinib | 10uM | 6h | UFL1 | 0.00 | 0.42 |
| HT29_13_GR_ChDB_0120 | Crizotinib | 10uM | 6h | SPTSSA | 0.00 | 0.48 |
| HT29_13_GR_ChDB_0120 | Crizotinib | 10uM | 6h | SDHAF1 | 0.00 | 0.43 |
| HT29_13_GR_ChDB_0120 | Crizotinib | 10uM | 6h | PANK3 | 0.00 | 0.19 |
| HT29_13_GR_ChDB_0120 | Crizotinib | 10uM | 6h | NSMAF | 0.00 | 0.40 |
| HT29_13_GR_ChDB_0120 | Crizotinib | 10uM | 6h | DEPDC1 | 0.00 | 0.32 |
| HT29_13_GR_ChDB_0120 | Crizotinib | 10uM | 6h | BRIP1 | 0.00 | 0.24 |
| HT29_13_GR_ChDB_0120 | Crizotinib | 10uM | 6h | ARMC1 | 0.00 | 0.48 |
| HT29_13_GR_ChDB_0120 | Crizotinib | 10uM | 6h | CEP135 | 0.00 | 0.42 |
| HT29_13_GR_ChDB_0120 | Crizotinib | 10uM | 6h | MPHOSPH6 | 0.00 | 0.23 |
| HT29_13_GR_ChDB_0120 | Crizotinib | 10uM | 6h | DEXI | 0.00 | 0.35 |
| HT29_13_GR_ChDB_0120 | Crizotinib | 10uM | 6h | ZNF239 | 0.00 | 0.17 |
| HT29_13_GR_ChDB_0120 | Crizotinib | 10uM | 6h | RIF1 | 0.00 | 0.44 |
| HT29_13_GR_ChDB_0120 | Crizotinib | 10uM | 6h | ENTPD4 | 0.00 | 0.27 |
| HT29_13_GR_ChDB_0120 | Crizotinib | 10uM | 6h | ARHGAP26 | 0.00 | 0.49 |
| HT29_13_GR_ChDB_0120 | Crizotinib | 10uM | 6h | DUSP7 | 0.00 | 0.36 |
| HT29_13_GR_ChDB_0120 | Crizotinib | 10uM | 6h | BRD1 | 0.00 | 2.76 |
| HT29_13_GR_ChDB_0120 | Crizotinib | 10uM | 6h | MTRF1 | 0.00 | 0.43 |
| HT29_13_GR_ChDB_0120 | Crizotinib | 10uM | 6h | DENND1B | 0.00 | 0.38 |
| HT29_13_GR_ChDB_0120 | Crizotinib | 10uM | 6h | PELI1 | 0.00 | 2.17 |
| HT29_13_GR_ChDB_0120 | Crizotinib | 10uM | 6h | SNIP1 | 0.00 | 3.97 |
| HT29_13_GR_ChDB_0120 | Crizotinib | 10uM | 6h | HECA | 0.00 | 3.82 |
| HT29_13_GR_ChDB_0120 | Crizotinib | 10uM | 6h | TWSG1 | 0.00 | 2.88 |
| HT29_13_GR_ChDB_0120 | Crizotinib | 10uM | 6h | CYTH2 | 0.00 | 3.38 |
| HT29_13_GR_ChDB_0120 | Crizotinib | 10uM | 6h | IRF1 | 0.00 | 5.09 |
| HT29_13_GR_ChDB_0120 | Crizotinib | 10uM | 6h | SPINK1 | 0.00 | 2.29 |
| HT29_13_GR_ChDB_0120 | Crizotinib | 10uM | 6h | OAZ2 | 0.00 | 2.12 |
| HT29_13_GR_ChDB_0120 | Crizotinib | 10uM | 6h | MORC3 | 0.00 | 2.11 |
| HT29_13_GR_ChDB_0120 | Crizotinib | 10uM | 6h | LFNG | 0.00 | 4.99 |
| HT29_13_GR_ChDB_0120 | Crizotinib | 10uM | 6h | SIRT1 | 0.00 | 3.98 |
| HT29_13_GR_ChDB_0120 | Crizotinib | 10uM | 6h | MAPK1 | 0.00 | 2.61 |
| HT29_13_GR_ChDB_0120 | Crizotinib | 10uM | 6h | CCNL2 | 0.00 | 2.10 |
| HT29_13_GR_ChDB_0120 | Crizotinib | 10uM | 6h | DCAF8 | 0.00 | 2.74 |
| HT29_13_GR_ChDB_0120 | Crizotinib | 10uM | 6h | TMEM51 | 0.00 | 2.86 |
| HT29_13_GR_ChDB_0120 | Crizotinib | 10uM | 6h | JHDM1D | 0.00 | 3.83 |
| HT29_13_GR_ChDB_0120 | Crizotinib | 10uM | 6h | RBM4 | 0.00 | 6.94 |
| HT29_13_GR_ChDB_0120 | Crizotinib | 10uM | 6h | IDH3G | 0.00 | 2.01 |
| HT29_13_GR_ChDB_0120 | Crizotinib | 10uM | 6h | KIF5B | 0.00 | 3.00 |
| HT29_13_GR_ChDB_0120 | Crizotinib | 10uM | 6h | TXNDC9 | 0.00 | 3.17 |
| HT29_13_GR_ChDB_0120 | Crizotinib | 10uM | 6h | EDN1 | 0.00 | 3.89 |
| HT29_13_GR_ChDB_0120 | Crizotinib | 10uM | 6h | RNF38 | 0.00 | 3.26 |
| HT29_13_GR_ChDB_0120 | Crizotinib | 10uM | 6h | PTMA | 0.00 | 2.40 |
| HT29_13_GR_ChDB_0120 | Crizotinib | 10uM | 6h | ALCAM | 0.00 | 2.71 |
| HT29_13_GR_ChDB_0120 | Crizotinib | 10uM | 6h | SNX10 | 0.00 | 0.48 |
| HT29_13_GR_ChDB_0120 | Crizotinib | 10uM | 6h | ORC2 | 0.00 | 0.39 |
| HT29_13_GR_ChDB_0120 | Crizotinib | 10uM | 6h | PAGR1 | 0.00 | 0.47 |
| HT29_13_GR_ChDB_0120 | Crizotinib | 10uM | 6h | FAM203A | 0.00 | 0.47 |
| HT29_13_GR_ChDB_0120 | Crizotinib | 10uM | 6h | HMBS | 0.00 | 0.39 |
| HT29_13_GR_ChDB_0120 | Crizotinib | 10uM | 6h | IVNS1ABP | 0.00 | 0.35 |
| HT29_13_GR_ChDB_0120 | Crizotinib | 10uM | 6h | ACTR3B | 0.00 | 0.30 |
| HT29_13_GR_ChDB_0120 | Crizotinib | 10uM | 6h | MON2 | 0.00 | 0.47 |
| HT29_13_GR_ChDB_0120 | Crizotinib | 10uM | 6h | TMEM131 | 0.00 | 0.33 |
| HT29_13_GR_ChDB_0120 | Crizotinib | 10uM | 6h | DSN1 | 0.00 | 0.23 |
| HT29_13_GR_ChDB_0120 | Crizotinib | 10uM | 6h | DLEU1 | 0.00 | 0.44 |
| HT29_13_GR_ChDB_0120 | Crizotinib | 10uM | 6h | APOOL | 0.00 | 0.48 |
| HT29_13_GR_ChDB_0120 | Crizotinib | 10uM | 6h | ALG6 | 0.00 | 0.34 |
| HT29_13_GR_ChDB_0120 | Crizotinib | 10uM | 6h | NOL9 | 0.00 | 0.40 |
| HT29_13_GR_ChDB_0120 | Crizotinib | 10uM | 6h | URB2 | 0.00 | 0.41 |
| HT29_13_GR_ChDB_0120 | Crizotinib | 10uM | 6h | FAM49B | 0.00 | 0.46 |
| HT29_13_GR_ChDB_0120 | Crizotinib | 10uM | 6h | SEC62 | 0.00 | 0.39 |
| HT29_13_GR_ChDB_0120 | Crizotinib | 10uM | 6h | RBFOX2 | 0.00 | 0.39 |
| HT29_13_GR_ChDB_0120 | Crizotinib | 10uM | 6h | TUBGCP5 | 0.00 | 0.45 |
| HT29_13_GR_ChDB_0120 | Crizotinib | 10uM | 6h | ATP11A | 0.00 | 0.34 |
| HT29_13_GR_ChDB_0120 | Crizotinib | 10uM | 6h | UQCC | 0.00 | 0.46 |
| HT29_13_GR_ChDB_0120 | Crizotinib | 10uM | 6h | MYO1B | 0.00 | 0.45 |
| HT29_13_GR_ChDB_0120 | Crizotinib | 10uM | 6h | NKTR | 0.00 | 0.47 |
| HT29_13_GR_ChDB_0120 | Crizotinib | 10uM | 6h | TMEM223 | 0.00 | 0.24 |
| HT29_13_GR_ChDB_0120 | Crizotinib | 10uM | 6h | CAPRIN2 | 0.00 | 0.42 |
| HT29_13_GR_ChDB_0120 | Crizotinib | 10uM | 6h | THOC1 | 0.00 | 0.37 |
| HT29_13_GR_ChDB_0120 | Crizotinib | 10uM | 6h | DUS4L | 0.00 | 0.34 |
| HT29_13_GR_ChDB_0120 | Crizotinib | 10uM | 6h | C17orf62 | 0.00 | 0.37 |
| HT29_13_GR_ChDB_0120 | Crizotinib | 10uM | 6h | ELL3 | 0.00 | 0.31 |
| HT29_13_GR_ChDB_0120 | Crizotinib | 10uM | 6h | SAP30L | 0.00 | 0.42 |
| HT29_13_GR_ChDB_0120 | Crizotinib | 10uM | 6h | RBM26 | 0.00 | 0.46 |
| HT29_13_GR_ChDB_0120 | Crizotinib | 10uM | 6h | ELF2 | 0.00 | 3.43 |
| HT29_13_GR_ChDB_0120 | Crizotinib | 10uM | 6h | C12orf49 | 0.00 | 2.16 |
| HT29_13_GR_ChDB_0120 | Crizotinib | 10uM | 6h | C1orf106 | 0.00 | 2.21 |
| HT29_13_GR_ChDB_0120 | Crizotinib | 10uM | 6h | MAPK6 | 0.00 | 2.13 |
| HT29_13_GR_ChDB_0120 | Crizotinib | 10uM | 6h | TOX3 | 0.00 | 3.54 |
| HT29_13_GR_ChDB_0120 | Crizotinib | 10uM | 6h | DGKH | 0.00 | 2.65 |
| HT29_13_GR_ChDB_0120 | Crizotinib | 10uM | 6h | ACVR2A | 0.00 | 2.32 |
| HT29_13_GR_ChDB_0120 | Crizotinib | 10uM | 6h | RNF19A | 0.00 | 4.87 |
| HT29_13_GR_ChDB_0120 | Crizotinib | 10uM | 6h | ZFAND3 | 0.00 | 2.67 |
| HT29_13_GR_ChDB_0120 | Crizotinib | 10uM | 6h | JMJD1C | 0.00 | 4.96 |
| HT29_13_GR_ChDB_0120 | Crizotinib | 10uM | 6h | SMIM14 | 0.00 | 2.02 |
| HT29_13_GR_ChDB_0120 | Crizotinib | 10uM | 6h | GGNBP2 | 0.00 | 2.51 |
| HT29_13_GR_ChDB_0120 | Crizotinib | 10uM | 6h | USP53 | 0.00 | 3.88 |
| HT29_13_GR_ChDB_0120 | Crizotinib | 10uM | 6h | ZBTB6 | 0.00 | 2.17 |
| HT29_13_GR_ChDB_0120 | Crizotinib | 10uM | 6h | GMCL1 | 0.00 | 0.31 |
| HT29_13_GR_ChDB_0120 | Crizotinib | 10uM | 6h | EDEM3 | 0.00 | 0.38 |
| HT29_13_GR_ChDB_0120 | Crizotinib | 10uM | 6h | SHMT1 | 0.00 | 0.49 |
| HT29_13_GR_ChDB_0120 | Crizotinib | 10uM | 6h | ANK3 | 0.00 | 0.34 |
| HT29_13_GR_ChDB_0120 | Crizotinib | 10uM | 6h | PPIP5K2 | 0.00 | 0.37 |
| HT29_13_GR_ChDB_0120 | Crizotinib | 10uM | 6h | PSD3 | 0.00 | 0.35 |
| HT29_13_GR_ChDB_0120 | Crizotinib | 10uM | 6h | DUS2L | 0.00 | 0.49 |
| HT29_13_GR_ChDB_0120 | Crizotinib | 10uM | 6h | CCP110 | 0.00 | 0.38 |
| HT29_13_GR_ChDB_0120 | Crizotinib | 10uM | 6h | STX6 | 0.00 | 0.40 |
| HT29_13_GR_ChDB_0120 | Crizotinib | 10uM | 6h | NUP214 | 0.00 | 0.36 |
| HT29_13_GR_ChDB_0120 | Crizotinib | 10uM | 6h | NME7 | 0.00 | 0.45 |
| HT29_13_GR_ChDB_0120 | Crizotinib | 10uM | 6h | GALNT2 | 0.00 | 0.42 |
| HT29_13_GR_ChDB_0120 | Crizotinib | 10uM | 6h | EPS15 | 0.00 | 0.28 |
| HT29_13_GR_ChDB_0120 | Crizotinib | 10uM | 6h | TTC27 | 0.00 | 0.50 |
| HT29_13_GR_ChDB_0120 | Crizotinib | 10uM | 6h | ATP2C2 | 0.00 | 0.45 |
| HT29_13_GR_ChDB_0120 | Crizotinib | 10uM | 6h | RERE | 0.00 | 0.50 |
| HT29_13_GR_ChDB_0120 | Crizotinib | 10uM | 6h | SELT | 0.00 | 0.41 |
| HT29_13_GR_ChDB_0120 | Crizotinib | 10uM | 6h | UNC50 | 0.00 | 0.23 |
| HT29_13_GR_ChDB_0120 | Crizotinib | 10uM | 6h | DNAJC24 | 0.00 | 0.25 |
| HT29_13_GR_ChDB_0120 | Crizotinib | 10uM | 6h | ITGB6 | 0.00 | 0.35 |
| HT29_13_GR_ChDB_0120 | Crizotinib | 10uM | 6h | FAM8A1 | 0.00 | 0.32 |
| HT29_13_GR_ChDB_0120 | Crizotinib | 10uM | 6h | TRAPPC10 | 0.00 | 0.47 |
| HT29_13_GR_ChDB_0120 | Crizotinib | 10uM | 6h | RIN2 | 0.00 | 0.28 |
| HT29_13_GR_ChDB_0120 | Crizotinib | 10uM | 6h | CLOCK | 0.00 | 0.33 |
| HT29_13_GR_ChDB_0120 | Crizotinib | 10uM | 6h | ZNF638 | 0.00 | 0.40 |
| HT29_13_GR_ChDB_0120 | Crizotinib | 10uM | 6h | SEMA3C | 0.00 | 0.50 |
| HT29_13_GR_ChDB_0120 | Crizotinib | 10uM | 6h | EHBP1 | 0.00 | 0.27 |
| HT29_13_GR_ChDB_0120 | Crizotinib | 10uM | 6h | ELOVL1 | 0.00 | 0.47 |
| HT29_13_GR_ChDB_0120 | Crizotinib | 10uM | 6h | FAM216A | 0.00 | 0.42 |
| HT29_13_GR_ChDB_0120 | Crizotinib | 10uM | 6h | TGFBR3 | 0.00 | 2.34 |
| HT29_13_GR_ChDB_0120 | Crizotinib | 10uM | 6h | YAP1 | 0.00 | 4.59 |
| HT29_13_GR_ChDB_0120 | Crizotinib | 10uM | 6h | GTF2I | 0.00 | 2.08 |
| HT29_13_GR_ChDB_0120 | Crizotinib | 10uM | 6h | TMOD3 | 0.00 | 3.90 |
| HT29_13_GR_ChDB_0120 | Crizotinib | 10uM | 6h | RNF103 | 0.00 | 4.38 |
| HT29_13_GR_ChDB_0120 | Crizotinib | 10uM | 6h | DFFA | 0.00 | 0.50 |
| HT29_13_GR_ChDB_0120 | Crizotinib | 10uM | 6h | GPRC5A | 0.00 | 0.44 |
| HT29_13_GR_ChDB_0120 | Crizotinib | 10uM | 6h | PCSK5 | 0.00 | 0.49 |
| HT29_13_GR_ChDB_0120 | Crizotinib | 10uM | 6h | NACC2 | 0.00 | 0.33 |
| HT29_13_GR_ChDB_0120 | Crizotinib | 10uM | 6h | KLF10 | 0.00 | 2.52 |
| HT29_13_GR_ChDB_0120 | Crizotinib | 10uM | 6h | RHOF | 0.00 | 2.25 |
| HT29_13_GR_ChDB_0120 | Crizotinib | 10uM | 6h | BANP | 0.00 | 2.26 |
| HT29_13_GR_ChDB_0120 | Crizotinib | 10uM | 6h | PIK3CA | 0.00 | 2.94 |
| HT29_13_GR_ChDB_0120 | Crizotinib | 10uM | 6h | ZBTB7C | 0.00 | 2.38 |
| HT29_13_GR_ChDB_0120 | Crizotinib | 10uM | 6h | IP6K2 | 0.00 | 2.95 |
| HT29_13_GR_ChDB_0120 | Crizotinib | 10uM | 6h | LINS | 0.00 | 3.89 |
| HT29_13_GR_ChDB_0120 | Crizotinib | 10uM | 6h | ARID4A | 0.00 | 2.40 |
| HT29_13_GR_ChDB_0120 | Crizotinib | 10uM | 6h | FAM102A | 0.00 | 2.99 |
| HT29_13_GR_ChDB_0120 | Crizotinib | 10uM | 6h | DCP2 | 0.00 | 2.95 |
| HT29_13_GR_ChDB_0120 | Crizotinib | 10uM | 6h | STAG2 | 0.00 | 2.11 |
| HT29_13_GR_ChDB_0120 | Crizotinib | 10uM | 6h | ZNF227 | 0.00 | 2.31 |
| HT29_13_GR_ChDB_0120 | Crizotinib | 10uM | 6h | HINFP | 0.00 | 3.07 |
| HT29_13_GR_ChDB_0120 | Crizotinib | 10uM | 6h | SOX9 | 0.00 | 2.14 |
| HT29_13_GR_ChDB_0120 | Crizotinib | 10uM | 6h | SIGIRR | 0.00 | 0.45 |
| HT29_13_GR_ChDB_0120 | Crizotinib | 10uM | 6h | NUPL2 | 0.00 | 0.36 |
| HT29_13_GR_ChDB_0120 | Crizotinib | 10uM | 6h | SFXN1 | 0.00 | 0.27 |
| HT29_13_GR_ChDB_0120 | Crizotinib | 10uM | 6h | REPIN1 | 0.00 | 0.41 |
| HT29_13_GR_ChDB_0120 | Crizotinib | 10uM | 6h | TFB1M | 0.00 | 0.38 |
| HT29_13_GR_ChDB_0120 | Crizotinib | 10uM | 6h | SNX19 | 0.00 | 0.43 |
| HT29_13_GR_ChDB_0120 | Crizotinib | 10uM | 6h | SCML1 | 0.00 | 0.46 |
| HT29_13_GR_ChDB_0120 | Crizotinib | 10uM | 6h | TBC1D30 | 0.00 | 0.37 |
| HT29_13_GR_ChDB_0120 | Crizotinib | 10uM | 6h | PHC3 | 0.00 | 0.28 |
| HT29_13_GR_ChDB_0120 | Crizotinib | 10uM | 6h | SLC7A6 | 0.00 | 0.27 |
| HT29_13_GR_ChDB_0120 | Crizotinib | 10uM | 6h | CTPS1 | 0.00 | 0.25 |
| HT29_13_GR_ChDB_0120 | Crizotinib | 10uM | 6h | RRP9 | 0.00 | 0.48 |
| HT29_13_GR_ChDB_0120 | Crizotinib | 10uM | 6h | SELRC1 | 0.00 | 0.42 |
| HT29_13_GR_ChDB_0120 | Crizotinib | 10uM | 6h | CEP192 | 0.00 | 0.26 |
| HT29_13_GR_ChDB_0120 | Crizotinib | 10uM | 6h | MARC1 | 0.00 | 0.48 |
| HT29_13_GR_ChDB_0120 | Crizotinib | 10uM | 6h | BCAP29 | 0.00 | 0.33 |
| HT29_13_GR_ChDB_0120 | Crizotinib | 10uM | 6h | UBA5 | 0.00 | 0.48 |
| HT29_13_GR_ChDB_0120 | Crizotinib | 10uM | 6h | UBE2G1 | 0.00 | 0.45 |
| HT29_13_GR_ChDB_0120 | Crizotinib | 10uM | 6h | ARL5A | 0.00 | 0.48 |
| HT29_13_GR_ChDB_0120 | Crizotinib | 10uM | 6h | PRKCI | 0.00 | 0.48 |
| HT29_13_GR_ChDB_0120 | Crizotinib | 10uM | 6h | METTL8 | 0.00 | 0.34 |
| HT29_13_GR_ChDB_0120 | Crizotinib | 10uM | 6h | NREP | 0.00 | 0.32 |
| HT29_13_GR_ChDB_0120 | Crizotinib | 10uM | 6h | ZNF587B | 0.00 | 0.33 |
| HT29_13_GR_ChDB_0120 | Crizotinib | 10uM | 6h | EFNB2 | 0.00 | 0.29 |
| HT29_13_GR_ChDB_0120 | Crizotinib | 10uM | 6h | B4GALT6 | 0.00 | 0.28 |
| HT29_13_GR_ChDB_0120 | Crizotinib | 10uM | 6h | STX16 | 0.00 | 0.41 |
| HT29_13_GR_ChDB_0120 | Crizotinib | 10uM | 6h | IL1RN | 0.00 | 0.42 |
| HT29_13_GR_ChDB_0120 | Crizotinib | 10uM | 6h | TNK2 | 0.00 | 0.47 |
| HT29_13_GR_ChDB_0120 | Crizotinib | 10uM | 6h | UBAC1 | 0.00 | 0.43 |
| HT29_13_GR_ChDB_0120 | Crizotinib | 10uM | 6h | STX12 | 0.00 | 0.48 |
| HT29_13_GR_ChDB_0120 | Crizotinib | 10uM | 6h | CEP76 | 0.00 | 0.46 |
| HT29_13_GR_ChDB_0120 | Crizotinib | 10uM | 6h | UBA3 | 0.00 | 0.44 |
| HT29_13_GR_ChDB_0120 | Crizotinib | 10uM | 6h | KIAA1598 | 0.00 | 0.41 |
| HT29_13_GR_ChDB_0120 | Crizotinib | 10uM | 6h | STEAP3 | 0.00 | 0.45 |
| HT29_13_GR_ChDB_0120 | Crizotinib | 10uM | 6h | RSAD1 | 0.00 | 0.30 |
| HT29_13_GR_ChDB_0120 | Crizotinib | 10uM | 6h | TIPRL | 0.00 | 0.36 |
| HT29_13_GR_ChDB_0120 | Crizotinib | 10uM | 6h | TRMT13 | 0.00 | 0.41 |
| HT29_13_GR_ChDB_0120 | Crizotinib | 10uM | 6h | INADL | 0.00 | 0.29 |
| HT29_13_GR_ChDB_0120 | Crizotinib | 10uM | 6h | CCDC51 | 0.00 | 0.36 |
| HT29_13_GR_ChDB_0120 | Crizotinib | 10uM | 6h | FAM168B | 0.00 | 3.21 |
| HT29_13_GR_ChDB_0120 | Crizotinib | 10uM | 6h | TTC13 | 0.00 | 0.38 |
| HT29_13_GR_ChDB_0120 | Crizotinib | 10uM | 6h | ASAP1 | 0.00 | 0.38 |
| HT29_13_GR_ChDB_0120 | Crizotinib | 10uM | 6h | SECISBP2 | 0.00 | 0.41 |
| HT29_13_GR_ChDB_0120 | Crizotinib | 10uM | 6h | XIST | 0.00 | 0.44 |
| HT29_13_GR_ChDB_0120 | Crizotinib | 10uM | 6h | CPOX | 0.00 | 0.41 |
| HT29_13_GR_ChDB_0120 | Crizotinib | 10uM | 6h | UBE2G2 | 0.00 | 0.18 |
| HT29_13_GR_ChDB_0120 | Crizotinib | 10uM | 6h | LINC00675 | 0.00 | 2.14 |
| HT29_13_GR_ChDB_0120 | Crizotinib | 10uM | 6h | CITED2 | 0.00 | 2.35 |
| HT29_13_GR_ChDB_0120 | Crizotinib | 10uM | 6h | ZDHHC7 | 0.00 | 2.76 |
| HT29_13_GR_ChDB_0120 | Crizotinib | 10uM | 6h | SMPDL3A | 0.00 | 3.92 |
| HT29_13_GR_ChDB_0120 | Crizotinib | 10uM | 6h | ZNF410 | 0.00 | 2.24 |
| HT29_13_GR_ChDB_0120 | Crizotinib | 10uM | 6h | ZNF394 | 0.00 | 2.75 |
| HT29_13_GR_ChDB_0120 | Crizotinib | 10uM | 6h | PRRC2B | 0.00 | 0.44 |
| HT29_13_GR_ChDB_0120 | Crizotinib | 10uM | 6h | CSTF3 | 0.00 | 0.42 |
| HT29_13_GR_ChDB_0120 | Crizotinib | 10uM | 6h | CHRNA5 | 0.00 | 0.47 |
| HT29_13_GR_ChDB_0120 | Crizotinib | 10uM | 6h | PGRMC2 | 0.00 | 0.49 |
| HT29_13_GR_ChDB_0120 | Crizotinib | 10uM | 6h | PHACTR2 | 0.00 | 0.45 |
| HT29_13_GR_ChDB_0120 | Crizotinib | 10uM | 6h | C2orf68 | 0.00 | 0.40 |
| HT29_13_GR_ChDB_0120 | Crizotinib | 10uM | 6h | RPP25 | 0.00 | 0.26 |
| HT29_13_GR_ChDB_0120 | Crizotinib | 10uM | 6h | SLC6A6 | 0.00 | 0.22 |
| HT29_13_GR_ChDB_0120 | Crizotinib | 10uM | 6h | RPP30 | 0.00 | 0.32 |
| HT29_13_GR_ChDB_0120 | Crizotinib | 10uM | 6h | ZNF148 | 0.00 | 0.42 |
| HT29_13_GR_ChDB_0120 | Crizotinib | 10uM | 6h | FAR2 | 0.00 | 0.46 |
| HT29_13_GR_ChDB_0120 | Crizotinib | 10uM | 6h | RHOBTB3 | 0.00 | 0.30 |
| HT29_13_GR_ChDB_0120 | Crizotinib | 10uM | 6h | ZNF518A | 0.00 | 0.26 |
| HT29_13_GR_ChDB_0120 | Crizotinib | 10uM | 6h | CDK12 | 0.00 | 0.42 |
| HT29_13_GR_ChDB_0120 | Crizotinib | 10uM | 6h | DCLRE1A | 0.00 | 0.48 |
| HT29_13_GR_ChDB_0120 | Crizotinib | 10uM | 6h | NIPAL3 | 0.00 | 0.42 |
| HT29_13_GR_ChDB_0120 | Crizotinib | 10uM | 6h | MTR | 0.00 | 0.26 |
| HT29_13_GR_ChDB_0120 | Crizotinib | 10uM | 6h | MICAL2 | 0.00 | 0.43 |
| HT29_13_GR_ChDB_0120 | Crizotinib | 10uM | 6h | SETD6 | 0.00 | 0.32 |
| HT29_13_GR_ChDB_0120 | Crizotinib | 10uM | 6h | CNOT6 | 0.00 | 0.41 |
| HT29_13_GR_ChDB_0120 | Crizotinib | 10uM | 6h | GTF2H2 | 0.00 | 0.18 |
| HT29_13_GR_ChDB_0120 | Crizotinib | 10uM | 6h | CPT2 | 0.00 | 0.47 |
| HT29_13_GR_ChDB_0120 | Crizotinib | 10uM | 6h | RPRD1A | 0.00 | 0.49 |
| HT29_13_GR_ChDB_0120 | Crizotinib | 10uM | 6h | RBM12 | 0.00 | 0.30 |
| HT29_13_GR_ChDB_0120 | Crizotinib | 10uM | 6h | TEX30 | 0.00 | 0.44 |
| HT29_13_GR_ChDB_0120 | Crizotinib | 10uM | 6h | SLC4A7 | 0.00 | 0.28 |
| HT29_13_GR_ChDB_0120 | Crizotinib | 10uM | 6h | UBC | 0.00 | 2.06 |
| HT29_13_GR_ChDB_0120 | Crizotinib | 10uM | 6h | ZNF350 | 0.00 | 2.76 |
| HT29_13_GR_ChDB_0120 | Crizotinib | 10uM | 6h | IRS2 | 0.00 | 4.80 |
| HT29_13_GR_ChDB_0120 | Crizotinib | 10uM | 6h | CDKN2AIP | 0.00 | 2.90 |
| HT29_13_GR_ChDB_0120 | Crizotinib | 10uM | 6h | CHEK1 | 0.00 | 2.24 |
| HT29_13_GR_ChDB_0120 | Crizotinib | 10uM | 6h | TRIOBP | 0.00 | 0.40 |
| HT29_13_GR_ChDB_0120 | Crizotinib | 10uM | 6h | LRRC8D | 0.00 | 0.41 |
| HT29_13_GR_ChDB_0120 | Crizotinib | 10uM | 6h | RARS | 0.00 | 0.48 |
| HT29_13_GR_ChDB_0120 | Crizotinib | 10uM | 6h | EPB41L1 | 0.00 | 0.39 |
| HT29_13_GR_ChDB_0120 | Crizotinib | 10uM | 6h | SLC12A2 | 0.00 | 2.57 |
| HT29_13_GR_ChDB_0120 | Crizotinib | 10uM | 6h | ATP6V1G1 | 0.00 | 2.06 |
| HT29_13_GR_ChDB_0120 | Crizotinib | 10uM | 6h | PRPF3 | 0.00 | 3.42 |
| HT29_13_GR_ChDB_0120 | Crizotinib | 10uM | 6h | KBTBD2 | 0.00 | 2.83 |
| HT29_13_GR_ChDB_0120 | Crizotinib | 10uM | 6h | BCAR3 | 0.00 | 2.16 |
| HT29_13_GR_ChDB_0120 | Crizotinib | 10uM | 6h | CDKN2B | 0.00 | 13.09 |
| HT29_13_GR_ChDB_0120 | Crizotinib | 10uM | 6h | LRIG1 | 0.00 | 2.83 |
| HT29_13_GR_ChDB_0120 | Crizotinib | 10uM | 6h | DNAJB14 | 0.00 | 2.19 |
| HT29_13_GR_ChDB_0120 | Crizotinib | 10uM | 6h | MAD2L1BP | 0.00 | 2.46 |
| HT29_13_GR_ChDB_0120 | Crizotinib | 10uM | 6h | SDC4 | 0.00 | 4.78 |
| HT29_13_GR_ChDB_0120 | Crizotinib | 10uM | 6h | SLC30A1 | 0.00 | 3.70 |
| HT29_13_GR_ChDB_0120 | Crizotinib | 10uM | 6h | RC3H1 | 0.00 | 6.48 |
| HT29_13_GR_ChDB_0120 | Crizotinib | 10uM | 6h | SLC38A2 | 0.00 | 3.70 |
| HT29_13_GR_ChDB_0120 | Crizotinib | 10uM | 6h | GTF2B | 0.00 | 2.68 |
| HT29_13_GR_ChDB_0120 | Crizotinib | 10uM | 6h | DHX8 | 0.00 | 3.12 |
| HT29_13_GR_ChDB_0120 | Crizotinib | 10uM | 6h | TBCC | 0.00 | 2.23 |
| HT29_13_GR_ChDB_0120 | Crizotinib | 10uM | 6h | CUTC | 0.00 | 0.41 |
| HT29_13_GR_ChDB_0120 | Crizotinib | 10uM | 6h | ASB13 | 0.00 | 0.28 |
| HT29_13_GR_ChDB_0120 | Crizotinib | 10uM | 6h | ERLIN2 | 0.00 | 0.36 |
| HT29_13_GR_ChDB_0120 | Crizotinib | 10uM | 6h | DCAF16 | 0.00 | 0.25 |
| HT29_13_GR_ChDB_0120 | Crizotinib | 10uM | 6h | CDS1 | 0.00 | 0.32 |
| HT29_13_GR_ChDB_0120 | Crizotinib | 10uM | 6h | CCNO | 0.00 | 0.49 |
| HT29_13_GR_ChDB_0120 | Crizotinib | 10uM | 6h | XPA | 0.00 | 0.48 |
| HT29_13_GR_ChDB_0120 | Crizotinib | 10uM | 6h | SLC37A1 | 0.00 | 0.25 |
| HT29_13_GR_ChDB_0120 | Crizotinib | 10uM | 6h | ALDH1A3 | 0.00 | 0.25 |
| HT29_13_GR_ChDB_0120 | Crizotinib | 10uM | 6h | PTCD2 | 0.00 | 0.21 |
| HT29_13_GR_ChDB_0120 | Crizotinib | 10uM | 6h | CDC42SE1 | 0.00 | 0.42 |
| HT29_13_GR_ChDB_0120 | Crizotinib | 10uM | 6h | PITPNA | 0.00 | 0.43 |
| HT29_13_GR_ChDB_0120 | Crizotinib | 10uM | 6h | GALNT3 | 0.00 | 0.44 |
| HT29_13_GR_ChDB_0120 | Crizotinib | 10uM | 6h | DMXL1 | 0.00 | 0.30 |
| HT29_13_GR_ChDB_0120 | Crizotinib | 10uM | 6h | VPS54 | 0.00 | 0.49 |
| HT29_13_GR_ChDB_0120 | Crizotinib | 10uM | 6h | CENPJ | 0.00 | 0.36 |
| HT29_13_GR_ChDB_0120 | Crizotinib | 10uM | 6h | PIGV | 0.00 | 0.47 |
| HT29_13_GR_ChDB_0120 | Crizotinib | 10uM | 6h | GALNT6 | 0.00 | 0.50 |
| HT29_13_GR_ChDB_0120 | Crizotinib | 10uM | 6h | ATP2B1 | 0.00 | 0.23 |
| HT29_13_GR_ChDB_0120 | Crizotinib | 10uM | 6h | MUM1 | 0.00 | 0.40 |
| HT29_13_GR_ChDB_0120 | Crizotinib | 10uM | 6h | HERC2 | 0.00 | 0.50 |
| HT29_13_GR_ChDB_0120 | Crizotinib | 10uM | 6h | C4orf27 | 0.00 | 0.39 |
| HT29_13_GR_ChDB_0120 | Crizotinib | 10uM | 6h | DMXL2 | 0.00 | 0.40 |
| HT29_13_GR_ChDB_0120 | Crizotinib | 10uM | 6h | FPGT | 0.00 | 0.48 |
| HT29_13_GR_ChDB_0120 | Crizotinib | 10uM | 6h | IL17RB | 0.00 | 0.45 |
| HT29_13_GR_ChDB_0120 | Crizotinib | 10uM | 6h | RAB28 | 0.00 | 0.42 |
| HT29_13_GR_ChDB_0120 | Crizotinib | 10uM | 6h | TERF2 | 0.00 | 0.45 |
| HT29_13_GR_ChDB_0120 | Crizotinib | 10uM | 6h | EFNA5 | 0.00 | 0.27 |
| HT29_13_GR_ChDB_0120 | Crizotinib | 10uM | 6h | PDE8A | 0.00 | 0.42 |
| HT29_13_GR_ChDB_0120 | Crizotinib | 10uM | 6h | ZCCHC11 | 0.00 | 0.37 |
| HT29_13_GR_ChDB_0120 | Crizotinib | 10uM | 6h | TMEM33 | 0.00 | 0.42 |
| HT29_13_GR_ChDB_0120 | Crizotinib | 10uM | 6h | IST1 | 0.00 | 2.68 |
| HT29_13_GR_ChDB_0120 | Crizotinib | 10uM | 6h | YPEL5 | 0.00 | 5.28 |
| HT29_13_GR_ChDB_0120 | Crizotinib | 10uM | 6h | C6orf48 | 0.00 | 2.02 |
| HT29_13_GR_ChDB_0120 | Crizotinib | 10uM | 6h | TOR1AIP2 | 0.00 | 2.29 |
| HT29_13_GR_ChDB_0120 | Crizotinib | 10uM | 6h | PCMTD2 | 0.00 | 0.36 |
| HT29_13_GR_ChDB_0120 | Crizotinib | 10uM | 6h | KATNBL1 | 0.00 | 0.38 |
| HT29_13_GR_ChDB_0120 | Crizotinib | 10uM | 6h | CEP104 | 0.00 | 0.43 |
| HT29_13_GR_ChDB_0120 | Crizotinib | 10uM | 6h | RPAP2 | 0.00 | 0.30 |
| HT29_13_GR_ChDB_0120 | Crizotinib | 10uM | 6h | SLCO1B3 | 0.00 | 0.36 |
| HT29_13_GR_ChDB_0120 | Crizotinib | 10uM | 6h | TBCCD1 | 0.00 | 0.44 |
| HT29_13_GR_ChDB_0120 | Crizotinib | 10uM | 6h | RALGAPB | 0.00 | 0.34 |
| HT29_13_GR_ChDB_0120 | Crizotinib | 10uM | 6h | BHLHE41 | 0.00 | 0.43 |
| HT29_13_GR_ChDB_0120 | Crizotinib | 10uM | 6h | FLNB | 0.00 | 0.37 |
| HT29_13_GR_ChDB_0120 | Crizotinib | 10uM | 6h | TMC5 | 0.00 | 0.45 |
| HT29_13_GR_ChDB_0120 | Crizotinib | 10uM | 6h | GPATCH4 | 0.00 | 0.48 |
| HT29_13_GR_ChDB_0120 | Crizotinib | 10uM | 6h | PIKFYVE | 0.00 | 0.47 |
| HT29_13_GR_ChDB_0120 | Crizotinib | 10uM | 6h | UGGT2 | 0.00 | 0.42 |
| HT29_13_GR_ChDB_0120 | Crizotinib | 10uM | 6h | LEPROTL1 | 0.00 | 0.43 |
| HT29_13_GR_ChDB_0120 | Crizotinib | 10uM | 6h | NPIPA1 | 0.00 | 0.35 |
| HT29_13_GR_ChDB_0120 | Crizotinib | 10uM | 6h | UTP20 | 0.00 | 0.22 |
| HT29_13_GR_ChDB_0120 | Crizotinib | 10uM | 6h | XPO6 | 0.00 | 0.48 |
| HT29_13_GR_ChDB_0120 | Crizotinib | 10uM | 6h | GOLPH3L | 0.00 | 2.61 |
| HT29_13_GR_ChDB_0120 | Crizotinib | 10uM | 6h | DCTN4 | 0.00 | 2.07 |
| HT29_13_GR_ChDB_0120 | Crizotinib | 10uM | 6h | KIAA1551 | 0.00 | 3.03 |
| HT29_13_GR_ChDB_0120 | Crizotinib | 10uM | 6h | EFNA1 | 0.00 | 10.43 |
| HT29_13_GR_ChDB_0120 | Crizotinib | 10uM | 6h | SYBU | 0.00 | 2.85 |
| HT29_13_GR_ChDB_0120 | Crizotinib | 10uM | 6h | C1orf116 | 0.00 | 2.48 |
| HT29_13_GR_ChDB_0120 | Crizotinib | 10uM | 6h | ZBTB11 | 0.00 | 3.07 |
| HT29_13_GR_ChDB_0120 | Crizotinib | 10uM | 6h | MSMO1 | 0.00 | 2.12 |
| HT29_13_GR_ChDB_0120 | Crizotinib | 10uM | 6h | SHOC2 | 0.00 | 2.68 |
| HT29_13_GR_ChDB_0120 | Crizotinib | 10uM | 6h | C1orf63 | 0.00 | 2.67 |
| HT29_13_GR_ChDB_0120 | Crizotinib | 10uM | 6h | CBR4 | 0.00 | 0.34 |
| HT29_13_GR_ChDB_0120 | Crizotinib | 10uM | 6h | MPHOSPH8 | 0.00 | 0.36 |
| HT29_13_GR_ChDB_0120 | Crizotinib | 10uM | 6h | FBXL15 | 0.00 | 0.48 |
| HT29_13_GR_ChDB_0120 | Crizotinib | 10uM | 6h | SNAPC4 | 0.00 | 0.47 |
| HT29_13_GR_ChDB_0120 | Crizotinib | 10uM | 6h | TMEM43 | 0.00 | 0.47 |
| HT29_13_GR_ChDB_0120 | Crizotinib | 10uM | 6h | TMEM243 | 0.00 | 0.46 |
| HT29_13_GR_ChDB_0120 | Crizotinib | 10uM | 6h | SAC3D1 | 0.00 | 0.34 |
| HT29_13_GR_ChDB_0120 | Crizotinib | 10uM | 6h | PROSER1 | 0.00 | 0.30 |
| HT29_13_GR_ChDB_0120 | Crizotinib | 10uM | 6h | SMCO4 | 0.00 | 0.30 |
| HT29_13_GR_ChDB_0120 | Crizotinib | 10uM | 6h | MYO19 | 0.00 | 0.36 |
| HT29_13_GR_ChDB_0120 | Crizotinib | 10uM | 6h | ZFC3H1 | 0.00 | 0.41 |
| HT29_13_GR_ChDB_0120 | Crizotinib | 10uM | 6h | COG5 | 0.00 | 0.48 |
| HT29_13_GR_ChDB_0120 | Crizotinib | 10uM | 6h | USO1 | 0.00 | 0.42 |
| HT29_13_GR_ChDB_0120 | Crizotinib | 10uM | 6h | IFT88 | 0.00 | 0.47 |
| HT29_13_GR_ChDB_0120 | Crizotinib | 10uM | 6h | C17orf80 | 0.00 | 0.39 |
| HT29_13_GR_ChDB_0120 | Crizotinib | 10uM | 6h | TMEM248 | 0.00 | 0.24 |
| HT29_13_GR_ChDB_0120 | Crizotinib | 10uM | 6h | GTPBP3 | 0.00 | 0.49 |
| HT29_13_GR_ChDB_0120 | Crizotinib | 10uM | 6h | RBM12B | 0.00 | 0.49 |
| HT29_13_GR_ChDB_0120 | Crizotinib | 10uM | 6h | LEPROT | 0.00 | 0.25 |
| HT29_13_GR_ChDB_0120 | Crizotinib | 10uM | 6h | C22orf29 | 0.00 | 0.47 |
| HT29_13_GR_ChDB_0120 | Crizotinib | 10uM | 6h | MANEA | 0.00 | 0.29 |
| HT29_13_GR_ChDB_0120 | Crizotinib | 10uM | 6h | ZFYVE16 | 0.00 | 0.43 |
| HT29_13_GR_ChDB_0120 | Crizotinib | 10uM | 6h | PROM1 | 0.00 | 0.23 |
| HT29_13_GR_ChDB_0120 | Crizotinib | 10uM | 6h | ZNF106 | 0.00 | 0.43 |
| HT29_13_GR_ChDB_0120 | Crizotinib | 10uM | 6h | WRAP73 | 0.00 | 0.45 |
| HT29_13_GR_ChDB_0120 | Crizotinib | 10uM | 6h | KDSR | 0.00 | 0.38 |
| HT29_13_GR_ChDB_0120 | Crizotinib | 10uM | 6h | APPL1 | 0.00 | 0.46 |
| HT29_13_GR_ChDB_0120 | Crizotinib | 10uM | 6h | ZNF16 | 0.00 | 2.09 |
| HT29_13_GR_ChDB_0120 | Crizotinib | 10uM | 6h | FOXA1 | 0.00 | 2.44 |
| HT29_13_GR_ChDB_0120 | Crizotinib | 10uM | 6h | ETS1 | 0.00 | 3.17 |
| HT29_13_GR_ChDB_0120 | Crizotinib | 10uM | 6h | ZNF184 | 0.00 | 2.06 |
| HT29_13_GR_ChDB_0120 | Crizotinib | 10uM | 6h | NDUFA10 | 0.00 | 4.01 |
| HT29_13_GR_ChDB_0120 | Crizotinib | 10uM | 6h | VSIG10 | 0.00 | 0.50 |
| HT29_13_GR_ChDB_0120 | Crizotinib | 10uM | 6h | NUPL1 | 0.00 | 0.20 |
| HT29_13_GR_ChDB_0120 | Crizotinib | 10uM | 6h | RRP1 | 0.00 | 0.43 |
| HT29_13_GR_ChDB_0120 | Crizotinib | 10uM | 6h | VANGL1 | 0.00 | 0.46 |
| HT29_13_GR_ChDB_0120 | Crizotinib | 10uM | 6h | WDR26 | 0.00 | 0.45 |
| HT29_13_GR_ChDB_0120 | Crizotinib | 10uM | 6h | SH2D4A | 0.00 | 0.49 |
| HT29_13_GR_ChDB_0120 | Crizotinib | 10uM | 6h | SPATA2L | 0.00 | 0.47 |
| HT29_13_GR_ChDB_0120 | Crizotinib | 10uM | 6h | BICD1 | 0.00 | 0.27 |
| HT29_13_GR_ChDB_0120 | Crizotinib | 10uM | 6h | DENND5B | 0.00 | 0.35 |
| HT29_13_GR_ChDB_0120 | Crizotinib | 10uM | 6h | SSFA2 | 0.00 | 3.62 |
| HT29_13_GR_ChDB_0120 | Crizotinib | 10uM | 6h | MOSPD1 | 0.00 | 2.23 |
| HT29_13_GR_ChDB_0120 | Crizotinib | 10uM | 6h | SMIM7 | 0.00 | 2.80 |
| HT29_13_GR_ChDB_0120 | Crizotinib | 10uM | 6h | ZNF7 | 0.00 | 2.30 |
| HT29_13_GR_ChDB_0120 | Crizotinib | 10uM | 6h | NECAP2 | 0.00 | 2.33 |
| HT29_13_GR_ChDB_0120 | Crizotinib | 10uM | 6h | MTPAP | 0.00 | 2.29 |
| HT29_13_GR_ChDB_0120 | Crizotinib | 10uM | 6h | WBP5 | 0.00 | 2.00 |
| HT29_13_GR_ChDB_0120 | Crizotinib | 10uM | 6h | PHC2 | 0.00 | 2.01 |
| HT29_13_GR_ChDB_0120 | Crizotinib | 10uM | 6h | LIN37 | 0.00 | 3.01 |
| HT29_13_GR_ChDB_0120 | Crizotinib | 10uM | 6h | MT1F | 0.00 | 2.37 |
| HT29_13_GR_ChDB_0120 | Crizotinib | 10uM | 6h | STK4 | 0.00 | 0.31 |
| HT29_13_GR_ChDB_0120 | Crizotinib | 10uM | 6h | MPZL2 | 0.00 | 0.24 |
| HT29_13_GR_ChDB_0120 | Crizotinib | 10uM | 6h | ARHGEF26 | 0.00 | 0.37 |
| HT29_13_GR_ChDB_0120 | Crizotinib | 10uM | 6h | ROBO1 | 0.00 | 0.46 |
| HT29_13_GR_ChDB_0120 | Crizotinib | 10uM | 6h | PTPRB | 0.00 | 0.38 |
| HT29_13_GR_ChDB_0120 | Crizotinib | 10uM | 6h | FERMT1 | 0.00 | 0.45 |
| HT29_13_GR_ChDB_0120 | Crizotinib | 10uM | 6h | HOOK1 | 0.00 | 0.38 |
| HT29_13_GR_ChDB_0120 | Crizotinib | 10uM | 6h | ENTPD5 | 0.00 | 0.39 |
| HT29_13_GR_ChDB_0120 | Crizotinib | 10uM | 6h | REPS1 | 0.00 | 0.46 |
| HT29_13_GR_ChDB_0120 | Crizotinib | 10uM | 6h | DTWD1 | 0.00 | 0.26 |
| HT29_13_GR_ChDB_0120 | Crizotinib | 10uM | 6h | DPM2 | 0.00 | 0.29 |
| HT29_13_GR_ChDB_0120 | Crizotinib | 10uM | 6h | DEF8 | 0.00 | 0.46 |
| HT29_13_GR_ChDB_0120 | Crizotinib | 10uM | 6h | ADNP | 0.00 | 0.47 |
| HT29_13_GR_ChDB_0120 | Crizotinib | 10uM | 6h | LIMD1 | 0.00 | 0.20 |
| HT29_13_GR_ChDB_0120 | Crizotinib | 10uM | 6h | SACS | 0.00 | 0.14 |
| HT29_13_GR_ChDB_0120 | Crizotinib | 10uM | 6h | SIPA1L3 | 0.00 | 0.28 |
| HT29_13_GR_ChDB_0120 | Crizotinib | 10uM | 6h | FAM208B | 0.00 | 0.33 |
| HT29_13_GR_ChDB_0120 | Crizotinib | 10uM | 6h | TXLNG | 0.00 | 0.32 |
| HT29_13_GR_ChDB_0120 | Crizotinib | 10uM | 6h | MBTPS2 | 0.00 | 0.19 |
| HT29_13_GR_ChDB_0120 | Crizotinib | 10uM | 6h | IMPACT | 0.00 | 0.50 |
| HT29_13_GR_ChDB_0120 | Crizotinib | 10uM | 6h | FAN1 | 0.00 | 0.38 |
| HT29_13_GR_ChDB_0120 | Crizotinib | 10uM | 6h | DDX31 | 0.00 | 0.45 |
| HT29_13_GR_ChDB_0120 | Crizotinib | 10uM | 6h | SLC25A15 | 0.00 | 0.35 |
| HT29_13_GR_ChDB_0120 | Crizotinib | 10uM | 6h | TRMT1L | 0.00 | 0.39 |
| HT29_13_GR_ChDB_0120 | Crizotinib | 10uM | 6h | EIF4G3 | 0.00 | 0.48 |
| HT29_13_GR_ChDB_0120 | Crizotinib | 10uM | 6h | REV3L | 0.00 | 0.46 |
| HT29_13_GR_ChDB_0120 | Crizotinib | 10uM | 6h | SOGA2 | 0.00 | 0.25 |
| HT29_13_GR_ChDB_0120 | Crizotinib | 10uM | 6h | PVRL1 | 0.00 | 0.44 |
| HT29_13_GR_ChDB_0120 | Crizotinib | 10uM | 6h | UBE2C | 0.00 | 2.04 |
| HT29_13_GR_ChDB_0120 | Crizotinib | 10uM | 6h | GNE | 0.00 | 3.34 |
| HT29_13_GR_ChDB_0120 | Crizotinib | 10uM | 6h | DCUN1D1 | 0.00 | 2.39 |
| HT29_13_GR_ChDB_0120 | Crizotinib | 10uM | 6h | PCF11 | 0.00 | 3.07 |
| HT29_13_GR_ChDB_0120 | Crizotinib | 10uM | 6h | C17orf75 | 0.00 | 0.30 |
| HT29_13_GR_ChDB_0120 | Crizotinib | 10uM | 6h | CEP164 | 0.00 | 0.48 |
| HT29_13_GR_ChDB_0120 | Crizotinib | 10uM | 6h | AGAP1 | 0.00 | 0.46 |
| HT29_13_GR_ChDB_0120 | Crizotinib | 10uM | 6h | HERC4 | 0.00 | 0.43 |
| HT29_13_GR_ChDB_0120 | Crizotinib | 10uM | 6h | PRKCQ | 0.00 | 0.45 |
| HT29_13_GR_ChDB_0120 | Crizotinib | 10uM | 6h | TPST2 | 0.00 | 0.42 |
| HT29_13_GR_ChDB_0120 | Crizotinib | 10uM | 6h | GSTCD | 0.00 | 0.42 |
| HT29_13_GR_ChDB_0120 | Crizotinib | 10uM | 6h | PMEPA1 | 0.00 | 0.25 |
| HT29_13_GR_ChDB_0120 | Crizotinib | 10uM | 6h | PCOLCE2 | 0.00 | 0.43 |
| HT29_13_GR_ChDB_0120 | Crizotinib | 10uM | 6h | KPNA4 | 0.00 | 0.27 |
| HT29_13_GR_ChDB_0120 | Crizotinib | 10uM | 6h | ZXDC | 0.00 | 0.47 |
| HT29_13_GR_ChDB_0120 | Crizotinib | 10uM | 6h | PLCXD1 | 0.00 | 0.40 |
| HT29_13_GR_ChDB_0120 | Crizotinib | 10uM | 6h | MON1B | 0.00 | 0.34 |
| HT29_13_GR_ChDB_0120 | Crizotinib | 10uM | 6h | YEATS2 | 0.00 | 0.37 |
| HT29_13_GR_ChDB_0120 | Crizotinib | 10uM | 6h | FAM169A | 0.00 | 0.37 |
| HT29_13_GR_ChDB_0120 | Crizotinib | 10uM | 6h | TNS4 | 0.00 | 0.37 |
| HT29_13_GR_ChDB_0120 | Crizotinib | 10uM | 6h | FNBP1L | 0.00 | 2.53 |
| HT29_13_GR_ChDB_0120 | Crizotinib | 10uM | 6h | MED17 | 0.00 | 2.71 |
| HT29_13_GR_ChDB_0120 | Crizotinib | 10uM | 6h | NUPR1 | 0.00 | 14.44 |
| HT29_13_GR_ChDB_0120 | Crizotinib | 10uM | 6h | CUL1 | 0.00 | 2.35 |
| HT29_13_GR_ChDB_0120 | Crizotinib | 10uM | 6h | ATG14 | 0.00 | 2.45 |
| HT29_13_GR_ChDB_0120 | Crizotinib | 10uM | 6h | LTB | 0.00 | 4.58 |
| HT29_13_GR_ChDB_0120 | Crizotinib | 10uM | 6h | ZNF140 | 0.00 | 2.22 |
| HT29_13_GR_ChDB_0120 | Crizotinib | 10uM | 6h | GLI2 | 0.00 | 0.46 |
| HT29_13_GR_ChDB_0120 | Crizotinib | 10uM | 6h | TMEM187 | 0.00 | 0.47 |
| HT29_13_GR_ChDB_0120 | Crizotinib | 10uM | 6h | RABL3 | 0.00 | 0.49 |
| HT29_13_GR_ChDB_0120 | Crizotinib | 10uM | 6h | DIXDC1 | 0.00 | 0.44 |
| HT29_13_GR_ChDB_0120 | Crizotinib | 10uM | 6h | KCTD15 | 0.00 | 0.34 |
| HT29_13_GR_ChDB_0120 | Crizotinib | 10uM | 6h | SOWAHC | 0.00 | 0.46 |
| HT29_13_GR_ChDB_0120 | Crizotinib | 10uM | 6h | CLSPN | 0.00 | 0.45 |
| HT29_13_GR_ChDB_0120 | Crizotinib | 10uM | 6h | KIF13A | 0.00 | 0.47 |
| HT29_13_GR_ChDB_0120 | Crizotinib | 10uM | 6h | CUX1 | 0.00 | 0.43 |
| HT29_13_GR_ChDB_0120 | Crizotinib | 10uM | 6h | SSH1 | 0.00 | 0.46 |
| HT29_13_GR_ChDB_0120 | Crizotinib | 10uM | 6h | EHD4 | 0.00 | 0.39 |
| HT29_13_GR_ChDB_0120 | Crizotinib | 10uM | 6h | RFXAP | 0.00 | 0.50 |
| HT29_13_GR_ChDB_0120 | Crizotinib | 10uM | 6h | TTC17 | 0.00 | 0.39 |
| HT29_13_GR_ChDB_0120 | Crizotinib | 10uM | 6h | CLASP1 | 0.00 | 0.49 |
| HT29_13_GR_ChDB_0120 | Crizotinib | 10uM | 6h | ADCY7 | 0.00 | 0.39 |
| HT29_13_GR_ChDB_0120 | Crizotinib | 10uM | 6h | TTF2 | 0.00 | 0.24 |
| HT29_13_GR_ChDB_0120 | Crizotinib | 10uM | 6h | CCDC85C | 0.00 | 0.30 |
| HT29_13_GR_ChDB_0120 | Crizotinib | 10uM | 6h | AHCTF1 | 0.00 | 0.45 |
| HT29_13_GR_ChDB_0120 | Crizotinib | 10uM | 6h | DDX46 | 0.00 | 0.32 |
| HT29_13_GR_ChDB_0120 | Crizotinib | 10uM | 6h | PUS7L | 0.00 | 0.25 |
| HT29_13_GR_ChDB_0120 | Crizotinib | 10uM | 6h | PFKM | 0.00 | 0.48 |
| HT29_13_GR_ChDB_0120 | Crizotinib | 10uM | 6h | TTC4 | 0.00 | 0.45 |
| HT29_13_GR_ChDB_0120 | Crizotinib | 10uM | 6h | DTX4 | 0.00 | 0.33 |
| HT29_13_GR_ChDB_0120 | Crizotinib | 10uM | 6h | MT1H | 0.00 | 2.97 |
| HT29_13_GR_ChDB_0120 | Crizotinib | 10uM | 6h | FGD6 | 0.00 | 2.42 |
| HT29_13_GR_ChDB_0120 | Crizotinib | 10uM | 6h | RNF44 | 0.00 | 2.01 |
| HT29_13_GR_ChDB_0120 | Crizotinib | 10uM | 6h | GPBP1L1 | 0.00 | 3.20 |
| HT29_13_GR_ChDB_0120 | Crizotinib | 10uM | 6h | C11orf57 | 0.00 | 2.44 |
| HT29_13_GR_ChDB_0120 | Crizotinib | 10uM | 6h | TBL1XR1 | 0.00 | 2.59 |
| HT29_13_GR_ChDB_0120 | Crizotinib | 10uM | 6h | ZNF322 | 0.00 | 3.58 |
| HT29_13_GR_ChDB_0120 | Crizotinib | 10uM | 6h | C4orf19 | 0.00 | 0.38 |
| HT29_13_GR_ChDB_0120 | Crizotinib | 10uM | 6h | SORT1 | 0.00 | 0.49 |
| HT29_13_GR_ChDB_0120 | Crizotinib | 10uM | 6h | RNF141 | 0.00 | 0.49 |
| HT29_13_GR_ChDB_0120 | Crizotinib | 10uM | 6h | SLC12A8 | 0.00 | 0.42 |
| HT29_13_GR_ChDB_0120 | Crizotinib | 10uM | 6h | PHF20L1 | 0.00 | 0.32 |
| HT29_13_GR_ChDB_0120 | Crizotinib | 10uM | 6h | WNT11 | 0.00 | 0.23 |
| HT29_13_GR_ChDB_0120 | Crizotinib | 10uM | 6h | RNFT1 | 0.00 | 0.28 |
| HT29_13_GR_ChDB_0120 | Crizotinib | 10uM | 6h | EOGT | 0.00 | 0.43 |
| HT29_13_GR_ChDB_0120 | Crizotinib | 10uM | 6h | HKDC1 | 0.00 | 0.49 |
| HT29_13_GR_ChDB_0120 | Crizotinib | 10uM | 6h | GUF1 | 0.00 | 0.49 |
| HT29_13_GR_ChDB_0120 | Crizotinib | 10uM | 6h | NBPF1 | 0.00 | 0.40 |
| HT29_13_GR_ChDB_0120 | Crizotinib | 10uM | 6h | POLR1B | 0.00 | 0.46 |
| HT29_13_GR_ChDB_0120 | Crizotinib | 10uM | 6h | ZBTB44 | 0.00 | 0.41 |
| HT29_13_GR_ChDB_0120 | Crizotinib | 10uM | 6h | SLC35C2 | 0.00 | 0.26 |
| HT29_13_GR_ChDB_0120 | Crizotinib | 10uM | 6h | FAM35A | 0.00 | 0.42 |
| HT29_13_GR_ChDB_0120 | Crizotinib | 10uM | 6h | SPAG9 | 0.00 | 0.44 |
| HT29_13_GR_ChDB_0120 | Crizotinib | 10uM | 6h | INPP5F | 0.00 | 0.34 |
| HT29_13_GR_ChDB_0120 | Crizotinib | 10uM | 6h | ENAH | 0.00 | 22.25 |
| HT29_13_GR_ChDB_0120 | Crizotinib | 10uM | 6h | RBM22 | 0.00 | 2.28 |
| HT29_13_GR_ChDB_0120 | Crizotinib | 10uM | 6h | DKK1 | 0.00 | 0.27 |
| HT29_13_GR_ChDB_0120 | Crizotinib | 10uM | 6h | SAR1B | 0.00 | 0.32 |
| HT29_13_GR_ChDB_0120 | Crizotinib | 10uM | 6h | FBXO41 | 0.00 | 0.49 |
| HT29_13_GR_ChDB_0120 | Crizotinib | 10uM | 6h | EMC2 | 0.00 | 0.37 |
| HT29_13_GR_ChDB_0120 | Crizotinib | 10uM | 6h | PTPN13 | 0.00 | 0.49 |
| HT29_13_GR_ChDB_0120 | Crizotinib | 10uM | 6h | ZBTB18 | 0.00 | 0.44 |
| HT29_13_GR_ChDB_0120 | Crizotinib | 10uM | 6h | GTF2H2B | 0.00 | 0.14 |
| HT29_13_GR_ChDB_0120 | Crizotinib | 10uM | 6h | TMEM168 | 0.00 | 0.23 |
| HT29_13_GR_ChDB_0120 | Crizotinib | 10uM | 6h | SHB | 0.00 | 2.15 |
| HT29_13_GR_ChDB_0120 | Crizotinib | 10uM | 6h | LACTB2 | 0.00 | 0.34 |
| HT29_13_GR_ChDB_0120 | Crizotinib | 10uM | 6h | STEAP4 | 0.00 | 0.27 |
| HT29_13_GR_ChDB_0120 | Crizotinib | 10uM | 6h | ICK | 0.00 | 0.42 |
| HT29_13_GR_ChDB_0120 | Crizotinib | 10uM | 6h | ARL15 | 0.00 | 0.26 |
| HT29_13_GR_ChDB_0120 | Crizotinib | 10uM | 6h | NDUFAF7 | 0.00 | 0.36 |
| HT29_13_GR_ChDB_0120 | Crizotinib | 10uM | 6h | MLLT4 | 0.00 | 0.44 |
| HT29_13_GR_ChDB_0120 | Crizotinib | 10uM | 6h | EPN3 | 0.00 | 0.38 |
| HT29_13_GR_ChDB_0120 | Crizotinib | 10uM | 6h | FZD3 | 0.00 | 0.32 |
| HT29_13_GR_ChDB_0120 | Crizotinib | 10uM | 6h | DOCK5 | 0.00 | 0.31 |
| HT29_13_GR_ChDB_0120 | Crizotinib | 10uM | 6h | SEMA3A | 0.00 | 0.30 |
| HT29_13_GR_ChDB_0120 | Crizotinib | 10uM | 6h | THNSL1 | 0.00 | 0.24 |
| HT29_13_GR_ChDB_0120 | Crizotinib | 10uM | 6h | PTPRJ | 0.00 | 0.38 |
| HT29_13_GR_ChDB_0120 | Crizotinib | 10uM | 6h | EXOC1 | 0.00 | 0.47 |
| HT29_13_GR_ChDB_0120 | Crizotinib | 10uM | 6h | C17orf85 | 0.00 | 0.35 |
| HT29_13_GR_ChDB_0120 | Crizotinib | 10uM | 6h | SAP30BP | 0.00 | 4.16 |
| HT29_13_GR_ChDB_0120 | Crizotinib | 10uM | 6h | GNG12 | 0.00 | 2.04 |
| HT29_13_GR_ChDB_0120 | Crizotinib | 10uM | 6h | TTC33 | 0.00 | 2.18 |
| HT29_13_GR_ChDB_0120 | Crizotinib | 10uM | 6h | MARCH5 | 0.00 | 2.24 |
| HT29_13_GR_ChDB_0120 | Crizotinib | 10uM | 6h | EFNA2 | 0.00 | 0.43 |
| HT29_13_GR_ChDB_0120 | Crizotinib | 10uM | 6h | PPP6R3 | 0.00 | 0.36 |
| HT29_13_GR_ChDB_0120 | Crizotinib | 10uM | 6h | TNS3 | 0.00 | 0.19 |
| HT29_13_GR_ChDB_0120 | Crizotinib | 10uM | 6h | BBX | 0.00 | 0.33 |
| HT29_13_GR_ChDB_0120 | Crizotinib | 10uM | 6h | DNAJA4 | 0.00 | 0.22 |
| HT29_13_GR_ChDB_0120 | Crizotinib | 10uM | 6h | GOLGA3 | 0.00 | 0.41 |
| HT29_13_GR_ChDB_0120 | Crizotinib | 10uM | 6h | CHML | 0.00 | 0.26 |
| HT29_13_GR_ChDB_0120 | Crizotinib | 10uM | 6h | NSUN6 | 0.00 | 0.42 |
| HT29_13_GR_ChDB_0120 | Crizotinib | 10uM | 6h | RALGPS2 | 0.00 | 0.30 |
| HT29_13_GR_ChDB_0120 | Crizotinib | 10uM | 6h | SMC6 | 0.00 | 0.40 |
| HT29_13_GR_ChDB_0120 | Crizotinib | 10uM | 6h | ARMC9 | 0.00 | 0.50 |
| HT29_13_GR_ChDB_0120 | Crizotinib | 10uM | 6h | TMEM165 | 0.00 | 0.39 |
| HT29_13_GR_ChDB_0120 | Crizotinib | 10uM | 6h | TAF4B | 0.00 | 0.37 |
| HT29_13_GR_ChDB_0120 | Crizotinib | 10uM | 6h | RCAN3 | 0.00 | 0.42 |
| HT29_13_GR_ChDB_0120 | Crizotinib | 10uM | 6h | PCYOX1 | 0.00 | 0.48 |
| HT29_13_GR_ChDB_0120 | Crizotinib | 10uM | 6h | YTHDF3 | 0.00 | 2.50 |
| HT29_13_GR_ChDB_0120 | Crizotinib | 10uM | 6h | CBY1 | 0.00 | 0.43 |
| HT29_13_GR_ChDB_0120 | Crizotinib | 10uM | 6h | VIPR1 | 0.00 | 0.44 |
| HT29_13_GR_ChDB_0120 | Crizotinib | 10uM | 6h | SYTL2 | 0.00 | 0.36 |
| HT29_13_GR_ChDB_0120 | Crizotinib | 10uM | 6h | GFM1 | 0.00 | 0.34 |
| HT29_13_GR_ChDB_0120 | Crizotinib | 10uM | 6h | SGPP1 | 0.00 | 0.40 |
| HT29_13_GR_ChDB_0120 | Crizotinib | 10uM | 6h | LOC389906 | 0.00 | 0.48 |
| HT29_13_GR_ChDB_0120 | Crizotinib | 10uM | 6h | KLHL2 | 0.00 | 0.49 |
| HT29_13_GR_ChDB_0120 | Crizotinib | 10uM | 6h | KANSL3 | 0.00 | 0.40 |
| HT29_13_GR_ChDB_0120 | Crizotinib | 10uM | 6h | TSPAN14 | 0.00 | 0.44 |
| HT29_13_GR_ChDB_0120 | Crizotinib | 10uM | 6h | AVPI1 | 0.00 | 0.40 |
| HT29_13_GR_ChDB_0120 | Crizotinib | 10uM | 6h | KIF3A | 0.00 | 0.40 |
| HT29_13_GR_ChDB_0120 | Crizotinib | 10uM | 6h | ZMAT3 | 0.00 | 0.26 |
| HT29_13_GR_ChDB_0120 | Crizotinib | 10uM | 6h | KMT2D | 0.00 | 0.27 |
| HT29_13_GR_ChDB_0120 | Crizotinib | 10uM | 6h | SLC25A30 | 0.00 | 0.41 |
| HT29_13_GR_ChDB_0120 | Crizotinib | 10uM | 6h | C1GALT1 | 0.00 | 0.36 |
| HT29_13_GR_ChDB_0120 | Crizotinib | 10uM | 6h | EPPK1 | 0.00 | 0.47 |
| HT29_13_GR_ChDB_0120 | Crizotinib | 10uM | 6h | ST3GAL1 | 0.00 | 0.43 |
| HT29_13_GR_ChDB_0120 | Crizotinib | 10uM | 6h | ZNF37A | 0.00 | 0.24 |
| HT29_13_GR_ChDB_0120 | Crizotinib | 10uM | 6h | PTER | 0.00 | 0.33 |
| HT29_13_GR_ChDB_0120 | Crizotinib | 10uM | 6h | MAVS | 0.00 | 0.46 |
| HT29_13_GR_ChDB_0120 | Crizotinib | 10uM | 6h | ELP3 | 0.00 | 0.50 |
| HT29_13_GR_ChDB_0120 | Crizotinib | 10uM | 6h | RBM17 | 0.00 | 0.24 |
| HT29_13_GR_ChDB_0120 | Crizotinib | 10uM | 6h | POLR1D | 0.00 | 2.34 |
| HT29_13_GR_ChDB_0120 | Crizotinib | 10uM | 6h | PIGG | 0.00 | 0.47 |
| HT29_13_GR_ChDB_0120 | Crizotinib | 10uM | 6h | LRRC37A2 | 0.00 | 0.48 |
| HT29_13_GR_ChDB_0120 | Crizotinib | 10uM | 6h | KIAA1199 | 0.00 | 0.16 |
| HT29_13_GR_ChDB_0120 | Crizotinib | 10uM | 6h | LOC399491 | 0.00 | 0.30 |
| HT29_13_GR_ChDB_0120 | Crizotinib | 10uM | 6h | BCL11B | 0.00 | 0.36 |
| HT29_13_GR_ChDB_0120 | Crizotinib | 10uM | 6h | SLC25A40 | 0.00 | 0.41 |
| HT29_13_GR_ChDB_0120 | Crizotinib | 10uM | 6h | TOM1L1 | 0.00 | 3.47 |
| HT29_13_GR_ChDB_0120 | Crizotinib | 10uM | 6h | LRIG2 | 0.00 | 2.34 |
| HT29_13_GR_ChDB_0120 | Crizotinib | 10uM | 6h | XPO1 | 0.00 | 2.29 |
| HT29_13_GR_ChDB_0120 | Crizotinib | 10uM | 6h | MUS81 | 0.00 | 0.37 |
| HT29_13_GR_ChDB_0120 | Crizotinib | 10uM | 6h | TRAPPC11 | 0.00 | 0.47 |
| HT29_13_GR_ChDB_0120 | Crizotinib | 10uM | 6h | CCDC132 | 0.00 | 0.32 |
| HT29_13_GR_ChDB_0120 | Crizotinib | 10uM | 6h | KLHL29 | 0.00 | 0.29 |
| HT29_13_GR_ChDB_0120 | Crizotinib | 10uM | 6h | DCBLD2 | 0.00 | 0.35 |
| HT29_13_GR_ChDB_0120 | Crizotinib | 10uM | 6h | PRR14L | 0.00 | 0.24 |
| HT29_13_GR_ChDB_0120 | Crizotinib | 10uM | 6h | NFIX | 0.00 | 0.48 |
| HT29_13_GR_ChDB_0120 | Crizotinib | 10uM | 6h | SOCS7 | 0.00 | 0.31 |
| HT29_13_GR_ChDB_0120 | Crizotinib | 10uM | 6h | RAB11FIP1 | 0.00 | 0.45 |
| HT29_13_GR_ChDB_0120 | Crizotinib | 10uM | 6h | ANKS1A | 0.00 | 0.43 |
| HT29_13_GR_ChDB_0120 | Crizotinib | 10uM | 6h | PWP2 | 0.00 | 0.46 |
| HT29_13_GR_ChDB_0120 | Crizotinib | 10uM | 6h | PHF14 | 0.00 | 0.31 |
| HT29_13_GR_ChDB_0120 | Crizotinib | 10uM | 6h | STAMBPL1 | 0.00 | 0.39 |
| HT29_13_GR_ChDB_0120 | Crizotinib | 10uM | 6h | ANKFY1 | 0.00 | 0.47 |
| HT29_13_GR_ChDB_0120 | Crizotinib | 10uM | 6h | CASC5 | 0.00 | 0.40 |
| HT29_13_GR_ChDB_0120 | Crizotinib | 10uM | 6h | TRIP12 | 0.00 | 0.32 |
| HT29_13_GR_ChDB_0120 | Crizotinib | 10uM | 6h | PAQR3 | 0.00 | 0.21 |
| HT29_13_GR_ChDB_0120 | Crizotinib | 10uM | 6h | NIN | 0.00 | 0.41 |
| HT29_13_GR_ChDB_0120 | Crizotinib | 10uM | 6h | EIF5A2 | 0.00 | 0.29 |
| HT29_13_GR_ChDB_0120 | Crizotinib | 10uM | 6h | RBM4B | 0.00 | 0.43 |
| HT29_13_GR_ChDB_0120 | Crizotinib | 10uM | 6h | TAOK1 | 0.00 | 0.48 |
| HT29_13_GR_ChDB_0120 | Crizotinib | 10uM | 6h | LONRF3 | 0.00 | 0.21 |
| HT29_13_GR_ChDB_0120 | Crizotinib | 10uM | 6h | NABP1 | 0.00 | 0.40 |
| HT29_13_GR_ChDB_0120 | Crizotinib | 10uM | 6h | NAGK | 0.00 | 0.47 |
| HT29_13_GR_ChDB_0120 | Crizotinib | 10uM | 6h | TRIM5 | 0.00 | 0.45 |
| HT29_13_GR_ChDB_0120 | Crizotinib | 10uM | 6h | MKLN1 | 0.00 | 0.46 |
| HT29_13_GR_ChDB_0120 | Crizotinib | 10uM | 6h | STX17 | 0.00 | 0.31 |
| HT29_13_GR_ChDB_0120 | Crizotinib | 10uM | 6h | CKAP2 | 0.00 | 2.27 |
| HT29_13_GR_ChDB_0120 | Crizotinib | 10uM | 6h | DOLK | 0.00 | 0.49 |
| HT29_13_GR_ChDB_0120 | Crizotinib | 10uM | 6h | CYP2R1 | 0.00 | 0.44 |
| HT29_13_GR_ChDB_0120 | Crizotinib | 10uM | 6h | TTPAL | 0.00 | 0.31 |
| HT29_13_GR_ChDB_0120 | Crizotinib | 10uM | 6h | FBXO22 | 0.00 | 0.43 |
| HT29_13_GR_ChDB_0120 | Crizotinib | 10uM | 6h | MED31 | 0.00 | 0.37 |
| HT29_13_GR_ChDB_0120 | Crizotinib | 10uM | 6h | EXTL2 | 0.00 | 0.38 |
| HT29_13_GR_ChDB_0120 | Crizotinib | 10uM | 6h | TWISTNB | 0.00 | 0.47 |
| HT29_13_GR_ChDB_0120 | Crizotinib | 10uM | 6h | FAM135A | 0.00 | 0.25 |
| HT29_13_GR_ChDB_0120 | Crizotinib | 10uM | 6h | CRYBG3 | 0.00 | 0.30 |
| HT29_13_GR_ChDB_0120 | Crizotinib | 10uM | 6h | SLC35F5 | 0.00 | 0.30 |
| HT29_13_GR_ChDB_0120 | Crizotinib | 10uM | 6h | AGPAT3 | 0.00 | 0.49 |
| HT29_13_GR_ChDB_0120 | Crizotinib | 10uM | 6h | ELP6 | 0.00 | 0.45 |
| HT29_13_GR_ChDB_0120 | Crizotinib | 10uM | 6h | PLEKHA2 | 0.00 | 0.44 |
| HT29_13_GR_ChDB_0120 | Crizotinib | 10uM | 6h | CCDC82 | 0.00 | 0.41 |
| HT29_13_GR_ChDB_0120 | Crizotinib | 10uM | 6h | SPC24 | 0.00 | 0.50 |
| HT29_13_GR_ChDB_0120 | Crizotinib | 10uM | 6h | SLC25A38 | 0.00 | 2.20 |
| HT29_13_GR_ChDB_0120 | Crizotinib | 10uM | 6h | CDC23 | 0.00 | 2.04 |
| HT29_13_GR_ChDB_0120 | Crizotinib | 10uM | 6h | SPICE1 | 0.00 | 0.41 |
| HT29_13_GR_ChDB_0120 | Crizotinib | 10uM | 6h | POU2F1 | 0.00 | 0.42 |
| HT29_13_GR_ChDB_0120 | Crizotinib | 10uM | 6h | PDE12 | 0.00 | 0.35 |
| HT29_13_GR_ChDB_0120 | Crizotinib | 10uM | 6h | ACSL4 | 0.00 | 0.44 |
| HT29_14_GR_ChDB_0154 | BEZ235 | 1uM | 6h | KLF6 | 1.00 | 5.34 |
| HT29_14_GR_ChDB_0157 | Afatinib | 10uM | 6h | VEGFA | 1.00 | 2.73 |
| HT29_14_GR_ChDB_0157 | Afatinib | 10uM | 6h | HMOX1 | 0.17 | 11.31 |
| HT29_14_GR_ChDB_0157 | Afatinib | 10uM | 6h | SLC22A18 | 0.12 | 2.44 |
| HT29_14_GR_ChDB_0157 | Afatinib | 10uM | 6h | GCLC | 0.12 | 2.37 |
| HT29_14_GR_ChDB_0157 | Afatinib | 10uM | 6h | MAPK8 | 0.09 | 0.48 |
| HT29_14_GR_ChDB_0157 | Afatinib | 10uM | 6h | KLF6 | 0.07 | 2.19 |
| HT29_14_GR_ChDB_0157 | Afatinib | 10uM | 6h | ARL4C | 0.06 | 2.69 |
| HT29_14_GR_ChDB_0157 | Afatinib | 10uM | 6h | SQSTM1 | 0.04 | 2.69 |
| HT29_14_GR_ChDB_0157 | Afatinib | 10uM | 6h | LIPH | 0.04 | 2.12 |
| HT29_14_GR_ChDB_0157 | Afatinib | 10uM | 6h | RRAGC | 0.04 | 2.07 |
| HT29_14_GR_ChDB_0157 | Afatinib | 10uM | 6h | DDIT3 | 0.04 | 9.09 |
| HT29_14_GR_ChDB_0157 | Afatinib | 10uM | 6h | KLF4 | 0.03 | 2.07 |
| HT29_14_GR_ChDB_0157 | Afatinib | 10uM | 6h | ID2 | 0.03 | 0.42 |
| HT29_14_GR_ChDB_0157 | Afatinib | 10uM | 6h | TRIB3 | 0.03 | 3.66 |
| HT29_14_GR_ChDB_0157 | Afatinib | 10uM | 6h | ETV5 | 0.03 | 2.84 |
| HT29_14_GR_ChDB_0157 | Afatinib | 10uM | 6h | ARHGEF2 | 0.03 | 2.13 |
| HT29_14_GR_ChDB_0157 | Afatinib | 10uM | 6h | EGR1 | 0.02 | 2.39 |
| HT29_14_GR_ChDB_0157 | Afatinib | 10uM | 6h | PLK2 | 0.02 | 0.49 |
| HT29_14_GR_ChDB_0157 | Afatinib | 10uM | 6h | AKR1C3 | 0.02 | 3.05 |
| HT29_14_GR_ChDB_0157 | Afatinib | 10uM | 6h | PLAUR | 0.02 | 2.10 |
| HT29_14_GR_ChDB_0157 | Afatinib | 10uM | 6h | GADD45A | 0.02 | 3.11 |
| HT29_14_GR_ChDB_0157 | Afatinib | 10uM | 6h | AKR1C1 | 0.02 | 6.79 |
| HT29_14_GR_ChDB_0157 | Afatinib | 10uM | 6h | MBNL2 | 0.01 | 2.78 |
| HT29_14_GR_ChDB_0157 | Afatinib | 10uM | 6h | TUBB3 | 0.01 | 0.43 |
| HT29_14_GR_ChDB_0157 | Afatinib | 10uM | 6h | PCK2 | 0.01 | 3.71 |
| HT29_14_GR_ChDB_0157 | Afatinib | 10uM | 6h | BTG1 | 0.01 | 2.41 |
| HT29_14_GR_ChDB_0157 | Afatinib | 10uM | 6h | SLC7A11 | 0.01 | 5.90 |
| HT29_14_GR_ChDB_0157 | Afatinib | 10uM | 6h | NT5E | 0.01 | 2.09 |
| HT29_14_GR_ChDB_0157 | Afatinib | 10uM | 6h | SMAD3 | 0.01 | 2.35 |
| HT29_14_GR_ChDB_0157 | Afatinib | 10uM | 6h | SLC38A2 | 0.01 | 2.11 |
| HT29_14_GR_ChDB_0157 | Afatinib | 10uM | 6h | ABHD2 | 0.01 | 2.12 |
| HT29_14_GR_ChDB_0157 | Afatinib | 10uM | 6h | SARS | 0.01 | 2.99 |
| HT29_14_GR_ChDB_0157 | Afatinib | 10uM | 6h | CAB39 | 0.01 | 2.00 |
| HT29_14_GR_ChDB_0157 | Afatinib | 10uM | 6h | DDIT4 | 0.00 | 13.83 |
| HT29_14_GR_ChDB_0157 | Afatinib | 10uM | 6h | DDX5 | 0.00 | 0.37 |
| HT29_14_GR_ChDB_0157 | Afatinib | 10uM | 6h | TRIM16 | 0.00 | 2.05 |
| HT29_14_GR_ChDB_0157 | Afatinib | 10uM | 6h | CARS | 0.00 | 2.11 |
| HT29_14_GR_ChDB_0157 | Afatinib | 10uM | 6h | SLC25A32 | 0.00 | 0.49 |
| HT29_14_GR_ChDB_0157 | Afatinib | 10uM | 6h | SPDEF | 0.00 | 0.44 |
| HT29_14_GR_ChDB_0157 | Afatinib | 10uM | 6h | SAR1B | 0.00 | 0.43 |
| HT29_14_GR_ChDB_0157 | Afatinib | 10uM | 6h | MXD1 | 0.00 | 2.24 |
| HT29_14_GR_ChDB_0157 | Afatinib | 10uM | 6h | CLIC4 | 0.00 | 2.45 |
| HT29_14_GR_ChDB_0157 | Afatinib | 10uM | 6h | EXOSC4 | 0.00 | 0.49 |
| HT29_14_GR_ChDB_0157 | Afatinib | 10uM | 6h | RGS3 | 0.00 | 2.24 |
| HT29_14_GR_ChDB_0157 | Afatinib | 10uM | 6h | CALCOCO2 | 0.00 | 2.53 |
| HT29_14_GR_ChDB_0157 | Afatinib | 10uM | 6h | GNA12 | 0.00 | 0.48 |
| HT29_14_GR_ChDB_0157 | Afatinib | 10uM | 6h | MAT2A | 0.00 | 0.36 |
| HT29_14_GR_ChDB_0157 | Afatinib | 10uM | 6h | SLC25A16 | 0.00 | 2.03 |
| HT29_14_GR_ChDB_0157 | Afatinib | 10uM | 6h | CTH | 0.00 | 7.02 |
| HT29_14_GR_ChDB_0157 | Afatinib | 10uM | 6h | GRB10 | 0.00 | 2.32 |
| HT29_14_GR_ChDB_0157 | Afatinib | 10uM | 6h | NOP16 | 0.00 | 0.44 |
| HT29_14_GR_ChDB_0157 | Afatinib | 10uM | 6h | BCAN | 0.00 | 2.11 |
| HT29_14_GR_ChDB_0157 | Afatinib | 10uM | 6h | SNTB1 | 0.00 | 2.41 |
| HT29_14_GR_ChDB_0157 | Afatinib | 10uM | 6h | ANKRD11 | 0.00 | 2.59 |
| HT29_14_GR_ChDB_0157 | Afatinib | 10uM | 6h | NINJ1 | 0.00 | 2.07 |
| HT29_14_GR_ChDB_0157 | Afatinib | 10uM | 6h | OPTN | 0.00 | 2.07 |
| HT29_14_GR_ChDB_0157 | Afatinib | 10uM | 6h | GULP1 | 0.00 | 2.94 |
| HT29_14_GR_ChDB_0157 | Afatinib | 10uM | 6h | PSMD11 | 0.00 | 0.47 |
| HT29_14_GR_ChDB_0157 | Afatinib | 10uM | 6h | FARSA | 0.00 | 0.47 |
| HT29_14_GR_ChDB_0157 | Afatinib | 10uM | 6h | HNRNPA0 | 0.00 | 0.45 |
| HT29_14_GR_ChDB_0157 | Afatinib | 10uM | 6h | LMF2 | 0.00 | 2.42 |
| HT29_14_GR_ChDB_0157 | Afatinib | 10uM | 6h | DPYSL2 | 0.00 | 2.10 |
| HT29_14_GR_ChDB_0157 | Afatinib | 10uM | 6h | SH2B3 | 0.00 | 2.03 |
| HT29_14_GR_ChDB_0157 | Afatinib | 10uM | 6h | MYH14 | 0.00 | 2.35 |
| HT29_14_GR_ChDB_0157 | Afatinib | 10uM | 6h | GOLGA3 | 0.00 | 2.08 |
| HT29_14_GR_ChDB_0157 | Afatinib | 10uM | 6h | KIAA1551 | 0.00 | 2.16 |
| HT29_14_GR_ChDB_0157 | Afatinib | 10uM | 6h | HSPA13 | 0.00 | 2.05 |
| HT29_14_GR_ChDB_0157 | Afatinib | 10uM | 6h | SLPI | 0.00 | 2.06 |
| HT29_14_GR_ChDB_0157 | Afatinib | 10uM | 6h | LMO7 | 0.00 | 2.27 |
| HT29_14_GR_ChDB_0157 | Afatinib | 10uM | 6h | FHL2 | 0.00 | 2.35 |
| HT29_14_GR_ChDB_0157 | Afatinib | 10uM | 6h | APOBEC3B | 0.00 | 2.37 |
| HT29_14_GR_ChDB_0157 | Afatinib | 10uM | 6h | CLDN1 | 0.00 | 2.02 |
| HT29_14_GR_ChDB_0157 | Afatinib | 10uM | 6h | GDF15 | 0.00 | 3.31 |
| HT29_14_GR_ChDB_0157 | Afatinib | 10uM | 6h | FASTKD2 | 0.00 | 0.43 |
| HT29_14_GR_ChDB_0157 | Afatinib | 10uM | 6h | JMJD4 | 0.00 | 0.49 |
| HT29_14_GR_ChDB_0157 | Afatinib | 10uM | 6h | LEPROT | 0.00 | 0.48 |
| HT29_14_GR_ChDB_0157 | Afatinib | 10uM | 6h | TSR1 | 0.00 | 0.50 |
| HT29_14_GR_ChDB_0157 | Afatinib | 10uM | 6h | BCL2L11 | 0.00 | 0.44 |
| HT29_14_GR_ChDB_0157 | Afatinib | 10uM | 6h | NFATC2IP | 0.00 | 0.42 |
| HT29_14_GR_ChDB_0157 | Afatinib | 10uM | 6h | PMAIP1 | 0.00 | 2.48 |
| HT29_14_GR_ChDB_0157 | Afatinib | 10uM | 6h | FAM178A | 0.00 | 2.02 |
| HT29_14_GR_ChDB_0157 | Afatinib | 10uM | 6h | RNF19B | 0.00 | 2.02 |
| HT29_14_GR_ChDB_0157 | Afatinib | 10uM | 6h | PMM1 | 0.00 | 2.08 |
| HT29_14_GR_ChDB_0157 | Afatinib | 10uM | 6h | ULBP2 | 0.00 | 2.59 |
| HT29_14_GR_ChDB_0157 | Afatinib | 10uM | 6h | MGEA5 | 0.00 | 2.29 |
| HT29_14_GR_ChDB_0157 | Afatinib | 10uM | 6h | BRE | 0.00 | 2.36 |
| HT29_14_GR_ChDB_0157 | Afatinib | 10uM | 6h | HIST3H2A | 0.00 | 2.10 |
| HT29_14_GR_ChDB_0157 | Afatinib | 10uM | 6h | CCNG2 | 0.00 | 3.40 |
| HT29_14_GR_ChDB_0157 | Afatinib | 10uM | 6h | HK2 | 0.00 | 0.47 |
| HT29_14_GR_ChDB_0157 | Afatinib | 10uM | 6h | SEC62 | 0.00 | 0.50 |
| HT29_14_GR_ChDB_0157 | Afatinib | 10uM | 6h | USP53 | 0.00 | 2.01 |
| HT29_14_GR_ChDB_0157 | Afatinib | 10uM | 6h | AMIGO2 | 0.00 | 2.17 |
| HT29_14_GR_ChDB_0157 | Afatinib | 10uM | 6h | DNAJB4 | 0.00 | 2.34 |
| HT29_14_GR_ChDB_0157 | Afatinib | 10uM | 6h | AARS | 0.00 | 2.23 |
| HT29_14_GR_ChDB_0157 | Afatinib | 10uM | 6h | C9orf16 | 0.00 | 2.15 |
| HT29_14_GR_ChDB_0157 | Afatinib | 10uM | 6h | FTSJ3 | 0.00 | 0.46 |
| HT29_14_GR_ChDB_0157 | Afatinib | 10uM | 6h | URB2 | 0.00 | 0.46 |
| HT29_14_GR_ChDB_0157 | Afatinib | 10uM | 6h | HS1BP3 | 0.00 | 2.20 |
| HT29_14_GR_ChDB_0157 | Afatinib | 10uM | 6h | CEBPG | 0.00 | 2.21 |
| HT29_14_GR_ChDB_0157 | Afatinib | 10uM | 6h | FAM102A | 0.00 | 2.31 |
| HT29_14_GR_ChDB_0157 | Afatinib | 10uM | 6h | DAAM1 | 0.00 | 2.28 |
| HT29_14_GR_ChDB_0157 | Afatinib | 10uM | 6h | AGA | 0.00 | 2.46 |
| HT29_14_GR_ChDB_0157 | Afatinib | 10uM | 6h | SNX16 | 0.00 | 2.00 |
| HT29_14_GR_ChDB_0157 | Afatinib | 10uM | 6h | PIP5K1B | 0.00 | 2.03 |
| HT29_14_GR_ChDB_0157 | Afatinib | 10uM | 6h | ARSD | 0.00 | 2.06 |
| HT29_14_GR_ChDB_0157 | Afatinib | 10uM | 6h | BBIP1 | 0.00 | 2.29 |
| HT29_14_GR_ChDB_0157 | Afatinib | 10uM | 6h | IFRD1 | 0.00 | 4.26 |
| HT29_14_GR_ChDB_0157 | Afatinib | 10uM | 6h | NUPR1 | 0.00 | 7.36 |
| HT29_14_GR_ChDB_0157 | Afatinib | 10uM | 6h | RRP9 | 0.00 | 0.49 |
| HT29_14_GR_ChDB_0157 | Afatinib | 10uM | 6h | GTPBP4 | 0.00 | 0.46 |
| HT29_14_GR_ChDB_0157 | Afatinib | 10uM | 6h | U2SURP | 0.00 | 0.47 |
| HT29_14_GR_ChDB_0157 | Afatinib | 10uM | 6h | FGD2 | 0.00 | 3.78 |
| HT29_14_GR_ChDB_0157 | Afatinib | 10uM | 6h | GCLM | 0.00 | 2.20 |
| HT29_14_GR_ChDB_0157 | Afatinib | 10uM | 6h | ASS1 | 0.00 | 2.38 |
| HT29_14_GR_ChDB_0157 | Afatinib | 10uM | 6h | WASF2 | 0.00 | 2.47 |
| HT29_14_GR_ChDB_0157 | Afatinib | 10uM | 6h | FOXO3 | 0.00 | 2.35 |
| HT29_14_GR_ChDB_0157 | Afatinib | 10uM | 6h | PSAT1 | 0.00 | 2.07 |
| HT29_14_GR_ChDB_0157 | Afatinib | 10uM | 6h | XPO1 | 0.00 | 2.08 |
| HT29_14_GR_ChDB_0157 | Afatinib | 10uM | 6h | DDX17 | 0.00 | 0.47 |
| HT29_14_GR_ChDB_0157 | Afatinib | 10uM | 6h | MAPKAP1 | 0.00 | 0.49 |
| HT29_14_GR_ChDB_0157 | Afatinib | 10uM | 6h | WNT11 | 0.00 | 0.30 |
| HT29_14_GR_ChDB_0157 | Afatinib | 10uM | 6h | OSER1 | 0.00 | 0.47 |
| HT29_14_GR_ChDB_0157 | Afatinib | 10uM | 6h | XIAP | 0.00 | 0.50 |
| HT29_14_GR_ChDB_0157 | Afatinib | 10uM | 6h | TNFRSF10D | 0.00 | 0.43 |
| HT29_2_GR_ChDB_0018 | Gemcitabine | 1uM | 6h | AURKA | 1.00 | 0.36 |
| HT29_2_GR_ChDB_0018 | Gemcitabine | 1uM | 6h | PDPK1 | 0.83 | 0.45 |
| HT29_2_GR_ChDB_0018 | Gemcitabine | 1uM | 6h | KDM5B | 0.28 | 0.48 |
| HT29_2_GR_ChDB_0018 | Gemcitabine | 1uM | 6h | IL8 | 0.23 | 3.12 |
| HT29_2_GR_ChDB_0018 | Gemcitabine | 1uM | 6h | CENPE | 0.22 | 0.29 |
| HT29_2_GR_ChDB_0018 | Gemcitabine | 1uM | 6h | TOP2A | 0.19 | 0.46 |
| HT29_2_GR_ChDB_0018 | Gemcitabine | 1uM | 6h | ADM | 0.11 | 2.52 |
| HT29_2_GR_ChDB_0018 | Gemcitabine | 1uM | 6h | KIF20A | 0.10 | 0.21 |
| HT29_2_GR_ChDB_0018 | Gemcitabine | 1uM | 6h | CCNE1 | 0.03 | 2.31 |
| HT29_2_GR_ChDB_0018 | Gemcitabine | 1uM | 6h | DLGAP5 | 0.02 | 0.44 |
| HT29_2_GR_ChDB_0018 | Gemcitabine | 1uM | 6h | SFN | 0.02 | 2.14 |
| HT29_2_GR_ChDB_0018 | Gemcitabine | 1uM | 6h | PTGS2 | 0.01 | 2.41 |
| HT29_2_GR_ChDB_0018 | Gemcitabine | 1uM | 6h | CXCR4 | 0.01 | 0.47 |
| HT29_2_GR_ChDB_0018 | Gemcitabine | 1uM | 6h | PSRC1 | 0.00 | 0.38 |
| HT29_3_GR_ChDB_0021 | Mitomycin C | 10uM | 6h | MYC | 1.00 | 0.44 |
| HT29_3_GR_ChDB_0021 | Mitomycin C | 10uM | 6h | RNF19B | 0.46 | 2.20 |
| HT29_3_GR_ChDB_0021 | Mitomycin C | 10uM | 6h | TNIK | 0.09 | 0.41 |
| HT29_3_GR_ChDB_0021 | Mitomycin C | 10uM | 6h | CCNE2 | 0.00 | 3.71 |
| HT29_3_GR_ChDB_0021 | Mitomycin C | 10uM | 6h | CDK5R1 | 0.00 | 2.33 |
| HT29_3_GR_ChDB_0021 | Mitomycin C | 10uM | 6h | MYO10 | 0.00 | 0.47 |
| HT29_3_GR_ChDB_0021 | Mitomycin C | 10uM | 6h | CDK6 | 0.00 | 0.47 |
| HT29_3_GR_ChDB_0021 | Mitomycin C | 10uM | 6h | SMAD3 | 0.00 | 0.48 |
| HT29_3_GR_ChDB_0021 | Mitomycin C | 10uM | 6h | EXT1 | 0.00 | 0.28 |
| HT29_3_GR_ChDB_0021 | Mitomycin C | 10uM | 6h | CENPE | 0.00 | 0.28 |
| HT29_3_GR_ChDB_0021 | Mitomycin C | 10uM | 6h | KLHL7 | 0.00 | 2.57 |
| HT29_3_GR_ChDB_0021 | Mitomycin C | 10uM | 6h | ANK3 | 0.00 | 0.33 |
| HT29_3_GR_ChDB_0021 | Mitomycin C | 10uM | 6h | MBNL2 | 0.00 | 0.44 |
| HT29_3_GR_ChDB_0021 | Mitomycin C | 10uM | 6h | H1F0 | 0.00 | 0.32 |
| HT29_3_GR_ChDB_0021 | Mitomycin C | 10uM | 6h | TCF7L2 | 0.00 | 0.37 |
| HT29_3_GR_ChDB_0021 | Mitomycin C | 10uM | 6h | INPP5A | 0.00 | 0.49 |
| HT29_3_GR_ChDB_0021 | Mitomycin C | 10uM | 6h | SRPK2 | 0.00 | 0.47 |
| HT29_3_GR_ChDB_0021 | Mitomycin C | 10uM | 6h | OGT | 0.00 | 0.44 |
| HT29_3_GR_ChDB_0021 | Mitomycin C | 10uM | 6h | CDKN2B | 0.00 | 2.29 |
| HT29_3_GR_ChDB_0021 | Mitomycin C | 10uM | 6h | IRF7 | 0.00 | 2.01 |
| HT29_3_GR_ChDB_0021 | Mitomycin C | 10uM | 6h | E2F8 | 0.00 | 2.02 |
| HT29_3_GR_ChDB_0021 | Mitomycin C | 10uM | 6h | PSRC1 | 0.00 | 0.38 |
| HT29_3_GR_ChDB_0021 | Mitomycin C | 10uM | 6h | PTK2 | 0.00 | 0.45 |
| HT29_3_GR_ChDB_0026 | Methotrexate | 1uM | 6h | MYC | 1.00 | 0.44 |
| HT29_3_GR_ChDB_0026 | Methotrexate | 1uM | 6h | PIK3R1 | 0.39 | 0.47 |
| HT29_3_GR_ChDB_0026 | Methotrexate | 1uM | 6h | AURKA | 0.13 | 0.33 |
| HT29_3_GR_ChDB_0026 | Methotrexate | 1uM | 6h | KIF20A | 0.07 | 0.26 |
| HT29_3_GR_ChDB_0026 | Methotrexate | 1uM | 6h | IL8 | 0.06 | 2.61 |
| HT29_3_GR_ChDB_0026 | Methotrexate | 1uM | 6h | TOP2A | 0.05 | 0.48 |
| HT29_3_GR_ChDB_0026 | Methotrexate | 1uM | 6h | CENPE | 0.03 | 0.35 |
| HT29_3_GR_ChDB_0026 | Methotrexate | 1uM | 6h | DLGAP5 | 0.02 | 0.38 |
| HT29_3_GR_ChDB_0026 | Methotrexate | 1uM | 6h | TSEN2 | 0.02 | 0.41 |
| HT29_3_GR_ChDB_0026 | Methotrexate | 1uM | 6h | PHF1 | 0.01 | 2.21 |
| HT29_3_GR_ChDB_0026 | Methotrexate | 1uM | 6h | AKR1C1 | 0.00 | 2.08 |
| HT29_3_GR_ChDB_0026 | Methotrexate | 1uM | 6h | PTGS2 | 0.00 | 2.29 |
| HT29_3_GR_ChDB_0026 | Methotrexate | 1uM | 6h | MAT2A | 0.00 | 0.43 |
| HT29_3_GR_ChDB_0028 | Temsirolimus | 10uM | 6h | SQLE | 1.00 | 0.48 |
| HT29_3_GR_ChDB_0028 | Temsirolimus | 10uM | 6h | SQSTM1 | 0.82 | 2.39 |
| HT29_3_GR_ChDB_0028 | Temsirolimus | 10uM | 6h | GNAS | 0.34 | 2.43 |
| HT29_3_GR_ChDB_0028 | Temsirolimus | 10uM | 6h | EXOSC4 | 0.32 | 0.46 |
| HT29_3_GR_ChDB_0028 | Temsirolimus | 10uM | 6h | BCCIP | 0.25 | 0.49 |
| HT29_3_GR_ChDB_0028 | Temsirolimus | 10uM | 6h | CTH | 0.24 | 3.04 |
| HT29_3_GR_ChDB_0028 | Temsirolimus | 10uM | 6h | CREB3L4 | 0.19 | 2.53 |
| HT29_3_GR_ChDB_0028 | Temsirolimus | 10uM | 6h | RNF167 | 0.16 | 2.12 |
| HT29_3_GR_ChDB_0028 | Temsirolimus | 10uM | 6h | SGK2 | 0.14 | 0.40 |
| HT29_3_GR_ChDB_0028 | Temsirolimus | 10uM | 6h | BCL6 | 0.09 | 2.02 |
| HT29_3_GR_ChDB_0028 | Temsirolimus | 10uM | 6h | HAGH | 0.07 | 2.52 |
| HT29_3_GR_ChDB_0028 | Temsirolimus | 10uM | 6h | SLC7A11 | 0.07 | 3.17 |
| HT29_3_GR_ChDB_0028 | Temsirolimus | 10uM | 6h | PLAUR | 0.06 | 2.12 |
| HT29_3_GR_ChDB_0028 | Temsirolimus | 10uM | 6h | DDX5 | 0.05 | 0.42 |
| HT29_3_GR_ChDB_0028 | Temsirolimus | 10uM | 6h | BNIP3L | 0.05 | 2.41 |
| HT29_3_GR_ChDB_0028 | Temsirolimus | 10uM | 6h | HBP1 | 0.05 | 3.79 |
| HT29_3_GR_ChDB_0028 | Temsirolimus | 10uM | 6h | MBNL2 | 0.05 | 2.22 |
| HT29_3_GR_ChDB_0028 | Temsirolimus | 10uM | 6h | NDRG1 | 0.04 | 3.77 |
| HT29_3_GR_ChDB_0028 | Temsirolimus | 10uM | 6h | CBLB | 0.02 | 2.40 |
| HT29_3_GR_ChDB_0028 | Temsirolimus | 10uM | 6h | DNAJB9 | 0.02 | 2.13 |
| HT29_3_GR_ChDB_0028 | Temsirolimus | 10uM | 6h | SOS2 | 0.01 | 2.42 |
| HT29_3_GR_ChDB_0028 | Temsirolimus | 10uM | 6h | HMGCS1 | 0.00 | 0.50 |
| HT29_3_GR_ChDB_0028 | Temsirolimus | 10uM | 6h | CHMP1B | 0.00 | 2.14 |
| HT29_3_GR_ChDB_0028 | Temsirolimus | 10uM | 6h | KDM4B | 0.00 | 2.21 |
| HT29_3_GR_ChDB_0028 | Temsirolimus | 10uM | 6h | FAXDC2 | 0.00 | 2.75 |
| HT29_3_GR_ChDB_0028 | Temsirolimus | 10uM | 6h | ELF3 | 0.00 | 2.04 |
| HT29_3_GR_ChDB_0028 | Temsirolimus | 10uM | 6h | GULP1 | 0.00 | 3.09 |
| HT29_3_GR_ChDB_0028 | Temsirolimus | 10uM | 6h | TMCO3 | 0.00 | 2.06 |
| HT29_3_GR_ChDB_0028 | Temsirolimus | 10uM | 6h | KLHL24 | 0.00 | 3.80 |
| HT29_3_GR_ChDB_0028 | Temsirolimus | 10uM | 6h | SLC30A5 | 0.00 | 2.02 |
| HT29_3_GR_ChDB_0028 | Temsirolimus | 10uM | 6h | SEC24D | 0.00 | 3.13 |
| HT29_3_GR_ChDB_0028 | Temsirolimus | 10uM | 6h | PIP5K1B | 0.00 | 2.14 |
| HT29_3_GR_ChDB_0028 | Temsirolimus | 10uM | 6h | TMEM19 | 0.00 | 2.14 |
| HT29_3_GR_ChDB_0028 | Temsirolimus | 10uM | 6h | SYTL2 | 0.00 | 2.05 |
| HT29_3_GR_ChDB_0028 | Temsirolimus | 10uM | 6h | IDI1 | 0.00 | 0.42 |
| HT29_3_GR_ChDB_0028 | Temsirolimus | 10uM | 6h | CCPG1 | 0.00 | 2.13 |
| HT29_3_GR_ChDB_0028 | Temsirolimus | 10uM | 6h | AZI2 | 0.00 | 2.09 |
| HT29_3_GR_ChDB_0028 | Temsirolimus | 10uM | 6h | GTPBP4 | 0.00 | 0.47 |
| HT29_3_GR_ChDB_0028 | Temsirolimus | 10uM | 6h | RNASEH1 | 0.00 | 0.47 |
| HT29_3_GR_ChDB_0028 | Temsirolimus | 10uM | 6h | SPSB3 | 0.00 | 2.67 |
| HT29_3_GR_ChDB_0028 | Temsirolimus | 10uM | 6h | CLCN7 | 0.00 | 2.13 |
| HT29_3_GR_ChDB_0028 | Temsirolimus | 10uM | 6h | LAMB3 | 0.00 | 2.46 |
| HT29_3_GR_ChDB_0028 | Temsirolimus | 10uM | 6h | GOLGB1 | 0.00 | 2.11 |
| HT29_3_GR_ChDB_0028 | Temsirolimus | 10uM | 6h | NBR1 | 0.00 | 2.04 |
| HT29_3_GR_ChDB_0028 | Temsirolimus | 10uM | 6h | MARCKS | 0.00 | 2.19 |
| HT29_3_GR_ChDB_0028 | Temsirolimus | 10uM | 6h | NUCB2 | 0.00 | 2.47 |
| HT29_3_GR_ChDB_0028 | Temsirolimus | 10uM | 6h | STAT2 | 0.00 | 2.36 |
| HT29_3_GR_ChDB_0028 | Temsirolimus | 10uM | 6h | VIL1 | 0.00 | 2.18 |
| HT29_3_GR_ChDB_0028 | Temsirolimus | 10uM | 6h | ANKRA2 | 0.00 | 2.94 |
| HT29_3_GR_ChDB_0028 | Temsirolimus | 10uM | 6h | DEPTOR | 0.00 | 2.55 |
| HT29_3_GR_ChDB_0028 | Temsirolimus | 10uM | 6h | ULBP2 | 0.00 | 2.56 |
| HT29_3_GR_ChDB_0028 | Temsirolimus | 10uM | 6h | GDF15 | 0.00 | 2.77 |
| HT29_3_GR_ChDB_0028 | Temsirolimus | 10uM | 6h | ZNF623 | 0.00 | 2.41 |
| HT29_3_GR_ChDB_0028 | Temsirolimus | 10uM | 6h | NR1D2 | 0.00 | 2.66 |
| HT29_3_GR_ChDB_0028 | Temsirolimus | 10uM | 6h | CRBN | 0.00 | 2.46 |
| HT29_3_GR_ChDB_0028 | Temsirolimus | 10uM | 6h | MRPS12 | 0.00 | 0.41 |
| HT29_3_GR_ChDB_0028 | Temsirolimus | 10uM | 6h | STIP1 | 0.00 | 0.49 |
| HT29_3_GR_ChDB_0028 | Temsirolimus | 10uM | 6h | CCNG2 | 0.00 | 4.33 |
| HT29_3_GR_ChDB_0028 | Temsirolimus | 10uM | 6h | DENND4C | 0.00 | 2.07 |
| HT29_3_GR_ChDB_0028 | Temsirolimus | 10uM | 6h | BBIP1 | 0.00 | 2.29 |
| HT29_3_GR_ChDB_0028 | Temsirolimus | 10uM | 6h | MARCH8 | 0.00 | 2.04 |
| HT29_3_GR_ChDB_0028 | Temsirolimus | 10uM | 6h | AMIGO2 | 0.00 | 2.13 |
| HT29_3_GR_ChDB_0028 | Temsirolimus | 10uM | 6h | BICD2 | 0.00 | 0.49 |
| HT29_3_GR_ChDB_0028 | Temsirolimus | 10uM | 6h | PCF11 | 0.00 | 2.07 |
| HT29_3_GR_ChDB_0028 | Temsirolimus | 10uM | 6h | CYP3A5 | 0.00 | 2.73 |
| HT29_3_GR_ChDB_0028 | Temsirolimus | 10uM | 6h | SCEL | 0.00 | 2.54 |
| HT29_3_GR_ChDB_0028 | Temsirolimus | 10uM | 6h | ITGB6 | 0.00 | 2.67 |
| HT29_3_GR_ChDB_0028 | Temsirolimus | 10uM | 6h | PNRC1 | 0.00 | 3.54 |
| HT29_3_GR_ChDB_0028 | Temsirolimus | 10uM | 6h | ATP9A | 0.00 | 2.24 |
| HT29_3_GR_ChDB_0028 | Temsirolimus | 10uM | 6h | CREB3L2 | 0.00 | 2.68 |
| HT29_3_GR_ChDB_0028 | Temsirolimus | 10uM | 6h | ZNF33B | 0.00 | 2.27 |
| HT29_3_GR_ChDB_0028 | Temsirolimus | 10uM | 6h | GPRC5C | 0.00 | 2.69 |
| HT29_3_GR_ChDB_0028 | Temsirolimus | 10uM | 6h | RBMS2 | 0.00 | 2.05 |
| HT29_3_GR_ChDB_0028 | Temsirolimus | 10uM | 6h | YPEL5 | 0.00 | 2.07 |
| HT29_3_GR_ChDB_0028 | Temsirolimus | 10uM | 6h | APOBEC3B | 0.00 | 3.09 |
| HT29_3_GR_ChDB_0028 | Temsirolimus | 10uM | 6h | KRIT1 | 0.00 | 2.35 |
| HT29_3_GR_ChDB_0028 | Temsirolimus | 10uM | 6h | SMIM14 | 0.00 | 2.74 |
| HT29_3_GR_ChDB_0028 | Temsirolimus | 10uM | 6h | PSMD11 | 0.00 | 0.43 |
| HT29_3_GR_ChDB_0028 | Temsirolimus | 10uM | 6h | GALNT6 | 0.00 | 0.48 |
| HT29_3_GR_ChDB_0028 | Temsirolimus | 10uM | 6h | DHTKD1 | 0.00 | 2.16 |
| HT29_3_GR_ChDB_0028 | Temsirolimus | 10uM | 6h | LAMA3 | 0.00 | 2.76 |
| HT29_3_GR_ChDB_0028 | Temsirolimus | 10uM | 6h | ARFGAP3 | 0.00 | 2.50 |
| HT29_3_GR_ChDB_0028 | Temsirolimus | 10uM | 6h | LPP | 0.00 | 2.40 |
| HT29_3_GR_ChDB_0028 | Temsirolimus | 10uM | 6h | SLC33A1 | 0.00 | 2.52 |
| HT29_3_GR_ChDB_0028 | Temsirolimus | 10uM | 6h | CCDC28A | 0.00 | 3.20 |
| HT29_3_GR_ChDB_0028 | Temsirolimus | 10uM | 6h | SLC1A4 | 0.00 | 2.08 |
| HT29_3_GR_ChDB_0028 | Temsirolimus | 10uM | 6h | HECA | 0.00 | 2.28 |
| HT29_3_GR_ChDB_0028 | Temsirolimus | 10uM | 6h | RNF125 | 0.00 | 2.15 |
| HT29_3_GR_ChDB_0028 | Temsirolimus | 10uM | 6h | HOXB5 | 0.00 | 2.80 |
| HT29_3_GR_ChDB_0028 | Temsirolimus | 10uM | 6h | SERINC1 | 0.00 | 2.09 |
| HT29_3_GR_ChDB_0028 | Temsirolimus | 10uM | 6h | EFNA1 | 0.00 | 2.47 |
| HT29_3_GR_ChDB_0028 | Temsirolimus | 10uM | 6h | INF2 | 0.00 | 0.47 |
| HT29_3_GR_ChDB_0028 | Temsirolimus | 10uM | 6h | ARMC7 | 0.00 | 0.46 |
| HT29_3_GR_ChDB_0028 | Temsirolimus | 10uM | 6h | HIP1 | 0.00 | 0.50 |
| HT29_3_GR_ChDB_0028 | Temsirolimus | 10uM | 6h | MCM10 | 0.00 | 0.46 |
| HT29_4_GR_ChDB_0033 | SN38 | 3uM | 6h | ADM | 1.00 | 5.87 |
| HT29_4_GR_ChDB_0033 | SN38 | 3uM | 6h | JUNB | 0.14 | 3.51 |
| HT29_4_GR_ChDB_0033 | SN38 | 3uM | 6h | TGFBR2 | 0.11 | 0.17 |
| HT29_4_GR_ChDB_0033 | SN38 | 3uM | 6h | MAPK14 | 0.10 | 0.32 |
| HT29_4_GR_ChDB_0033 | SN38 | 3uM | 6h | CDK6 | 0.07 | 0.49 |
| HT29_4_GR_ChDB_0033 | SN38 | 3uM | 6h | PRKCZ | 0.07 | 0.31 |
| HT29_4_GR_ChDB_0033 | SN38 | 3uM | 6h | KRAS | 0.06 | 0.43 |
| HT29_4_GR_ChDB_0033 | SN38 | 3uM | 6h | CREB1 | 0.04 | 0.30 |
| HT29_4_GR_ChDB_0033 | SN38 | 3uM | 6h | BRD4 | 0.04 | 0.33 |
| HT29_4_GR_ChDB_0033 | SN38 | 3uM | 6h | AIMP1 | 0.04 | 2.29 |
| HT29_4_GR_ChDB_0033 | SN38 | 3uM | 6h | ABL1 | 0.04 | 0.44 |
| HT29_4_GR_ChDB_0033 | SN38 | 3uM | 6h | PRKACA | 0.03 | 2.30 |
| HT29_4_GR_ChDB_0033 | SN38 | 3uM | 6h | PTK2 | 0.03 | 0.38 |
| HT29_4_GR_ChDB_0033 | SN38 | 3uM | 6h | RXRA | 0.02 | 0.28 |
| HT29_4_GR_ChDB_0033 | SN38 | 3uM | 6h | ZNF416 | 0.02 | 3.21 |
| HT29_4_GR_ChDB_0033 | SN38 | 3uM | 6h | GRWD1 | 0.02 | 0.41 |
| HT29_4_GR_ChDB_0033 | SN38 | 3uM | 6h | TUBB2A | 0.02 | 3.24 |
| HT29_4_GR_ChDB_0033 | SN38 | 3uM | 6h | PTGS2 | 0.02 | 2.63 |
| HT29_4_GR_ChDB_0033 | SN38 | 3uM | 6h | WNK1 | 0.02 | 0.27 |
| HT29_4_GR_ChDB_0033 | SN38 | 3uM | 6h | IRS1 | 0.02 | 0.30 |
| HT29_4_GR_ChDB_0033 | SN38 | 3uM | 6h | SORD | 0.02 | 2.16 |
| HT29_4_GR_ChDB_0033 | SN38 | 3uM | 6h | RASSF1 | 0.01 | 2.57 |
| HT29_4_GR_ChDB_0033 | SN38 | 3uM | 6h | MYB | 0.01 | 0.22 |
| HT29_4_GR_ChDB_0033 | SN38 | 3uM | 6h | OGG1 | 0.01 | 2.15 |
| HT29_4_GR_ChDB_0033 | SN38 | 3uM | 6h | TCF7L2 | 0.01 | 0.10 |
| HT29_4_GR_ChDB_0033 | SN38 | 3uM | 6h | CHEK1 | 0.01 | 0.46 |
| HT29_4_GR_ChDB_0033 | SN38 | 3uM | 6h | MKNK2 | 0.01 | 2.96 |
| HT29_4_GR_ChDB_0033 | SN38 | 3uM | 6h | RAF1 | 0.01 | 0.30 |
| HT29_4_GR_ChDB_0033 | SN38 | 3uM | 6h | ZMIZ1 | 0.01 | 0.20 |
| HT29_4_GR_ChDB_0033 | SN38 | 3uM | 6h | LGALS8 | 0.01 | 2.62 |
| HT29_4_GR_ChDB_0033 | SN38 | 3uM | 6h | CFLAR | 0.01 | 0.28 |
| HT29_4_GR_ChDB_0033 | SN38 | 3uM | 6h | EXT1 | 0.01 | 0.09 |
| HT29_4_GR_ChDB_0033 | SN38 | 3uM | 6h | DDX49 | 0.01 | 2.07 |
| HT29_4_GR_ChDB_0033 | SN38 | 3uM | 6h | NCOA3 | 0.01 | 0.38 |
| HT29_4_GR_ChDB_0033 | SN38 | 3uM | 6h | ERCC1 | 0.01 | 2.31 |
| HT29_4_GR_ChDB_0033 | SN38 | 3uM | 6h | SGK1 | 0.01 | 2.14 |
| HT29_4_GR_ChDB_0033 | SN38 | 3uM | 6h | DCLRE1B | 0.01 | 2.92 |
| HT29_4_GR_ChDB_0033 | SN38 | 3uM | 6h | FOS | 0.01 | 2.23 |
| HT29_4_GR_ChDB_0033 | SN38 | 3uM | 6h | USP7 | 0.01 | 0.31 |
| HT29_4_GR_ChDB_0033 | SN38 | 3uM | 6h | PSRC1 | 0.01 | 0.21 |
| HT29_4_GR_ChDB_0033 | SN38 | 3uM | 6h | PHF13 | 0.01 | 2.66 |
| HT29_4_GR_ChDB_0033 | SN38 | 3uM | 6h | GK5 | 0.01 | 0.48 |
| HT29_4_GR_ChDB_0033 | SN38 | 3uM | 6h | PTEN | 0.01 | 0.37 |
| HT29_4_GR_ChDB_0033 | SN38 | 3uM | 6h | NEU1 | 0.01 | 2.07 |
| HT29_4_GR_ChDB_0033 | SN38 | 3uM | 6h | GABPB1 | 0.00 | 0.25 |
| HT29_4_GR_ChDB_0033 | SN38 | 3uM | 6h | RPS6KA3 | 0.00 | 0.40 |
| HT29_4_GR_ChDB_0033 | SN38 | 3uM | 6h | DDX42 | 0.00 | 0.36 |
| HT29_4_GR_ChDB_0033 | SN38 | 3uM | 6h | MBNL2 | 0.00 | 0.23 |
| HT29_4_GR_ChDB_0033 | SN38 | 3uM | 6h | ETNK1 | 0.00 | 0.44 |
| HT29_4_GR_ChDB_0033 | SN38 | 3uM | 6h | SENP6 | 0.00 | 0.26 |
| HT29_4_GR_ChDB_0033 | SN38 | 3uM | 6h | HSBP1 | 0.00 | 2.78 |
| HT29_4_GR_ChDB_0033 | SN38 | 3uM | 6h | CHKA | 0.00 | 0.27 |
| HT29_4_GR_ChDB_0033 | SN38 | 3uM | 6h | CCNA2 | 0.00 | 0.34 |
| HT29_4_GR_ChDB_0033 | SN38 | 3uM | 6h | STK24 | 0.00 | 0.34 |
| HT29_4_GR_ChDB_0033 | SN38 | 3uM | 6h | TP53BP1 | 0.00 | 0.49 |
| HT29_4_GR_ChDB_0033 | SN38 | 3uM | 6h | CDK7 | 0.00 | 0.33 |
| HT29_4_GR_ChDB_0033 | SN38 | 3uM | 6h | ZBTB9 | 0.00 | 2.96 |
| HT29_4_GR_ChDB_0033 | SN38 | 3uM | 6h | ASF1B | 0.00 | 2.72 |
| HT29_4_GR_ChDB_0033 | SN38 | 3uM | 6h | PRMT2 | 0.00 | 2.09 |
| HT29_4_GR_ChDB_0033 | SN38 | 3uM | 6h | POLE2 | 0.00 | 0.46 |
| HT29_4_GR_ChDB_0033 | SN38 | 3uM | 6h | GATA6 | 0.00 | 0.39 |
| HT29_4_GR_ChDB_0033 | SN38 | 3uM | 6h | NOTCH1 | 0.00 | 0.46 |
| HT29_4_GR_ChDB_0033 | SN38 | 3uM | 6h | ZAK | 0.00 | 0.40 |
| HT29_4_GR_ChDB_0033 | SN38 | 3uM | 6h | MAP3K4 | 0.00 | 0.21 |
| HT29_4_GR_ChDB_0033 | SN38 | 3uM | 6h | PRKCQ | 0.00 | 0.44 |
| HT29_4_GR_ChDB_0033 | SN38 | 3uM | 6h | PPP3CA | 0.00 | 0.47 |
| HT29_4_GR_ChDB_0033 | SN38 | 3uM | 6h | BUB1B | 0.00 | 0.09 |
| HT29_4_GR_ChDB_0033 | SN38 | 3uM | 6h | KIF2C | 0.00 | 0.46 |
| HT29_4_GR_ChDB_0033 | SN38 | 3uM | 6h | MELK | 0.00 | 0.32 |
| HT29_4_GR_ChDB_0033 | SN38 | 3uM | 6h | KIF11 | 0.00 | 0.13 |
| HT29_4_GR_ChDB_0033 | SN38 | 3uM | 6h | ABHD6 | 0.00 | 0.45 |
| HT29_4_GR_ChDB_0033 | SN38 | 3uM | 6h | TMEM2 | 0.00 | 0.27 |
| HT29_4_GR_ChDB_0033 | SN38 | 3uM | 6h | CHD1 | 0.00 | 0.18 |
| HT29_4_GR_ChDB_0033 | SN38 | 3uM | 6h | PIK3C3 | 0.00 | 0.34 |
| HT29_4_GR_ChDB_0033 | SN38 | 3uM | 6h | SCNN1A | 0.00 | 0.45 |
| HT29_4_GR_ChDB_0033 | SN38 | 3uM | 6h | ETS2 | 0.00 | 0.34 |
| HT29_4_GR_ChDB_0033 | SN38 | 3uM | 6h | MET | 0.00 | 0.40 |
| HT29_4_GR_ChDB_0033 | SN38 | 3uM | 6h | SMC4 | 0.00 | 0.43 |
| HT29_4_GR_ChDB_0033 | SN38 | 3uM | 6h | SMURF1 | 0.00 | 0.21 |
| HT29_4_GR_ChDB_0033 | SN38 | 3uM | 6h | PPARG | 0.00 | 0.24 |
| HT29_4_GR_ChDB_0033 | SN38 | 3uM | 6h | MED6 | 0.00 | 0.46 |
| HT29_4_GR_ChDB_0033 | SN38 | 3uM | 6h | GSK3B | 0.00 | 0.20 |
| HT29_4_GR_ChDB_0033 | SN38 | 3uM | 6h | MAP7 | 0.00 | 0.45 |
| HT29_4_GR_ChDB_0033 | SN38 | 3uM | 6h | E2F3 | 0.00 | 0.44 |
| HT29_4_GR_ChDB_0033 | SN38 | 3uM | 6h | RPS6KB1 | 0.00 | 0.33 |
| HT29_4_GR_ChDB_0033 | SN38 | 3uM | 6h | FGFR2 | 0.00 | 0.40 |
| HT29_4_GR_ChDB_0033 | SN38 | 3uM | 6h | SMAD6 | 0.00 | 0.37 |
| HT29_4_GR_ChDB_0033 | SN38 | 3uM | 6h | MDM2 | 0.00 | 0.32 |
| HT29_4_GR_ChDB_0033 | SN38 | 3uM | 6h | HBEGF | 0.00 | 4.47 |
| HT29_4_GR_ChDB_0033 | SN38 | 3uM | 6h | GTF2E2 | 0.00 | 0.49 |
| HT29_4_GR_ChDB_0033 | SN38 | 3uM | 6h | CENPF | 0.00 | 0.35 |
| HT29_4_GR_ChDB_0033 | SN38 | 3uM | 6h | GADD45B | 0.00 | 3.78 |
| HT29_4_GR_ChDB_0033 | SN38 | 3uM | 6h | NIPBL | 0.00 | 0.31 |
| HT29_4_GR_ChDB_0033 | SN38 | 3uM | 6h | PLK4 | 0.00 | 0.43 |
| HT29_4_GR_ChDB_0033 | SN38 | 3uM | 6h | TIMM22 | 0.00 | 2.43 |
| HT29_4_GR_ChDB_0033 | SN38 | 3uM | 6h | PDS5A | 0.00 | 0.33 |
| HT29_4_GR_ChDB_0033 | SN38 | 3uM | 6h | HNRNPDL | 0.00 | 0.40 |
| HT29_4_GR_ChDB_0033 | SN38 | 3uM | 6h | UBR5 | 0.00 | 0.33 |
| HT29_4_GR_ChDB_0033 | SN38 | 3uM | 6h | USP34 | 0.00 | 0.34 |
| HT29_4_GR_ChDB_0033 | SN38 | 3uM | 6h | TRIO | 0.00 | 0.33 |
| HT29_4_GR_ChDB_0033 | SN38 | 3uM | 6h | CDC16 | 0.00 | 0.48 |
| HT29_4_GR_ChDB_0033 | SN38 | 3uM | 6h | RBM48 | 0.00 | 2.75 |
| HT29_4_GR_ChDB_0033 | SN38 | 3uM | 6h | ID2 | 0.00 | 3.21 |
| HT29_4_GR_ChDB_0033 | SN38 | 3uM | 6h | RBM3 | 0.00 | 2.56 |
| HT29_4_GR_ChDB_0033 | SN38 | 3uM | 6h | GTF3C2 | 0.00 | 0.49 |
| HT29_4_GR_ChDB_0033 | SN38 | 3uM | 6h | ASPM | 0.00 | 0.21 |
| HT29_4_GR_ChDB_0033 | SN38 | 3uM | 6h | SMCHD1 | 0.00 | 0.25 |
| HT29_4_GR_ChDB_0033 | SN38 | 3uM | 6h | RAPGEF2 | 0.00 | 0.23 |
| HT29_4_GR_ChDB_0033 | SN38 | 3uM | 6h | UBE3A | 0.00 | 0.25 |
| HT29_4_GR_ChDB_0033 | SN38 | 3uM | 6h | MKI67 | 0.00 | 0.43 |
| HT29_4_GR_ChDB_0033 | SN38 | 3uM | 6h | FYN | 0.00 | 0.32 |
| HT29_4_GR_ChDB_0033 | SN38 | 3uM | 6h | NCOA2 | 0.00 | 0.20 |
| HT29_4_GR_ChDB_0033 | SN38 | 3uM | 6h | PAIP1 | 0.00 | 0.17 |
| HT29_4_GR_ChDB_0033 | SN38 | 3uM | 6h | G3BP1 | 0.00 | 0.33 |
| HT29_4_GR_ChDB_0033 | SN38 | 3uM | 6h | CCNB1 | 0.00 | 0.37 |
| HT29_4_GR_ChDB_0033 | SN38 | 3uM | 6h | DCUN1D4 | 0.00 | 0.31 |
| HT29_4_GR_ChDB_0033 | SN38 | 3uM | 6h | GDF15 | 0.00 | 0.45 |
| HT29_4_GR_ChDB_0033 | SN38 | 3uM | 6h | ITSN1 | 0.00 | 0.43 |
| HT29_4_GR_ChDB_0033 | SN38 | 3uM | 6h | PRRC2C | 0.00 | 0.36 |
| HT29_4_GR_ChDB_0033 | SN38 | 3uM | 6h | CTBP2 | 0.00 | 0.23 |
| HT29_4_GR_ChDB_0033 | SN38 | 3uM | 6h | CAMSAP2 | 0.00 | 0.26 |
| HT29_4_GR_ChDB_0033 | SN38 | 3uM | 6h | NFIB | 0.00 | 0.30 |
| HT29_4_GR_ChDB_0033 | SN38 | 3uM | 6h | RGS2 | 0.00 | 2.37 |
| HT29_4_GR_ChDB_0033 | SN38 | 3uM | 6h | TACSTD2 | 0.00 | 2.28 |
| HT29_4_GR_ChDB_0033 | SN38 | 3uM | 6h | MTHFR | 0.00 | 2.13 |
| HT29_4_GR_ChDB_0033 | SN38 | 3uM | 6h | HLA-E | 0.00 | 3.13 |
| HT29_4_GR_ChDB_0033 | SN38 | 3uM | 6h | RFC3 | 0.00 | 0.40 |
| HT29_4_GR_ChDB_0033 | SN38 | 3uM | 6h | GTSE1 | 0.00 | 0.40 |
| HT29_4_GR_ChDB_0033 | SN38 | 3uM | 6h | BUB1 | 0.00 | 0.47 |
| HT29_4_GR_ChDB_0033 | SN38 | 3uM | 6h | NCK1 | 0.00 | 0.35 |
| HT29_4_GR_ChDB_0033 | SN38 | 3uM | 6h | UBAP2L | 0.00 | 0.42 |
| HT29_4_GR_ChDB_0033 | SN38 | 3uM | 6h | SLC11A2 | 0.00 | 0.42 |
| HT29_4_GR_ChDB_0033 | SN38 | 3uM | 6h | MTF2 | 0.00 | 0.29 |
| HT29_4_GR_ChDB_0033 | SN38 | 3uM | 6h | SETD2 | 0.00 | 0.25 |
| HT29_4_GR_ChDB_0033 | SN38 | 3uM | 6h | SKP2 | 0.00 | 0.40 |
| HT29_4_GR_ChDB_0033 | SN38 | 3uM | 6h | SMC3 | 0.00 | 0.27 |
| HT29_4_GR_ChDB_0033 | SN38 | 3uM | 6h | ANKRD12 | 0.00 | 0.19 |
| HT29_4_GR_ChDB_0033 | SN38 | 3uM | 6h | HMGXB4 | 0.00 | 0.48 |
| HT29_4_GR_ChDB_0033 | SN38 | 3uM | 6h | CSNK1A1 | 0.00 | 0.44 |
| HT29_4_GR_ChDB_0033 | SN38 | 3uM | 6h | PPP2R5C | 0.00 | 0.40 |
| HT29_4_GR_ChDB_0033 | SN38 | 3uM | 6h | PRKD3 | 0.00 | 0.37 |
| HT29_4_GR_ChDB_0033 | SN38 | 3uM | 6h | PHTF2 | 0.00 | 0.36 |
| HT29_4_GR_ChDB_0033 | SN38 | 3uM | 6h | MARCH6 | 0.00 | 0.31 |
| HT29_4_GR_ChDB_0033 | SN38 | 3uM | 6h | OGT | 0.00 | 0.39 |
| HT29_4_GR_ChDB_0033 | SN38 | 3uM | 6h | BRIP1 | 0.00 | 0.32 |
| HT29_4_GR_ChDB_0033 | SN38 | 3uM | 6h | HMGCR | 0.00 | 0.33 |
| HT29_4_GR_ChDB_0033 | SN38 | 3uM | 6h | FBXW2 | 0.00 | 0.44 |
| HT29_4_GR_ChDB_0033 | SN38 | 3uM | 6h | AURKA | 0.00 | 0.22 |
| HT29_4_GR_ChDB_0033 | SN38 | 3uM | 6h | RAI14 | 0.00 | 0.38 |
| HT29_4_GR_ChDB_0033 | SN38 | 3uM | 6h | TRAPPC10 | 0.00 | 0.44 |
| HT29_4_GR_ChDB_0033 | SN38 | 3uM | 6h | SNAPC3 | 0.00 | 0.49 |
| HT29_4_GR_ChDB_0033 | SN38 | 3uM | 6h | DUSP1 | 0.00 | 3.45 |
| HT29_4_GR_ChDB_0033 | SN38 | 3uM | 6h | CCDC92 | 0.00 | 2.53 |
| HT29_4_GR_ChDB_0033 | SN38 | 3uM | 6h | JUN | 0.00 | 3.87 |
| HT29_4_GR_ChDB_0033 | SN38 | 3uM | 6h | EGR1 | 0.00 | 4.47 |
| HT29_4_GR_ChDB_0033 | SN38 | 3uM | 6h | UBE2I | 0.00 | 2.65 |
| HT29_4_GR_ChDB_0033 | SN38 | 3uM | 6h | FZD7 | 0.00 | 2.51 |
| HT29_4_GR_ChDB_0033 | SN38 | 3uM | 6h | ING2 | 0.00 | 2.43 |
| HT29_4_GR_ChDB_0033 | SN38 | 3uM | 6h | CDK5R1 | 0.00 | 2.99 |
| HT29_4_GR_ChDB_0033 | SN38 | 3uM | 6h | CRELD2 | 0.00 | 2.10 |
| HT29_4_GR_ChDB_0033 | SN38 | 3uM | 6h | TAOK2 | 0.00 | 2.08 |
| HT29_4_GR_ChDB_0033 | SN38 | 3uM | 6h | TUBGCP3 | 0.00 | 0.35 |
| HT29_4_GR_ChDB_0033 | SN38 | 3uM | 6h | TMPO | 0.00 | 0.47 |
| HT29_4_GR_ChDB_0033 | SN38 | 3uM | 6h | EZR | 0.00 | 0.45 |
| HT29_4_GR_ChDB_0033 | SN38 | 3uM | 6h | CREBZF | 0.00 | 0.42 |
| HT29_4_GR_ChDB_0033 | SN38 | 3uM | 6h | DNAJA3 | 0.00 | 0.49 |
| HT29_4_GR_ChDB_0033 | SN38 | 3uM | 6h | TNIK | 0.00 | 0.37 |
| HT29_4_GR_ChDB_0033 | SN38 | 3uM | 6h | RRAS2 | 0.00 | 0.23 |
| HT29_4_GR_ChDB_0033 | SN38 | 3uM | 6h | PICALM | 0.00 | 0.44 |
| HT29_4_GR_ChDB_0033 | SN38 | 3uM | 6h | ZMYM4 | 0.00 | 0.38 |
| HT29_4_GR_ChDB_0033 | SN38 | 3uM | 6h | SQLE | 0.00 | 0.46 |
| HT29_4_GR_ChDB_0033 | SN38 | 3uM | 6h | SPEN | 0.00 | 0.44 |
| HT29_4_GR_ChDB_0033 | SN38 | 3uM | 6h | OTUD4 | 0.00 | 0.25 |
| HT29_4_GR_ChDB_0033 | SN38 | 3uM | 6h | KMT2A | 0.00 | 0.29 |
| HT29_4_GR_ChDB_0033 | SN38 | 3uM | 6h | SNTB1 | 0.00 | 0.35 |
| HT29_4_GR_ChDB_0033 | SN38 | 3uM | 6h | PAFAH1B1 | 0.00 | 0.23 |
| HT29_4_GR_ChDB_0033 | SN38 | 3uM | 6h | CD58 | 0.00 | 0.34 |
| HT29_4_GR_ChDB_0033 | SN38 | 3uM | 6h | EMC2 | 0.00 | 0.28 |
| HT29_4_GR_ChDB_0033 | SN38 | 3uM | 6h | MCM3AP | 0.00 | 0.37 |
| HT29_4_GR_ChDB_0033 | SN38 | 3uM | 6h | MSH6 | 0.00 | 0.45 |
| HT29_4_GR_ChDB_0033 | SN38 | 3uM | 6h | TUG1 | 0.00 | 0.38 |
| HT29_4_GR_ChDB_0033 | SN38 | 3uM | 6h | BRCA2 | 0.00 | 0.44 |
| HT29_4_GR_ChDB_0033 | SN38 | 3uM | 6h | CDYL | 0.00 | 0.24 |
| HT29_4_GR_ChDB_0033 | SN38 | 3uM | 6h | SRSF1 | 0.00 | 0.32 |
| HT29_4_GR_ChDB_0033 | SN38 | 3uM | 6h | ROCK2 | 0.00 | 0.12 |
| HT29_4_GR_ChDB_0033 | SN38 | 3uM | 6h | CSTF1 | 0.00 | 0.30 |
| HT29_4_GR_ChDB_0033 | SN38 | 3uM | 6h | U2SURP | 0.00 | 0.35 |
| HT29_4_GR_ChDB_0033 | SN38 | 3uM | 6h | RAB3GAP1 | 0.00 | 0.41 |
| HT29_4_GR_ChDB_0033 | SN38 | 3uM | 6h | TRIM2 | 0.00 | 0.43 |
| HT29_4_GR_ChDB_0033 | SN38 | 3uM | 6h | RREB1 | 0.00 | 0.12 |
| HT29_4_GR_ChDB_0033 | SN38 | 3uM | 6h | PKP4 | 0.00 | 0.43 |
| HT29_4_GR_ChDB_0033 | SN38 | 3uM | 6h | TPR | 0.00 | 0.39 |
| HT29_4_GR_ChDB_0033 | SN38 | 3uM | 6h | PTPN12 | 0.00 | 0.18 |
| HT29_4_GR_ChDB_0033 | SN38 | 3uM | 6h | ZMYND8 | 0.00 | 0.34 |
| HT29_4_GR_ChDB_0033 | SN38 | 3uM | 6h | LARP4B | 0.00 | 0.17 |
| HT29_4_GR_ChDB_0033 | SN38 | 3uM | 6h | ARID1A | 0.00 | 0.22 |
| HT29_4_GR_ChDB_0033 | SN38 | 3uM | 6h | SFN | 0.00 | 2.02 |
| HT29_4_GR_ChDB_0033 | SN38 | 3uM | 6h | MBNL1 | 0.00 | 0.24 |
| HT29_4_GR_ChDB_0033 | SN38 | 3uM | 6h | LDLR | 0.00 | 0.37 |
| HT29_4_GR_ChDB_0033 | SN38 | 3uM | 6h | HMGCS1 | 0.00 | 0.28 |
| HT29_4_GR_ChDB_0033 | SN38 | 3uM | 6h | SOCS5 | 0.00 | 0.36 |
| HT29_4_GR_ChDB_0033 | SN38 | 3uM | 6h | ATG5 | 0.00 | 0.47 |
| HT29_4_GR_ChDB_0033 | SN38 | 3uM | 6h | RAD17 | 0.00 | 0.34 |
| HT29_4_GR_ChDB_0033 | SN38 | 3uM | 6h | TOPBP1 | 0.00 | 0.26 |
| HT29_4_GR_ChDB_0033 | SN38 | 3uM | 6h | INADL | 0.00 | 0.45 |
| HT29_4_GR_ChDB_0033 | SN38 | 3uM | 6h | KAZN | 0.00 | 0.26 |
| HT29_4_GR_ChDB_0033 | SN38 | 3uM | 6h | PHIP | 0.00 | 0.24 |
| HT29_4_GR_ChDB_0033 | SN38 | 3uM | 6h | MTUS1 | 0.00 | 0.34 |
| HT29_4_GR_ChDB_0033 | SN38 | 3uM | 6h | CREM | 0.00 | 2.35 |
| HT29_4_GR_ChDB_0033 | SN38 | 3uM | 6h | SAT1 | 0.00 | 5.14 |
| HT29_4_GR_ChDB_0033 | SN38 | 3uM | 6h | ZBTB43 | 0.00 | 2.61 |
| HT29_4_GR_ChDB_0033 | SN38 | 3uM | 6h | TMEM222 | 0.00 | 2.23 |
| HT29_4_GR_ChDB_0033 | SN38 | 3uM | 6h | TRAPPC6A | 0.00 | 2.45 |
| HT29_4_GR_ChDB_0033 | SN38 | 3uM | 6h | C9orf16 | 0.00 | 2.48 |
| HT29_4_GR_ChDB_0033 | SN38 | 3uM | 6h | TSPYL2 | 0.00 | 3.23 |
| HT29_4_GR_ChDB_0033 | SN38 | 3uM | 6h | CD2BP2 | 0.00 | 2.21 |
| HT29_4_GR_ChDB_0033 | SN38 | 3uM | 6h | SKIV2L | 0.00 | 2.08 |
| HT29_4_GR_ChDB_0033 | SN38 | 3uM | 6h | NCAPH | 0.00 | 0.15 |
| HT29_4_GR_ChDB_0033 | SN38 | 3uM | 6h | ZMYM2 | 0.00 | 0.19 |
| HT29_4_GR_ChDB_0033 | SN38 | 3uM | 6h | RABGAP1 | 0.00 | 0.23 |
| HT29_4_GR_ChDB_0033 | SN38 | 3uM | 6h | ASXL1 | 0.00 | 0.47 |
| HT29_4_GR_ChDB_0033 | SN38 | 3uM | 6h | EXPH5 | 0.00 | 0.42 |
| HT29_4_GR_ChDB_0033 | SN38 | 3uM | 6h | WDR26 | 0.00 | 0.23 |
| HT29_4_GR_ChDB_0033 | SN38 | 3uM | 6h | LSM14A | 0.00 | 0.40 |
| HT29_4_GR_ChDB_0033 | SN38 | 3uM | 6h | PCM1 | 0.00 | 0.37 |
| HT29_4_GR_ChDB_0033 | SN38 | 3uM | 6h | SYNJ2 | 0.00 | 0.36 |
| HT29_4_GR_ChDB_0033 | SN38 | 3uM | 6h | FBXO28 | 0.00 | 0.38 |
| HT29_4_GR_ChDB_0033 | SN38 | 3uM | 6h | EP300 | 0.00 | 0.40 |
| HT29_4_GR_ChDB_0033 | SN38 | 3uM | 6h | PHF3 | 0.00 | 0.29 |
| HT29_4_GR_ChDB_0033 | SN38 | 3uM | 6h | GOLGA4 | 0.00 | 0.34 |
| HT29_4_GR_ChDB_0033 | SN38 | 3uM | 6h | UBE2B | 0.00 | 0.29 |
| HT29_4_GR_ChDB_0033 | SN38 | 3uM | 6h | GCC2 | 0.00 | 0.45 |
| HT29_4_GR_ChDB_0033 | SN38 | 3uM | 6h | ORC4 | 0.00 | 0.33 |
| HT29_4_GR_ChDB_0033 | SN38 | 3uM | 6h | BUB3 | 0.00 | 0.48 |
| HT29_4_GR_ChDB_0033 | SN38 | 3uM | 6h | ACSL3 | 0.00 | 0.23 |
| HT29_4_GR_ChDB_0033 | SN38 | 3uM | 6h | KRIT1 | 0.00 | 0.21 |
| HT29_4_GR_ChDB_0033 | SN38 | 3uM | 6h | SUZ12 | 0.00 | 0.42 |
| HT29_4_GR_ChDB_0033 | SN38 | 3uM | 6h | DLG1 | 0.00 | 0.34 |
| HT29_4_GR_ChDB_0033 | SN38 | 3uM | 6h | SS18 | 0.00 | 0.43 |
| HT29_4_GR_ChDB_0033 | SN38 | 3uM | 6h | PPP2R1B | 0.00 | 0.41 |
| HT29_4_GR_ChDB_0033 | SN38 | 3uM | 6h | MAP4K5 | 0.00 | 0.41 |
| HT29_4_GR_ChDB_0033 | SN38 | 3uM | 6h | WBP4 | 0.00 | 0.42 |
| HT29_4_GR_ChDB_0033 | SN38 | 3uM | 6h | RANBP2 | 0.00 | 0.33 |
| HT29_4_GR_ChDB_0033 | SN38 | 3uM | 6h | CCDC93 | 0.00 | 0.42 |
| HT29_4_GR_ChDB_0033 | SN38 | 3uM | 6h | COBLL1 | 0.00 | 0.25 |
| HT29_4_GR_ChDB_0033 | SN38 | 3uM | 6h | PDE3B | 0.00 | 0.33 |
| HT29_4_GR_ChDB_0033 | SN38 | 3uM | 6h | STX6 | 0.00 | 0.38 |
| HT29_4_GR_ChDB_0033 | SN38 | 3uM | 6h | ROCK1 | 0.00 | 0.40 |
| HT29_4_GR_ChDB_0033 | SN38 | 3uM | 6h | ETV6 | 0.00 | 0.38 |
| HT29_4_GR_ChDB_0033 | SN38 | 3uM | 6h | SLC19A1 | 0.00 | 0.31 |
| HT29_4_GR_ChDB_0033 | SN38 | 3uM | 6h | GSE1 | 0.00 | 0.36 |
| HT29_4_GR_ChDB_0033 | SN38 | 3uM | 6h | TIA1 | 0.00 | 0.39 |
| HT29_4_GR_ChDB_0033 | SN38 | 3uM | 6h | RBM39 | 0.00 | 0.19 |
| HT29_4_GR_ChDB_0033 | SN38 | 3uM | 6h | TGFBRAP1 | 0.00 | 0.41 |
| HT29_4_GR_ChDB_0033 | SN38 | 3uM | 6h | NUP98 | 0.00 | 0.29 |
| HT29_4_GR_ChDB_0033 | SN38 | 3uM | 6h | PWP1 | 0.00 | 0.41 |
| HT29_4_GR_ChDB_0033 | SN38 | 3uM | 6h | MINA | 0.00 | 0.40 |
| HT29_4_GR_ChDB_0033 | SN38 | 3uM | 6h | PAK2 | 0.00 | 0.43 |
| HT29_4_GR_ChDB_0033 | SN38 | 3uM | 6h | ITGB1BP1 | 0.00 | 0.34 |
| HT29_4_GR_ChDB_0033 | SN38 | 3uM | 6h | FMR1 | 0.00 | 0.34 |
| HT29_4_GR_ChDB_0033 | SN38 | 3uM | 6h | CDK13 | 0.00 | 0.29 |
| HT29_4_GR_ChDB_0033 | SN38 | 3uM | 6h | RFC1 | 0.00 | 0.28 |
| HT29_4_GR_ChDB_0033 | SN38 | 3uM | 6h | DLG3 | 0.00 | 0.34 |
| HT29_4_GR_ChDB_0033 | SN38 | 3uM | 6h | TLK2 | 0.00 | 0.33 |
| HT29_4_GR_ChDB_0033 | SN38 | 3uM | 6h | EIF3A | 0.00 | 0.40 |
| HT29_4_GR_ChDB_0033 | SN38 | 3uM | 6h | RBFOX2 | 0.00 | 0.43 |
| HT29_4_GR_ChDB_0033 | SN38 | 3uM | 6h | LARP7 | 0.00 | 0.43 |
| HT29_4_GR_ChDB_0033 | SN38 | 3uM | 6h | DIAPH2 | 0.00 | 0.42 |
| HT29_4_GR_ChDB_0033 | SN38 | 3uM | 6h | STAG1 | 0.00 | 0.34 |
| HT29_4_GR_ChDB_0033 | SN38 | 3uM | 6h | USP24 | 0.00 | 0.15 |
| HT29_4_GR_ChDB_0033 | SN38 | 3uM | 6h | ZDHHC17 | 0.00 | 0.28 |
| HT29_4_GR_ChDB_0033 | SN38 | 3uM | 6h | ZBED5 | 0.00 | 0.19 |
| HT29_4_GR_ChDB_0033 | SN38 | 3uM | 6h | MED8 | 0.00 | 0.32 |
| HT29_4_GR_ChDB_0033 | SN38 | 3uM | 6h | SUPT20H | 0.00 | 0.34 |
| HT29_4_GR_ChDB_0033 | SN38 | 3uM | 6h | PUM2 | 0.00 | 0.14 |
| HT29_4_GR_ChDB_0033 | SN38 | 3uM | 6h | NEIL3 | 0.00 | 0.42 |
| HT29_4_GR_ChDB_0033 | SN38 | 3uM | 6h | NUFIP1 | 0.00 | 0.34 |
| HT29_4_GR_ChDB_0033 | SN38 | 3uM | 6h | CTDSPL | 0.00 | 0.32 |
| HT29_4_GR_ChDB_0033 | SN38 | 3uM | 6h | IER2 | 0.00 | 2.62 |
| HT29_4_GR_ChDB_0033 | SN38 | 3uM | 6h | BAD | 0.00 | 2.58 |
| HT29_4_GR_ChDB_0033 | SN38 | 3uM | 6h | NFE2L2 | 0.00 | 0.35 |
| HT29_4_GR_ChDB_0033 | SN38 | 3uM | 6h | POT1 | 0.00 | 0.48 |
| HT29_4_GR_ChDB_0033 | SN38 | 3uM | 6h | PRKCH | 0.00 | 0.42 |
| HT29_4_GR_ChDB_0033 | SN38 | 3uM | 6h | ABI2 | 0.00 | 0.35 |
| HT29_4_GR_ChDB_0033 | SN38 | 3uM | 6h | UBE2J1 | 0.00 | 0.39 |
| HT29_4_GR_ChDB_0033 | SN38 | 3uM | 6h | PRKAG2 | 0.00 | 0.47 |
| HT29_4_GR_ChDB_0033 | SN38 | 3uM | 6h | ATF7IP | 0.00 | 0.41 |
| HT29_4_GR_ChDB_0033 | SN38 | 3uM | 6h | GTPBP4 | 0.00 | 0.42 |
| HT29_4_GR_ChDB_0033 | SN38 | 3uM | 6h | FNDC3A | 0.00 | 0.27 |
| HT29_4_GR_ChDB_0033 | SN38 | 3uM | 6h | FASTKD2 | 0.00 | 0.35 |
| HT29_4_GR_ChDB_0033 | SN38 | 3uM | 6h | RNGTT | 0.00 | 0.38 |
| HT29_4_GR_ChDB_0033 | SN38 | 3uM | 6h | ZHX2 | 0.00 | 0.16 |
| HT29_4_GR_ChDB_0033 | SN38 | 3uM | 6h | CLASP2 | 0.00 | 0.29 |
| HT29_4_GR_ChDB_0033 | SN38 | 3uM | 6h | RAD51C | 0.00 | 0.48 |
| HT29_4_GR_ChDB_0033 | SN38 | 3uM | 6h | HK2 | 0.00 | 0.29 |
| HT29_4_GR_ChDB_0033 | SN38 | 3uM | 6h | MEIS2 | 0.00 | 0.15 |
| HT29_4_GR_ChDB_0033 | SN38 | 3uM | 6h | OSBPL2 | 0.00 | 0.48 |
| HT29_4_GR_ChDB_0033 | SN38 | 3uM | 6h | DOCK9 | 0.00 | 0.33 |
| HT29_4_GR_ChDB_0033 | SN38 | 3uM | 6h | GRB10 | 0.00 | 0.31 |
| HT29_4_GR_ChDB_0033 | SN38 | 3uM | 6h | AMBRA1 | 0.00 | 0.44 |
| HT29_4_GR_ChDB_0033 | SN38 | 3uM | 6h | ZCCHC14 | 0.00 | 0.34 |
| HT29_4_GR_ChDB_0033 | SN38 | 3uM | 6h | MACF1 | 0.00 | 0.49 |
| HT29_4_GR_ChDB_0033 | SN38 | 3uM | 6h | HNRNPA0 | 0.00 | 2.27 |
| HT29_4_GR_ChDB_0033 | SN38 | 3uM | 6h | DHRS1 | 0.00 | 2.14 |
| HT29_4_GR_ChDB_0033 | SN38 | 3uM | 6h | COPZ1 | 0.00 | 2.01 |
| HT29_4_GR_ChDB_0033 | SN38 | 3uM | 6h | ABCF2 | 0.00 | 5.90 |
| HT29_4_GR_ChDB_0033 | SN38 | 3uM | 6h | SDF2 | 0.00 | 2.30 |
| HT29_4_GR_ChDB_0033 | SN38 | 3uM | 6h | STX4 | 0.00 | 2.16 |
| HT29_4_GR_ChDB_0033 | SN38 | 3uM | 6h | ATG12 | 0.00 | 8.35 |
| HT29_4_GR_ChDB_0033 | SN38 | 3uM | 6h | RPL15 | 0.00 | 2.20 |
| HT29_4_GR_ChDB_0033 | SN38 | 3uM | 6h | CDK16 | 0.00 | 2.06 |
| HT29_4_GR_ChDB_0033 | SN38 | 3uM | 6h | CTSL1 | 0.00 | 2.25 |
| HT29_4_GR_ChDB_0033 | SN38 | 3uM | 6h | SCAND1 | 0.00 | 2.55 |
| HT29_4_GR_ChDB_0033 | SN38 | 3uM | 6h | RHBDD3 | 0.00 | 2.07 |
| HT29_4_GR_ChDB_0033 | SN38 | 3uM | 6h | TGOLN2 | 0.00 | 3.32 |
| HT29_4_GR_ChDB_0033 | SN38 | 3uM | 6h | EIF1 | 0.00 | 22.66 |
| HT29_4_GR_ChDB_0033 | SN38 | 3uM | 6h | ADO | 0.00 | 3.54 |
| HT29_4_GR_ChDB_0033 | SN38 | 3uM | 6h | RNF113A | 0.00 | 2.72 |
| HT29_4_GR_ChDB_0033 | SN38 | 3uM | 6h | GART | 0.00 | 2.55 |
| HT29_4_GR_ChDB_0033 | SN38 | 3uM | 6h | KAT6A | 0.00 | 0.32 |
| HT29_4_GR_ChDB_0033 | SN38 | 3uM | 6h | KIF14 | 0.00 | 0.21 |
| HT29_4_GR_ChDB_0033 | SN38 | 3uM | 6h | KIF20A | 0.00 | 0.43 |
| HT29_4_GR_ChDB_0033 | SN38 | 3uM | 6h | PPFIA1 | 0.00 | 0.32 |
| HT29_4_GR_ChDB_0033 | SN38 | 3uM | 6h | USP46 | 0.00 | 0.49 |
| HT29_4_GR_ChDB_0033 | SN38 | 3uM | 6h | CUX1 | 0.00 | 0.42 |
| HT29_4_GR_ChDB_0033 | SN38 | 3uM | 6h | LEPROT | 0.00 | 0.25 |
| HT29_4_GR_ChDB_0033 | SN38 | 3uM | 6h | TJP1 | 0.00 | 0.35 |
| HT29_4_GR_ChDB_0033 | SN38 | 3uM | 6h | RANBP9 | 0.00 | 0.24 |
| HT29_4_GR_ChDB_0033 | SN38 | 3uM | 6h | SCAF8 | 0.00 | 0.20 |
| HT29_4_GR_ChDB_0033 | SN38 | 3uM | 6h | GPSM2 | 0.00 | 0.27 |
| HT29_4_GR_ChDB_0033 | SN38 | 3uM | 6h | SEC23IP | 0.00 | 0.35 |
| HT29_4_GR_ChDB_0033 | SN38 | 3uM | 6h | KIAA0368 | 0.00 | 0.48 |
| HT29_4_GR_ChDB_0033 | SN38 | 3uM | 6h | EPS15 | 0.00 | 0.27 |
| HT29_4_GR_ChDB_0033 | SN38 | 3uM | 6h | WHSC1 | 0.00 | 0.44 |
| HT29_4_GR_ChDB_0033 | SN38 | 3uM | 6h | WIPF2 | 0.00 | 0.45 |
| HT29_4_GR_ChDB_0033 | SN38 | 3uM | 6h | VGLL4 | 0.00 | 0.43 |
| HT29_4_GR_ChDB_0033 | SN38 | 3uM | 6h | PTPRK | 0.00 | 0.15 |
| HT29_4_GR_ChDB_0033 | SN38 | 3uM | 6h | NKTR | 0.00 | 0.41 |
| HT29_4_GR_ChDB_0033 | SN38 | 3uM | 6h | CHTOP | 0.00 | 0.43 |
| HT29_4_GR_ChDB_0033 | SN38 | 3uM | 6h | HIPK1 | 0.00 | 0.42 |
| HT29_4_GR_ChDB_0033 | SN38 | 3uM | 6h | YLPM1 | 0.00 | 0.35 |
| HT29_4_GR_ChDB_0033 | SN38 | 3uM | 6h | CHD9 | 0.00 | 0.39 |
| HT29_4_GR_ChDB_0033 | SN38 | 3uM | 6h | STRN3 | 0.00 | 0.12 |
| HT29_4_GR_ChDB_0033 | SN38 | 3uM | 6h | MAGI1 | 0.00 | 0.43 |
| HT29_4_GR_ChDB_0033 | SN38 | 3uM | 6h | PPM1B | 0.00 | 0.34 |
| HT29_4_GR_ChDB_0033 | SN38 | 3uM | 6h | PRPF4B | 0.00 | 0.26 |
| HT29_4_GR_ChDB_0033 | SN38 | 3uM | 6h | FOXO1 | 0.00 | 0.27 |
| HT29_4_GR_ChDB_0033 | SN38 | 3uM | 6h | CNOT4 | 0.00 | 0.44 |
| HT29_4_GR_ChDB_0033 | SN38 | 3uM | 6h | FZD5 | 0.00 | 0.49 |
| HT29_4_GR_ChDB_0033 | SN38 | 3uM | 6h | KIF2A | 0.00 | 0.12 |
| HT29_4_GR_ChDB_0033 | SN38 | 3uM | 6h | LGR5 | 0.00 | 0.09 |
| HT29_4_GR_ChDB_0033 | SN38 | 3uM | 6h | GEMIN2 | 0.00 | 0.41 |
| HT29_4_GR_ChDB_0033 | SN38 | 3uM | 6h | MVB12B | 0.00 | 0.45 |
| HT29_4_GR_ChDB_0033 | SN38 | 3uM | 6h | SAP30 | 0.00 | 0.42 |
| HT29_4_GR_ChDB_0033 | SN38 | 3uM | 6h | YEATS2 | 0.00 | 0.29 |
| HT29_4_GR_ChDB_0033 | SN38 | 3uM | 6h | TLE1 | 0.00 | 0.25 |
| HT29_4_GR_ChDB_0033 | SN38 | 3uM | 6h | KPNA1 | 0.00 | 0.42 |
| HT29_4_GR_ChDB_0033 | SN38 | 3uM | 6h | ACAP2 | 0.00 | 0.18 |
| HT29_4_GR_ChDB_0033 | SN38 | 3uM | 6h | EIF4E | 0.00 | 0.32 |
| HT29_4_GR_ChDB_0033 | SN38 | 3uM | 6h | EED | 0.00 | 0.35 |
| HT29_4_GR_ChDB_0033 | SN38 | 3uM | 6h | UBTF | 0.00 | 0.49 |
| HT29_4_GR_ChDB_0033 | SN38 | 3uM | 6h | OSBPL10 | 0.00 | 0.21 |
| HT29_4_GR_ChDB_0033 | SN38 | 3uM | 6h | RAD54B | 0.00 | 0.20 |
| HT29_4_GR_ChDB_0033 | SN38 | 3uM | 6h | NOL3 | 0.00 | 0.38 |
| HT29_4_GR_ChDB_0033 | SN38 | 3uM | 6h | SPDEF | 0.00 | 0.48 |
| HT29_4_GR_ChDB_0033 | SN38 | 3uM | 6h | TTK | 0.00 | 0.16 |
| HT29_4_GR_ChDB_0033 | SN38 | 3uM | 6h | NCAPG | 0.00 | 0.48 |
| HT29_4_GR_ChDB_0033 | SN38 | 3uM | 6h | DEPDC1 | 0.00 | 0.42 |
| HT29_4_GR_ChDB_0033 | SN38 | 3uM | 6h | MPZL1 | 0.00 | 0.49 |
| HT29_4_GR_ChDB_0033 | SN38 | 3uM | 6h | PUM1 | 0.00 | 0.22 |
| HT29_4_GR_ChDB_0033 | SN38 | 3uM | 6h | PPP1R12A | 0.00 | 0.17 |
| HT29_4_GR_ChDB_0033 | SN38 | 3uM | 6h | EPB41L1 | 0.00 | 0.43 |
| HT29_4_GR_ChDB_0033 | SN38 | 3uM | 6h | ZNF609 | 0.00 | 0.43 |
| HT29_4_GR_ChDB_0033 | SN38 | 3uM | 6h | APC | 0.00 | 0.29 |
| HT29_4_GR_ChDB_0033 | SN38 | 3uM | 6h | IVNS1ABP | 0.00 | 0.34 |
| HT29_4_GR_ChDB_0033 | SN38 | 3uM | 6h | LIMK2 | 0.00 | 0.31 |
| HT29_4_GR_ChDB_0033 | SN38 | 3uM | 6h | DENND2D | 0.00 | 0.46 |
| HT29_4_GR_ChDB_0033 | SN38 | 3uM | 6h | SRC | 0.00 | 0.49 |
| HT29_4_GR_ChDB_0033 | SN38 | 3uM | 6h | SOX4 | 0.00 | 0.43 |
| HT29_4_GR_ChDB_0033 | SN38 | 3uM | 6h | NRIP1 | 0.00 | 0.18 |
| HT29_4_GR_ChDB_0033 | SN38 | 3uM | 6h | ZC3H14 | 0.00 | 0.37 |
| HT29_4_GR_ChDB_0033 | SN38 | 3uM | 6h | BPTF | 0.00 | 0.32 |
| HT29_4_GR_ChDB_0033 | SN38 | 3uM | 6h | FAM208A | 0.00 | 0.21 |
| HT29_4_GR_ChDB_0033 | SN38 | 3uM | 6h | DR1 | 0.00 | 0.43 |
| HT29_4_GR_ChDB_0033 | SN38 | 3uM | 6h | RAD1 | 0.00 | 0.35 |
| HT29_4_GR_ChDB_0033 | SN38 | 3uM | 6h | DICER1 | 0.00 | 0.44 |
| HT29_4_GR_ChDB_0033 | SN38 | 3uM | 6h | TRIM24 | 0.00 | 0.27 |
| HT29_4_GR_ChDB_0033 | SN38 | 3uM | 6h | TNFRSF21 | 0.00 | 0.26 |
| HT29_4_GR_ChDB_0033 | SN38 | 3uM | 6h | HOXC4 | 0.00 | 0.48 |
| HT29_4_GR_ChDB_0033 | SN38 | 3uM | 6h | EDEM3 | 0.00 | 0.50 |
| HT29_4_GR_ChDB_0033 | SN38 | 3uM | 6h | BARD1 | 0.00 | 0.28 |
| HT29_4_GR_ChDB_0033 | SN38 | 3uM | 6h | DAAM1 | 0.00 | 0.47 |
| HT29_4_GR_ChDB_0033 | SN38 | 3uM | 6h | FAM193A | 0.00 | 0.45 |
| HT29_4_GR_ChDB_0033 | SN38 | 3uM | 6h | ANGEL2 | 0.00 | 0.35 |
| HT29_4_GR_ChDB_0033 | SN38 | 3uM | 6h | NBN | 0.00 | 0.48 |
| HT29_4_GR_ChDB_0033 | SN38 | 3uM | 6h | TANK | 0.00 | 0.25 |
| HT29_4_GR_ChDB_0033 | SN38 | 3uM | 6h | SWAP70 | 0.00 | 0.38 |
| HT29_4_GR_ChDB_0033 | SN38 | 3uM | 6h | SPRED2 | 0.00 | 0.18 |
| HT29_4_GR_ChDB_0033 | SN38 | 3uM | 6h | MFN1 | 0.00 | 0.46 |
| HT29_4_GR_ChDB_0033 | SN38 | 3uM | 6h | MTX2 | 0.00 | 0.36 |
| HT29_4_GR_ChDB_0033 | SN38 | 3uM | 6h | PDHX | 0.00 | 0.44 |
| HT29_4_GR_ChDB_0033 | SN38 | 3uM | 6h | RIF1 | 0.00 | 0.23 |
| HT29_4_GR_ChDB_0033 | SN38 | 3uM | 6h | GCDH | 0.00 | 2.25 |
| HT29_4_GR_ChDB_0033 | SN38 | 3uM | 6h | DOHH | 0.00 | 2.02 |
| HT29_4_GR_ChDB_0033 | SN38 | 3uM | 6h | SLC9A1 | 0.00 | 2.15 |
| HT29_4_GR_ChDB_0033 | SN38 | 3uM | 6h | HAUS5 | 0.00 | 2.08 |
| HT29_4_GR_ChDB_0033 | SN38 | 3uM | 6h | ZNF428 | 0.00 | 2.43 |
| HT29_4_GR_ChDB_0033 | SN38 | 3uM | 6h | C19orf60 | 0.00 | 2.22 |
| HT29_4_GR_ChDB_0033 | SN38 | 3uM | 6h | RRP7A | 0.00 | 3.25 |
| HT29_4_GR_ChDB_0033 | SN38 | 3uM | 6h | CAMLG | 0.00 | 2.45 |
| HT29_4_GR_ChDB_0033 | SN38 | 3uM | 6h | HCCS | 0.00 | 2.00 |
| HT29_4_GR_ChDB_0033 | SN38 | 3uM | 6h | FASTKD5 | 0.00 | 2.14 |
| HT29_4_GR_ChDB_0033 | SN38 | 3uM | 6h | ZCCHC10 | 0.00 | 2.25 |
| HT29_4_GR_ChDB_0033 | SN38 | 3uM | 6h | RPS28 | 0.00 | 2.03 |
| HT29_4_GR_ChDB_0033 | SN38 | 3uM | 6h | PRKCSH | 0.00 | 2.03 |
| HT29_4_GR_ChDB_0033 | SN38 | 3uM | 6h | GLUL | 0.00 | 2.36 |
| HT29_4_GR_ChDB_0033 | SN38 | 3uM | 6h | AAMDC | 0.00 | 2.10 |
| HT29_4_GR_ChDB_0033 | SN38 | 3uM | 6h | CSPG5 | 0.00 | 2.06 |
| HT29_4_GR_ChDB_0033 | SN38 | 3uM | 6h | DUSP5 | 0.00 | 2.39 |
| HT29_4_GR_ChDB_0033 | SN38 | 3uM | 6h | WDR55 | 0.00 | 2.08 |
| HT29_4_GR_ChDB_0033 | SN38 | 3uM | 6h | CASKIN2 | 0.00 | 2.00 |
| HT29_4_GR_ChDB_0033 | SN38 | 3uM | 6h | BRD2 | 0.00 | 2.96 |
| HT29_4_GR_ChDB_0033 | SN38 | 3uM | 6h | PIGT | 0.00 | 2.03 |
| HT29_4_GR_ChDB_0033 | SN38 | 3uM | 6h | AKAP17A | 0.00 | 2.25 |
| HT29_4_GR_ChDB_0033 | SN38 | 3uM | 6h | STRN | 0.00 | 0.23 |
| HT29_4_GR_ChDB_0033 | SN38 | 3uM | 6h | LAMB1 | 0.00 | 0.38 |
| HT29_4_GR_ChDB_0033 | SN38 | 3uM | 6h | LIMCH1 | 0.00 | 0.45 |
| HT29_4_GR_ChDB_0033 | SN38 | 3uM | 6h | DTX4 | 0.00 | 0.42 |
| HT29_4_GR_ChDB_0033 | SN38 | 3uM | 6h | PIK3R4 | 0.00 | 0.23 |
| HT29_4_GR_ChDB_0033 | SN38 | 3uM | 6h | ELMO2 | 0.00 | 0.49 |
| HT29_4_GR_ChDB_0033 | SN38 | 3uM | 6h | BRD7 | 0.00 | 0.43 |
| HT29_4_GR_ChDB_0033 | SN38 | 3uM | 6h | TARDBP | 0.00 | 0.50 |
| HT29_4_GR_ChDB_0033 | SN38 | 3uM | 6h | ARIH1 | 0.00 | 0.35 |
| HT29_4_GR_ChDB_0033 | SN38 | 3uM | 6h | NSMCE4A | 0.00 | 0.36 |
| HT29_4_GR_ChDB_0033 | SN38 | 3uM | 6h | STX12 | 0.00 | 0.48 |
| HT29_4_GR_ChDB_0033 | SN38 | 3uM | 6h | APPBP2 | 0.00 | 0.44 |
| HT29_4_GR_ChDB_0033 | SN38 | 3uM | 6h | FASTKD1 | 0.00 | 0.35 |
| HT29_4_GR_ChDB_0033 | SN38 | 3uM | 6h | MAT2A | 0.00 | 0.38 |
| HT29_4_GR_ChDB_0033 | SN38 | 3uM | 6h | TAF9 | 0.00 | 0.39 |
| HT29_4_GR_ChDB_0033 | SN38 | 3uM | 6h | HS3ST1 | 0.00 | 0.37 |
| HT29_4_GR_ChDB_0033 | SN38 | 3uM | 6h | ZNF45 | 0.00 | 0.46 |
| HT29_4_GR_ChDB_0033 | SN38 | 3uM | 6h | ANAPC10 | 0.00 | 0.36 |
| HT29_4_GR_ChDB_0033 | SN38 | 3uM | 6h | RBMS1 | 0.00 | 0.25 |
| HT29_4_GR_ChDB_0033 | SN38 | 3uM | 6h | DST | 0.00 | 0.43 |
| HT29_4_GR_ChDB_0033 | SN38 | 3uM | 6h | UTP18 | 0.00 | 0.46 |
| HT29_4_GR_ChDB_0033 | SN38 | 3uM | 6h | ENSA | 0.00 | 0.26 |
| HT29_4_GR_ChDB_0033 | SN38 | 3uM | 6h | PPP2R5E | 0.00 | 0.35 |
| HT29_4_GR_ChDB_0033 | SN38 | 3uM | 6h | PRRC2B | 0.00 | 0.45 |
| HT29_4_GR_ChDB_0033 | SN38 | 3uM | 6h | LARP4 | 0.00 | 0.38 |
| HT29_4_GR_ChDB_0033 | SN38 | 3uM | 6h | C18orf25 | 0.00 | 0.38 |
| HT29_4_GR_ChDB_0033 | SN38 | 3uM | 6h | CNOT2 | 0.00 | 0.15 |
| HT29_4_GR_ChDB_0033 | SN38 | 3uM | 6h | CUL3 | 0.00 | 0.27 |
| HT29_4_GR_ChDB_0033 | SN38 | 3uM | 6h | SSX2IP | 0.00 | 0.42 |
| HT29_4_GR_ChDB_0033 | SN38 | 3uM | 6h | SLC25A12 | 0.00 | 0.39 |
| HT29_4_GR_ChDB_0033 | SN38 | 3uM | 6h | EBAG9 | 0.00 | 0.45 |
| HT29_4_GR_ChDB_0033 | SN38 | 3uM | 6h | BRCA1 | 0.00 | 0.26 |
| HT29_4_GR_ChDB_0033 | SN38 | 3uM | 6h | USP10 | 0.00 | 0.27 |
| HT29_4_GR_ChDB_0033 | SN38 | 3uM | 6h | BTAF1 | 0.00 | 0.24 |
| HT29_4_GR_ChDB_0033 | SN38 | 3uM | 6h | ZNF638 | 0.00 | 0.35 |
| HT29_4_GR_ChDB_0033 | SN38 | 3uM | 6h | WAPAL | 0.00 | 0.25 |
| HT29_4_GR_ChDB_0033 | SN38 | 3uM | 6h | C2CD2 | 0.00 | 0.41 |
| HT29_4_GR_ChDB_0033 | SN38 | 3uM | 6h | TXLNG | 0.00 | 0.44 |
| HT29_4_GR_ChDB_0033 | SN38 | 3uM | 6h | DDX3X | 0.00 | 0.41 |
| HT29_4_GR_ChDB_0033 | SN38 | 3uM | 6h | RAD50 | 0.00 | 0.39 |
| HT29_4_GR_ChDB_0033 | SN38 | 3uM | 6h | CENPI | 0.00 | 0.47 |
| HT29_4_GR_ChDB_0033 | SN38 | 3uM | 6h | SLCO3A1 | 0.00 | 0.36 |
| HT29_4_GR_ChDB_0033 | SN38 | 3uM | 6h | TGS1 | 0.00 | 0.50 |
| HT29_4_GR_ChDB_0033 | SN38 | 3uM | 6h | PARD3 | 0.00 | 0.29 |
| HT29_4_GR_ChDB_0033 | SN38 | 3uM | 6h | FXR1 | 0.00 | 0.39 |
| HT29_4_GR_ChDB_0033 | SN38 | 3uM | 6h | RABEP1 | 0.00 | 0.31 |
| HT29_4_GR_ChDB_0033 | SN38 | 3uM | 6h | CDC40 | 0.00 | 0.47 |
| HT29_4_GR_ChDB_0033 | SN38 | 3uM | 6h | PPFIBP1 | 0.00 | 0.18 |
| HT29_4_GR_ChDB_0033 | SN38 | 3uM | 6h | SLC38A1 | 0.00 | 0.32 |
| HT29_4_GR_ChDB_0033 | SN38 | 3uM | 6h | MAPKAPK5 | 0.00 | 0.33 |
| HT29_4_GR_ChDB_0033 | SN38 | 3uM | 6h | BAZ1B | 0.00 | 0.43 |
| HT29_4_GR_ChDB_0033 | SN38 | 3uM | 6h | SLK | 0.00 | 0.34 |
| HT29_4_GR_ChDB_0033 | SN38 | 3uM | 6h | HIPK3 | 0.00 | 0.48 |
| HT29_4_GR_ChDB_0033 | SN38 | 3uM | 6h | MICAL2 | 0.00 | 0.50 |
| HT29_4_GR_ChDB_0033 | SN38 | 3uM | 6h | SLC23A2 | 0.00 | 0.47 |
| HT29_4_GR_ChDB_0033 | SN38 | 3uM | 6h | NRF1 | 0.00 | 0.45 |
| HT29_4_GR_ChDB_0033 | SN38 | 3uM | 6h | USP32 | 0.00 | 0.21 |
| HT29_4_GR_ChDB_0033 | SN38 | 3uM | 6h | DYRK1A | 0.00 | 0.14 |
| HT29_4_GR_ChDB_0033 | SN38 | 3uM | 6h | SLC5A3 | 0.00 | 0.42 |
| HT29_4_GR_ChDB_0033 | SN38 | 3uM | 6h | ARFGEF2 | 0.00 | 0.50 |
| HT29_4_GR_ChDB_0033 | SN38 | 3uM | 6h | TMEM131 | 0.00 | 0.45 |
| HT29_4_GR_ChDB_0033 | SN38 | 3uM | 6h | IGF1R | 0.00 | 0.22 |
| HT29_4_GR_ChDB_0033 | SN38 | 3uM | 6h | CEP57 | 0.00 | 0.44 |
| HT29_4_GR_ChDB_0033 | SN38 | 3uM | 6h | RER1 | 0.00 | 2.36 |
| HT29_4_GR_ChDB_0033 | SN38 | 3uM | 6h | CEP104 | 0.00 | 2.29 |
| HT29_4_GR_ChDB_0033 | SN38 | 3uM | 6h | LAMP2 | 0.00 | 2.10 |
| HT29_4_GR_ChDB_0033 | SN38 | 3uM | 6h | WDR83OS | 0.00 | 2.21 |
| HT29_4_GR_ChDB_0033 | SN38 | 3uM | 6h | SIVA1 | 0.00 | 2.25 |
| HT29_4_GR_ChDB_0033 | SN38 | 3uM | 6h | RSL1D1 | 0.00 | 6.19 |
| HT29_4_GR_ChDB_0033 | SN38 | 3uM | 6h | ZNF419 | 0.00 | 2.71 |
| HT29_4_GR_ChDB_0033 | SN38 | 3uM | 6h | BBS1 | 0.00 | 2.36 |
| HT29_4_GR_ChDB_0033 | SN38 | 3uM | 6h | YIF1A | 0.00 | 2.06 |
| HT29_4_GR_ChDB_0033 | SN38 | 3uM | 6h | KLF4 | 0.00 | 2.22 |
| HT29_4_GR_ChDB_0033 | SN38 | 3uM | 6h | HMGCL | 0.00 | 2.47 |
| HT29_4_GR_ChDB_0033 | SN38 | 3uM | 6h | MAP1LC3B | 0.00 | 2.73 |
| HT29_4_GR_ChDB_0033 | SN38 | 3uM | 6h | SAP18 | 0.00 | 3.25 |
| HT29_4_GR_ChDB_0033 | SN38 | 3uM | 6h | CLN5 | 0.00 | 2.24 |
| HT29_4_GR_ChDB_0033 | SN38 | 3uM | 6h | DLX2 | 0.00 | 2.45 |
| HT29_4_GR_ChDB_0033 | SN38 | 3uM | 6h | BCL2L11 | 0.00 | 2.17 |
| HT29_4_GR_ChDB_0033 | SN38 | 3uM | 6h | ZNF580 | 0.00 | 4.62 |
| HT29_4_GR_ChDB_0033 | SN38 | 3uM | 6h | ZNF280B | 0.00 | 2.00 |
| HT29_4_GR_ChDB_0033 | SN38 | 3uM | 6h | MAPKAPK5-AS1 | 0.00 | 2.98 |
| HT29_4_GR_ChDB_0033 | SN38 | 3uM | 6h | CHAF1A | 0.00 | 0.45 |
| HT29_4_GR_ChDB_0033 | SN38 | 3uM | 6h | ADORA2B | 0.00 | 0.45 |
| HT29_4_GR_ChDB_0033 | SN38 | 3uM | 6h | RBPJ | 0.00 | 0.29 |
| HT29_4_GR_ChDB_0033 | SN38 | 3uM | 6h | CEP350 | 0.00 | 0.24 |
| HT29_4_GR_ChDB_0033 | SN38 | 3uM | 6h | UBAP2 | 0.00 | 0.44 |
| HT29_4_GR_ChDB_0033 | SN38 | 3uM | 6h | SETD5 | 0.00 | 0.26 |
| HT29_4_GR_ChDB_0033 | SN38 | 3uM | 6h | ZCCHC11 | 0.00 | 0.42 |
| HT29_4_GR_ChDB_0033 | SN38 | 3uM | 6h | TEX10 | 0.00 | 0.27 |
| HT29_4_GR_ChDB_0033 | SN38 | 3uM | 6h | CTCF | 0.00 | 0.39 |
| HT29_4_GR_ChDB_0033 | SN38 | 3uM | 6h | TAF5 | 0.00 | 0.34 |
| HT29_4_GR_ChDB_0033 | SN38 | 3uM | 6h | PIAS1 | 0.00 | 0.32 |
| HT29_4_GR_ChDB_0033 | SN38 | 3uM | 6h | CREBBP | 0.00 | 0.20 |
| HT29_4_GR_ChDB_0033 | SN38 | 3uM | 6h | ANK3 | 0.00 | 0.35 |
| HT29_4_GR_ChDB_0033 | SN38 | 3uM | 6h | QRSL1 | 0.00 | 0.28 |
| HT29_4_GR_ChDB_0033 | SN38 | 3uM | 6h | TRAM2 | 0.00 | 0.45 |
| HT29_4_GR_ChDB_0033 | SN38 | 3uM | 6h | NBPF1 | 0.00 | 0.36 |
| HT29_4_GR_ChDB_0033 | SN38 | 3uM | 6h | FEZ2 | 0.00 | 0.36 |
| HT29_4_GR_ChDB_0033 | SN38 | 3uM | 6h | KIAA0020 | 0.00 | 0.35 |
| HT29_4_GR_ChDB_0033 | SN38 | 3uM | 6h | ABI1 | 0.00 | 0.44 |
| HT29_4_GR_ChDB_0033 | SN38 | 3uM | 6h | TRIM33 | 0.00 | 0.19 |
| HT29_4_GR_ChDB_0033 | SN38 | 3uM | 6h | TMF1 | 0.00 | 0.38 |
| HT29_4_GR_ChDB_0033 | SN38 | 3uM | 6h | DNAJC2 | 0.00 | 0.34 |
| HT29_4_GR_ChDB_0033 | SN38 | 3uM | 6h | ATP1B1 | 0.00 | 0.40 |
| HT29_4_GR_ChDB_0033 | SN38 | 3uM | 6h | AGAP1 | 0.00 | 0.30 |
| HT29_4_GR_ChDB_0033 | SN38 | 3uM | 6h | BMPR1A | 0.00 | 0.23 |
| HT29_4_GR_ChDB_0033 | SN38 | 3uM | 6h | CDK17 | 0.00 | 0.24 |
| HT29_4_GR_ChDB_0033 | SN38 | 3uM | 6h | BICD2 | 0.00 | 0.40 |
| HT29_4_GR_ChDB_0033 | SN38 | 3uM | 6h | ATP13A3 | 0.00 | 0.41 |
| HT29_4_GR_ChDB_0033 | SN38 | 3uM | 6h | SON | 0.00 | 0.41 |
| HT29_4_GR_ChDB_0033 | SN38 | 3uM | 6h | ARFGEF1 | 0.00 | 0.30 |
| HT29_4_GR_ChDB_0033 | SN38 | 3uM | 6h | GOSR1 | 0.00 | 0.36 |
| HT29_4_GR_ChDB_0033 | SN38 | 3uM | 6h | CEP135 | 0.00 | 0.41 |
| HT29_4_GR_ChDB_0033 | SN38 | 3uM | 6h | SGMS1 | 0.00 | 0.26 |
| HT29_4_GR_ChDB_0033 | SN38 | 3uM | 6h | PTBP3 | 0.00 | 0.40 |
| HT29_4_GR_ChDB_0033 | SN38 | 3uM | 6h | CDC27 | 0.00 | 0.45 |
| HT29_4_GR_ChDB_0033 | SN38 | 3uM | 6h | NARG2 | 0.00 | 0.46 |
| HT29_4_GR_ChDB_0033 | SN38 | 3uM | 6h | SFSWAP | 0.00 | 0.35 |
| HT29_4_GR_ChDB_0033 | SN38 | 3uM | 6h | INPP4A | 0.00 | 0.46 |
| HT29_4_GR_ChDB_0033 | SN38 | 3uM | 6h | KIF15 | 0.00 | 0.35 |
| HT29_4_GR_ChDB_0033 | SN38 | 3uM | 6h | WAC | 0.00 | 0.35 |
| HT29_4_GR_ChDB_0033 | SN38 | 3uM | 6h | WDR59 | 0.00 | 0.43 |
| HT29_4_GR_ChDB_0033 | SN38 | 3uM | 6h | ADIPOR2 | 0.00 | 0.45 |
| HT29_4_GR_ChDB_0033 | SN38 | 3uM | 6h | RRP1B | 0.00 | 0.48 |
| HT29_4_GR_ChDB_0033 | SN38 | 3uM | 6h | FOXK2 | 0.00 | 0.32 |
| HT29_4_GR_ChDB_0033 | SN38 | 3uM | 6h | SMAD2 | 0.00 | 0.42 |
| HT29_4_GR_ChDB_0033 | SN38 | 3uM | 6h | PTPN2 | 0.00 | 0.44 |
| HT29_4_GR_ChDB_0033 | SN38 | 3uM | 6h | DENND5A | 0.00 | 0.32 |
| HT29_4_GR_ChDB_0033 | SN38 | 3uM | 6h | MGEA5 | 0.00 | 0.37 |
| HT29_4_GR_ChDB_0033 | SN38 | 3uM | 6h | ZNF587B | 0.00 | 0.23 |
| HT29_4_GR_ChDB_0033 | SN38 | 3uM | 6h | SYNRG | 0.00 | 0.47 |
| HT29_4_GR_ChDB_0033 | SN38 | 3uM | 6h | THAP4 | 0.00 | 0.49 |
| HT29_4_GR_ChDB_0033 | SN38 | 3uM | 6h | SMAD1 | 0.00 | 0.35 |
| HT29_4_GR_ChDB_0033 | SN38 | 3uM | 6h | CRLF3 | 0.00 | 0.36 |
| HT29_4_GR_ChDB_0033 | SN38 | 3uM | 6h | PNN | 0.00 | 0.19 |
| HT29_4_GR_ChDB_0033 | SN38 | 3uM | 6h | SART3 | 0.00 | 0.34 |
| HT29_4_GR_ChDB_0033 | SN38 | 3uM | 6h | SLC7A1 | 0.00 | 0.40 |
| HT29_4_GR_ChDB_0033 | SN38 | 3uM | 6h | ZFX | 0.00 | 0.45 |
| HT29_4_GR_ChDB_0033 | SN38 | 3uM | 6h | ANKMY2 | 0.00 | 0.45 |
| HT29_4_GR_ChDB_0033 | SN38 | 3uM | 6h | AHCTF1 | 0.00 | 0.21 |
| HT29_4_GR_ChDB_0033 | SN38 | 3uM | 6h | SKA1 | 0.00 | 0.39 |
| HT29_4_GR_ChDB_0033 | SN38 | 3uM | 6h | ARHGAP19 | 0.00 | 0.46 |
| HT29_4_GR_ChDB_0033 | SN38 | 3uM | 6h | XRCC4 | 0.00 | 0.33 |
| HT29_4_GR_ChDB_0033 | SN38 | 3uM | 6h | KAT6B | 0.00 | 0.16 |
| HT29_4_GR_ChDB_0033 | SN38 | 3uM | 6h | TAF1B | 0.00 | 0.27 |
| HT29_4_GR_ChDB_0033 | SN38 | 3uM | 6h | MTDH | 0.00 | 0.36 |
| HT29_4_GR_ChDB_0033 | SN38 | 3uM | 6h | SRBD1 | 0.00 | 0.47 |
| HT29_4_GR_ChDB_0033 | SN38 | 3uM | 6h | HEATR3 | 0.00 | 0.45 |
| HT29_4_GR_ChDB_0033 | SN38 | 3uM | 6h | ZC3H4 | 0.00 | 0.27 |
| HT29_4_GR_ChDB_0033 | SN38 | 3uM | 6h | SNTB2 | 0.00 | 0.48 |
| HT29_4_GR_ChDB_0033 | SN38 | 3uM | 6h | KLC1 | 0.00 | 0.20 |
| HT29_4_GR_ChDB_0033 | SN38 | 3uM | 6h | TSEN2 | 0.00 | 0.30 |
| HT29_4_GR_ChDB_0033 | SN38 | 3uM | 6h | KNTC1 | 0.00 | 0.45 |
| HT29_4_GR_ChDB_0033 | SN38 | 3uM | 6h | AZIN1 | 0.00 | 0.47 |
| HT29_4_GR_ChDB_0033 | SN38 | 3uM | 6h | USP33 | 0.00 | 0.45 |
| HT29_4_GR_ChDB_0033 | SN38 | 3uM | 6h | USP16 | 0.00 | 0.47 |
| HT29_4_GR_ChDB_0033 | SN38 | 3uM | 6h | RAB28 | 0.00 | 0.37 |
| HT29_4_GR_ChDB_0033 | SN38 | 3uM | 6h | TNFRSF1A | 0.00 | 0.29 |
| HT29_4_GR_ChDB_0033 | SN38 | 3uM | 6h | TLK1 | 0.00 | 0.26 |
| HT29_4_GR_ChDB_0033 | SN38 | 3uM | 6h | MED14 | 0.00 | 0.35 |
| HT29_4_GR_ChDB_0033 | SN38 | 3uM | 6h | FLNB | 0.00 | 0.46 |
| HT29_4_GR_ChDB_0033 | SN38 | 3uM | 6h | CAB39 | 0.00 | 0.27 |
| HT29_4_GR_ChDB_0033 | SN38 | 3uM | 6h | MTPAP | 0.00 | 0.48 |
| HT29_4_GR_ChDB_0033 | SN38 | 3uM | 6h | ENOX2 | 0.00 | 0.43 |
| HT29_4_GR_ChDB_0033 | SN38 | 3uM | 6h | CCDC41 | 0.00 | 0.38 |
| HT29_4_GR_ChDB_0033 | SN38 | 3uM | 6h | CCNL1 | 0.00 | 0.40 |
| HT29_4_GR_ChDB_0033 | SN38 | 3uM | 6h | VPS54 | 0.00 | 0.28 |
| HT29_4_GR_ChDB_0033 | SN38 | 3uM | 6h | PAGR1 | 0.00 | 0.42 |
| HT29_4_GR_ChDB_0033 | SN38 | 3uM | 6h | NFAT5 | 0.00 | 0.32 |
| HT29_4_GR_ChDB_0033 | SN38 | 3uM | 6h | NEMF | 0.00 | 0.31 |
| HT29_4_GR_ChDB_0033 | SN38 | 3uM | 6h | METTL3 | 0.00 | 0.47 |
| HT29_4_GR_ChDB_0033 | SN38 | 3uM | 6h | NCOR1 | 0.00 | 0.39 |
| HT29_4_GR_ChDB_0033 | SN38 | 3uM | 6h | KLHL20 | 0.00 | 0.39 |
| HT29_4_GR_ChDB_0033 | SN38 | 3uM | 6h | ATXN7 | 0.00 | 0.21 |
| HT29_4_GR_ChDB_0033 | SN38 | 3uM | 6h | HJURP | 0.00 | 0.43 |
| HT29_4_GR_ChDB_0033 | SN38 | 3uM | 6h | NASP | 0.00 | 0.41 |
| HT29_4_GR_ChDB_0033 | SN38 | 3uM | 6h | ARHGEF7 | 0.00 | 0.13 |
| HT29_4_GR_ChDB_0033 | SN38 | 3uM | 6h | CELF1 | 0.00 | 0.33 |
| HT29_4_GR_ChDB_0033 | SN38 | 3uM | 6h | USP48 | 0.00 | 0.33 |
| HT29_4_GR_ChDB_0033 | SN38 | 3uM | 6h | MAP4K4 | 0.00 | 0.50 |
| HT29_4_GR_ChDB_0033 | SN38 | 3uM | 6h | MED27 | 0.00 | 0.37 |
| HT29_4_GR_ChDB_0033 | SN38 | 3uM | 6h | ZNF324 | 0.00 | 2.81 |
| HT29_4_GR_ChDB_0033 | SN38 | 3uM | 6h | H2AFX | 0.00 | 2.34 |
| HT29_4_GR_ChDB_0033 | SN38 | 3uM | 6h | LIN37 | 0.00 | 2.44 |
| HT29_4_GR_ChDB_0033 | SN38 | 3uM | 6h | MRPS12 | 0.00 | 2.20 |
| HT29_4_GR_ChDB_0033 | SN38 | 3uM | 6h | DNPH1 | 0.00 | 2.21 |
| HT29_4_GR_ChDB_0033 | SN38 | 3uM | 6h | TMEM160 | 0.00 | 2.08 |
| HT29_4_GR_ChDB_0033 | SN38 | 3uM | 6h | OGFOD2 | 0.00 | 2.05 |
| HT29_4_GR_ChDB_0033 | SN38 | 3uM | 6h | ASB6 | 0.00 | 2.05 |
| HT29_4_GR_ChDB_0033 | SN38 | 3uM | 6h | MSL3 | 0.00 | 2.10 |
| HT29_4_GR_ChDB_0033 | SN38 | 3uM | 6h | BIRC5 | 0.00 | 2.56 |
| HT29_4_GR_ChDB_0033 | SN38 | 3uM | 6h | FEM1C | 0.00 | 2.00 |
| HT29_4_GR_ChDB_0033 | SN38 | 3uM | 6h | SIGMAR1 | 0.00 | 2.56 |
| HT29_4_GR_ChDB_0033 | SN38 | 3uM | 6h | NDUFAF4 | 0.00 | 3.20 |
| HT29_4_GR_ChDB_0033 | SN38 | 3uM | 6h | PI3 | 0.00 | 2.32 |
| HT29_4_GR_ChDB_0033 | SN38 | 3uM | 6h | CCS | 0.00 | 2.17 |
| HT29_4_GR_ChDB_0033 | SN38 | 3uM | 6h | PMAIP1 | 0.00 | 9.83 |
| HT29_4_GR_ChDB_0033 | SN38 | 3uM | 6h | PCYOX1L | 0.00 | 4.44 |
| HT29_4_GR_ChDB_0033 | SN38 | 3uM | 6h | CTSL2 | 0.00 | 2.02 |
| HT29_4_GR_ChDB_0033 | SN38 | 3uM | 6h | MFAP3 | 0.00 | 3.36 |
| HT29_4_GR_ChDB_0033 | SN38 | 3uM | 6h | BTG1 | 0.00 | 2.58 |
| HT29_4_GR_ChDB_0033 | SN38 | 3uM | 6h | BPGM | 0.00 | 2.19 |
| HT29_4_GR_ChDB_0033 | SN38 | 3uM | 6h | FOSL1 | 0.00 | 2.30 |
| HT29_4_GR_ChDB_0033 | SN38 | 3uM | 6h | NMB | 0.00 | 2.14 |
| HT29_4_GR_ChDB_0033 | SN38 | 3uM | 6h | RHOB | 0.00 | 4.08 |
| HT29_4_GR_ChDB_0033 | SN38 | 3uM | 6h | NAT1 | 0.00 | 4.72 |
| HT29_4_GR_ChDB_0033 | SN38 | 3uM | 6h | NT5DC2 | 0.00 | 2.08 |
| HT29_4_GR_ChDB_0033 | SN38 | 3uM | 6h | ZNF408 | 0.00 | 2.11 |
| HT29_4_GR_ChDB_0033 | SN38 | 3uM | 6h | BCCIP | 0.00 | 2.13 |
| HT29_4_GR_ChDB_0033 | SN38 | 3uM | 6h | PFKL | 0.00 | 2.27 |
| HT29_4_GR_ChDB_0033 | SN38 | 3uM | 6h | CAPN1 | 0.00 | 2.55 |
| HT29_4_GR_ChDB_0033 | SN38 | 3uM | 6h | DGAT1 | 0.00 | 2.11 |
| HT29_4_GR_ChDB_0033 | SN38 | 3uM | 6h | OSER1 | 0.00 | 2.37 |
| HT29_4_GR_ChDB_0033 | SN38 | 3uM | 6h | CLDN15 | 0.00 | 2.30 |
| HT29_4_GR_ChDB_0033 | SN38 | 3uM | 6h | PSMC3IP | 0.00 | 2.17 |
| HT29_4_GR_ChDB_0033 | SN38 | 3uM | 6h | ASB1 | 0.00 | 2.08 |
| HT29_4_GR_ChDB_0033 | SN38 | 3uM | 6h | PABPN1 | 0.00 | 4.80 |
| HT29_4_GR_ChDB_0033 | SN38 | 3uM | 6h | SCML1 | 0.00 | 3.81 |
| HT29_4_GR_ChDB_0033 | SN38 | 3uM | 6h | KRCC1 | 0.00 | 2.47 |
| HT29_4_GR_ChDB_0033 | SN38 | 3uM | 6h | TUSC2 | 0.00 | 2.57 |
| HT29_4_GR_ChDB_0033 | SN38 | 3uM | 6h | ARPC5L | 0.00 | 2.73 |
| HT29_4_GR_ChDB_0033 | SN38 | 3uM | 6h | TOB1 | 0.00 | 2.61 |
| HT29_4_GR_ChDB_0033 | SN38 | 3uM | 6h | BCL10 | 0.00 | 2.22 |
| HT29_4_GR_ChDB_0033 | SN38 | 3uM | 6h | PUS3 | 0.00 | 2.13 |
| HT29_4_GR_ChDB_0033 | SN38 | 3uM | 6h | CYR61 | 0.00 | 7.76 |
| HT29_4_GR_ChDB_0033 | SN38 | 3uM | 6h | UBE2D2 | 0.00 | 0.39 |
| HT29_4_GR_ChDB_0033 | SN38 | 3uM | 6h | DAPK1 | 0.00 | 0.24 |
| HT29_4_GR_ChDB_0033 | SN38 | 3uM | 6h | EEA1 | 0.00 | 0.25 |
| HT29_4_GR_ChDB_0033 | SN38 | 3uM | 6h | NR2C2 | 0.00 | 0.35 |
| HT29_4_GR_ChDB_0033 | SN38 | 3uM | 6h | CBL | 0.00 | 0.45 |
| HT29_4_GR_ChDB_0033 | SN38 | 3uM | 6h | PRKAA1 | 0.00 | 0.49 |
| HT29_4_GR_ChDB_0033 | SN38 | 3uM | 6h | TAB2 | 0.00 | 0.39 |
| HT29_4_GR_ChDB_0033 | SN38 | 3uM | 6h | SP1 | 0.00 | 0.45 |
| HT29_4_GR_ChDB_0033 | SN38 | 3uM | 6h | SAV1 | 0.00 | 0.37 |
| HT29_4_GR_ChDB_0033 | SN38 | 3uM | 6h | ASH1L | 0.00 | 0.27 |
| HT29_4_GR_ChDB_0033 | SN38 | 3uM | 6h | TRMT11 | 0.00 | 0.49 |
| HT29_4_GR_ChDB_0033 | SN38 | 3uM | 6h | AFF4 | 0.00 | 0.26 |
| HT29_4_GR_ChDB_0033 | SN38 | 3uM | 6h | FAM198B | 0.00 | 0.36 |
| HT29_4_GR_ChDB_0033 | SN38 | 3uM | 6h | GPHN | 0.00 | 0.38 |
| HT29_4_GR_ChDB_0033 | SN38 | 3uM | 6h | B4GALT5 | 0.00 | 0.35 |
| HT29_4_GR_ChDB_0033 | SN38 | 3uM | 6h | RALGAPB | 0.00 | 0.42 |
| HT29_4_GR_ChDB_0033 | SN38 | 3uM | 6h | N4BP2L2 | 0.00 | 0.33 |
| HT29_4_GR_ChDB_0033 | SN38 | 3uM | 6h | TACC2 | 0.00 | 0.19 |
| HT29_4_GR_ChDB_0033 | SN38 | 3uM | 6h | AKAP11 | 0.00 | 0.44 |
| HT29_4_GR_ChDB_0033 | SN38 | 3uM | 6h | PMM2 | 0.00 | 0.18 |
| HT29_4_GR_ChDB_0033 | SN38 | 3uM | 6h | TFCP2 | 0.00 | 0.37 |
| HT29_4_GR_ChDB_0033 | SN38 | 3uM | 6h | RBPMS | 0.00 | 0.39 |
| HT29_4_GR_ChDB_0033 | SN38 | 3uM | 6h | KIAA1199 | 0.00 | 0.15 |
| HT29_4_GR_ChDB_0033 | SN38 | 3uM | 6h | SNX13 | 0.00 | 0.42 |
| HT29_4_GR_ChDB_0033 | SN38 | 3uM | 6h | POGK | 0.00 | 0.43 |
| HT29_4_GR_ChDB_0033 | SN38 | 3uM | 6h | UBA2 | 0.00 | 0.40 |
| HT29_4_GR_ChDB_0033 | SN38 | 3uM | 6h | AMIGO2 | 0.00 | 0.43 |
| HT29_4_GR_ChDB_0033 | SN38 | 3uM | 6h | ARL4C | 0.00 | 0.46 |
| HT29_4_GR_ChDB_0033 | SN38 | 3uM | 6h | TCERG1 | 0.00 | 0.44 |
| HT29_4_GR_ChDB_0033 | SN38 | 3uM | 6h | CUL5 | 0.00 | 0.40 |
| HT29_4_GR_ChDB_0033 | SN38 | 3uM | 6h | TGFA | 0.00 | 0.41 |
| HT29_4_GR_ChDB_0033 | SN38 | 3uM | 6h | HOXB6 | 0.00 | 0.32 |
| HT29_4_GR_ChDB_0033 | SN38 | 3uM | 6h | TAF1A | 0.00 | 0.47 |
| HT29_4_GR_ChDB_0033 | SN38 | 3uM | 6h | PHF15 | 0.00 | 0.25 |
| HT29_4_GR_ChDB_0033 | SN38 | 3uM | 6h | SLC1A4 | 0.00 | 0.44 |
| HT29_4_GR_ChDB_0033 | SN38 | 3uM | 6h | SPC25 | 0.00 | 0.49 |
| HT29_4_GR_ChDB_0033 | SN38 | 3uM | 6h | KITLG | 0.00 | 0.12 |
| HT29_4_GR_ChDB_0033 | SN38 | 3uM | 6h | RBM12 | 0.00 | 0.40 |
| HT29_4_GR_ChDB_0033 | SN38 | 3uM | 6h | YAP1 | 0.00 | 0.38 |
| HT29_4_GR_ChDB_0033 | SN38 | 3uM | 6h | DNA2 | 0.00 | 0.29 |
| HT29_4_GR_ChDB_0033 | SN38 | 3uM | 6h | CEP152 | 0.00 | 0.31 |
| HT29_4_GR_ChDB_0033 | SN38 | 3uM | 6h | USP53 | 0.00 | 0.45 |
| HT29_4_GR_ChDB_0033 | SN38 | 3uM | 6h | MTAP | 0.00 | 0.35 |
| HT29_4_GR_ChDB_0033 | SN38 | 3uM | 6h | FANCL | 0.00 | 0.28 |
| HT29_4_GR_ChDB_0033 | SN38 | 3uM | 6h | DTWD1 | 0.00 | 0.46 |
| HT29_4_GR_ChDB_0033 | SN38 | 3uM | 6h | PRDM10 | 0.00 | 0.38 |
| HT29_4_GR_ChDB_0033 | SN38 | 3uM | 6h | STEAP4 | 0.00 | 0.47 |
| HT29_4_GR_ChDB_0033 | SN38 | 3uM | 6h | INTS7 | 0.00 | 0.35 |
| HT29_4_GR_ChDB_0033 | SN38 | 3uM | 6h | BLZF1 | 0.00 | 0.48 |
| HT29_4_GR_ChDB_0033 | SN38 | 3uM | 6h | MED13 | 0.00 | 0.23 |
| HT29_4_GR_ChDB_0033 | SN38 | 3uM | 6h | VEZF1 | 0.00 | 0.40 |
| HT29_4_GR_ChDB_0033 | SN38 | 3uM | 6h | GCLC | 0.00 | 0.28 |
| HT29_4_GR_ChDB_0033 | SN38 | 3uM | 6h | FBXO11 | 0.00 | 0.16 |
| HT29_4_GR_ChDB_0033 | SN38 | 3uM | 6h | MED1 | 0.00 | 0.46 |
| HT29_4_GR_ChDB_0033 | SN38 | 3uM | 6h | RAB11FIP2 | 0.00 | 0.39 |
| HT29_4_GR_ChDB_0033 | SN38 | 3uM | 6h | VPRBP | 0.00 | 0.29 |
| HT29_4_GR_ChDB_0033 | SN38 | 3uM | 6h | ING3 | 0.00 | 0.39 |
| HT29_4_GR_ChDB_0033 | SN38 | 3uM | 6h | BMP4 | 0.00 | 0.30 |
| HT29_4_GR_ChDB_0033 | SN38 | 3uM | 6h | WWP1 | 0.00 | 0.14 |
| HT29_4_GR_ChDB_0033 | SN38 | 3uM | 6h | SP3 | 0.00 | 0.34 |
| HT29_4_GR_ChDB_0033 | SN38 | 3uM | 6h | EPHA1 | 0.00 | 0.43 |
| HT29_4_GR_ChDB_0033 | SN38 | 3uM | 6h | GOLPH3 | 0.00 | 0.18 |
| HT29_4_GR_ChDB_0033 | SN38 | 3uM | 6h | DCLRE1C | 0.00 | 0.48 |
| HT29_4_GR_ChDB_0033 | SN38 | 3uM | 6h | FAM60A | 0.00 | 0.32 |
| HT29_4_GR_ChDB_0033 | SN38 | 3uM | 6h | TRIP12 | 0.00 | 0.40 |
| HT29_4_GR_ChDB_0033 | SN38 | 3uM | 6h | CD55 | 0.00 | 0.40 |
| HT29_4_GR_ChDB_0033 | SN38 | 3uM | 6h | SETX | 0.00 | 0.24 |
| HT29_4_GR_ChDB_0033 | SN38 | 3uM | 6h | SP100 | 0.00 | 0.43 |
| HT29_4_GR_ChDB_0033 | SN38 | 3uM | 6h | IGF2BP3 | 0.00 | 0.32 |
| HT29_4_GR_ChDB_0033 | SN38 | 3uM | 6h | PDS5B | 0.00 | 0.21 |
| HT29_4_GR_ChDB_0033 | SN38 | 3uM | 6h | AIM1 | 0.00 | 0.45 |
| HT29_4_GR_ChDB_0033 | SN38 | 3uM | 6h | SLTM | 0.00 | 0.31 |
| HT29_4_GR_ChDB_0033 | SN38 | 3uM | 6h | ERBB2IP | 0.00 | 0.20 |
| HT29_4_GR_ChDB_0033 | SN38 | 3uM | 6h | ARL5A | 0.00 | 0.42 |
| HT29_4_GR_ChDB_0033 | SN38 | 3uM | 6h | ZNF544 | 0.00 | 0.44 |
| HT29_4_GR_ChDB_0033 | SN38 | 3uM | 6h | KLHL12 | 0.00 | 0.41 |
| HT29_4_GR_ChDB_0033 | SN38 | 3uM | 6h | ASAP1 | 0.00 | 0.21 |
| HT29_4_GR_ChDB_0033 | SN38 | 3uM | 6h | SLC4A7 | 0.00 | 0.15 |
| HT29_4_GR_ChDB_0033 | SN38 | 3uM | 6h | AKAP9 | 0.00 | 0.25 |
| HT29_4_GR_ChDB_0033 | SN38 | 3uM | 6h | LNPEP | 0.00 | 0.43 |
| HT29_4_GR_ChDB_0033 | SN38 | 3uM | 6h | UBE2G1 | 0.00 | 0.31 |
| HT29_4_GR_ChDB_0033 | SN38 | 3uM | 6h | PKN2 | 0.00 | 0.21 |
| HT29_4_GR_ChDB_0033 | SN38 | 3uM | 6h | MYO1B | 0.00 | 0.40 |
| HT29_4_GR_ChDB_0033 | SN38 | 3uM | 6h | DOCK5 | 0.00 | 0.36 |
| HT29_4_GR_ChDB_0033 | SN38 | 3uM | 6h | MLLT4 | 0.00 | 0.29 |
| HT29_4_GR_ChDB_0033 | SN38 | 3uM | 6h | REPS1 | 0.00 | 0.22 |
| HT29_4_GR_ChDB_0033 | SN38 | 3uM | 6h | AGGF1 | 0.00 | 0.41 |
| HT29_4_GR_ChDB_0033 | SN38 | 3uM | 6h | ZNF407 | 0.00 | 0.42 |
| HT29_4_GR_ChDB_0033 | SN38 | 3uM | 6h | PSD3 | 0.00 | 0.38 |
| HT29_4_GR_ChDB_0033 | SN38 | 3uM | 6h | NFIC | 0.00 | 0.45 |
| HT29_4_GR_ChDB_0033 | SN38 | 3uM | 6h | PPIG | 0.00 | 0.27 |
| HT29_4_GR_ChDB_0033 | SN38 | 3uM | 6h | KIAA0947 | 0.00 | 0.37 |
| HT29_4_GR_ChDB_0033 | SN38 | 3uM | 6h | UHRF1BP1L | 0.00 | 0.41 |
| HT29_4_GR_ChDB_0033 | SN38 | 3uM | 6h | RHOT1 | 0.00 | 0.28 |
| HT29_4_GR_ChDB_0033 | SN38 | 3uM | 6h | HOXA10 | 0.00 | 0.31 |
| HT29_4_GR_ChDB_0033 | SN38 | 3uM | 6h | PVRL3 | 0.00 | 0.20 |
| HT29_4_GR_ChDB_0033 | SN38 | 3uM | 6h | MED4 | 0.00 | 0.31 |
| HT29_4_GR_ChDB_0033 | SN38 | 3uM | 6h | POLQ | 0.00 | 0.31 |
| HT29_4_GR_ChDB_0033 | SN38 | 3uM | 6h | PLEKHA5 | 0.00 | 0.11 |
| HT29_4_GR_ChDB_0033 | SN38 | 3uM | 6h | HNF4A | 0.00 | 0.34 |
| HT29_4_GR_ChDB_0033 | SN38 | 3uM | 6h | PHF2 | 0.00 | 0.48 |
| HT29_4_GR_ChDB_0033 | SN38 | 3uM | 6h | MAP3K5 | 0.00 | 0.41 |
| HT29_4_GR_ChDB_0033 | SN38 | 3uM | 6h | ZZZ3 | 0.00 | 0.32 |
| HT29_4_GR_ChDB_0033 | SN38 | 3uM | 6h | BBX | 0.00 | 0.35 |
| HT29_4_GR_ChDB_0033 | SN38 | 3uM | 6h | PDZD8 | 0.00 | 0.20 |
| HT29_4_GR_ChDB_0033 | SN38 | 3uM | 6h | TMEM165 | 0.00 | 0.25 |
| HT29_4_GR_ChDB_0033 | SN38 | 3uM | 6h | MLPH | 0.00 | 0.46 |
| HT29_4_GR_ChDB_0033 | SN38 | 3uM | 6h | CDC73 | 0.00 | 0.36 |
| HT29_4_GR_ChDB_0033 | SN38 | 3uM | 6h | RB1CC1 | 0.00 | 0.27 |
| HT29_4_GR_ChDB_0033 | SN38 | 3uM | 6h | ARHGAP35 | 0.00 | 0.13 |
| HT29_4_GR_ChDB_0033 | SN38 | 3uM | 6h | NUP153 | 0.00 | 0.15 |
| HT29_4_GR_ChDB_0033 | SN38 | 3uM | 6h | ARHGAP11A | 0.00 | 0.50 |
| HT29_4_GR_ChDB_0033 | SN38 | 3uM | 6h | STK4 | 0.00 | 0.40 |
| HT29_4_GR_ChDB_0033 | SN38 | 3uM | 6h | TRAF3 | 0.00 | 0.39 |
| HT29_4_GR_ChDB_0033 | SN38 | 3uM | 6h | SYNCRIP | 0.00 | 0.24 |
| HT29_4_GR_ChDB_0033 | SN38 | 3uM | 6h | UFL1 | 0.00 | 0.49 |
| HT29_4_GR_ChDB_0033 | SN38 | 3uM | 6h | WWC1 | 0.00 | 0.34 |
| HT29_4_GR_ChDB_0033 | SN38 | 3uM | 6h | KIF3A | 0.00 | 0.36 |
| HT29_4_GR_ChDB_0033 | SN38 | 3uM | 6h | GNE | 0.00 | 0.33 |
| HT29_4_GR_ChDB_0033 | SN38 | 3uM | 6h | PRRG1 | 0.00 | 0.28 |
| HT29_4_GR_ChDB_0033 | SN38 | 3uM | 6h | PIK3R1 | 0.00 | 0.15 |
| HT29_4_GR_ChDB_0033 | SN38 | 3uM | 6h | CUTC | 0.00 | 0.27 |
| HT29_4_GR_ChDB_0033 | SN38 | 3uM | 6h | AAK1 | 0.00 | 0.47 |
| HT29_4_GR_ChDB_0033 | SN38 | 3uM | 6h | CDC42EP4 | 0.00 | 0.49 |
| HT29_4_GR_ChDB_0033 | SN38 | 3uM | 6h | PDXK | 0.00 | 0.40 |
| HT29_4_GR_ChDB_0033 | SN38 | 3uM | 6h | PEX14 | 0.00 | 0.24 |
| HT29_4_GR_ChDB_0033 | SN38 | 3uM | 6h | C9orf114 | 0.00 | 0.44 |
| HT29_4_GR_ChDB_0033 | SN38 | 3uM | 6h | TASP1 | 0.00 | 0.33 |
| HT29_4_GR_ChDB_0033 | SN38 | 3uM | 6h | STX7 | 0.00 | 0.40 |
| HT29_4_GR_ChDB_0033 | SN38 | 3uM | 6h | DENND4C | 0.00 | 0.22 |
| HT29_4_GR_ChDB_0033 | SN38 | 3uM | 6h | POU2F1 | 0.00 | 0.45 |
| HT29_4_GR_ChDB_0033 | SN38 | 3uM | 6h | RAB40C | 0.00 | 0.27 |
| HT29_4_GR_ChDB_0033 | SN38 | 3uM | 6h | GON4L | 0.00 | 0.38 |
| HT29_4_GR_ChDB_0033 | SN38 | 3uM | 6h | LONRF3 | 0.00 | 0.45 |
| HT29_4_GR_ChDB_0033 | SN38 | 3uM | 6h | CDK5RAP2 | 0.00 | 0.40 |
| HT29_4_GR_ChDB_0033 | SN38 | 3uM | 6h | RNFT1 | 0.00 | 0.48 |
| HT29_4_GR_ChDB_0033 | SN38 | 3uM | 6h | FAM20B | 0.00 | 0.47 |
| HT29_4_GR_ChDB_0033 | SN38 | 3uM | 6h | SATB1 | 0.00 | 0.29 |
| HT29_4_GR_ChDB_0033 | SN38 | 3uM | 6h | METTL1 | 0.00 | 0.26 |
| HT29_4_GR_ChDB_0033 | SN38 | 3uM | 6h | REV3L | 0.00 | 0.26 |
| HT29_4_GR_ChDB_0033 | SN38 | 3uM | 6h | LMO4 | 0.00 | 0.29 |
| HT29_4_GR_ChDB_0033 | SN38 | 3uM | 6h | GIGYF2 | 0.00 | 0.38 |
| HT29_4_GR_ChDB_0033 | SN38 | 3uM | 6h | NRP1 | 0.00 | 0.43 |
| HT29_4_GR_ChDB_0033 | SN38 | 3uM | 6h | TRAPPC13 | 0.00 | 0.46 |
| HT29_4_GR_ChDB_0033 | SN38 | 3uM | 6h | PPP3R1 | 0.00 | 0.24 |
| HT29_4_GR_ChDB_0033 | SN38 | 3uM | 6h | MAX | 0.00 | 0.45 |
| HT29_4_GR_ChDB_0033 | SN38 | 3uM | 6h | CHMP2B | 0.00 | 0.47 |
| HT29_4_GR_ChDB_0033 | SN38 | 3uM | 6h | PRIM1 | 0.00 | 0.44 |
| HT29_4_GR_ChDB_0033 | SN38 | 3uM | 6h | EFNA5 | 0.00 | 0.33 |
| HT29_4_GR_ChDB_0033 | SN38 | 3uM | 6h | OSBPL3 | 0.00 | 0.41 |
| HT29_4_GR_ChDB_0033 | SN38 | 3uM | 6h | PTPRJ | 0.00 | 0.32 |
| HT29_4_GR_ChDB_0033 | SN38 | 3uM | 6h | SOS1 | 0.00 | 0.32 |
| HT29_4_GR_ChDB_0033 | SN38 | 3uM | 6h | FBXO9 | 0.00 | 0.49 |
| HT29_4_GR_ChDB_0033 | SN38 | 3uM | 6h | CAMSAP1 | 0.00 | 0.33 |
| HT29_4_GR_ChDB_0033 | SN38 | 3uM | 6h | TAOK3 | 0.00 | 0.49 |
| HT29_4_GR_ChDB_0033 | SN38 | 3uM | 6h | TRRAP | 0.00 | 0.42 |
| HT29_4_GR_ChDB_0033 | SN38 | 3uM | 6h | NOC3L | 0.00 | 0.24 |
| HT29_4_GR_ChDB_0033 | SN38 | 3uM | 6h | SEL1L | 0.00 | 0.43 |
| HT29_4_GR_ChDB_0033 | SN38 | 3uM | 6h | PIK3CB | 0.00 | 0.13 |
| HT29_4_GR_ChDB_0033 | SN38 | 3uM | 6h | CRYBG3 | 0.00 | 0.31 |
| HT29_4_GR_ChDB_0033 | SN38 | 3uM | 6h | KCMF1 | 0.00 | 0.35 |
| HT29_4_GR_ChDB_0033 | SN38 | 3uM | 6h | ADSS | 0.00 | 0.32 |
| HT29_4_GR_ChDB_0033 | SN38 | 3uM | 6h | ZFYVE16 | 0.00 | 0.28 |
| HT29_4_GR_ChDB_0033 | SN38 | 3uM | 6h | ARHGAP29 | 0.00 | 0.19 |
| HT29_4_GR_ChDB_0033 | SN38 | 3uM | 6h | RAP1GAP2 | 0.00 | 0.36 |
| HT29_4_GR_ChDB_0033 | SN38 | 3uM | 6h | WTAP | 0.00 | 0.27 |
| HT29_4_GR_ChDB_0033 | SN38 | 3uM | 6h | ARFIP1 | 0.00 | 0.39 |
| HT29_4_GR_ChDB_0033 | SN38 | 3uM | 6h | SEC24B | 0.00 | 0.19 |
| HT29_4_GR_ChDB_0033 | SN38 | 3uM | 6h | FOSB | 0.00 | 2.91 |
| HT29_4_GR_ChDB_0033 | SN38 | 3uM | 6h | AKR7A2 | 0.00 | 2.10 |
| HT29_4_GR_ChDB_0033 | SN38 | 3uM | 6h | ZNF250 | 0.00 | 2.07 |
| HT29_4_GR_ChDB_0033 | SN38 | 3uM | 6h | MAD2L1BP | 0.00 | 2.85 |
| HT29_4_GR_ChDB_0033 | SN38 | 3uM | 6h | LINS | 0.00 | 2.15 |
| HT29_4_GR_ChDB_0033 | SN38 | 3uM | 6h | NKIRAS2 | 0.00 | 2.11 |
| HT29_4_GR_ChDB_0033 | SN38 | 3uM | 6h | FSTL3 | 0.00 | 2.13 |
| HT29_4_GR_ChDB_0033 | SN38 | 3uM | 6h | IER5 | 0.00 | 3.43 |
| HT29_4_GR_ChDB_0033 | SN38 | 3uM | 6h | RNF25 | 0.00 | 2.17 |
| HT29_4_GR_ChDB_0033 | SN38 | 3uM | 6h | ULBP2 | 0.00 | 3.59 |
| HT29_4_GR_ChDB_0033 | SN38 | 3uM | 6h | IRX5 | 0.00 | 2.69 |
| HT29_4_GR_ChDB_0033 | SN38 | 3uM | 6h | ZKSCAN1 | 0.00 | 2.14 |
| HT29_4_GR_ChDB_0033 | SN38 | 3uM | 6h | HLA-G | 0.00 | 2.23 |
| HT29_4_GR_ChDB_0033 | SN38 | 3uM | 6h | SPATA2 | 0.00 | 3.15 |
| HT29_4_GR_ChDB_0033 | SN38 | 3uM | 6h | TMEM187 | 0.00 | 2.33 |
| HT29_4_GR_ChDB_0033 | SN38 | 3uM | 6h | ETV3 | 0.00 | 2.71 |
| HT29_4_GR_ChDB_0033 | SN38 | 3uM | 6h | EGFR | 0.00 | 0.40 |
| HT29_4_GR_ChDB_0033 | SN38 | 3uM | 6h | METTL8 | 0.00 | 0.45 |
| HT29_4_GR_ChDB_0033 | SN38 | 3uM | 6h | ITCH | 0.00 | 0.24 |
| HT29_4_GR_ChDB_0033 | SN38 | 3uM | 6h | CAPRIN1 | 0.00 | 0.41 |
| HT29_4_GR_ChDB_0033 | SN38 | 3uM | 6h | SPATS2 | 0.00 | 0.45 |
| HT29_4_GR_ChDB_0033 | SN38 | 3uM | 6h | H1F0 | 0.00 | 0.37 |
| HT29_4_GR_ChDB_0033 | SN38 | 3uM | 6h | SLC2A1 | 0.00 | 0.49 |
| HT29_4_GR_ChDB_0033 | SN38 | 3uM | 6h | STIL | 0.00 | 0.49 |
| HT29_4_GR_ChDB_0033 | SN38 | 3uM | 6h | MTRF1 | 0.00 | 0.49 |
| HT29_4_GR_ChDB_0033 | SN38 | 3uM | 6h | COPS2 | 0.00 | 0.47 |
| HT29_4_GR_ChDB_0033 | SN38 | 3uM | 6h | FRYL | 0.00 | 0.37 |
| HT29_4_GR_ChDB_0033 | SN38 | 3uM | 6h | PANK3 | 0.00 | 0.41 |
| HT29_4_GR_ChDB_0033 | SN38 | 3uM | 6h | DDX10 | 0.00 | 0.09 |
| HT29_4_GR_ChDB_0033 | SN38 | 3uM | 6h | WHSC1L1 | 0.00 | 0.37 |
| HT29_4_GR_ChDB_0033 | SN38 | 3uM | 6h | UBE2H | 0.00 | 0.41 |
| HT29_4_GR_ChDB_0033 | SN38 | 3uM | 6h | ATXN7L3B | 0.00 | 0.30 |
| HT29_4_GR_ChDB_0033 | SN38 | 3uM | 6h | GAPVD1 | 0.00 | 0.33 |
| HT29_4_GR_ChDB_0033 | SN38 | 3uM | 6h | CRY1 | 0.00 | 0.35 |
| HT29_4_GR_ChDB_0033 | SN38 | 3uM | 6h | SENP2 | 0.00 | 0.43 |
| HT29_4_GR_ChDB_0033 | SN38 | 3uM | 6h | TTF2 | 0.00 | 0.36 |
| HT29_4_GR_ChDB_0033 | SN38 | 3uM | 6h | NVL | 0.00 | 0.38 |
| HT29_4_GR_ChDB_0033 | SN38 | 3uM | 6h | HERC4 | 0.00 | 0.37 |
| HT29_4_GR_ChDB_0033 | SN38 | 3uM | 6h | KDM4C | 0.00 | 0.27 |
| HT29_4_GR_ChDB_0033 | SN38 | 3uM | 6h | KIAA1704 | 0.00 | 0.44 |
| HT29_4_GR_ChDB_0033 | SN38 | 3uM | 6h | NPAT | 0.00 | 0.23 |
| HT29_4_GR_ChDB_0033 | SN38 | 3uM | 6h | RBM25 | 0.00 | 0.36 |
| HT29_4_GR_ChDB_0033 | SN38 | 3uM | 6h | TJP2 | 0.00 | 0.39 |
| HT29_4_GR_ChDB_0033 | SN38 | 3uM | 6h | C1GALT1 | 0.00 | 0.35 |
| HT29_4_GR_ChDB_0033 | SN38 | 3uM | 6h | RYK | 0.00 | 0.43 |
| HT29_4_GR_ChDB_0033 | SN38 | 3uM | 6h | NAT10 | 0.00 | 0.41 |
| HT29_4_GR_ChDB_0033 | SN38 | 3uM | 6h | ZNF586 | 0.00 | 0.35 |
| HT29_4_GR_ChDB_0033 | SN38 | 3uM | 6h | KIF20B | 0.00 | 0.15 |
| HT29_4_GR_ChDB_0033 | SN38 | 3uM | 6h | NAA15 | 0.00 | 0.40 |
| HT29_4_GR_ChDB_0033 | SN38 | 3uM | 6h | ESRP1 | 0.00 | 0.41 |
| HT29_4_GR_ChDB_0033 | SN38 | 3uM | 6h | NUP160 | 0.00 | 0.43 |
| HT29_4_GR_ChDB_0033 | SN38 | 3uM | 6h | EFNB2 | 0.00 | 0.37 |
| HT29_4_GR_ChDB_0033 | SN38 | 3uM | 6h | SLC35A1 | 0.00 | 0.43 |
| HT29_4_GR_ChDB_0033 | SN38 | 3uM | 6h | B4GALT6 | 0.00 | 0.43 |
| HT29_4_GR_ChDB_0033 | SN38 | 3uM | 6h | TCF12 | 0.00 | 0.18 |
| HT29_4_GR_ChDB_0033 | SN38 | 3uM | 6h | ERICH1 | 0.00 | 0.36 |
| HT29_4_GR_ChDB_0033 | SN38 | 3uM | 6h | C10orf12 | 0.00 | 0.40 |
| HT29_4_GR_ChDB_0033 | SN38 | 3uM | 6h | SRPK2 | 0.00 | 0.22 |
| HT29_4_GR_ChDB_0033 | SN38 | 3uM | 6h | STXBP3 | 0.00 | 0.35 |
| HT29_4_GR_ChDB_0033 | SN38 | 3uM | 6h | PHLPP2 | 0.00 | 0.42 |
| HT29_4_GR_ChDB_0033 | SN38 | 3uM | 6h | ARHGAP17 | 0.00 | 0.45 |
| HT29_4_GR_ChDB_0033 | SN38 | 3uM | 6h | MSL2 | 0.00 | 0.47 |
| HT29_4_GR_ChDB_0033 | SN38 | 3uM | 6h | DNAAF2 | 0.00 | 0.50 |
| HT29_4_GR_ChDB_0033 | SN38 | 3uM | 6h | MYO10 | 0.00 | 0.28 |
| HT29_4_GR_ChDB_0033 | SN38 | 3uM | 6h | MBP | 0.00 | 0.29 |
| HT29_4_GR_ChDB_0033 | SN38 | 3uM | 6h | WWP2 | 0.00 | 0.29 |
| HT29_4_GR_ChDB_0033 | SN38 | 3uM | 6h | FOXJ3 | 0.00 | 0.12 |
| HT29_4_GR_ChDB_0033 | SN38 | 3uM | 6h | TMC5 | 0.00 | 0.24 |
| HT29_4_GR_ChDB_0033 | SN38 | 3uM | 6h | ELF2 | 0.00 | 0.41 |
| HT29_4_GR_ChDB_0033 | SN38 | 3uM | 6h | YTHDF3 | 0.00 | 0.49 |
| HT29_4_GR_ChDB_0033 | SN38 | 3uM | 6h | TAF1 | 0.00 | 0.25 |
| HT29_4_GR_ChDB_0033 | SN38 | 3uM | 6h | MLLT10 | 0.00 | 0.28 |
| HT29_4_GR_ChDB_0033 | SN38 | 3uM | 6h | SPAG9 | 0.00 | 0.37 |
| HT29_4_GR_ChDB_0033 | SN38 | 3uM | 6h | RASA1 | 0.00 | 0.16 |
| HT29_4_GR_ChDB_0033 | SN38 | 3uM | 6h | ZNF148 | 0.00 | 0.22 |
| HT29_4_GR_ChDB_0033 | SN38 | 3uM | 6h | SPAST | 0.00 | 0.30 |
| HT29_4_GR_ChDB_0033 | SN38 | 3uM | 6h | STAM2 | 0.00 | 0.39 |
| HT29_4_GR_ChDB_0033 | SN38 | 3uM | 6h | MORC3 | 0.00 | 0.49 |
| HT29_4_GR_ChDB_0033 | SN38 | 3uM | 6h | PRRC2A | 0.00 | 0.50 |
| HT29_4_GR_ChDB_0033 | SN38 | 3uM | 6h | NHLRC2 | 0.00 | 0.44 |
| HT29_4_GR_ChDB_0033 | SN38 | 3uM | 6h | SCAF11 | 0.00 | 0.46 |
| HT29_4_GR_ChDB_0033 | SN38 | 3uM | 6h | MARCH7 | 0.00 | 0.36 |
| HT29_4_GR_ChDB_0033 | SN38 | 3uM | 6h | VANGL1 | 0.00 | 0.23 |
| HT29_4_GR_ChDB_0033 | SN38 | 3uM | 6h | BCOR | 0.00 | 0.45 |
| HT29_4_GR_ChDB_0033 | SN38 | 3uM | 6h | TPBG | 0.00 | 2.05 |
| HT29_4_GR_ChDB_0033 | SN38 | 3uM | 6h | RPS6KB2 | 0.00 | 2.48 |
| HT29_4_GR_ChDB_0033 | SN38 | 3uM | 6h | TFAP2A | 0.00 | 2.12 |
| HT29_4_GR_ChDB_0033 | SN38 | 3uM | 6h | SLC10A3 | 0.00 | 2.17 |
| HT29_4_GR_ChDB_0033 | SN38 | 3uM | 6h | UBFD1 | 0.00 | 2.22 |
| HT29_4_GR_ChDB_0033 | SN38 | 3uM | 6h | CDKN2B | 0.00 | 15.12 |
| HT29_4_GR_ChDB_0033 | SN38 | 3uM | 6h | DEGS1 | 0.00 | 2.02 |
| HT29_4_GR_ChDB_0033 | SN38 | 3uM | 6h | POLDIP2 | 0.00 | 2.01 |
| HT29_4_GR_ChDB_0033 | SN38 | 3uM | 6h | ZNF574 | 0.00 | 3.98 |
| HT29_4_GR_ChDB_0033 | SN38 | 3uM | 6h | FBXL15 | 0.00 | 2.10 |
| HT29_4_GR_ChDB_0033 | SN38 | 3uM | 6h | C22orf29 | 0.00 | 2.79 |
| HT29_4_GR_ChDB_0033 | SN38 | 3uM | 6h | ZNF222 | 0.00 | 2.27 |
| HT29_4_GR_ChDB_0033 | SN38 | 3uM | 6h | FBXO5 | 0.00 | 2.06 |
| HT29_4_GR_ChDB_0033 | SN38 | 3uM | 6h | FICD | 0.00 | 2.82 |
| HT29_4_GR_ChDB_0033 | SN38 | 3uM | 6h | GINS3 | 0.00 | 2.04 |
| HT29_4_GR_ChDB_0033 | SN38 | 3uM | 6h | UNKL | 0.00 | 2.22 |
| HT29_4_GR_ChDB_0033 | SN38 | 3uM | 6h | MOAP1 | 0.00 | 2.16 |
| HT29_4_GR_ChDB_0033 | SN38 | 3uM | 6h | SNX5 | 0.00 | 5.33 |
| HT29_4_GR_ChDB_0033 | SN38 | 3uM | 6h | CITED2 | 0.00 | 3.05 |
| HT29_4_GR_ChDB_0033 | SN38 | 3uM | 6h | RNMTL1 | 0.00 | 2.50 |
| HT29_4_GR_ChDB_0033 | SN38 | 3uM | 6h | SNIP1 | 0.00 | 2.32 |
| HT29_4_GR_ChDB_0033 | SN38 | 3uM | 6h | ATP6V0C | 0.00 | 2.26 |
| HT29_4_GR_ChDB_0033 | SN38 | 3uM | 6h | CIAO1 | 0.00 | 2.36 |
| HT29_4_GR_ChDB_0033 | SN38 | 3uM | 6h | NR4A2 | 0.00 | 2.64 |
| HT29_4_GR_ChDB_0033 | SN38 | 3uM | 6h | TNFRSF10D | 0.00 | 5.93 |
| HT29_4_GR_ChDB_0033 | SN38 | 3uM | 6h | MAFF | 0.00 | 2.96 |
| HT29_4_GR_ChDB_0033 | SN38 | 3uM | 6h | TOB2 | 0.00 | 2.66 |
| HT29_4_GR_ChDB_0033 | SN38 | 3uM | 6h | NUDT1 | 0.00 | 2.06 |
| HT29_4_GR_ChDB_0033 | SN38 | 3uM | 6h | THOC6 | 0.00 | 2.36 |
| HT29_4_GR_ChDB_0033 | SN38 | 3uM | 6h | MRPL11 | 0.00 | 2.14 |
| HT29_4_GR_ChDB_0033 | SN38 | 3uM | 6h | C11orf68 | 0.00 | 7.17 |
| HT29_4_GR_ChDB_0033 | SN38 | 3uM | 6h | MSX2 | 0.00 | 3.84 |
| HT29_4_GR_ChDB_0033 | SN38 | 3uM | 6h | CCT5 | 0.00 | 2.63 |
| HT29_4_GR_ChDB_0033 | SN38 | 3uM | 6h | RAB22A | 0.00 | 3.42 |
| HT29_4_GR_ChDB_0033 | SN38 | 3uM | 6h | DPP4 | 0.00 | 2.21 |
| HT29_4_GR_ChDB_0033 | SN38 | 3uM | 6h | MFSD5 | 0.00 | 2.79 |
| HT29_4_GR_ChDB_0033 | SN38 | 3uM | 6h | ZNF468 | 0.00 | 2.05 |
| HT29_4_GR_ChDB_0033 | SN38 | 3uM | 6h | C1orf116 | 0.00 | 2.03 |
| HT29_4_GR_ChDB_0033 | SN38 | 3uM | 6h | TSC1 | 0.00 | 2.06 |
| HT29_4_GR_ChDB_0033 | SN38 | 3uM | 6h | TMEM106C | 0.00 | 2.24 |
| HT29_4_GR_ChDB_0033 | SN38 | 3uM | 6h | DTYMK | 0.00 | 2.16 |
| HT29_4_GR_ChDB_0033 | SN38 | 3uM | 6h | E2F1 | 0.00 | 2.58 |
| HT29_4_GR_ChDB_0033 | SN38 | 3uM | 6h | SUV39H1 | 0.00 | 2.23 |
| HT29_4_GR_ChDB_0033 | SN38 | 3uM | 6h | AUNIP | 0.00 | 2.00 |
| HT29_4_GR_ChDB_0033 | SN38 | 3uM | 6h | BRPF1 | 0.00 | 2.33 |
| HT29_4_GR_ChDB_0033 | SN38 | 3uM | 6h | GTPBP3 | 0.00 | 2.50 |
| HT29_4_GR_ChDB_0033 | SN38 | 3uM | 6h | ZBTB1 | 0.00 | 2.67 |
| HT29_4_GR_ChDB_0033 | SN38 | 3uM | 6h | CBX4 | 0.00 | 2.31 |
| HT29_4_GR_ChDB_0033 | SN38 | 3uM | 6h | TNFSF9 | 0.00 | 2.75 |
| HT29_4_GR_ChDB_0033 | SN38 | 3uM | 6h | OPN3 | 0.00 | 3.02 |
| HT29_4_GR_ChDB_0033 | SN38 | 3uM | 6h | CGGBP1 | 0.00 | 2.10 |
| HT29_4_GR_ChDB_0033 | SN38 | 3uM | 6h | ZNF330 | 0.00 | 2.02 |
| HT29_4_GR_ChDB_0033 | SN38 | 3uM | 6h | LOC100505761 | 0.00 | 2.14 |
| HT29_4_GR_ChDB_0033 | SN38 | 3uM | 6h | NDUFA7 | 0.00 | 2.10 |
| HT29_4_GR_ChDB_0033 | SN38 | 3uM | 6h | PCF11 | 0.00 | 2.52 |
| HT29_4_GR_ChDB_0033 | SN38 | 3uM | 6h | OSR2 | 0.00 | 4.15 |
| HT29_4_GR_ChDB_0033 | SN38 | 3uM | 6h | RIOK2 | 0.00 | 3.31 |
| HT29_4_GR_ChDB_0033 | SN38 | 3uM | 6h | CHCHD7 | 0.00 | 3.21 |
| HT29_4_GR_ChDB_0033 | SN38 | 3uM | 6h | C10orf88 | 0.00 | 2.07 |
| HT29_4_GR_ChDB_0033 | SN38 | 3uM | 6h | MYLIP | 0.00 | 2.25 |
| HT29_4_GR_ChDB_0033 | SN38 | 3uM | 6h | ASTE1 | 0.00 | 2.08 |
| HT29_4_GR_ChDB_0033 | SN38 | 3uM | 6h | RAB14 | 0.00 | 0.38 |
| HT29_4_GR_ChDB_0033 | SN38 | 3uM | 6h | GNAQ | 0.00 | 0.40 |
| HT29_4_GR_ChDB_0033 | SN38 | 3uM | 6h | SHOC2 | 0.00 | 0.33 |
| HT29_4_GR_ChDB_0033 | SN38 | 3uM | 6h | TBC1D4 | 0.00 | 0.28 |
| HT29_4_GR_ChDB_0033 | SN38 | 3uM | 6h | ASF1A | 0.00 | 0.32 |
| HT29_4_GR_ChDB_0033 | SN38 | 3uM | 6h | ADCY7 | 0.00 | 0.47 |
| HT29_4_GR_ChDB_0033 | SN38 | 3uM | 6h | TRIM16 | 0.00 | 0.31 |
| HT29_4_GR_ChDB_0033 | SN38 | 3uM | 6h | SMAD3 | 0.00 | 0.34 |
| HT29_4_GR_ChDB_0033 | SN38 | 3uM | 6h | CHM | 0.00 | 0.41 |
| HT29_4_GR_ChDB_0033 | SN38 | 3uM | 6h | CASP7 | 0.00 | 0.49 |
| HT29_4_GR_ChDB_0033 | SN38 | 3uM | 6h | XPO1 | 0.00 | 0.35 |
| HT29_4_GR_ChDB_0033 | SN38 | 3uM | 6h | NCOA6 | 0.00 | 0.29 |
| HT29_4_GR_ChDB_0033 | SN38 | 3uM | 6h | NUMB | 0.00 | 0.41 |
| HT29_4_GR_ChDB_0033 | SN38 | 3uM | 6h | GNAI1 | 0.00 | 0.21 |
| HT29_4_GR_ChDB_0033 | SN38 | 3uM | 6h | SMAGP | 0.00 | 0.29 |
| HT29_4_GR_ChDB_0033 | SN38 | 3uM | 6h | ZC3H13 | 0.00 | 0.23 |
| HT29_4_GR_ChDB_0033 | SN38 | 3uM | 6h | ANKRD28 | 0.00 | 0.30 |
| HT29_4_GR_ChDB_0033 | SN38 | 3uM | 6h | SNW1 | 0.00 | 0.37 |
| HT29_4_GR_ChDB_0033 | SN38 | 3uM | 6h | SLC7A11 | 0.00 | 0.39 |
| HT29_4_GR_ChDB_0033 | SN38 | 3uM | 6h | PTCD2 | 0.00 | 0.37 |
| HT29_4_GR_ChDB_0033 | SN38 | 3uM | 6h | CDC14B | 0.00 | 0.29 |
| HT29_4_GR_ChDB_0033 | SN38 | 3uM | 6h | TNK2 | 0.00 | 0.41 |
| HT29_4_GR_ChDB_0033 | SN38 | 3uM | 6h | PEX3 | 0.00 | 0.41 |
| HT29_4_GR_ChDB_0033 | SN38 | 3uM | 6h | KIN | 0.00 | 0.14 |
| HT29_4_GR_ChDB_0033 | SN38 | 3uM | 6h | ITSN2 | 0.00 | 0.19 |
| HT29_4_GR_ChDB_0033 | SN38 | 3uM | 6h | EHBP1 | 0.00 | 0.09 |
| HT29_4_GR_ChDB_0033 | SN38 | 3uM | 6h | ASCC3 | 0.00 | 0.43 |
| HT29_4_GR_ChDB_0033 | SN38 | 3uM | 6h | PPFIBP2 | 0.00 | 0.46 |
| HT29_4_GR_ChDB_0033 | SN38 | 3uM | 6h | SSBP3 | 0.00 | 0.45 |
| HT29_4_GR_ChDB_0033 | SN38 | 3uM | 6h | MAP4K3 | 0.00 | 0.17 |
| HT29_4_GR_ChDB_0033 | SN38 | 3uM | 6h | GPR110 | 0.00 | 0.37 |
| HT29_4_GR_ChDB_0033 | SN38 | 3uM | 6h | EPS8 | 0.00 | 0.41 |
| HT29_4_GR_ChDB_0033 | SN38 | 3uM | 6h | MAPK6 | 0.00 | 0.37 |
| HT29_4_GR_ChDB_0033 | SN38 | 3uM | 6h | ELF1 | 0.00 | 0.27 |
| HT29_4_GR_ChDB_0033 | SN38 | 3uM | 6h | PPP6R3 | 0.00 | 0.32 |
| HT29_4_GR_ChDB_0033 | SN38 | 3uM | 6h | RRP15 | 0.00 | 0.43 |
| HT29_4_GR_ChDB_0033 | SN38 | 3uM | 6h | SH2D4A | 0.00 | 0.17 |
| HT29_4_GR_ChDB_0033 | SN38 | 3uM | 6h | PRPF3 | 0.00 | 0.45 |
| HT29_4_GR_ChDB_0033 | SN38 | 3uM | 6h | OFD1 | 0.00 | 0.38 |
| HT29_4_GR_ChDB_0033 | SN38 | 3uM | 6h | ATF2 | 0.00 | 0.22 |
| HT29_4_GR_ChDB_0033 | SN38 | 3uM | 6h | RAB27B | 0.00 | 0.50 |
| HT29_4_GR_ChDB_0033 | SN38 | 3uM | 6h | NFATC3 | 0.00 | 0.33 |
| HT29_4_GR_ChDB_0033 | SN38 | 3uM | 6h | KLF5 | 0.00 | 0.45 |
| HT29_4_GR_ChDB_0033 | SN38 | 3uM | 6h | KLHL9 | 0.00 | 0.33 |
| HT29_4_GR_ChDB_0033 | SN38 | 3uM | 6h | PKP2 | 0.00 | 0.33 |
| HT29_4_GR_ChDB_0033 | SN38 | 3uM | 6h | MTERFD2 | 0.00 | 0.36 |
| HT29_4_GR_ChDB_0033 | SN38 | 3uM | 6h | SLC38A2 | 0.00 | 0.46 |
| HT29_4_GR_ChDB_0033 | SN38 | 3uM | 6h | RNF111 | 0.00 | 0.44 |
| HT29_4_GR_ChDB_0033 | SN38 | 3uM | 6h | EHMT1 | 0.00 | 0.26 |
| HT29_4_GR_ChDB_0033 | SN38 | 3uM | 6h | METTL4 | 0.00 | 0.42 |
| HT29_4_GR_ChDB_0033 | SN38 | 3uM | 6h | EHF | 0.00 | 0.38 |
| HT29_4_GR_ChDB_0033 | SN38 | 3uM | 6h | SMEK1 | 0.00 | 0.23 |
| HT29_4_GR_ChDB_0033 | SN38 | 3uM | 6h | KIF13A | 0.00 | 0.23 |
| HT29_4_GR_ChDB_0033 | SN38 | 3uM | 6h | HOXB8 | 0.00 | 0.25 |
| HT29_4_GR_ChDB_0033 | SN38 | 3uM | 6h | MED17 | 0.00 | 0.42 |
| HT29_4_GR_ChDB_0033 | SN38 | 3uM | 6h | PHF20L1 | 0.00 | 0.30 |
| HT29_4_GR_ChDB_0033 | SN38 | 3uM | 6h | MYCBP2 | 0.00 | 0.31 |
| HT29_4_GR_ChDB_0033 | SN38 | 3uM | 6h | EZH2 | 0.00 | 0.46 |
| HT29_4_GR_ChDB_0033 | SN38 | 3uM | 6h | PBX3 | 0.00 | 0.49 |
| HT29_4_GR_ChDB_0033 | SN38 | 3uM | 6h | NDC80 | 0.00 | 0.18 |
| HT29_4_GR_ChDB_0033 | SN38 | 3uM | 6h | ZNF518A | 0.00 | 0.23 |
| HT29_4_GR_ChDB_0033 | SN38 | 3uM | 6h | PANX1 | 0.00 | 0.42 |
| HT29_4_GR_ChDB_0033 | SN38 | 3uM | 6h | LRRC8B | 0.00 | 0.23 |
| HT29_4_GR_ChDB_0033 | SN38 | 3uM | 6h | RAP2B | 0.00 | 0.45 |
| HT29_4_GR_ChDB_0033 | SN38 | 3uM | 6h | BAZ1A | 0.00 | 0.27 |
| HT29_4_GR_ChDB_0033 | SN38 | 3uM | 6h | REV1 | 0.00 | 0.32 |
| HT29_4_GR_ChDB_0033 | SN38 | 3uM | 6h | RPAP3 | 0.00 | 0.50 |
| HT29_4_GR_ChDB_0033 | SN38 | 3uM | 6h | YEATS4 | 0.00 | 0.23 |
| HT29_4_GR_ChDB_0033 | SN38 | 3uM | 6h | JMJD1C | 0.00 | 0.19 |
| HT29_4_GR_ChDB_0033 | SN38 | 3uM | 6h | ADNP | 0.00 | 0.47 |
| HT29_4_GR_ChDB_0033 | SN38 | 3uM | 6h | ATXN2 | 0.00 | 0.44 |
| HT29_4_GR_ChDB_0033 | SN38 | 3uM | 6h | STK10 | 0.00 | 0.39 |
| HT29_4_GR_ChDB_0033 | SN38 | 3uM | 6h | SPTLC2 | 0.00 | 0.34 |
| HT29_4_GR_ChDB_0033 | SN38 | 3uM | 6h | NCK2 | 0.00 | 0.46 |
| HT29_4_GR_ChDB_0033 | SN38 | 3uM | 6h | PRKX | 0.00 | 0.39 |
| HT29_4_GR_ChDB_0033 | SN38 | 3uM | 6h | CDC7 | 0.00 | 0.30 |
| HT29_4_GR_ChDB_0033 | SN38 | 3uM | 6h | ORC2 | 0.00 | 0.19 |
| HT29_4_GR_ChDB_0033 | SN38 | 3uM | 6h | MPP6 | 0.00 | 0.47 |
| HT29_4_GR_ChDB_0033 | SN38 | 3uM | 6h | POLR3G | 0.00 | 0.35 |
| HT29_4_GR_ChDB_0033 | SN38 | 3uM | 6h | SIK3 | 0.00 | 0.30 |
| HT29_4_GR_ChDB_0033 | SN38 | 3uM | 6h | PIKFYVE | 0.00 | 0.49 |
| HT29_4_GR_ChDB_0033 | SN38 | 3uM | 6h | MORC2 | 0.00 | 0.39 |
| HT29_4_GR_ChDB_0033 | SN38 | 3uM | 6h | RBM26 | 0.00 | 0.42 |
| HT29_4_GR_ChDB_0033 | SN38 | 3uM | 6h | SYBU | 0.00 | 0.41 |
| HT29_4_GR_ChDB_0033 | SN38 | 3uM | 6h | UTP6 | 0.00 | 0.47 |
| HT29_4_GR_ChDB_0033 | SN38 | 3uM | 6h | SLC25A32 | 0.00 | 0.39 |
| HT29_4_GR_ChDB_0033 | SN38 | 3uM | 6h | KPNA3 | 0.00 | 0.46 |
| HT29_4_GR_ChDB_0033 | SN38 | 3uM | 6h | RAD54L | 0.00 | 0.48 |
| HT29_4_GR_ChDB_0033 | SN38 | 3uM | 6h | DNAJC6 | 0.00 | 0.34 |
| HT29_4_GR_ChDB_0033 | SN38 | 3uM | 6h | EFNA4 | 0.00 | 0.42 |
| HT29_4_GR_ChDB_0033 | SN38 | 3uM | 6h | OVOL2 | 0.00 | 0.22 |
| HT29_4_GR_ChDB_0033 | SN38 | 3uM | 6h | FBXW11 | 0.00 | 0.20 |
| HT29_4_GR_ChDB_0033 | SN38 | 3uM | 6h | ZFAND5 | 0.00 | 0.49 |
| HT29_4_GR_ChDB_0033 | SN38 | 3uM | 6h | RTF1 | 0.00 | 0.41 |
| HT29_4_GR_ChDB_0033 | SN38 | 3uM | 6h | PDE12 | 0.00 | 0.45 |
| HT29_4_GR_ChDB_0033 | SN38 | 3uM | 6h | MECOM | 0.00 | 0.18 |
| HT29_4_GR_ChDB_0033 | SN38 | 3uM | 6h | BTBD7 | 0.00 | 0.41 |
| HT29_4_GR_ChDB_0033 | SN38 | 3uM | 6h | TIAL1 | 0.00 | 0.41 |
| HT29_4_GR_ChDB_0033 | SN38 | 3uM | 6h | HERC2 | 0.00 | 0.35 |
| HT29_4_GR_ChDB_0033 | SN38 | 3uM | 6h | ZCCHC6 | 0.00 | 0.30 |
| HT29_4_GR_ChDB_0033 | SN38 | 3uM | 6h | DIAPH3 | 0.00 | 0.37 |
| HT29_4_GR_ChDB_0033 | SN38 | 3uM | 6h | FAM135A | 0.00 | 0.15 |
| HT29_4_GR_ChDB_0033 | SN38 | 3uM | 6h | LIMD1 | 0.00 | 0.37 |
| HT29_4_GR_ChDB_0033 | SN38 | 3uM | 6h | ARMC8 | 0.00 | 0.48 |
| HT29_4_GR_ChDB_0033 | SN38 | 3uM | 6h | MDN1 | 0.00 | 0.46 |
| HT29_4_GR_ChDB_0033 | SN38 | 3uM | 6h | ARHGEF18 | 0.00 | 0.48 |
| HT29_4_GR_ChDB_0033 | SN38 | 3uM | 6h | GTF2IRD1 | 0.00 | 0.25 |
| HT29_4_GR_ChDB_0033 | SN38 | 3uM | 6h | NOL9 | 0.00 | 0.48 |
| HT29_4_GR_ChDB_0033 | SN38 | 3uM | 6h | CSPP1 | 0.00 | 0.28 |
| HT29_4_GR_ChDB_0033 | SN38 | 3uM | 6h | PTPN3 | 0.00 | 0.28 |
| HT29_4_GR_ChDB_0033 | SN38 | 3uM | 6h | EXOSC9 | 0.00 | 0.34 |
| HT29_4_GR_ChDB_0033 | SN38 | 3uM | 6h | ORC5 | 0.00 | 0.19 |
| HT29_4_GR_ChDB_0033 | SN38 | 3uM | 6h | KDM5A | 0.00 | 0.28 |
| HT29_4_GR_ChDB_0033 | SN38 | 3uM | 6h | EDN1 | 0.00 | 0.39 |
| HT29_4_GR_ChDB_0033 | SN38 | 3uM | 6h | RC3H2 | 0.00 | 0.32 |
| HT29_4_GR_ChDB_0033 | SN38 | 3uM | 6h | TUBD1 | 0.00 | 0.44 |
| HT29_4_GR_ChDB_0033 | SN38 | 3uM | 6h | MAGEF1 | 0.00 | 0.41 |
| HT29_4_GR_ChDB_0033 | SN38 | 3uM | 6h | USP3 | 0.00 | 0.33 |
| HT29_4_GR_ChDB_0033 | SN38 | 3uM | 6h | PITPNB | 0.00 | 0.32 |
| HT29_4_GR_ChDB_0033 | SN38 | 3uM | 6h | SPAG1 | 0.00 | 0.36 |
| HT29_4_GR_ChDB_0033 | SN38 | 3uM | 6h | MGAT5 | 0.00 | 0.21 |
| HT29_4_GR_ChDB_0033 | SN38 | 3uM | 6h | OXR1 | 0.00 | 0.34 |
| HT29_4_GR_ChDB_0033 | SN38 | 3uM | 6h | KIF5B | 0.00 | 0.47 |
| HT29_4_GR_ChDB_0033 | SN38 | 3uM | 6h | USP8 | 0.00 | 0.39 |
| HT29_4_GR_ChDB_0033 | SN38 | 3uM | 6h | STAM | 0.00 | 0.31 |
| HT29_4_GR_ChDB_0033 | SN38 | 3uM | 6h | PTPN13 | 0.00 | 0.35 |
| HT29_4_GR_ChDB_0033 | SN38 | 3uM | 6h | RMND5B | 0.00 | 0.39 |
| HT29_4_GR_ChDB_0033 | SN38 | 3uM | 6h | TMEM223 | 0.00 | 0.29 |
| HT29_4_GR_ChDB_0033 | SN38 | 3uM | 6h | PYROXD1 | 0.00 | 0.43 |
| HT29_4_GR_ChDB_0033 | SN38 | 3uM | 6h | AGFG1 | 0.00 | 0.24 |
| HT29_4_GR_ChDB_0033 | SN38 | 3uM | 6h | ARID4B | 0.00 | 0.16 |
| HT29_4_GR_ChDB_0033 | SN38 | 3uM | 6h | MTMR6 | 0.00 | 0.40 |
| HT29_4_GR_ChDB_0033 | SN38 | 3uM | 6h | SBNO1 | 0.00 | 0.47 |
| HT29_4_GR_ChDB_0033 | SN38 | 3uM | 6h | FAM208B | 0.00 | 0.30 |
| HT29_4_GR_ChDB_0033 | SN38 | 3uM | 6h | SDAD1 | 0.00 | 0.46 |
| HT29_4_GR_ChDB_0033 | SN38 | 3uM | 6h | TFB1M | 0.00 | 0.25 |
| HT29_4_GR_ChDB_0033 | SN38 | 3uM | 6h | HSPA14 | 0.00 | 0.34 |
| HT29_4_GR_ChDB_0033 | SN38 | 3uM | 6h | PFKFB3 | 0.00 | 0.50 |
| HT29_4_GR_ChDB_0033 | SN38 | 3uM | 6h | SSFA2 | 0.00 | 0.19 |
| HT29_4_GR_ChDB_0033 | SN38 | 3uM | 6h | IL8 | 0.00 | 0.37 |
| HT29_4_GR_ChDB_0033 | SN38 | 3uM | 6h | SMAD5 | 0.00 | 0.44 |
| HT29_4_GR_ChDB_0033 | SN38 | 3uM | 6h | EREG | 0.00 | 0.26 |
| HT29_4_GR_ChDB_0033 | SN38 | 3uM | 6h | MPHOSPH9 | 0.00 | 0.25 |
| HT29_4_GR_ChDB_0033 | SN38 | 3uM | 6h | FUT4 | 0.00 | 0.24 |
| HT29_4_GR_ChDB_0033 | SN38 | 3uM | 6h | CEP192 | 0.00 | 0.32 |
| HT29_4_GR_ChDB_0033 | SN38 | 3uM | 6h | C4orf19 | 0.00 | 0.33 |
| HT29_4_GR_ChDB_0033 | SN38 | 3uM | 6h | RBM23 | 0.00 | 0.50 |
| HT29_4_GR_ChDB_0033 | SN38 | 3uM | 6h | EGLN1 | 0.00 | 0.24 |
| HT29_4_GR_ChDB_0033 | SN38 | 3uM | 6h | HOXB7 | 0.00 | 0.33 |
| HT29_4_GR_ChDB_0033 | SN38 | 3uM | 6h | DNAJA2 | 0.00 | 0.45 |
| HT29_4_GR_ChDB_0033 | SN38 | 3uM | 6h | ECT2 | 0.00 | 0.37 |
| HT29_4_GR_ChDB_0033 | SN38 | 3uM | 6h | FOXO3 | 0.00 | 0.40 |
| HT29_4_GR_ChDB_0033 | SN38 | 3uM | 6h | TRIB3 | 0.00 | 0.44 |
| HT29_4_GR_ChDB_0033 | SN38 | 3uM | 6h | CHD7 | 0.00 | 0.19 |
| HT29_4_GR_ChDB_0033 | SN38 | 3uM | 6h | ZNF207 | 0.00 | 0.38 |
| HT29_4_GR_ChDB_0033 | SN38 | 3uM | 6h | FARS2 | 0.00 | 0.42 |
| HT29_4_GR_ChDB_0033 | SN38 | 3uM | 6h | KALRN | 0.00 | 0.49 |
| HT29_4_GR_ChDB_0033 | SN38 | 3uM | 6h | NGLY1 | 0.00 | 0.49 |
| HT29_4_GR_ChDB_0033 | SN38 | 3uM | 6h | LRRC1 | 0.00 | 0.12 |
| HT29_4_GR_ChDB_0033 | SN38 | 3uM | 6h | FBXL5 | 0.00 | 0.31 |
| HT29_4_GR_ChDB_0033 | SN38 | 3uM | 6h | PHC3 | 0.00 | 0.45 |
| HT29_4_GR_ChDB_0033 | SN38 | 3uM | 6h | KDM4B | 0.00 | 0.47 |
| HT29_4_GR_ChDB_0033 | SN38 | 3uM | 6h | UBXN7 | 0.00 | 0.32 |
| HT29_4_GR_ChDB_0033 | SN38 | 3uM | 6h | CNOT6 | 0.00 | 0.38 |
| HT29_4_GR_ChDB_0033 | SN38 | 3uM | 6h | DCUN1D1 | 0.00 | 0.50 |
| HT29_4_GR_ChDB_0033 | SN38 | 3uM | 6h | TMEM39A | 0.00 | 0.46 |
| HT29_4_GR_ChDB_0033 | SN38 | 3uM | 6h | COL4A3BP | 0.00 | 0.25 |
| HT29_4_GR_ChDB_0033 | SN38 | 3uM | 6h | DENND1B | 0.00 | 0.29 |
| HT29_4_GR_ChDB_0033 | SN38 | 3uM | 6h | RAI1 | 0.00 | 0.31 |
| HT29_4_GR_ChDB_0033 | SN38 | 3uM | 6h | POLR1B | 0.00 | 0.40 |
| HT29_4_GR_ChDB_0033 | SN38 | 3uM | 6h | ZNF215 | 0.00 | 0.39 |
| HT29_4_GR_ChDB_0033 | SN38 | 3uM | 6h | RALGPS2 | 0.00 | 0.44 |
| HT29_4_GR_ChDB_0033 | SN38 | 3uM | 6h | SYTL2 | 0.00 | 0.36 |
| HT29_4_GR_ChDB_0033 | SN38 | 3uM | 6h | GNB1L | 0.00 | 0.23 |
| HT29_4_GR_ChDB_0033 | SN38 | 3uM | 6h | MPHOSPH8 | 0.00 | 0.48 |
| HT29_4_GR_ChDB_0033 | SN38 | 3uM | 6h | TNS4 | 0.00 | 0.12 |
| HT29_4_GR_ChDB_0033 | SN38 | 3uM | 6h | PVR | 0.00 | 0.44 |
| HT29_4_GR_ChDB_0033 | SN38 | 3uM | 6h | LPIN2 | 0.00 | 0.36 |
| HT29_4_GR_ChDB_0033 | SN38 | 3uM | 6h | DMXL1 | 0.00 | 0.40 |
| HT29_4_GR_ChDB_0033 | SN38 | 3uM | 6h | CXADR | 0.00 | 0.34 |
| HT29_4_GR_ChDB_0033 | SN38 | 3uM | 6h | GULP1 | 0.00 | 0.34 |
| HT29_4_GR_ChDB_0033 | SN38 | 3uM | 6h | CLOCK | 0.00 | 0.34 |
| HT29_4_GR_ChDB_0033 | SN38 | 3uM | 6h | PTPN14 | 0.00 | 0.32 |
| HT29_4_GR_ChDB_0033 | SN38 | 3uM | 6h | CLIP1 | 0.00 | 0.19 |
| HT29_4_GR_ChDB_0033 | SN38 | 3uM | 6h | RC3H1 | 0.00 | 0.28 |
| HT29_4_GR_ChDB_0033 | SN38 | 3uM | 6h | RSF1 | 0.00 | 0.36 |
| HT29_4_GR_ChDB_0033 | SN38 | 3uM | 6h | ATAD2 | 0.00 | 0.40 |
| HT29_4_GR_ChDB_0033 | SN38 | 3uM | 6h | NAA16 | 0.00 | 0.36 |
| HT29_4_GR_ChDB_0033 | SN38 | 3uM | 6h | THADA | 0.00 | 0.40 |
| HT29_4_GR_ChDB_0033 | SN38 | 3uM | 6h | INTS6 | 0.00 | 0.31 |
| HT29_4_GR_ChDB_0033 | SN38 | 3uM | 6h | CARD10 | 0.00 | 0.35 |
| HT29_4_GR_ChDB_0033 | SN38 | 3uM | 6h | CDC42SE1 | 0.00 | 0.44 |
| HT29_4_GR_ChDB_0033 | SN38 | 3uM | 6h | TMEM57 | 0.00 | 0.47 |
| HT29_4_GR_ChDB_0033 | SN38 | 3uM | 6h | PPP1R10 | 0.00 | 0.46 |
| HT29_4_GR_ChDB_0033 | SN38 | 3uM | 6h | MARK3 | 0.00 | 0.34 |
| HT29_4_GR_ChDB_0033 | SN38 | 3uM | 6h | XK | 0.00 | 0.50 |
| HT29_4_GR_ChDB_0033 | SN38 | 3uM | 6h | WNT11 | 0.00 | 0.23 |
| HT29_4_GR_ChDB_0033 | SN38 | 3uM | 6h | ZFHX3 | 0.00 | 0.24 |
| HT29_4_GR_ChDB_0033 | SN38 | 3uM | 6h | USP15 | 0.00 | 0.38 |
| HT29_4_GR_ChDB_0033 | SN38 | 3uM | 6h | PLAA | 0.00 | 0.40 |
| HT29_4_GR_ChDB_0033 | SN38 | 3uM | 6h | ITGB8 | 0.00 | 0.17 |
| HT29_4_GR_ChDB_0033 | SN38 | 3uM | 6h | MON2 | 0.00 | 0.42 |
| HT29_4_GR_ChDB_0033 | SN38 | 3uM | 6h | WDR67 | 0.00 | 0.33 |
| HT29_4_GR_ChDB_0033 | SN38 | 3uM | 6h | RAB11FIP3 | 0.00 | 0.44 |
| HT29_4_GR_ChDB_0033 | SN38 | 3uM | 6h | HIF1AN | 0.00 | 0.40 |
| HT29_4_GR_ChDB_0033 | SN38 | 3uM | 6h | RFWD3 | 0.00 | 0.43 |
| HT29_4_GR_ChDB_0033 | SN38 | 3uM | 6h | KIAA1551 | 0.00 | 0.47 |
| HT29_4_GR_ChDB_0033 | SN38 | 3uM | 6h | SMUG1 | 0.00 | 0.49 |
| HT29_4_GR_ChDB_0033 | SN38 | 3uM | 6h | RNF43 | 0.00 | 0.18 |
| HT29_4_GR_ChDB_0033 | SN38 | 3uM | 6h | FBXW7 | 0.00 | 0.23 |
| HT29_4_GR_ChDB_0033 | SN38 | 3uM | 6h | NIN | 0.00 | 0.20 |
| HT29_4_GR_ChDB_0033 | SN38 | 3uM | 6h | CLSPN | 0.00 | 0.36 |
| HT29_4_GR_ChDB_0033 | SN38 | 3uM | 6h | ARL15 | 0.00 | 0.25 |
| HT29_4_GR_ChDB_0033 | SN38 | 3uM | 6h | ANKH | 0.00 | 0.45 |
| HT29_4_GR_ChDB_0033 | SN38 | 3uM | 6h | CASC5 | 0.00 | 0.33 |
| HT29_4_GR_ChDB_0033 | SN38 | 3uM | 6h | TRMT1L | 0.00 | 0.42 |
| HT29_4_GR_ChDB_0033 | SN38 | 3uM | 6h | SMG9 | 0.00 | 0.44 |
| HT29_4_GR_ChDB_0033 | SN38 | 3uM | 6h | ARHGEF26 | 0.00 | 0.38 |
| HT29_4_GR_ChDB_0033 | SN38 | 3uM | 6h | LOC389906 | 0.00 | 0.48 |
| HT29_4_GR_ChDB_0033 | SN38 | 3uM | 6h | PAPOLG | 0.00 | 0.43 |
| HT29_4_GR_ChDB_0033 | SN38 | 3uM | 6h | UNC50 | 0.00 | 0.48 |
| HT29_4_GR_ChDB_0033 | SN38 | 3uM | 6h | PDCD10 | 0.00 | 0.32 |
| HT29_4_GR_ChDB_0033 | SN38 | 3uM | 6h | DHX32 | 0.00 | 0.31 |
| HT29_4_GR_ChDB_0033 | SN38 | 3uM | 6h | TMEM127 | 0.00 | 0.29 |
| HT29_4_GR_ChDB_0033 | SN38 | 3uM | 6h | PSMC6 | 0.00 | 0.41 |
| HT29_4_GR_ChDB_0033 | SN38 | 3uM | 6h | KIAA0247 | 0.00 | 0.46 |
| HT29_4_GR_ChDB_0033 | SN38 | 3uM | 6h | TP53BP2 | 0.00 | 0.45 |
| HT29_4_GR_ChDB_0033 | SN38 | 3uM | 6h | CD2AP | 0.00 | 0.25 |
| HT29_4_GR_ChDB_0033 | SN38 | 3uM | 6h | USP6NL | 0.00 | 0.29 |
| HT29_4_GR_ChDB_0033 | SN38 | 3uM | 6h | ATP2B1 | 0.00 | 0.26 |
| HT29_4_GR_ChDB_0033 | SN38 | 3uM | 6h | ANKS1A | 0.00 | 0.15 |
| HT29_4_GR_ChDB_0033 | SN38 | 3uM | 6h | KANK1 | 0.00 | 0.30 |
| HT29_4_GR_ChDB_0033 | SN38 | 3uM | 6h | SASH1 | 0.00 | 0.24 |
| HT29_4_GR_ChDB_0033 | SN38 | 3uM | 6h | SOGA2 | 0.00 | 0.17 |
| HT29_4_GR_ChDB_0033 | SN38 | 3uM | 6h | MAPKAP1 | 0.00 | 0.42 |
| HT29_4_GR_ChDB_0033 | SN38 | 3uM | 6h | CAPRIN2 | 0.00 | 0.36 |
| HT29_4_GR_ChDB_0033 | SN38 | 3uM | 6h | SHQ1 | 0.00 | 0.31 |
| HT29_4_GR_ChDB_0033 | SN38 | 3uM | 6h | TSPAN14 | 0.00 | 0.45 |
| HT29_4_GR_ChDB_0033 | SN38 | 3uM | 6h | TRMT61B | 0.00 | 0.24 |
| HT29_4_GR_ChDB_0033 | SN38 | 3uM | 6h | ANKRD27 | 0.00 | 0.39 |
| HT29_4_GR_ChDB_0033 | SN38 | 3uM | 6h | SARS | 0.00 | 0.50 |
| HT29_4_GR_ChDB_0033 | SN38 | 3uM | 6h | PSEN1 | 0.00 | 0.31 |
| HT29_4_GR_ChDB_0033 | SN38 | 3uM | 6h | HELZ | 0.00 | 0.30 |
| HT29_4_GR_ChDB_0033 | SN38 | 3uM | 6h | COX10 | 0.00 | 0.32 |
| HT29_4_GR_ChDB_0033 | SN38 | 3uM | 6h | CDS1 | 0.00 | 0.41 |
| HT29_4_GR_ChDB_0033 | SN38 | 3uM | 6h | TSPAN5 | 0.00 | 0.33 |
| HT29_4_GR_ChDB_0033 | SN38 | 3uM | 6h | DTL | 0.00 | 0.24 |
| HT29_4_GR_ChDB_0033 | SN38 | 3uM | 6h | PDGFC | 0.00 | 0.20 |
| HT29_4_GR_ChDB_0033 | SN38 | 3uM | 6h | PPA2 | 0.00 | 0.44 |
| HT29_4_GR_ChDB_0033 | SN38 | 3uM | 6h | UVRAG | 0.00 | 0.47 |
| HT29_4_GR_ChDB_0033 | SN38 | 3uM | 6h | SMURF2 | 0.00 | 0.30 |
| HT29_4_GR_ChDB_0033 | SN38 | 3uM | 6h | OCLN | 0.00 | 0.48 |
| HT29_4_GR_ChDB_0033 | SN38 | 3uM | 6h | GPRC5A | 0.00 | 0.39 |
| HT29_4_GR_ChDB_0033 | SN38 | 3uM | 6h | CEP170 | 0.00 | 0.18 |
| HT29_4_GR_ChDB_0033 | SN38 | 3uM | 6h | PIP4K2A | 0.00 | 0.24 |
| HT29_4_GR_ChDB_0033 | SN38 | 3uM | 6h | SLC16A5 | 0.00 | 0.40 |
| HT29_4_GR_ChDB_0033 | SN38 | 3uM | 6h | NUP85 | 0.00 | 0.36 |
| HT29_4_GR_ChDB_0033 | SN38 | 3uM | 6h | C12orf4 | 0.00 | 0.34 |
| HT29_4_GR_ChDB_0033 | SN38 | 3uM | 6h | ELP3 | 0.00 | 0.35 |
| HT29_4_GR_ChDB_0033 | SN38 | 3uM | 6h | IPO7 | 0.00 | 0.35 |
| HT29_4_GR_ChDB_0033 | SN38 | 3uM | 6h | PTPRB | 0.00 | 0.38 |
| HT29_4_GR_ChDB_0033 | SN38 | 3uM | 6h | CUL1 | 0.00 | 0.16 |
| HT29_4_GR_ChDB_0033 | SN38 | 3uM | 6h | AFF1 | 0.00 | 0.19 |
| HT29_4_GR_ChDB_0033 | SN38 | 3uM | 6h | STX3 | 0.00 | 0.23 |
| HT29_4_GR_ChDB_0033 | SN38 | 3uM | 6h | ESRP2 | 0.00 | 0.26 |
| HT29_4_GR_ChDB_0033 | SN38 | 3uM | 6h | KLF3 | 0.00 | 0.48 |
| HT29_4_GR_ChDB_0033 | SN38 | 3uM | 6h | LATS1 | 0.00 | 0.44 |
| HT29_4_GR_ChDB_0033 | SN38 | 3uM | 6h | HOOK1 | 0.00 | 0.48 |
| HT29_4_GR_ChDB_0033 | SN38 | 3uM | 6h | PACSIN2 | 0.00 | 0.34 |
| HT29_4_GR_ChDB_0033 | SN38 | 3uM | 6h | TRAK1 | 0.00 | 0.47 |
| HT29_4_GR_ChDB_0033 | SN38 | 3uM | 6h | AHR | 0.00 | 0.50 |
| HT29_4_GR_ChDB_0033 | SN38 | 3uM | 6h | ATXN1 | 0.00 | 0.43 |
| HT29_4_GR_ChDB_0033 | SN38 | 3uM | 6h | TRIP4 | 0.00 | 0.37 |
| HT29_4_GR_ChDB_0033 | SN38 | 3uM | 6h | SHB | 0.00 | 0.30 |
| HT29_4_GR_ChDB_0033 | SN38 | 3uM | 6h | ESPL1 | 0.00 | 0.41 |
| HT29_4_GR_ChDB_0033 | SN38 | 3uM | 6h | FANCC | 0.00 | 0.45 |
| HT29_4_GR_ChDB_0033 | SN38 | 3uM | 6h | LRP8 | 0.00 | 0.41 |
| HT29_4_GR_ChDB_0033 | SN38 | 3uM | 6h | HNF1B | 0.00 | 0.36 |
| HT29_4_GR_ChDB_0033 | SN38 | 3uM | 6h | DGKD | 0.00 | 0.28 |
| HT29_4_GR_ChDB_0033 | SN38 | 3uM | 6h | KPNA4 | 0.00 | 0.41 |
| HT29_4_GR_ChDB_0033 | SN38 | 3uM | 6h | FAM102A | 0.00 | 0.40 |
| HT29_4_GR_ChDB_0033 | SN38 | 3uM | 6h | SPECC1L | 0.00 | 0.39 |
| HT29_4_GR_ChDB_0033 | SN38 | 3uM | 6h | SIK2 | 0.00 | 0.46 |
| HT29_4_GR_ChDB_0033 | SN38 | 3uM | 6h | SIPA1L3 | 0.00 | 0.49 |
| HT29_4_GR_ChDB_0033 | SN38 | 3uM | 6h | TBC1D30 | 0.00 | 0.29 |
| HT29_4_GR_ChDB_0033 | SN38 | 3uM | 6h | ZNF451 | 0.00 | 0.47 |
| HT29_4_GR_ChDB_0033 | SN38 | 3uM | 6h | RBM47 | 0.00 | 0.33 |
| HT29_4_GR_ChDB_0033 | SN38 | 3uM | 6h | TEX2 | 0.00 | 0.32 |
| HT29_4_GR_ChDB_0033 | SN38 | 3uM | 6h | ZCCHC8 | 0.00 | 0.38 |
| HT29_4_GR_ChDB_0033 | SN38 | 3uM | 6h | ZNF277 | 0.00 | 0.33 |
| HT29_4_GR_ChDB_0033 | SN38 | 3uM | 6h | PANK2 | 0.00 | 0.31 |
| HT29_4_GR_ChDB_0033 | SN38 | 3uM | 6h | IPPK | 0.00 | 0.32 |
| HT29_4_GR_ChDB_0033 | SN38 | 3uM | 6h | DCAF16 | 0.00 | 0.33 |
| HT29_4_GR_ChDB_0033 | SN38 | 3uM | 6h | LUZP1 | 0.00 | 0.41 |
| HT29_4_GR_ChDB_0033 | SN38 | 3uM | 6h | POLR3B | 0.00 | 0.17 |
| HT29_4_GR_ChDB_0033 | SN38 | 3uM | 6h | PREP | 0.00 | 0.48 |
| HT29_4_GR_ChDB_0033 | SN38 | 3uM | 6h | MLLT3 | 0.00 | 0.31 |
| HT29_4_GR_ChDB_0033 | SN38 | 3uM | 6h | SEMA3A | 0.00 | 0.30 |
| HT29_4_GR_ChDB_0033 | SN38 | 3uM | 6h | TOR1AIP2 | 0.00 | 0.42 |
| HT29_4_GR_ChDB_0033 | SN38 | 3uM | 6h | KLHDC2 | 0.00 | 0.32 |
| HT29_4_GR_ChDB_0033 | SN38 | 3uM | 6h | CAAP1 | 0.00 | 0.38 |
| HT29_4_GR_ChDB_0033 | SN38 | 3uM | 6h | MFSD6 | 0.00 | 0.41 |
| HT29_4_GR_ChDB_0035 | Topotecan | 3uM | 6h | CDK6 | 1.00 | 0.25 |
| HT29_4_GR_ChDB_0035 | Topotecan | 3uM | 6h | PTK2 | 0.99 | 0.12 |
| HT29_4_GR_ChDB_0035 | Topotecan | 3uM | 6h | IGF1R | 0.59 | 0.22 |
| HT29_4_GR_ChDB_0035 | Topotecan | 3uM | 6h | MYC | 0.46 | 0.34 |
| HT29_4_GR_ChDB_0035 | Topotecan | 3uM | 6h | MET | 0.45 | 0.35 |
| HT29_4_GR_ChDB_0035 | Topotecan | 3uM | 6h | RPS6KA3 | 0.34 | 0.34 |
| HT29_4_GR_ChDB_0035 | Topotecan | 3uM | 6h | EXT1 | 0.33 | 0.13 |
| HT29_4_GR_ChDB_0035 | Topotecan | 3uM | 6h | PIK3C2A | 0.21 | 0.50 |
| HT29_4_GR_ChDB_0035 | Topotecan | 3uM | 6h | EGFR | 0.15 | 0.40 |
| HT29_4_GR_ChDB_0035 | Topotecan | 3uM | 6h | CAMK2D | 0.15 | 0.48 |
| HT29_4_GR_ChDB_0035 | Topotecan | 3uM | 6h | MELK | 0.14 | 0.28 |
| HT29_4_GR_ChDB_0035 | Topotecan | 3uM | 6h | IGF2BP2 | 0.13 | 0.29 |
| HT29_4_GR_ChDB_0035 | Topotecan | 3uM | 6h | MSH2 | 0.12 | 0.42 |
| HT29_4_GR_ChDB_0035 | Topotecan | 3uM | 6h | PIK3R1 | 0.12 | 0.37 |
| HT29_4_GR_ChDB_0035 | Topotecan | 3uM | 6h | BRCA1 | 0.11 | 0.44 |
| HT29_4_GR_ChDB_0035 | Topotecan | 3uM | 6h | TGFBR2 | 0.10 | 0.32 |
| HT29_4_GR_ChDB_0035 | Topotecan | 3uM | 6h | MAP2K4 | 0.09 | 0.46 |
| HT29_4_GR_ChDB_0035 | Topotecan | 3uM | 6h | ROCK1 | 0.08 | 0.42 |
| HT29_4_GR_ChDB_0035 | Topotecan | 3uM | 6h | KIF11 | 0.08 | 0.42 |
| HT29_4_GR_ChDB_0035 | Topotecan | 3uM | 6h | TCF7L2 | 0.08 | 0.15 |
| HT29_4_GR_ChDB_0035 | Topotecan | 3uM | 6h | GSK3B | 0.07 | 0.28 |
| HT29_4_GR_ChDB_0035 | Topotecan | 3uM | 6h | ADM | 0.07 | 5.24 |
| HT29_4_GR_ChDB_0035 | Topotecan | 3uM | 6h | CDK8 | 0.07 | 0.45 |
| HT29_4_GR_ChDB_0035 | Topotecan | 3uM | 6h | PIK3CB | 0.06 | 0.27 |
| HT29_4_GR_ChDB_0035 | Topotecan | 3uM | 6h | POT1 | 0.06 | 0.37 |
| HT29_4_GR_ChDB_0035 | Topotecan | 3uM | 6h | STK24 | 0.06 | 0.41 |
| HT29_4_GR_ChDB_0035 | Topotecan | 3uM | 6h | MAP3K5 | 0.06 | 0.41 |
| HT29_4_GR_ChDB_0035 | Topotecan | 3uM | 6h | NLK | 0.06 | 0.40 |
| HT29_4_GR_ChDB_0035 | Topotecan | 3uM | 6h | BMPR1A | 0.06 | 0.32 |
| HT29_4_GR_ChDB_0035 | Topotecan | 3uM | 6h | MBNL1 | 0.05 | 0.19 |
| HT29_4_GR_ChDB_0035 | Topotecan | 3uM | 6h | BUB1B | 0.05 | 0.34 |
| HT29_4_GR_ChDB_0035 | Topotecan | 3uM | 6h | CSNK1A1 | 0.05 | 0.44 |
| HT29_4_GR_ChDB_0035 | Topotecan | 3uM | 6h | STK39 | 0.05 | 0.26 |
| HT29_4_GR_ChDB_0035 | Topotecan | 3uM | 6h | IRS1 | 0.05 | 0.30 |
| HT29_4_GR_ChDB_0035 | Topotecan | 3uM | 6h | HBEGF | 0.04 | 2.52 |
| HT29_4_GR_ChDB_0035 | Topotecan | 3uM | 6h | GRB10 | 0.04 | 0.39 |
| HT29_4_GR_ChDB_0035 | Topotecan | 3uM | 6h | MBNL2 | 0.04 | 0.23 |
| HT29_4_GR_ChDB_0035 | Topotecan | 3uM | 6h | MAP4K3 | 0.04 | 0.34 |
| HT29_4_GR_ChDB_0035 | Topotecan | 3uM | 6h | AURKA | 0.04 | 0.44 |
| HT29_4_GR_ChDB_0035 | Topotecan | 3uM | 6h | RAD50 | 0.04 | 0.37 |
| HT29_4_GR_ChDB_0035 | Topotecan | 3uM | 6h | NRP1 | 0.03 | 0.43 |
| HT29_4_GR_ChDB_0035 | Topotecan | 3uM | 6h | CFLAR | 0.03 | 0.49 |
| HT29_4_GR_ChDB_0035 | Topotecan | 3uM | 6h | KDM5B | 0.03 | 0.45 |
| HT29_4_GR_ChDB_0035 | Topotecan | 3uM | 6h | STAT2 | 0.03 | 2.20 |
| HT29_4_GR_ChDB_0035 | Topotecan | 3uM | 6h | ATR | 0.03 | 0.47 |
| HT29_4_GR_ChDB_0035 | Topotecan | 3uM | 6h | ZNF672 | 0.03 | 2.56 |
| HT29_4_GR_ChDB_0035 | Topotecan | 3uM | 6h | PIK3C3 | 0.03 | 0.34 |
| HT29_4_GR_ChDB_0035 | Topotecan | 3uM | 6h | ZMIZ1 | 0.03 | 0.23 |
| HT29_4_GR_ChDB_0035 | Topotecan | 3uM | 6h | HEY1 | 0.03 | 2.34 |
| HT29_4_GR_ChDB_0035 | Topotecan | 3uM | 6h | AAK1 | 0.03 | 0.47 |
| HT29_4_GR_ChDB_0035 | Topotecan | 3uM | 6h | FBXO11 | 0.03 | 0.39 |
| HT29_4_GR_ChDB_0035 | Topotecan | 3uM | 6h | ROCK2 | 0.03 | 0.18 |
| HT29_4_GR_ChDB_0035 | Topotecan | 3uM | 6h | BTG1 | 0.03 | 2.38 |
| HT29_4_GR_ChDB_0035 | Topotecan | 3uM | 6h | ASCC3 | 0.03 | 0.33 |
| HT29_4_GR_ChDB_0035 | Topotecan | 3uM | 6h | MAPK8 | 0.03 | 0.37 |
| HT29_4_GR_ChDB_0035 | Topotecan | 3uM | 6h | CREBBP | 0.02 | 0.39 |
| HT29_4_GR_ChDB_0035 | Topotecan | 3uM | 6h | SMAD3 | 0.02 | 0.40 |
| HT29_4_GR_ChDB_0035 | Topotecan | 3uM | 6h | PKN2 | 0.02 | 0.27 |
| HT29_4_GR_ChDB_0035 | Topotecan | 3uM | 6h | VRK2 | 0.02 | 0.34 |
| HT29_4_GR_ChDB_0035 | Topotecan | 3uM | 6h | ZNF77 | 0.02 | 2.51 |
| HT29_4_GR_ChDB_0035 | Topotecan | 3uM | 6h | CENPE | 0.02 | 0.25 |
| HT29_4_GR_ChDB_0035 | Topotecan | 3uM | 6h | PRKCA | 0.02 | 0.49 |
| HT29_4_GR_ChDB_0035 | Topotecan | 3uM | 6h | NRIP1 | 0.02 | 0.27 |
| HT29_4_GR_ChDB_0035 | Topotecan | 3uM | 6h | BCR | 0.02 | 0.43 |
| HT29_4_GR_ChDB_0035 | Topotecan | 3uM | 6h | NEK7 | 0.02 | 0.32 |
| HT29_4_GR_ChDB_0035 | Topotecan | 3uM | 6h | ZAK | 0.02 | 0.47 |
| HT29_4_GR_ChDB_0035 | Topotecan | 3uM | 6h | MYCBP2 | 0.02 | 0.31 |
| HT29_4_GR_ChDB_0035 | Topotecan | 3uM | 6h | MAPKAP1 | 0.02 | 0.41 |
| HT29_4_GR_ChDB_0035 | Topotecan | 3uM | 6h | RNF19B | 0.02 | 2.52 |
| HT29_4_GR_ChDB_0035 | Topotecan | 3uM | 6h | GPR110 | 0.02 | 0.46 |
| HT29_4_GR_ChDB_0035 | Topotecan | 3uM | 6h | LGR5 | 0.02 | 0.10 |
| HT29_4_GR_ChDB_0035 | Topotecan | 3uM | 6h | MAP3K4 | 0.02 | 0.44 |
| HT29_4_GR_ChDB_0035 | Topotecan | 3uM | 6h | CDC42BPA | 0.02 | 0.39 |
| HT29_4_GR_ChDB_0035 | Topotecan | 3uM | 6h | PDS5A | 0.02 | 0.37 |
| HT29_4_GR_ChDB_0035 | Topotecan | 3uM | 6h | HSPA2 | 0.02 | 4.86 |
| HT29_4_GR_ChDB_0035 | Topotecan | 3uM | 6h | NRBF2 | 0.02 | 2.06 |
| HT29_4_GR_ChDB_0035 | Topotecan | 3uM | 6h | POLA1 | 0.02 | 0.27 |
| HT29_4_GR_ChDB_0035 | Topotecan | 3uM | 6h | DGKD | 0.01 | 0.39 |
| HT29_4_GR_ChDB_0035 | Topotecan | 3uM | 6h | RNF166 | 0.01 | 2.05 |
| HT29_4_GR_ChDB_0035 | Topotecan | 3uM | 6h | HMGCL | 0.01 | 2.06 |
| HT29_4_GR_ChDB_0035 | Topotecan | 3uM | 6h | PPARG | 0.01 | 0.18 |
| HT29_4_GR_ChDB_0035 | Topotecan | 3uM | 6h | RAD23B | 0.01 | 0.46 |
| HT29_4_GR_ChDB_0035 | Topotecan | 3uM | 6h | FBXO5 | 0.01 | 2.14 |
| HT29_4_GR_ChDB_0035 | Topotecan | 3uM | 6h | HEXIM1 | 0.01 | 6.72 |
| HT29_4_GR_ChDB_0035 | Topotecan | 3uM | 6h | CDK10 | 0.01 | 2.15 |
| HT29_4_GR_ChDB_0035 | Topotecan | 3uM | 6h | ZNF232 | 0.01 | 2.13 |
| HT29_4_GR_ChDB_0035 | Topotecan | 3uM | 6h | PUM2 | 0.01 | 0.43 |
| HT29_4_GR_ChDB_0035 | Topotecan | 3uM | 6h | USP6NL | 0.01 | 0.33 |
| HT29_4_GR_ChDB_0035 | Topotecan | 3uM | 6h | DYRK1A | 0.01 | 0.35 |
| HT29_4_GR_ChDB_0035 | Topotecan | 3uM | 6h | NAT1 | 0.01 | 3.40 |
| HT29_4_GR_ChDB_0035 | Topotecan | 3uM | 6h | CASK | 0.01 | 0.41 |
| HT29_4_GR_ChDB_0035 | Topotecan | 3uM | 6h | PEX13 | 0.01 | 2.62 |
| HT29_4_GR_ChDB_0035 | Topotecan | 3uM | 6h | ZKSCAN1 | 0.01 | 2.37 |
| HT29_4_GR_ChDB_0035 | Topotecan | 3uM | 6h | PAFAH1B1 | 0.01 | 0.40 |
| HT29_4_GR_ChDB_0035 | Topotecan | 3uM | 6h | NXF1 | 0.01 | 2.95 |
| HT29_4_GR_ChDB_0035 | Topotecan | 3uM | 6h | FYN | 0.01 | 0.29 |
| HT29_4_GR_ChDB_0035 | Topotecan | 3uM | 6h | VRK1 | 0.01 | 0.47 |
| HT29_4_GR_ChDB_0035 | Topotecan | 3uM | 6h | TFAP2A | 0.01 | 2.27 |
| HT29_4_GR_ChDB_0035 | Topotecan | 3uM | 6h | TJP1 | 0.01 | 0.42 |
| HT29_4_GR_ChDB_0035 | Topotecan | 3uM | 6h | AFF1 | 0.01 | 0.36 |
| HT29_4_GR_ChDB_0035 | Topotecan | 3uM | 6h | UVRAG | 0.01 | 0.47 |
| HT29_4_GR_ChDB_0035 | Topotecan | 3uM | 6h | GGA2 | 0.01 | 2.71 |
| HT29_4_GR_ChDB_0035 | Topotecan | 3uM | 6h | MAST2 | 0.01 | 0.36 |
| HT29_4_GR_ChDB_0035 | Topotecan | 3uM | 6h | SPDEF | 0.01 | 0.48 |
| HT29_4_GR_ChDB_0035 | Topotecan | 3uM | 6h | PHF13 | 0.01 | 2.39 |
| HT29_4_GR_ChDB_0035 | Topotecan | 3uM | 6h | ASF1B | 0.01 | 2.67 |
| HT29_4_GR_ChDB_0035 | Topotecan | 3uM | 6h | STK10 | 0.01 | 0.50 |
| HT29_4_GR_ChDB_0035 | Topotecan | 3uM | 6h | DENND1B | 0.01 | 0.29 |
| HT29_4_GR_ChDB_0035 | Topotecan | 3uM | 6h | TLK1 | 0.01 | 0.29 |
| HT29_4_GR_ChDB_0035 | Topotecan | 3uM | 6h | GNB1L | 0.01 | 0.29 |
| HT29_4_GR_ChDB_0035 | Topotecan | 3uM | 6h | MAP4K5 | 0.01 | 0.48 |
| HT29_4_GR_ChDB_0035 | Topotecan | 3uM | 6h | PAPOLA | 0.01 | 0.39 |
| HT29_4_GR_ChDB_0035 | Topotecan | 3uM | 6h | CDKN2C | 0.01 | 3.71 |
| HT29_4_GR_ChDB_0035 | Topotecan | 3uM | 6h | CAB39 | 0.01 | 0.49 |
| HT29_4_GR_ChDB_0035 | Topotecan | 3uM | 6h | PRKACB | 0.01 | 0.27 |
| HT29_4_GR_ChDB_0035 | Topotecan | 3uM | 6h | MPHOSPH9 | 0.01 | 0.45 |
| HT29_4_GR_ChDB_0035 | Topotecan | 3uM | 6h | LPAR2 | 0.01 | 3.17 |
| HT29_4_GR_ChDB_0035 | Topotecan | 3uM | 6h | XRCC4 | 0.01 | 0.23 |
| HT29_4_GR_ChDB_0035 | Topotecan | 3uM | 6h | RASSF1 | 0.01 | 2.28 |
| HT29_4_GR_ChDB_0035 | Topotecan | 3uM | 6h | MECOM | 0.01 | 0.37 |
| HT29_4_GR_ChDB_0035 | Topotecan | 3uM | 6h | USP32 | 0.01 | 0.32 |
| HT29_4_GR_ChDB_0035 | Topotecan | 3uM | 6h | ITCH | 0.01 | 0.39 |
| HT29_4_GR_ChDB_0035 | Topotecan | 3uM | 6h | GPR125 | 0.01 | 0.49 |
| HT29_4_GR_ChDB_0035 | Topotecan | 3uM | 6h | RYK | 0.01 | 0.36 |
| HT29_4_GR_ChDB_0035 | Topotecan | 3uM | 6h | SNX13 | 0.01 | 0.42 |
| HT29_4_GR_ChDB_0035 | Topotecan | 3uM | 6h | FOXO1 | 0.01 | 0.33 |
| HT29_4_GR_ChDB_0035 | Topotecan | 3uM | 6h | CDK2 | 0.01 | 2.26 |
| HT29_4_GR_ChDB_0035 | Topotecan | 3uM | 6h | PTEN | 0.01 | 0.46 |
| HT29_4_GR_ChDB_0035 | Topotecan | 3uM | 6h | RRAGC | 0.01 | 2.08 |
| HT29_4_GR_ChDB_0035 | Topotecan | 3uM | 6h | TAOK3 | 0.01 | 0.49 |
| HT29_4_GR_ChDB_0035 | Topotecan | 3uM | 6h | FOXJ2 | 0.01 | 2.59 |
| HT29_4_GR_ChDB_0035 | Topotecan | 3uM | 6h | DLX1 | 0.01 | 2.40 |
| HT29_4_GR_ChDB_0035 | Topotecan | 3uM | 6h | IGF2BP3 | 0.01 | 0.28 |
| HT29_4_GR_ChDB_0035 | Topotecan | 3uM | 6h | CCDC92 | 0.01 | 2.19 |
| HT29_4_GR_ChDB_0035 | Topotecan | 3uM | 6h | SLC7A1 | 0.01 | 0.50 |
| HT29_4_GR_ChDB_0035 | Topotecan | 3uM | 6h | TRRAP | 0.01 | 0.39 |
| HT29_4_GR_ChDB_0035 | Topotecan | 3uM | 6h | PPP3CA | 0.01 | 0.22 |
| HT29_4_GR_ChDB_0035 | Topotecan | 3uM | 6h | LAMB1 | 0.01 | 0.46 |
| HT29_4_GR_ChDB_0035 | Topotecan | 3uM | 6h | FBXL12 | 0.01 | 2.15 |
| HT29_4_GR_ChDB_0035 | Topotecan | 3uM | 6h | ANXA3 | 0.01 | 0.46 |
| HT29_4_GR_ChDB_0035 | Topotecan | 3uM | 6h | OAT | 0.01 | 2.25 |
| HT29_4_GR_ChDB_0035 | Topotecan | 3uM | 6h | HMGA2 | 0.01 | 0.16 |
| HT29_4_GR_ChDB_0035 | Topotecan | 3uM | 6h | GLUL | 0.01 | 2.11 |
| HT29_4_GR_ChDB_0035 | Topotecan | 3uM | 6h | SLC29A1 | 0.01 | 2.07 |
| HT29_4_GR_ChDB_0035 | Topotecan | 3uM | 6h | PDGFC | 0.01 | 0.21 |
| HT29_4_GR_ChDB_0035 | Topotecan | 3uM | 6h | OSR2 | 0.01 | 2.93 |
| HT29_4_GR_ChDB_0035 | Topotecan | 3uM | 6h | EHF | 0.01 | 0.44 |
| HT29_4_GR_ChDB_0035 | Topotecan | 3uM | 6h | HK2 | 0.01 | 0.48 |
| HT29_4_GR_ChDB_0035 | Topotecan | 3uM | 6h | SCNN1A | 0.01 | 0.43 |
| HT29_4_GR_ChDB_0035 | Topotecan | 3uM | 6h | XPO7 | 0.01 | 0.49 |
| HT29_4_GR_ChDB_0035 | Topotecan | 3uM | 6h | RFWD2 | 0.01 | 0.21 |
| HT29_4_GR_ChDB_0035 | Topotecan | 3uM | 6h | IFIT5 | 0.01 | 3.04 |
| HT29_4_GR_ChDB_0035 | Topotecan | 3uM | 6h | PTGS2 | 0.01 | 3.49 |
| HT29_4_GR_ChDB_0035 | Topotecan | 3uM | 6h | ATF2 | 0.01 | 0.43 |
| HT29_4_GR_ChDB_0035 | Topotecan | 3uM | 6h | AP1S2 | 0.01 | 2.37 |
| HT29_4_GR_ChDB_0035 | Topotecan | 3uM | 6h | NVL | 0.01 | 0.38 |
| HT29_4_GR_ChDB_0035 | Topotecan | 3uM | 6h | PPP2R5E | 0.01 | 0.37 |
| HT29_4_GR_ChDB_0035 | Topotecan | 3uM | 6h | MLLT3 | 0.01 | 0.31 |
| HT29_4_GR_ChDB_0035 | Topotecan | 3uM | 6h | MKNK2 | 0.01 | 2.52 |
| HT29_4_GR_ChDB_0035 | Topotecan | 3uM | 6h | PBRM1 | 0.01 | 0.30 |
| HT29_4_GR_ChDB_0035 | Topotecan | 3uM | 6h | EPB41L2 | 0.01 | 0.47 |
| HT29_4_GR_ChDB_0035 | Topotecan | 3uM | 6h | DLG1 | 0.01 | 0.21 |
| HT29_4_GR_ChDB_0035 | Topotecan | 3uM | 6h | KAT6B | 0.00 | 0.21 |
| HT29_4_GR_ChDB_0035 | Topotecan | 3uM | 6h | ADCY3 | 0.00 | 0.48 |
| HT29_4_GR_ChDB_0035 | Topotecan | 3uM | 6h | MAPK1 | 0.00 | 0.49 |
| HT29_4_GR_ChDB_0035 | Topotecan | 3uM | 6h | ABCC1 | 0.00 | 0.27 |
| HT29_4_GR_ChDB_0035 | Topotecan | 3uM | 6h | EXOC6 | 0.00 | 0.25 |
| HT29_4_GR_ChDB_0035 | Topotecan | 3uM | 6h | MSX2 | 0.00 | 2.38 |
| HT29_4_GR_ChDB_0035 | Topotecan | 3uM | 6h | CAMLG | 0.00 | 2.49 |
| HT29_4_GR_ChDB_0035 | Topotecan | 3uM | 6h | GOLIM4 | 0.00 | 0.48 |
| HT29_4_GR_ChDB_0035 | Topotecan | 3uM | 6h | NOTCH2 | 0.00 | 0.49 |
| HT29_4_GR_ChDB_0035 | Topotecan | 3uM | 6h | XPC | 0.00 | 2.10 |
| HT29_4_GR_ChDB_0035 | Topotecan | 3uM | 6h | YAP1 | 0.00 | 0.37 |
| HT29_4_GR_ChDB_0035 | Topotecan | 3uM | 6h | SAT1 | 0.00 | 2.35 |
| HT29_4_GR_ChDB_0035 | Topotecan | 3uM | 6h | ERBB2IP | 0.00 | 0.25 |
| HT29_4_GR_ChDB_0035 | Topotecan | 3uM | 6h | MAP4K4 | 0.00 | 0.37 |
| HT29_4_GR_ChDB_0035 | Topotecan | 3uM | 6h | TNFRSF21 | 0.00 | 0.33 |
| HT29_4_GR_ChDB_0035 | Topotecan | 3uM | 6h | HS2ST1 | 0.00 | 0.34 |
| HT29_4_GR_ChDB_0035 | Topotecan | 3uM | 6h | PSIP1 | 0.00 | 0.50 |
| HT29_4_GR_ChDB_0035 | Topotecan | 3uM | 6h | FBXW11 | 0.00 | 0.48 |
| HT29_4_GR_ChDB_0035 | Topotecan | 3uM | 6h | CDC25A | 0.00 | 2.03 |
| HT29_4_GR_ChDB_0035 | Topotecan | 3uM | 6h | RAB3GAP1 | 0.00 | 0.36 |
| HT29_4_GR_ChDB_0035 | Topotecan | 3uM | 6h | SIK3 | 0.00 | 0.30 |
| HT29_4_GR_ChDB_0035 | Topotecan | 3uM | 6h | NKIRAS2 | 0.00 | 2.00 |
| HT29_4_GR_ChDB_0035 | Topotecan | 3uM | 6h | LGR4 | 0.00 | 0.26 |
| HT29_4_GR_ChDB_0035 | Topotecan | 3uM | 6h | FKBP14 | 0.00 | 3.24 |
| HT29_4_GR_ChDB_0035 | Topotecan | 3uM | 6h | UBE2K | 0.00 | 0.43 |
| HT29_4_GR_ChDB_0035 | Topotecan | 3uM | 6h | WNT11 | 0.00 | 0.23 |
| HT29_4_GR_ChDB_0035 | Topotecan | 3uM | 6h | PDS5B | 0.00 | 0.27 |
| HT29_4_GR_ChDB_0035 | Topotecan | 3uM | 6h | SENP6 | 0.00 | 0.50 |
| HT29_4_GR_ChDB_0035 | Topotecan | 3uM | 6h | SFMBT1 | 0.00 | 0.36 |
| HT29_4_GR_ChDB_0035 | Topotecan | 3uM | 6h | MYO10 | 0.00 | 0.29 |
| HT29_4_GR_ChDB_0035 | Topotecan | 3uM | 6h | WNK1 | 0.00 | 0.37 |
| HT29_4_GR_ChDB_0035 | Topotecan | 3uM | 6h | DLGAP5 | 0.00 | 0.38 |
| HT29_4_GR_ChDB_0035 | Topotecan | 3uM | 6h | METAP2 | 0.00 | 0.40 |
| HT29_4_GR_ChDB_0035 | Topotecan | 3uM | 6h | ACSL4 | 0.00 | 0.40 |
| HT29_4_GR_ChDB_0035 | Topotecan | 3uM | 6h | PTPN2 | 0.00 | 0.42 |
| HT29_4_GR_ChDB_0035 | Topotecan | 3uM | 6h | FEN1 | 0.00 | 2.33 |
| HT29_4_GR_ChDB_0035 | Topotecan | 3uM | 6h | NEDD4 | 0.00 | 0.47 |
| HT29_4_GR_ChDB_0035 | Topotecan | 3uM | 6h | BTG3 | 0.00 | 2.30 |
| HT29_4_GR_ChDB_0035 | Topotecan | 3uM | 6h | B3GAT3 | 0.00 | 2.43 |
| HT29_4_GR_ChDB_0035 | Topotecan | 3uM | 6h | PPIG | 0.00 | 0.48 |
| HT29_4_GR_ChDB_0035 | Topotecan | 3uM | 6h | CTSL1 | 0.00 | 2.03 |
| HT29_4_GR_ChDB_0035 | Topotecan | 3uM | 6h | UTP18 | 0.00 | 0.39 |
| HT29_4_GR_ChDB_0035 | Topotecan | 3uM | 6h | FOXJ3 | 0.00 | 0.33 |
| HT29_4_GR_ChDB_0035 | Topotecan | 3uM | 6h | TRAFD1 | 0.00 | 2.03 |
| HT29_4_GR_ChDB_0035 | Topotecan | 3uM | 6h | ITSN1 | 0.00 | 0.43 |
| HT29_4_GR_ChDB_0035 | Topotecan | 3uM | 6h | ZMYM2 | 0.00 | 0.45 |
| HT29_4_GR_ChDB_0035 | Topotecan | 3uM | 6h | UBE3A | 0.00 | 0.44 |
| HT29_4_GR_ChDB_0035 | Topotecan | 3uM | 6h | DEPDC7 | 0.00 | 2.63 |
| HT29_4_GR_ChDB_0035 | Topotecan | 3uM | 6h | NR4A2 | 0.00 | 2.17 |
| HT29_4_GR_ChDB_0035 | Topotecan | 3uM | 6h | RNF8 | 0.00 | 2.15 |
| HT29_4_GR_ChDB_0035 | Topotecan | 3uM | 6h | BAD | 0.00 | 2.26 |
| HT29_4_GR_ChDB_0035 | Topotecan | 3uM | 6h | ZBED1 | 0.00 | 2.06 |
| HT29_4_GR_ChDB_0035 | Topotecan | 3uM | 6h | ABHD2 | 0.00 | 0.48 |
| HT29_4_GR_ChDB_0035 | Topotecan | 3uM | 6h | B3GNT1 | 0.00 | 2.05 |
| HT29_4_GR_ChDB_0035 | Topotecan | 3uM | 6h | POLR2D | 0.00 | 2.00 |
| HT29_4_GR_ChDB_0035 | Topotecan | 3uM | 6h | OGG1 | 0.00 | 2.76 |
| HT29_4_GR_ChDB_0035 | Topotecan | 3uM | 6h | HMMR | 0.00 | 0.35 |
| HT29_4_GR_ChDB_0035 | Topotecan | 3uM | 6h | ING2 | 0.00 | 2.69 |
| HT29_4_GR_ChDB_0035 | Topotecan | 3uM | 6h | MED28 | 0.00 | 2.47 |
| HT29_4_GR_ChDB_0035 | Topotecan | 3uM | 6h | CD83 | 0.00 | 2.70 |
| HT29_4_GR_ChDB_0035 | Topotecan | 3uM | 6h | ZNF697 | 0.00 | 2.22 |
| HT29_4_GR_ChDB_0035 | Topotecan | 3uM | 6h | HMOX1 | 0.00 | 0.48 |
| HT29_4_GR_ChDB_0035 | Topotecan | 3uM | 6h | TUBB2A | 0.00 | 2.29 |
| HT29_4_GR_ChDB_0035 | Topotecan | 3uM | 6h | ABCC4 | 0.00 | 0.34 |
| HT29_4_GR_ChDB_0035 | Topotecan | 3uM | 6h | DCLRE1B | 0.00 | 2.88 |
| HT29_4_GR_ChDB_0035 | Topotecan | 3uM | 6h | PRR15L | 0.00 | 0.35 |
| HT29_4_GR_ChDB_0035 | Topotecan | 3uM | 6h | FBXO3 | 0.00 | 2.11 |
| HT29_4_GR_ChDB_0035 | Topotecan | 3uM | 6h | KITLG | 0.00 | 0.26 |
| HT29_4_GR_ChDB_0035 | Topotecan | 3uM | 6h | DTX3L | 0.00 | 2.35 |
| HT29_4_GR_ChDB_0035 | Topotecan | 3uM | 6h | SATB1 | 0.00 | 0.47 |
| HT29_4_GR_ChDB_0035 | Topotecan | 3uM | 6h | NFIB | 0.00 | 0.24 |
| HT29_4_GR_ChDB_0035 | Topotecan | 3uM | 6h | GPR126 | 0.00 | 0.40 |
| HT29_4_GR_ChDB_0035 | Topotecan | 3uM | 6h | HNF1B | 0.00 | 0.44 |
| HT29_4_GR_ChDB_0035 | Topotecan | 3uM | 6h | SFN | 0.00 | 2.20 |
| HT29_4_GR_ChDB_0035 | Topotecan | 3uM | 6h | NFAT5 | 0.00 | 0.23 |
| HT29_4_GR_ChDB_0035 | Topotecan | 3uM | 6h | GTF2F2 | 0.00 | 0.42 |
| HT29_4_GR_ChDB_0035 | Topotecan | 3uM | 6h | RAB11A | 0.00 | 0.46 |
| HT29_4_GR_ChDB_0035 | Topotecan | 3uM | 6h | RNGTT | 0.00 | 0.38 |
| HT29_4_GR_ChDB_0035 | Topotecan | 3uM | 6h | PRMT2 | 0.00 | 2.50 |
| HT29_4_GR_ChDB_0035 | Topotecan | 3uM | 6h | ZNF277 | 0.00 | 0.43 |
| HT29_4_GR_ChDB_0035 | Topotecan | 3uM | 6h | NT5C2 | 0.00 | 0.48 |
| HT29_4_GR_ChDB_0035 | Topotecan | 3uM | 6h | NIPBL | 0.00 | 0.36 |
| HT29_4_GR_ChDB_0035 | Topotecan | 3uM | 6h | ELOVL6 | 0.00 | 0.33 |
| HT29_4_GR_ChDB_0035 | Topotecan | 3uM | 6h | PLXNA2 | 0.00 | 0.41 |
| HT29_4_GR_ChDB_0035 | Topotecan | 3uM | 6h | TRIM2 | 0.00 | 0.43 |
| HT29_4_GR_ChDB_0035 | Topotecan | 3uM | 6h | RBMS1 | 0.00 | 0.25 |
| HT29_4_GR_ChDB_0035 | Topotecan | 3uM | 6h | PKDCC | 0.00 | 0.25 |
| HT29_4_GR_ChDB_0035 | Topotecan | 3uM | 6h | KLF5 | 0.00 | 0.46 |
| HT29_4_GR_ChDB_0035 | Topotecan | 3uM | 6h | SLC38A1 | 0.00 | 0.38 |
| HT29_4_GR_ChDB_0035 | Topotecan | 3uM | 6h | MTA3 | 0.00 | 0.46 |
| HT29_4_GR_ChDB_0035 | Topotecan | 3uM | 6h | NUMB | 0.00 | 0.43 |
| HT29_4_GR_ChDB_0035 | Topotecan | 3uM | 6h | AKAP9 | 0.00 | 0.42 |
| HT29_4_GR_ChDB_0035 | Topotecan | 3uM | 6h | DDX10 | 0.00 | 0.16 |
| HT29_4_GR_ChDB_0035 | Topotecan | 3uM | 6h | RAI14 | 0.00 | 0.45 |
| HT29_4_GR_ChDB_0035 | Topotecan | 3uM | 6h | POLR3B | 0.00 | 0.45 |
| HT29_4_GR_ChDB_0035 | Topotecan | 3uM | 6h | E2F5 | 0.00 | 0.44 |
| HT29_4_GR_ChDB_0035 | Topotecan | 3uM | 6h | THADA | 0.00 | 0.36 |
| HT29_4_GR_ChDB_0035 | Topotecan | 3uM | 6h | IDE | 0.00 | 0.49 |
| HT29_4_GR_ChDB_0035 | Topotecan | 3uM | 6h | EIF4E | 0.00 | 0.37 |
| HT29_4_GR_ChDB_0035 | Topotecan | 3uM | 6h | MEIS2 | 0.00 | 0.44 |
| HT29_4_GR_ChDB_0035 | Topotecan | 3uM | 6h | PMM2 | 0.00 | 0.37 |
| HT29_4_GR_ChDB_0035 | Topotecan | 3uM | 6h | TASP1 | 0.00 | 0.33 |
| HT29_4_GR_ChDB_0035 | Topotecan | 3uM | 6h | SLC7A11 | 0.00 | 0.39 |
| HT29_4_GR_ChDB_0035 | Topotecan | 3uM | 6h | NT5E | 0.00 | 0.46 |
| HT29_4_GR_ChDB_0035 | Topotecan | 3uM | 6h | FAT1 | 0.00 | 0.45 |
| HT29_4_GR_ChDB_0035 | Topotecan | 3uM | 6h | RHOD | 0.00 | 2.07 |
| HT29_4_GR_ChDB_0035 | Topotecan | 3uM | 6h | SLC2A1 | 0.00 | 0.41 |
| HT29_4_GR_ChDB_0035 | Topotecan | 3uM | 6h | ARID4B | 0.00 | 0.49 |
| HT29_4_GR_ChDB_0035 | Topotecan | 3uM | 6h | CD97 | 0.00 | 0.47 |
| HT29_4_GR_ChDB_0035 | Topotecan | 3uM | 6h | BIRC6 | 0.00 | 0.22 |
| HT29_4_GR_ChDB_0035 | Topotecan | 3uM | 6h | POU2F1 | 0.00 | 0.45 |
| HT29_4_GR_ChDB_0035 | Topotecan | 3uM | 6h | E2F8 | 0.00 | 3.10 |
| HT29_4_GR_ChDB_0035 | Topotecan | 3uM | 6h | ASAP2 | 0.00 | 0.22 |
| HT29_4_GR_ChDB_0035 | Topotecan | 3uM | 6h | EFNB2 | 0.00 | 0.39 |
| HT29_4_GR_ChDB_0035 | Topotecan | 3uM | 6h | NF1 | 0.00 | 0.37 |
| HT29_4_GR_ChDB_0035 | Topotecan | 3uM | 6h | ID1 | 0.00 | 0.30 |
| HT29_4_GR_ChDB_0035 | Topotecan | 3uM | 6h | MAP7 | 0.00 | 0.38 |
| HT29_4_GR_ChDB_0035 | Topotecan | 3uM | 6h | ARHGAP35 | 0.00 | 0.42 |
| HT29_4_GR_ChDB_0035 | Topotecan | 3uM | 6h | PAK2 | 0.00 | 0.37 |
| HT29_4_GR_ChDB_0035 | Topotecan | 3uM | 6h | NR2C2 | 0.00 | 0.37 |
| HT29_4_GR_ChDB_0035 | Topotecan | 3uM | 6h | TFPI | 0.00 | 0.39 |
| HT29_4_GR_ChDB_0035 | Topotecan | 3uM | 6h | COBL | 0.00 | 0.36 |
| HT29_4_GR_ChDB_0035 | Topotecan | 3uM | 6h | UIMC1 | 0.00 | 0.40 |
| HT29_4_GR_ChDB_0035 | Topotecan | 3uM | 6h | CTBP2 | 0.00 | 0.24 |
| HT29_4_GR_ChDB_0035 | Topotecan | 3uM | 6h | STK3 | 0.00 | 0.13 |
| HT29_4_GR_ChDB_0035 | Topotecan | 3uM | 6h | GPR39 | 0.00 | 0.48 |
| HT29_4_GR_ChDB_0035 | Topotecan | 3uM | 6h | ITGA2 | 0.00 | 0.29 |
| HT29_4_GR_ChDB_0035 | Topotecan | 3uM | 6h | TSEN2 | 0.00 | 0.32 |
| HT29_4_GR_ChDB_0035 | Topotecan | 3uM | 6h | PTPRK | 0.00 | 0.14 |
| HT29_4_GR_ChDB_0035 | Topotecan | 3uM | 6h | ADSS | 0.00 | 0.46 |
| HT29_4_GR_ChDB_0035 | Topotecan | 3uM | 6h | TP53RK | 0.00 | 2.29 |
| HT29_4_GR_ChDB_0035 | Topotecan | 3uM | 6h | MAGI3 | 0.00 | 0.27 |
| HT29_4_GR_ChDB_0035 | Topotecan | 3uM | 6h | HNF4G | 0.00 | 0.48 |
| HT29_4_GR_ChDB_0035 | Topotecan | 3uM | 6h | CRCP | 0.00 | 0.49 |
| HT29_4_GR_ChDB_0035 | Topotecan | 3uM | 6h | SMAD6 | 0.00 | 0.37 |
| HT29_4_GR_ChDB_0035 | Topotecan | 3uM | 6h | TRPM7 | 0.00 | 0.37 |
| HT29_4_GR_ChDB_0035 | Topotecan | 3uM | 6h | AMPD2 | 0.00 | 2.37 |
| HT29_4_GR_ChDB_0035 | Topotecan | 3uM | 6h | LMO4 | 0.00 | 0.47 |
| HT29_4_GR_ChDB_0035 | Topotecan | 3uM | 6h | ARHGEF12 | 0.00 | 0.38 |
| HT29_4_GR_ChDB_0035 | Topotecan | 3uM | 6h | PREP | 0.00 | 0.43 |
| HT29_4_GR_ChDB_0035 | Topotecan | 3uM | 6h | PCM1 | 0.00 | 0.45 |
| HT29_4_GR_ChDB_0035 | Topotecan | 3uM | 6h | CLOCK | 0.00 | 0.36 |
| HT29_4_GR_ChDB_0035 | Topotecan | 3uM | 6h | TNIK | 0.00 | 0.18 |
| HT29_4_GR_ChDB_0035 | Topotecan | 3uM | 6h | TCIRG1 | 0.00 | 2.05 |
| HT29_4_GR_ChDB_0035 | Topotecan | 3uM | 6h | SMURF2 | 0.00 | 0.47 |
| HT29_4_GR_ChDB_0035 | Topotecan | 3uM | 6h | CDK9 | 0.00 | 2.05 |
| HT29_4_GR_ChDB_0035 | Topotecan | 3uM | 6h | LDLR | 0.00 | 0.41 |
| HT29_4_GR_ChDB_0035 | Topotecan | 3uM | 6h | OPA1 | 0.00 | 0.41 |
| HT29_4_GR_ChDB_0035 | Topotecan | 3uM | 6h | IGFBP4 | 0.00 | 0.41 |
| HT29_4_GR_ChDB_0035 | Topotecan | 3uM | 6h | KLF6 | 0.00 | 0.36 |
| HT29_4_GR_ChDB_0035 | Topotecan | 3uM | 6h | DAPK1 | 0.00 | 0.29 |
| HT29_4_GR_ChDB_0035 | Topotecan | 3uM | 6h | H2AFX | 0.00 | 2.13 |
| HT29_4_GR_ChDB_0035 | Topotecan | 3uM | 6h | MTAP | 0.00 | 0.29 |
| HT29_4_GR_ChDB_0035 | Topotecan | 3uM | 6h | BRCA2 | 0.00 | 0.48 |
| HT29_4_GR_ChDB_0035 | Topotecan | 3uM | 6h | ATRX | 0.00 | 0.38 |
| HT29_4_GR_ChDB_0035 | Topotecan | 3uM | 6h | SGK223 | 0.00 | 0.45 |
| HT29_4_GR_ChDB_0035 | Topotecan | 3uM | 6h | BUB1 | 0.00 | 0.50 |
| HT29_4_GR_ChDB_0035 | Topotecan | 3uM | 6h | SPECC1L | 0.00 | 0.41 |
| HT29_4_GR_ChDB_0035 | Topotecan | 3uM | 6h | CDK17 | 0.00 | 0.50 |
| HT29_4_GR_ChDB_0035 | Topotecan | 3uM | 6h | GNAQ | 0.00 | 0.29 |
| HT29_4_GR_ChDB_0035 | Topotecan | 3uM | 6h | TRIM24 | 0.00 | 0.48 |
| HT29_4_GR_ChDB_0035 | Topotecan | 3uM | 6h | PEAK1 | 0.00 | 0.38 |
| HT29_4_GR_ChDB_0035 | Topotecan | 3uM | 6h | HSBP1 | 0.00 | 2.28 |
| HT29_4_GR_ChDB_0035 | Topotecan | 3uM | 6h | CDK13 | 0.00 | 0.49 |
| HT29_4_GR_ChDB_0035 | Topotecan | 3uM | 6h | PRMT3 | 0.00 | 0.44 |
| HT29_4_GR_ChDB_0035 | Topotecan | 3uM | 6h | PTPN12 | 0.00 | 0.36 |
| HT29_4_GR_ChDB_0035 | Topotecan | 3uM | 6h | NAA25 | 0.00 | 0.36 |
| HT29_4_GR_ChDB_0035 | Topotecan | 3uM | 6h | SHB | 0.00 | 0.37 |
| HT29_4_GR_ChDB_0035 | Topotecan | 3uM | 6h | MTX2 | 0.00 | 0.42 |
| HT29_4_GR_ChDB_0035 | Topotecan | 3uM | 6h | XPR1 | 0.00 | 0.49 |
| HT29_4_GR_ChDB_0035 | Topotecan | 3uM | 6h | MAD2L1BP | 0.00 | 2.73 |
| HT29_4_GR_ChDB_0035 | Topotecan | 3uM | 6h | MFHAS1 | 0.00 | 0.43 |
| HT29_4_GR_ChDB_0035 | Topotecan | 3uM | 6h | ATP5S | 0.00 | 0.49 |
| HT29_4_GR_ChDB_0035 | Topotecan | 3uM | 6h | SPTLC2 | 0.00 | 0.46 |
| HT29_4_GR_ChDB_0035 | Topotecan | 3uM | 6h | SOS1 | 0.00 | 0.35 |
| HT29_4_GR_ChDB_0035 | Topotecan | 3uM | 6h | MED4 | 0.00 | 0.50 |
| HT29_4_GR_ChDB_0035 | Topotecan | 3uM | 6h | CHST12 | 0.00 | 2.32 |
| HT29_4_GR_ChDB_0035 | Topotecan | 3uM | 6h | STAM2 | 0.00 | 0.46 |
| HT29_4_GR_ChDB_0035 | Topotecan | 3uM | 6h | TRIM13 | 0.00 | 2.41 |
| HT29_4_GR_ChDB_0035 | Topotecan | 3uM | 6h | UBAC2 | 0.00 | 0.30 |
| HT29_4_GR_ChDB_0035 | Topotecan | 3uM | 6h | MEF2A | 0.00 | 0.48 |
| HT29_4_GR_ChDB_0035 | Topotecan | 3uM | 6h | OPN3 | 0.00 | 3.27 |
| HT29_4_GR_ChDB_0035 | Topotecan | 3uM | 6h | ZBTB9 | 0.00 | 2.05 |
| HT29_4_GR_ChDB_0035 | Topotecan | 3uM | 6h | GATAD2A | 0.00 | 0.48 |
| HT29_4_GR_ChDB_0035 | Topotecan | 3uM | 6h | PSRC1 | 0.00 | 0.48 |
| HT29_4_GR_ChDB_0035 | Topotecan | 3uM | 6h | KIF14 | 0.00 | 0.39 |
| HT29_4_GR_ChDB_0035 | Topotecan | 3uM | 6h | CAV1 | 0.00 | 0.49 |
| HT29_4_GR_ChDB_0035 | Topotecan | 3uM | 6h | SMURF1 | 0.00 | 0.40 |
| HT29_4_GR_ChDB_0035 | Topotecan | 3uM | 6h | PRKX | 0.00 | 0.48 |
| HT29_4_GR_ChDB_0035 | Topotecan | 3uM | 6h | SP3 | 0.00 | 0.44 |
| HT29_4_GR_ChDB_0035 | Topotecan | 3uM | 6h | GPSM2 | 0.00 | 0.37 |
| HT29_4_GR_ChDB_0035 | Topotecan | 3uM | 6h | ATXN1 | 0.00 | 0.43 |
| HT29_4_GR_ChDB_0035 | Topotecan | 3uM | 6h | CHCHD7 | 0.00 | 2.68 |
| HT29_4_GR_ChDB_0035 | Topotecan | 3uM | 6h | UBE3C | 0.00 | 0.47 |
| HT29_4_GR_ChDB_0035 | Topotecan | 3uM | 6h | GNA12 | 0.00 | 0.35 |
| HT29_4_GR_ChDB_0035 | Topotecan | 3uM | 6h | RCOR1 | 0.00 | 0.45 |
| HT29_4_GR_ChDB_0035 | Topotecan | 3uM | 6h | MLLT10 | 0.00 | 0.37 |
| HT29_4_GR_ChDB_0035 | Topotecan | 3uM | 6h | SUCNR1 | 0.00 | 0.46 |
| HT29_4_GR_ChDB_0035 | Topotecan | 3uM | 6h | MNAT1 | 0.00 | 0.31 |
| HT29_4_GR_ChDB_0035 | Topotecan | 3uM | 6h | ATP11B | 0.00 | 0.40 |
| HT29_4_GR_ChDB_0035 | Topotecan | 3uM | 6h | FDXR | 0.00 | 2.34 |
| HT29_4_GR_ChDB_0035 | Topotecan | 3uM | 6h | SLC35A3 | 0.00 | 0.46 |
| HT29_4_GR_ChDB_0035 | Topotecan | 3uM | 6h | GFPT1 | 0.00 | 0.31 |
| HT29_4_GR_ChDB_0035 | Topotecan | 3uM | 6h | LRRC16A | 0.00 | 0.49 |
| HT29_4_GR_ChDB_0035 | Topotecan | 3uM | 6h | GPRC5A | 0.00 | 0.36 |
| HT29_4_GR_ChDB_0035 | Topotecan | 3uM | 6h | KDM4B | 0.00 | 0.47 |
| HT29_4_GR_ChDB_0035 | Topotecan | 3uM | 6h | ARID5B | 0.00 | 0.21 |
| HT29_4_GR_ChDB_0035 | Topotecan | 3uM | 6h | FAF1 | 0.00 | 0.24 |
| HT29_4_GR_ChDB_0035 | Topotecan | 3uM | 6h | DUSP3 | 0.00 | 2.43 |
| HT29_4_GR_ChDB_0035 | Topotecan | 3uM | 6h | TRIO | 0.00 | 0.33 |
| HT29_4_GR_ChDB_0035 | Topotecan | 3uM | 6h | LRP6 | 0.00 | 0.49 |
| HT29_4_GR_ChDB_0035 | Topotecan | 3uM | 6h | SPRED2 | 0.00 | 0.37 |
| HT29_4_GR_ChDB_0035 | Topotecan | 3uM | 6h | SMARCA2 | 0.00 | 0.44 |
| HT29_4_GR_ChDB_0035 | Topotecan | 3uM | 6h | DROSHA | 0.00 | 0.44 |
| HT29_4_GR_ChDB_0035 | Topotecan | 3uM | 6h | PRKCH | 0.00 | 0.42 |
| HT29_4_GR_ChDB_0036 | Doxorubicin | 3uM | 6h | ADM | 1.00 | 5.97 |
| HT29_4_GR_ChDB_0036 | Doxorubicin | 3uM | 6h | MYC | 0.40 | 0.30 |
| HT29_4_GR_ChDB_0036 | Doxorubicin | 3uM | 6h | JUN | 0.20 | 0.41 |
| HT29_4_GR_ChDB_0036 | Doxorubicin | 3uM | 6h | EGFR | 0.15 | 0.42 |
| HT29_4_GR_ChDB_0036 | Doxorubicin | 3uM | 6h | BUB1B | 0.10 | 0.11 |
| HT29_4_GR_ChDB_0036 | Doxorubicin | 3uM | 6h | IGF1R | 0.09 | 0.45 |
| HT29_4_GR_ChDB_0036 | Doxorubicin | 3uM | 6h | HEXIM1 | 0.05 | 4.66 |
| HT29_4_GR_ChDB_0036 | Doxorubicin | 3uM | 6h | TRIB3 | 0.04 | 0.31 |
| HT29_4_GR_ChDB_0036 | Doxorubicin | 3uM | 6h | HBEGF | 0.04 | 3.27 |
| HT29_4_GR_ChDB_0036 | Doxorubicin | 3uM | 6h | HERPUD1 | 0.04 | 0.23 |
| HT29_4_GR_ChDB_0036 | Doxorubicin | 3uM | 6h | APOM | 0.03 | 2.11 |
| HT29_4_GR_ChDB_0036 | Doxorubicin | 3uM | 6h | CDKN2C | 0.02 | 5.10 |
| HT29_4_GR_ChDB_0036 | Doxorubicin | 3uM | 6h | RNF146 | 0.02 | 2.95 |
| HT29_4_GR_ChDB_0036 | Doxorubicin | 3uM | 6h | CTGF | 0.02 | 4.15 |
| HT29_4_GR_ChDB_0036 | Doxorubicin | 3uM | 6h | CFLAR | 0.02 | 0.27 |
| HT29_4_GR_ChDB_0036 | Doxorubicin | 3uM | 6h | EGR1 | 0.02 | 4.31 |
| HT29_4_GR_ChDB_0036 | Doxorubicin | 3uM | 6h | IRS1 | 0.02 | 0.24 |
| HT29_4_GR_ChDB_0036 | Doxorubicin | 3uM | 6h | PARP2 | 0.01 | 0.26 |
| HT29_4_GR_ChDB_0036 | Doxorubicin | 3uM | 6h | NAT1 | 0.01 | 3.13 |
| HT29_4_GR_ChDB_0036 | Doxorubicin | 3uM | 6h | ATF4 | 0.01 | 0.17 |
| HT29_4_GR_ChDB_0036 | Doxorubicin | 3uM | 6h | HOXB5 | 0.01 | 2.44 |
| HT29_4_GR_ChDB_0036 | Doxorubicin | 3uM | 6h | MAPKAP1 | 0.01 | 0.41 |
| HT29_4_GR_ChDB_0036 | Doxorubicin | 3uM | 6h | PAK2 | 0.01 | 0.22 |
| HT29_4_GR_ChDB_0036 | Doxorubicin | 3uM | 6h | CTCF | 0.01 | 0.48 |
| HT29_4_GR_ChDB_0036 | Doxorubicin | 3uM | 6h | OPN3 | 0.01 | 2.13 |
| HT29_4_GR_ChDB_0036 | Doxorubicin | 3uM | 6h | PPARG | 0.01 | 0.36 |
| HT29_4_GR_ChDB_0036 | Doxorubicin | 3uM | 6h | NR2F2 | 0.01 | 0.41 |
| HT29_4_GR_ChDB_0036 | Doxorubicin | 3uM | 6h | PLXNA1 | 0.01 | 0.35 |
| HT29_4_GR_ChDB_0036 | Doxorubicin | 3uM | 6h | CTSL2 | 0.01 | 3.05 |
| HT29_4_GR_ChDB_0036 | Doxorubicin | 3uM | 6h | RSPRY1 | 0.01 | 2.39 |
| HT29_4_GR_ChDB_0036 | Doxorubicin | 3uM | 6h | CANT1 | 0.01 | 0.34 |
| HT29_4_GR_ChDB_0036 | Doxorubicin | 3uM | 6h | BCL6 | 0.01 | 3.22 |
| HT29_4_GR_ChDB_0036 | Doxorubicin | 3uM | 6h | SRSF3 | 0.01 | 0.48 |
| HT29_4_GR_ChDB_0036 | Doxorubicin | 3uM | 6h | RPS6KA3 | 0.01 | 0.47 |
| HT29_4_GR_ChDB_0036 | Doxorubicin | 3uM | 6h | SLC11A2 | 0.01 | 0.44 |
| HT29_4_GR_ChDB_0036 | Doxorubicin | 3uM | 6h | ID2 | 0.00 | 3.11 |
| HT29_4_GR_ChDB_0036 | Doxorubicin | 3uM | 6h | PSRC1 | 0.00 | 0.30 |
| HT29_4_GR_ChDB_0036 | Doxorubicin | 3uM | 6h | DNAJB9 | 0.00 | 2.22 |
| HT29_4_GR_ChDB_0036 | Doxorubicin | 3uM | 6h | TGFBR2 | 0.00 | 0.25 |
| HT29_4_GR_ChDB_0036 | Doxorubicin | 3uM | 6h | GRK6 | 0.00 | 0.34 |
| HT29_4_GR_ChDB_0036 | Doxorubicin | 3uM | 6h | TCF7L2 | 0.00 | 0.39 |
| HT29_4_GR_ChDB_0036 | Doxorubicin | 3uM | 6h | NFIC | 0.00 | 0.38 |
| HT29_4_GR_ChDB_0036 | Doxorubicin | 3uM | 6h | PRKCZ | 0.00 | 0.31 |
| HT29_4_GR_ChDB_0036 | Doxorubicin | 3uM | 6h | XRCC4 | 0.00 | 0.46 |
| HT29_4_GR_ChDB_0036 | Doxorubicin | 3uM | 6h | ZFX | 0.00 | 0.49 |
| HT29_4_GR_ChDB_0036 | Doxorubicin | 3uM | 6h | PTPN12 | 0.00 | 0.38 |
| HT29_4_GR_ChDB_0036 | Doxorubicin | 3uM | 6h | TNFRSF11A | 0.00 | 0.37 |
| HT29_4_GR_ChDB_0036 | Doxorubicin | 3uM | 6h | FBXO5 | 0.00 | 2.14 |
| HT29_4_GR_ChDB_0036 | Doxorubicin | 3uM | 6h | BTG1 | 0.00 | 3.14 |
| HT29_4_GR_ChDB_0036 | Doxorubicin | 3uM | 6h | PDS5B | 0.00 | 0.36 |
| HT29_4_GR_ChDB_0036 | Doxorubicin | 3uM | 6h | ALAD | 0.00 | 0.47 |
| HT29_4_GR_ChDB_0036 | Doxorubicin | 3uM | 6h | ATG4A | 0.00 | 2.32 |
| HT29_4_GR_ChDB_0036 | Doxorubicin | 3uM | 6h | DOK4 | 0.00 | 0.50 |
| HT29_4_GR_ChDB_0036 | Doxorubicin | 3uM | 6h | TIPARP | 0.00 | 0.41 |
| HT29_4_GR_ChDB_0036 | Doxorubicin | 3uM | 6h | CCNA2 | 0.00 | 0.37 |
| HT29_4_GR_ChDB_0036 | Doxorubicin | 3uM | 6h | BIRC5 | 0.00 | 2.00 |
| HT29_4_GR_ChDB_0036 | Doxorubicin | 3uM | 6h | CXCR4 | 0.00 | 3.72 |
| HT29_4_GR_ChDB_0036 | Doxorubicin | 3uM | 6h | PIK3R1 | 0.00 | 0.34 |
| HT29_4_GR_ChDB_0036 | Doxorubicin | 3uM | 6h | PHF15 | 0.00 | 0.48 |
| HT29_4_GR_ChDB_0036 | Doxorubicin | 3uM | 6h | POLR3B | 0.00 | 0.40 |
| HT29_4_GR_ChDB_0036 | Doxorubicin | 3uM | 6h | PISD | 0.00 | 0.39 |
| HT29_4_GR_ChDB_0036 | Doxorubicin | 3uM | 6h | GNA12 | 0.00 | 0.40 |
| HT29_4_GR_ChDB_0036 | Doxorubicin | 3uM | 6h | PPFIBP2 | 0.00 | 0.46 |
| HT29_4_GR_ChDB_0036 | Doxorubicin | 3uM | 6h | SOX4 | 0.00 | 0.40 |
| HT29_4_GR_ChDB_0036 | Doxorubicin | 3uM | 6h | GATA6 | 0.00 | 0.35 |
| HT29_4_GR_ChDB_0036 | Doxorubicin | 3uM | 6h | HMGA2 | 0.00 | 0.30 |
| HT29_4_GR_ChDB_0036 | Doxorubicin | 3uM | 6h | TLE1 | 0.00 | 0.47 |
| HT29_4_GR_ChDB_0036 | Doxorubicin | 3uM | 6h | GPR110 | 0.00 | 0.42 |
| HT29_4_GR_ChDB_0036 | Doxorubicin | 3uM | 6h | PRR15L | 0.00 | 0.35 |
| HT29_4_GR_ChDB_0036 | Doxorubicin | 3uM | 6h | KIF11 | 0.00 | 0.31 |
| HT29_4_GR_ChDB_0036 | Doxorubicin | 3uM | 6h | NR4A2 | 0.00 | 2.50 |
| HT29_4_GR_ChDB_0036 | Doxorubicin | 3uM | 6h | PEX13 | 0.00 | 2.27 |
| HT29_4_GR_ChDB_0036 | Doxorubicin | 3uM | 6h | ARHGEF7 | 0.00 | 0.36 |
| HT29_4_GR_ChDB_0036 | Doxorubicin | 3uM | 6h | CREB1 | 0.00 | 0.30 |
| HT29_4_GR_ChDB_0036 | Doxorubicin | 3uM | 6h | NR2F6 | 0.00 | 0.48 |
| HT29_4_GR_ChDB_0036 | Doxorubicin | 3uM | 6h | CDC42SE1 | 0.00 | 0.37 |
| HT29_4_GR_ChDB_0036 | Doxorubicin | 3uM | 6h | SLC38A1 | 0.00 | 0.50 |
| HT29_4_GR_ChDB_0036 | Doxorubicin | 3uM | 6h | ETS2 | 0.00 | 0.17 |
| HT29_4_GR_ChDB_0036 | Doxorubicin | 3uM | 6h | BTBD3 | 0.00 | 0.40 |
| HT29_4_GR_ChDB_0036 | Doxorubicin | 3uM | 6h | IL8 | 0.00 | 0.46 |
| HT29_4_GR_ChDB_0036 | Doxorubicin | 3uM | 6h | FOSL2 | 0.00 | 0.44 |
| HT29_4_GR_ChDB_0036 | Doxorubicin | 3uM | 6h | DUSP2 | 0.00 | 0.48 |
| HT29_4_GR_ChDB_0036 | Doxorubicin | 3uM | 6h | GSTZ1 | 0.00 | 2.24 |
| HT29_4_GR_ChDB_0036 | Doxorubicin | 3uM | 6h | FAM104A | 0.00 | 0.36 |
| HT29_4_GR_ChDB_0036 | Doxorubicin | 3uM | 6h | BCL3 | 0.00 | 0.46 |
| HT29_4_GR_ChDB_0036 | Doxorubicin | 3uM | 6h | CHKA | 0.00 | 0.44 |
| HT29_4_GR_ChDB_0036 | Doxorubicin | 3uM | 6h | BRCA2 | 0.00 | 0.47 |
| HT29_4_GR_ChDB_0036 | Doxorubicin | 3uM | 6h | CREM | 0.00 | 2.29 |
| HT29_4_GR_ChDB_0036 | Doxorubicin | 3uM | 6h | CEBPG | 0.00 | 0.49 |
| HT29_4_GR_ChDB_0036 | Doxorubicin | 3uM | 6h | EIF4E | 0.00 | 0.34 |
| HT29_4_GR_ChDB_0036 | Doxorubicin | 3uM | 6h | PRKACB | 0.00 | 0.45 |
| HT29_4_GR_ChDB_0036 | Doxorubicin | 3uM | 6h | RIPK1 | 0.00 | 0.49 |
| HT29_4_GR_ChDB_0036 | Doxorubicin | 3uM | 6h | HMOX1 | 0.00 | 0.28 |
| HT29_4_GR_ChDB_0036 | Doxorubicin | 3uM | 6h | BAMBI | 0.00 | 2.62 |
| HT29_4_GR_ChDB_0036 | Doxorubicin | 3uM | 6h | HOXC4 | 0.00 | 0.48 |
| HT29_4_GR_ChDB_0036 | Doxorubicin | 3uM | 6h | TNFRSF1A | 0.00 | 0.36 |
| HT29_4_GR_ChDB_0036 | Doxorubicin | 3uM | 6h | TRIM16 | 0.00 | 0.39 |
| HT29_4_GR_ChDB_0036 | Doxorubicin | 3uM | 6h | ZNF394 | 0.00 | 2.04 |
| HT29_4_GR_ChDB_0036 | Doxorubicin | 3uM | 6h | ASCC3 | 0.00 | 0.43 |
| HT29_4_GR_ChDB_0036 | Doxorubicin | 3uM | 6h | NIPBL | 0.00 | 0.46 |
| HT29_4_GR_ChDB_0036 | Doxorubicin | 3uM | 6h | SMAD6 | 0.00 | 0.40 |
| HT29_4_GR_ChDB_0037 | Etoposide | 30uM | 6h | MYC | 1.00 | 0.46 |
| HT29_4_GR_ChDB_0037 | Etoposide | 30uM | 6h | AURKA | 0.39 | 0.36 |
| HT29_4_GR_ChDB_0037 | Etoposide | 30uM | 6h | TCF7L2 | 0.21 | 0.36 |
| HT29_4_GR_ChDB_0037 | Etoposide | 30uM | 6h | PPARG | 0.17 | 0.38 |
| HT29_4_GR_ChDB_0037 | Etoposide | 30uM | 6h | ADM | 0.14 | 2.56 |
| HT29_4_GR_ChDB_0037 | Etoposide | 30uM | 6h | STK39 | 0.08 | 0.42 |
| HT29_4_GR_ChDB_0037 | Etoposide | 30uM | 6h | WDR61 | 0.05 | 0.47 |
| HT29_4_GR_ChDB_0037 | Etoposide | 30uM | 6h | HMMR | 0.04 | 0.40 |
| HT29_4_GR_ChDB_0037 | Etoposide | 30uM | 6h | BCR | 0.04 | 0.48 |
| HT29_4_GR_ChDB_0037 | Etoposide | 30uM | 6h | CCNE1 | 0.03 | 2.11 |
| HT29_4_GR_ChDB_0037 | Etoposide | 30uM | 6h | KDM4C | 0.03 | 0.47 |
| HT29_4_GR_ChDB_0037 | Etoposide | 30uM | 6h | ZFX | 0.03 | 0.39 |
| HT29_4_GR_ChDB_0037 | Etoposide | 30uM | 6h | CREM | 0.02 | 2.26 |
| HT29_4_GR_ChDB_0037 | Etoposide | 30uM | 6h | LGR5 | 0.02 | 0.37 |
| HT29_4_GR_ChDB_0037 | Etoposide | 30uM | 6h | IL8 | 0.02 | 2.15 |
| HT29_4_GR_ChDB_0037 | Etoposide | 30uM | 6h | ITGA2 | 0.01 | 0.50 |
| HT29_4_GR_ChDB_0037 | Etoposide | 30uM | 6h | ATG4A | 0.01 | 2.44 |
| HT29_4_GR_ChDB_0037 | Etoposide | 30uM | 6h | KIF20A | 0.01 | 0.28 |
| HT29_4_GR_ChDB_0037 | Etoposide | 30uM | 6h | NTSR1 | 0.00 | 0.47 |
| HT29_4_GR_ChDB_0037 | Etoposide | 30uM | 6h | SMAD3 | 0.00 | 0.45 |
| HT29_4_GR_ChDB_0037 | Etoposide | 30uM | 6h | BIK | 0.00 | 2.23 |
| HT29_4_GR_ChDB_0037 | Etoposide | 30uM | 6h | HMGA2 | 0.00 | 0.38 |
| HT29_4_GR_ChDB_0037 | Etoposide | 30uM | 6h | CTSL2 | 0.00 | 2.26 |
| HT29_4_GR_ChDB_0037 | Etoposide | 30uM | 6h | TNIK | 0.00 | 0.49 |
| HT29_4_GR_ChDB_0037 | Etoposide | 30uM | 6h | PIK3R1 | 0.00 | 0.42 |
| HT29_4_GR_ChDB_0037 | Etoposide | 30uM | 6h | CDC20 | 0.00 | 0.48 |
| HT29_4_GR_ChDB_0037 | Etoposide | 30uM | 6h | GTSE1 | 0.00 | 0.43 |
| HT29_4_GR_ChDB_0037 | Etoposide | 30uM | 6h | DDIT4 | 0.00 | 2.23 |
| HT29_4_GR_ChDB_0037 | Etoposide | 30uM | 6h | PDE6D | 0.00 | 2.03 |
| HT29_4_GR_ChDB_0037 | Etoposide | 30uM | 6h | LPAR2 | 0.00 | 2.19 |
| HT29_4_GR_ChDB_0037 | Etoposide | 30uM | 6h | E2F1 | 0.00 | 2.05 |
| HT29_4_GR_ChDB_0037 | Etoposide | 30uM | 6h | ULBP2 | 0.00 | 2.60 |
| HT29_4_GR_ChDB_0037 | Etoposide | 30uM | 6h | DEPDC1 | 0.00 | 0.34 |
| HT29_4_GR_ChDB_0037 | Etoposide | 30uM | 6h | ASPM | 0.00 | 0.43 |
| HT29_4_GR_ChDB_0037 | Etoposide | 30uM | 6h | DLGAP5 | 0.00 | 0.47 |
| HT29_4_GR_ChDB_0037 | Etoposide | 30uM | 6h | EIF3B | 0.00 | 0.45 |
| HT29_4_GR_ChDB_0037 | Etoposide | 30uM | 6h | KIF14 | 0.00 | 0.47 |
| HT29_4_GR_ChDB_0037 | Etoposide | 30uM | 6h | TARDBP | 0.00 | 0.48 |
| HT29_4_GR_ChDB_0037 | Etoposide | 30uM | 6h | PTPN2 | 0.00 | 0.45 |
| HT29_4_GR_ChDB_0037 | Etoposide | 30uM | 6h | SPDEF | 0.00 | 0.48 |
| HT29_4_GR_ChDB_0037 | Etoposide | 30uM | 6h | PNRC1 | 0.00 | 2.72 |
| HT29_4_GR_ChDB_0037 | Etoposide | 30uM | 6h | MEGF9 | 0.00 | 2.11 |
| HT29_4_GR_ChDB_0037 | Etoposide | 30uM | 6h | MBNL1 | 0.00 | 0.43 |
| HT29_4_GR_ChDB_0037 | Etoposide | 30uM | 6h | SIPA1L3 | 0.00 | 0.49 |
| HT29_4_GR_ChDB_0037 | Etoposide | 30uM | 6h | CD97 | 0.00 | 0.47 |
| HT29_4_GR_ChDB_0037 | Etoposide | 30uM | 6h | CCNB1 | 0.00 | 0.38 |
| HT29_4_GR_ChDB_0037 | Etoposide | 30uM | 6h | LRCH1 | 0.00 | 0.47 |
| HT29_4_GR_ChDB_0037 | Etoposide | 30uM | 6h | CTBP2 | 0.00 | 0.49 |
| HT29_4_GR_ChDB_0037 | Etoposide | 30uM | 6h | CENPE | 0.00 | 0.39 |
| HT29_4_GR_ChDB_0037 | Etoposide | 30uM | 6h | PSRC1 | 0.00 | 0.35 |
| HT29_4_GR_ChDB_0037 | Etoposide | 30uM | 6h | IGF2BP3 | 0.00 | 0.50 |
| HT29_4_GR_ChDB_0037 | Etoposide | 30uM | 6h | PHF1 | 0.00 | 2.00 |
| HT29_4_GR_ChDB_0037 | Etoposide | 30uM | 6h | IL17RB | 0.00 | 2.27 |
| HT29_4_GR_ChDB_0037 | Etoposide | 30uM | 6h | CCNF | 0.00 | 0.49 |
| HT29_4_GR_ChDB_0037 | Etoposide | 30uM | 6h | GNAQ | 0.00 | 0.49 |
| HT29_4_GR_ChDB_0037 | Etoposide | 30uM | 6h | PRR11 | 0.00 | 0.39 |
| HT29_4_GR_ChDB_0037 | Etoposide | 30uM | 6h | GPSM2 | 0.00 | 0.50 |
| HT29_4_GR_ChDB_0037 | Etoposide | 30uM | 6h | ELOVL6 | 0.00 | 0.44 |
| HT29_4_GR_ChDB_0037 | Etoposide | 30uM | 6h | PTPRK | 0.00 | 0.39 |
| HT29_4_GR_ChDB_0037 | Etoposide | 30uM | 6h | SLC1A4 | 0.00 | 2.20 |
| HT29_4_GR_ChDB_0037 | Etoposide | 30uM | 6h | DUSP1 | 0.00 | 2.04 |
| HT29_4_GR_ChDB_0037 | Etoposide | 30uM | 6h | EIF4G3 | 0.00 | 0.42 |
| HT29_4_GR_ChDB_0037 | Etoposide | 30uM | 6h | OSBPL10 | 0.00 | 0.45 |
| HT29_4_GR_ChDB_0037 | Etoposide | 30uM | 6h | SLC25A37 | 0.00 | 0.49 |
| HT29_4_GR_ChDB_0037 | Etoposide | 30uM | 6h | STAG1 | 0.00 | 0.43 |
| HT29_4_GR_ChDB_0037 | Etoposide | 30uM | 6h | TNK2 | 0.00 | 0.40 |
| HT29_4_GR_ChDB_0037 | Etoposide | 30uM | 6h | CRCP | 0.00 | 0.45 |
| HT29_4_GR_ChDB_0037 | Etoposide | 30uM | 6h | VAV2 | 0.00 | 0.43 |
| HT29_4_GR_ChDB_0037 | Etoposide | 30uM | 6h | WDR43 | 0.00 | 0.50 |
| HT29_4_GR_ChDB_0037 | Etoposide | 30uM | 6h | MYO19 | 0.00 | 0.46 |
| HT29_4_GR_ChDB_0037 | Etoposide | 30uM | 6h | EREG | 0.00 | 0.42 |
| HT29_4_GR_ChDB_0037 | Etoposide | 30uM | 6h | PRPF38B | 0.00 | 0.48 |
| HT29_4_GR_ChDB_0037 | Etoposide | 30uM | 6h | HNRNPDL | 0.00 | 0.48 |
| HT29_4_GR_ChDB_0037 | Etoposide | 30uM | 6h | KITLG | 0.00 | 0.47 |
| HT29_4_GR_ChDB_0037 | Etoposide | 30uM | 6h | CCDC85C | 0.00 | 0.50 |
| HT29_4_GR_ChDB_0037 | Etoposide | 30uM | 6h | DOCK5 | 0.00 | 0.45 |
| HT29_4_GR_ChDB_0037 | Etoposide | 30uM | 6h | PSD3 | 0.00 | 0.46 |
| HT29_4_GR_ChDB_0037 | Etoposide | 30uM | 6h | WNT11 | 0.00 | 0.40 |
| HT29_4_GR_ChDB_0038 | Mitoxantrone | 3uM | 6h | ADM | 1.00 | 8.11 |
| HT29_4_GR_ChDB_0038 | Mitoxantrone | 3uM | 6h | IGF1R | 0.62 | 0.43 |
| HT29_4_GR_ChDB_0038 | Mitoxantrone | 3uM | 6h | PTK2 | 0.59 | 0.44 |
| HT29_4_GR_ChDB_0038 | Mitoxantrone | 3uM | 6h | MYC | 0.53 | 0.39 |
| HT29_4_GR_ChDB_0038 | Mitoxantrone | 3uM | 6h | TGFBR2 | 0.50 | 0.22 |
| HT29_4_GR_ChDB_0038 | Mitoxantrone | 3uM | 6h | PLXNA1 | 0.38 | 0.32 |
| HT29_4_GR_ChDB_0038 | Mitoxantrone | 3uM | 6h | EGFR | 0.35 | 0.43 |
| HT29_4_GR_ChDB_0038 | Mitoxantrone | 3uM | 6h | BUB1B | 0.32 | 0.21 |
| HT29_4_GR_ChDB_0038 | Mitoxantrone | 3uM | 6h | PIK3R1 | 0.28 | 0.27 |
| HT29_4_GR_ChDB_0038 | Mitoxantrone | 3uM | 6h | ABL1 | 0.22 | 0.44 |
| HT29_4_GR_ChDB_0038 | Mitoxantrone | 3uM | 6h | TRIB3 | 0.14 | 0.34 |
| HT29_4_GR_ChDB_0038 | Mitoxantrone | 3uM | 6h | GSK3B | 0.14 | 0.42 |
| HT29_4_GR_ChDB_0038 | Mitoxantrone | 3uM | 6h | GRB10 | 0.12 | 0.32 |
| HT29_4_GR_ChDB_0038 | Mitoxantrone | 3uM | 6h | BMPR1A | 0.11 | 0.30 |
| HT29_4_GR_ChDB_0038 | Mitoxantrone | 3uM | 6h | MAP3K4 | 0.11 | 0.41 |
| HT29_4_GR_ChDB_0038 | Mitoxantrone | 3uM | 6h | MAX | 0.11 | 0.42 |
| HT29_4_GR_ChDB_0038 | Mitoxantrone | 3uM | 6h | ZMIZ1 | 0.07 | 0.22 |
| HT29_4_GR_ChDB_0038 | Mitoxantrone | 3uM | 6h | SIK1 | 0.07 | 0.44 |
| HT29_4_GR_ChDB_0038 | Mitoxantrone | 3uM | 6h | BTG1 | 0.07 | 4.13 |
| HT29_4_GR_ChDB_0038 | Mitoxantrone | 3uM | 6h | FAM104A | 0.06 | 0.49 |
| HT29_4_GR_ChDB_0038 | Mitoxantrone | 3uM | 6h | HBEGF | 0.05 | 2.82 |
| HT29_4_GR_ChDB_0038 | Mitoxantrone | 3uM | 6h | KIF11 | 0.05 | 0.20 |
| HT29_4_GR_ChDB_0038 | Mitoxantrone | 3uM | 6h | CD58 | 0.05 | 0.49 |
| HT29_4_GR_ChDB_0038 | Mitoxantrone | 3uM | 6h | MECOM | 0.05 | 0.41 |
| HT29_4_GR_ChDB_0038 | Mitoxantrone | 3uM | 6h | SMAD3 | 0.05 | 0.35 |
| HT29_4_GR_ChDB_0038 | Mitoxantrone | 3uM | 6h | MAP4K3 | 0.05 | 0.29 |
| HT29_4_GR_ChDB_0038 | Mitoxantrone | 3uM | 6h | FBXO5 | 0.05 | 2.02 |
| HT29_4_GR_ChDB_0038 | Mitoxantrone | 3uM | 6h | IRS1 | 0.05 | 0.25 |
| HT29_4_GR_ChDB_0038 | Mitoxantrone | 3uM | 6h | TCF7L2 | 0.04 | 0.23 |
| HT29_4_GR_ChDB_0038 | Mitoxantrone | 3uM | 6h | FOS | 0.04 | 2.92 |
| HT29_4_GR_ChDB_0038 | Mitoxantrone | 3uM | 6h | ATF3 | 0.04 | 4.58 |
| HT29_4_GR_ChDB_0038 | Mitoxantrone | 3uM | 6h | GLRX | 0.04 | 3.11 |
| HT29_4_GR_ChDB_0038 | Mitoxantrone | 3uM | 6h | NEU1 | 0.04 | 2.78 |
| HT29_4_GR_ChDB_0038 | Mitoxantrone | 3uM | 6h | PRKCZ | 0.04 | 0.41 |
| HT29_4_GR_ChDB_0038 | Mitoxantrone | 3uM | 6h | EGR1 | 0.04 | 5.73 |
| HT29_4_GR_ChDB_0038 | Mitoxantrone | 3uM | 6h | ID2 | 0.03 | 3.04 |
| HT29_4_GR_ChDB_0038 | Mitoxantrone | 3uM | 6h | MAP3K5 | 0.03 | 0.41 |
| HT29_4_GR_ChDB_0038 | Mitoxantrone | 3uM | 6h | XBP1 | 0.03 | 0.43 |
| HT29_4_GR_ChDB_0038 | Mitoxantrone | 3uM | 6h | EPHB4 | 0.03 | 0.40 |
| HT29_4_GR_ChDB_0038 | Mitoxantrone | 3uM | 6h | PUM2 | 0.03 | 0.26 |
| HT29_4_GR_ChDB_0038 | Mitoxantrone | 3uM | 6h | HEXIM1 | 0.03 | 7.45 |
| HT29_4_GR_ChDB_0038 | Mitoxantrone | 3uM | 6h | CTCF | 0.03 | 0.42 |
| HT29_4_GR_ChDB_0038 | Mitoxantrone | 3uM | 6h | CCNA2 | 0.03 | 0.33 |
| HT29_4_GR_ChDB_0038 | Mitoxantrone | 3uM | 6h | MAP3K8 | 0.03 | 0.45 |
| HT29_4_GR_ChDB_0038 | Mitoxantrone | 3uM | 6h | GTF2B | 0.02 | 3.38 |
| HT29_4_GR_ChDB_0038 | Mitoxantrone | 3uM | 6h | DGKD | 0.02 | 0.35 |
| HT29_4_GR_ChDB_0038 | Mitoxantrone | 3uM | 6h | CLK2 | 0.02 | 0.40 |
| HT29_4_GR_ChDB_0038 | Mitoxantrone | 3uM | 6h | GRK6 | 0.02 | 0.43 |
| HT29_4_GR_ChDB_0038 | Mitoxantrone | 3uM | 6h | NRIP1 | 0.02 | 0.31 |
| HT29_4_GR_ChDB_0038 | Mitoxantrone | 3uM | 6h | TRAFD1 | 0.02 | 2.21 |
| HT29_4_GR_ChDB_0038 | Mitoxantrone | 3uM | 6h | NAT1 | 0.02 | 4.73 |
| HT29_4_GR_ChDB_0038 | Mitoxantrone | 3uM | 6h | SFMBT1 | 0.02 | 0.47 |
| HT29_4_GR_ChDB_0038 | Mitoxantrone | 3uM | 6h | SAT1 | 0.02 | 3.59 |
| HT29_4_GR_ChDB_0038 | Mitoxantrone | 3uM | 6h | DUSP14 | 0.02 | 0.49 |
| HT29_4_GR_ChDB_0038 | Mitoxantrone | 3uM | 6h | ZKSCAN1 | 0.02 | 2.07 |
| HT29_4_GR_ChDB_0038 | Mitoxantrone | 3uM | 6h | ZBTB24 | 0.02 | 0.46 |
| HT29_4_GR_ChDB_0038 | Mitoxantrone | 3uM | 6h | TBK1 | 0.02 | 0.32 |
| HT29_4_GR_ChDB_0038 | Mitoxantrone | 3uM | 6h | MAD2L1BP | 0.02 | 2.30 |
| HT29_4_GR_ChDB_0038 | Mitoxantrone | 3uM | 6h | TP53 | 0.02 | 2.16 |
| HT29_4_GR_ChDB_0038 | Mitoxantrone | 3uM | 6h | FBXL12 | 0.02 | 2.06 |
| HT29_4_GR_ChDB_0038 | Mitoxantrone | 3uM | 6h | STK10 | 0.02 | 0.45 |
| HT29_4_GR_ChDB_0038 | Mitoxantrone | 3uM | 6h | CUL4B | 0.02 | 2.47 |
| HT29_4_GR_ChDB_0038 | Mitoxantrone | 3uM | 6h | RAD50 | 0.02 | 0.46 |
| HT29_4_GR_ChDB_0038 | Mitoxantrone | 3uM | 6h | PAFAH1B1 | 0.02 | 0.18 |
| HT29_4_GR_ChDB_0038 | Mitoxantrone | 3uM | 6h | ZFX | 0.02 | 0.32 |
| HT29_4_GR_ChDB_0038 | Mitoxantrone | 3uM | 6h | HERPUD1 | 0.02 | 0.41 |
| HT29_4_GR_ChDB_0038 | Mitoxantrone | 3uM | 6h | PPARG | 0.02 | 0.38 |
| HT29_4_GR_ChDB_0038 | Mitoxantrone | 3uM | 6h | GADD45B | 0.02 | 6.16 |
| HT29_4_GR_ChDB_0038 | Mitoxantrone | 3uM | 6h | NFIC | 0.02 | 0.39 |
| HT29_4_GR_ChDB_0038 | Mitoxantrone | 3uM | 6h | DUSP6 | 0.02 | 2.03 |
| HT29_4_GR_ChDB_0038 | Mitoxantrone | 3uM | 6h | SMURF1 | 0.02 | 0.21 |
| HT29_4_GR_ChDB_0038 | Mitoxantrone | 3uM | 6h | EDN1 | 0.01 | 0.32 |
| HT29_4_GR_ChDB_0038 | Mitoxantrone | 3uM | 6h | SPEN | 0.01 | 0.49 |
| HT29_4_GR_ChDB_0038 | Mitoxantrone | 3uM | 6h | LIMK2 | 0.01 | 0.38 |
| HT29_4_GR_ChDB_0038 | Mitoxantrone | 3uM | 6h | FGFR2 | 0.01 | 0.41 |
| HT29_4_GR_ChDB_0038 | Mitoxantrone | 3uM | 6h | CDCA4 | 0.01 | 0.25 |
| HT29_4_GR_ChDB_0038 | Mitoxantrone | 3uM | 6h | CTSL2 | 0.01 | 2.36 |
| HT29_4_GR_ChDB_0038 | Mitoxantrone | 3uM | 6h | GMEB1 | 0.01 | 0.40 |
| HT29_4_GR_ChDB_0038 | Mitoxantrone | 3uM | 6h | DYRK1A | 0.01 | 0.49 |
| HT29_4_GR_ChDB_0038 | Mitoxantrone | 3uM | 6h | NDRG1 | 0.01 | 4.04 |
| HT29_4_GR_ChDB_0038 | Mitoxantrone | 3uM | 6h | BCL6 | 0.01 | 4.41 |
| HT29_4_GR_ChDB_0038 | Mitoxantrone | 3uM | 6h | FOXO1 | 0.01 | 0.31 |
| HT29_4_GR_ChDB_0038 | Mitoxantrone | 3uM | 6h | CENPE | 0.01 | 0.25 |
| HT29_4_GR_ChDB_0038 | Mitoxantrone | 3uM | 6h | BRCA2 | 0.01 | 0.48 |
| HT29_4_GR_ChDB_0038 | Mitoxantrone | 3uM | 6h | DGKE | 0.01 | 0.41 |
| HT29_4_GR_ChDB_0038 | Mitoxantrone | 3uM | 6h | PIK3R3 | 0.01 | 3.46 |
| HT29_4_GR_ChDB_0038 | Mitoxantrone | 3uM | 6h | SRC | 0.01 | 0.49 |
| HT29_4_GR_ChDB_0038 | Mitoxantrone | 3uM | 6h | ROCK2 | 0.01 | 0.39 |
| HT29_4_GR_ChDB_0038 | Mitoxantrone | 3uM | 6h | TJP1 | 0.01 | 0.48 |
| HT29_4_GR_ChDB_0038 | Mitoxantrone | 3uM | 6h | NFIB | 0.01 | 0.39 |
| HT29_4_GR_ChDB_0038 | Mitoxantrone | 3uM | 6h | HMGB3 | 0.01 | 0.43 |
| HT29_4_GR_ChDB_0038 | Mitoxantrone | 3uM | 6h | MED7 | 0.01 | 0.37 |
| HT29_4_GR_ChDB_0038 | Mitoxantrone | 3uM | 6h | DDX10 | 0.01 | 0.48 |
| HT29_4_GR_ChDB_0038 | Mitoxantrone | 3uM | 6h | GNB1L | 0.01 | 0.21 |
| HT29_4_GR_ChDB_0038 | Mitoxantrone | 3uM | 6h | E2F5 | 0.01 | 0.29 |
| HT29_4_GR_ChDB_0038 | Mitoxantrone | 3uM | 6h | NLK | 0.01 | 0.28 |
| HT29_4_GR_ChDB_0038 | Mitoxantrone | 3uM | 6h | ERCC6L | 0.01 | 0.40 |
| HT29_4_GR_ChDB_0038 | Mitoxantrone | 3uM | 6h | POLR3B | 0.01 | 0.39 |
| HT29_4_GR_ChDB_0038 | Mitoxantrone | 3uM | 6h | SPTLC2 | 0.01 | 0.44 |
| HT29_4_GR_ChDB_0038 | Mitoxantrone | 3uM | 6h | AKAP1 | 0.01 | 0.49 |
| HT29_4_GR_ChDB_0038 | Mitoxantrone | 3uM | 6h | PFKFB3 | 0.01 | 0.43 |
| HT29_4_GR_ChDB_0038 | Mitoxantrone | 3uM | 6h | COPZ1 | 0.01 | 2.03 |
| HT29_4_GR_ChDB_0038 | Mitoxantrone | 3uM | 6h | DFFB | 0.01 | 0.42 |
| HT29_4_GR_ChDB_0038 | Mitoxantrone | 3uM | 6h | SMURF2 | 0.01 | 0.45 |
| HT29_4_GR_ChDB_0038 | Mitoxantrone | 3uM | 6h | SLC29A2 | 0.01 | 0.36 |
| HT29_4_GR_ChDB_0038 | Mitoxantrone | 3uM | 6h | CDT1 | 0.01 | 0.45 |
| HT29_4_GR_ChDB_0038 | Mitoxantrone | 3uM | 6h | ATP2A2 | 0.01 | 0.48 |
| HT29_4_GR_ChDB_0038 | Mitoxantrone | 3uM | 6h | FOXA1 | 0.01 | 0.40 |
| HT29_4_GR_ChDB_0038 | Mitoxantrone | 3uM | 6h | DOT1L | 0.01 | 0.46 |
| HT29_4_GR_ChDB_0038 | Mitoxantrone | 3uM | 6h | ASCC3 | 0.01 | 0.45 |
| HT29_4_GR_ChDB_0038 | Mitoxantrone | 3uM | 6h | MKNK2 | 0.01 | 2.03 |
| HT29_4_GR_ChDB_0038 | Mitoxantrone | 3uM | 6h | CHCHD7 | 0.01 | 3.25 |
| HT29_4_GR_ChDB_0038 | Mitoxantrone | 3uM | 6h | WTAP | 0.01 | 0.43 |
| HT29_4_GR_ChDB_0038 | Mitoxantrone | 3uM | 6h | MSX2 | 0.01 | 2.52 |
| HT29_4_GR_ChDB_0038 | Mitoxantrone | 3uM | 6h | NAA25 | 0.01 | 0.36 |
| HT29_4_GR_ChDB_0038 | Mitoxantrone | 3uM | 6h | UROD | 0.01 | 2.21 |
| HT29_4_GR_ChDB_0038 | Mitoxantrone | 3uM | 6h | PRKCQ | 0.01 | 0.44 |
| HT29_4_GR_ChDB_0038 | Mitoxantrone | 3uM | 6h | SLC22A18 | 0.01 | 2.15 |
| HT29_4_GR_ChDB_0038 | Mitoxantrone | 3uM | 6h | PTPRK | 0.01 | 0.44 |
| HT29_4_GR_ChDB_0038 | Mitoxantrone | 3uM | 6h | DNAJB1 | 0.01 | 0.38 |
| HT29_4_GR_ChDB_0038 | Mitoxantrone | 3uM | 6h | CSNK1D | 0.01 | 0.37 |
| HT29_4_GR_ChDB_0038 | Mitoxantrone | 3uM | 6h | PNN | 0.01 | 0.35 |
| HT29_4_GR_ChDB_0038 | Mitoxantrone | 3uM | 6h | MTF2 | 0.01 | 0.43 |
| HT29_4_GR_ChDB_0038 | Mitoxantrone | 3uM | 6h | DNAJB9 | 0.01 | 2.11 |
| HT29_4_GR_ChDB_0038 | Mitoxantrone | 3uM | 6h | NXF1 | 0.01 | 2.48 |
| HT29_4_GR_ChDB_0038 | Mitoxantrone | 3uM | 6h | UIMC1 | 0.01 | 0.33 |
| HT29_4_GR_ChDB_0038 | Mitoxantrone | 3uM | 6h | WHSC1 | 0.01 | 0.49 |
[truncated: 106,142 more chars]
